# Sex differences in mortality among children, adolescents, and young people aged 0–24 years: a systematic assessment of national, regional, and global trends from 1990 to 2021

**DOI:** 10.1016/S2214-109X(23)00376-5

**Published:** 2023-09-19

**Authors:** Fengqing Chao, Bruno Masquelier, Danzhen You, Lucia Hug, Yang Liu, David Sharrow, Håvard Rue, Hernando Ombao, Leontine Alkema, UN Inter-agency Group for Child Mortality Estimation, Bochen Cao, Victor Gaigbe-Togbe, Thomas Spoorenberg, Kathleen Louise Strong, Emi Suzuki, Danzhen You

**Affiliations:** a Statistics Program, Computer, Electrical and Mathematical Sciences and Engineering Division, King Abdullah University of Science and Technology, Thuwal, Makkah, Saudi Arabia; b University of Louvain, Louvain-la-Neuve, Wallonia, Belgium; c Division of Data, Analytics, Planning and Monitoring, United Nations Children's Fund, New York, NY, USA; d Department of Biostatistics and Epidemiology, School of Public Health and Health Sciences, University of Massachusetts, Amherst, MA, USA

## Abstract

**Background:**

Differences in mortality exist between sexes because of biological, genetic, and social factors. Sex differentials are well documented in children younger than 5 years but have not been systematically examined for ages 5–24 years. We aimed to estimate the sex ratio of mortality from birth to age 24 years and reconstruct trends in sex-specific mortality between 1990 and 2021 for 200 countries, major regions, and the world.

**Methods:**

We compiled comprehensive databases on the mortality sex ratio (ratio of male to female mortality rates) for individuals aged 0–4 years, 5–14 years, and 15–24 years. The databases contain mortality rates from death registration systems, full birth and sibling histories from surveys, and reports on household deaths in censuses. We modelled the sex ratio of age-specific mortality as a function of the mortality in both sexes using Bayesian hierarchical time-series models. We report the levels and trends of sex ratios and estimate the expected female mortality and excess female mortality rates (the difference between the estimated female mortality and the expected female mortality) to identify countries with outlying sex ratios.

**Findings:**

Globally, the mortality sex ratio was 1·13 (ie, boys were more likely to die than girls of the same age) for ages 0–4 years (90% uncertainty interval 1·11 to 1·15) in 2021. This ratio increased with age to 1·16 (1·12 to 1·20) for 5–14 years, reaching 1·65 for 15–24 years (1·52 to 1·75). In all age groups, the global sex ratio of mortality increased between 1990 and 2021, driven by faster declines in female mortality. In 2021, the probability of a newborn male reaching age 25 years was 94·1% (93·7 to 94·4), compared with 95·1% for a newborn female (94·7 to 95·3). We found a disadvantage of females versus males (compared with countries with similar total mortality) in 2021 in five countries for ages 0–4 years (Algeria, Bangladesh, Egypt, India, and Iran), one country (Suriname) for ages 5–14 years, and 13 countries for ages 15–24 years (including Bangladesh and India). We found the reverse pattern (disadvantage of males *vs* females compared with countries of similar total mortality) in one country in ages 0–4 years (Vietnam) and eight countries in ages 15–24 years (including Brazil and Mexico). Globally, the number of excess female deaths from birth to age 24 years was 86 563 (–6059 to 164 000) in 2021, down from 544 636 (453 982 to 633 265) in 1990.

**Interpretation:**

The global sex ratio of mortality for all age groups in the first 25 years of life increased between 1990 and 2021. Targeted interventions should focus on countries with outlying sex ratios of mortality to reduce disparities due to discrimination in health care, nutrition, and violence.

**Funding:**

The Bill & Melinda Gates Foundation, US Agency for International Development, and King Abdullah University of Science and Technology.

## Introduction

Sex differences in mortality can reveal important biological, genetic, and sociocultural factors contributing to health and disease outcomes. In children younger than 5 years, mortality is higher for boys than for girls. Boys are more likely to be born prematurely, and the burden of congenital malformations and respiratory conditions is larger in boys than in girls.^1^, ^2^ For example, infant deaths are characterised by an approximate 50% male excess compared with female deaths in the USA and European countries for several respiratory conditions.^3^ But discriminatory practices related to health care and nutrition also play a role and can reduce increase mortality for girls.^4^, ^5^, ^6^ Strong preferences for male births over female births have been documented in many parts of the world, leading to skewed sex ratios at birth and neglect of girls.^7^ A study done in 57 low-income and middle-income countries found evidence of gender biases in care seeking among caregivers against common childhood illnesses; girls were significantly less likely to receive adequate care than boys in six countries (including India), whereas the reverse was true in only two countries.^8^

Establishing what the sex ratio of mortality (ratio of male to female mortality) should be in the absence of any gender bias is difficult, as this reference varies by age and changes over time. According to a 2014 study, the estimated sex ratio (the ratio of male to female mortality rates) was globally higher (1·13) in infants younger than 1 year in 2012 than in children aged 1–4 years (0·95).^6^ The sex ratio of under-5 mortality also tends to increase over time as mortality rates for both sexes decrease.^6^ This relationship exists because the mortality decline is associated with a concentration of deaths closer to the period immediately after birth, where cause-of-death patterns are particularly disadvantageous to boys.^5^ Alkema and colleagues^6^ used this relationship between the mortality level of both sexes and the sex ratio to identify countries with outlying sex ratios. The authors identified 15 countries where girls’ mortality was higher than expected in 2012, compared with countries with the same mortality levels, and these countries were primarily in south Asia (Afghanistan, Bangladesh, India, Nepal, and Pakistan) and China. In another study, Costa and colleagues^9^ tried to explain the differences between the observed and expected sex ratios of mortality in children younger than 5 years. They found no association with national wealth, women's characteristics, or gender inequality indices. Still, in countries where there was some evidence of a mortality bias against girls, boys were also more likely to receive care compared with girls.^9^

**Research in context**

**Evidence before this study**

We searched PubMed with the terms “children” [OR] “adolescents” [OR] “youth” [AND] “mortality” [AND] “sex differences” [OR] “sex ratio” for papers published between Jan 1, 1970, and June 6, 2023, in English or French. Existing studies on sex ratios in all-cause mortality primarily concentrated on neonates and children younger than 5 years. The sex ratios in mortality have not been systematically examined in older children, adolescents, and young people aged 5–24 years on the basis of empirical measurements referring specifically to this age range.

**Added value of this study**

We systematically assessed the sex ratios of mortality rates in the early life course, from birth to age 24 years, for 200 countries and territories from 1990 to 2021. We compiled three extensive databases, including publicly available data sources for children (aged 0–4 years), adolescents (5–14 years), and young people (15–24 years). We modelled the relationship between the sex ratio and total mortality rate for each age group. This study provides insight into levels and trends in sex ratios of mortality and pinpoints countries with outlying sex ratios. We showed that chances of survival up to age 24 years tend to improve more rapidly for girls than boys as total mortality decreases, with a reversal of this trend at very low mortality.

**Implications of all the available evidence**

Further research should focus on explaining differences across countries and regions, and shed light on the contribution of cause-specific mortality to these sex differences. Targeted interventions or legislation should be taken to reduce sex disparities due to discrimination or excessive exposure to violence.

Sex differences in mortality extend beyond age 5 years but have been less studied in older children and adolescents aged 5–14 years or youth aged 15–24 years. This gap is presumably because most deaths in these age groups occur in low-income and middle-income countries (97·7% in 2021; 90% uncertainty interval [UI] 97·5–97·9),^10^ where death registration systems are often incomplete. Surveys and censuses are used in the absence of death registration, but they tend to provide inconsistent estimates of sex ratios because of sampling and non-sampling errors. Yet, disaggregating mortality indicators by sex for older children, adolescents, and youth is crucial as these groups comprise about a third of the global population and account for a large proportion of premature deaths. In 2021, 2·1 million deaths occurred in individuals aged 5–24 years, and most were preventable.^11^, ^12^ Disaggregating mortality by sex also helps to target interventions to address specific causes of death. For example, self-harm is the leading cause of death among females aged 15–19 years, whereas for males aged 15–19 years, road traffic injuries are the leading cause of death.^12^ Previous research on sex differences among older children and adolescents has focused on specific age groups (often aged 10–19 years) and was based on varying methodologies across age groups and particular data.^13^, ^14^, ^15^ Ward and colleagues^13^ analysed sex differences in survival in ages 10–24 years using estimates from the Global Burden of Diseases, Injuries, and Risk Factors Study (GBD).^13^ The authors observed that 61% of deaths at these ages in 2019 occurred in males and that sex differences have increased in recent decades because of a more rapid decline in female mortality since 1950 (a decrease of 30% among females *vs* 15% among males). However, Ward and colleagues’^13^ estimates of sex differences in mortality in the population aged 10–24 years were not data driven; the estimates were inferred from a combination of differences in survival in those younger than 5 years and the mortality in those aged 15–60 years, including model age patterns of mortality and covariates.^16^ Moreover, Ward and colleagues did not produce UIs regarding sex ratios and could not pinpoint countries with outlying sex ratios of mortality.

In this study, we aimed to estimate the levels and trends in the sex ratio of mortality by age group from birth to the 25th birthday (hence 0–24 completed years) for 200 countries and territories from 1990 to 2021. In each age group, we assessed the relationship between sex ratios of mortality and national-level mortality using data across all country-years. We used the national-level mortality estimates^10^, ^11^, ^17^ to construct sex-specific mortality rates for all country-years, including those without empirical data. Instead of treating observations equally, we produced more comprehensive estimates of sex ratios because such estimates follow higher-quality data closer than lower-quality data. Our modelling approach also quantifies the uncertainty around estimates to identify countries where sex ratios are higher or lower than expected.

## Methods

### Database construction

The database for this study was compiled by the UN Inter-agency Group for Child Mortality Estimation (UN IGME). The UN IGME includes members from UNICEF, WHO, the World Bank Group, and the UN Department of Economic and Social Affairs, Population Division. This group compiles and assesses the quality of all available nationally representative data on neonatal, child, youth, and adolescent mortality.^11^ UN IGME produce all-cause mortality estimates that are internationally comparable for older children, adolescents, and youth (aged 5–24 years). Empirical databases and final estimates are updated annually and are available in the public domain.

For this study, we used data from vital registration systems, surveys, and censuses (from 1954–2020). We extracted the sex ratios of mortality rates for six age groups: younger than 1 year, 1–4 years, 5–9 years, 10–14 years, and 15–19 years, and 20–24 years. We report results for three aggregated age groups (0–4 years, 5–14 years, and 15–24 years; aggregation steps in appendix pp 10–11). We provide full details on all used data sources, broken down by country and age group (appendix pp 69–200). The construction of the databases for both sexes has been detailed elsewhere.^17^, ^18^ The extraction of sex ratios from all data sources are detailed in the appendix (p 2). Ethics approval was not required for this secondary population-level data analysis. The inclusion criteria for the sex ratio observations followed the same criteria as for observations of total mortality for each age group. Extreme observations (less than 2·4% of the total number of observations in each age group) were removed (appendix p 2).

### Statistical analysis

#### Estimating sex ratios

For sex ratios in mortality in children younger than 5 years, we used the model detailed by Alkema and colleagues.^6^ Above age 5 years, we adapted the model because the database for older age groups contains only direct estimates, which can be disaggregated by age to make the modelling approach more efficient. Both models, for mortality below and above age 5 years, use the sex ratio from empirical data and the estimates of national-level mortality rates (for females and males combined) published by the UN IGME in 2023 as inputs.^11^ Details on the model specification, implementation, and validation are presented in the appendix (pp 4–14).

Country-specific sex ratios were modelled using the product of the expected sex ratio (based on the global model and total mortality rate in the country-year) and a country-specific multiplier, capturing the temporal effect and representing the relative difference in sex ratio compared with other countries at similar total mortality rates. These multipliers were modelled with a national-level function in which country-specific levels were assumed to fluctuate around 1. The Bayesian hierarchical model estimates the global sex ratio, given the total mortality rates and within-country temporal fluctuations simultaneously, allowing the two model elements to inform each other.

The data quality model incorporated (1) stochastic and sampling variance caused by too few person-years and deaths or by small samples and (2) non-sampling variance, which is assumed to differ across data sources. The Bayesian estimates are pooled towards informative observations and are less influenced by weakly informative observations.

#### Computation and model performance assessment

We used the integrated nested Laplace approximation (INLA) algorithm to generate samples from the posterior distribution of the parameters.^19^ This approach produced a set of trajectories of national sex ratios for all age groups and associated measures of sex-specific mortality, excess female mortality, and sex-specific deaths. Estimates of the final sex ratios were combined with national-level mortality rate estimates to obtain country-year, sex-specific mortality rates, accounting for the uncertainty in the national-level mortality rates.^11^ The sex-specific mortality rates and related deaths were adjusted to account for excess crisis-related deaths if a national crisis was identified (appendix pp 11–12).

We derived aggregated mortality rates by sex by applying the proportions of sex-specific deaths within a region to the aggregated UN IGME mortality rate in a region.^11^ We followed the UN IGME protocols and computed 90% UIs for all indicators of interest using the 5th and 95th percentiles of the posterior distributions.

We assessed model performance for each age group through an out-of-sample validation by leaving out around 20% of the data points (appendix pp 13–14). The validation results suggested that the proposed model was reasonably well calibrated, with generally conservative UIs (ie, wider than expected). The INLA algorithm was implemented using the R package INLA (version 22.05.07),^20^ and the analysis was done in R (version 4.1.0).

#### Calculation of excess deaths and identification of outlying sex ratios

We used male mortality as a reference level for each country-year to compute the expected and estimated female mortality based on the total mortality and expected and estimated sex ratios. We defined and calculated excess female mortality as the difference between the expected and estimated female mortality rate for the country-year (where a negative excess female mortality is equivalent to lower-than-expected female mortality). We considered the sex ratio for a country-year outlying if two conditions hold: (1) the posterior probability that excess female mortality is greater or less than zero is at least 95% and (2) the absolute value of the median excess female mortality is greater than one death per 1000 population.

#### Country consultation

A joint WHO and UNICEF country consultation was conducted in 2022 with the 200 countries for which UN IGME estimates were produced, to allow representatives of the Ministry of Health, National Statistics Office or other responsible government agencies to review the data inputs, the methodology and the draft estimates and provide additional data.

### Role of the funding source

The funder of the study had no role in study design, data collection, data analysis, data interpretation, or writing of the report.

## Results

We report sex ratios with median estimates and 90% UIs for 200 countries, seven regions, and worldwide in 1990 and 2021, with results disaggregated by age groups (appendix pp 24–68).

Table 1 displays sex ratios for the age groups 0–4 years, 5–14 years, and 15–24 years for 1990 and 2021 for the world and UNICEF regions (the regional classification is in the appendix p 16). Globally, the sex ratio in 2021 was 1·13 for ages 0–4 years (90% UI 1·11–1·15), and increased as children and adolescents aged, from 1·16 (1·12–1·20) in children aged 5–14 years, to 1·65 (1·52–1·75) in young people aged 15–24 years (table 1). In 2021, there was little regional variation in the sex ratio for children younger than 5 years, ranging from 1·05 (1·01–1·09) in south Asia to 1·24 (1·21–1·27) in Europe and central Asia (table 1). In 2021, the sex ratio was higher in older children and adolescents aged 5–14 years than in children younger than 5 years in all regions except sub-Saharan Africa (table 1). We found an increase the sex ratio in all regions from age 5–14 years to 15–24 years (table 1). We also found a wider geographical variation in this older age group; the sex ratio among young people aged 15–24 years was 1·30 (1·17–1·42) in sub-Saharan Africa and 1·44 (1·21–1·70) in south Asia, but was greater than 2·00 in all other regions and reached 3·33 (3·05–3·54) in Latin America and the Caribbean (table 1).

**Table 1:** Estimates and 90% UIs for sex ratios and ratios of estimated to expected female mortality

	**Sex ratio**			**Estimated to expected female mortality ratio**		
	1990	2021	Change (1990–2021)	1990	2021	Change (1990–2021)
**Probability of dying aged 0–4 years**						
World *†	1·06 (1·05 to 1·07)	1·13 (1·11 to 1·15)	0·07 (0·05 to 0·09)‡	1·06 (1·05 to 1·07)§	1·03 (1·01 to 1·05)§	−0·03 (−0·06 to −0·01)‡
South Asia*†	0·97 (0·95 to 0·99)	1·05 (1·01 to 1·09)	0·08 (0·04 to 0·12)‡	1·16 (1·13 to 1·18)§	1·16 (1·11 to 1·20)§	0·00 (−0·05 to 0·05)
Europe and central Asia	1·21 (1·19 to 1·23)	1·24 (1·21 to 1·27)	0·03 (0·00 to 0·06)‡	0·99 (0·97 to 1·01)	0·98 (0·96 to 1·00)	−0·01 (−0·03 to 0·02)
Middle East and north Africa*	1·07 (1·05 to 1·08)	1·16 (1·12 to 1·20)	0·10 (0·05 to 0·14)‡	1·10 (1·08 to 1·12)§	1·05 (1·00 to 1·09)	−0·05 (−0·10 to −0·01)‡
Sub-Saharan Africa*	1·11 (1·10 to 1·12)	1·16 (1·13 to 1·18)	0·05 (0·02 to 0·08)‡	0·98 (0·97 to 0·99)§	0·98 (0·96 to 1·01)	0·00 (−0·02 to 0·03)
Latin America and the Caribbean	1·18 (1·15 to 1·22)	1·22 (1·19 to 1·26)	0·04 (0·00 to 0·08)	1·00 (0·97 to 1·03)	1·00 (0·97 to 1·03)	0·00 (−0·03 to 0·03)
East Asia and the Pacific*	1·12 (1·08 to 1·17)	1·20 (1·16 to 1·24)	0·08 (0·02 to 0·13)‡	1·05 (1·01 to 1·09)§	1·01 (0·97 to 1·04)	−0·04 (−0·09 to 0·01)
North America	1·25 (1·24 to 1·27)	1·20 (1·16 to 1·24)	−0·05 (−0·09 to −0·01)‡	0·97 (0·96 to 0·99)§	0·99 (0·96 to 1·03)	0·02 (−0·01 to 0·05)
**Probability of dying aged 5–14 years**						
World	1·06 (1·04 to 1·09)	1·16 (1·12 to 1·20)	0·10 (0·06 to 0·13)‡	1·03 (1·00 to 1·06)§	1·00 (0·96 to 1·04)	−0·03 (−0·06 to 0·01)
South Asia*	0·94 (0·89 to 0·98)	1·27 (1·16 to 1·38)	0·33 (0·21 to 0·45)‡	1·10 (1·05 to 1·17)§	1·13 (1·01 to 1·24)§	0·02 (−0·11 to 0·15)
Europe and central Asia	1·51 (1·47 to 1·54)	1·36 (1·33 to 1·39)	−0·14 (−0·19 to −0·10)‡	0·92 (0·89 to 0·95)§	1·04 (1·02 to 1·06)§	0·12 (0·08 to 0·16)‡
Middle East and north Africa	1·15 (1·11 to 1·19)	1·43 (1·35 to 1·52)	0·28 (0·19 to 0·38)‡	0·99 (0·93 to 1·03)	0·98 (0·87 to 1·06)	0·00 (−0·12 to 0·10)
Sub-Saharan Africa	1·05 (1·01 to 1·09)	1·09 (1·04 to 1·14)	0·04 (−0·03 to 0·10)	0·99 (0·97 to 1·02)	0·97 (0·93 to 1·03)	−0·02 (−0·08 to 0·05)
Latin America and the Caribbean	1·34 (1·31 to 1·37)	1·32 (1·27 to 1·37)	−0·02 (−0·09 to 0·04)	1·02 (0·99 to 1·04)	1·08 (1·02 to 1·13)§	0·06 (0·00 to 0·12)
East Asia and the Pacific	1·23 (1·16 to 1·30)	1·49 (1·37 to 1·61)	0·25 (0·12 to 0·40)‡	1·00 (0·93 to 1·07)	0·95 (0·87 to 1·04)	−0·05 (−0·16 to 0·07)
North America	1·50 (1·46 to 1·53)	1·30 (1·23 to 1·38)	−0·20 (−0·28 to −0·11)‡	1·00 (0·97 to 1·02)	1·05 (0·99 to 1·11)	0·05 (−0·01 to 0·12)
**Probability of dying aged 15–24 years**						
World*	1·29 (1·23 to 1·35)	1·65 (1·52 to 1·75)	0·36 (0·22 to 0·49)‡	1·25 (1·16 to 1·34)§	1·04 (0·89 to 1·16)	−0·21 (−0·38 to −0·07)‡
South Asia*	0·82 (0·76 to 0·89)	1·44 (1·21 to 1·70)	0·61 (0·38 to 0·89)‡	2·47 (2·18 to 2·75)§	1·62 (0·99 to 1·96)	−0·85 (−1·51 to −0·39)‡
Europe and central Asia	2·62 (2·49 to 2·75)	2·20 (2·12 to 2·28)	−0·42 (−0·57 to −0·27)‡	0·88 (0·71 to 0·94)§	1·12 (1·07 to 1·17)§	0·23 (0·16 to 0·41)‡
Middle East and north Africa	1·81 (1·64 to 2·04)	2·72 (2·35 to 3·04)	0·91 (0·44 to 1·27)‡	1·11 (0·67 to 1·32)	1·03 (0·87 to 1·18)	−0·09 (−0·35 to 0·37)
Sub-Saharan Africa	1·06 (0·98 to 1·13)	1·30 (1·17 to 1·42)	0·24 (0·09 to 0·40)‡	0·90 (0·80 to 1·01)	0·96 (0·78 to 1·16)	0·06 (−0·15 to 0·27)
Latin America and the Caribbean	2·34 (2·22 to 2·45)	3·33 (3·05 to 3·54)	0·99 (0·69 to 1·24)‡	0·78 (0·70 to 0·88)§	0·59 (0·51 to 0·68)§	−0·19 (−0·32 to −0·08)‡
East Asia and the Pacific	1·85 (1·62 to 2·09)	2·29 (1·88 to 2·70)	0·44 (−0·05 to 0·93)	1·14 (0·98 to 1·33)	1·05 (0·73 to 1·27)	−0·09 (−0·44 to 0·19)
North America	2·98 (2·92 to 3·03)	2·55 (2·21 to 2·91)	−0·43 (−0·77 to −0·06)‡	0·82 (0·80 to 0·85)§	0·99 (0·87 to 1·14)	0·17 (0·04 to 0·32)‡

UI=uncertainty interval.

* Sex ratio is outlying for 1990.

† Sex ratio is outlying for 2021.

‡ Change is significantly different from zero.

§ Ratio of estimated to expected female mortality is significantly different from one.

In all age groups, the global sex ratio increased significantly from 1990 to 2021, from 1·06 (90% UI 1·05–1·07) to 1·13 (1·11–1·15) in children aged 0–4 years, from 1·06 (1·04–1·09) to 1·16 (1·12–1·20) in the age group 5–14 years, and from 1·29 (1·23–1·35) to 1·65 (1·52–1·75) in young people aged 15–24 years (table 1). In 1990, the sex ratio of mortality was the lowest in south Asia, at levels significantly below 1 from ages 0–24 years (table 1). The south Asia ratios were 0·97 (0·95–0·99) for age 0–4 years, 0·94 (0·89–0·98) for 5–14 years, and 0·82 (0·76–0·89) for 15–24 years (table 1). From 1990 to 2021, sex ratios in south Asia increased significantly in all age groups, with males facing higher risks of dying from birth to age 24 years compared with females (table 1). Despite the increased sex ratios for all age groups, in 2021, south Asia still had the lowest sex ratio in children aged 0–4 years (1·05 [1·01–1·09]) and the second lowest sex ratio in ages 5–14 years (1·27 [1·16–1·38]) and ages 15–24 years (1·44 [1·21–1·70]; table 1). Among the seven regions, the sex ratio increased significantly in three regions among ages 5–14 years, with the largest absolute increase estimated in south Asia (table 1). The sex ratio among young people aged 15–24 years changed significantly in all regions except east Asia and the Pacific (table 1). The greatest absolute increases in youth sex ratios were estimated in Latin America and the Caribbean (from 2·34 [2·22–2·45] in 1990 to 3·33 [3·05–3·54] in 2021) and in the Middle East and north Africa (from 1·81 [1·64–2·04] in 1990 to 2·72 [2·35–3·04] in 2021; table 1). By contrast, North America's sex ratios of mortality declined significantly for all age groups, as they did in Europe and central Asia for ages 5–24 years (table 1).

Overall, in 2021, a newborn male's probability of reaching age 25 years was 94·1% (90% UI 93·7–94·4), compared with 95·1% (94·7–95·3) for a newborn female. The higher risk of male versus female mortality before age 25 years, combined with a higher number of male births than female births, resulted in more deaths in males than females in the population aged 0–24 years in 2021 (table 2). Males accounted for 56·2% (55·7–56·7) of deaths in the age group 0–24 years in 2021, compared with 53·4% (53·2–53·7) in 1990 (table 2). This increased proportion in 2021 is a combination of the increase in sex ratios of mortality over time and the growing concentration of mortality in older age groups. In 2021, among young children aged 0–4 years, between 52·9% (in south Asia) and 56·8% (in east Asia and the Pacific) of deaths were in males (table 2). These proportions among children aged 5–14 years varied between 52·7% in sub-Saharan Africa and 62·4% in east Asia and the Pacific (table 2). For young people aged 15–24 years, this proportion ranged from 56·9% in sub-Saharan Africa to 77·4% in Latin America and the Caribbean (table 2).

**Table 2:** Estimates and 90% UIs for the sex-specific number of deaths (in thousands) in 1990 and 2021

	**0–4 years**		**5–14 years**		**15–24 years**	
	Female	Male	Female	Male	Female	Male
**1990**						
World	6040 (5980–6190); 47·1%	6790 (6760–7000); 52·9%	771 (756–802); 47·2%	861 (842–898); 52·8%	727 (703–797); 42·7%	975 (943–1060); 57·3%
South Asia	2400 (2340–2480); 49·2%	2480 (2440–2580); 50·8%	290 (278–304); 49·9%	291 (279–306); 50·1%	290 (269–314); 53·3%	254 (236–276); 46·7%
Europe and central Asia	173 (170–181); 43·9%	221 (217–230); 56·1%	21 (20–22); 38·9%	33 (32–34); 61·1%	33 (32–37); 26·6%	91 (88–99); 73·4%
Middle East and north Africa	265 (260–276); 47·3%	295 (290–308); 52·7%	34 (32–37); 45·3%	41 (39–44); 54·7%	25 (22–30); 34·2%	48 (43–60); 65·8%
Sub-Saharan Africa	1830 (1800–1880); 46·6%	2100 (2080–2180); 53·4%	267 (256–288); 48·3%	286 (275–307); 51·7%	201 (190–262); 48·8%	211 (198–258); 51·2%
Latin America and the Caribbean	291 (282–302); 44·8%	359 (350–374); 55·2%	25 (24–26); 42·4%	34 (33–35); 57·6%	38 (37–40); 29·9%	89 (86–92); 70·1%
East Asia and the Pacific	1070 (1020–1140); 45·0%	1310 (1260–1410); 55·0%	131 (121–145); 43·5%	170 (156–191); 56·5%	130 (114–156); 34·0%	252 (222–295); 66·0%
North America	21 (20–21); 42·9%	28 (26–27); 57·1%	4 (4–4); 40·0%	6 (6–6); 60·0%	10 (9–10); 25·0%	30 (30–31); 75·0%
**2021**						
World	2300 (2180–2580); 45·6%	2740 (2610–3070); 54·4%	370 (359–409); 44·6%	459 (446–505); 55·4%	472 (453–543); 36·2%	831 (799–939); 63·8%
South Asia	607 (554–671); 47·1%	681 (621–752); 52·9%	69 (61–84); 42·1%	95 (85–113); 57·9%	129 (112–158); 39·1%	201 (172–251); 60·9%
Europe and central Asia	34 (32–37); 43·6%	44 (42–49); 56·4%	7 (6–7); 41·2%	10 (9–10); 58·8%	14 (14–15); 29·8%	33 (32–34); 70·2%
Middle East and north Africa	96 (79–134); 44·9%	118 (96–164); 55·1%	14 (13–18); 38·9%	22 (20–26); 61·1%	18 (16–21); 26·1%	51 (46–59); 73·9%
Sub-Saharan Africa	1320 (1200–1580); 45·5%	1580 (1450–1880); 54·5%	233 (222–265); 47·3%	260 (247–296); 52·7%	224 (206–277); 43·1%	296 (275–358); 56·9%
Latin America and the Caribbean	68 (64–75); 43·9%	87 (82–96); 56·1%	12 (11–13); 42·9%	16 (16–18); 57·1%	28 (26–31); 22·6%	96 (92–103); 77·4%
East Asia and the Pacific	160 (146–182); 43·2%	210 (191–239); 56·8%	32 (27–40); 37·6%	53 (45–67); 62·4%	49 (39–72); 28·2%	125 (98–181); 71·8%
North America	11 (11–12); 44·0%	14 (13–15); 56·0%	3 (2–3); 42·9%	4 (3–4); 57·1%	11 (10–12); 27·5%	29 (27–32); 72·5%

Regional deaths might not sum up to the global deaths because of rounding. The proportion of sex-specific deaths among total deaths in an age group for the world and each region is given after the 90% UI. The proportions may not sum up to 1 due to rounding. UI=uncertainty interval.

**Figure 1:**
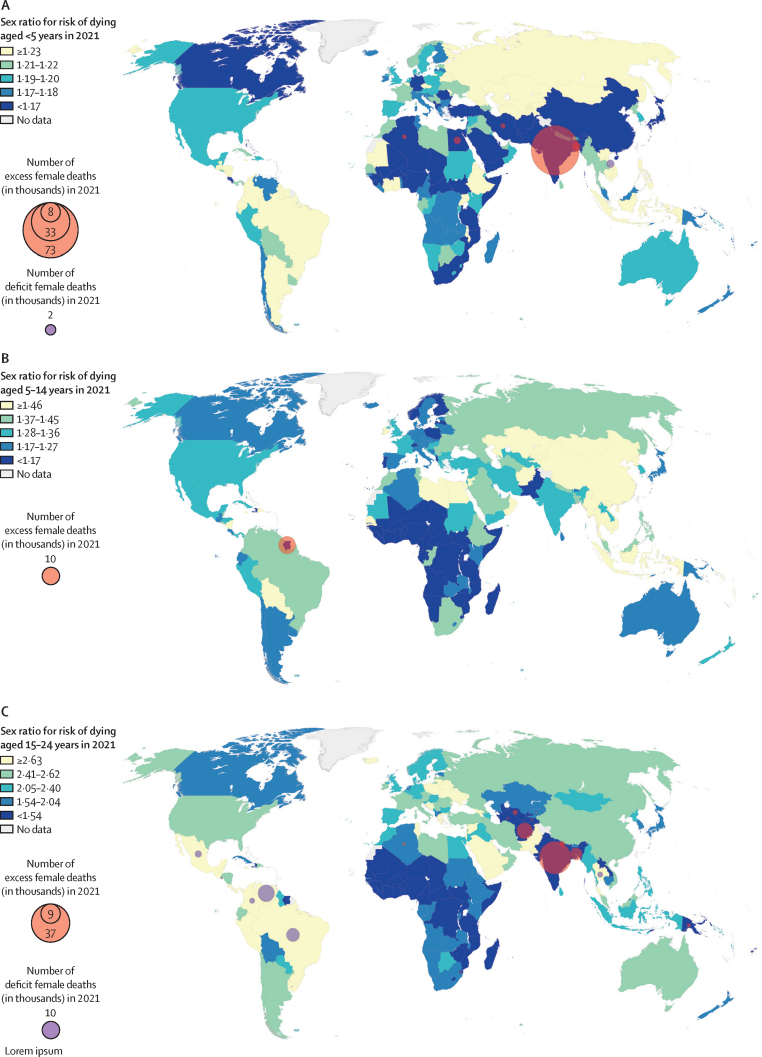
Sex ratios for the mortality rate and number of excess female deaths for age groups 0–4, 5–14, and 15–24 years in 2021 Plot shows median estimates. Numbers of excess and deficit female deaths are only shown for countries with outlying sex ratios in 2021, compared with countries of similar mortality. The colour codes are quintiles (with 20th, 40th, 60th, and 80th percentiles as cutoffs) of the sex ratio median estimates across all countries in 2021 for each age group.

Figure 1 displays the country-specific sex ratios of the probability of a newborn child dying before reaching age 5 years, the probability of a child aged 5 years dying before reaching 15 years, and the probability of an adolescent aged 15 dying before reaching 25 in 2021. We found a strong correlation between the sex ratio of mortality in 1990 between age 0–4 years and 5–14 years (0·64; p<0·0001) and between age 5–14 years and 15–24 years (0·58; p<0·0001; figure 1). However, these correlations were much lower in 2021: 0·20 (p=0·0052) between 0–4 years and 5–14 years and 0·43 between 5–14 years and 15–24 years (p<0·0001), reflecting distinct developments by age group over the past 30 years (figure 1).

**Figure 2:**
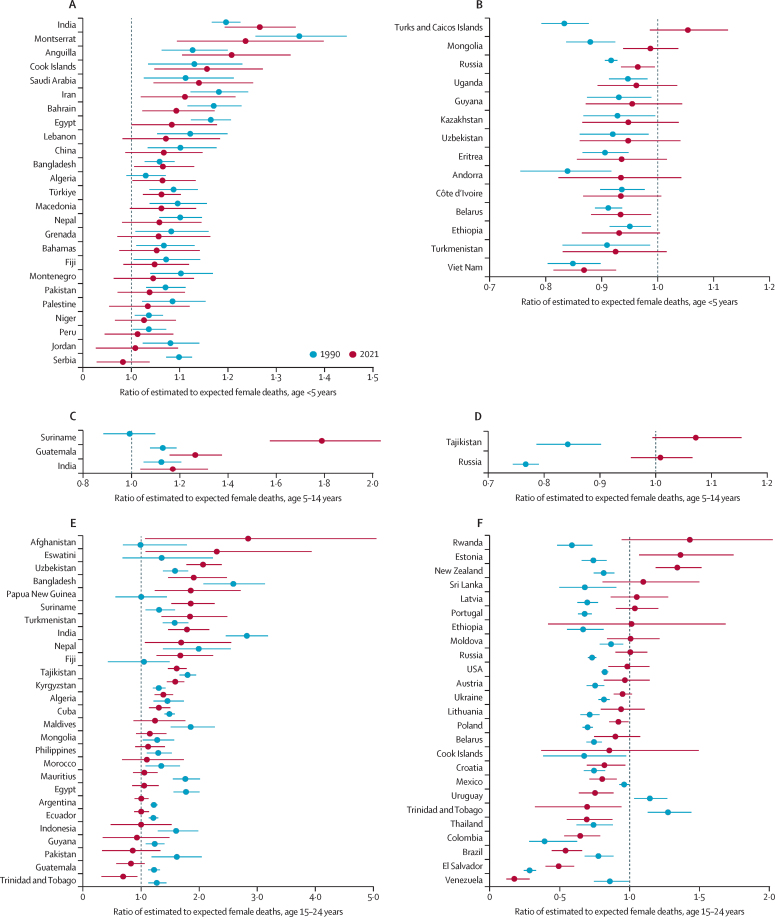
Overview of the ratio of estimated to expected female mortality, for countries with outlying sex ratios and higher-than-expected female mortality (left) and lower-than-expected female mortality (right) in 1990 and 2021 Ratio for risk of dying aged younger than 5 years in 2021 (A, B), aged 5–14 years in 2021 (C, D), and aged 15–24 years (E, F). Countries are ordered by decreasing point estimates for the year 2021. Error bars are 90% uncertainty intervals.

The observed sex ratio should be compared with an expected level to account for the increased likelihood that boys will die from conditions such as being born prematurely. Table 3 summarises excess female mortality with the corresponding number of excess deaths. In five countries (Algeria, Bangladesh, Egypt, India, and Iran), girls aged 0–4 years faced significantly higher risks of dying than boys in 2021 (table 3), compared to expected levels. In children and young adolescents aged 5–14, Suriname was the only country identified with an outlying sex ratio in 2021 (table 3). Still, the absolute number of annual excess deaths in females aged 5–14 years was low (table 3). Among young people aged 15–24 years, 21 countries had an outlying sex ratio in 2021, where 13 countries had higher-than-expected female mortality, and eight countries had lower-than-expected female mortality, resulting in negative excess female mortality and deaths (figure 2). Afghanistan had the largest excess female probability of being alive at age 15 years and dying before age 25 years (20·8 [90% UI 3·3–38·5] deaths per 1000 population; table 3). After considering the population sizes, the highest number of excess female deaths was estimated in India at 37 200 (23 600–52 300) in those aged 15–24 years in 2021 (table 3).

**Table 3:** Estimates and 90% UIs for sex ratios, ratios of estimated to expected female mortality, excess female mortality, and associated number of excess deaths (as a percentage of the total number of deaths within each age group) for countries with outlying sex ratios in 2021

	**Sex ratio**	**Estimated to expected female mortality rate**	**Excess female mortality rate (per 1000)**	**Excess female deaths**	**Deaths, %**
**Probability of dying aged 0–4 years**					
Algeria	1·16 (1·09 to 1·24)	1·06 (1·00 to 1·13)	1·2 (0·0 to 2·5)	596 (24 to 1220)	2·8%
Bangladesh	1·16 (1·10 to 1·23)	1·06 (1·01 to 1·13)	1·5 (0·1 to 3·0)	2270 (190 to 4480)	2·8%
Egypt	1·14 (1·05 to 1·24)	1·08 (1·00 to 1·18)	1·3 (0·0 to 3·2)	1610 (0 to 3920)	3·4%
India	0·98 (0·92 to 1·04)	1·26 (1·19 to 1·34)	6·5 (4·7 to 8·2)	73 200 (53 700 to 93 700)	10·3%
Iran	1·10 (1·01 to 1·20)	1·11 (1·02 to 1·21)	1·2 (0·2 to 2·9)	725 (128 to 1830)	4·6%
Vietnam	1·42 (1·33 to 1·51)	0·87 (0·81 to 0·93)	−2·6 (−3·8 to −1·4)	−1830 (−2700 to −990)	−6·0%
**Probability of dying aged 5–14 years**					
Suriname	0·84 (0·74 to 0·94)	1·79 (1·57 to 2·03)	1·9 (1·3 to 2·5)	10 (7 to 14)	23·9%
**Probability of dying aged 15–24 years**					
Afghanistan	0·62 (0·44 to 0·88)	2·85 (1·07 to 5·06)	20·8 (3·3 to 38·5)	9050 (1730 to 16 700)	34·1%
Algeria	1·80 (1·60 to 2·02)	1·38 (1·23 to 1·55)	1·1 (0·7 to 1·5)	312 (197 to 433)	9·6%
Bangladesh	1·34 (1·03 to 1·75)	1·91 (1·47 to 2·48)	3·2 (1·8 to 4·9)	5290 (2960 to 7980)	19·9%
Brazil	4·33 (3·58 to 5·20)	0·54 (0·44 to 0·66)	−3·8 (−5·0 to −2·7)	−6290 (−8230 to −4370)	−15·6%
Colombia	3·80 (3·13 to 4·57)	0·64 (0·53 to 0·78)	−2·5 (−3·5 to −1·4)	−1050 (−1500 to −600)	−11·5%
El Salvador	4·90 (4·00 to 5·99)	0·49 (0·40 to 0·60)	−3·6 (−4·7 to −2·6)	−220 (−290 to −160)	−16·7%
Eswatini	0·90 (0·58 to 1·43)	2·30 (1·08 to 3·94)	13·5 (1·5 to 23·1)	153 (18 to 263)	27·9%
Fiji	1·50 (1·12 to 1·96)	1·68 (1·27 to 2·24)	3·2 (1·4 to 5·3)	24 (11 to 41)	15·9%
India	1·41 (1·16 to 1·71)	1·79 (1·47 to 2·17)	3·1 (1·9 to 4·3)	37 200 (23 600 to 52 300)	17·2%
Kyrgyzstan	1·59 (1·46 to 1·74)	1·59 (1·45 to 1·75)	2·1 (1·7 to 2·6)	110 (90 to 130)	14·2%
Mexico	2·98 (2·67 to 3·32)	0·80 (0·71 to 0·91)	−1·5 (−2·3 to −0·7)	−1610 (−2520 to −730)	−6·2%
Nepal	1·43 (0·96 to 2·10)	1·69 (1·07 to 2·55)	3·2 (0·4 to 7·4)	1020 (120 to 2430)	16·3%
Papua New Guinea	1·29 (0·90 to 1·85)	1·86 (1·24 to 2·72)	5·8 (1·9 to 10·2)	539 (184 to 951)	19·0%
Suriname	1·33 (1·10 to 1·60)	1·86 (1·52 to 2·27)	4·5 (2·9 to 6·4)	24 (16 to 34)	19·6%
Tajikistan	1·50 (1·36 to 1·64)	1·61 (1·46 to 1·79)	1·3 (1·0 to 1·6)	111 (87 to 136)	14·8%
Thailand	3·30 (2·63 to 4·07)	0·69 (0·55 to 0·87)	−2·5 (−4·1 to −0·9)	−1070 (−1750 to −380)	−9·7%
Trinidad and Tobago	3·20 (2·46 to 4·09)	0·69 (0·32 to 0·94)	−2·7 (−17·8 to −0·5)	−26 (−172 to −4)	−9·6%
Turkmenistan	1·35 (1·01 to 1·79)	1·84 (1·35 to 2·49)	3·8 (1·7 to 6·8)	188 (85 to 336)	18·6%
Uruguay	3·34 (2·84 to 3·91)	0·75 (0·63 to 0·88)	−1·4 (−2·2 to −0·6)	−34 (−54 to −15)	−7·5%
Uzbekistan	1·21 (1·04 to 1·40)	2·07 (1·78 to 2·39)	3·4 (2·6 to 4·2)	874 (678 to 1070)	22·9%
Venezuela	5·00 (3·68 to 6·68)	0·17 (0·11 to 0·28)	−44·5 (−71·2 to −20·0)	−10 000 (−20 000 to 0)	−77·5%

Countries are ordered alphabetically. UI=uncertainty interval.

## Discussion

This study is the first to provide estimates of sex ratios with UIs from birth to age 24 years and to quantify country-specific deviations from the expected sex ratio for the same level of mortality. We used the empirical measurements available in the public domain and provided age-specific and sex-disaggregated mortality rates. These findings can be used to inform the construction of national life tables, and track progress with a sex-based perspective in the context of the Sustainable Development Goals and the UN Global Strategy for Women's, Children's, and Adolescents’ Health.^21^

We showed that the sex ratio rapidly increases as children age, with a particularly notable increase between the 5–14 years age group and the 15–24 years age group. This finding has important implications because mortality has declined more rapidly in neonates and young children than in adolescents and young people since 1990.^10^ As the burden of mortality gradually shifts to older ages, the proportion of male deaths will increase. Already 56·2% of all deaths below age 25 years were in males in 2021 globally, and this proportion reached 64·8% in Latin America and the Caribbean and 65·3% in North America.

Regional estimates present contrasting patterns, reflecting the state of the health transition in different regions (and thus the levels of mortality for both sexes and the associated cause-of-death distribution), the extent of discrimination against girls, and the disproportionate exposure of males to specific causes of death (eg, accidents and violence). In particular, in 1990, south Asia had atypically low sex ratios at all ages, falling below unity, reflecting the extent of discrimination against girls in that region. The region has since experienced rapid growth in the sex ratio to the point at which it now has a higher sex ratio of mortality than sub-Saharan Africa in the age groups 5–14 years and 15–24 years. Despite this progress, in four countries in south Asia (Afghanistan, Bangladesh, India, and Nepal), we estimated that 134 138 excess female deaths occurred in 2021 below the age of 25 years, potentially due to discriminatory practices. Discrimination against girls is a complex issue with deep-rooted historical factors, including patriarchal norms placing girls in subordinate positions and limiting their access to education and health, patrilineal kinship systems implying that productive assets are passed down through the male line whereas girls and women have costs associated with dowry, and preferences for male children due to a perceived higher potential for earnings and source of support at older ages.^22^

Discrimination against girls should not obfuscate that as children reach adolescence and adulthood, some countries experience widening gaps to the disadvantage of boys. Latin America and the Caribbean, in particular, stand out as a region with high sex ratios, particularly above age 15 years, deviating from the experience of other regions, because of the increased contribution of violent deaths at these ages. In their study on cause-specific mortality fractions in 5–19-year-olds, Liu and colleagues^12^ estimated that injuries caused 54·3% of deaths in this age range in Latin America and the Caribbean in 2019, compared with 32·9% globally. Interpersonal violence accounted for 40·9% of deaths in males aged 15–19 years in this region, compared with 12·3% globally.

Since 1990, sex ratios have increased in all age groups because of faster progress against female mortality. Such an increase has been found worldwide and in most regions, although the sex ratio tends to stabilise or decline in countries with low mortality levels, as observed in North America. The mechanism to explain the stabilisation and subsequent decline in the sex ratio when mortality reaches lower levels requires further research. In other countries, trends since 1990 reflect, partly, the varying paces of the mortality decline and, partly, sociocultural changes related to, for example, less discrimination against girls than against boys or greater exposure of boys than girls to specific causes of violence. Cause-specific estimates for 5–19-year-olds have revealed that mortality rates due to road traffic injuries have declined moderately compared with other causes, such as measles, whereas mortality due to collective violence has increased in 2010–19.^12^

**Figure 3:**
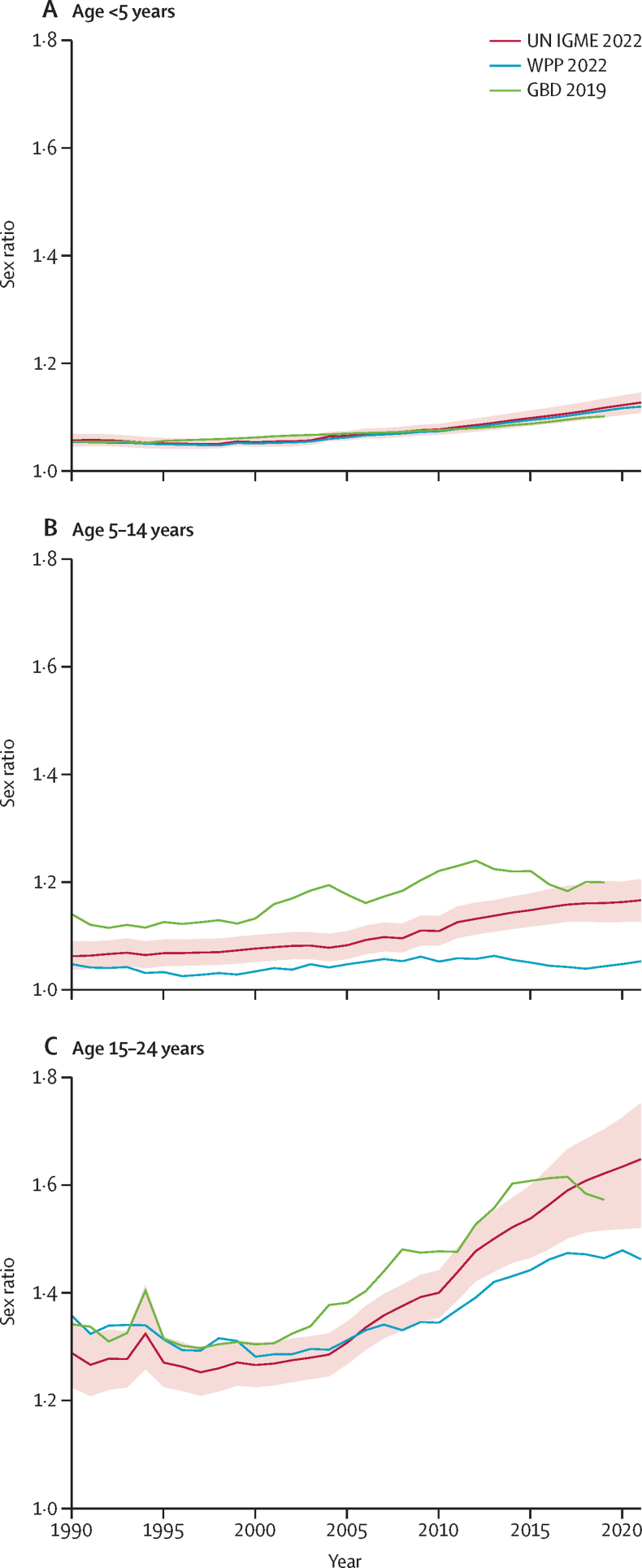
Global trends in the sex ratio of dying younger than 5 years, aged 5–14 years, and aged 15–24 years, 1990–2021 Figure shows 90% uncertainty intervals from the UN IGME 2022 results. GBD=Global Burden of Diseases, Injuries, and Risk Factors Study. IGME=Inter-agency Group for Child Mortality Estimation. WPP=World Population Prospects results.

Our estimates of the sex ratios in the first 25 years of life contrast with previous estimates. The estimates for children younger than 5 years are close to those in GBD 2019 or the 2022 World Population Prospects (WPP; figure 3).^13^, ^23^ Still, essential discrepancies exist when assessing children and young adolescents aged 5–14 years or youth aged 15–24 years. The GBD estimates predict higher mortality for boys aged 5–24 years than in this study, except in 2018 and 2019 for young people aged 15–24 years. By contrast, WPP predicts lower sex ratios of mortality among children aged 5–14 years years since 1990 and in young people aged 15–24 years over the past 16 years. Differences between this study and results from GBD 2019 and WPP 2022 are magnified when considering the national level. Between ages 5 and 14 years, the sex ratio estimated in this study for 2019 differs by more than 20% from GBD estimates in 36 countries (62 countries compared with WPP). These discrepancies were more common between ages 15 and 24 years in 2019 (GBD produced results up to 2019): for 53 countries, the sex ratio we estimated differs by more than 20% from that of GBD, which is the case for 73 countries for WPP. These differences in sex ratio are combined with differences in mortality levels, which we have documented elsewhere.^10^, ^17^ There are several advantages in the proposed approach over the previous estimates—for example, using estimates that refer to these age groups without resorting to inferences based on sex differentials observed at other ages, modelling sex inequalities by specific 1-year or 5-year age groups, accounting for sampling and reporting errors in surveys and census data, and measuring the sex-specific completeness of death registration. We found strong and positive correlations of sex ratios between neighbouring age groups in 1990, but these correlations were much reduced in 2021. The reduced correlation over time in sex ratios between neighbouring age groups suggests that estimating sex ratios in older age groups from those observed in young children might be misleading.

However, the proposed approach also has limitations. First, although the UN IGME strive to compile all relevant data for child mortality estimation, the data sources are constrained to be nationally representative. Other sources such as the Health and Demographic Surveillance System or hospital or facility data might be relevant to child mortality estimation but are not included here as data sources. Second, these estimates are based on the global pattern for countries without age-disaggregated and sex-disaggregated mortality data. Third, relying on the relationship between the sex ratio and total mortality (female and male combined) level, we could introduce errors in the sex ratio if the total mortality itself is biased. Fourth, we do not use covariates that could improve the estimates (appendix p 14). Fifth, the proposed model can produce plausible estimates and short-term projections in periods of about 20 years (as verified by the validation results); however, the model requires further updates to produce reliable long-term projections.

Further studies should identify the contribution of varying cause-of-death distributions and social factors, such as discriminatory practices or violence, in producing outlying sex ratios. The contrasting regional patterns and trends in sex ratios also show the importance of age-disaggregated and sex-disaggregated data for monitoring progress against premature mortality. Finally, as outlined by the Global Accelerated Action for the Health of Adolescents framework,^24^ our results also show the urgency for sex-specific and age-adjusted interventions to address certain subpopulations’ needs.

## Data sharing

Empirical databases and final estimates are updated annually and are available in the public domain (http://www.childmortality.org) and the appendix. R code scripts are available from the corresponding author upon request.

## Declaration of interests

We declare no competing interests.
