# Supplementary material for: Sex differences in mortality among children, adolescents, and young people aged 0–24 years: a systematic assessment of national, regional, and global trends from 1990 to 2021
Source: Lancet Glob Health. 2023 Sep 19;11(10):e1519–30. doi: 10.1016/S2214-109X(23)00376-5 (PMC10522776; doi:10.1016/S2214-109X(23)00376-5)
Supplement: Supplementary appendix [file mmc1.pdf]

THE LANCET
Global Health

Supplementary appendix

This appendix formed part of the original submission and has been peer reviewed. We post it as supplied by the authors.

Supplement to: Chao F, Masquelier B, You D, et al. Sex differences in mortality among children, adolescents, and young people aged 0–24 years: a systematic assessment of national, regional, and global trends from 1990 to 2021. *Lancet Glob Health* 2023; **11:** e1519–30.

Web Appendix for
Sex differences in mortality among children, adolescents, and youth aged 0–24: A systematic assessment of national, regional, and global trends from 1990 to 2021

Fengqing Chao[*1], Bruno Masquelier[2], Danzhen You[3], Lucia Hug[3], Yang Liu[3], David Sharrow[3], Håvard Rue[1], Hernando Ombao[1], and Leontine Alkema[4]

[1]Statistics Program, Computer, Electrical and Mathematical Sciences and Engineering Division, 4700 King Abdullah University of Science and Technology (KAUST), Thuwal 23955-6900, Saudi Arabia

[2]University of Louvain, Louvain-la-Neuve, Belgium

[3]Division of Data, Analytics, Planning and Monitoring, United Nations Children's Fund, New York, NY, USA

[4]Department of Biostatistics and Epidemiology, School of Public Health and Health Sciences, University of Massachusetts, Amherst, MA, USA

on behalf of the UN Inter-agency Group for Child Mortality Estimation

August 1, 2023

[*]Corresponding author (FC); Email: fengqing.chao@kaust.edu.sa

Contents

1 Data **1**

2 Data pre-processing **1**

2.1 Extraction by data source type . . . 2
2.2 Adjustments for the incompleteness of death registration . . . 2
2.3 Exclusion criteria . . . 3

3 Methods for sex ratio for five-year age groups 5–24 **4**

3.1 Data model . . . 4
3.2 Process model . . . 6
3.3 Model summary . . . 7
3.4 Derivation of sex-specific mortality . . . 7
3.5 Derivation of sex ratio for 10-year age groups . . . 10
3.6 Computation of final results . . . 11
3.6.1 Constructing posterior samples of outcomes that include crisis-related deaths and uncertainty in national-level mortality rate . . . 11
3.6.2 Rounding . . . 12
3.6.3 Constructing uncertainty intervals and point estimates . . . 12
3.6.4 Imputing results for countries without data . . . 13

4 Validation exercise and results **13**

5 Global expected sex ratios by age group **14**

6 Discussion on adding covariates in Bayesian models **14**

7 GATHER Compliance **16**

8 Supplementary tables **18**

List of Tables

2 Distribution of observations by data source type and age for children aged 0–4 . . . 1
3 Distribution of observations by data source type and age group for children, adolescents, and youth aged 5–24 . . . 2
4 Excluded observation distribution by data exclusion criteria and age group . . . 4
5 Notation summary . . . 9
6 Derivation of sex-specific mortality rate for a 5-year age group . . . 10
7 Validation results for left-out observations by age group. . . . 13
8 Summary of differences in sex ratio estimates in observation years 2000, 2005, and 2010 based on training dataset and full dataset. . . . 14
9 Checklist of information that should be included in new reports of global health estimates . . . 16
10 Classification of countries by UNICEF regions . . . 18
11 Completeness of death registration for intercensal periods . . . 19
12 Country-years with crisis adjustments, by age group . . . 22
13 Estimates and 90% uncertainty intervals for sex ratios related results for IMR in 1990 and 2021, for the world, UNICEF regions, and all countries . . . 24
14 Estimates and 90% uncertainty intervals for sex ratios related results for CMR in 1990 and 2021, for the world, UNICEF regions, and all countries . . . 29

15 Estimates and 90% uncertainty intervals for sex ratios related results for U5MR in 1990 and 2021, for the world, UNICEF regions, and all countries . . . 34
16 Estimates and 90% uncertainty intervals for sex ratios related results for 5q5 in 1990 and 2021, for the world, UNICEF regions, and all countries . . . 39
17 Estimates and 90% uncertainty intervals for sex ratios related results for 5q10 in 1990 and 2021, for the world, UNICEF regions, and all countries . . . 44
18 Estimates and 90% uncertainty intervals for sex ratios related results for 10q5 in 1990 and 2021, for the world, UNICEF regions, and all countries . . . 49
19 Estimates and 90% uncertainty intervals for sex ratios related results for 5q15 in 1990 and 2021, for the world, UNICEF regions, and all countries . . . 54
20 Estimates and 90% uncertainty intervals for sex ratios related results for 5q20 in 1990 and 2021, for the world, UNICEF regions, and all countries . . . 59
21 Estimates and 90% uncertainty intervals for sex ratios related results for 10q15 in 1990 and 2021, for the world, UNICEF regions, and all countries . . . 64
22 Sex ratio data sources for age group 0–1, by country . . . 69
23 Sex ratio data sources for age group 1–4, by country . . . 90
24 Sex ratio data sources for age group 0–4, by country . . . 111
25 Sex ratio data sources for age group 5–9, by country . . . 121
26 Sex ratio data sources for age group 10–14, by country . . . 144
27 Sex ratio data sources for age group 15–19, by country . . . 167
28 Sex ratio data sources for age group 20–24, by country . . . 184

List of Figures

1 Data and model process overview . . . 5
2 Global relation between total mortality and sex ratio . . . 8
3 Overview of the global relations between sex ratios and total mortality levels . . . 15

List of Abbreviations

ACLED	Armed Conflict Location and Event Data Project
CMR	Child Mortality Rate
CRED	Centre for Research on the Epidemiology of Disasters
DDM	Death Distribution Method
DHS	Demographic and Health Survey
EM-DAT	Emergency Events Database
GATHER	Guidelines for Accurate and Transparent Health Estimates Reporting
GGB	Generalized Growth Balance method
GHE	Global Health Estimates
HDSS	Health and Demographic Surveillance Systems
HMD	Human Mortality Database
IGME	UN Inter-agency Group for Child Mortality Estimation
IMR	Infant Mortality Rate
INLA	Integrated Nested Laplace Approximation
MICS	Multiple Indicator Cluster Surveys
PC	Penalized Complexity
RW1	first-order Random Walk
RW2	second-order Random Walk
SEG	Synthetic Extinct Generation method

SRS	Sample Registration System
U5MR	Under-5 Mortality Rate
UCDP	Uppsala Conflict Data Program
UN IGME	UN Inter-agency Group for Child Mortality Estimation
VR	Vital Registration
WHS	World Health Surveys
WPP	World Population Prospects

1 Data

Table 2 and 3 give an overview of the observations by data source type and age group. The detailed information on country-specific data series names and the assigned data source types for each age group are in the Supplementary Tables 22 (0–1), 23 (1–4), 24 (0–4), 25 (5–9), 26 (10–14), 27 (15–19), 28 (20–24). Data source types group data series with similar characteristics, such as data collection method, survey sampling design, and data reporting method.

We categorized all data sources into eight types for data under age five. For age groups under five. Specifically, the groupings of data series into different data source types are:

- Census Indirect: census based on summary birth histories;
- Demographic and Health Surveys (DHS) Direct: standard DHS with full birth histories;
- Multiple Indicator Cluster Surveys (MICS) Direct: MICS based on full birth histories;
- MICS Indirect: MICS based on summary birth histories;
- Other DHS Direct: Interim DHS, Special DHS, DHS AIDS Indicator Survey, DHS Malaria Indicators Survey, National DHS, World Fertility Survey, and surveys with similar sampling design and based on full birth histories;
- Others Direct: census direct, Reproductive Health Survey, Pan-Arab Programme on Family Health, National MICS, panel data, and national reports, based on full birth histories;
- Others Indirect: all types of DHS surveys, Reproductive Health Surveys, and national reports, based on summary birth histories

For data above age five, we classified all data sources into three types:

- Demographic and Health Surveys (DHS) Direct: including standard DHS;
- Others Direct: including Multiple Indicator Cluster Surveys (MICS), World Health Surveys (WHS), and Panel data;
- Vital Registration (VR) or Sample Registration System (SRS).

	Age group		
**Data source**	**0–1**	**1–4**	**0–4**
Census Indirect	5	0	5
DHS Direct	1479	1370	0
MICS Direct	276	244	0
MICS Indirect	0	0	271
Other DHS Direct	355	335	0
Others Direct	308	290	0
Others Indirect	0	0	262
VR/SRS	4307	4094	32
**Total**	**6,730**	**6,333**	**778**
**Countries with data**	196	195	94
**Reference year range**	1950–2021	1950–2021	1950–2017

Table 2: **Distribution of observations by data source type and age for children aged 0–4.** Direct: data obtained from full birth histories. Indirect: data obtained from summary birth histories.

2 Data pre-processing

The database we use for model fitting (summarized in Section 1) is based on multiple data quality assessments and pre-processing steps.

	Age group			
**Data source**	**5–9**	**10–14**	**15–19**	**20–24**
DHS Direct	1,038	1,015	834	834
Others Direct	568	550	266	267
VR/SRS	3,702	3,647	3,745	3,723
**Total**	**5,308**	**5,212**	**4,845**	**4,824**
**Countries with data**	190	190	178	179
**Reference year range**	1954–2021	1954–2021	1958–2021	1958–2021

Table 3: **Distribution of observations by data source type and age group for children, adolescents, and youth aged 5–24.** Direct: data obtained from full sibling histories. Others Direct: data from recent household deaths reported in censuses and large-scale surveys.

2.1 Extraction by data source type

Vital registration data Nationally representative vital registration data were extracted from the WHO mortality database and the Human Mortality Database (1; 2). Completeness of death registration data for children below age five was estimated as the reported under-five mortality rate (U5MR) in vital registration data divided by the corresponding UN IGME estimate, which may rely on other data sources, such as surveys and census data. We estimated the completeness of death registration for the age groups 5–14 and 15–24 using death distribution methods, comparing the age distribution of deaths between two censuses with the age distribution of the population enumerated (3). The stochastic standard error of the observation was calculated using the Poisson approximation based on the number of births or the population turning 5 or 15 in a given year, estimated from the 2022 World Population Prospects (WPP) (4). We also included estimates from sample vital registration systems in India (5) and Bangladesh (6), the Chinese national mortality surveillance system (7), and the Rapid Mortality Surveillance in South Africa (8).

Birth and sibling histories in surveys Data from full birth histories collected in DHS, MICS, World Health Surveys, World Fertility Surveys, Reproductive Health Surveys, and selected surveys from the Pan-Arab Programme on Family Health were used to compute sex ratios for children and adolescents below age 15, for periods of varying lengths, optimised to capture shorter-term changes for country-years with sufficient information (9). Data from full sibling survival histories from DHS and MICS were used to estimate the sex ratio in youth aged 15–24. In all age groups, the sampling variance was calculated using the Jackknife estimation to account for the survey sample design. Indirect methods based on the number of children ever born and surviving, called summary birth histories, were used for children below age five.

Censuses Reports on recent household deaths were used to compute sex-specific estimates. The calculation of age-specific mortality rates was derived from standard life table methods. Data from the summary birth histories in censuses were also used for children under five.

2.2 Adjustments for the incompleteness of death registration

Vital registration data were extracted from the WHO mortality database[1]. When available, the latest estimates from the Human Mortality Database (HMD)[2] were also added. We assumed that death registration was complete in 38 countries included in the HMD. For the other countries, we evaluated the completeness based on "death distribution methods" (DDMs). The VR data were only adjusted for completeness of death reporting for mortality data above age 5. Loess regression (for smaller gaps in years) and linear interpolation (for gaps greater than five years) were applied to generate the time series using the available estimates of child completeness to impute completeness in years when vital registration data were unavailable. Table 11 presents the completeness estimates by country-year.

[1] https://www.who.int/healthinfo/mortality_data/en/
[2] https://www.mortality.org/

DDMs compare the age distribution of deaths in the VR with the age distribution of the population enumerated in censuses to estimate the fraction of deaths that are reported. Three methods are applicable when two censuses are available and deaths have been recorded in the intercensal period: the generalized growth balance method (GGB) (10; 11), the synthetic extinct generation method (SEG) (12), and a combination of the two, called the hybrid method (or GGB>SEG). All methods are well described in the book *Tools for Demographic Estimation* (13), with Excel spreadsheets to facilitate their use. We employed here the GGB>SEG approach, which has been shown to perform better than the GGB and SEG methods used separately (14). Completeness was estimated for each country for periods between pairs of recent censuses for which an age distribution of the population was available in the Demographic Yearbook. After the last census, it is impossible to estimate completeness with death distribution methods, so completeness levels were kept constant until 2021.

Using the DDM package in R (15), we separately estimated completeness for males and females. Vital statistics were excluded when the estimated completeness was less than 80% (for at least one sex), adjusted when completeness was between 80% and 95%, and included without adjustment when completeness was greater than or equal to 95%. The VR data were adjusted as follows:

$$\text{adjusted VR data} = \frac{\text{original VR data}}{\text{completeness}}$$

Completeness was assumed to be invariant by age in the age group 5-24. In a few countries, vital registration data were incorporated to estimate mortality above age 5, despite being deemed too incomplete to be used for under-five mortality. The completeness of death registration could be lower among children due to common under-reporting of neonatal deaths (16). In some countries (e.g. Brazil, Republic of Korea, Syria, Turkey, Singapore, Bahrain, and Sri Lanka), we have preferred not to adjust upwards the deaths reported to vital statistics, either because of large migrations flows that could invalidate the method, or because published studies had investigated the completeness of death registration (see (17) for further details).

Uncertainty was added in countries with adjusted VR data to account for the uncertainty associated with the completeness estimate. Murray et al. (2010) demonstrate that uncertainty intervals around completeness estimates from DDM are large, roughly about 25% (18). This is partly because death distribution methods are based on assumptions often poorly respected in practice (e.g. constant under-registration by age). We proceeded as follows:

1. The sex-specific completeness was simulated with a uniform distribution: Uniform(completeness - 25%, completeness + 25%).
2. The number of deaths with additional uncertainty was simulated by: Poisson($\frac{\text{reported deaths}}{\text{simulated completeness}}$)
3. The sex-specific mortality rates with additional uncertainty were simulated using a standard life table approach with the simulated number of deaths from the previous step.

2.3 Exclusion criteria

Before fitting the observations to the Bayesian model, we excluded data points based on the following rules:

- Exclude observations with sex ratio outside [0.1, 10] for ages under five, and outside [0.1, 10] for ages above five (refer to Table 4 for the number of observations that fall outside the intervals by age group).
- Exclude observations during national crisis periods.
- Exclude observations from surveys or censuses with strong evidence of poor data quality.

Table 4 summarizes the excluded data points by each exclusion criterion.

Data series were excluded from the sex-specific analysis when the review conducted to develop estimates for both sexes had concluded that there was substantial evidence of recall errors. The procedures to identify recall errors in surveys and censuses are discussed in (19) for the age group 0-4, (20) for the age group 5-14 and (17) for the age group 15-24. We have not used a grading method to assess the quality of survey or census data, but a series of tests are put in place to identify sources of erroneous data. For example, in censuses, we assess parity ratios from one survey to the next to detect omissions of children in summary birth histories (21). In full birth histories from surveys, we use

Exclusion criteria	Age group 0–1	1–4	0–4	5–9	10–14	15–19	20–24
Sex ratio outside [0.2, 5]	530	2,033	562	–	–	–	–
Sex ratio outside [0.1, 10]	–	–	–	14	105	36	66
During national crisis	237	235	240	57	57	46	46
**Total**	**767**	**2,263**	**801**	**71**	**162**	**82**	**111**
**Percentage of total**	0.9%	2.3%	1.0%	1.1%	2.4%	1.4%	1.8%

Table 4: **Excluded observation distribution by data exclusion criteria and age group.** The total number of excluded observations may not be the sum of the excluded number of data under each criterion because some excluded data satisfy more than one criterion.

consistency checks such as the ratio between neonatal and post-neonatal mortality and the distribution of deaths by day (22). In sibling survival histories, we examine the plausibility of the relationship between the probabilities ${}_{10}q_{15}$ and ${}_5q_0$ against high-quality life tables from Health and Demographic Surveillance Systems (HDSS) and the Human Mortality Database (17).

3 Methods for sex ratio for five-year age groups 5–24

We provide a full model specification in this web appendix. The statistical model for estimating the sex ratio of mortality for 0–1, 1–4, and 0–4 are detailed in (23).

In the following Section 3.1 and Section 3.2, we summarize the model to estimate the sex ratio of male mortality to female mortality for the age group for age groups 5–9, 10–14, 15–19, and 20–24. Since the same model is applied to each age group, we omit the index for age group, and all the notations in Section 3.1 and Section 3.2 are age specific.

The outcome of interest is $\exp\{\Psi_{c,t}\}$, the sex ratio of mortality rate in the country c in year t, where $\Psi_{c,t}$ is the log of the sex ratio. For the country and year indexes, $c \in \{1,\cdots,k\}$ with $k = 200$ referring to the total number of countries, and $t \in \{1,\cdots,j\}$ with $j = 72$ indicating the individual years from 1950 ($t = 1$) to the year 2021 ($t = j$). Throughout the report, $\mathcal{N}(\mu,\sigma^2)$ refers to a normal distribution with mean μ and variance σ^2 and $\mathcal{U}(a,b)$ refers to a continuous uniform distribution with lower and upper bounds at a and b respectively.

The complete data process and model overview are in the flow chart Figure 1. The detailed explanations of the model and the posterior distribution are in the rest of this section.

3.1 Data model

Let z_i to be the ith observed sex ratio and $y_i = \log(z_i)$. We have the model:

$$
\begin{aligned}
y_i &= \Psi_{c[i],t[i]} + \varepsilon_i, \text{ for } i \in \{1,\cdots,n\}, & (1)\\
\varepsilon_i|\omega_{s[i]} &\sim \mathcal{N}(0, u_i^2 + \omega_{s[i]}^2), & (2)\\
\omega_s &= 0, \text{ for } s = m, & (3)\\
\tau_{\omega,s} = \omega_s^{-2} &\sim \mathscr{PC}(z, 0.01), \text{ for } s \in \{1,\cdots,m-1\}. & (4)
\end{aligned}
$$

For a certain age group, the observations of the sex ratio are indexed by $i \in \{1,\cdots,n\}$. y_i denotes the ith log of observed sex ratio of mortality rate in country $c[i]$ in year $t[i]$ and is modeled on the log-scale. $\exp\{\Psi_{c,t}\}$ is the outcome of interest, the true sex ratio of mortality rate, in country c in year t. ε_i is the error term for y_i.

The error term ε_i follows a normal distribution as shown in Equation 2. The variance is the sum of two parts: (i) known stochastic/sampling error variance u_i^2, (ii) unknown non-sampling error variance $\omega_{s[i]}^2$ that differs by data source type s. The sampling variance is pre-calculated using the Jackknife resampling method by the `R`-package **demogsurv**[3] to account for the uncertainty due to the sample design of the surveys. We assume the non-sampling error for VR/SRS

[3]Available at: `https://github.com/mrc-ide/demogsurv`. Accessed on July 27th, 2020.

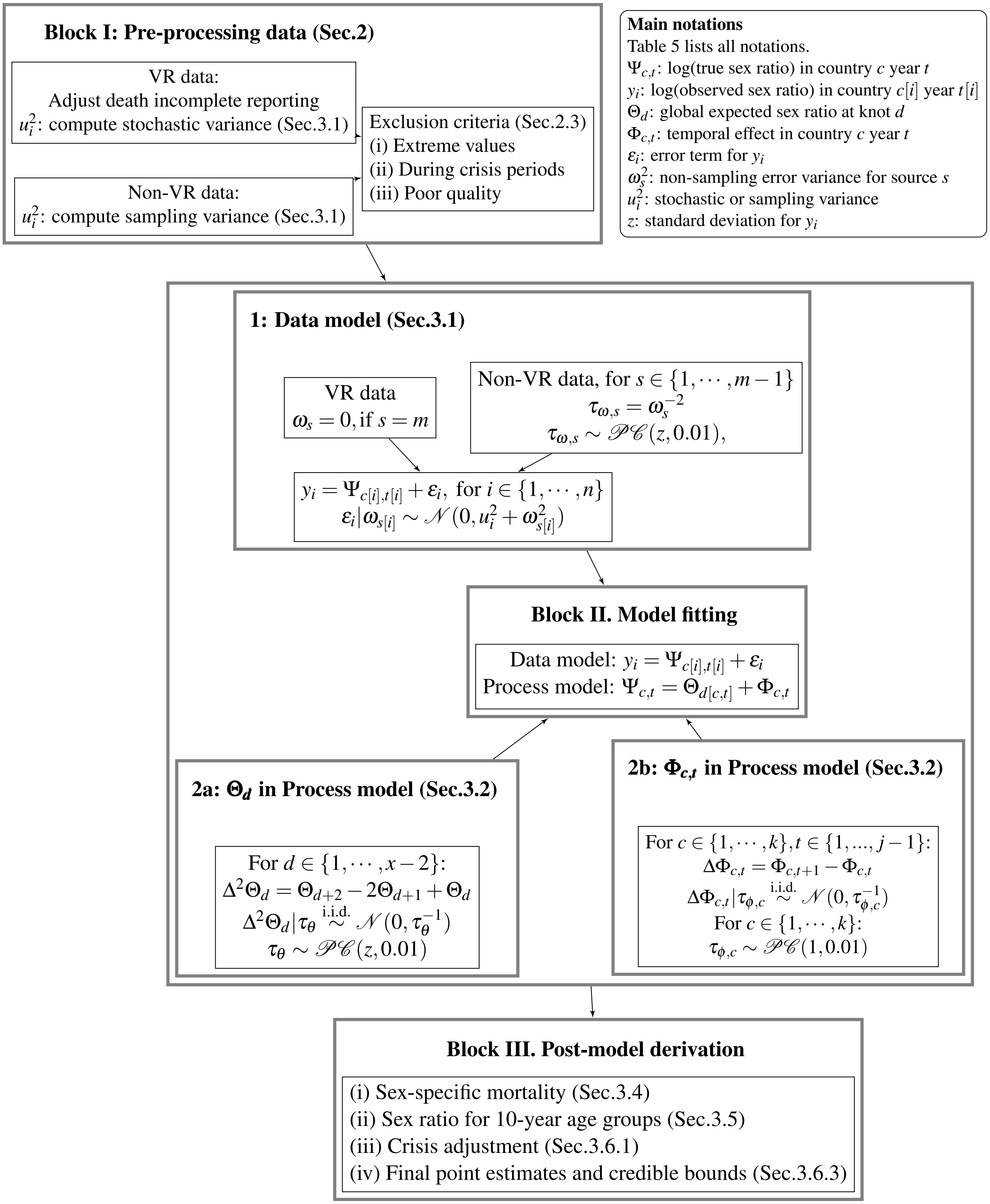

Figure 1: **Data and model process overview.** This flow chart summarizes the major steps of pre-precessing data (Block I), the Bayesian modelling steps (Block II), where $\Psi_{c,t}$ refers to the true sex ratio of mortality on the log-scale, and the post-model derivation (Block III).

data is zero (i.e. $s = m$ refers to data source type VR/SRS). For data from non-VR/SRS source types, we estimate non-sampling error variances that vary across data source types ($s \in \{1, \cdots, m-1\}$ refer to the set of non-VR/SRS data source types). z is the standard deviation of y_i. We assign Penalized Complexity (PC) priors for the precision parameter $\tau_{\omega,s}$ (24).

The Bayesian estimates are pooled towards informative observations and are less influenced by weakly informative observations. Retrospective estimates based on surveys and recent household deaths could be affected by differential underreporting of deaths in boys or girls. However, comparisons of birth histories in surveys with prospective demographic surveillance did not find that data quality markers were associated with the sex of the child (25). In sibling survival histories, there is evidence that underreporting of deaths is more frequent for brothers than sisters (26), so our estimates of the sex ratio in the age group 15–24 could be biased slightly downwards in countries where sibling data are our primary data source.

PC priors are default priors assigned to precision parameters used in the R-package R-INLA, which we used for Bayesian inference (refer to Section 3.3, paragraph Statistical computation). The main advantages of PC priors are that they are invariant to reparameterizations and have excellent robustness properties according to (24).

3.2 Process model

The process model for $\Psi_{c,t}$ is defined as:

$$\Psi_{c,t} \quad = \quad \Theta_{d[c,t]} + \Phi_{c,t}, \tag{5}$$

where $\Theta_{d[c,t]}$ is the global expected sex ratio evaluated at the total mortality rate (female and male combined) for country c in year t (more explanations in the next paragraph). $\Phi_{c,t}$ is the year-by-year fluctuation around the global relation, capturing the intra-country temporal correlation between country-specific observations.

$\Theta_{d[c,t]}$ models the global non-linear relation between $\Psi_{c,t}$ and the total mortality rate in country c, year t. Specifically, let $V_{c,t}$ denote the log of total (male and female combined) mortality rate for country c year t, taken from the UN Inter-agency Group for Child Mortality Estimation (UN IGME) 2022 estimates (27). We define a grid of values κ_d for $d \in \{1, \cdots, x\}$. x is the number of grid points where Θ_d is evaluated. $x = 310$ for age group 5–9, $x = 230$ for age group 10–14, $x = 260$ for age group 15–19, $x = 275$ for age group 20–24. x differs across age groups because the range of total mortality and country-years with data in different age groups varies. For all the five-year age groups from 5 to 24, we set $\kappa_1 = \log(1/1000)$, corresponding to 1 death per 1000 population on the log scale. We set κ_x as the 99th percentile of $V_{c,t}$ across all country-years with data in each age group. We match each $V_{c,t}$ to the κ_d with the smallest absolute difference from $V_{c,t}$, denoting the dth index for country-year c,t as $d[c,t]$. To model the relation between Θ_d and κ_d, we use a second-order random walk (RW2) process. We assume Θ_d is constant outside the range of κ_1 and κ_x across all c and t. In particular,

$$\Delta^2\Theta_d \quad = \quad \Theta_{d+2} - 2\Theta_{d+1} + \Theta_d, \text{ for } d \in \{1, \cdots, x-2\} \tag{6}$$

$$\Delta^2\Theta_d|\tau_\theta \quad \overset{\text{i.i.d.}}{\sim} \quad \mathcal{N}(0, \tau_\theta^{-1}), \text{ for } d \in \{1, \cdots, x-2\}, \tag{7}$$

$$\tau_\theta \quad \sim \quad \mathscr{PC}(z, 0.01). \tag{8}$$

We assign a Penalized Complex (PC) prior to the global precision parameter τ_θ:

$$\tau_\theta \quad \sim \quad \mathscr{PC}(z, 0.01). \tag{9}$$

where z is the standard deviation of y_i. The PC prior is a vague prior. (24) documented the PC prior specification in detail.

Figure 2 illustrates the RW2 model results for Θ_d for age groups 5–9, 10–14, 15–19 and 20–24. The estimated model results (in purple) align with the empirical relationships demonstrated in loess curves. When developing our estimates, we explored modelling Θ_d on a regional level instead of on a global level. We decided against the use of a regional model for three reasons. First, some outlying countries with specific discrimination practices or distinct cause-specific mortality patterns would no longer be apparent, as we would lose the comparison with other countries in other regions with the same mortality level. Second, it was not easy to interpret the regional trends, and we could no

longer examine national deviations in the same consistent framework between birth and 25th birthday since the model for U5MR uses a global reference. Third, the uncertainty around estimates was slightly increased with a regional model.

$\Phi_{c,t}$ models the discrepancy between $\Psi_{c,t}$ and global trend Θ_d. We use a first-order random walk (RW1) model for $\Phi_{c,t}$ and assign a PC prior to country-specific precision parameter $\tau_{\phi,c}$:

$$\begin{aligned}
\Delta\Phi_{c,t} &= \Phi_{c,t+1} - \Phi_{c,t}, \text{ for } c \in \{1,\cdots,k\}, t \in \{1,...,j-1\} && (10)\\
\Delta\Phi_{c,t}|\tau_{\phi,c} &\overset{\text{i.i.d.}}{\sim} \mathscr{N}(0,\tau_{\phi,c}^{-1}), \text{ for } c \in \{1,\cdots,k\}, t \in \{1,...,j-1\}, && (11)\\
\tau_{\phi,c} &\sim \mathscr{PC}(1,0.01), \text{ for } c \in \{1,\cdots,k\}. && (12)
\end{aligned}$$

The sum-to-zero constraint is imposed on $\Phi_{c,t}$ so that the precision matrix is full-rank:

$$\sum_{t=1}^{j} \Phi_{c,t} = 0, \text{ for } c \in \{1,\cdots,k\}. \tag{13}$$

3.3 Model summary

Notations Table 5 summarizes the notation and indexes used in Section 3.1 and Section 3.2.

Data model

$$\begin{aligned}
y_i &= \Psi_{c[i],t[i]} + \varepsilon_i, \text{ for } i \in \{1,\cdots,n\},\\
\varepsilon_i|\omega_{s[i]} &\sim \mathscr{N}(0, u_i^2 + \omega_{s[i]}^2),\\
\omega_s &= 0, \text{ for } s = m.
\end{aligned}$$

Process model

$$\begin{aligned}
\Psi_{c,t} &= \Theta_{d[c,t]} + \Phi_{c,t},\\
\Delta^2\Theta_d &= \Theta_{d+2} - 2\Theta_{d+1} + \Theta_d, \text{ for } d \in \{1,\cdots,x-2\}\\
\Delta^2\Theta_d|\tau_\theta &\overset{\text{i.i.d.}}{\sim} \mathscr{N}(0,\tau_\theta^{-1}), \text{ for } d \in \{1,\cdots,x-2\},\\
\Delta\Phi_{c,t} &= \Phi_{c,t+1} - \Phi_{c,t}, \text{ for } c \in \{1,\cdots,k\}, t \in \{1,...,j-1\}\\
\Delta\Phi_{c,t}|\tau_{\phi,c} &\overset{\text{i.i.d.}}{\sim} \mathscr{N}(0,\tau_{\phi,c}^{-1}), \text{ for } c \in \{1,\cdots,k\}, t \in \{1,...,j-1\}.
\end{aligned}$$

Prior distributions Non-informative priors are assigned to hyper-parameters:

$$\begin{aligned}
\tau_{\omega,s} = \omega_s^{-2} &\sim \mathscr{PC}(z,0.01), \text{ for } s \in \{1,\cdots,m-1\},\\
\tau_\theta &\sim \mathscr{PC}(z,0.01),\\
\tau_{\phi,c} &\sim \mathscr{PC}(1,0.01), \text{ for } c \in \{1,\cdots,k\}.
\end{aligned}$$

Statistical computation We use the Integrated Nested Laplace Approximation (INLA) for Bayesian inference (28). We use the R-package `R-INLA` (29) to implement the INLA estimation procedure. The statistical source code is available upon request.

3.4 Derivation of sex-specific mortality

For each country-year, we calculated the sex-specific mortality by fulfilling (i) the number of female and male deaths sum up to the total number of deaths based on the life-table method; and (ii) the ratio of male to female mortality equals the sex ratio estimates from the Bayesian model. Given the total mortality rates $Q_{c,t}$ and the estimated sex ratio

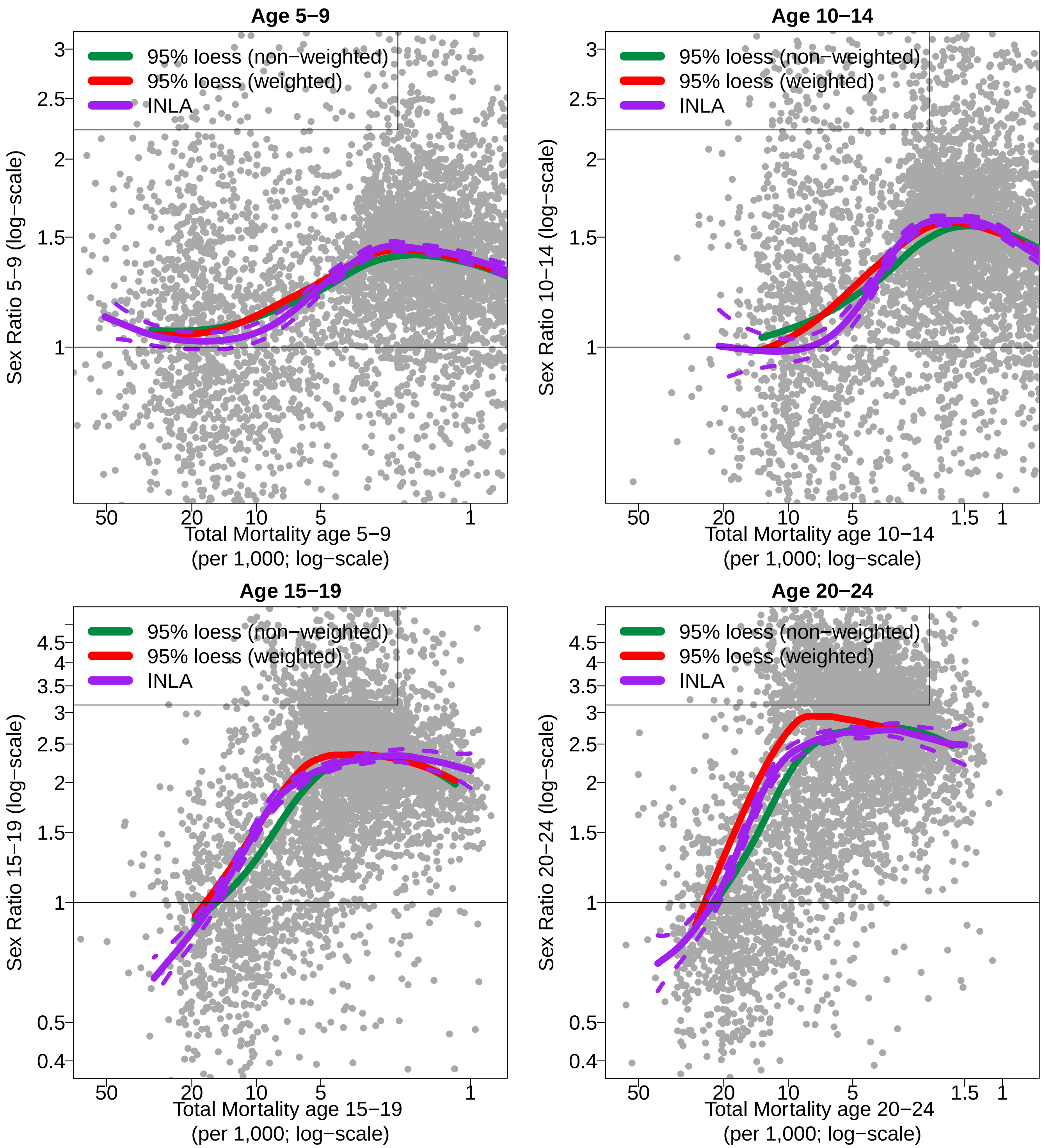

Figure 2: **Global relation between total mortality and sex ratio.** Green curves are loess fittings (using a re-descending M estimator with Tukey's biweight function). Red curves are symmetric loess by weighting data points by the reverse of their sampling error variances. Purple solid curves are the mean of the model fittings using the RW2 model, and dashed curves are 90% uncertainty intervals.

Symbol	Description
	Indexes
i	Indicator for observations across all country-years, $i \in \{1, \cdots, n\}$.
c	Indicator for country, $c \in \{1, \cdots, k\}$, where $k = 200$. $c[i]$ refers to the country index of observation i.
t	Indicator for year, $t \in \{1, \cdots, j\}$, where $j = 72$. $t = 1$ corresponds to the year 1950 and $t = j$ to the year 2021. $t[i]$ refers to the year index of observation i.
s	Indicator for data source type, $s \in \{1, \cdots, m\}$, where $m = 3$. $s = 1$ corresponds to data source type DHS, $s = 2$ to others, and $s = 3$ to VR/SRS.
d	Indicator for the dth unique value of the total mortality rate κ_d and corresponding global expected sex ratio Θ_d. $d[c,t]$ refers to the index based on total mortality in country-year c,t. More explanations are in the row of κ_d.
	Known quantities
z_i	ith observed sex ratio of mortality rate for country $c[i]$ in year $t[i]$ with data source type $s[i]$.
y_i	$y_i = \log(z_i)$.
u_i^2	The ith sampling error variance for y_i.
z	The standard deviation of y_i.
$Q_{c,t}$	The total mortality rate for country c in year t (20).
$V_{c,t}$	$V_{c,t} = \log(Q_{c,t})$
κ_d	Grid of $V_{c,t}$ where Θ_d is evaluated for $d \in \{1, \cdots, x\}$, and $x = 260$. $\kappa_1 = \log(1/1000)$, i.e. log of 1 death per 1000 population (same κ_1 value for all age groups). κ_x is the 99th percentile of $V_{c,t}$ across all country-years with data (different κ_x values across age groups).
	Unknown parameters
$\Psi_{c,t}$	The true log of sex ratio of mortality rate for country c, year t.
$\Theta_{d[c,t]}$	The global expected sex ratio evaluated at κ_d.
$\Phi_{c,t}$	The year-by-year within country discrepancy between $\Psi_{c,t}$ and Θ_d.
$\tau_{\omega,s}$	The precision parameter for non-sampling error variance with data source type s.
τ_θ	The precision parameter for the second-order increment $\Delta^2\Theta_d$.
$\tau_{\phi,c}$	The country-specific precision parameter for the first-order increment $\Delta\Phi_{c,t}$ for country c.

Table 5: **Notation summary.**

of the mortality rates $\Psi_{c,t}$, we derive the sex-specific mortality rates as shown in Table 6. In this subsection, we omit the indexes for country c and year t and use superscripts $[F]$ and $[M]$ for male- and female-specific indicators.

To incorporate the uncertainties in the sex ratio of mortality, total mortality, and population sizes, the calculation steps in Table 6 are applied to each posterior sample of:

- Each posterior sample of the log of sex ratio Ψ: from the Bayesian model.
- Each trajectory of the total mortality Q: from (27) (not part of this modelling process).
- Each trajectory of the numbers of the total and male population (N and $N^{[M]}$) at the beginning of a 5-year age group: from the UN WPP (4) (not part of this modelling process).

Table 6: **Derivation of sex-specific mortality rate for a 5-year age group.**

Step #	Description
1:	Initialize female and male mortality rates ${}^{(0)}Q^{[F]}$ and ${}^{(0)}Q^{[M]}$ with based on total mortality Q and log of sex ratio Ψ, and the proportion of males $N^{[M]}$ in the whole population N at the beginning of a certain age group (e.g. for age group 15–19, the beginning of the age group is age 15): $w = N^{[M]}/N$: ${}^{(0)}Q^{[M]} = Q/(w+(1-w)/\exp\{\Psi\})$, ${}^{(0)}Q^{[F]} = {}^{(0)}Q^{[M]}/\exp\{\Psi\}$,
2:	Following the life table calculation, we compute the central rate of mortality[4] for the female, male, and total population (denoted as $m^{[F]}$, $m^{[M]}$, and m respectively): ${}^{(0)}m^{[F]} = {}^{(0)}Q^{[F]}/(5(1-{}^{(0)}Q^{[F]}/2))$ ${}^{(0)}m^{[M]} = {}^{(0)}Q^{[M]}/(5(1-{}^{(0)}Q^{[M]}/2))$ ${}^{(0)}m = ({}^{(0)}m^{[F]}N^{[F]} + {}^{(0)}m^{[M]}N^{[M]})/N$ We use the usual approximation to compute m values by assuming the average population size in each age group within one year is $N(1-Q^*)$, where Q^* is $Q^{[F]}$ or $Q^{[M]}$. The average population size for a 5-year age group within one year is approximated by $5N(1-Q^*)$.
3:	Compute the total mortality rate based on initial values: ${}^{(0)}Q = 5\,{}^{(0)}m/(1+(5-5/2)\,{}^{(0)}m)$
4:	Compute the relative difference between the given total mortality Q with the calculated total mortality ${}^{(0)}Q$: ${}^{(0)}re = \lvert {}^{(0)}Q - Q\rvert/Q$
5:	**if** ${}^{(0)}re < 10^{-8}$ **then**
6:	**stop** Set ${}^{(0)}Q^{[F]}$ and ${}^{(0)}Q^{[M]}$ as the final sex-specific mortality rates. **else**
7:	**for** $g \in \{1,\cdots,G\}$ **do**
8:	Simulate 10^5 equally distributed male mortality rate within the interval $\left[{}^{(g-1)}Q^{[M]} - {}^{(g-1)}Q^{[M]}/10^{-3}, {}^{(g-1)}Q^{[M]} + {}^{(g-1)}Q^{[M]}/10^{-3}\right]$. Denote each simulation as ${}^{(g)}Q_s^{[M]}$ for $s \in \{1,\cdots,10^5\}$.
9:	Simulations for female mortality rates are: ${}^{(g)}Q_s^{[F]} = {}^{(g)}Q_s^{[M]}/\Psi$ for $s \in \{1,\cdots,10^5\}$.
10:	Repeat steps 2–6 until ${}^{(g)}re < 10^{-8}$.

3.5 Derivation of sex ratio for 10-year age groups

This subsection explains how we derived the sex ratio for 15–24 based on sex ratios 15–19 and 20–24. The same procedures apply to deriving the sex ratio for 5–14 based on sex ratios for 5–9 and 10–14.

The sex ratio for mortality rate from age group 15–24 is calculated from $S_{1,c,t}$ and $S_{2,c,t}$ through standard cohort equations. Sex-specific mortality rates for age groups 15–19 ($a=1$) and 20–24 ($a=2$) are derived as follows (omitting

[4] It is approximately equal to the average number of deaths each year at age x last birthday, divided by the average population at that age in the same year.

country c and year t subscripts, and using superscripts $[F]$ and $[M]$ for male- and female-specific indicators):

$$Q_a^{[M]} = \frac{Q_a}{w_a + \frac{1-w_a}{S_a}}, \text{ for } a \in \{1,2\},$$
$$Q_a^{[F]} = \frac{Q_a^{[M]}}{S_a}, \text{ for } a \in \{1,2\}.$$

w_1 refers to the proportion of males at the age of 15. w_2 indicates the ratio of male survivors to age 19 over the total number of survivors up to age 19:

$$w_1 = \frac{N_1^{[M]}}{N_1},$$
$$w_2 = \frac{N_2^{[M]}}{N_2} = \frac{w_1(1-Q_1^{[M]})}{1-Q_1},$$

where N_1 refers to the total population at age 15, and N_2 is the number of survivors up to age 19 (approximated by the population at age 15 times the mortality rate for age group 15–19).

Given the mortality rates for age groups 15–19 and 20–24 by sex, the sex-specific mortality rates for age group 15–24 are calculated as follows:

$$Q_3 = 1-(1-Q_1)(1-Q_2),$$

for males and females, which are then used to produce sex ratios of mortality rate for the age group 15–24:

$$S_{3,c,t} = \frac{Q_{3,c,t}^{[M]}}{Q_{3,c,t}^{[F]}}.$$

3.6 Computation of final results

Throughout the project, we presented our results in the format of "point estimate [lower bound; upper bound]". E.g. the age-sex-specific mortality rate and deaths are presented as $Q_{a,c,t}^{P.E.}[Q_{a,c,t}^{L};Q_{a,c,t}^{U}]$ and $D_{a,c,t}^{P.E.}[D_{a,c,t}^{L};D_{a,c,t}^{U}]$ respectively. We use superscripts $[F]$ and $[M]$ for male- and female-specific indicators. This section will explain how we derived each component in the result.

3.6.1 Constructing posterior samples of outcomes that include crisis-related deaths and uncertainty in national-level mortality rate

All the indicators in this section are country-year-specific. Hence, we omit the indexes c and t for country and year for simplicity.

The sex-specific mortality rates and related deaths were adjusted in country-years to account for abrupt increases in mortality due to conflicts and disasters, which would otherwise not be present in the smoothed mortality curves obtained from the statistical model. No specific adjustment was made for the coronavirus disease 2019 (COVID-19) pandemic because the UN IGME did not find sufficient evidence to warrant a systematic adjustment for national both-sex estimates based on available data from over 110 countries in 2020 and over 80 countries in 2021 (27). Moreover, estimates from vital statistics data already reflected the potential disruptions introduced by the pandemic. Data were lacking in other countries without vital statistics to conclude on effects of the pandemic on sex differences in mortality below the age of 25.

Table 12 lists all the country-years with crisis adjustments by age groups. Crisis adjustments were made during the identified crisis periods in 36 countries for ages 0–4, 53 countries for ages 5–14, and 48 countries for ages 15–24 to account for crisis-related deaths.

The following criteria were used to identify crises:

1. The crisis was isolated to a few years,
2. Crisis deaths in each age group were $>$10% of non-crisis deaths in this age group,

3. The crisis-specific risk of dying was greater than 0.2 per 1,000,

4. The number of crisis deaths in each age group was greater than 10.

To include crisis-related deaths in the crisis-free sex-specific estimates obtained from the model, we followed the procedures used by the UN IGME for adjusting national-level mortality rates (30; 27). The g-th posterior sample for sex-specific mortality rate including crisis-related deaths, denoted as ${}^{(g)}Q(\text{full})_a^{[\text{sex}]}$, is defined as follows for sex $\in \{F, M\}$:

$$
{}^{(g)}Q(\text{full})_a^{[\text{sex}]} = \begin{cases} {}^{(g)}Q(\text{crisis-free})_a^{[\text{sex}]} + Q(\text{crisis})_a^{[\text{sex}]}, & \text{if affected by crisis} \\ {}^{(g)}Q(\text{crisis-free})_a^{[\text{sex}]} & \text{o.w.} \end{cases}
$$

$Q(\text{crisis-free})_a^{(g)}$ is the g-th posterior sample of crisis-free country-year-age-sex-specific mortality rate that includes uncertainty in national-level mortality (Section 3.4).

$Q(\text{crisis})_a^{[\text{sex}]}$ is the country-year-age-sex-specific mortality rate due to crisis. No uncertainty of crisis-related mortality rate has been included in the final adjusted sex-specific mortality rate results. Age-sex distributions for conflict deaths were applied to estimates of total conflict deaths by country-year based on available distributions of conflict deaths by age and sex for specific conflicts (31). Estimated deaths for major natural disasters up to and including 2021 were obtained from the Emergency Events Database (EM-DAT), the International Disaster Database, which is maintained by the Centre for Research on the Epidemiology of Disasters (CRED)(32). Global Health Estimates (GHE) age-sex distributions were used. These are based on several studies of earthquake and tsunami deaths (31). Deaths due to armed conflict and violence are from the Armed Conflict Location and Event Data Project (ACLED) (33), the Uppsala Conflict Data Program (UCDP) for Battle-Related Deaths, Non-State Conflict Dataset (34), and One-sided Violence Dataset (34).

Posterior samples for the sex ratio of mortality rates (with crisis-related deaths), inclusive of uncertainty in the national-level death (including crisis-related deaths), were given by:

$$
{}^{(g)}\exp\{\Psi(\text{full})\}_a = \frac{{}^{(g)}Q(\text{full})_a^{[\text{M}]}}{{}^{(g)}Q(\text{full})_a^{[\text{F}]}}.
$$

3.6.2 Rounding

We kept three significant figures for all reported estimates and uncertainty intervals. We round the number of sex-specific deaths for each country-year to its nearest integer.

3.6.3 Constructing uncertainty intervals and point estimates

All the indicators in this section are country-year-specific. Hence, we omit the indexes c and t for country and year for simplicity. The 90% uncertainty intervals for $\Psi(\text{full})_a$, $Q(\text{full})_a$, and $D(\text{full})_a$, denoted as $[*^L; *^U]$, are the 5th and 95th percentiles of the corresponding posterior samples:

$$
\begin{aligned}
^L &= \text{percentile}_{5\%}\left\{{}^{(1)}, \ldots, {}^{(G)}*\right\}, \\
^U &= \text{percentile}_{95\%}\left\{{}^{(1)}, \ldots, {}^{(G)}*\right\}.
\end{aligned}
$$

where $*$ can be substituted with $\Psi(\text{full})_a$, $Q(\text{full})_a$, and $D(\text{full})_a$.

The point estimates $\exp\{\Psi(\text{full})\}_a^{\text{P.E.}}$ are combined with the point estimates of total mortality rate $Q(\text{full})_a^{\text{P.E.}}$ and the number of total deaths $D(\text{full})_a^{\text{P.E.}}$ (both include crisis-related deaths and were obtained from the UN IGME 2023 results (27), not part of the modelling process) to derive the point estimates of sex-specific mortality $Q(\text{full})_a^{\text{P.E.[sex]}}$ and point estimates of sex-specific deaths $D(\text{full})_a^{\text{P.E.[sex]}}$ using the same procedure in Section 3.4.

Sex-specific deaths to be consistent with total deaths Due to rounding (Section 3.6.2) and the birth-week cohort method, the sum of the sex-specific deaths for a particular country-year $D(\text{full})_a^{\text{P.E.[F]}} + D(\text{full})_a^{\text{P.E.[M]}}$ may be slightly different than the total deaths for the same country-year $D(\text{full})_a^{\text{P.E.}}$, where total deaths computation is not part of the modelling process. When the inconsistency occurs for a certain country-year, we take the difference between $D(\text{full})_a^{\text{P.E.[F]}} + D(\text{full})_a^{\text{P.E.[M]}}$ and $D(\text{full})_a^{\text{P.E.}}$ and distribute it proportionally to the sex-specific deaths. This adjustment is typically small but ensures that $D(\text{full})_a^{\text{P.E.[F]}} + D(\text{full})_a^{\text{P.E.[M]}} = D(\text{full})_a^{\text{P.E.}}$ for all country-years. We adjust point estimates but not the uncertainty intervals.

3.6.4 Imputing results for countries without data

Estimates for countries without data were imputed from the model assumptions and parameter estimates. They were based on the expected sex ratios (determined by the national-level mortality rate for that country), the uncertainty in country-specific deviations based on simulations of the country-year-specific multiplier (that captured the variability unexplained by the expected sex ratios), and the uncertainty in national-level mortality rates.

4 Validation exercise and results

We validate the model performance by leaving out data collected after a particular year, referred to as the "survey year", rather than randomly leaving out data. This validation approach has been used in various previous global health and population studies (35; 23; 36; 37; 38; 39; 40; 41). The left-out observations are roughly 20% of the total observations (due to the varying number of observations collected in each survey year, the left-out observations can be slightly above or below 20%). We call the left-out observations "testing dataset" and the remaining observations "training dataset".

For each left-out observation in the testing dataset, we generate a posterior predictive distribution based on the Bayesian model fittings for the training dataset. We compute the error as the difference between the left-out observation and the median of the posterior predictive distribution. We report the median errors of all the left-out observations and the median of the absolute errors. We also report the coverage of the 90% prediction intervals (PIs). The lower and upper bound of the 90% PI for each left-out observation is the 5th and 95th percentiles of the posterior predictive distribution. To summarise the coverage, we compute the proportion of left-out observations that fall outside the 90%

We also validate the model performance by comparing the model estimates based on the full and training datasets. We compute the error for each country-year as the difference between the median estimates based on the full dataset and those based on the training dataset. We report the median of these errors and absolute errors for all the country-years. We check the percentage of the country-years where the median estimates based on the full dataset fall outside the credible bounds of the estimates based on the training dataset. Table 8 shows the results for the comparison between estimates obtained based on the full dataset and estimates based on the training set. Median errors and the median absolute errors were close to zero, and the proportion of updated estimates that fell outside the uncertainty intervals constructed based on the training set was small.

**Age group**	**5–9**	**10–14**	**15–19**	**20–24**
Median error	-0.04	-0.07	-0.04	-0.02
Median absolute error	0.30	0.33	0.35	0.40
% of left-out observations below 90% prediction interval	4.3	5.5	3.8	3.5
% of left-out observations above 90% prediction interval	4.0	2.8	1.6	1.0
**Expected proportions (%)**	**5**	**5**	**5**	**5**
# left out observations	1,083	1,061	965	961
% of left out to total observations	20.6	20.6	20.1	20.1
Survey year left out	2013	2013	2013	2013

Table 7: **Validation results for left-out observations by age group.** Errors are defined as the difference between a left-out observation and the posterior median of its predictive distribution. Survey year left out: left out all observations that were collected in or after the year.

**Age group**	**5–9**			**10–14**		
**Year**	**2000**	**2005**	**2010**	**2000**	**2005**	**2010**
Median error	-0.00	-0.00	-0.01	-0.00	-0.00	-0.01
Median absolute error	0.01	0.01	0.02	0.01	0.02	0.03
Below 90% uncertainty interval (%)	0.0	0.0	0.5	0.0	0.5	1.0
Above 90% uncertainty interval (%)	0.0	0.0	0.0	0.0	0.0	0.0
**Expected proportions (%)**	**≤5**	**≤5**	**≤5**	**≤5**	**≤5**	**≤5**
**Age group**	**15–19**			**20–24**		
**Year**	**2000**	**2005**	**2010**	**2000**	**2005**	**2010**
Median error	-0.00	-0.00	-0.00	-0.00	-0.00	-0.01
Median absolute error	0.01	0.01	0.03	0.01	0.02	0.04
Below 90% uncertainty interval (%)	0.0	0.5	1.5	0.0	1.0	1.0
Above 90% uncertainty interval (%)	0.0	0.0	0.0	0.0	0.0	0.0
**Expected proportions (%)**	**≤5**	**≤5**	**≤5**	**≤5**	**≤5**	**≤5**

Table 8: **Summary of differences in sex ratio estimates in observation years 2000, 2005, and 2010 based on the training and full data sets.** Errors are the differences between estimates based on the full dataset and the training set. The proportions refer to the proportions (%) of countries where the median sex ratio estimates based on the full data set fall below or above their corresponding 90% uncertainty intervals based on the training dataset. The results are broken down by age groups and observation years.

5 Global expected sex ratios by age group

Figure 3 illustrates the expected sex ratios for total mortality based on the global relationship between mortality levels and sex ratios for the 0-4, 5-14, and 15-24 age groups. Similar patterns occur for all age groups; as the total mortality declines, the sex ratio initially increases until it reaches a maximum, followed by a slight reduction when the total mortality further decreases.

6 Discussion on adding covariates in Bayesian models

Our main objective with this study was to produce robust estimates of sex-specific mortality using a model that does not use covariates. We could have included generic covariates such as GDP per capita, adult literacy rate, indicators related to cause-of-death distribution such as the prevalence of HIV (42) or maternal mortality ratio (43), and sex-specific indicators such as the difference between female and male expected years of schooling or the male and female estimated earned income, sex-specific mortality related/due to infectious diseases, especially zoonotic diseases (44; 45), or information on sex differentials in health status such as nutrition, immunisation (46). This could have improved the estimation, but many aforementioned covariates are unavailable for all countries worldwide or result from complex modelling. Furthermore, the set of covariates would need to change for each age group. In addition, the resulting estimates would no longer be helpful to pinpoint countries with outlying sex ratios (as covariates could affect this outlying situation), and we would no longer be in a position to relate the sex ratios to these potential drivers (as this would be circular).

With our parsimonious model, we only make use of a strong and well-established relationship between overall mortality and the sex ratio, and we can, therefore (1) coherently estimate sex ratios from birth to age 25, and (2) produce robust estimates of sex-specific mortality which can now be analysed in relation to these drivers.

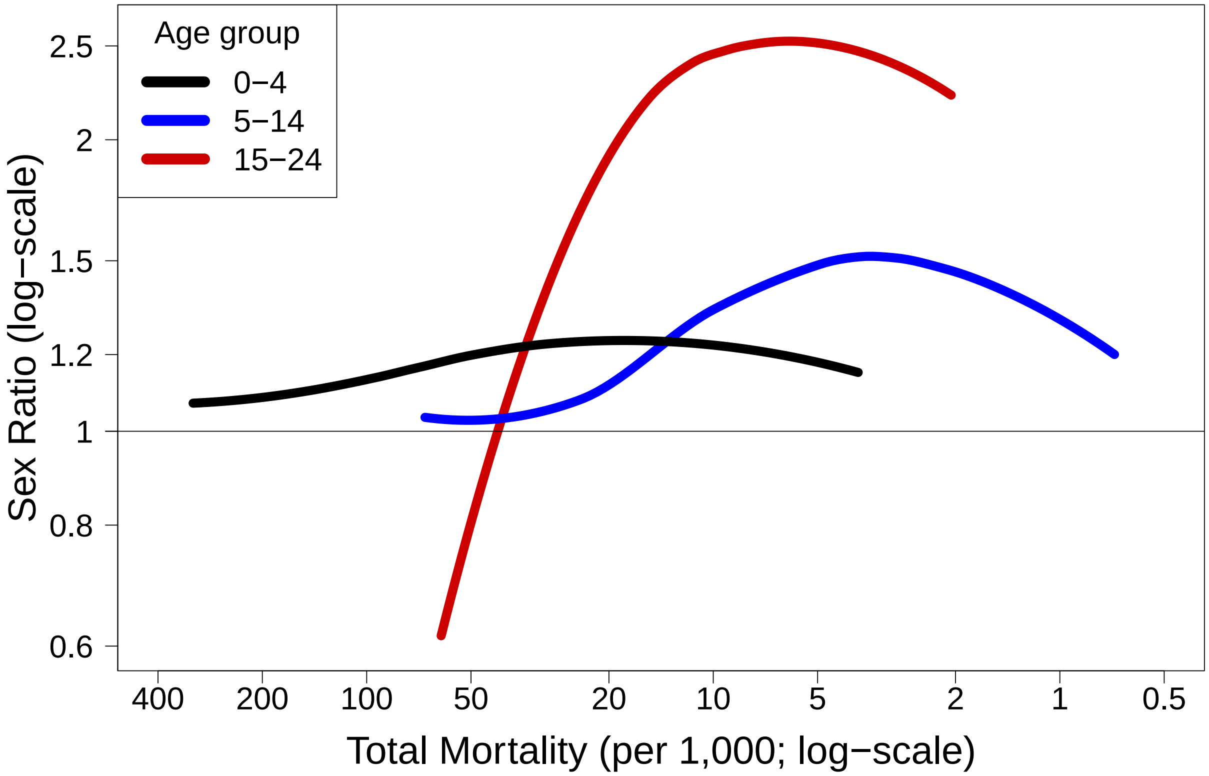

Figure 3: **Overview of the global relations between sex ratios and total mortality levels.** The curves illustrate the relation between sex ratios and total mortality based on the Bayesian models.

7 GATHER Compliance

Guidelines for Accurate and Transparent Health Estimates Reporting (GATHER) are reported in Table 9.

Table 9: **Checklist of information that should be included in new reports of global health estimates.** M: main manuscript. A: Appendix. Section titles after "M" are stated for the main manuscript. Page numbers after "A" are shown for the appendix.

Item #	Checklist item	Reported on page # or section #
**Objectives and funding**		
1	Define the indicator(s), populations (including age, sex, and geographic entities), and time period(s) for which estimates were made.	M-Introduction
2	List the funding sources for the work.	M-Summary
**Objectives and funding**		
For all data inputs from multiple sources that are synthesized as part of the study:		
3	Describe how the data were identified and how the data were accessed.	M-Database construction, A1–4
4	Specify the inclusion and exclusion criteria. Identify all ad-hoc exclusions.	M-Database construction, A4
5	Provide information on all included data sources and their main characteristics. For each data source used, report reference information or contact name/institution, the population represented, data collection method, year(s) of data collection, sex and age range, diagnostic criteria or measurement method, and sample size, as relevant.	A69–200
6	Identify and describe any categories of input data that have potentially important biases (e.g., based on characteristics listed in item 5).	A1–4
For data inputs that contribute to the analysis but were not synthesized as part of the study:		
7	Describe and give sources for any other data inputs.	M-Database construction, A1, A69–200
For all data inputs:		
8	Provide all data inputs in a file format from which data can be efficiently extracted (e.g., a spreadsheet rather than a PDF), including all relevant meta-data listed in item 5. For any data inputs that cannot be shared because of ethical or legal reasons, such as third-party ownership, provide a contact name or the name of the institution that retains the right to the data.	M-Database construction
**Data analysis**		
9	Provide a conceptual overview of the data analysis method. A diagram may be helpful.	M-Database construction, A5
10	Provide a detailed description of all analysis steps, including mathematical formulae. This description should cover, as relevant, data cleaning, data pre-processing, data adjustments and weighting of data sources, and mathematical or statistical model(s).	M-Statistical analysis, A1–11
11	Describe how candidate models were evaluated and how the final model(s) were selected.	M-Statistical analysis, A13
12	Provide the results of an evaluation of model performance, if done, as well as the results of any relevant sensitivity analysis.	A13–14

Table 9 – continued from previous page

Item #	Checklist item	Reported on page # or section #
13	Describe methods for calculating the uncertainty of the estimates. State which sources of uncertainty were, and were not, accounted for in the uncertainty analysis.	A11–13
14	State how analytic or statistical source code used to generate estimates can be accessed.	M-Statistical analysis
**Results and Discussion**		
15	Provide published estimates in a file format from which data can be efficiently extracted.	A24–68
16	Report a quantitative measure of the uncertainty of the estimates (e.g., uncertainty intervals).	A24–68
17	Interpret results in light of existing evidence. If updating a previous set of estimates, describe the reasons for changes in estimates.	M-Results
18	Discuss limitations of the estimates. Include a discussion of any modelling assumptions or data limitations that affect the interpretation of the estimates.	M-Discussion

8 Supplementary tables

UNICEF Region	Country
South Asia	Afghanistan; Bangladesh; Bhutan; India; Maldives; Nepal; Pakistan; Sri Lanka
Europe and Central Asia	Albania; Andorra; Armenia; Austria; Azerbaijan; Belarus; Belgium; Bosnia and Herzegovina; Bulgaria; Croatia; Cyprus; Czech Republic; Denmark; Estonia; Finland; France; Georgia; Germany; Greece; Hungary; Iceland; Ireland; Italy; Kazakhstan; Kosovo; Kyrgyzstan; Latvia; Lithuania; Luxembourg; Macedonia; Malta; Republic of Moldova; Monaco; Montenegro; Montserrat; Netherlands; Norway; Poland; Portugal; Romania; Russian Federation; San Marino; Serbia; Slovakia; Slovenia; Spain; Sweden; Switzerland; Tajikistan; Turkey; Turkmenistan; Ukraine; United Kingdom; Uzbekistan
Middle East and North Africa	Algeria; Bahrain; Egypt; Iran (Islamic Republic of); Iraq; Israel; Jordan; Kuwait; Lebanon; Libya; Morocco; Oman; Qatar; Saudi Arabia; State of Palestine; Syria; Tunisia; United Arab Emirates; Yemen
Sub-Saharan Africa	Angola; Benin; Botswana; Burkina Faso; Burundi; Cameroon; Cape Verde; Central African Republic; Chad; Comoros; Congo; Democratic Republic of the Congo; Cote d'Ivoire; Djibouti; Equatorial Guinea; Eritrea; Ethiopia; Gabon; The Gambia; Ghana; Guinea; Guinea-Bissau; Kenya; Lesotho; Liberia; Madagascar; Malawi; Mali; Mauritania; Mauritius; Mozambique; Namibia; Niger; Nigeria; Rwanda; Sao Tome and Principe; Senegal; Seychelles; Sierra Leone; Somalia; South Africa; South Sudan; Sudan; Swaziland; Tanzania; Togo; Uganda; Zambia; Zimbabwe
Latin America and Caribbean	Anguilla; Antigua and Barbuda; Argentina; Bahamas; Barbados; Belize; Bolivia (Plurinational State of); Brazil; British Virgin Islands; Chile; Colombia; Costa Rica; Cuba; Dominica; Dominican Republic; Ecuador; El Salvador; Grenada; Guatemala; Guyana; Haiti; Honduras; Jamaica; Mexico; Nicaragua; Panama; Paraguay; Peru; Saint Kitts and Nevis; Saint Lucia; Saint Vincent and the Grenadines; Suriname; Trinidad and Tobago; Turks and Caicos Islands; Uruguay; Venezuela (Bolivarian Republic of)
East Asia and Pacific	Australia; Brunei; Cambodia; China; Cook Islands; Federated States of Micronesia; Fiji; Indonesia; Japan; Kiribati; Democratic People's Republic of Korea; Republic of Korea; Lao People's Democratic Republic; Malaysia; Marshall Islands; Mongolia; Myanmar; Nauru; New Zealand; Niue; Palau; Papua New Guinea; Philippines; Samoa; Singapore; Solomon Islands; Thailand; Timor Leste; Tonga; Tuvalu; Vanuatu; Vietnam
North America	Canada; United States of America

Table 10: **Classification of countries by UNICEF regions.**

Table 11: **Completeness of death registration for intercensal periods.**

Country	First census	Second census	Males	Females	Both sexes
Albania	1989	2001	1.00	1.00	1.00
Albania	2001	2011	1.00	1.00	1.00
Algeria	1998	2008	1.00	1.00	1.00
Antigua and Barbuda	2001	2011	1.00	1.00	1.00
Argentina	1991	2001	1.00	1.00	1.00
Argentina	2001	2010	1.00	1.00	1.00
Armenia	1989	2001	1.00	1.00	1.00
Armenia	2001	2011	1.00	1.00	1.00
Azerbaijan	1989	1999	0.93	0.86	0.90
Azerbaijan	1999	2009	0.92	0.93	0.93
Bahamas	1990	2000	0.81	0.81	0.81
Bahamas	2000	2010	0.90	0.89	0.89
Bahrain	1991	2001	1.00	1.00	1.00
Bahrain	2001	2010	0.92	0.89	0.91
Barbados	2000	2010	1.00	1.00	1.00
Belize	1991	2000	1.00	1.00	1.00
Belize	2000	2010	1.00	0.91	0.93
Brazil	1991	2000	1.00	1.00	1.00
Brazil	2000	2010	0.93	0.92	0.92
Brunei Darussalam	1991	2011	1.00	1.00	1.00
Cape Verde	1990	2000	0.86	1.00	0.93
Cape Verde	2000	2010	1.00	1.00	1.00
Colombia	1993	2005	0.95	1.00	1.00
Cook Islands	1996	2006	1.00	1.00	1.00
Costa Rica	1984	2000	1.00	0.90	1.00
Costa Rica	2000	2011	1.00	0.92	1.00
Cuba	1981	2002	1.00	1.00	1.00
Cuba	2002	2012	1.00	1.00	1.00
Cyprus	1992	2001	1.00	1.00	1.00
Cyprus	2001	2011	0.88	0.87	0.88
Dominica	1991	2001	1.00	0.92	1.00
Dominica	2001	2011	1.00	1.00	1.00
Dominican Republic	2002	2010	0.54	0.53	0.54
Ecuador	1990	2001	1.00	1.00	1.00
Ecuador	2001	2010	0.66	0.66	0.66
Egypt	1996	2006	1.00	1.00	1.00
Egypt	2006	2017	0.90	1.00	1.00
El Salvador	1992	2007	1.00	1.00	1.00
Fiji	2007	2017	0.93	1.00	1.00
Georgia	1989	2002	0.91	0.80	0.85
Georgia	2002	2014	1.00	1.00	1.00
Grenada	1981	2001	0.79	0.84	0.82
Grenada	2001	2011	1.00	1.00	1.00
Guatemala	1981	2018	1.00	1.00	1.00
Guyana	1991	2002	1.00	1.00	1.00
Guyana	2002	2012	1.00	1.00	1.00

Continued on next page

Table 11 – continued from previous page

Country	First census	Second census	Males	Females	Both sexes
Iran (Islamic Republic of)	1996	2006	0.44	0.44	0.44
Iran (Islamic Republic of)	2006	2011	0.84	0.84	0.84
Jamaica	1991	2001	0.82	0.90	0.86
Jamaica	2001	2011	0.90	1.00	0.93
Jordan	2004	2015	0.49	0.55	0.51
Kazakhstan	1999	2009	1.00	1.00	1.00
Kiribati	1995	2005	0.50	0.40	0.45
Kuwait	1995	2005	1.00	1.00	1.00
Kyrgyzstan	1989	1999	1.00	1.00	1.00
Kyrgyzstan	1999	2009	1.00	1.00	1.00
Malaysia	1991	2000	1.00	1.00	1.00
Malaysia	2000	2010	1.00	1.00	1.00
Maldives	1985	1995	1.00	0.93	1.00
Maldives	1995	2006	1.00	1.00	1.00
Malta	1995	2005	1.00	0.94	1.00
Marshall Islands	1988	1999	0.74	0.46	0.60
Mauritius	1990	2000	1.00	1.00	1.00
Mauritius	2000	2011	1.00	1.00	1.00
Mexico	1990	2000	1.00	1.00	1.00
Mexico	2000	2010	1.00	1.00	1.00
Mongolia	1989	2000	0.88	0.82	0.85
Mongolia	2000	2010	1.00	1.00	1.00
Montenegro	1991	2003	1.00	0.89	1.00
Montenegro	2003	2011	1.00	1.00	1.00
Morocco	1994	2004	0.45	0.25	0.35
Nicaragua	1995	2005	0.79	0.74	0.76
North Macedonia	1991	2002	1.00	0.94	1.00
Panama	1990	2000	0.88	0.87	0.88
Panama	2000	2010	0.90	0.87	0.89
Paraguay	1992	2002	0.76	0.78	0.77
Peru	1993	2007	0.75	0.80	0.77
Peru	2007	2017	0.69	0.78	0.73
Philippines	1990	2000	1.00	1.00	1.00
Philippines	2000	2015	0.91	1.00	1.00
Qatar	1997	2010	1.00	1.00	1.00
Republic of Korea	1985	1995	0.89	0.82	0.86
Republic of Korea	1995	2005	1.00	1.00	1.00
Republic of Korea	2005	2015	0.81	0.77	0.79
Republic of Moldova	1989	2004	1.00	1.00	1.00
Republic of Moldova	2004	2014	1.00	1.00	1.00
Romania	1992	2002	1.00	1.00	1.00
Romania	2002	2011	1.00	1.00	1.00
Saint Kitts and Nevis	1991	2001	1.00	1.00	1.00
Saint Lucia	1991	2001	1.00	1.00	1.00
Saint Vincent and the Grenadines	1991	2001	1.00	1.00	1.00
Saint Vincent and the Grenadines	2001	2012	1.00	1.00	1.00
Serbia	1991	2002	1.00	1.00	1.00

Continued on next page

Table 11 – continued from previous page

Country	First census	Second census	Males	Females	Both sexes
Serbia	2002	2011	0.94	1.00	1.00
Seychelles	1994	2002	1.00	1.00	1.00
Seychelles	2002	2010	1.00	1.00	1.00
Singapore	1990	2000	0.75	0.78	0.77
Singapore	2000	2010	0.83	0.90	0.86
South Africa	1991	2001	1.00	0.82	1.00
South Africa	2001	2011	0.89	0.91	0.90
Sri Lanka	1981	2001	1.00	1.00	1.00
Sri Lanka	2001	2012	1.00	0.90	1.00
Suriname	1980	2004	0.87	0.92	0.89
Suriname	2004	2012	0.93	0.83	0.88
Tajikistan	1989	2000	0.92	0.70	0.80
Tajikistan	2000	2010	1.00	1.00	1.00
Thailand	1990	2000	0.81	0.83	0.82
Thailand	2000	2010	0.83	0.87	0.85
Tonga	1996	2006	0.93	1.00	1.00
Tonga	2006	2016	1.00	1.00	1.00
Trinidad and Tobago	1990	2000	1.00	1.00	1.00
Trinidad and Tobago	2000	2011	1.00	1.00	1.00
Tunisia	1994	2004	0.89	0.81	0.86
Turkey	2000	2011	0.49	0.54	0.51
Turkmenistan	1989	1995	0.94	0.83	0.89
Uruguay	1996	2004	1.00	1.00	1.00
Uruguay	2004	2011	1.00	1.00	1.00
Uzbekistan	1979	1989	0.91	0.85	0.88
Venezuela (Bolivarian Republic of)	1990	2001	1.00	1.00	1.00
Venezuela (Bolivarian Republic of)	2001	2011	0.89	0.85	0.88
Zimbabwe	1992	2002	0.71	0.32	0.48

Table 12: **Country-years with crisis adjustments, by age group.**

Country	Age Group		
	**0–4**	**5–14**	**15–24**
Armenia	1988		
Burundi	1972; 1993	1993	1993
Bangladesh	1970; 1971	1991	1991
Bahamas	2019	2019	2019
Bosnia and Herzegovina	1992; 1993; 1994; 1995	1992; 1993; 1995	1992; 1993; 1994; 1995
Congo	2019	2019	2019
Dominica	1979		
Guatemala	1976; 1982	2005	
Honduras	1974; 1998	1998	1998
Croatia	1991	1991; 1995	1991; 1995
Haiti	2010	2004; 2010; 2021	2010
Indonesia	1965; 2004	2004	2004
Japan	2011	1995; 2011	2011
Kuwait	1990	1990	1990; 1991
Libya	2011	2011	
Sri Lanka	1989; 2004; 2009	2004; 2009	2004
Maldives	2004	2004	2004
Myanmar	2008	2008	2008; 2021
Nicaragua	1972; 1998	1998	1990; 1998
Peru	1970		
State of Palestine	2014	2014	1990; 1995; 2014; 2018; 2021
Rwanda	1994	1994	1994
Sudan	1983; 1984; 1985	2008	2008
Solomon Islands	1975	2007	
El Salvador	1980	1998; 2001; 2009	1990
Sao Tome and Principe	1986		
Swaziland	1983		
Syria	1981; 1982; 2011; 2012; 2013; 2014; 2015; 2016; 2017; 2018; 2019; 2020; 2021	2011; 2012; 2013; 2014; 2015; 2016; 2017; 2018; 2019; 2020; 2021	2011; 2012; 2013; 2014; 2015; 2016; 2017; 2018; 2019; 2020; 2021
Samoa	1964; 2009	2009	2009
Brazil	2016		
Guinea	2014; 2015		
Liberia	2014; 2015	2003; 2014; 2015	1990; 2003; 2015
Venezuela (Bolivarian Republic of)	2015; 2016; 2017; 2018; 2019; 2020; 2021	2017; 2018; 2019; 2020; 2021	2017; 2018; 2019; 2020; 2021
Sierra Leone	2014; 2015		1995
Afghanistan		1998	
Angola		1990; 1992	
Albania		1997	1997
Azerbaijan		1992	2008; 2012; 2020
Bhutan		2000	
Botswana		2006	
Chile		2010	
China		2008	
Cape Verde		1995	1995
Algeria		2003	
Ecuador		2016	
Fiji		2016	
Georgia		1991	1992; 1993; 2008
Guinea-Bissau		1997	1997; 1998; 1999
Iran (Islamic Republic of)		1990; 2003	1990; 2003
Nepal		2015	2002; 2015
New Zealand		2011	
Oman		2007	
Pakistan		2005	2005

Continued on next page

Table 12 – continued from previous page

Country	Age Group		
	**0–4**	**5–14**	**15–24**
Philippines		1991; 2013	
Papua New Guinea		1998	1998
Thailand		2004	
Tajikistan		1992; 1996	2000; 2010; 2011
Turkey		1999	1999; 2016
Central African Republic			2017; 2021
Democratic Republic of the Congo			1996
Ethiopia			1990; 1999
Iraq			2013; 2019
Lebanon			1990; 2006; 2017
Republic of Moldova			1992
Macedonia			2001
Russian Federation			1995
Chad			1990
Trinidad and Tobago			2018
Ukraine			2014

Table 13: **Estimates and 90% uncertainty intervals for sex ratios for IMR in 1990 and 2021, the change in sex ratios from 1990 to 2021, sex-specific IMR in 2021, and ratios of estimated to expected female IMR and their change from 1990 to 2021 for the world, UNICEF regions, and all countries.** ¶: Sex ratio is outlying in 1990. §: Sex ratio is outlying in 2021. *: The ratio of estimated to expected female mortality is significantly different from one. †: Change is significantly different from zero.

	Sex ratio IMR			Sex-specific IMR in 2021 (per 1,000)		Estimated/Expected female IMR		
	1990	2021	Change 1990–2021	Male	Female	1990	2021	Change 1990–2021
World¶	1.13 [1.11; 1.15]	1.17 [1.14; 1.19]	0.04 [0.01; 0.07]†	30.6 [28.8; 32.8]	26.1 [24.6; 28.0]	1.04 [1.03; 1.06]*	1.03 [1.01; 1.05]*	-0.01 [-0.04; 0.01]
South Asia¶§	1.08 [1.05; 1.11]	1.09 [1.04; 1.14]	0.01 [-0.05; 0.06]	32.0 [29.4; 35.0]	29.4 [27.1; 32.1]	1.09 [1.06; 1.11]*	1.13 [1.08; 1.18]*	0.04 [-0.02; 0.10]
Europe and Central Asia	1.24 [1.22; 1.27]	1.25 [1.22; 1.28]	0.00 [-0.03; 0.04]	7.2 [6.8; 8.0]	5.8 [5.5; 6.4]	0.98 [0.96; 1.00]	0.98 [0.95; 1.00]	0.00 [-0.03; 0.03]
Middle East and North Africa¶	1.12 [1.10; 1.14]	1.18 [1.14; 1.23]	0.06 [0.02; 0.12]†	19.6 [16.2; 25.4]	16.5 [13.7; 21.4]	1.08 [1.06; 1.11]*	1.04 [0.99; 1.09]	-0.04 [-0.09; 0.01]
Sub-Saharan Africa	1.17 [1.15; 1.18]	1.22 [1.19; 1.25]	0.05 [0.02; 0.09]†	54.7 [50.6; 62.2]	44.9 [41.3; 51.1]	0.99 [0.98; 1.01]	0.99 [0.96; 1.02]	0.00 [-0.03; 0.03]
Latin America and Caribbean	1.22 [1.18; 1.26]	1.24 [1.19; 1.28]	0.01 [-0.04; 0.06]	15.0 [14.0; 16.4]	12.1 [11.3; 13.3]	0.99 [0.96; 1.03]	1.00 [0.96; 1.03]	0.00 [-0.04; 0.04]
East Asia and Pacific	1.15 [1.10; 1.21]	1.21 [1.16; 1.25]	0.05 [-0.02; 0.12]	12.9 [11.5; 14.2]	10.7 [9.5; 11.8]	1.05 [1.00; 1.11]	1.01 [0.97; 1.05]	-0.04 [-0.11; 0.02]
North America	1.26 [1.24; 1.27]	1.19 [1.15; 1.24]	-0.06 [-0.11; -0.01]†	5.7 [5.4; 6.0]	4.8 [4.5; 5.1]	0.98 [0.97; 0.99]*	1.00 [0.96; 1.04]	0.02 [-0.02; 0.06]
Afghanistan	1.10 [1.03; 1.17]	1.16 [1.06; 1.26]	0.06 [-0.05; 0.18]	46.5 [35.4; 59.8]	40.2 [30.5; 51.6]	1.05 [0.99; 1.12]	1.06 [0.97; 1.15]	0.01 [-0.10; 0.12]
Albania	1.33 [1.23; 1.44]	1.22 [1.14; 1.31]	-0.11 [-0.24; 0.02]	9.2 [8.5; 10.1]	7.6 [6.9; 8.3]	0.92 [0.85; 1.00]	1.00 [0.94; 1.07]	0.08 [-0.02; 0.17]
Algeria	1.18 [1.13; 1.23]	1.18 [1.10; 1.26]	0.00 [-0.10; 0.10]	20.7 [18.2; 23.7]	17.5 [15.4; 20.1]	1.04 [0.99; 1.08]	1.06 [0.98; 1.14]	0.02 [-0.07; 0.11]
Andorra	1.30 [1.16; 1.48]	1.25 [1.12; 1.42]	-0.05 [-0.20; 0.10]	2.9 [0.8; 11.0]	2.3 [0.6; 8.8]	0.94 [0.82; 1.06]	0.96 [0.83; 1.07]	0.02 [-0.10; 0.13]
Angola	1.15 [1.07; 1.25]	1.25 [1.13; 1.37]	0.10 [-0.04; 0.23]	52.2 [25.7; 94.4]	41.7 [20.5; 75.9]	0.99 [0.92; 1.07]	0.97 [0.88; 1.08]	-0.02 [-0.13; 0.10]
Anguilla¶	1.07 [1.00; 1.15]	0.98 [0.89; 1.07]	-0.09 [-0.19; 0.01]	3.9 [1.8; 8.1]	3.9 [1.9; 8.3]	1.15 [1.07; 1.24]*	1.21 [1.11; 1.34]*	0.06 [-0.06; 0.18]
Antigua and Barbuda	1.18 [1.07; 1.31]	1.16 [1.04; 1.30]	-0.02 [-0.16; 0.11]	5.5 [3.6; 8.6]	4.8 [3.1; 7.4]	1.04 [0.95; 1.15]	1.03 [0.92; 1.15]	-0.01 [-0.13; 0.11]
Argentina	1.25 [1.23; 1.27]	1.31 [1.26; 1.36]	0.06 [0.00; 0.11]†	6.9 [6.6; 7.3]	5.3 [5.0; 5.6]	0.99 [0.98; 1.01]	0.92 [0.89; 0.96]*	-0.07 [-0.11; -0.03]†
Armenia	1.24 [1.14; 1.34]	1.23 [1.10; 1.36]	-0.01 [-0.15; 0.12]	10.5 [8.2; 13.2]	8.6 [6.7; 10.8]	0.99 [0.91; 1.08]	1.01 [0.90; 1.12]	0.02 [-0.09; 0.13]
Australia	1.28 [1.24; 1.32]	1.19 [1.13; 1.26]	-0.08 [-0.15; -0.01]†	3.4 [3.2; 3.7]	2.9 [2.7; 3.1]	0.96 [0.93; 0.99]*	0.99 [0.94; 1.05]	0.04 [-0.02; 0.10]
Austria	1.26 [1.21; 1.31]	1.19 [1.11; 1.28]	-0.06 [-0.16; 0.04]	3.2 [2.9; 3.6]	2.7 [2.4; 3.0]	0.97 [0.93; 1.01]	0.99 [0.93; 1.07]	0.02 [-0.05; 0.10]
Azerbaijan	1.17 [1.09; 1.26]	1.22 [1.10; 1.35]	0.05 [-0.08; 0.19]	18.2 [10.8; 30.5]	14.9 [8.8; 25.2]	1.01 [0.94; 1.09]	1.02 [0.92; 1.13]	0.01 [-0.10; 0.12]
Bahamas¶	1.16 [1.09; 1.24]	1.17 [1.06; 1.27]	0.00 [-0.11; 0.12]	12.3 [10.5; 14.3]	10.5 [8.9; 12.3]	1.07 [1.01; 1.14]*	1.06 [0.97; 1.16]	-0.02 [-0.12; 0.10]
Bahrain¶	1.04 [0.99; 1.10]	1.09 [1.01; 1.18]	0.05 [-0.05; 0.15]	6.2 [5.3; 7.3]	5.6 [4.8; 6.7]	1.20 [1.14; 1.26]*	1.09 [1.01; 1.19]*	-0.10 [-0.20; 0.00]
Bangladesh§	1.16 [1.12; 1.21]	1.16 [1.08; 1.24]	-0.01 [-0.09; 0.09]	24.5 [22.2; 27.0]	21.2 [19.1; 23.4]	1.00 [0.96; 1.04]	1.07 [1.00; 1.15]*	0.07 [-0.01; 0.16]
Barbados	1.21 [1.12; 1.30]	1.18 [1.07; 1.30]	-0.02 [-0.15; 0.10]	12.0 [7.9; 18.4]	10.1 [6.6; 15.6]	1.03 [0.96; 1.11]	1.05 [0.95; 1.15]	0.01 [-0.10; 0.13]
Belarus¶	1.37 [1.32; 1.41]	1.26 [1.17; 1.35]	-0.11 [-0.20; -0.01]†	2.3 [1.9; 2.6]	1.8 [1.5; 2.1]	0.91 [0.88; 0.94]*	0.94 [0.88; 1.01]	0.04 [-0.04; 0.11]
Belgium	1.34 [1.29; 1.39]	1.27 [1.19; 1.35]	-0.07 [-0.16; 0.03]	3.8 [3.4; 4.2]	3.0 [2.7; 3.3]	0.92 [0.88; 0.95]*	0.93 [0.88; 1.00]*	0.02 [-0.05; 0.09]
Belize	1.24 [1.14; 1.36]	1.20 [1.11; 1.31]	-0.04 [-0.18; 0.10]	10.5 [8.4; 12.9]	8.7 [7.0; 10.8]	1.00 [0.91; 1.09]	1.02 [0.94; 1.11]	0.02 [-0.09; 0.14]
Benin	1.15 [1.09; 1.20]	1.23 [1.13; 1.33]	0.08 [-0.03; 0.20]	60.7 [50.6; 73.2]	49.4 [41.1; 59.7]	1.01 [0.96; 1.07]	0.98 [0.90; 1.07]	-0.03 [-0.12; 0.07]
Bhutan	1.12 [1.02; 1.22]	1.20 [1.09; 1.34]	0.09 [-0.04; 0.23]	24.5 [14.8; 38.9]	20.3 [12.2; 32.5]	1.05 [0.96; 1.15]	1.03 [0.93; 1.14]	-0.03 [-0.15; 0.10]
Bolivia (Plurinational State of)	1.17 [1.12; 1.23]	1.23 [1.12; 1.36]	0.06 [-0.06; 0.19]	22.2 [15.6; 31.1]	18.1 [12.7; 25.5]	1.00 [0.96; 1.05]	1.01 [0.92; 1.11]	0.01 [-0.09; 0.11]
Bosnia and Herzegovina	1.24 [1.19; 1.29]	1.21 [1.12; 1.32]	-0.03 [-0.13; 0.08]	5.3 [4.1; 6.8]	4.4 [3.4; 5.6]	1.00 [0.96; 1.05]	0.98 [0.91; 1.06]	-0.02 [-0.11; 0.07]
Botswana	1.25 [1.14; 1.37]	1.23 [1.11; 1.36]	-0.02 [-0.17; 0.12]	31.1 [9.8; 89.2]	25.4 [7.9; 72.6]	0.99 [0.90; 1.08]	1.00 [0.90; 1.12]	0.02 [-0.10; 0.14]
Brazil	1.23 [1.14; 1.33]	1.26 [1.13; 1.40]	0.03 [-0.10; 0.17]	14.3 [12.1; 16.9]	11.4 [9.6; 13.4]	0.98 [0.91; 1.06]	0.99 [0.89; 1.10]	0.00 [-0.10; 0.11]
British Virgin Islands	1.26 [1.18; 1.33]	1.18 [1.08; 1.30]	-0.07 [-0.20; 0.06]	10.7 [4.9; 23.5]	9.1 [4.1; 19.7]	0.99 [0.94; 1.05]	1.03 [0.94; 1.14]	0.04 [-0.06; 0.16]
Brunei	1.18 [1.10; 1.27]	1.20 [1.09; 1.31]	0.02 [-0.10; 0.14]	10.5 [8.7; 12.6]	8.7 [7.3; 10.6]	1.04 [0.97; 1.12]	1.03 [0.94; 1.12]	-0.01 [-0.12; 0.09]
Bulgaria	1.29 [1.25; 1.34]	1.18 [1.12; 1.26]	-0.11 [-0.19; -0.03]†	5.7 [5.3; 6.1]	4.8 [4.5; 5.2]	0.96 [0.93; 0.99]*	1.01 [0.95; 1.07]	0.04 [-0.02; 0.11]
Burkina Faso	1.17 [1.11; 1.23]	1.20 [1.10; 1.32]	0.04 [-0.08; 0.16]	56.4 [42.9; 74.6]	46.8 [35.7; 62.1]	1.00 [0.95; 1.05]	1.01 [0.92; 1.10]	0.01 [-0.09; 0.12]
Burundi	1.19 [1.11; 1.28]	1.25 [1.14; 1.37]	0.06 [-0.07; 0.19]	41.7 [27.9; 62.2]	33.4 [22.2; 50.0]	0.97 [0.90; 1.05]	0.98 [0.89; 1.08]	0.01 [-0.10; 0.12]
Cambodia	1.22 [1.16; 1.28]	1.28 [1.16; 1.39]	0.05 [-0.08; 0.18]	23.8 [11.6; 47.7]	18.6 [9.2; 37.3]	0.96 [0.91; 1.01]	0.97 [0.89; 1.07]	0.01 [-0.09; 0.12]
Cameroon	1.21 [1.14; 1.27]	1.24 [1.13; 1.35]	0.03 [-0.09; 0.16]	51.8 [41.8; 64.1]	41.9 [33.6; 51.8]	0.97 [0.92; 1.03]	0.98 [0.90; 1.07]	0.01 [-0.09; 0.11]

¶: Sex ratio is outlying in 1990. §: Sex ratio is outlying in 2021. *: The ratio of estimated to expected female mortality is significantly different from one. †: Change is significantly different from zero.

Continued on next page

Table 13 – continued from previous page

	Sex ratio IMR			Sex-specific IMR in 2021 (per 1,000)		Estimated/Expected female IMR		
	1990	2021	Change 1990–2021	Male	Female	1990	2021	Change 1990–2021
Canada	1.25 [1.21; 1.29]	1.15 [1.10; 1.21]	-0.09 [-0.16; -0.03]†	4.7 [4.5; 5.0]	4.1 [3.9; 4.3]	0.97 [0.94; 1.00]	1.03 [0.98; 1.08]	0.06 [0.00; 0.11]†
Cape Verde	1.19 [1.13; 1.25]	1.21 [1.11; 1.32]	0.02 [-0.09; 0.14]	12.8 [9.7; 16.6]	10.6 [8.0; 13.7]	1.03 [0.98; 1.08]	1.02 [0.94; 1.11]	-0.01 [-0.10; 0.09]
Central African Republic	1.19 [1.12; 1.26]	1.22 [1.12; 1.33]	0.03 [-0.09; 0.15]	82.6 [67.9; 99.4]	67.9 [55.7; 82.1]	0.97 [0.91; 1.03]	0.97 [0.89; 1.06]	0.00 [-0.09; 0.11]
Chad	1.16 [1.10; 1.22]	1.22 [1.11; 1.33]	0.06 [-0.06; 0.18]	72.3 [55.4; 94.4]	59.4 [45.4; 77.6]	1.00 [0.94; 1.06]	0.98 [0.90; 1.08]	-0.02 [-0.12; 0.09]
Chile	1.21 [1.18; 1.24]	1.18 [1.11; 1.27]	-0.03 [-0.11; 0.06]	6.1 [5.1; 7.2]	5.1 [4.3; 6.1]	1.03 [1.00; 1.05]*	1.01 [0.95; 1.08]	-0.01 [-0.08; 0.06]
China¶	1.11 [1.02; 1.20]	1.12 [1.02; 1.23]	0.01 [-0.11; 0.13]	5.3 [4.8; 6.0]	4.8 [4.2; 5.3]	1.10 [1.02; 1.20]*	1.06 [0.97; 1.17]	-0.04 [-0.16; 0.08]
Colombia	1.27 [1.20; 1.35]	1.25 [1.13; 1.37]	-0.03 [-0.16; 0.11]	12.2 [8.0; 18.5]	9.8 [6.4; 14.9]	0.97 [0.92; 1.03]	0.99 [0.90; 1.09]	0.02 [-0.08; 0.12]
Comoros	1.14 [1.04; 1.24]	1.13 [1.02; 1.26]	0.00 [-0.13; 0.12]	41.7 [32.1; 53.9]	36.7 [28.2; 47.9]	1.04 [0.95; 1.13]	1.08 [0.98; 1.21]	0.05 [-0.07; 0.17]
Congo	1.21 [1.12; 1.31]	1.24 [1.12; 1.36]	0.02 [-0.11; 0.16]	35.3 [21.3; 57.3]	28.6 [17.3; 46.4]	0.99 [0.92; 1.08]	1.00 [0.90; 1.10]	0.01 [-0.11; 0.12]
Democratic Republic of the Congo	1.12 [1.05; 1.20]	1.21 [1.10; 1.33]	0.09 [-0.04; 0.22]	68.2 [43.4; 101.8]	56.2 [36.0; 83.8]	1.03 [0.96; 1.10]	0.99 [0.89; 1.09]	-0.04 [-0.15; 0.08]
Cook Islands	1.36 [1.23; 1.50]	1.27 [1.13; 1.42]	-0.09 [-0.24; 0.07]	6.8 [3.7; 12.8]	5.4 [3.0; 10.1]	0.92 [0.83; 1.02]	0.95 [0.85; 1.06]	0.03 [-0.08; 0.14]
Costa Rica	1.28 [1.23; 1.33]	1.12 [1.05; 1.19]	-0.16 [-0.24; -0.08]†	6.6 [6.1; 7.1]	5.9 [5.4; 6.4]	0.97 [0.94; 1.01]	1.07 [1.01; 1.14]*	0.10 [0.03; 0.18]†
Cote d'Ivoire	1.20 [1.14; 1.27]	1.29 [1.18; 1.40]	0.08 [-0.04; 0.21]	62.6 [48.7; 80.6]	48.7 [37.8; 63.3]	0.97 [0.91; 1.02]	0.94 [0.86; 1.03]	-0.03 [-0.12; 0.07]
Croatia	1.31 [1.25; 1.36]	1.20 [1.11; 1.29]	-0.11 [-0.21; 0.00]†	4.3 [3.9; 4.7]	3.6 [3.2; 3.9]	0.95 [0.91; 0.99]*	0.99 [0.92; 1.06]	0.04 [-0.04; 0.12]
Cuba	1.34 [1.30; 1.38]	1.24 [1.17; 1.32]	-0.10 [-0.18; -0.01]†	4.4 [3.6; 5.6]	3.6 [2.9; 4.5]	0.92 [0.89; 0.95]*	0.96 [0.90; 1.02]	0.03 [-0.03; 0.10]
Cyprus	1.22 [1.14; 1.30]	1.17 [1.07; 1.29]	-0.05 [-0.17; 0.08]	2.5 [1.8; 3.5]	2.1 [1.6; 3.0]	1.01 [0.94; 1.08]	1.01 [0.92; 1.11]	0.00 [-0.10; 0.11]
Czech Republic	1.31 [1.24; 1.39]	1.29 [1.20; 1.38]	-0.02 [-0.13; 0.10]	2.5 [2.3; 2.7]	1.9 [1.8; 2.1]	0.94 [0.89; 1.00]*	0.92 [0.86; 0.98]*	-0.03 [-0.10; 0.05]
Denmark	1.29 [1.24; 1.36]	1.19 [1.11; 1.27]	-0.10 [-0.20; -0.01]†	3.4 [3.1; 3.7]	2.8 [2.6; 3.1]	0.94 [0.90; 0.98]*	1.00 [0.93; 1.07]	0.06 [-0.02; 0.14]
Djibouti	1.17 [1.06; 1.28]	1.20 [1.08; 1.33]	0.03 [-0.10; 0.17]	49.8 [28.6; 82.9]	41.5 [23.8; 69.6]	1.01 [0.91; 1.10]	1.01 [0.91; 1.13]	0.01 [-0.11; 0.13]
Dominica	1.15 [1.04; 1.25]	1.16 [1.04; 1.29]	0.01 [-0.11; 0.15]	34.3 [28.9; 40.6]	29.6 [24.9; 35.3]	1.08 [0.99; 1.19]	1.06 [0.96; 1.19]	-0.02 [-0.14; 0.11]
Dominican Republic	1.19 [1.12; 1.25]	1.20 [1.09; 1.31]	0.01 [-0.11; 0.14]	29.6 [22.3; 38.7]	24.8 [18.7; 32.4]	1.03 [0.97; 1.09]	1.04 [0.95; 1.14]	0.01 [-0.10; 0.12]
Ecuador	1.29 [1.19; 1.41]	1.26 [1.15; 1.39]	-0.03 [-0.17; 0.11]	11.9 [10.4; 13.6]	9.4 [8.2; 10.8]	0.95 [0.87; 1.03]	0.98 [0.89; 1.07]	0.03 [-0.07; 0.14]
Egypt¶	1.06 [1.01; 1.10]	1.16 [1.05; 1.27]	0.10 [-0.02; 0.22]	17.4 [11.3; 26.2]	15.0 [9.8; 22.7]	1.14 [1.09; 1.19]*	1.08 [0.98; 1.18]	-0.06 [-0.17; 0.05]
El Salvador	1.20 [1.10; 1.31]	1.22 [1.09; 1.35]	0.02 [-0.11; 0.16]	11.7 [6.9; 19.8]	9.6 [5.7; 16.3]	1.02 [0.93; 1.11]	1.01 [0.91; 1.13]	-0.01 [-0.12; 0.11]
Equatorial Guinea	1.16 [1.04; 1.30]	1.22 [1.09; 1.36]	0.06 [-0.08; 0.20]	62.6 [37.0; 104.7]	51.4 [30.3; 86.0]	0.99 [0.88; 1.11]	0.99 [0.88; 1.11]	0.00 [-0.13; 0.12]
Eritrea¶	1.32 [1.24; 1.40]	1.32 [1.20; 1.46]	0.00 [-0.14; 0.15]	32.8 [19.0; 53.3]	24.8 [14.2; 40.4]	0.88 [0.83; 0.94]*	0.93 [0.85; 1.03]	0.05 [-0.05; 0.16]
Estonia	1.31 [1.24; 1.38]	1.22 [1.11; 1.34]	-0.09 [-0.21; 0.04]	1.7 [1.4; 2.1]	1.4 [1.1; 1.7]	0.95 [0.90; 1.00]	0.97 [0.88; 1.07]	0.02 [-0.08; 0.12]
Ethiopia¶	1.22 [1.16; 1.29]	1.34 [1.22; 1.47]	0.12 [-0.01; 0.26]	39.1 [31.7; 48.2]	29.1 [23.5; 36.1]	0.94 [0.89; 0.99]*	0.92 [0.84; 1.01]	-0.02 [-0.11; 0.08]
Federated States of Micronesia	1.32 [1.19; 1.47]	1.32 [1.19; 1.48]	0.00 [-0.15; 0.16]	23.8 [10.9; 48.4]	18.0 [8.2; 36.3]	0.93 [0.83; 1.03]	0.94 [0.83; 1.04]	0.01 [-0.10; 0.12]
Fiji	1.16 [1.07; 1.25]	1.18 [1.10; 1.28]	0.03 [-0.09; 0.15]	25.2 [22.6; 27.9]	21.3 [19.0; 23.7]	1.08 [1.00; 1.16]	1.05 [0.98; 1.13]	-0.03 [-0.14; 0.09]
Finland	1.22 [1.17; 1.28]	1.18 [1.10; 1.28]	-0.04 [-0.14; 0.06]	1.9 [1.7; 2.1]	1.6 [1.5; 1.8]	0.98 [0.93; 1.02]	1.00 [0.93; 1.08]	0.02 [-0.06; 0.11]
France	1.35 [1.32; 1.39]	1.23 [1.16; 1.29]	-0.13 [-0.20; -0.06]†	3.8 [3.5; 4.1]	3.1 [2.8; 3.4]	0.90 [0.88; 0.92]*	0.97 [0.92; 1.02]	0.07 [0.01; 0.12]†
Gabon	1.27 [1.16; 1.39]	1.27 [1.15; 1.42]	0.00 [-0.14; 0.15]	32.8 [20.4; 50.4]	25.7 [16.0; 39.5]	0.94 [0.86; 1.03]	0.97 [0.87; 1.08]	0.03 [-0.08; 0.14]
The Gambia	1.23 [1.13; 1.35]	1.29 [1.17; 1.41]	0.05 [-0.09; 0.19]	38.1 [31.6; 45.5]	29.6 [24.5; 35.6]	0.96 [0.87; 1.05]	0.96 [0.87; 1.06]	0.00 [-0.11; 0.11]
Georgia	1.29 [1.17; 1.42]	1.26 [1.13; 1.40]	-0.03 [-0.17; 0.12]	9.4 [8.3; 10.6]	7.4 [6.5; 8.5]	0.95 [0.86; 1.04]	0.97 [0.87; 1.08]	0.02 [-0.09; 0.13]
Germany	1.31 [1.28; 1.34]	1.17 [1.13; 1.21]	-0.14 [-0.19; -0.09]†	3.3 [3.1; 3.4]	2.8 [2.7; 2.9]	0.92 [0.90; 0.95]*	1.01 [0.98; 1.05]	0.09 [0.05; 0.13]†
Ghana	1.19 [1.13; 1.26]	1.25 [1.16; 1.36]	0.06 [-0.05; 0.19]	36.1 [28.6; 45.7]	28.8 [22.7; 36.4]	0.99 [0.94; 1.05]	0.98 [0.90; 1.07]	-0.01 [-0.10; 0.09]
Greece	1.17 [1.13; 1.22]	1.18 [1.10; 1.26]	0.00 [-0.08; 0.09]	3.5 [3.1; 4.0]	3.0 [2.6; 3.4]	1.04 [1.01; 1.08]*	1.00 [0.94; 1.07]	-0.04 [-0.11; 0.04]
Grenada¶	1.13 [1.04; 1.23]	1.16 [1.05; 1.28]	0.03 [-0.09; 0.16]	15.5 [12.7; 19.0]	13.3 [10.8; 16.3]	1.10 [1.01; 1.20]*	1.06 [0.97; 1.18]	-0.04 [-0.15; 0.08]
Guatemala	1.24 [1.18; 1.31]	1.25 [1.15; 1.37]	0.01 [-0.11; 0.13]	21.6 [15.7; 29.2]	17.3 [12.5; 23.4]	0.97 [0.92; 1.02]	1.00 [0.91; 1.09]	0.03 [-0.07; 0.13]
Guinea	1.14 [1.09; 1.20]	1.25 [1.15; 1.37]	0.11 [-0.01; 0.23]	70.8 [59.1; 86.0]	56.5 [47.0; 68.9]	1.00 [0.95; 1.05]	0.96 [0.87; 1.04]	-0.04 [-0.14; 0.06]
Guinea-Bissau	1.16 [1.08; 1.25]	1.25 [1.13; 1.37]	0.09 [-0.05; 0.22]	55.3 [35.3; 85.5]	44.2 [28.3; 68.7]	0.98 [0.91; 1.06]	0.97 [0.88; 1.07]	-0.01 [-0.12; 0.10]
Guyana¶	1.31 [1.22; 1.41]	1.29 [1.16; 1.43]	-0.02 [-0.16; 0.12]	26.0 [15.4; 42.9]	20.2 [12.0; 33.4]	0.93 [0.86; 1.00]*	0.96 [0.87; 1.06]	0.04 [-0.07; 0.15]
Haiti	1.19 [1.12; 1.26]	1.23 [1.12; 1.35]	0.05 [-0.08; 0.18]	50.1 [36.5; 68.8]	40.6 [29.5; 55.8]	0.98 [0.92; 1.04]	0.99 [0.90; 1.09]	0.01 [-0.10; 0.12]
Honduras	1.25 [1.17; 1.34]	1.26 [1.15; 1.38]	0.00 [-0.12; 0.14]	15.8 [12.0; 21.2]	12.6 [9.5; 16.8]	0.97 [0.91; 1.05]	0.99 [0.90; 1.08]	0.01 [-0.09; 0.12]
Hungary	1.25 [1.21; 1.29]	1.20 [1.13; 1.28]	-0.04 [-0.12; 0.05]	3.6 [3.2; 4.1]	3.0 [2.6; 3.4]	1.00 [0.97; 1.03]	0.98 [0.92; 1.05]	-0.01 [-0.08; 0.06]

¶: Sex ratio is outlying in 1990. §: Sex ratio is outlying in 2021. *: The ratio of estimated to expected female mortality is significantly different from one. †: Change is significantly different from zero.

Continued on next page

Table 13 – continued from previous page

	Sex ratio IMR			Sex-specific IMR in 2021 (per 1,000)		Estimated/Expected female IMR		
	1990	2021	Change 1990–2021	Male	Female	1990	2021	Change 1990–2021
Iceland	1.19 [1.08; 1.30]	1.19 [1.07; 1.32]	0.00 [-0.13; 0.14]	2.3 [1.7; 2.9]	1.9 [1.5; 2.5]	1.00 [0.92; 1.10]	1.00 [0.90; 1.11]	0.00 [-0.12; 0.11]
India¶§	1.06 [1.02; 1.09]	1.01 [0.95; 1.09]	-0.04 [-0.12; 0.04]	25.7 [23.2; 28.3]	25.3 [22.9; 27.9]	1.12 [1.08; 1.15]*	1.23 [1.14; 1.31]*	0.11 [0.02; 0.20]†
Indonesia	1.23 [1.18; 1.29]	1.24 [1.14; 1.35]	0.01 [-0.11; 0.13]	20.9 [16.5; 26.4]	16.8 [13.3; 21.3]	0.97 [0.93; 1.02]	1.00 [0.92; 1.09]	0.03 [-0.07; 0.13]
Iran (Islamic Republic of)¶	1.06 [1.00; 1.12]	1.12 [1.01; 1.23]	0.06 [-0.05; 0.18]	11.5 [6.4; 20.0]	10.3 [5.7; 17.9]	1.16 [1.09; 1.23]*	1.10 [1.00; 1.22]	-0.06 [-0.18; 0.06]
Iraq	1.18 [1.11; 1.25]	1.22 [1.11; 1.34]	0.04 [-0.08; 0.17]	22.7 [16.8; 30.0]	18.7 [13.8; 24.6]	1.04 [0.97; 1.11]	1.02 [0.93; 1.12]	-0.02 [-0.13; 0.09]
Ireland	1.25 [1.19; 1.31]	1.18 [1.10; 1.28]	-0.06 [-0.17; 0.04]	3.0 [2.5; 3.4]	2.5 [2.2; 2.9]	0.98 [0.93; 1.02]	1.00 [0.93; 1.08]	0.02 [-0.06; 0.11]
Israel	1.14 [1.10; 1.18]	1.19 [1.12; 1.26]	0.05 [-0.03; 0.13]	2.9 [2.8; 3.1]	2.5 [2.3; 2.6]	1.08 [1.04; 1.12]*	1.00 [0.94; 1.05]	-0.08 [-0.15; -0.01]†
Italy	1.23 [1.20; 1.26]	1.17 [1.11; 1.23]	-0.06 [-0.12; 0.01]	2.4 [2.2; 2.6]	2.0 [1.9; 2.2]	0.99 [0.97; 1.02]	1.01 [0.96; 1.06]	0.02 [-0.04; 0.08]
Jamaica	1.34 [1.22; 1.48]	1.30 [1.17; 1.45]	-0.04 [-0.19; 0.11]	12.1 [6.1; 23.1]	9.3 [4.7; 17.9]	0.93 [0.84; 1.02]	0.95 [0.85; 1.05]	0.02 [-0.09; 0.13]
Japan	1.17 [1.14; 1.20]	1.10 [1.06; 1.15]	-0.06 [-0.12; -0.01]†	1.8 [1.8; 1.9]	1.6 [1.6; 1.7]	1.02 [0.99; 1.04]	1.07 [1.03; 1.12]*	0.06 [0.01; 0.11]†
Jordan¶	1.14 [1.07; 1.22]	1.24 [1.12; 1.36]	0.09 [-0.03; 0.22]	13.8 [9.6; 19.9]	11.2 [7.8; 16.1]	1.09 [1.02; 1.16]*	1.00 [0.91; 1.11]	-0.08 [-0.19; 0.03]
Kazakhstan¶	1.32 [1.23; 1.43]	1.30 [1.18; 1.45]	-0.02 [-0.16; 0.13]	10.3 [9.7; 10.9]	7.9 [7.4; 8.5]	0.92 [0.85; 1.00]*	0.94 [0.85; 1.04]	0.02 [-0.08; 0.13]
Kenya	1.17 [1.11; 1.23]	1.22 [1.13; 1.33]	0.05 [-0.06; 0.17]	30.7 [25.5; 36.8]	25.1 [20.7; 30.1]	1.02 [0.97; 1.08]	1.01 [0.93; 1.10]	-0.01 [-0.11; 0.09]
Kiribati	1.20 [1.10; 1.30]	1.23 [1.11; 1.36]	0.03 [-0.10; 0.17]	42.1 [23.7; 72.0]	34.1 [19.3; 58.5]	1.00 [0.91; 1.09]	1.00 [0.89; 1.11]	0.00 [-0.11; 0.12]
Democratic People's Republic of Korea	1.24 [1.10; 1.39]	1.23 [1.09; 1.38]	-0.01 [-0.15; 0.14]	11.2 [8.7; 14.3]	9.1 [7.1; 11.7]	1.00 [0.89; 1.13]	1.00 [0.89; 1.12]	0.00 [-0.12; 0.12]
Republic of Korea	1.18 [1.09; 1.27]	1.21 [1.15; 1.27]	0.03 [-0.08; 0.13]	2.7 [2.6; 2.8]	2.2 [2.1; 2.4]	1.05 [0.98; 1.14]	0.98 [0.93; 1.03]	-0.07 [-0.17; 0.02]
Kosovo	1.15 [1.05; 1.27]	1.18 [1.07; 1.29]	0.03 [-0.11; 0.15]	9.8 [8.5; 11.2]	8.3 [7.2; 9.6]	1.02 [0.92; 1.13]	1.04 [0.95; 1.14]	0.02 [-0.09; 0.14]
Kuwait	1.16 [1.11; 1.21]	1.21 [1.13; 1.29]	0.05 [-0.04; 0.14]	8.2 [7.3; 9.1]	6.7 [6.0; 7.5]	1.06 [1.01; 1.11]*	1.00 [0.94; 1.07]	-0.06 [-0.13; 0.03]
Kyrgyzstan	1.22 [1.13; 1.33]	1.25 [1.13; 1.39]	0.03 [-0.11; 0.17]	17.3 [16.3; 18.4]	13.8 [12.8; 14.8]	0.99 [0.91; 1.08]	0.99 [0.90; 1.10]	0.00 [-0.11; 0.12]
Lao People's Democratic Republic	1.19 [1.11; 1.27]	1.27 [1.15; 1.39]	0.08 [-0.05; 0.22]	38.1 [27.1; 52.2]	30.0 [21.4; 41.3]	0.98 [0.91; 1.05]	0.97 [0.88; 1.07]	0.00 [-0.11; 0.10]
Latvia	1.29 [1.23; 1.35]	1.16 [1.07; 1.26]	-0.13 [-0.24; -0.01]†	3.4 [2.9; 4.0]	2.9 [2.5; 3.4]	0.96 [0.92; 1.01]	1.02 [0.94; 1.11]	0.06 [-0.03; 0.15]
Lebanon¶	1.07 [0.99; 1.16]	1.11 [0.99; 1.23]	0.04 [-0.08; 0.16]	7.4 [6.0; 9.2]	6.7 [5.4; 8.3]	1.16 [1.07; 1.25]*	1.08 [0.98; 1.22]	-0.07 [-0.19; 0.05]
Lesotho	1.21 [1.14; 1.29]	1.23 [1.12; 1.35]	0.02 [-0.11; 0.14]	62.6 [44.9; 89.1]	50.9 [36.3; 72.2]	0.98 [0.92; 1.05]	0.98 [0.89; 1.08]	0.00 [-0.11; 0.11]
Liberia	1.17 [1.11; 1.24]	1.21 [1.11; 1.32]	0.04 [-0.07; 0.17]	62.1 [47.6; 81.8]	51.1 [39.1; 67.7]	0.97 [0.92; 1.03]	0.99 [0.91; 1.09]	0.02 [-0.08; 0.12]
Libya	1.22 [1.13; 1.31]	1.23 [1.12; 1.37]	0.02 [-0.11; 0.16]	10.2 [5.8; 17.9]	8.2 [4.6; 14.5]	1.01 [0.94; 1.09]	0.99 [0.89; 1.10]	-0.02 [-0.13; 0.09]
Lithuania	1.25 [1.20; 1.30]	1.19 [1.10; 1.28]	-0.06 [-0.16; 0.05]	2.9 [2.5; 3.4]	2.5 [2.1; 2.9]	0.99 [0.95; 1.03]	1.00 [0.92; 1.08]	0.01 [-0.08; 0.09]
Luxembourg	1.23 [1.13; 1.34]	1.20 [1.08; 1.33]	-0.03 [-0.17; 0.10]	2.4 [1.9; 3.2]	2.0 [1.5; 2.7]	0.99 [0.91; 1.07]	0.99 [0.89; 1.10]	0.00 [-0.10; 0.12]
Macedonia¶	1.13 [1.06; 1.20]	1.10 [1.03; 1.19]	-0.03 [-0.12; 0.08]	4.9 [3.9; 6.2]	4.4 [3.5; 5.6]	1.09 [1.03; 1.16]*	1.08 [1.00; 1.16]*	-0.02 [-0.12; 0.08]
Madagascar	1.15 [1.09; 1.21]	1.23 [1.13; 1.34]	0.08 [-0.03; 0.20]	49.9 [43.1; 58.6]	40.5 [35.0; 47.7]	1.02 [0.97; 1.07]	0.99 [0.90; 1.08]	-0.03 [-0.12; 0.07]
Malawi	1.11 [1.07; 1.16]	1.28 [1.17; 1.39]	0.16 [0.05; 0.28]†	34.9 [23.3; 50.5]	27.3 [18.1; 39.6]	1.02 [0.98; 1.07]	0.97 [0.89; 1.05]	-0.06 [-0.15; 0.04]
Malaysia	1.24 [1.12; 1.38]	1.20 [1.09; 1.31]	-0.05 [-0.19; 0.10]	7.0 [6.3; 7.8]	5.9 [5.2; 6.6]	1.00 [0.90; 1.12]	1.01 [0.92; 1.11]	0.01 [-0.11; 0.12]
Maldives	1.21 [1.11; 1.32]	1.19 [1.08; 1.30]	-0.02 [-0.15; 0.11]	5.5 [4.6; 6.6]	4.7 [3.9; 5.6]	0.99 [0.91; 1.09]	1.01 [0.92; 1.10]	0.01 [-0.09; 0.12]
Mali	1.17 [1.12; 1.21]	1.18 [1.09; 1.29]	0.02 [-0.08; 0.12]	66.7 [56.5; 79.0]	56.3 [47.6; 66.9]	0.98 [0.95; 1.03]	1.02 [0.93; 1.10]	0.03 [-0.06; 0.13]
Malta	1.22 [1.12; 1.32]	1.15 [1.04; 1.27]	-0.06 [-0.19; 0.08]	5.4 [4.5; 6.5]	4.7 [3.9; 5.7]	1.01 [0.93; 1.10]	1.03 [0.94; 1.14]	0.02 [-0.09; 0.13]
Marshall Islands	1.25 [1.12; 1.39]	1.28 [1.15; 1.43]	0.04 [-0.11; 0.18]	27.7 [16.8; 45.2]	21.6 [13.2; 35.3]	0.98 [0.88; 1.10]	0.97 [0.86; 1.08]	-0.02 [-0.13; 0.10]
Mauritania	1.23 [1.15; 1.32]	1.24 [1.14; 1.35]	0.01 [-0.11; 0.14]	35.6 [28.8; 43.9]	28.7 [23.0; 35.5]	0.97 [0.90; 1.04]	0.99 [0.91; 1.08]	0.03 [-0.07; 0.13]
Mauritius¶	1.33 [1.27; 1.39]	1.24 [1.15; 1.33]	-0.09 [-0.20; 0.02]	16.9 [14.1; 20.2]	13.6 [11.4; 16.3]	0.94 [0.90; 0.98]*	1.00 [0.94; 1.08]	0.06 [-0.02; 0.15]
Mexico	1.22 [1.13; 1.31]	1.22 [1.15; 1.29]	0.00 [-0.11; 0.12]	12.5 [10.6; 14.6]	10.2 [8.7; 12.0]	1.01 [0.94; 1.10]	1.01 [0.96; 1.07]	0.00 [-0.10; 0.10]
Republic of Moldova	1.28 [1.16; 1.42]	1.25 [1.12; 1.40]	-0.03 [-0.17; 0.12]	13.5 [9.5; 19.4]	10.8 [7.6; 15.4]	0.97 [0.88; 1.07]	0.99 [0.88; 1.10]	0.02 [-0.09; 0.13]
Monaco	1.24 [1.16; 1.33]	1.23 [1.12; 1.34]	-0.01 [-0.14; 0.12]	1.1 [0.7; 1.7]	0.9 [0.5; 1.4]	0.95 [0.89; 1.02]	0.96 [0.88; 1.05]	0.01 [-0.09; 0.11]
Mongolia¶	1.40 [1.30; 1.49]	1.26 [1.19; 1.33]	-0.14 [-0.25; -0.02]†	14.1 [13.0; 15.4]	11.2 [10.2; 12.2]	0.84 [0.79; 0.91]*	0.98 [0.93; 1.04]	0.14 [0.06; 0.22]†
Montenegro¶	1.12 [1.05; 1.20]	1.13 [1.03; 1.24]	0.01 [-0.11; 0.13]	2.0 [1.6; 2.5]	1.8 [1.5; 2.2]	1.11 [1.04; 1.18]*	1.05 [0.96; 1.15]	-0.06 [-0.17; 0.05]
Montserrat¶	0.90 [0.83; 0.97]	0.96 [0.83; 1.09]	0.06 [-0.05; 0.18]	4.4 [1.6; 12.0]	4.6 [1.7; 12.7]	1.38 [1.28; 1.50]*	1.26 [1.09; 1.45]*	-0.13 [-0.27; 0.04]
Morocco	1.18 [1.11; 1.25]	1.23 [1.12; 1.36]	0.06 [-0.07; 0.19]	17.0 [11.7; 24.1]	13.8 [9.5; 19.4]	1.02 [0.96; 1.08]	1.01 [0.91; 1.11]	-0.01 [-0.11; 0.10]
Mozambique	1.10 [1.04; 1.15]	1.17 [1.07; 1.28]	0.07 [-0.04; 0.19]	54.8 [37.4; 81.3]	47.0 [31.9; 69.6]	1.04 [0.99; 1.09]	1.04 [0.95; 1.14]	0.00 [-0.11; 0.11]
Myanmar	1.23 [1.13; 1.33]	1.24 [1.13; 1.37]	0.02 [-0.12; 0.16]	37.3 [24.8; 54.1]	30.0 [19.9; 43.4]	0.96 [0.89; 1.04]	0.99 [0.90; 1.09]	0.03 [-0.08; 0.14]

¶: Sex ratio is outlying in 1990. §: Sex ratio is outlying in 2021. *: The ratio of estimated to expected female mortality is significantly different from one. †: Change is significantly different from zero.

Continued on next page

Table 13 – continued from previous page

	Sex ratio IMR			Sex-specific IMR in 2021 (per 1,000)		Estimated/Expected female IMR		
	1990	2021	Change 1990–2021	Male	Female	1990	2021	Change 1990–2021
Namibia	1.19 [1.11; 1.28]	1.23 [1.11; 1.36]	0.04 [-0.09; 0.18]	32.3 [18.0; 55.6]	26.3 [14.7; 45.2]	1.02 [0.95; 1.10]	1.00 [0.91; 1.11]	-0.02 [-0.13; 0.10]
Nauru	1.21 [1.08; 1.37]	1.24 [1.11; 1.40]	0.03 [-0.11; 0.18]	25.6 [13.3; 46.2]	20.5 [10.7; 37.2]	1.00 [0.89; 1.13]	1.00 [0.89; 1.12]	0.00 [-0.13; 0.12]
Nepal	1.13 [1.07; 1.19]	1.21 [1.10; 1.31]	0.08 [-0.04; 0.20]	24.9 [19.4; 32.1]	20.6 [16.1; 26.5]	1.04 [0.98; 1.09]	1.03 [0.95; 1.13]	0.00 [-0.10; 0.11]
Netherlands	1.31 [1.27; 1.36]	1.20 [1.14; 1.27]	-0.11 [-0.19; -0.03]†	3.8 [3.6; 4.0]	3.2 [3.0; 3.3]	0.92 [0.89; 0.96]*	0.99 [0.94; 1.04]	0.06 [0.00; 0.12]†
New Zealand	1.27 [1.22; 1.33]	1.19 [1.10; 1.28]	-0.09 [-0.19; 0.02]	4.3 [3.9; 4.7]	3.6 [3.2; 4.0]	0.96 [0.92; 1.01]	1.00 [0.92; 1.08]	0.03 [-0.05; 0.12]
Nicaragua	1.23 [1.16; 1.30]	1.26 [1.14; 1.39]	0.02 [-0.10; 0.17]	12.7 [10.9; 14.6]	10.1 [8.6; 11.7]	0.98 [0.93; 1.05]	0.98 [0.89; 1.08]	0.00 [-0.10; 0.10]
Niger	1.11 [1.06; 1.16]	1.17 [1.07; 1.26]	0.06 [-0.05; 0.17]	64.0 [55.0; 75.2]	54.8 [47.0; 64.6]	1.03 [0.99; 1.08]	1.03 [0.95; 1.12]	0.00 [-0.10; 0.10]
Nigeria	1.18 [1.13; 1.24]	1.20 [1.11; 1.29]	0.01 [-0.09; 0.12]	76.7 [58.7; 102.0]	64.1 [49.0; 85.6]	0.97 [0.92; 1.02]	0.99 [0.92; 1.08]	0.03 [-0.06; 0.12]
Niue	1.24 [1.11; 1.40]	1.25 [1.11; 1.40]	0.00 [-0.15; 0.15]	22.6 [9.8; 49.5]	18.2 [7.8; 39.9]	1.00 [0.89; 1.12]	0.99 [0.88; 1.12]	-0.01 [-0.13; 0.11]
Norway	1.27 [1.21; 1.33]	1.22 [1.13; 1.31]	-0.06 [-0.16; 0.05]	1.9 [1.7; 2.2]	1.6 [1.4; 1.8]	0.95 [0.91; 1.00]*	0.98 [0.91; 1.05]	0.02 [-0.06; 0.11]
Oman	1.22 [1.11; 1.34]	1.22 [1.09; 1.36]	0.00 [-0.14; 0.14]	9.5 [8.5; 10.7]	7.8 [6.9; 8.8]	1.01 [0.92; 1.12]	1.00 [0.90; 1.12]	-0.01 [-0.12; 0.10]
Pakistan	1.13 [1.08; 1.18]	1.20 [1.12; 1.29]	0.08 [-0.03; 0.18]	57.5 [46.8; 70.3]	47.8 [38.9; 58.3]	1.03 [0.98; 1.08]	1.01 [0.93; 1.09]	-0.02 [-0.11; 0.07]
Palau	1.25 [1.10; 1.40]	1.25 [1.11; 1.40]	0.00 [-0.15; 0.15]	16.7 [8.0; 35.5]	13.4 [6.4; 28.7]	0.99 [0.88; 1.12]	0.99 [0.88; 1.12]	0.00 [-0.12; 0.12]
Panama	1.24 [1.11; 1.37]	1.23 [1.10; 1.38]	-0.01 [-0.15; 0.15]	13.1 [6.6; 26.0]	10.7 [5.3; 21.1]	1.00 [0.90; 1.12]	1.00 [0.89; 1.12]	0.00 [-0.12; 0.12]
Papua New Guinea	1.13 [1.04; 1.24]	1.19 [1.07; 1.32]	0.06 [-0.08; 0.19]	37.3 [26.0; 52.4]	31.4 [21.9; 44.4]	1.06 [0.97; 1.16]	1.04 [0.93; 1.15]	-0.02 [-0.14; 0.10]
Paraguay	1.20 [1.09; 1.32]	1.22 [1.10; 1.37]	0.02 [-0.11; 0.17]	17.1 [8.3; 34.5]	14.0 [6.7; 28.1]	1.03 [0.93; 1.13]	1.01 [0.90; 1.13]	-0.02 [-0.13; 0.10]
Peru	1.17 [1.13; 1.22]	1.21 [1.11; 1.33]	0.04 [-0.07; 0.16]	12.0 [9.5; 15.4]	9.9 [7.8; 12.7]	1.03 [0.99; 1.07]	1.02 [0.93; 1.11]	-0.01 [-0.10; 0.09]
Philippines¶	1.30 [1.23; 1.38]	1.24 [1.13; 1.36]	-0.06 [-0.18; 0.07]	22.6 [15.9; 30.9]	18.2 [12.7; 24.9]	0.94 [0.89; 1.00]*	1.00 [0.91; 1.10]	0.06 [-0.04; 0.17]
Poland	1.28 [1.26; 1.31]	1.19 [1.14; 1.24]	-0.09 [-0.15; -0.03]†	4.0 [3.9; 4.2]	3.4 [3.3; 3.5]	0.97 [0.95; 0.99]*	1.00 [0.95; 1.04]	0.03 [-0.02; 0.08]
Portugal	1.25 [1.21; 1.29]	1.20 [1.12; 1.28]	-0.05 [-0.14; 0.04]	2.8 [2.5; 3.0]	2.3 [2.1; 2.5]	0.99 [0.96; 1.02]	0.99 [0.93; 1.06]	0.00 [-0.07; 0.07]
Qatar	1.18 [1.10; 1.28]	1.13 [1.05; 1.22]	-0.05 [-0.17; 0.07]	4.8 [4.3; 5.4]	4.2 [3.7; 4.8]	1.06 [0.98; 1.14]	1.05 [0.97; 1.13]	-0.01 [-0.11; 0.10]
Romania	1.26 [1.23; 1.29]	1.16 [1.11; 1.22]	-0.10 [-0.16; -0.04]†	5.6 [5.4; 5.9]	4.9 [4.6; 5.1]	0.99 [0.97; 1.01]	1.02 [0.98; 1.07]	0.04 [-0.01; 0.09]
Russian Federation¶	1.36 [1.34; 1.38]	1.23 [1.19; 1.28]	-0.13 [-0.18; -0.08]†	4.5 [4.2; 4.7]	3.6 [3.4; 3.8]	0.92 [0.90; 0.93]*	0.96 [0.93; 1.00]*	0.05 [0.01; 0.08]†
Rwanda	1.15 [1.10; 1.21]	1.22 [1.11; 1.33]	0.06 [-0.05; 0.18]	32.5 [20.7; 49.0]	26.7 [17.0; 40.1]	1.02 [0.96; 1.07]	1.02 [0.93; 1.11]	0.00 [-0.10; 0.11]
Saint Kitts and Nevis	1.23 [1.12; 1.36]	1.22 [1.10; 1.36]	-0.01 [-0.15; 0.13]	13.5 [9.0; 20.7]	11.0 [7.2; 16.8]	1.01 [0.92; 1.11]	1.01 [0.91; 1.12]	0.00 [-0.11; 0.12]
Saint Lucia	1.22 [1.14; 1.31]	1.21 [1.10; 1.33]	-0.01 [-0.14; 0.12]	24.5 [20.8; 28.8]	20.3 [17.2; 23.9]	1.02 [0.95; 1.10]	1.03 [0.93; 1.13]	0.01 [-0.10; 0.12]
Samoa	1.21 [1.11; 1.33]	1.23 [1.12; 1.35]	0.02 [-0.12; 0.16]	15.8 [11.0; 22.4]	12.8 [8.9; 18.2]	1.02 [0.93; 1.12]	1.01 [0.92; 1.11]	-0.02 [-0.13; 0.10]
San Marino	1.23 [1.10; 1.39]	1.18 [1.05; 1.33]	-0.06 [-0.20; 0.09]	1.6 [0.8; 3.4]	1.4 [0.7; 2.9]	1.00 [0.89; 1.13]	1.00 [0.89; 1.13]	0.00 [-0.12; 0.12]
Sao Tome and Principe	1.22 [1.12; 1.34]	1.26 [1.14; 1.40]	0.04 [-0.10; 0.18]	13.5 [7.6; 23.8]	10.7 [6.0; 18.9]	0.97 [0.89; 1.07]	0.98 [0.88; 1.09]	0.01 [-0.11; 0.12]
Saudi Arabia	1.13 [1.02; 1.24]	1.05 [0.95; 1.16]	-0.08 [-0.20; 0.05]	5.9 [4.4; 8.1]	5.6 [4.2; 7.7]	1.09 [1.00; 1.21]	1.13 [1.03; 1.26]*	0.04 [-0.09; 0.17]
Senegal	1.18 [1.13; 1.23]	1.28 [1.18; 1.39]	0.10 [-0.01; 0.22]	32.6 [26.3; 39.9]	25.5 [20.5; 31.3]	1.01 [0.97; 1.06]	0.97 [0.89; 1.05]	-0.04 [-0.13; 0.05]
Serbia¶	1.13 [1.10; 1.16]	1.23 [1.15; 1.30]	0.09 [0.01; 0.18]†	5.2 [4.8; 5.6]	4.3 [3.9; 4.6]	1.10 [1.07; 1.13]*	0.97 [0.91; 1.03]	-0.13 [-0.20; -0.06]†
Seychelles	1.17 [1.06; 1.27]	1.17 [1.05; 1.29]	0.01 [-0.13; 0.14]	12.9 [9.9; 16.9]	11.0 [8.4; 14.5]	1.06 [0.98; 1.17]	1.05 [0.96; 1.17]	-0.01 [-0.13; 0.11]
Sierra Leone	1.15 [1.08; 1.22]	1.18 [1.09; 1.28]	0.03 [-0.08; 0.15]	84.4 [71.6; 98.9]	71.7 [60.4; 83.9]	0.99 [0.93; 1.05]	1.00 [0.93; 1.09]	0.01 [-0.08; 0.11]
Singapore	1.18 [1.12; 1.24]	1.18 [1.09; 1.28]	0.00 [-0.11; 0.11]	1.9 [1.5; 2.3]	1.6 [1.3; 1.9]	1.02 [0.97; 1.07]	1.00 [0.92; 1.09]	-0.01 [-0.11; 0.08]
Slovakia	1.31 [1.26; 1.35]	1.21 [1.13; 1.28]	-0.10 [-0.19; -0.01]†	5.1 [4.8; 5.4]	4.2 [3.9; 4.5]	0.95 [0.92; 0.99]*	0.99 [0.93; 1.05]	0.04 [-0.03; 0.11]
Slovenia	1.26 [1.19; 1.33]	1.18 [1.08; 1.29]	-0.08 [-0.20; 0.04]	1.9 [1.6; 2.2]	1.6 [1.4; 1.9]	0.97 [0.92; 1.03]	1.00 [0.92; 1.10]	0.03 [-0.07; 0.14]
Solomon Islands	1.18 [1.06; 1.32]	1.20 [1.08; 1.34]	0.02 [-0.12; 0.15]	17.5 [10.7; 27.0]	14.6 [8.9; 22.5]	1.04 [0.94; 1.16]	1.03 [0.93; 1.15]	-0.01 [-0.13; 0.11]
Somalia	1.14 [1.03; 1.25]	1.17 [1.06; 1.30]	0.04 [-0.09; 0.17]	76.4 [39.4; 157.0]	65.1 [33.5; 135.8]	1.02 [0.93; 1.12]	1.01 [0.90; 1.13]	-0.01 [-0.13; 0.12]
South Africa§	1.20 [1.12; 1.30]	1.17 [1.10; 1.24]	-0.04 [-0.15; 0.07]	28.4 [26.1; 31.0]	24.4 [22.3; 26.6]	1.01 [0.94; 1.09]	1.06 [1.00; 1.13]*	0.05 [-0.04; 0.15]
South Sudan	1.12 [1.00; 1.25]	1.18 [1.05; 1.32]	0.06 [-0.07; 0.20]	68.9 [28.8; 145.8]	58.5 [24.7; 124.0]	1.02 [0.92; 1.14]	1.01 [0.90; 1.14]	-0.01 [-0.13; 0.12]
Spain	1.23 [1.19; 1.26]	1.20 [1.14; 1.27]	-0.03 [-0.10; 0.05]	2.8 [2.6; 3.0]	2.3 [2.2; 2.5]	0.99 [0.96; 1.02]	0.99 [0.93; 1.04]	0.00 [-0.06; 0.05]
Sri Lanka	1.23 [1.20; 1.26]	1.23 [1.14; 1.31]	-0.01 [-0.09; 0.08]	6.3 [4.7; 8.5]	5.2 [3.8; 6.9]	1.01 [0.99; 1.04]	0.98 [0.92; 1.05]	-0.03 [-0.10; 0.04]
Saint Vincent and the Grenadines	1.16 [1.08; 1.25]	1.20 [1.08; 1.33]	0.04 [-0.09; 0.17]	13.7 [10.0; 18.6]	11.4 [8.3; 15.7]	1.07 [1.00; 1.16]	1.03 [0.93; 1.14]	-0.04 [-0.15; 0.08]
State of Palestine¶	1.11 [1.03; 1.19]	1.18 [1.08; 1.30]	0.07 [-0.05; 0.20]	13.8 [10.0; 19.0]	11.7 [8.4; 16.2]	1.11 [1.03; 1.19]*	1.05 [0.96; 1.15]	-0.06 [-0.18; 0.05]
Sudan	1.22 [1.15; 1.30]	1.26 [1.16; 1.38]	0.04 [-0.09; 0.17]	43.3 [31.6; 59.7]	34.3 [25.0; 47.5]	0.96 [0.91; 1.03]	0.97 [0.88; 1.06]	0.01 [-0.10; 0.11]

¶: Sex ratio is outlying in 1990. §: Sex ratio is outlying in 2021. *: The ratio of estimated to expected female mortality is significantly different from one. †: Change is significantly different from zero.

Continued on next page

Table 13 – continued from previous page

	Sex ratio IMR			Sex-specific IMR in 2021 (per 1,000)		Estimated/Expected female IMR		
	**1990**	**2021**	**Change 1990–2021**	**Male**	**Female**	**1990**	**2021**	**Change 1990–2021**
Suriname	1.25 [1.13; 1.38]	1.28 [1.15; 1.43]	0.03 [-0.11; 0.18]	17.2 [10.8; 27.4]	13.4 [8.4; 21.7]	0.98 [0.89; 1.08]	0.97 [0.87; 1.08]	-0.01 [-0.12; 0.10]
Eswatini	1.26 [1.16; 1.36]	1.24 [1.12; 1.37]	-0.02 [-0.16; 0.12]	45.8 [29.2; 69.4]	36.9 [23.7; 57.2]	0.96 [0.89; 1.04]	0.99 [0.89; 1.09]	0.03 [-0.08; 0.14]
Sweden	1.25 [1.20; 1.30]	1.20 [1.13; 1.28]	-0.05 [-0.14; 0.04]	2.2 [2.0; 2.4]	1.8 [1.7; 2.0]	0.96 [0.92; 1.00]	0.99 [0.93; 1.05]	0.03 [-0.05; 0.10]
Switzerland	1.27 [1.21; 1.32]	1.17 [1.10; 1.24]	-0.10 [-0.19; -0.01]†	3.6 [3.4; 3.9]	3.1 [2.9; 3.3]	0.96 [0.92; 1.00]*	1.01 [0.95; 1.08]	0.06 [-0.02; 0.13]
Syria	1.19 [1.12; 1.27]	1.23 [1.11; 1.36]	0.04 [-0.09; 0.18]	20.3 [10.5; 27.8]	16.4 [8.6; 22.5]	1.04 [0.97; 1.11]	1.00 [0.91; 1.11]	-0.04 [-0.14; 0.08]
Tajikistan	1.21 [1.13; 1.31]	1.29 [1.18; 1.43]	0.08 [-0.05; 0.22]	31.1 [18.6; 51.9]	24.0 [14.2; 40.1]	0.97 [0.90; 1.05]	0.95 [0.87; 1.05]	-0.02 [-0.12; 0.09]
Tanzania¶	1.09 [1.04; 1.15]	1.19 [1.09; 1.31]	0.10 [-0.01; 0.23]	37.1 [26.7; 51.4]	31.0 [22.3; 43.1]	1.07 [1.01; 1.12]*	1.03 [0.94; 1.13]	-0.04 [-0.14; 0.08]
Thailand	1.26 [1.16; 1.36]	1.23 [1.10; 1.36]	-0.03 [-0.16; 0.11]	7.8 [6.5; 10.3]	6.4 [5.3; 8.4]	0.98 [0.91; 1.07]	0.99 [0.89; 1.10]	0.01 [-0.10; 0.11]
Timor Leste	1.11 [1.03; 1.20]	1.19 [1.09; 1.32]	0.08 [-0.04; 0.21]	46.7 [31.5; 67.6]	39.1 [26.4; 56.8]	1.03 [0.95; 1.12]	1.02 [0.92; 1.13]	-0.01 [-0.12; 0.12]
Togo	1.18 [1.11; 1.26]	1.22 [1.12; 1.33]	0.04 [-0.09; 0.16]	47.6 [37.5; 60.2]	38.9 [30.8; 49.5]	0.99 [0.93; 1.05]	1.00 [0.92; 1.10]	0.01 [-0.09; 0.12]
Tonga	1.28 [1.15; 1.43]	1.27 [1.15; 1.40]	-0.01 [-0.16; 0.13]	10.7 [5.7; 18.6]	8.4 [4.5; 14.6]	0.97 [0.87; 1.08]	0.97 [0.87; 1.07]	0.00 [-0.12; 0.11]
Trinidad and Tobago	1.20 [1.08; 1.32]	1.22 [1.09; 1.37]	0.03 [-0.11; 0.17]	16.0 [6.5; 39.3]	13.1 [5.3; 32.2]	1.04 [0.94; 1.15]	1.01 [0.90; 1.13]	-0.03 [-0.15; 0.09]
Tunisia	1.19 [1.11; 1.27]	1.20 [1.13; 1.28]	0.01 [-0.09; 0.12]	15.3 [12.9; 18.0]	12.7 [10.7; 15.0]	1.03 [0.96; 1.10]	1.03 [0.97; 1.10]	0.01 [-0.09; 0.10]
Turkey¶	1.12 [1.06; 1.18]	1.15 [1.10; 1.20]	0.03 [-0.05; 0.11]	8.2 [7.0; 9.7]	7.2 [6.1; 8.5]	1.08 [1.02; 1.15]*	1.06 [1.02; 1.11]*	-0.02 [-0.10; 0.05]
Turkmenistan¶	1.33 [1.21; 1.48]	1.34 [1.20; 1.50]	0.01 [-0.15; 0.16]	40.9 [27.3; 59.6]	30.5 [20.3; 44.8]	0.90 [0.80; 0.99]*	0.92 [0.82; 1.02]	0.02 [-0.08; 0.13]
Turks and Caicos Islands¶	1.54 [1.45; 1.64]	1.05 [0.97; 1.13]	-0.49 [-0.61; -0.37]†	3.9 [2.3; 6.7]	3.7 [2.2; 6.4]	0.80 [0.76; 0.85]*	1.13 [1.05; 1.22]*	0.33 [0.23; 0.43]†
Tuvalu	1.27 [1.14; 1.40]	1.27 [1.14; 1.41]	0.00 [-0.14; 0.15]	20.2 [11.4; 34.1]	15.9 [9.0; 26.9]	0.97 [0.87; 1.07]	0.98 [0.88; 1.09]	0.01 [-0.10; 0.13]
Uganda	1.15 [1.09; 1.20]	1.24 [1.14; 1.35]	0.09 [-0.02; 0.22]	34.4 [25.2; 45.9]	27.7 [20.2; 36.9]	1.01 [0.96; 1.07]	1.00 [0.91; 1.09]	-0.02 [-0.11; 0.08]
Ukraine	1.26 [1.14; 1.40]	1.22 [1.09; 1.37]	-0.04 [-0.19; 0.11]	7.7 [7.2; 8.3]	6.3 [5.8; 6.8]	0.99 [0.89; 1.09]	0.99 [0.89; 1.11]	0.00 [-0.11; 0.12]
United Arab Emirates	1.29 [1.19; 1.41]	1.22 [1.12; 1.33]	-0.07 [-0.21; 0.06]	6.0 [5.1; 7.0]	4.9 [4.2; 5.8]	0.96 [0.88; 1.05]	0.98 [0.90; 1.07]	0.02 [-0.08; 0.12]
United Kingdom	1.30 [1.27; 1.33]	1.21 [1.15; 1.26]	-0.10 [-0.16; -0.04]†	4.0 [3.8; 4.2]	3.3 [3.1; 3.5]	0.94 [0.92; 0.96]*	0.98 [0.94; 1.03]	0.05 [0.00; 0.10]†
United States of America	1.26 [1.24; 1.27]	1.20 [1.15; 1.25]	-0.06 [-0.11; -0.01]†	5.8 [5.5; 6.2]	4.9 [4.6; 5.2]	0.98 [0.97; 0.99]*	1.00 [0.96; 1.04]	0.02 [-0.02; 0.06]
Uruguay	1.26 [1.21; 1.30]	1.23 [1.15; 1.31]	-0.03 [-0.12; 0.07]	5.5 [5.0; 6.1]	4.5 [4.0; 4.9]	0.99 [0.96; 1.03]	0.97 [0.91; 1.03]	-0.02 [-0.09; 0.05]
Uzbekistan¶	1.33 [1.23; 1.45]	1.32 [1.19; 1.47]	-0.01 [-0.16; 0.13]	14.3 [12.8; 16.3]	10.8 [9.6; 12.5]	0.90 [0.83; 0.98]*	0.94 [0.84; 1.05]	0.04 [-0.06; 0.15]
Vanuatu	1.17 [1.04; 1.32]	1.18 [1.04; 1.31]	0.01 [-0.14; 0.14]	21.3 [12.2; 36.2]	18.1 [10.3; 31.0]	1.05 [0.94; 1.20]	1.06 [0.95; 1.19]	0.01 [-0.12; 0.13]
Venezuela (Bolivarian Republic of)	1.25 [1.22; 1.27]	1.19 [1.11; 1.27]	-0.06 [-0.14; 0.03]	22.8 [16.8; 30.7]	19.1 [14.1; 25.7]	1.00 [0.98; 1.02]	0.99 [0.93; 1.06]	-0.01 [-0.07; 0.07]
Vietnam¶	1.33 [1.24; 1.42]	1.31 [1.22; 1.41]	-0.01 [-0.13; 0.11]	18.6 [16.4; 21.2]	14.2 [12.4; 16.2]	0.93 [0.87; 0.99]*	0.95 [0.88; 1.02]	0.02 [-0.06; 0.11]
Yemen	1.18 [1.13; 1.23]	1.19 [1.09; 1.31]	0.02 [-0.10; 0.14]	51.0 [24.6; 98.4]	42.7 [20.6; 82.2]	1.00 [0.95; 1.04]	1.01 [0.92; 1.12]	0.01 [-0.09; 0.12]
Zambia	1.14 [1.08; 1.19]	1.20 [1.11; 1.31]	0.07 [-0.04; 0.19]	43.8 [33.2; 57.8]	36.3 [27.5; 48.1]	1.02 [0.97; 1.07]	1.02 [0.93; 1.11]	0.00 [-0.10; 0.10]
Zimbabwe	1.28 [1.20; 1.36]	1.25 [1.15; 1.36]	-0.03 [-0.15; 0.10]	39.6 [28.8; 53.7]	31.6 [23.1; 42.9]	0.95 [0.89; 1.01]	0.98 [0.90; 1.07]	0.04 [-0.07; 0.14]

Table 14: **Estimates and 90% uncertainty intervals for sex ratios for CMR in 1990 and 2021, the change in sex ratios from 1990 to 2021, sex-specific CMR in 2021, and ratios of estimated to expected female CMR and their change from 1990 to 2021 for the world, UNICEF regions, and all countries.** ¶: Sex ratio is outlying in 1990. §: Sex ratio is outlying in 2021. *: The ratio of estimated to expected female mortality is significantly different from one. †: Change is significantly different from zero.

	Sex ratio CMR			Sex-specific CMR in 2021 (per 1,000)		Estimated/Expected female CMR		
	1990	2021	Change 1990–2021	Male	Female	1990	2021	Change 1990–2021
World¶	0.91 [0.90; 0.93]	1.01 [0.99; 1.04]	0.10 [0.07; 0.13]†	10.0 [8.8; 11.4]	9.9 [8.7; 11.3]	1.11 [1.09; 1.13]*	1.03 [1.00; 1.06]	-0.08 [-0.11; -0.05]†
South Asia¶§	0.76 [0.73; 0.78]	0.88 [0.84; 0.93]	0.13 [0.08; 0.18]†	6.1 [5.3; 7.0]	6.9 [6.0; 7.9]	1.34 [1.30; 1.38]*	1.30 [1.23; 1.38]*	-0.03 [-0.12; 0.05]
Europe and Central Asia	1.07 [1.04; 1.11]	1.19 [1.16; 1.22]	0.12 [0.07; 0.16]†	1.2 [1.1; 1.3]	1.0 [0.9; 1.1]	1.02 [0.99; 1.06]	1.01 [0.97; 1.03]	-0.02 [-0.06; 0.02]
Middle East and North Africa¶	0.92 [0.89; 0.95]	1.06 [1.01; 1.12]	0.15 [0.09; 0.20]†	4.0 [2.9; 7.1]	3.8 [2.7; 6.6]	1.16 [1.12; 1.20]*	1.08 [0.98; 1.14]	-0.08 [-0.18; -0.01]†
Sub-Saharan Africa¶	1.04 [1.03; 1.06]	1.05 [1.02; 1.09]	0.01 [-0.03; 0.04]	24.9 [22.1; 31.5]	23.7 [21.0; 30.1]	0.96 [0.95; 0.98]*	0.98 [0.95; 1.01]	0.01 [-0.02; 0.05]
Latin America and Caribbean	1.06 [1.02; 1.10]	1.16 [1.12; 1.20]	0.10 [0.06; 0.15]†	2.5 [2.3; 2.9]	2.2 [2.0; 2.5]	1.02 [0.97; 1.06]	1.01 [0.97; 1.05]	-0.01 [-0.06; 0.04]
East Asia and Pacific	1.03 [0.96; 1.11]	1.19 [1.11; 1.25]	0.16 [0.08; 0.22]†	3.2 [2.7; 3.5]	2.7 [2.3; 3.0]	1.03 [0.96; 1.11]	0.99 [0.93; 1.05]	-0.04 [-0.11; 0.02]
North America	1.25 [1.22; 1.28]	1.24 [1.19; 1.31]	0.00 [-0.06; 0.06]	1.0 [0.9; 1.0]	0.8 [0.7; 0.8]	0.96 [0.94; 0.98]*	0.96 [0.92; 1.01]	0.00 [-0.05; 0.05]
Afghanistan	0.95 [0.88; 1.04]	1.06 [0.96; 1.16]	0.11 [-0.01; 0.21]	13.2 [8.3; 20.4]	12.5 [7.9; 19.2]	1.05 [0.97; 1.14]	1.03 [0.92; 1.15]	-0.02 [-0.15; 0.11]
Albania¶	0.78 [0.69; 0.88]	0.79 [0.72; 0.90]	0.01 [-0.07; 0.12]	0.9 [0.9; 1.0]	1.2 [1.1; 1.3]	1.53 [1.36; 1.72]*	1.48 [1.33; 1.66]*	-0.05 [-0.23; 0.13]
Algeria	1.13 [1.08; 1.19]	1.08 [1.00; 1.16]	-0.05 [-0.15; 0.04]	3.4 [2.9; 3.8]	3.1 [2.7; 3.6]	1.00 [0.95; 1.06]	1.11 [1.03; 1.20]*	0.12 [0.02; 0.22]†
Andorra¶	1.81 [1.59; 2.00]	2.64 [1.68; 2.07]	0.83 [-0.13; 0.28]	0.2 [0.0; 0.6]	0.1 [0.0; 0.3]	0.65 [0.56; 0.74]*	0.64 [0.58; 0.71]*	-0.01 [-0.09; 0.09]
Angola	1.07 [0.97; 1.18]	1.08 [0.96; 1.21]	0.01 [-0.11; 0.14]	24.3 [6.6; 59.2]	22.5 [6.1; 54.6]	0.94 [0.85; 1.04]	0.97 [0.84; 1.12]	0.02 [-0.10; 0.18]
Anguilla	1.18 [1.09; 1.27]	1.15 [1.13; 1.36]	-0.03 [-0.06; 0.19]	0.1 [0.1; 0.3]	0.1 [0.1; 0.3]	1.02 [0.94; 1.10]	0.97 [0.88; 1.06]	-0.05 [-0.15; 0.05]
Antigua and Barbuda	1.22 [1.09; 1.38]	1.22 [1.08; 1.39]	0.00 [-0.14; 0.14]	1.1 [0.7; 1.7]	0.9 [0.6; 1.4]	0.98 [0.87; 1.11]	0.98 [0.87; 1.12]	0.00 [-0.11; 0.12]
Argentina	1.15 [1.12; 1.19]	1.14 [1.09; 1.21]	-0.01 [-0.07; 0.07]	0.8 [0.8; 0.9]	0.7 [0.7; 0.8]	1.04 [1.01; 1.07]*	1.04 [0.99; 1.10]	0.00 [-0.06; 0.07]
Armenia	1.17 [1.02; 1.35]	1.21 [1.05; 1.42]	0.04 [-0.09; 0.19]	1.3 [1.0; 1.6]	1.1 [0.8; 1.3]	0.98 [0.84; 1.13]	0.99 [0.85; 1.14]	0.01 [-0.11; 0.13]
Australia	1.28 [1.22; 1.34]	1.21 [1.13; 1.32]	-0.07 [-0.17; 0.05]	0.6 [0.6; 0.7]	0.5 [0.5; 0.5]	0.94 [0.89; 0.98]*	0.99 [0.91; 1.06]	0.05 [-0.03; 0.13]
Austria	1.26 [1.18; 1.33]	1.26 [1.15; 1.38]	0.01 [-0.12; 0.14]	0.7 [0.7; 0.8]	0.6 [0.5; 0.7]	0.96 [0.90; 1.02]	0.95 [0.87; 1.05]	0.00 [-0.10; 0.10]
Azerbaijan	1.00 [0.88; 1.13]	1.16 [1.01; 1.33]	0.16 [0.04; 0.30]†	2.2 [1.3; 4.2]	1.9 [1.1; 3.6]	1.04 [0.91; 1.19]	1.03 [0.90; 1.19]	-0.01 [-0.13; 0.11]
Bahamas	1.15 [1.04; 1.27]	1.18 [1.05; 1.32]	0.03 [-0.11; 0.16]	1.9 [1.6; 2.2]	1.6 [1.4; 1.9]	1.04 [0.94; 1.16]	1.02 [0.91; 1.15]	-0.02 [-0.14; 0.10]
Bahrain	1.17 [1.06; 1.28]	1.11 [1.02; 1.21]	-0.06 [-0.18; 0.06]	1.1 [0.9; 1.2]	1.0 [0.8; 1.1]	1.03 [0.94; 1.13]	1.08 [0.99; 1.18]	0.05 [-0.06; 0.16]
Bangladesh¶	0.85 [0.81; 0.89]	1.18 [1.10; 1.27]	0.33 [0.24; 0.43]†	4.8 [4.2; 5.6]	4.1 [3.5; 4.8]	1.19 [1.13; 1.24]*	1.02 [0.95; 1.10]	-0.17 [-0.26; -0.08]†
Barbados	1.21 [1.08; 1.35]	1.21 [1.07; 1.38]	0.00 [-0.13; 0.15]	0.9 [0.6; 1.5]	0.8 [0.5; 1.2]	0.99 [0.89; 1.11]	0.99 [0.87; 1.12]	-0.01 [-0.12; 0.11]
Belarus	1.29 [1.23; 1.35]	1.32 [1.22; 1.44]	0.03 [-0.08; 0.17]	0.8 [0.7; 0.9]	0.6 [0.5; 0.7]	0.93 [0.89; 0.97]*	0.90 [0.83; 0.98]*	-0.03 [-0.11; 0.06]
Belgium	1.22 [1.15; 1.28]	1.23 [1.13; 1.34]	0.01 [-0.10; 0.14]	0.8 [0.7; 0.9]	0.6 [0.6; 0.7]	0.99 [0.94; 1.05]	0.98 [0.90; 1.06]	-0.01 [-0.11; 0.08]
Belize	1.12 [0.99; 1.26]	1.17 [1.04; 1.33]	0.05 [-0.08; 0.19]	1.7 [1.4; 2.1]	1.5 [1.2; 1.8]	1.03 [0.91; 1.18]	1.02 [0.91; 1.16]	0.00 [-0.13; 0.12]
Benin	1.02 [0.97; 1.08]	1.04 [0.95; 1.14]	0.02 [-0.08; 0.13]	30.6 [23.0; 40.5]	29.4 [22.1; 38.9]	0.98 [0.93; 1.04]	0.97 [0.89; 1.07]	-0.01 [-0.10; 0.09]
Bhutan	0.99 [0.88; 1.12]	1.18 [1.04; 1.36]	0.19 [0.07; 0.33]†	4.7 [2.4; 10.0]	4.0 [2.0; 8.4]	1.01 [0.90; 1.15]	0.99 [0.86; 1.14]	-0.02 [-0.14; 0.11]
Bolivia (Plurinational State of)	0.99 [0.93; 1.05]	1.14 [1.03; 1.27]	0.15 [0.04; 0.28]†	4.9 [3.4; 7.6]	4.3 [2.9; 6.7]	1.02 [0.96; 1.09]	1.04 [0.93; 1.16]	0.02 [-0.09; 0.14]
Bosnia and Herzegovina	1.33 [1.22; 1.44]	1.27 [1.15; 1.41]	-0.06 [-0.18; 0.08]	0.9 [0.7; 1.1]	0.7 [0.5; 0.9]	0.90 [0.83; 0.98]*	0.94 [0.85; 1.05]	0.04 [-0.06; 0.14]
Botswana	1.09 [0.98; 1.25]	1.17 [1.03; 1.33]	0.07 [-0.06; 0.21]	7.3 [1.8; 40.9]	6.2 [1.5; 35.1]	1.00 [0.87; 1.13]	0.97 [0.81; 1.14]	-0.02 [-0.18; 0.13]
Brazil	1.13 [1.00; 1.26]	1.22 [1.07; 1.38]	0.09 [-0.03; 0.23]	1.7 [1.5; 2.0]	1.4 [1.2; 1.7]	0.98 [0.86; 1.10]	0.99 [0.87; 1.12]	0.01 [-0.11; 0.12]
British Virgin Islands	1.27 [1.20; 1.35]	1.50 [1.33; 1.64]	0.23 [0.05; 0.37]†	0.7 [0.3; 1.4]	0.4 [0.2; 1.0]	0.94 [0.89; 1.00]*	0.81 [0.73; 0.90]*	-0.13 [-0.22; -0.03]†
Brunei	1.21 [1.10; 1.34]	1.22 [1.08; 1.35]	0.00 [-0.14; 0.14]	2.0 [1.7; 2.5]	1.7 [1.4; 2.0]	0.99 [0.90; 1.09]	0.99 [0.89; 1.11]	0.00 [-0.11; 0.12]
Bulgaria	1.20 [1.15; 1.26]	1.13 [1.03; 1.22]	-0.08 [-0.18; 0.03]	1.1 [1.0; 1.2]	1.0 [0.9; 1.1]	1.00 [0.95; 1.04]	1.07 [0.98; 1.16]	0.07 [-0.02; 0.17]
Burkina Faso	1.02 [0.97; 1.07]	1.01 [0.92; 1.11]	0.00 [-0.11; 0.10]	32.8 [17.7; 58.0]	32.4 [17.5; 57.6]	0.99 [0.94; 1.05]	1.01 [0.91; 1.11]	0.02 [-0.09; 0.13]
Burundi	0.99 [0.92; 1.08]	1.07 [0.95; 1.20]	0.08 [-0.05; 0.21]	16.1 [7.3; 32.4]	15.0 [7.2; 29.3]	1.01 [0.93; 1.10]	0.99 [0.87; 1.15]	-0.02 [-0.15; 0.15]
Cambodia	1.00 [0.93; 1.07]	1.19 [1.07; 1.32]	0.19 [0.06; 0.32]†	3.9 [1.9; 9.0]	3.3 [1.6; 7.6]	1.01 [0.94; 1.09]	1.00 [0.89; 1.12]	-0.01 [-0.13; 0.11]
Cameroon	1.02 [0.96; 1.09]	1.09 [0.99; 1.19]	0.07 [-0.04; 0.18]	24.9 [17.0; 34.9]	22.9 [15.6; 32.2]	0.98 [0.93; 1.04]	0.95 [0.86; 1.04]	-0.04 [-0.14; 0.07]
Canada	1.24 [1.19; 1.29]	1.19 [1.11; 1.28]	-0.05 [-0.14; 0.05]	0.7 [0.6; 0.7]	0.6 [0.5; 0.6]	0.97 [0.93; 1.01]	1.01 [0.94; 1.08]	0.04 [-0.04; 0.12]

¶: Sex ratio is outlying in 1990. §: Sex ratio is outlying in 2021. *: The ratio of estimated to expected female mortality is significantly different from one. †: Change is significantly different from zero.

Continued on next page

Table 14 – continued from previous page

	Sex ratio CMR			Sex-specific CMR in 2021 (per 1,000)		Estimated/Expected female CMR		
	1990	2021	Change 1990–2021	Male	Female	1990	2021	Change 1990–2021
Cape Verde	1.06 [0.99; 1.14]	1.18 [1.07; 1.32]	0.12 [0.00; 0.26]†	2.1 [1.6; 2.7]	1.7 [1.3; 2.3]	1.01 [0.94; 1.09]	1.01 [0.91; 1.12]	0.00 [-0.11; 0.12]
Central African Republic	0.97 [0.89; 1.05]	0.99 [0.88; 1.08]	0.02 [-0.10; 0.12]	26.3 [17.4; 39.5]	26.6 [17.7; 40.3]	1.04 [0.96; 1.13]	1.05 [0.94; 1.17]	0.01 [-0.11; 0.13]
Chad	1.06 [1.00; 1.12]	1.03 [0.94; 1.13]	-0.03 [-0.13; 0.08]	44.5 [25.7; 73.1]	43.3 [25.0; 70.9]	0.95 [0.90; 1.01]	0.98 [0.89; 1.08]	0.03 [-0.07; 0.13]
Chile	1.28 [1.23; 1.33]	1.19 [1.09; 1.29]	-0.09 [-0.19; 0.02]	1.0 [0.9; 1.2]	0.9 [0.7; 1.0]	0.94 [0.91; 0.98]*	1.01 [0.93; 1.10]	0.07 [-0.01; 0.16]
China	1.01 [0.89; 1.14]	1.12 [0.97; 1.29]	0.12 [-0.01; 0.24]	2.0 [1.8; 2.3]	1.8 [1.6; 2.0]	1.09 [0.96; 1.25]	1.07 [0.93; 1.23]	-0.02 [-0.15; 0.10]
Colombia	1.14 [1.04; 1.25]	1.19 [1.05; 1.33]	0.04 [-0.09; 0.18]	2.0 [1.3; 3.1]	1.7 [1.1; 2.6]	1.02 [0.93; 1.13]	1.01 [0.91; 1.14]	-0.01 [-0.12; 0.12]
Comoros	1.01 [0.90; 1.14]	1.07 [0.94; 1.21]	0.06 [-0.07; 0.18]	11.2 [7.2; 17.4]	10.5 [6.7; 16.2]	0.99 [0.88; 1.12]	1.04 [0.90; 1.20]	0.04 [-0.09; 0.18]
Congo	1.01 [0.92; 1.11]	1.13 [1.00; 1.26]	0.12 [-0.01; 0.25]	12.0 [5.2; 29.1]	10.6 [4.8; 25.2]	1.00 [0.91; 1.10]	0.97 [0.84; 1.14]	-0.03 [-0.17; 0.14]
Democratic Republic of the Congo	1.04 [0.95; 1.14]	1.10 [0.99; 1.24]	0.06 [-0.05; 0.20]	18.5 [7.9; 44.8]	16.8 [7.1; 40.8]	0.97 [0.88; 1.06]	0.96 [0.84; 1.10]	-0.01 [-0.13; 0.13]
Cook Islands¶§	0.32 [0.25; 0.40]	0.29 [0.24; 0.41]	-0.03 [-0.03; 0.04]	0.5 [0.3; 0.9]	1.6 [0.9; 2.9]	3.82 [2.98; 4.90]*	3.79 [2.90; 4.97]*	-0.03 [-0.45; 0.46]
Costa Rica	1.21 [1.14; 1.28]	1.25 [1.15; 1.36]	0.04 [-0.07; 0.17]	1.6 [1.4; 1.7]	1.3 [1.1; 1.4]	0.99 [0.94; 1.05]	0.96 [0.88; 1.04]	-0.03 [-0.13; 0.06]
Cote d'Ivoire¶	1.15 [1.07; 1.23]	1.12 [1.02; 1.25]	-0.02 [-0.14; 0.11]	21.3 [13.7; 32.4]	18.9 [12.1; 28.5]	0.88 [0.82; 0.94]*	0.92 [0.82; 1.03]	0.04 [-0.06; 0.15]
Croatia	1.26 [1.16; 1.36]	1.06 [0.97; 1.17]	-0.20 [-0.31; -0.07]†	0.7 [0.7; 0.8]	0.7 [0.6; 0.8]	0.96 [0.88; 1.03]	1.13 [1.02; 1.24]*	0.17 [0.06; 0.29]†
Cuba	1.22 [1.17; 1.27]	1.19 [1.10; 1.28]	-0.03 [-0.13; 0.08]	1.1 [0.9; 1.4]	0.9 [0.7; 1.2]	0.99 [0.94; 1.03]	1.01 [0.94; 1.09]	0.03 [-0.06; 0.11]
Cyprus	1.17 [1.03; 1.34]	1.18 [1.03; 1.35]	0.01 [-0.13; 0.14]	0.5 [0.4; 0.8]	0.5 [0.3; 0.7]	1.03 [0.89; 1.16]	1.02 [0.89; 1.17]	-0.01 [-0.12; 0.12]
Czech Republic	1.25 [1.17; 1.33]	1.23 [1.14; 1.32]	-0.02 [-0.13; 0.09]	0.6 [0.6; 0.7]	0.5 [0.5; 0.5]	0.96 [0.90; 1.02]	0.98 [0.91; 1.05]	0.02 [-0.07; 0.10]
Denmark	1.23 [1.15; 1.32]	1.22 [1.11; 1.35]	-0.01 [-0.13; 0.13]	0.5 [0.5; 0.6]	0.4 [0.4; 0.5]	0.98 [0.91; 1.05]	0.98 [0.89; 1.08]	0.00 [-0.10; 0.11]
Djibouti	1.08 [0.94; 1.27]	1.21 [1.04; 1.40]	0.12 [-0.02; 0.25]	9.4 [3.9; 23.0]	7.8 [3.2; 19.2]	0.94 [0.80; 1.08]	0.94 [0.78; 1.10]	0.00 [-0.14; 0.14]
Dominica	1.20 [1.06; 1.36]	1.21 [1.06; 1.37]	0.01 [-0.14; 0.15]	4.4 [3.7; 5.2]	3.6 [3.0; 4.3]	1.00 [0.88; 1.13]	1.00 [0.88; 1.13]	-0.01 [-0.12; 0.11]
Dominican Republic	1.10 [1.02; 1.20]	1.19 [1.07; 1.33]	0.09 [-0.03; 0.23]	6.4 [4.2; 10.0]	5.3 [3.5; 8.4]	0.97 [0.89; 1.06]	0.98 [0.87; 1.10]	0.01 [-0.11; 0.13]
Ecuador	1.05 [0.94; 1.16]	1.16 [1.04; 1.29]	0.12 [-0.01; 0.24]	1.9 [1.7; 2.2]	1.6 [1.4; 1.9]	1.04 [0.94; 1.17]	1.03 [0.93; 1.15]	-0.01 [-0.13; 0.11]
Egypt¶	0.84 [0.79; 0.88]	1.07 [0.95; 1.18]	0.24 [0.11; 0.35]†	2.9 [1.8; 5.1]	2.7 [1.7; 4.8]	1.24 [1.16; 1.31]*	1.13 [1.01; 1.27]*	-0.10 [-0.23; 0.03]
El Salvador	1.08 [0.96; 1.23]	1.21 [1.08; 1.38]	0.13 [0.00; 0.26]†	1.9 [1.2; 3.5]	1.6 [1.0; 2.9]	0.99 [0.87; 1.12]	0.99 [0.87; 1.12]	0.00 [-0.12; 0.12]
Equatorial Guinea	1.02 [0.90; 1.17]	1.06 [0.93; 1.21]	0.04 [-0.09; 0.16]	21.3 [8.7; 47.4]	20.0 [8.1; 44.6]	0.98 [0.86; 1.12]	0.99 [0.85; 1.16]	0.01 [-0.12; 0.15]
Eritrea	1.08 [1.01; 1.16]	1.19 [1.06; 1.32]	0.11 [-0.02; 0.24]	10.2 [4.7; 24.9]	8.6 [3.9; 21.4]	0.93 [0.87; 1.00]	0.94 [0.82; 1.08]	0.01 [-0.11; 0.15]
Estonia	1.28 [1.18; 1.38]	1.22 [1.10; 1.38]	-0.05 [-0.18; 0.10]	0.5 [0.4; 0.6]	0.4 [0.3; 0.5]	0.94 [0.87; 1.02]	0.97 [0.87; 1.10]	0.03 [-0.07; 0.15]
Ethiopia	1.05 [0.99; 1.11]	1.12 [1.01; 1.24]	0.07 [-0.05; 0.19]	13.7 [9.2; 20.3]	12.2 [8.2; 18.1]	0.96 [0.90; 1.02]	0.97 [0.86; 1.08]	0.01 [-0.10; 0.13]
Federated States of Micronesia	1.13 [0.97; 1.33]	1.21 [1.06; 1.44]	0.08 [-0.04; 0.25]	4.3 [1.7; 13.8]	3.6 [1.4; 10.8]	0.98 [0.82; 1.15]	0.96 [0.80; 1.12]	-0.02 [-0.17; 0.11]
Fiji	1.14 [1.03; 1.26]	1.16 [1.05; 1.27]	0.02 [-0.12; 0.15]	4.9 [4.2; 5.7]	4.2 [3.6; 5.0]	1.05 [0.95; 1.16]	1.04 [0.94; 1.14]	-0.01 [-0.14; 0.11]
Finland	1.26 [1.17; 1.35]	1.23 [1.11; 1.36]	-0.03 [-0.15; 0.12]	0.4 [0.4; 0.5]	0.3 [0.3; 0.4]	0.96 [0.89; 1.03]	0.98 [0.88; 1.08]	0.02 [-0.08; 0.12]
France	1.29 [1.24; 1.33]	1.21 [1.14; 1.29]	-0.08 [-0.16; 0.02]	1.0 [0.9; 1.1]	0.8 [0.7; 0.9]	0.93 [0.90; 0.97]*	0.99 [0.93; 1.05]	0.06 [-0.01; 0.13]
Gabon	1.02 [0.92; 1.14]	1.12 [0.99; 1.26]	0.09 [-0.03; 0.23]	11.3 [5.9; 24.6]	10.1 [5.3; 21.8]	0.99 [0.89; 1.10]	0.99 [0.85; 1.14]	0.00 [-0.14; 0.14]
The Gambia	1.04 [0.95; 1.15]	1.09 [0.98; 1.22]	0.05 [-0.07; 0.17]	15.0 [9.4; 22.9]	13.8 [8.6; 21.0]	0.96 [0.88; 1.06]	0.98 [0.87; 1.11]	0.02 [-0.10; 0.14]
Georgia	1.14 [0.92; 1.32]	1.18 [0.97; 1.38]	0.04 [-0.09; 0.19]	1.1 [1.0; 1.3]	1.0 [0.8; 1.1]	1.01 [0.87; 1.26]	1.01 [0.87; 1.24]	0.00 [-0.13; 0.12]
Germany	1.24 [1.19; 1.28]	1.14 [1.08; 1.21]	-0.10 [-0.17; -0.01]†	0.6 [0.5; 0.6]	0.5 [0.5; 0.5]	0.97 [0.94; 1.01]	1.05 [0.99; 1.11]	0.08 [0.01; 0.15]†
Ghana	1.06 [1.00; 1.13]	1.19 [1.08; 1.31]	0.13 [0.00; 0.25]†	12.8 [8.2; 19.8]	10.8 [7.0; 16.7]	0.95 [0.89; 1.01]	0.92 [0.82; 1.03]	-0.03 [-0.13; 0.09]
Greece	1.17 [1.10; 1.24]	1.13 [1.03; 1.23]	-0.03 [-0.15; 0.07]	0.5 [0.4; 0.5]	0.4 [0.4; 0.5]	1.03 [0.97; 1.09]	1.07 [0.98; 1.17]	0.04 [-0.06; 0.14]
Grenada	1.19 [1.06; 1.33]	1.22 [1.07; 1.35]	0.03 [-0.12; 0.16]	2.0 [1.6; 2.4]	1.6 [1.3; 2.0]	1.01 [0.90; 1.13]	1.00 [0.89; 1.12]	-0.01 [-0.13; 0.11]
Guatemala¶	0.96 [0.88; 1.03]	1.13 [1.02; 1.26]	0.18 [0.07; 0.31]†	3.7 [2.5; 6.0]	3.3 [2.2; 5.2]	1.09 [1.00; 1.18]*	1.05 [0.95; 1.18]	-0.03 [-0.15; 0.09]
Guinea	0.99 [0.94; 1.05]	1.04 [0.95; 1.14]	0.04 [-0.06; 0.16]	38.0 [28.6; 50.8]	36.6 [28.0; 48.3]	1.01 [0.96; 1.07]	0.97 [0.88; 1.07]	-0.04 [-0.14; 0.06]
Guinea-Bissau	1.04 [0.96; 1.14]	1.07 [0.97; 1.20]	0.03 [-0.09; 0.16]	26.4 [11.7; 51.0]	24.7 [10.9; 47.6]	0.96 [0.88; 1.05]	0.96 [0.85; 1.08]	0.00 [-0.12; 0.12]
Guyana	1.13 [1.01; 1.27]	1.29 [1.13; 1.47]	0.15 [0.01; 0.30]†	5.1 [2.5; 11.6]	4.0 [2.0; 9.1]	0.94 [0.83; 1.06]	0.92 [0.79; 1.05]	-0.02 [-0.14; 0.10]
Haiti	1.03 [0.96; 1.11]	1.11 [1.00; 1.24]	0.08 [-0.03; 0.21]	14.6 [8.5; 25.5]	13.1 [7.7; 22.5]	0.97 [0.91; 1.04]	0.96 [0.85; 1.10]	-0.01 [-0.13; 0.12]
Honduras	1.07 [0.97; 1.18]	1.17 [1.04; 1.31]	0.10 [-0.03; 0.23]	2.5 [1.9; 3.7]	2.2 [1.6; 3.1]	1.00 [0.91; 1.12]	1.03 [0.91; 1.15]	0.02 [-0.09; 0.14]
Hungary	1.25 [1.18; 1.32]	1.17 [1.07; 1.28]	-0.07 [-0.19; 0.04]	0.7 [0.7; 0.8]	0.6 [0.6; 0.7]	0.96 [0.91; 1.02]	1.03 [0.94; 1.12]	0.06 [-0.03; 0.16]
Iceland	1.10 [0.97; 1.25]	1.12 [0.98; 1.26]	0.02 [-0.12; 0.14]	0.6 [0.4; 0.7]	0.5 [0.4; 0.7]	1.09 [0.96; 1.24]	1.07 [0.95; 1.23]	-0.01 [-0.14; 0.12]

¶: Sex ratio is outlying in 1990. §: Sex ratio is outlying in 2021. *: The ratio of estimated to expected female mortality is significantly different from one. †: Change is significantly different from zero.

Continued on next page

Table 14 – continued from previous page

	Sex ratio CMR			Sex-specific CMR in 2021 (per 1,000)		Estimated/Expected female CMR		
	1990	2021	Change 1990–2021	Male	Female	1990	2021	Change 1990–2021
India¶§	0.72 [0.70; 0.75]	0.82 [0.76; 0.88]	0.09 [0.03; 0.16]†	4.7 [4.1; 5.5]	5.8 [5.0; 6.7]	1.40 [1.35; 1.45]*	1.47 [1.36; 1.58]*	0.07 [-0.05; 0.19]
Indonesia	1.01 [0.96; 1.07]	1.17 [1.06; 1.30]	0.16 [0.05; 0.29]†	3.6 [2.7; 5.2]	3.1 [2.3; 4.4]	1.02 [0.96; 1.08]	1.02 [0.92; 1.13]	0.00 [-0.10; 0.12]
Iran (Islamic Republic of)¶	0.87 [0.79; 0.95]	1.02 [0.88; 1.15]	0.15 [0.03; 0.26]†	1.8 [1.0; 3.4]	1.8 [1.0; 3.4]	1.26 [1.15; 1.38]*	1.19 [1.04; 1.37]*	-0.07 [-0.20; 0.08]
Iraq	1.09 [0.99; 1.21]	1.19 [1.07; 1.35]	0.10 [-0.02; 0.24]	4.2 [2.8; 6.4]	3.5 [2.3; 5.3]	0.99 [0.90; 1.10]	1.00 [0.89; 1.12]	0.00 [-0.11; 0.12]
Ireland	1.24 [1.16; 1.32]	1.21 [1.10; 1.35]	-0.03 [-0.15; 0.11]	0.4 [0.4; 0.5]	0.4 [0.3; 0.4]	0.97 [0.91; 1.03]	0.99 [0.89; 1.09]	0.02 [-0.08; 0.13]
Israel	1.10 [1.05; 1.16]	1.17 [1.09; 1.26]	0.06 [-0.03; 0.17]	0.7 [0.7; 0.8]	0.6 [0.6; 0.7]	1.09 [1.03; 1.15]*	1.03 [0.96; 1.11]	-0.06 [-0.15; 0.03]
Italy	1.13 [1.09; 1.18]	1.18 [1.10; 1.27]	0.05 [-0.04; 0.15]	0.4 [0.4; 0.4]	0.4 [0.3; 0.4]	1.06 [1.02; 1.11]*	1.01 [0.95; 1.09]	-0.05 [-0.13; 0.04]
Jamaica	1.28 [1.12; 1.48]	1.27 [1.11; 1.48]	-0.01 [-0.16; 0.14]	1.9 [1.0; 4.3]	1.5 [0.8; 3.4]	0.94 [0.81; 1.07]	0.95 [0.81; 1.08]	0.01 [-0.10; 0.12]
Japan	1.29 [1.25; 1.33]	1.13 [1.06; 1.20]	-0.16 [-0.24; -0.09]†	0.6 [0.6; 0.6]	0.5 [0.5; 0.6]	0.93 [0.90; 0.96]*	1.07 [1.01; 1.13]*	0.14 [0.07; 0.21]†
Jordan	1.11 [1.01; 1.21]	1.17 [1.05; 1.29]	0.06 [-0.07; 0.19]	2.2 [1.6; 3.4]	1.9 [1.3; 2.9]	1.05 [0.96; 1.16]	1.03 [0.93; 1.15]	-0.02 [-0.14; 0.10]
Kazakhstan	1.17 [1.03; 1.32]	1.23 [1.08; 1.41]	0.06 [-0.08; 0.20]	1.3 [1.2; 1.3]	1.0 [0.9; 1.1]	0.97 [0.85; 1.11]	0.98 [0.85; 1.11]	0.01 [-0.11; 0.13]
Kenya	1.05 [0.99; 1.12]	1.13 [1.03; 1.25]	0.08 [-0.03; 0.20]	10.0 [7.3; 13.8]	8.8 [6.5; 12.2]	0.96 [0.90; 1.02]	0.99 [0.89; 1.10]	0.03 [-0.08; 0.14]
Kiribati	1.02 [0.89; 1.17]	1.11 [0.96; 1.29]	0.10 [-0.03; 0.22]	10.9 [4.3; 27.5]	9.8 [3.8; 24.5]	1.00 [0.87; 1.16]	1.00 [0.83; 1.18]	0.00 [-0.15; 0.15]
Democratic People's Republic of Korea	1.10 [0.95; 1.30]	1.19 [1.02; 1.39]	0.09 [-0.05; 0.22]	5.8 [4.5; 7.5]	4.8 [3.7; 6.3]	1.00 [0.85; 1.17]	0.99 [0.85; 1.17]	0.00 [-0.13; 0.12]
Republic of Korea	1.18 [1.09; 1.29]	1.19 [1.11; 1.27]	0.00 [-0.12; 0.13]	0.5 [0.4; 0.5]	0.4 [0.4; 0.4]	1.02 [0.94; 1.11]	1.01 [0.94; 1.08]	0.00 [-0.11; 0.10]
Kosovo	0.92 [0.75; 1.07]	1.00 [0.85; 1.16]	0.08 [-0.01; 0.20]	0.9 [0.8; 1.1]	0.9 [0.8; 1.1]	1.14 [0.98; 1.44]	1.18 [1.03; 1.42]*	0.04 [-0.12; 0.17]
Kuwait	1.14 [1.09; 1.20]	1.21 [1.11; 1.32]	0.07 [-0.04; 0.17]	1.4 [1.2; 1.5]	1.1 [1.0; 1.3]	0.99 [0.94; 1.04]	0.99 [0.91; 1.08]	0.00 [-0.08; 0.10]
Kyrgyzstan	1.11 [0.99; 1.28]	1.22 [1.07; 1.40]	0.11 [-0.03; 0.24]	2.1 [1.9; 2.2]	1.7 [1.5; 1.8]	0.98 [0.85; 1.12]	0.98 [0.86; 1.12]	0.00 [-0.12; 0.12]
Lao People's Democratic Republic	1.01 [0.91; 1.11]	1.14 [1.01; 1.27]	0.13 [0.00; 0.25]	9.1 [5.2; 15.5]	8.0 [4.6; 13.7]	1.00 [0.90; 1.10]	1.00 [0.88; 1.14]	0.00 [-0.12; 0.14]
Latvia	1.29 [1.20; 1.38]	1.27 [1.15; 1.41]	-0.01 [-0.15; 0.13]	0.6 [0.5; 0.7]	0.5 [0.4; 0.5]	0.93 [0.87; 1.00]	0.94 [0.85; 1.05]	0.01 [-0.09; 0.11]
Lebanon	1.21 [1.07; 1.38]	1.20 [1.07; 1.40]	-0.01 [-0.13; 0.15]	1.3 [1.1; 1.6]	1.1 [0.9; 1.3]	0.98 [0.85; 1.11]	0.99 [0.86; 1.12]	0.01 [-0.11; 0.13]
Lesotho	1.10 [0.99; 1.22]	1.11 [0.99; 1.25]	0.01 [-0.12; 0.14]	17.9 [10.6; 31.0]	16.1 [9.5; 28.1]	0.95 [0.85; 1.06]	0.95 [0.83; 1.08]	0.00 [-0.12; 0.13]
Liberia	1.05 [0.98; 1.13]	1.07 [0.96; 1.18]	0.01 [-0.10; 0.13]	21.1 [13.2; 33.2]	19.8 [12.6; 30.7]	0.96 [0.89; 1.02]	0.98 [0.87; 1.09]	0.02 [-0.09; 0.14]
Libya	1.05 [0.91; 1.18]	1.09 [0.93; 1.27]	0.04 [-0.07; 0.18]	1.6 [0.9; 2.9]	1.5 [0.8; 2.6]	1.12 [0.99; 1.30]	1.09 [0.95; 1.28]	-0.03 [-0.16; 0.11]
Lithuania	1.29 [1.22; 1.36]	1.26 [1.15; 1.38]	-0.03 [-0.16; 0.10]	0.7 [0.6; 0.8]	0.5 [0.5; 0.6]	0.93 [0.88; 0.99]*	0.96 [0.87; 1.05]	0.02 [-0.07; 0.12]
Luxembourg	1.22 [1.09; 1.37]	1.24 [1.10; 1.39]	0.02 [-0.12; 0.16]	0.6 [0.4; 0.7]	0.4 [0.3; 0.6]	0.98 [0.87; 1.10]	0.97 [0.86; 1.09]	-0.01 [-0.13; 0.10]
Macedonia	1.07 [1.01; 1.14]	1.24 [1.15; 1.34]	0.17 [0.06; 0.28]†	0.7 [0.6; 0.9]	0.6 [0.5; 0.8]	1.12 [1.05; 1.19]*	0.97 [0.90; 1.04]	-0.15 [-0.24; -0.06]†
Madagascar	1.05 [0.99; 1.11]	1.08 [0.98; 1.20]	0.03 [-0.07; 0.15]	22.6 [17.5; 29.4]	20.9 [16.1; 27.2]	0.96 [0.90; 1.02]	0.95 [0.86; 1.06]	-0.01 [-0.11; 0.10]
Malawi¶	1.09 [1.04; 1.15]	1.19 [1.09; 1.31]	0.10 [-0.01; 0.23]	11.9 [6.2; 23.8]	10.1 [5.1; 19.9]	0.92 [0.87; 0.97]*	0.92 [0.82; 1.04]	0.00 [-0.11; 0.12]
Malaysia	1.17 [1.04; 1.31]	1.16 [1.05; 1.28]	-0.01 [-0.14; 0.13]	1.2 [1.1; 1.3]	1.0 [0.9; 1.1]	1.03 [0.92; 1.15]	1.03 [0.94; 1.14]	0.01 [-0.11; 0.12]
Maldives	1.01 [0.90; 1.15]	1.18 [1.04; 1.34]	0.17 [0.03; 0.30]†	0.9 [0.8; 1.1]	0.8 [0.7; 1.0]	1.01 [0.89; 1.14]	1.02 [0.90; 1.15]	0.00 [-0.12; 0.13]
Mali	1.01 [0.97; 1.06]	1.00 [0.92; 1.10]	-0.01 [-0.10; 0.09]	37.9 [26.8; 52.8]	37.7 [26.6; 52.4]	0.99 [0.95; 1.04]	1.00 [0.92; 1.10]	0.01 [-0.09; 0.11]
Malta	1.15 [1.03; 1.28]	1.17 [1.03; 1.32]	0.02 [-0.11; 0.15]	0.8 [0.7; 1.0]	0.7 [0.6; 0.8]	1.04 [0.94; 1.17]	1.03 [0.91; 1.16]	-0.01 [-0.13; 0.11]
Marshall Islands	1.09 [0.94; 1.25]	1.16 [1.01; 1.35]	0.08 [-0.05; 0.22]	5.4 [2.7; 12.2]	4.7 [2.3; 10.4]	1.02 [0.88; 1.19]	1.00 [0.86; 1.17]	-0.02 [-0.17; 0.12]
Mauritania	1.05 [0.96; 1.14]	1.19 [1.07; 1.32]	0.14 [0.01; 0.26]†	9.3 [5.5; 15.2]	7.8 [4.6; 12.8]	0.96 [0.88; 1.05]	0.95 [0.84; 1.07]	-0.01 [-0.12; 0.11]
Mauritius	1.10 [1.03; 1.19]	1.11 [1.00; 1.22]	0.01 [-0.11; 0.12]	1.4 [1.2; 1.7]	1.2 [1.0; 1.5]	1.08 [1.01; 1.17]*	1.08 [0.98; 1.20]	0.00 [-0.11; 0.12]
Mexico	1.05 [0.96; 1.15]	1.14 [1.07; 1.21]	0.09 [-0.02; 0.20]	2.0 [1.7; 2.3]	1.7 [1.5; 2.1]	1.07 [0.97; 1.17]	1.06 [0.99; 1.12]	-0.01 [-0.13; 0.10]
Republic of Moldova	1.18 [1.02; 1.36]	1.20 [1.04; 1.39]	0.03 [-0.11; 0.17]	2.2 [1.6; 3.3]	1.8 [1.3; 2.8]	1.00 [0.86; 1.15]	1.00 [0.87; 1.15]	0.00 [-0.12; 0.13]
Monaco	1.25 [1.16; 1.34]	1.24 [1.14; 1.37]	-0.01 [-0.14; 0.13]	2.2 [1.4; 3.4]	1.7 [1.1; 2.8]	0.96 [0.89; 1.03]	0.96 [0.87; 1.06]	0.00 [-0.10; 0.11]
Mongolia	1.07 [0.97; 1.17]	1.20 [1.11; 1.29]	0.13 [0.01; 0.25]†	2.3 [2.1; 2.5]	1.9 [1.7; 2.1]	0.95 [0.86; 1.05]	1.00 [0.93; 1.08]	0.05 [-0.05; 0.15]
Montenegro	1.16 [1.01; 1.33]	1.18 [1.04; 1.35]	0.03 [-0.11; 0.16]	0.4 [0.3; 0.5]	0.3 [0.3; 0.4]	1.04 [0.90; 1.19]	1.02 [0.89; 1.16]	-0.02 [-0.14; 0.10]
Montserrat	1.04 [0.94; 1.15]	1.07 [0.94; 1.21]	0.03 [-0.09; 0.15]	0.8 [0.3; 2.1]	0.7 [0.3; 1.9]	1.15 [1.04; 1.27]*	1.12 [0.99; 1.29]	-0.03 [-0.16; 0.11]
Morocco	0.97 [0.90; 1.05]	1.12 [1.00; 1.27]	0.15 [0.04; 0.29]†	2.8 [1.9; 3.9]	2.5 [1.7; 3.5]	1.08 [1.00; 1.18]	1.07 [0.95; 1.19]	-0.02 [-0.14; 0.10]
Mozambique	1.06 [0.99; 1.13]	1.07 [0.98; 1.19]	0.02 [-0.10; 0.13]	20.2 [10.8; 38.5]	18.8 [10.2; 35.7]	0.95 [0.89; 1.01]	0.97 [0.87; 1.09]	0.02 [-0.09; 0.15]
Myanmar	1.00 [0.90; 1.11]	1.13 [0.99; 1.27]	0.13 [0.00; 0.26]†	8.8 [4.5; 16.7]	7.8 [4.1; 14.5]	1.01 [0.90; 1.13]	1.00 [0.87; 1.17]	-0.01 [-0.14; 0.14]
Namibia	1.08 [0.98; 1.19]	1.16 [1.04; 1.31]	0.08 [-0.04; 0.21]	10.7 [5.0; 28.7]	9.2 [4.2; 24.5]	0.95 [0.86; 1.04]	0.95 [0.82; 1.10]	0.01 [-0.13; 0.15]

¶: Sex ratio is outlying in 1990. §: Sex ratio is outlying in 2021. *: The ratio of estimated to expected female mortality is significantly different from one. †: Change is significantly different from zero.

Continued on next page

Table 14 – continued from previous page

	Sex ratio CMR			Sex-specific CMR in 2021 (per 1,000)		Estimated/Expected female CMR		
	1990	2021	Change 1990–2021	Male	Female	1990	2021	Change 1990–2021
Nauru	1.06 [0.91; 1.23]	1.19 [1.03; 1.40]	0.14 [0.01; 0.28]†	4.9 [2.1; 13.1]	4.1 [1.8; 10.9]	1.00 [0.85; 1.18]	0.98 [0.83; 1.15]	-0.02 [-0.16; 0.12]
Nepal¶	0.80 [0.75; 0.86]	1.00 [0.90; 1.12]	0.20 [0.09; 0.31]†	4.5 [3.1; 6.6]	4.5 [3.1; 6.6]	1.25 [1.17; 1.34]*	1.19 [1.06; 1.34]*	-0.06 [-0.19; 0.08]
Netherlands	1.25 [1.19; 1.31]	1.25 [1.15; 1.35]	0.00 [-0.12; 0.11]	0.6 [0.6; 0.6]	0.5 [0.5; 0.5]	0.96 [0.91; 1.01]	0.97 [0.89; 1.04]	0.01 [-0.08; 0.10]
New Zealand	1.27 [1.20; 1.36]	1.20 [1.09; 1.32]	-0.07 [-0.20; 0.05]	0.9 [0.8; 1.0]	0.7 [0.6; 0.8]	0.94 [0.88; 1.00]	1.00 [0.91; 1.10]	0.06 [-0.04; 0.17]
Nicaragua	1.08 [0.99; 1.17]	1.21 [1.08; 1.36]	0.13 [0.00; 0.27]†	2.0 [1.8; 2.4]	1.7 [1.4; 2.0]	0.98 [0.89; 1.07]	0.99 [0.88; 1.11]	0.01 [-0.10; 0.13]
Niger¶	0.97 [0.93; 1.01]	0.98 [0.90; 1.08]	0.02 [-0.07; 0.12]	58.6 [43.0; 79.7]	59.7 [43.8; 81.1]	1.04 [1.00; 1.09]*	1.02 [0.93; 1.12]	-0.02 [-0.12; 0.08]
Nigeria	1.03 [0.99; 1.08]	1.01 [0.94; 1.09]	-0.02 [-0.11; 0.06]	43.5 [29.1; 65.5]	43.0 [29.0; 64.9]	0.97 [0.92; 1.02]	1.00 [0.93; 1.08]	0.03 [-0.06; 0.12]
Niue	1.21 [1.03; 1.40]	1.18 [1.03; 1.40]	-0.02 [-0.14; 0.15]	4.1 [1.6; 14.7]	3.5 [1.3; 12.2]	1.00 [0.86; 1.16]	0.98 [0.82; 1.15]	-0.02 [-0.16; 0.11]
Norway	1.29 [1.20; 1.39]	1.23 [1.12; 1.37]	-0.06 [-0.19; 0.08]	0.4 [0.4; 0.5]	0.4 [0.3; 0.4]	0.93 [0.86; 1.00]*	0.97 [0.88; 1.07]	0.04 [-0.06; 0.15]
Oman	1.04 [0.88; 1.19]	1.13 [0.94; 1.30]	0.09 [-0.04; 0.21]	1.5 [1.3; 1.7]	1.4 [1.2; 1.6]	1.10 [0.96; 1.32]	1.06 [0.93; 1.28]	-0.04 [-0.17; 0.09]
Pakistan¶§	0.84 [0.79; 0.90]	0.93 [0.84; 1.04]	0.09 [-0.01; 0.19]	10.7 [7.5; 15.2]	11.5 [8.0; 16.3]	1.20 [1.12; 1.29]*	1.19 [1.06; 1.35]*	-0.01 [-0.15; 0.14]
Palau	1.21 [1.04; 1.39]	1.20 [1.04; 1.40]	0.00 [-0.14; 0.15]	1.2 [0.6; 2.7]	1.0 [0.5; 2.2]	0.98 [0.85; 1.14]	1.00 [0.86; 1.15]	0.01 [-0.11; 0.13]
Panama	1.19 [1.05; 1.37]	1.21 [1.05; 1.38]	0.02 [-0.13; 0.15]	2.1 [1.1; 5.1]	1.8 [0.9; 4.2]	0.99 [0.86; 1.13]	0.99 [0.87; 1.14]	0.00 [-0.11; 0.12]
Papua New Guinea	1.04 [0.92; 1.18]	1.13 [1.00; 1.29]	0.09 [-0.03; 0.22]	9.2 [5.1; 16.4]	8.1 [4.5; 14.5]	0.99 [0.87; 1.12]	0.99 [0.86; 1.15]	0.01 [-0.12; 0.14]
Paraguay	1.11 [0.99; 1.25]	1.21 [1.06; 1.37]	0.11 [-0.04; 0.24]	2.9 [1.4; 8.0]	2.4 [1.2; 6.7]	1.01 [0.89; 1.14]	0.99 [0.87; 1.13]	-0.02 [-0.14; 0.11]
Peru	0.97 [0.92; 1.03]	1.20 [1.09; 1.33]	0.23 [0.12; 0.36]†	3.5 [2.8; 4.5]	2.9 [2.3; 3.7]	1.05 [1.00; 1.12]	1.00 [0.91; 1.10]	-0.06 [-0.16; 0.05]
Philippines	1.05 [0.99; 1.13]	1.19 [1.07; 1.32]	0.13 [0.01; 0.26]†	5.8 [4.1; 9.6]	4.9 [3.4; 8.0]	1.00 [0.93; 1.07]	0.99 [0.88; 1.11]	0.00 [-0.12; 0.11]
Poland	1.26 [1.22; 1.31]	1.25 [1.17; 1.33]	-0.01 [-0.10; 0.08]	0.7 [0.7; 0.7]	0.6 [0.5; 0.6]	0.95 [0.92; 0.99]*	0.96 [0.90; 1.03]	0.01 [-0.06; 0.09]
Portugal	1.30 [1.24; 1.36]	1.25 [1.14; 1.36]	-0.05 [-0.17; 0.07]	0.6 [0.6; 0.7]	0.5 [0.4; 0.5]	0.92 [0.88; 0.97]*	0.96 [0.89; 1.05]	0.04 [-0.05; 0.14]
Qatar	1.15 [1.02; 1.29]	1.15 [1.02; 1.30]	-0.01 [-0.12; 0.14]	0.8 [0.7; 0.9]	0.7 [0.6; 0.8]	1.04 [0.93; 1.18]	1.04 [0.93; 1.18]	0.00 [-0.12; 0.11]
Romania	1.16 [1.13; 1.20]	1.15 [1.08; 1.23]	-0.01 [-0.09; 0.07]	1.3 [1.2; 1.3]	1.1 [1.0; 1.2]	0.99 [0.97; 1.02]	1.04 [0.98; 1.11]	0.05 [-0.03; 0.12]
Russian Federation	1.30 [1.27; 1.33]	1.23 [1.17; 1.29]	-0.07 [-0.14; -0.01]†	1.1 [1.0; 1.1]	0.9 [0.8; 0.9]	0.92 [0.90; 0.94]*	0.98 [0.93; 1.03]	0.06 [0.01; 0.11]†
Rwanda¶	1.09 [1.03; 1.15]	1.12 [1.02; 1.25]	0.03 [-0.08; 0.16]	10.6 [5.3; 23.2]	9.5 [4.8; 20.0]	0.92 [0.87; 0.97]*	0.98 [0.86; 1.12]	0.06 [-0.06; 0.20]
Saint Kitts and Nevis	1.18 [1.03; 1.36]	1.19 [1.03; 1.38]	0.01 [-0.13; 0.15]	2.5 [1.7; 3.9]	2.1 [1.4; 3.3]	1.01 [0.87; 1.17]	1.00 [0.87; 1.17]	0.00 [-0.12; 0.12]
Saint Lucia	1.20 [1.08; 1.33]	1.20 [1.06; 1.34]	0.00 [-0.14; 0.13]	2.7 [2.2; 3.1]	2.2 [1.9; 2.6]	1.01 [0.90; 1.11]	1.01 [0.90; 1.13]	0.00 [-0.11; 0.12]
Samoa	1.16 [1.02; 1.32]	1.16 [1.02; 1.32]	0.00 [-0.13; 0.13]	2.6 [1.7; 4.0]	2.2 [1.5; 3.5]	1.03 [0.90; 1.17]	1.03 [0.91; 1.18]	0.00 [-0.12; 0.12]
San Marino	1.32 [1.18; 1.51]	1.32 [1.15; 1.51]	0.00 [-0.17; 0.13]	0.2 [0.1; 0.4]	0.2 [0.1; 0.3]	0.91 [0.80; 1.02]	0.92 [0.80; 1.04]	0.01 [-0.09; 0.12]
Sao Tome and Principe	0.99 [0.88; 1.11]	1.19 [1.04; 1.34]	0.20 [0.06; 0.33]†	3.6 [2.1; 6.1]	3.0 [1.8; 5.2]	1.02 [0.91; 1.15]	1.02 [0.89; 1.16]	0.00 [-0.12; 0.12]
Saudi Arabia	0.96 [0.79; 1.12]	1.04 [0.85; 1.20]	0.08 [-0.05; 0.19]	1.0 [0.7; 1.4]	1.0 [0.7; 1.3]	1.17 [1.00; 1.45]	1.16 [1.00; 1.41]*	-0.01 [-0.17; 0.13]
Senegal¶	1.05 [1.01; 1.10]	1.10 [1.01; 1.21]	0.04 [-0.06; 0.16]	10.2 [5.9; 16.8]	9.3 [5.4; 15.2]	0.95 [0.91; 1.00]*	1.01 [0.91; 1.13]	0.06 [-0.05; 0.18]
Serbia	1.09 [1.04; 1.14]	1.13 [1.04; 1.25]	0.04 [-0.06; 0.16]	0.8 [0.7; 0.9]	0.7 [0.6; 0.8]	1.10 [1.05; 1.15]*	1.06 [0.96; 1.16]	-0.04 [-0.14; 0.06]
Seychelles	1.17 [1.04; 1.31]	1.18 [1.04; 1.34]	0.01 [-0.12; 0.15]	2.1 [1.6; 2.9]	1.8 [1.4; 2.4]	1.02 [0.91; 1.16]	1.01 [0.90; 1.16]	-0.01 [-0.13; 0.11]
Sierra Leone	1.05 [0.98; 1.14]	1.05 [0.96; 1.16]	0.00 [-0.11; 0.12]	29.5 [20.5; 42.2]	27.9 [19.7; 39.5]	0.95 [0.89; 1.02]	0.96 [0.88; 1.06]	0.01 [-0.09; 0.12]
Singapore	1.13 [1.05; 1.21]	1.15 [1.04; 1.28]	0.02 [-0.10; 0.16]	0.4 [0.3; 0.5]	0.3 [0.3; 0.4]	1.06 [0.99; 1.14]	1.04 [0.94; 1.15]	-0.02 [-0.13; 0.09]
Slovakia	1.17 [1.10; 1.25]	1.22 [1.11; 1.32]	0.04 [-0.07; 0.16]	1.1 [1.0; 1.2]	0.9 [0.8; 1.0]	1.02 [0.96; 1.09]	0.99 [0.91; 1.08]	-0.04 [-0.13; 0.06]
Slovenia	1.21 [1.09; 1.33]	1.18 [1.06; 1.32]	-0.03 [-0.15; 0.10]	0.5 [0.4; 0.5]	0.4 [0.3; 0.5]	0.99 [0.91; 1.10]	1.02 [0.91; 1.14]	0.02 [-0.09; 0.13]
Solomon Islands	1.15 [1.00; 1.32]	1.20 [1.04; 1.37]	0.05 [-0.09; 0.19]	3.0 [1.8; 5.5]	2.5 [1.5; 4.6]	1.00 [0.87; 1.17]	1.00 [0.87; 1.16]	0.00 [-0.13; 0.12]
Somalia	1.03 [0.92; 1.16]	1.02 [0.90; 1.16]	-0.01 [-0.13; 0.11]	43.9 [14.9; 120.3]	43.1 [14.5; 117.1]	0.97 [0.87; 1.09]	0.99 [0.87; 1.14]	0.02 [-0.10; 0.15]
South Africa	1.12 [1.03; 1.22]	1.18 [1.11; 1.26]	0.06 [-0.05; 0.17]	7.1 [6.3; 8.1]	6.0 [5.3; 6.8]	0.95 [0.87; 1.03]	0.98 [0.92; 1.05]	0.03 [-0.07; 0.13]
South Sudan	0.98 [0.85; 1.12]	0.99 [0.85; 1.14]	0.02 [-0.11; 0.13]	37.3 [8.1; 107.5]	37.6 [8.3; 109.6]	1.03 [0.90; 1.19]	1.04 [0.89; 1.24]	0.01 [-0.12; 0.17]
Spain	1.22 [1.17; 1.27]	1.20 [1.13; 1.29]	-0.02 [-0.12; 0.08]	0.5 [0.5; 0.6]	0.4 [0.4; 0.5]	0.98 [0.94; 1.02]	1.00 [0.93; 1.07]	0.02 [-0.06; 0.10]
Sri Lanka	1.00 [0.97; 1.04]	1.18 [1.09; 1.26]	0.17 [0.08; 0.26]†	1.1 [0.8; 1.4]	0.9 [0.7; 1.2]	1.20 [1.16; 1.24]*	1.03 [0.95; 1.11]	-0.17 [-0.26; -0.08]†
Saint Vincent and the Grenadines	1.19 [1.08; 1.32]	1.19 [1.06; 1.34]	0.00 [-0.13; 0.14]	1.3 [0.9; 1.7]	1.1 [0.8; 1.5]	1.01 [0.91; 1.12]	1.00 [0.89; 1.13]	0.00 [-0.12; 0.12]
State of Palestine	1.13 [1.03; 1.24]	1.26 [1.14; 1.40]	0.13 [0.00; 0.27]†	2.3 [1.7; 3.4]	1.9 [1.3; 2.7]	0.99 [0.90; 1.09]	0.95 [0.85; 1.06]	-0.04 [-0.15; 0.07]
Sudan	1.01 [0.94; 1.08]	1.06 [0.95; 1.19]	0.05 [-0.06; 0.18]	17.1 [9.2; 30.0]	16.1 [8.9; 27.3]	1.00 [0.93; 1.08]	0.99 [0.88; 1.14]	0.00 [-0.12; 0.14]
Suriname	1.15 [1.00; 1.35]	1.20 [1.03; 1.39]	0.05 [-0.10; 0.18]	2.0 [1.3; 3.6]	1.7 [1.1; 3.0]	1.01 [0.86; 1.18]	1.00 [0.86; 1.16]	-0.01 [-0.13; 0.12]

¶: Sex ratio is outlying in 1990. §: Sex ratio is outlying in 2021. *: The ratio of estimated to expected female mortality is significantly different from one. †: Change is significantly different from zero.

Continued on next page

Table 14 – continued from previous page

	Sex ratio CMR			Sex-specific CMR in 2021 (per 1,000)		Estimated/Expected female CMR		
	1990	2021	Change 1990–2021	Male	Female	1990	2021	Change 1990–2021
Eswatini	1.05 [0.94; 1.18]	1.10 [0.96; 1.23]	0.04 [-0.10; 0.17]	12.1 [5.7; 25.6]	11.0 [5.4; 22.7]	1.00 [0.89; 1.12]	1.00 [0.86; 1.18]	0.01 [-0.14; 0.17]
Sweden	1.22 [1.14; 1.30]	1.18 [1.08; 1.29]	-0.04 [-0.16; 0.08]	0.5 [0.4; 0.5]	0.4 [0.4; 0.4]	0.99 [0.92; 1.05]	1.02 [0.93; 1.11]	0.04 [-0.07; 0.14]
Switzerland	1.27 [1.19; 1.35]	1.22 [1.12; 1.34]	-0.05 [-0.17; 0.08]	0.5 [0.5; 0.5]	0.4 [0.4; 0.4]	0.95 [0.89; 1.01]	0.98 [0.90; 1.07]	0.03 [-0.06; 0.13]
Syria	1.07 [0.97; 1.18]	1.10 [0.99; 1.23]	0.03 [-0.08; 0.15]	4.1 [2.0; 6.4]	3.7 [1.8; 5.7]	1.08 [0.97; 1.22]	1.04 [0.94; 1.17]	-0.04 [-0.16; 0.07]
Tajikistan	1.03 [0.88; 1.16]	1.21 [1.04; 1.37]	0.18 [0.04; 0.31]†	4.2 [2.2; 9.6]	3.5 [1.8; 8.0]	1.00 [0.88; 1.18]	0.99 [0.86; 1.16]	-0.01 [-0.14; 0.11]
Tanzania	1.06 [1.00; 1.13]	1.11 [1.00; 1.22]	0.04 [-0.07; 0.16]	14.1 [7.9; 25.7]	12.8 [7.2; 23.0]	0.94 [0.89; 1.00]	0.98 [0.87; 1.10]	0.03 [-0.08; 0.16]
Thailand	1.15 [1.03; 1.28]	1.19 [1.05; 1.35]	0.04 [-0.10; 0.18]	1.3 [1.1; 1.7]	1.1 [0.9; 1.4]	1.01 [0.90; 1.13]	1.01 [0.89; 1.15]	0.00 [-0.11; 0.13]
Timor Leste	1.01 [0.91; 1.12]	1.13 [1.01; 1.28]	0.11 [0.01; 0.26]†	8.3 [4.5; 15.8]	7.4 [3.9; 13.8]	1.00 [0.89; 1.11]	1.00 [0.87; 1.15]	0.00 [-0.13; 0.14]
Togo	1.07 [1.00; 1.14]	1.08 [0.99; 1.19]	0.02 [-0.10; 0.13]	20.9 [13.6; 30.9]	19.3 [12.5; 28.6]	0.94 [0.88; 1.00]	0.96 [0.86; 1.07]	0.02 [-0.08; 0.13]
Tonga	1.26 [1.10; 1.45]	1.25 [1.10; 1.45]	-0.01 [-0.15; 0.15]	1.8 [1.0; 3.2]	1.4 [0.8; 2.5]	0.96 [0.83; 1.09]	0.96 [0.83; 1.09]	0.00 [-0.12; 0.11]
Trinidad and Tobago	1.11 [0.95; 1.27]	1.11 [0.96; 1.30]	0.00 [-0.11; 0.15]	1.9 [0.8; 6.1]	1.7 [0.7; 5.5]	1.08 [0.95; 1.27]	1.06 [0.92; 1.25]	-0.02 [-0.15; 0.11]
Tunisia¶	0.97 [0.89; 1.06]	1.12 [1.03; 1.21]	0.14 [0.03; 0.25]†	2.4 [2.0; 3.0]	2.2 [1.8; 2.7]	1.12 [1.02; 1.24]*	1.07 [0.99; 1.17]	-0.05 [-0.17; 0.07]
Turkey¶	0.95 [0.88; 1.03]	1.12 [1.06; 1.18]	0.16 [0.07; 0.26]†	1.4 [1.2; 1.6]	1.2 [1.1; 1.4]	1.10 [1.01; 1.19]*	1.08 [1.02; 1.13]*	-0.02 [-0.13; 0.08]
Turkmenistan	1.12 [0.98; 1.27]	1.24 [1.08; 1.43]	0.12 [-0.02; 0.26]	6.4 [3.5; 12.2]	5.1 [2.8; 9.8]	0.95 [0.83; 1.08]	0.95 [0.80; 1.09]	0.00 [-0.13; 0.12]
Turks and Caicos Islands	1.25 [1.16; 1.35]	1.77 [1.56; 1.90]	0.52 [0.31; 0.65]†	0.8 [0.5; 1.4]	0.5 [0.3; 0.8]	0.96 [0.89; 1.04]	0.70 [0.63; 0.77]*	-0.26 [-0.35; -0.18]†
Tuvalu	0.92 [0.68; 1.11]	1.03 [0.75; 1.24]	0.11 [-0.01; 0.23]	3.2 [1.7; 7.2]	3.1 [1.6; 7.0]	1.16 [0.98; 1.66]	1.13 [0.96; 1.60]	-0.03 [-0.19; 0.12]
Uganda¶§	1.16 [1.10; 1.23]	1.26 [1.13; 1.37]	0.10 [-0.03; 0.22]	12.6 [7.4; 21.4]	10.0 [5.9; 17.1]	0.87 [0.82; 0.92]*	0.88 [0.78; 0.98]*	0.01 [-0.10; 0.12]
Ukraine	1.13 [0.98; 1.31]	1.15 [0.98; 1.33]	0.02 [-0.12; 0.15]	1.3 [1.2; 1.4]	1.1 [1.0; 1.2]	1.05 [0.92; 1.22]	1.04 [0.91; 1.22]	-0.01 [-0.14; 0.11]
United Arab Emirates	1.21 [1.09; 1.35]	1.21 [1.10; 1.34]	0.00 [-0.14; 0.14]	1.0 [0.9; 1.2]	0.8 [0.7; 1.0]	1.00 [0.89; 1.11]	0.99 [0.90; 1.10]	0.00 [-0.12; 0.11]
United Kingdom	1.21 [1.17; 1.25]	1.17 [1.10; 1.24]	-0.04 [-0.13; 0.05]	0.6 [0.5; 0.6]	0.5 [0.5; 0.5]	0.99 [0.96; 1.03]	1.03 [0.97; 1.10]	0.04 [-0.04; 0.11]
United States of America	1.25 [1.22; 1.28]	1.25 [1.19; 1.32]	0.00 [-0.06; 0.07]	1.0 [0.9; 1.0]	0.8 [0.7; 0.8]	0.96 [0.94; 0.98]*	0.96 [0.91; 1.01]	0.00 [-0.05; 0.05]
Uruguay	1.14 [1.08; 1.21]	1.16 [1.06; 1.26]	0.02 [-0.09; 0.13]	0.9 [0.8; 1.0]	0.8 [0.7; 0.9]	1.05 [0.99; 1.12]	1.04 [0.95; 1.13]	-0.02 [-0.12; 0.09]
Uzbekistan	1.09 [0.95; 1.24]	1.20 [1.05; 1.38]	0.11 [-0.01; 0.26]	1.6 [1.5; 1.9]	1.4 [1.2; 1.6]	1.00 [0.87; 1.14]	1.00 [0.87; 1.15]	0.00 [-0.12; 0.12]
Vanuatu	1.14 [0.98; 1.31]	1.19 [1.02; 1.36]	0.04 [-0.09; 0.18]	3.9 [2.0; 9.1]	3.3 [1.7; 7.6]	1.02 [0.88; 1.19]	1.01 [0.87; 1.17]	-0.01 [-0.15; 0.12]
Venezuela (Bolivarian Republic of)	1.11 [1.07; 1.15]	1.11 [1.03; 1.21]	0.00 [-0.09; 0.11]	3.4 [2.4; 5.1]	3.1 [2.2; 4.6]	1.08 [1.04; 1.11]*	1.05 [0.96; 1.14]	-0.03 [-0.12; 0.07]
Vietnam¶§	1.52 [1.37; 1.69]	1.97 [1.73; 2.22]	0.45 [0.23; 0.66]†	5.6 [5.0; 6.3]	2.8 [2.5; 3.3]	0.69 [0.62; 0.77]*	0.60 [0.54; 0.69]*	-0.08 [-0.16; -0.01]†
Yemen¶	0.93 [0.87; 0.98]	1.01 [0.89; 1.13]	0.08 [-0.04; 0.20]	16.1 [4.9; 47.5]	16.0 [5.3; 44.5]	1.09 [1.02; 1.16]*	1.06 [0.92; 1.25]	-0.03 [-0.17; 0.16]
Zambia¶	1.06 [1.01; 1.11]	1.16 [1.06; 1.27]	0.10 [-0.01; 0.22]	19.5 [11.6; 31.3]	16.9 [10.0; 27.2]	0.95 [0.90; 1.00]*	0.91 [0.82; 1.00]	-0.04 [-0.14; 0.06]
Zimbabwe	1.04 [0.97; 1.13]	1.10 [1.00; 1.22]	0.06 [-0.06; 0.18]	15.0 [8.6; 26.0]	13.6 [7.8; 23.6]	0.97 [0.90; 1.05]	0.97 [0.87; 1.09]	0.00 [-0.11; 0.13]

Table 15: **Estimates and 90% uncertainty intervals for sex ratios for U5MR in 1990 and 2021, the change in sex ratios from 1990 to 2021, sex-specific U5MR in 2021, and ratios of estimated to expected female U5MR and their change from 1990 to 2021 for the world, UNICEF regions, and all countries.** ¶: Sex ratio is outlying in 1990. §: Sex ratio is outlying in 2021. *: The ratio of estimated to expected female mortality is significantly different from one. †: Change is significantly different from zero.

	Sex ratio U5MR			Sex-specific U5MR in 2021 (per 1,000)		Estimated/Expected female U5MR		
	1990	2021	Change 1990–2021	Male	Female	1990	2021	Change 1990–2021
World¶§	1.06 [1.05; 1.07]	1.13 [1.11; 1.15]	0.07 [0.05; 0.09]†	40.3 [37.4; 43.8]	35.7 [33.1; 39.0]	1.06 [1.05; 1.07]*	1.03 [1.01; 1.05]*	-0.03 [-0.06; -0.01]†
South Asia¶§	0.97 [0.95; 0.99]	1.05 [1.01; 1.09]	0.08 [0.04; 0.12]†	37.9 [34.6; 41.7]	36.1 [33.0; 39.8]	1.16 [1.13; 1.18]*	1.16 [1.11; 1.20]*	0.00 [-0.05; 0.05]
Europe and Central Asia	1.21 [1.19; 1.23]	1.24 [1.21; 1.27]	0.03 [0.00; 0.06]†	8.4 [7.9; 9.3]	6.8 [6.4; 7.5]	0.99 [0.97; 1.01]	0.98 [0.96; 1.00]	-0.01 [-0.03; 0.02]
Middle East and North Africa¶	1.07 [1.05; 1.08]	1.16 [1.12; 1.20]	0.10 [0.05; 0.14]†	23.5 [19.0; 32.2]	20.3 [16.4; 27.7]	1.10 [1.08; 1.12]*	1.05 [1.00; 1.09]	-0.05 [-0.10; -0.01]†
Sub-Saharan Africa¶	1.11 [1.10; 1.12]	1.16 [1.13; 1.18]	0.05 [0.02; 0.08]†	78.2 [71.6; 91.8]	67.5 [61.5; 79.7]	0.98 [0.97; 0.99]*	0.98 [0.96; 1.01]	0.00 [-0.02; 0.03]
Latin America and Caribbean	1.18 [1.15; 1.22]	1.22 [1.19; 1.26]	0.04 [0.00; 0.08]	17.5 [16.3; 19.2]	14.3 [13.3; 15.7]	1.00 [0.97; 1.03]	1.00 [0.97; 1.03]	0.00 [-0.03; 0.03]
East Asia and Pacific¶	1.12 [1.08; 1.17]	1.20 [1.16; 1.24]	0.08 [0.02; 0.13]†	16.0 [14.2; 17.7]	13.3 [11.8; 14.7]	1.05 [1.01; 1.09]*	1.01 [0.97; 1.04]	-0.04 [-0.09; 0.01]
North America	1.25 [1.24; 1.27]	1.20 [1.16; 1.24]	-0.05 [-0.09; -0.01]†	6.7 [6.3; 7.0]	5.6 [5.3; 5.9]	0.97 [0.96; 0.99]*	0.99 [0.96; 1.03]	0.02 [-0.01; 0.05]
Afghanistan	1.04 [1.00; 1.09]	1.13 [1.06; 1.21]	0.09 [0.00; 0.18]†	59.1 [43.4; 78.9]	52.2 [38.2; 69.6]	1.05 [1.00; 1.10]	1.05 [0.98; 1.13]	0.00 [-0.08; 0.09]
Albania	1.24 [1.15; 1.32]	1.17 [1.09; 1.24]	-0.07 [-0.18; 0.03]	10.2 [9.3; 11.0]	8.7 [8.0; 9.5]	0.99 [0.92; 1.06]	1.05 [0.99; 1.11]	0.06 [-0.04; 0.15]
Algeria§	1.17 [1.13; 1.22]	1.16 [1.09; 1.24]	-0.01 [-0.09; 0.08]	24.0 [21.1; 27.3]	20.6 [18.1; 23.5]	1.03 [0.99; 1.07]	1.06 [1.00; 1.13]*	0.03 [-0.04; 0.11]
Andorra¶	1.43 [1.31; 1.57]	1.29 [1.16; 1.45]	-0.15 [-0.28; 0.00]†	3.1 [0.8; 11.6]	2.4 [0.6; 9.1]	0.84 [0.76; 0.92]*	0.93 [0.82; 1.04]	0.10 [0.00; 0.20]
Angola	1.11 [1.06; 1.15]	1.19 [1.11; 1.28]	0.08 [-0.01; 0.17]	75.2 [31.7; 148.7]	63.3 [26.9; 125.2]	0.97 [0.93; 1.01]	0.97 [0.89; 1.06]	0.00 [-0.08; 0.09]
Anguilla¶	1.09 [1.03; 1.16]	0.99 [0.90; 1.08]	-0.11 [-0.20; -0.01]†	4.0 [1.9; 8.4]	4.1 [1.9; 8.6]	1.13 [1.06; 1.20]*	1.21 [1.10; 1.33]*	0.08 [-0.03; 0.20]
Antigua and Barbuda	1.19 [1.10; 1.30]	1.17 [1.06; 1.29]	-0.02 [-0.14; 0.10]	6.6 [4.3; 10.2]	5.6 [3.7; 8.8]	1.03 [0.95; 1.12]	1.02 [0.93; 1.12]	-0.01 [-0.10; 0.10]
Argentina	1.24 [1.22; 1.26]	1.29 [1.24; 1.33]	0.05 [0.00; 0.10]†	7.8 [7.4; 8.2]	6.0 [5.7; 6.4]	1.00 [0.98; 1.02]	0.93 [0.90; 0.97]*	-0.07 [-0.10; -0.03]†
Armenia	1.23 [1.14; 1.32]	1.22 [1.11; 1.34]	0.00 [-0.12; 0.12]	11.8 [9.2; 14.8]	9.6 [7.5; 12.1]	0.99 [0.91; 1.06]	1.00 [0.91; 1.10]	0.02 [-0.08; 0.11]
Australia	1.28 [1.24; 1.31]	1.20 [1.14; 1.25]	-0.08 [-0.14; -0.01]†	4.0 [3.8; 4.3]	3.4 [3.2; 3.6]	0.95 [0.93; 0.98]*	0.99 [0.95; 1.04]	0.04 [-0.01; 0.09]
Austria	1.26 [1.21; 1.30]	1.20 [1.14; 1.28]	-0.05 [-0.13; 0.03]	4.0 [3.6; 4.5]	3.3 [3.0; 3.7]	0.97 [0.93; 1.01]	0.99 [0.93; 1.05]	0.02 [-0.05; 0.09]
Azerbaijan	1.13 [1.07; 1.20]	1.21 [1.11; 1.33]	0.08 [-0.03; 0.20]	20.3 [12.1; 34.5]	16.8 [9.9; 28.7]	1.02 [0.96; 1.08]	1.02 [0.93; 1.12]	0.00 [-0.09; 0.11]
Bahamas¶	1.16 [1.10; 1.23]	1.17 [1.08; 1.26]	0.01 [-0.09; 0.11]	14.2 [12.1; 16.5]	12.1 [10.3; 14.2]	1.07 [1.01; 1.13]*	1.05 [0.98; 1.14]	-0.01 [-0.10; 0.08]
Bahrain¶	1.06 [1.01; 1.11]	1.10 [1.02; 1.17]	0.04 [-0.05; 0.12]	7.2 [6.2; 8.5]	6.6 [5.6; 7.8]	1.17 [1.12; 1.23]*	1.09 [1.02; 1.17]*	-0.08 [-0.16; 0.01]
Bangladesh¶§	1.05 [1.02; 1.08]	1.16 [1.10; 1.23]	0.11 [0.04; 0.19]†	29.2 [26.3; 32.4]	25.2 [22.6; 28.0]	1.06 [1.03; 1.09]*	1.06 [1.01; 1.13]*	0.01 [-0.06; 0.08]
Barbados	1.21 [1.13; 1.29]	1.18 [1.08; 1.30]	-0.02 [-0.14; 0.10]	12.9 [8.5; 19.8]	10.9 [7.1; 16.8]	1.03 [0.96; 1.10]	1.04 [0.95; 1.14]	0.01 [-0.09; 0.12]
Belarus¶	1.35 [1.31; 1.39]	1.27 [1.20; 1.35]	-0.08 [-0.16; 0.01]	3.0 [2.6; 3.5]	2.4 [2.0; 2.8]	0.91 [0.89; 0.94]*	0.93 [0.88; 0.99]*	0.02 [-0.04; 0.08]
Belgium	1.32 [1.27; 1.36]	1.26 [1.19; 1.33]	-0.05 [-0.14; 0.03]	4.6 [4.1; 5.1]	3.6 [3.2; 4.0]	0.93 [0.90; 0.96]*	0.94 [0.89; 1.00]*	0.01 [-0.04; 0.07]
Belize	1.22 [1.13; 1.31]	1.20 [1.11; 1.29]	-0.02 [-0.13; 0.10]	12.2 [9.8; 15.0]	10.2 [8.2; 12.5]	1.00 [0.93; 1.08]	1.02 [0.95; 1.10]	0.02 [-0.08; 0.12]
Benin	1.09 [1.05; 1.13]	1.16 [1.09; 1.23]	0.07 [-0.01; 0.15]	89.4 [72.6; 110.9]	77.4 [62.6; 95.6]	1.00 [0.97; 1.04]	0.98 [0.92; 1.04]	-0.02 [-0.09; 0.05]
Bhutan	1.08 [1.01; 1.15]	1.20 [1.10; 1.32]	0.12 [0.02; 0.24]†	29.0 [17.2; 48.5]	24.2 [14.2; 40.7]	1.04 [0.97; 1.11]	1.02 [0.93; 1.12]	-0.02 [-0.12; 0.08]
Bolivia (Plurinational State of)	1.11 [1.07; 1.15]	1.21 [1.12; 1.31]	0.10 [0.01; 0.21]†	27.0 [18.9; 38.5]	22.3 [15.6; 31.9]	1.01 [0.97; 1.05]	1.02 [0.94; 1.10]	0.01 [-0.07; 0.10]
Bosnia and Herzegovina	1.25 [1.20; 1.30]	1.22 [1.14; 1.31]	-0.03 [-0.12; 0.07]	6.1 [4.8; 7.9]	5.0 [3.9; 6.4]	0.99 [0.95; 1.03]	0.98 [0.91; 1.05]	-0.01 [-0.09; 0.06]
Botswana	1.21 [1.12; 1.30]	1.22 [1.11; 1.32]	0.01 [-0.11; 0.12]	38.2 [11.6; 127.5]	31.4 [9.4; 103.9]	0.99 [0.92; 1.06]	1.00 [0.90; 1.09]	0.01 [-0.10; 0.11]
Brazil	1.21 [1.13; 1.29]	1.25 [1.14; 1.38]	0.04 [-0.07; 0.17]	16.0 [13.5; 18.8]	12.7 [10.8; 15.1]	0.98 [0.92; 1.05]	0.99 [0.90; 1.08]	0.00 [-0.09; 0.10]
British Virgin Islands	1.26 [1.19; 1.33]	1.20 [1.10; 1.31]	-0.06 [-0.18; 0.06]	11.4 [5.1; 25.0]	9.5 [4.3; 20.6]	0.99 [0.94; 1.04]	1.02 [0.93; 1.12]	0.03 [-0.06; 0.14]
Brunei	1.19 [1.12; 1.26]	1.20 [1.11; 1.29]	0.01 [-0.09; 0.12]	12.5 [10.4; 15.0]	10.4 [8.7; 12.5]	1.03 [0.97; 1.09]	1.02 [0.95; 1.10]	-0.01 [-0.10; 0.08]
Bulgaria	1.27 [1.24; 1.31]	1.17 [1.12; 1.24]	-0.10 [-0.17; -0.03]†	6.8 [6.3; 7.3]	5.8 [5.4; 6.2]	0.97 [0.94; 1.00]*	1.02 [0.97; 1.07]	0.05 [-0.01; 0.10]
Burkina Faso	1.08 [1.04; 1.11]	1.12 [1.06; 1.20]	0.05 [-0.03; 0.12]	87.4 [59.6; 129.1]	77.7 [53.0; 115.1]	0.99 [0.96; 1.03]	1.01 [0.94; 1.08]	0.01 [-0.06; 0.09]
Burundi	1.10 [1.05; 1.16]	1.19 [1.11; 1.28]	0.09 [0.00; 0.19]†	57.2 [35.1; 92.6]	47.9 [29.3; 77.5]	0.99 [0.94; 1.04]	0.98 [0.91; 1.06]	0.00 [-0.09; 0.08]
Cambodia	1.15 [1.11; 1.20]	1.26 [1.16; 1.36]	0.11 [0.00; 0.22]†	27.6 [13.5; 56.4]	21.8 [10.8; 44.5]	0.98 [0.94; 1.02]	0.98 [0.90; 1.06]	0.00 [-0.09; 0.09]
Cameroon	1.13 [1.08; 1.17]	1.18 [1.11; 1.26]	0.05 [-0.03; 0.14]	75.4 [58.0; 96.7]	63.8 [48.9; 82.0]	0.98 [0.94; 1.02]	0.97 [0.91; 1.04]	-0.01 [-0.08; 0.07]
Canada	1.25 [1.22; 1.28]	1.16 [1.11; 1.21]	-0.09 [-0.14; -0.03]†	5.4 [5.1; 5.7]	4.7 [4.4; 4.9]	0.97 [0.95; 1.00]*	1.02 [0.98; 1.07]	0.05 [0.00; 0.10]†

¶: Sex ratio is outlying in 1990. §: Sex ratio is outlying in 2021. *: The ratio of estimated to expected female mortality is significantly different from one. †: Change is significantly different from zero.

Continued on next page

Table 15 – continued from previous page

	Sex ratio U5MR			Sex-specific U5MR in 2021 (per 1,000)		Estimated/Expected female U5MR		
	1990	2021	Change 1990–2021	Male	Female	1990	2021	Change 1990–2021
Cape Verde	1.15 [1.11; 1.20]	1.20 [1.12; 1.30]	0.05 [-0.04; 0.15]	14.8 [11.3; 19.3]	12.3 [9.4; 16.0]	1.02 [0.98; 1.07]	1.02 [0.95; 1.10]	0.00 [-0.08; 0.08]
Central African Republic	1.10 [1.05; 1.14]	1.15 [1.08; 1.23]	0.05 [-0.03; 0.14]	106.7 [83.8; 135.3]	92.7 [72.8; 118.1]	1.00 [0.95; 1.04]	0.99 [0.92; 1.07]	0.00 [-0.08; 0.08]
Chad	1.10 [1.06; 1.14]	1.13 [1.06; 1.21]	0.04 [-0.04; 0.12]	113.7 [79.3; 161.2]	100.1 [69.9; 141.8]	0.98 [0.94; 1.01]	0.98 [0.92; 1.05]	0.01 [-0.06; 0.08]
Chile	1.22 [1.20; 1.25]	1.18 [1.12; 1.25]	-0.04 [-0.11; 0.04]	7.1 [5.9; 8.5]	6.0 [5.0; 7.1]	1.01 [0.99; 1.03]	1.01 [0.95; 1.07]	0.00 [-0.06; 0.06]
China¶	1.09 [1.02; 1.16]	1.12 [1.04; 1.21]	0.03 [-0.06; 0.13]	7.3 [6.6; 8.2]	6.5 [5.8; 7.3]	1.10 [1.03; 1.18]*	1.07 [0.99; 1.15]	-0.03 [-0.13; 0.06]
Colombia	1.25 [1.19; 1.31]	1.24 [1.14; 1.35]	-0.01 [-0.12; 0.11]	14.2 [9.3; 21.6]	11.5 [7.5; 17.5]	0.98 [0.93; 1.03]	0.99 [0.91; 1.08]	0.01 [-0.08; 0.10]
Comoros	1.09 [1.02; 1.17]	1.12 [1.02; 1.21]	0.03 [-0.07; 0.12]	52.4 [39.1; 70.4]	46.8 [34.8; 63.2]	1.02 [0.95; 1.10]	1.07 [0.98; 1.18]	0.05 [-0.04; 0.15]
Congo	1.13 [1.07; 1.20]	1.20 [1.12; 1.30]	0.07 [-0.03; 0.18]	46.8 [26.3; 84.5]	38.9 [21.9; 70.2]	1.00 [0.94; 1.06]	0.99 [0.91; 1.08]	-0.01 [-0.10; 0.09]
Democratic Republic of the Congo	1.09 [1.04; 1.13]	1.19 [1.10; 1.28]	0.10 [0.01; 0.20]†	85.5 [50.8; 142.7]	72.1 [42.9; 120.4]	1.00 [0.96; 1.05]	0.98 [0.90; 1.07]	-0.02 [-0.11; 0.08]
Cook Islands¶	1.10 [1.01; 1.20]	1.04 [0.94; 1.15]	-0.06 [-0.16; 0.04]	7.3 [4.0; 13.6]	7.0 [3.9; 13.1]	1.13 [1.03; 1.23]*	1.16 [1.05; 1.27]*	0.03 [-0.09; 0.14]
Costa Rica	1.27 [1.23; 1.31]	1.14 [1.09; 1.20]	-0.12 [-0.19; -0.05]†	8.1 [7.5; 8.8]	7.1 [6.6; 7.7]	0.98 [0.95; 1.01]	1.05 [1.00; 1.11]*	0.08 [0.02; 0.14]†
Cote d'Ivoire¶	1.17 [1.13; 1.23]	1.24 [1.15; 1.33]	0.06 [-0.03; 0.16]	82.5 [61.6; 110.4]	66.7 [49.7; 89.6]	0.94 [0.90; 0.98]*	0.93 [0.87; 1.01]	0.00 [-0.08; 0.08]
Croatia	1.30 [1.25; 1.35]	1.18 [1.11; 1.25]	-0.12 [-0.21; -0.03]†	5.0 [4.6; 5.5]	4.3 [3.9; 4.7]	0.95 [0.91; 0.99]*	1.01 [0.95; 1.07]	0.06 [-0.01; 0.13]
Cuba	1.31 [1.28; 1.35]	1.23 [1.17; 1.29]	-0.09 [-0.15; -0.01]†	5.5 [4.5; 7.0]	4.5 [3.6; 5.6]	0.94 [0.91; 0.96]*	0.97 [0.92; 1.02]	0.03 [-0.02; 0.09]
Cyprus	1.22 [1.14; 1.29]	1.18 [1.08; 1.27]	-0.04 [-0.14; 0.06]	3.0 [2.2; 4.3]	2.6 [1.9; 3.6]	1.01 [0.95; 1.07]	1.01 [0.93; 1.10]	0.00 [-0.08; 0.09]
Czech Republic	1.30 [1.24; 1.37]	1.28 [1.21; 1.35]	-0.02 [-0.11; 0.07]	3.1 [2.8; 3.3]	2.4 [2.2; 2.6]	0.95 [0.90; 0.99]*	0.93 [0.88; 0.98]*	-0.02 [-0.08; 0.05]
Denmark	1.28 [1.23; 1.33]	1.19 [1.12; 1.27]	-0.09 [-0.17; 0.00]†	3.9 [3.6; 4.2]	3.3 [3.0; 3.5]	0.95 [0.91; 0.98]*	1.00 [0.94; 1.06]	0.05 [-0.02; 0.12]
Djibouti	1.14 [1.06; 1.23]	1.20 [1.10; 1.31]	0.06 [-0.05; 0.17]	58.8 [32.4; 103.7]	49.0 [26.9; 87.3]	0.99 [0.91; 1.07]	1.00 [0.91; 1.10]	0.01 [-0.09; 0.11]
Dominica	1.16 [1.07; 1.24]	1.16 [1.05; 1.28]	0.01 [-0.11; 0.13]	38.5 [32.6; 45.5]	33.1 [27.9; 39.3]	1.06 [0.99; 1.15]	1.06 [0.96; 1.17]	-0.01 [-0.11; 0.10]
Dominican Republic	1.16 [1.11; 1.22]	1.19 [1.11; 1.29]	0.03 [-0.07; 0.14]	35.8 [26.4; 48.2]	30.0 [22.1; 40.4]	1.02 [0.97; 1.07]	1.03 [0.95; 1.11]	0.01 [-0.08; 0.10]
Ecuador	1.23 [1.15; 1.32]	1.25 [1.15; 1.36]	0.02 [-0.09; 0.13]	13.8 [12.1; 15.7]	11.1 [9.6; 12.6]	0.97 [0.90; 1.04]	0.98 [0.91; 1.07]	0.02 [-0.07; 0.11]
Egypt¶§	0.99 [0.96; 1.03]	1.14 [1.05; 1.24]	0.15 [0.05; 0.25]†	20.2 [13.0; 31.1]	17.7 [11.4; 27.3]	1.16 [1.12; 1.20]*	1.08 [1.00; 1.18]*	-0.08 [-0.17; 0.02]
El Salvador	1.17 [1.09; 1.25]	1.21 [1.11; 1.33]	0.05 [-0.07; 0.16]	13.6 [8.1; 23.2]	11.2 [6.7; 19.1]	1.01 [0.94; 1.09]	1.01 [0.92; 1.11]	0.00 [-0.10; 0.10]
Equatorial Guinea	1.11 [1.02; 1.20]	1.17 [1.07; 1.28]	0.07 [-0.04; 0.17]	82.6 [45.3; 147.4]	70.4 [38.3; 125.7]	0.99 [0.91; 1.07]	0.99 [0.90; 1.09]	0.00 [-0.09; 0.10]
Eritrea¶	1.21 [1.16; 1.26]	1.29 [1.19; 1.39]	0.08 [-0.03; 0.18]	42.7 [23.6; 77.3]	33.2 [18.2; 60.6]	0.91 [0.87; 0.95]*	0.94 [0.86; 1.02]	0.03 [-0.05; 0.11]
Estonia	1.30 [1.24; 1.36]	1.22 [1.13; 1.32]	-0.08 [-0.18; 0.03]	2.2 [1.8; 2.7]	1.8 [1.4; 2.2]	0.95 [0.91; 0.99]*	0.97 [0.90; 1.05]	0.02 [-0.06; 0.11]
Ethiopia¶	1.14 [1.10; 1.18]	1.27 [1.19; 1.37]	0.14 [0.04; 0.24]†	52.2 [40.6; 67.6]	41.0 [31.8; 53.1]	0.95 [0.91; 0.99]*	0.93 [0.86; 1.00]	-0.02 [-0.09; 0.06]
Federated States of Micronesia	1.28 [1.17; 1.39]	1.30 [1.19; 1.44]	0.03 [-0.09; 0.16]	28.0 [12.6; 61.5]	21.5 [9.6; 46.7]	0.94 [0.86; 1.02]	0.94 [0.85; 1.03]	0.00 [-0.10; 0.09]
Fiji¶	1.15 [1.08; 1.23]	1.18 [1.11; 1.26]	0.03 [-0.08; 0.13]	29.9 [26.7; 33.5]	25.4 [22.6; 28.5]	1.07 [1.00; 1.14]*	1.05 [0.98; 1.12]	-0.02 [-0.12; 0.07]
Finland	1.23 [1.18; 1.28]	1.19 [1.12; 1.27]	-0.04 [-0.13; 0.05]	2.4 [2.1; 2.6]	2.0 [1.8; 2.2]	0.97 [0.94; 1.01]	1.00 [0.93; 1.06]	0.02 [-0.05; 0.10]
France	1.34 [1.31; 1.37]	1.22 [1.17; 1.27]	-0.12 [-0.18; -0.06]†	4.8 [4.4; 5.2]	3.9 [3.6; 4.3]	0.91 [0.89; 0.93]*	0.97 [0.93; 1.01]	0.07 [0.02; 0.11]†
Gabon	1.17 [1.10; 1.25]	1.23 [1.13; 1.34]	0.06 [-0.04; 0.16]	43.7 [26.0; 74.0]	35.6 [21.4; 59.8]	0.96 [0.90; 1.03]	0.97 [0.89; 1.06]	0.01 [-0.08; 0.10]
The Gambia	1.12 [1.07; 1.18]	1.22 [1.14; 1.31]	0.10 [0.01; 0.20]†	52.6 [40.7; 67.6]	43.0 [33.2; 55.2]	0.96 [0.91; 1.01]	0.96 [0.90; 1.04]	0.00 [-0.07; 0.08]
Georgia	1.26 [1.16; 1.37]	1.25 [1.13; 1.38]	-0.01 [-0.13; 0.12]	10.5 [9.3; 11.8]	8.4 [7.4; 9.5]	0.96 [0.88; 1.05]	0.98 [0.89; 1.08]	0.01 [-0.08; 0.11]
Germany	1.30 [1.27; 1.32]	1.17 [1.13; 1.20]	-0.13 [-0.18; -0.08]†	3.8 [3.7; 4.0]	3.3 [3.2; 3.4]	0.93 [0.91; 0.95]*	1.02 [0.99; 1.05]	0.09 [0.05; 0.12]†
Ghana	1.13 [1.09; 1.18]	1.23 [1.16; 1.32]	0.10 [0.01; 0.19]†	48.4 [36.6; 64.7]	39.3 [29.6; 52.4]	0.97 [0.94; 1.01]	0.96 [0.90; 1.04]	-0.01 [-0.08; 0.07]
Greece	1.17 [1.14; 1.21]	1.17 [1.11; 1.24]	0.00 [-0.08; 0.08]	4.0 [3.5; 4.5]	3.4 [3.0; 3.9]	1.04 [1.01; 1.08]*	1.01 [0.96; 1.07]	-0.03 [-0.10; 0.04]
Grenada¶	1.14 [1.06; 1.22]	1.17 [1.07; 1.27]	0.03 [-0.08; 0.14]	17.4 [14.2; 21.4]	14.9 [12.1; 18.3]	1.08 [1.01; 1.16]*	1.06 [0.97; 1.16]	-0.03 [-0.12; 0.08]
Guatemala	1.16 [1.11; 1.21]	1.23 [1.14; 1.33]	0.08 [-0.02; 0.18]	25.3 [18.1; 35.0]	20.6 [14.6; 28.5]	1.00 [0.96; 1.04]	1.00 [0.93; 1.08]	0.00 [-0.08; 0.09]
Guinea	1.07 [1.04; 1.11]	1.17 [1.10; 1.24]	0.10 [0.02; 0.17]†	106.1 [86.2; 132.3]	91.0 [73.9; 114.0]	1.00 [0.97; 1.04]	0.96 [0.90; 1.02]	-0.04 [-0.11; 0.03]
Guinea-Bissau	1.10 [1.06; 1.15]	1.18 [1.10; 1.27]	0.08 [-0.01; 0.17]	80.2 [46.1; 132.5]	67.8 [39.2; 112.2]	0.97 [0.93; 1.02]	0.97 [0.89; 1.05]	-0.01 [-0.09; 0.08]
Guyana¶	1.27 [1.19; 1.35]	1.29 [1.18; 1.40]	0.02 [-0.10; 0.14]	31.0 [17.9; 54.0]	24.1 [14.0; 42.0]	0.93 [0.87; 0.99]*	0.95 [0.87; 1.04]	0.02 [-0.06; 0.12]
Haiti	1.13 [1.08; 1.18]	1.20 [1.11; 1.30]	0.07 [-0.02; 0.17]	63.9 [44.8; 92.6]	53.1 [37.0; 76.9]	0.98 [0.94; 1.02]	0.98 [0.90; 1.07]	0.00 [-0.08; 0.09]
Honduras	1.20 [1.14; 1.28]	1.24 [1.15; 1.35]	0.04 [-0.07; 0.15]	18.3 [13.9; 24.8]	14.7 [11.2; 19.9]	0.98 [0.92; 1.04]	0.99 [0.92; 1.08]	0.01 [-0.08; 0.10]
Hungary	1.24 [1.21; 1.28]	1.20 [1.13; 1.27]	-0.05 [-0.12; 0.03]	4.3 [3.8; 4.9]	3.6 [3.2; 4.1]	0.99 [0.97; 1.02]	0.99 [0.94; 1.05]	0.00 [-0.06; 0.06]
Iceland	1.17 [1.08; 1.26]	1.17 [1.07; 1.28]	0.00 [-0.11; 0.12]	2.8 [2.2; 3.7]	2.4 [1.9; 3.1]	1.02 [0.95; 1.10]	1.01 [0.93; 1.11]	-0.01 [-0.10; 0.09]

¶: Sex ratio is outlying in 1990. §: Sex ratio is outlying in 2021. *: The ratio of estimated to expected female mortality is significantly different from one. †: Change is significantly different from zero.

Continued on next page

Table 15 – continued from previous page

	Sex ratio U5MR			Sex-specific U5MR in 2021 (per 1,000)		Estimated/Expected female U5MR		
	1990	2021	Change 1990–2021	Male	Female	1990	2021	Change 1990–2021
India¶§	0.94 [0.92; 0.96]	0.98 [0.92; 1.04]	0.04 [-0.02; 0.10]	30.3 [27.2; 33.6]	31.0 [27.8; 34.4]	1.19 [1.17; 1.22]*	1.26 [1.19; 1.34]*	0.07 [-0.01; 0.15]
Indonesia	1.17 [1.12; 1.21]	1.23 [1.14; 1.33]	0.07 [-0.03; 0.17]	24.4 [19.1; 31.4]	19.8 [15.6; 25.5]	0.99 [0.95; 1.02]	1.01 [0.93; 1.09]	0.02 [-0.06; 0.10]
Iran (Islamic Republic of)¶§	1.01 [0.96; 1.06]	1.10 [1.01; 1.20]	0.09 [0.00; 0.20]	13.2 [7.4; 23.3]	12.0 [6.7; 21.2]	1.18 [1.12; 1.24]*	1.11 [1.02; 1.21]*	-0.07 [-0.17; 0.04]
Iraq	1.16 [1.10; 1.22]	1.21 [1.12; 1.32]	0.05 [-0.05; 0.17]	26.8 [19.5; 36.1]	22.1 [16.1; 29.8]	1.03 [0.97; 1.09]	1.02 [0.94; 1.10]	-0.01 [-0.10; 0.08]
Ireland	1.25 [1.20; 1.30]	1.19 [1.11; 1.27]	-0.06 [-0.15; 0.03]	3.4 [2.9; 3.9]	2.9 [2.5; 3.3]	0.98 [0.94; 1.01]	1.00 [0.94; 1.07]	0.02 [-0.05; 0.10]
Israel	1.13 [1.10; 1.17]	1.18 [1.13; 1.24]	0.05 [-0.01; 0.12]	3.6 [3.4; 3.8]	3.1 [2.9; 3.2]	1.08 [1.05; 1.11]*	1.00 [0.96; 1.05]	-0.08 [-0.13; -0.02]†
Italy	1.22 [1.19; 1.24]	1.17 [1.12; 1.23]	-0.04 [-0.10; 0.02]	2.8 [2.6; 3.0]	2.4 [2.2; 2.6]	1.00 [0.98; 1.02]	1.01 [0.97; 1.06]	0.01 [-0.04; 0.06]
Jamaica	1.33 [1.23; 1.44]	1.30 [1.18; 1.42]	-0.03 [-0.16; 0.10]	14.0 [7.2; 27.3]	10.8 [5.5; 21.2]	0.93 [0.85; 1.01]	0.95 [0.86; 1.04]	0.02 [-0.07; 0.11]
Japan	1.20 [1.18; 1.22]	1.11 [1.07; 1.15]	-0.09 [-0.13; -0.05]†	2.4 [2.3; 2.5]	2.2 [2.1; 2.3]	0.99 [0.97; 1.01]	1.07 [1.04; 1.11]*	0.08 [0.04; 0.12]†
Jordan¶	1.13 [1.08; 1.19]	1.23 [1.12; 1.33]	0.09 [-0.02; 0.20]	16.0 [11.1; 23.3]	13.1 [9.1; 19.0]	1.08 [1.02; 1.14]*	1.01 [0.93; 1.10]	-0.07 [-0.16; 0.02]
Kazakhstan¶	1.30 [1.21; 1.39]	1.29 [1.18; 1.42]	-0.01 [-0.13; 0.12]	11.5 [11.0; 12.2]	8.9 [8.4; 9.5]	0.93 [0.87; 1.00]*	0.95 [0.87; 1.04]	0.02 [-0.07; 0.11]
Kenya	1.12 [1.08; 1.16]	1.20 [1.12; 1.28]	0.08 [-0.01; 0.17]	40.4 [32.7; 50.1]	33.7 [27.1; 41.8]	1.00 [0.96; 1.04]	1.00 [0.94; 1.08]	0.01 [-0.07; 0.08]
Kiribati	1.14 [1.08; 1.21]	1.20 [1.11; 1.31]	0.06 [-0.04; 0.17]	52.5 [27.9; 97.9]	43.6 [23.0; 81.4]	1.00 [0.94; 1.06]	0.99 [0.91; 1.09]	0.00 [-0.10; 0.09]
Democratic People's Republic of Korea	1.20 [1.09; 1.33]	1.22 [1.11; 1.34]	0.01 [-0.10; 0.12]	16.9 [13.2; 21.6]	13.9 [10.8; 17.9]	1.00 [0.91; 1.10]	1.00 [0.91; 1.10]	0.00 [-0.09; 0.09]
Republic of Korea	1.18 [1.10; 1.26]	1.20 [1.15; 1.26]	0.03 [-0.07; 0.12]	3.2 [3.0; 3.3]	2.6 [2.5; 2.7]	1.05 [0.98; 1.12]	0.99 [0.94; 1.03]	-0.06 [-0.14; 0.02]
Kosovo	1.11 [1.02; 1.20]	1.16 [1.06; 1.26]	0.05 [-0.06; 0.17]	10.7 [9.3; 12.3]	9.2 [8.0; 10.6]	1.04 [0.95; 1.14]	1.05 [0.97; 1.15]	0.01 [-0.09; 0.12]
Kuwait	1.16 [1.11; 1.20]	1.21 [1.14; 1.28]	0.05 [-0.03; 0.13]	9.5 [8.6; 10.6]	7.9 [7.1; 8.8]	1.05 [1.01; 1.09]*	1.00 [0.95; 1.06]	-0.04 [-0.11; 0.03]
Kyrgyzstan	1.20 [1.12; 1.30]	1.25 [1.14; 1.37]	0.05 [-0.07; 0.17]	19.3 [18.2; 20.5]	15.4 [14.4; 16.5]	0.99 [0.91; 1.06]	0.99 [0.90; 1.09]	0.01 [-0.09; 0.11]
Lao People's Democratic Republic	1.12 [1.06; 1.18]	1.24 [1.14; 1.34]	0.12 [0.01; 0.22]†	46.9 [32.2; 66.9]	37.8 [25.9; 54.3]	0.98 [0.93; 1.04]	0.98 [0.90; 1.06]	-0.01 [-0.09; 0.08]
Latvia	1.29 [1.24; 1.34]	1.18 [1.09; 1.26]	-0.11 [-0.21; -0.01]†	4.0 [3.4; 4.7]	3.4 [2.9; 4.0]	0.96 [0.92; 0.99]*	1.01 [0.94; 1.08]	0.05 [-0.02; 0.13]
Lebanon¶	1.10 [1.03; 1.17]	1.13 [1.02; 1.23]	0.03 [-0.08; 0.14]	8.7 [7.0; 10.8]	7.7 [6.2; 9.6]	1.12 [1.05; 1.20]*	1.07 [0.98; 1.18]	-0.05 [-0.15; 0.06]
Lesotho	1.19 [1.13; 1.25]	1.20 [1.11; 1.29]	0.01 [-0.09; 0.11]	79.3 [55.1; 117.7]	66.1 [45.7; 97.7]	0.98 [0.92; 1.03]	0.97 [0.90; 1.05]	0.00 [-0.09; 0.09]
Liberia	1.12 [1.07; 1.16]	1.17 [1.10; 1.25]	0.05 [-0.03; 0.14]	81.9 [60.3; 112.2]	69.9 [51.5; 96.2]	0.97 [0.93; 1.01]	0.99 [0.92; 1.06]	0.02 [-0.06; 0.10]
Libya	1.19 [1.12; 1.27]	1.21 [1.11; 1.33]	0.02 [-0.08; 0.14]	11.8 [6.7; 20.6]	9.7 [5.5; 17.0]	1.03 [0.96; 1.10]	1.00 [0.92; 1.10]	-0.02 [-0.12; 0.07]
Lithuania	1.26 [1.21; 1.30]	1.20 [1.12; 1.28]	-0.06 [-0.14; 0.04]	3.6 [3.1; 4.2]	3.0 [2.6; 3.5]	0.98 [0.95; 1.01]	0.99 [0.93; 1.06]	0.01 [-0.06; 0.08]
Luxembourg	1.23 [1.14; 1.32]	1.21 [1.11; 1.31]	-0.02 [-0.14; 0.09]	3.0 [2.3; 3.9]	2.5 [1.9; 3.3]	0.99 [0.92; 1.06]	0.99 [0.91; 1.07]	0.00 [-0.09; 0.10]
Macedonia¶	1.12 [1.07; 1.19]	1.12 [1.05; 1.20]	-0.01 [-0.09; 0.09]	5.6 [4.5; 7.1]	5.0 [4.0; 6.4]	1.10 [1.04; 1.16]*	1.06 [1.00; 1.13]	-0.03 [-0.12; 0.05]
Madagascar	1.10 [1.06; 1.14]	1.18 [1.10; 1.26]	0.08 [0.00; 0.16]	71.4 [60.0; 86.3]	60.6 [50.9; 73.4]	0.99 [0.96; 1.03]	0.98 [0.91; 1.05]	-0.02 [-0.09; 0.06]
Malawi	1.10 [1.06; 1.13]	1.25 [1.17; 1.34]	0.15 [0.07; 0.25]†	46.4 [29.3; 73.4]	37.1 [23.2; 58.3]	0.98 [0.95; 1.01]	0.95 [0.89; 1.03]	-0.02 [-0.10; 0.06]
Malaysia	1.23 [1.12; 1.35]	1.19 [1.10; 1.29]	-0.04 [-0.16; 0.08]	8.2 [7.4; 9.1]	6.9 [6.2; 7.7]	1.01 [0.92; 1.10]	1.01 [0.94; 1.10]	0.01 [-0.10; 0.11]
Maldives	1.15 [1.07; 1.23]	1.19 [1.09; 1.28]	0.04 [-0.07; 0.14]	6.5 [5.4; 7.7]	5.4 [4.6; 6.5]	1.00 [0.93; 1.07]	1.01 [0.93; 1.09]	0.01 [-0.08; 0.10]
Mali	1.08 [1.05; 1.11]	1.11 [1.05; 1.18]	0.03 [-0.03; 0.10]	102.0 [81.5; 127.8]	91.9 [73.4; 114.9]	0.99 [0.96; 1.02]	1.01 [0.95; 1.07]	0.02 [-0.04; 0.09]
Malta	1.21 [1.12; 1.30]	1.16 [1.06; 1.26]	-0.05 [-0.16; 0.07]	6.2 [5.2; 7.5]	5.4 [4.5; 6.5]	1.02 [0.95; 1.09]	1.03 [0.95; 1.12]	0.01 [-0.09; 0.11]
Marshall Islands	1.21 [1.11; 1.32]	1.26 [1.15; 1.39]	0.05 [-0.07; 0.17]	33.0 [19.4; 57.0]	26.2 [15.5; 45.1]	0.99 [0.90; 1.09]	0.97 [0.88; 1.07]	-0.02 [-0.12; 0.08]
Mauritania	1.15 [1.09; 1.21]	1.23 [1.15; 1.32]	0.08 [-0.01; 0.18]	44.6 [34.1; 58.4]	36.2 [27.6; 47.7]	0.96 [0.92; 1.01]	0.98 [0.91; 1.06]	0.02 [-0.06; 0.10]
Mauritius	1.29 [1.24; 1.35]	1.23 [1.15; 1.31]	-0.07 [-0.16; 0.03]	18.3 [15.2; 21.9]	14.9 [12.4; 17.8]	0.96 [0.92; 1.00]*	1.01 [0.95; 1.08]	0.05 [-0.02; 0.13]
Mexico	1.18 [1.11; 1.26]	1.21 [1.15; 1.27]	0.03 [-0.07; 0.12]	14.5 [12.3; 16.9]	12.0 [10.2; 14.0]	1.02 [0.96; 1.09]	1.02 [0.97; 1.07]	0.00 [-0.09; 0.08]
Republic of Moldova	1.26 [1.16; 1.37]	1.25 [1.13; 1.37]	-0.01 [-0.14; 0.11]	15.7 [11.1; 22.6]	12.6 [8.9; 18.1]	0.97 [0.89; 1.06]	0.99 [0.90; 1.09]	0.02 [-0.08; 0.11]
Monaco	1.25 [1.19; 1.31]	1.24 [1.16; 1.33]	-0.01 [-0.10; 0.09]	3.2 [2.0; 5.1]	2.6 [1.6; 4.1]	0.96 [0.91; 1.01]	0.96 [0.90; 1.03]	0.01 [-0.07; 0.08]
Mongolia¶	1.28 [1.22; 1.34]	1.25 [1.19; 1.32]	-0.03 [-0.12; 0.06]	16.3 [15.0; 17.8]	13.1 [12.0; 14.3]	0.88 [0.84; 0.92]*	0.99 [0.94; 1.04]	0.11 [0.04; 0.17]†
Montenegro¶	1.13 [1.06; 1.20]	1.14 [1.05; 1.23]	0.01 [-0.09; 0.12]	2.4 [2.0; 2.9]	2.1 [1.7; 2.6]	1.10 [1.04; 1.17]*	1.04 [0.96; 1.13]	-0.06 [-0.15; 0.04]
Montserrat¶	0.91 [0.85; 0.98]	0.97 [0.86; 1.09]	0.06 [-0.04; 0.16]	5.2 [1.9; 14.0]	5.3 [2.0; 14.5]	1.35 [1.26; 1.44]*	1.23 [1.09; 1.40]*	-0.11 [-0.23; 0.03]
Morocco	1.13 [1.07; 1.18]	1.22 [1.11; 1.33]	0.09 [-0.02; 0.20]	19.7 [13.6; 27.8]	16.2 [11.2; 22.8]	1.03 [0.98; 1.09]	1.02 [0.93; 1.11]	-0.02 [-0.11; 0.08]
Mozambique	1.08 [1.04; 1.12]	1.14 [1.07; 1.22]	0.06 [-0.02; 0.15]	73.9 [47.8; 117.1]	64.9 [41.8; 102.4]	1.00 [0.97; 1.04]	1.02 [0.94; 1.10]	0.01 [-0.07; 0.10]
Myanmar	1.15 [1.08; 1.22]	1.22 [1.12; 1.32]	0.07 [-0.04; 0.18]	45.8 [29.4; 69.9]	37.6 [23.9; 57.3]	0.98 [0.92; 1.04]	0.99 [0.91; 1.08]	0.01 [-0.08; 0.11]
Namibia	1.15 [1.09; 1.22]	1.21 [1.12; 1.31]	0.06 [-0.04; 0.16]	42.7 [22.9; 83.0]	35.3 [19.0; 68.2]	0.99 [0.94; 1.05]	0.99 [0.90; 1.08]	0.00 [-0.10; 0.09]

¶: Sex ratio is outlying in 1990. §: Sex ratio is outlying in 2021. *: The ratio of estimated to expected female mortality is significantly different from one. †: Change is significantly different from zero.

Continued on next page

Table 15 – continued from previous page

	Sex ratio U5MR			Sex-specific U5MR in 2021 (per 1,000)		Estimated/Expected female U5MR		
	1990	2021	Change 1990–2021	Male	Female	1990	2021	Change 1990–2021
Nauru	1.17 [1.07; 1.29]	1.24 [1.12; 1.37]	0.06 [-0.05; 0.19]	30.4 [15.4; 58.7]	24.6 [12.5; 47.8]	1.00 [0.91; 1.10]	0.99 [0.89; 1.10]	-0.01 [-0.11; 0.10]
Nepal¶	1.01 [0.98; 1.05]	1.17 [1.08; 1.26]	0.16 [0.06; 0.25]†	29.2 [22.5; 38.5]	25.0 [19.2; 32.9]	1.10 [1.06; 1.15]*	1.06 [0.98; 1.14]	-0.04 [-0.13; 0.05]
Netherlands	1.30 [1.26; 1.34]	1.21 [1.15; 1.27]	-0.09 [-0.16; -0.03]†	4.4 [4.2; 4.6]	3.7 [3.5; 3.9]	0.93 [0.90; 0.96]*	0.98 [0.94; 1.03]	0.05 [0.00; 0.11]†
New Zealand	1.27 [1.23; 1.32]	1.19 [1.11; 1.27]	-0.08 [-0.17; 0.01]	5.1 [4.6; 5.7]	4.3 [3.9; 4.8]	0.96 [0.92; 0.99]*	1.00 [0.93; 1.07]	0.04 [-0.03; 0.12]
Nicaragua	1.19 [1.13; 1.24]	1.25 [1.15; 1.36]	0.06 [-0.05; 0.18]	14.7 [12.6; 16.9]	11.8 [10.1; 13.6]	0.98 [0.94; 1.03]	0.98 [0.90; 1.07]	0.00 [-0.08; 0.09]
Niger¶	1.01 [0.98; 1.04]	1.07 [1.01; 1.13]	0.06 [-0.01; 0.13]	118.8 [95.4; 149.0]	111.2 [89.4; 139.6]	1.04 [1.01; 1.06]*	1.03 [0.97; 1.09]	-0.01 [-0.07; 0.06]
Nigeria	1.11 [1.08; 1.14]	1.12 [1.06; 1.18]	0.01 [-0.06; 0.08]	116.9 [86.1; 161.1]	104.3 [76.8; 144.5]	0.97 [0.94; 1.00]	1.00 [0.94; 1.06]	0.03 [-0.04; 0.09]
Niue	1.24 [1.12; 1.37]	1.24 [1.12; 1.37]	0.00 [-0.13; 0.13]	26.6 [11.4; 63.5]	21.6 [9.1; 51.5]	1.00 [0.90; 1.11]	0.99 [0.89; 1.10]	-0.01 [-0.11; 0.09]
Norway	1.28 [1.23; 1.33]	1.22 [1.14; 1.30]	-0.06 [-0.14; 0.03]	2.4 [2.1; 2.6]	2.0 [1.8; 2.2]	0.95 [0.91; 0.99]*	0.97 [0.91; 1.04]	0.03 [-0.04; 0.10]
Oman	1.18 [1.09; 1.28]	1.21 [1.09; 1.33]	0.02 [-0.09; 0.14]	11.1 [9.9; 12.3]	9.2 [8.2; 10.3]	1.03 [0.95; 1.12]	1.01 [0.92; 1.12]	-0.02 [-0.11; 0.08]
Pakistan¶	1.05 [1.01; 1.09]	1.15 [1.08; 1.22]	0.10 [0.02; 0.18]†	67.5 [53.9; 84.6]	58.8 [46.8; 73.4]	1.07 [1.03; 1.11]*	1.04 [0.97; 1.11]	-0.03 [-0.11; 0.05]
Palau	1.24 [1.12; 1.37]	1.24 [1.11; 1.39]	0.00 [-0.13; 0.15]	18.0 [8.5; 38.2]	14.4 [6.9; 30.8]	0.99 [0.90; 1.10]	0.99 [0.89; 1.11]	0.00 [-0.11; 0.11]
Panama	1.23 [1.12; 1.34]	1.22 [1.11; 1.36]	0.00 [-0.12; 0.13]	15.2 [7.7; 31.0]	12.5 [6.2; 25.2]	1.00 [0.92; 1.10]	1.00 [0.90; 1.10]	0.00 [-0.11; 0.10]
Papua New Guinea	1.11 [1.04; 1.18]	1.18 [1.08; 1.28]	0.07 [-0.03; 0.17]	46.2 [31.0; 67.9]	39.3 [26.4; 58.0]	1.04 [0.97; 1.11]	1.03 [0.94; 1.12]	-0.01 [-0.10; 0.09]
Paraguay	1.18 [1.09; 1.27]	1.22 [1.11; 1.35]	0.04 [-0.07; 0.16]	19.9 [9.7; 42.3]	16.3 [7.9; 34.7]	1.02 [0.94; 1.11]	1.01 [0.91; 1.11]	-0.02 [-0.11; 0.08]
Peru¶	1.11 [1.07; 1.14]	1.21 [1.13; 1.30]	0.10 [0.01; 0.20]†	15.4 [12.3; 19.7]	12.8 [10.1; 16.3]	1.04 [1.00; 1.07]*	1.01 [0.94; 1.09]	-0.02 [-0.10; 0.06]
Philippines	1.22 [1.17; 1.27]	1.23 [1.14; 1.33]	0.01 [-0.09; 0.11]	28.3 [19.9; 40.1]	23.0 [16.1; 32.7]	0.96 [0.92; 1.00]	1.00 [0.93; 1.08]	0.04 [-0.04; 0.13]
Poland	1.28 [1.26; 1.30]	1.20 [1.15; 1.24]	-0.08 [-0.14; -0.03]†	4.7 [4.5; 4.9]	3.9 [3.8; 4.1]	0.97 [0.95; 0.99]*	0.99 [0.95; 1.03]	0.02 [-0.02; 0.07]
Portugal	1.26 [1.22; 1.30]	1.21 [1.14; 1.27]	-0.05 [-0.13; 0.02]	3.4 [3.1; 3.7]	2.8 [2.6; 3.0]	0.98 [0.95; 1.00]	0.98 [0.93; 1.04]	0.01 [-0.05; 0.07]
Qatar	1.18 [1.10; 1.26]	1.13 [1.06; 1.22]	-0.04 [-0.15; 0.06]	5.6 [5.0; 6.3]	5.0 [4.4; 5.6]	1.05 [0.98; 1.12]	1.05 [0.98; 1.12]	-0.01 [-0.10; 0.09]
Romania	1.24 [1.22; 1.26]	1.16 [1.11; 1.21]	-0.08 [-0.13; -0.03]†	6.9 [6.6; 7.2]	5.9 [5.6; 6.2]	0.99 [0.97; 1.01]	1.03 [0.99; 1.07]	0.04 [0.00; 0.08]
Russian Federation¶	1.35 [1.33; 1.36]	1.23 [1.19; 1.27]	-0.12 [-0.16; -0.07]†	5.6 [5.3; 5.9]	4.5 [4.3; 4.8]	0.92 [0.91; 0.93]*	0.96 [0.93; 0.99]*	0.05 [0.02; 0.08]†
Rwanda	1.12 [1.08; 1.16]	1.19 [1.11; 1.28]	0.07 [-0.02; 0.16]	42.8 [26.0; 71.0]	35.9 [21.8; 59.3]	0.98 [0.94; 1.01]	1.01 [0.93; 1.09]	0.03 [-0.05; 0.12]
Saint Kitts and Nevis	1.22 [1.13; 1.33]	1.22 [1.11; 1.34]	0.00 [-0.12; 0.12]	16.0 [10.7; 24.6]	13.1 [8.6; 20.0]	1.01 [0.93; 1.09]	1.01 [0.92; 1.11]	0.00 [-0.09; 0.10]
Saint Lucia	1.22 [1.15; 1.29]	1.21 [1.11; 1.32]	-0.01 [-0.12; 0.11]	27.1 [23.0; 31.8]	22.4 [19.0; 26.4]	1.02 [0.96; 1.08]	1.03 [0.94; 1.12]	0.01 [-0.09; 0.11]
Samoa	1.20 [1.11; 1.30]	1.22 [1.13; 1.33]	0.02 [-0.10; 0.14]	18.3 [12.7; 26.3]	15.0 [10.4; 21.6]	1.02 [0.95; 1.11]	1.01 [0.93; 1.10]	-0.01 [-0.11; 0.09]
San Marino	1.24 [1.12; 1.38]	1.19 [1.08; 1.33]	-0.05 [-0.18; 0.09]	1.9 [0.9; 3.8]	1.6 [0.7; 3.2]	0.99 [0.89; 1.10]	0.99 [0.89; 1.10]	0.00 [-0.11; 0.11]
Sao Tome and Principe	1.13 [1.06; 1.20]	1.24 [1.14; 1.35]	0.12 [0.01; 0.22]†	17.1 [9.7; 29.7]	13.7 [7.8; 23.9]	0.99 [0.93; 1.06]	0.99 [0.91; 1.07]	0.00 [-0.09; 0.08]
Saudi Arabia¶	1.09 [1.00; 1.18]	1.05 [0.96; 1.14]	-0.04 [-0.14; 0.06]	6.9 [5.2; 9.5]	6.6 [4.9; 9.0]	1.11 [1.03; 1.21]*	1.14 [1.05; 1.25]*	0.03 [-0.08; 0.14]
Senegal	1.11 [1.07; 1.14]	1.23 [1.15; 1.31]	0.12 [0.04; 0.21]†	42.4 [31.9; 56.1]	34.5 [25.9; 45.7]	0.98 [0.95; 1.01]	0.98 [0.91; 1.05]	0.00 [-0.08; 0.07]
Serbia¶	1.13 [1.10; 1.15]	1.21 [1.15; 1.28]	0.09 [0.02; 0.16]†	6.0 [5.6; 6.5]	4.9 [4.6; 5.3]	1.10 [1.07; 1.12]*	0.98 [0.93; 1.04]	-0.12 [-0.18; -0.06]†
Seychelles	1.17 [1.07; 1.26]	1.17 [1.07; 1.28]	0.01 [-0.11; 0.12]	15.0 [11.5; 19.7]	12.8 [9.8; 16.8]	1.06 [0.98; 1.15]	1.05 [0.97; 1.15]	-0.01 [-0.11; 0.10]
Sierra Leone	1.10 [1.06; 1.14]	1.14 [1.08; 1.21]	0.04 [-0.03; 0.12]	111.4 [90.9; 136.9]	97.6 [79.3; 119.8]	0.98 [0.94; 1.01]	0.99 [0.93; 1.06]	0.02 [-0.05; 0.09]
Singapore	1.17 [1.12; 1.22]	1.18 [1.10; 1.26]	0.01 [-0.08; 0.10]	2.3 [1.8; 2.7]	1.9 [1.6; 2.3]	1.03 [0.99; 1.07]	1.01 [0.94; 1.08]	-0.02 [-0.09; 0.07]
Slovakia	1.28 [1.24; 1.33]	1.21 [1.15; 1.27]	-0.08 [-0.15; 0.00]†	6.2 [5.8; 6.5]	5.1 [4.8; 5.4]	0.96 [0.93; 0.99]*	0.99 [0.94; 1.04]	0.03 [-0.03; 0.08]
Slovenia	1.25 [1.19; 1.32]	1.18 [1.09; 1.27]	-0.07 [-0.17; 0.03]	2.3 [2.0; 2.7]	2.0 [1.7; 2.3]	0.98 [0.93; 1.03]	1.01 [0.94; 1.09]	0.03 [-0.05; 0.12]
Solomon Islands	1.18 [1.07; 1.28]	1.20 [1.09; 1.32]	0.02 [-0.09; 0.14]	20.4 [12.5; 32.3]	17.0 [10.4; 26.9]	1.03 [0.95; 1.14]	1.03 [0.94; 1.13]	-0.01 [-0.11; 0.10]
Somalia	1.09 [1.01; 1.17]	1.11 [1.03; 1.20]	0.02 [-0.06; 0.11]	117.0 [53.2; 260.2]	105.4 [48.1; 234.3]	1.00 [0.93; 1.07]	1.00 [0.92; 1.10]	0.00 [-0.08; 0.10]
South Africa	1.18 [1.12; 1.25]	1.17 [1.11; 1.23]	-0.01 [-0.10; 0.07]	35.3 [32.3; 38.8]	30.3 [27.6; 33.2]	0.99 [0.94; 1.06]	1.05 [1.00; 1.10]	0.05 [-0.03; 0.13]
South Sudan	1.05 [0.98; 1.13]	1.10 [1.02; 1.20]	0.05 [-0.03; 0.14]	103.6 [36.3; 240.0]	93.9 [33.1; 217.7]	1.02 [0.95; 1.10]	1.02 [0.93; 1.13]	0.00 [-0.09; 0.10]
Spain	1.23 [1.20; 1.26]	1.20 [1.15; 1.26]	-0.02 [-0.09; 0.04]	3.3 [3.1; 3.5]	2.8 [2.6; 2.9]	0.99 [0.97; 1.01]	0.99 [0.94; 1.03]	0.00 [-0.05; 0.05]
Sri Lanka	1.19 [1.17; 1.22]	1.22 [1.14; 1.29]	0.02 [-0.05; 0.10]	7.4 [5.5; 9.9]	6.1 [4.5; 8.1]	1.04 [1.02; 1.06]*	0.99 [0.93; 1.05]	-0.05 [-0.11; 0.01]
Saint Vincent and the Grenadines	1.17 [1.09; 1.24]	1.20 [1.09; 1.32]	0.03 [-0.08; 0.15]	14.9 [10.9; 20.3]	12.4 [9.1; 17.1]	1.06 [1.00; 1.13]	1.03 [0.94; 1.13]	-0.03 [-0.13; 0.07]
State of Palestine¶	1.11 [1.05; 1.18]	1.19 [1.10; 1.29]	0.08 [-0.03; 0.19]	16.1 [11.6; 22.3]	13.5 [9.8; 18.9]	1.08 [1.02; 1.15]*	1.03 [0.95; 1.12]	-0.05 [-0.15; 0.05]
Sudan	1.13 [1.08; 1.18]	1.20 [1.12; 1.29]	0.07 [-0.02; 0.16]	59.7 [40.6; 87.8]	49.8 [33.7; 73.5]	0.98 [0.93; 1.02]	0.98 [0.91; 1.05]	0.00 [-0.08; 0.08]
Suriname	1.23 [1.14; 1.35]	1.27 [1.15; 1.40]	0.04 [-0.09; 0.16]	19.1 [12.1; 30.8]	15.1 [9.5; 24.6]	0.99 [0.90; 1.07]	0.98 [0.88; 1.07]	-0.01 [-0.11; 0.09]

¶: Sex ratio is outlying in 1990. §: Sex ratio is outlying in 2021. *: The ratio of estimated to expected female mortality is significantly different from one. †: Change is significantly different from zero.

Continued on next page

Table 15 – continued from previous page

	Sex ratio U5MR			Sex-specific U5MR in 2021 (per 1,000)		Estimated/Expected female U5MR		
	1990	2021	Change 1990–2021	Male	Female	1990	2021	Change 1990–2021
Eswatini	1.20 [1.13; 1.28]	1.21 [1.11; 1.30]	0.00 [-0.11; 0.10]	57.4 [34.8; 93.0]	47.6 [29.0; 78.6]	0.97 [0.91; 1.04]	0.99 [0.91; 1.08]	0.02 [-0.07; 0.12]
Sweden	1.24 [1.20; 1.29]	1.19 [1.13; 1.26]	-0.05 [-0.13; 0.03]	2.7 [2.5; 2.9]	2.2 [2.1; 2.4]	0.97 [0.93; 1.00]	0.99 [0.94; 1.05]	0.03 [-0.03; 0.09]
Switzerland	1.27 [1.22; 1.31]	1.18 [1.11; 1.24]	-0.09 [-0.17; -0.01]†	4.1 [3.9; 4.4]	3.5 [3.3; 3.8]	0.95 [0.92; 0.99]*	1.01 [0.95; 1.07]	0.05 [-0.01; 0.12]
Syria	1.17 [1.11; 1.23]	1.21 [1.11; 1.32]	0.04 [-0.07; 0.16]	24.3 [12.6; 33.9]	20.1 [10.4; 27.9]	1.05 [0.99; 1.11]	1.01 [0.93; 1.10]	-0.04 [-0.13; 0.06]
Tajikistan	1.17 [1.10; 1.24]	1.28 [1.18; 1.40]	0.11 [0.00; 0.24]†	35.1 [20.7; 60.9]	27.4 [16.0; 47.6]	0.98 [0.92; 1.04]	0.96 [0.88; 1.05]	-0.02 [-0.11; 0.07]
Tanzania	1.08 [1.04; 1.12]	1.17 [1.09; 1.25]	0.09 [0.01; 0.18]†	50.7 [34.4; 76.0]	43.4 [29.4; 64.7]	1.01 [0.97; 1.05]	1.01 [0.94; 1.09]	0.00 [-0.08; 0.09]
Thailand	1.23 [1.16; 1.32]	1.22 [1.11; 1.34]	-0.01 [-0.13; 0.10]	9.1 [7.6; 12.0]	7.4 [6.2; 9.8]	0.99 [0.93; 1.06]	0.99 [0.91; 1.09]	0.00 [-0.09; 0.10]
Timor Leste	1.08 [1.02; 1.15]	1.18 [1.09; 1.29]	0.10 [0.00; 0.21]†	54.6 [35.8; 82.4]	46.2 [30.2; 69.7]	1.02 [0.96; 1.09]	1.02 [0.93; 1.11]	0.00 [-0.10; 0.10]
Togo	1.13 [1.09; 1.18]	1.18 [1.10; 1.25]	0.04 [-0.04; 0.13]	67.5 [50.6; 89.4]	57.4 [43.1; 76.5]	0.97 [0.93; 1.01]	0.99 [0.92; 1.06]	0.02 [-0.06; 0.10]
Tonga	1.28 [1.16; 1.40]	1.27 [1.16; 1.38]	-0.01 [-0.14; 0.12]	12.4 [6.7; 21.7]	9.8 [5.2; 17.1]	0.97 [0.89; 1.06]	0.96 [0.88; 1.05]	0.00 [-0.10; 0.09]
Trinidad and Tobago	1.18 [1.08; 1.29]	1.21 [1.10; 1.34]	0.03 [-0.09; 0.15]	17.8 [7.3; 45.3]	14.7 [6.0; 37.5]	1.04 [0.96; 1.14]	1.02 [0.92; 1.12]	-0.03 [-0.14; 0.08]
Tunisia	1.13 [1.07; 1.20]	1.19 [1.13; 1.25]	0.05 [-0.03; 0.14]	17.7 [14.9; 20.9]	14.9 [12.5; 17.7]	1.05 [0.99; 1.11]	1.04 [0.99; 1.10]	-0.01 [-0.09; 0.07]
Turkey¶	1.07 [1.03; 1.12]	1.14 [1.10; 1.19]	0.07 [0.01; 0.14]†	9.6 [8.2; 11.3]	8.4 [7.2; 9.9]	1.09 [1.04; 1.14]*	1.06 [1.02; 1.10]*	-0.02 [-0.09; 0.04]
Turkmenistan¶	1.28 [1.19; 1.40]	1.32 [1.20; 1.46]	0.04 [-0.09; 0.17]	47.0 [30.7; 70.9]	35.5 [23.1; 54.0]	0.91 [0.83; 0.99]*	0.92 [0.83; 1.02]	0.02 [-0.08; 0.11]
Turks and Caicos Islands¶	1.48 [1.40; 1.56]	1.13 [1.06; 1.21]	-0.35 [-0.45; -0.24]†	4.8 [2.8; 8.1]	4.2 [2.5; 7.2]	0.83 [0.79; 0.88]*	1.05 [0.99; 1.13]	0.22 [0.14; 0.30]†
Tuvalu	1.18 [1.08; 1.28]	1.23 [1.12; 1.34]	0.05 [-0.07; 0.17]	23.3 [13.0; 41.0]	19.0 [10.7; 33.6]	1.01 [0.93; 1.12]	1.01 [0.92; 1.10]	-0.01 [-0.11; 0.09]
Uganda¶	1.14 [1.10; 1.18]	1.24 [1.16; 1.33]	0.10 [0.01; 0.19]†	46.5 [32.4; 66.3]	37.5 [26.1; 53.2]	0.95 [0.91; 0.98]*	0.96 [0.89; 1.03]	0.02 [-0.06; 0.09]
Ukraine	1.24 [1.14; 1.36]	1.21 [1.10; 1.34]	-0.03 [-0.16; 0.09]	9.0 [8.4; 9.6]	7.4 [6.9; 8.0]	1.00 [0.91; 1.09]	1.00 [0.91; 1.10]	0.00 [-0.10; 0.10]
United Arab Emirates	1.28 [1.19; 1.38]	1.22 [1.13; 1.31]	-0.06 [-0.18; 0.05]	7.0 [5.9; 8.2]	5.7 [4.9; 6.8]	0.97 [0.90; 1.04]	0.98 [0.91; 1.06]	0.02 [-0.07; 0.11]
United Kingdom	1.29 [1.26; 1.31]	1.20 [1.15; 1.25]	-0.09 [-0.14; -0.03]†	4.6 [4.3; 4.8]	3.8 [3.6; 4.0]	0.95 [0.93; 0.96]*	0.99 [0.95; 1.03]	0.04 [0.00; 0.09]†
United States of America	1.25 [1.24; 1.27]	1.20 [1.16; 1.25]	-0.05 [-0.09; -0.01]†	6.8 [6.4; 7.2]	5.7 [5.3; 6.0]	0.97 [0.96; 0.99]*	0.99 [0.96; 1.03]	0.02 [-0.02; 0.05]
Uruguay	1.24 [1.20; 1.28]	1.22 [1.15; 1.29]	-0.02 [-0.10; 0.06]	6.4 [5.8; 7.0]	5.2 [4.7; 5.8]	1.00 [0.97; 1.03]	0.98 [0.92; 1.04]	-0.02 [-0.08; 0.04]
Uzbekistan¶	1.28 [1.20; 1.37]	1.30 [1.19; 1.44]	0.02 [-0.10; 0.15]	15.9 [14.3; 18.2]	12.2 [10.9; 14.0]	0.92 [0.86; 0.98]*	0.95 [0.86; 1.04]	0.03 [-0.06; 0.12]
Vanuatu	1.17 [1.05; 1.28]	1.18 [1.06; 1.29]	0.01 [-0.11; 0.12]	25.1 [14.1; 45.0]	21.3 [12.0; 38.4]	1.05 [0.95; 1.17]	1.05 [0.95; 1.17]	0.00 [-0.10; 0.11]
Venezuela (Bolivarian Republic of)	1.22 [1.20; 1.24]	1.18 [1.11; 1.25]	-0.04 [-0.11; 0.03]	26.1 [19.2; 35.6]	22.1 [16.3; 30.2]	1.01 [0.99; 1.03]	1.00 [0.94; 1.06]	-0.01 [-0.07; 0.05]
Vietnam¶§	1.37 [1.30; 1.45]	1.42 [1.33; 1.51]	0.05 [-0.06; 0.15]	24.1 [21.3; 27.3]	17.0 [15.0; 19.3]	0.85 [0.80; 0.90]*	0.87 [0.81; 0.93]*	0.02 [-0.05; 0.08]
Yemen	1.09 [1.05; 1.13]	1.14 [1.06; 1.23]	0.05 [-0.04; 0.15]	66.3 [29.5; 141.0]	58.0 [25.6; 122.5]	1.03 [0.99; 1.06]	1.02 [0.94; 1.12]	0.00 [-0.09; 0.10]
Zambia	1.10 [1.06; 1.13]	1.19 [1.11; 1.27]	0.09 [0.01; 0.18]†	62.5 [44.4; 87.3]	52.6 [37.4; 73.7]	0.99 [0.96; 1.02]	0.98 [0.91; 1.05]	-0.01 [-0.08; 0.07]
Zimbabwe	1.18 [1.13; 1.24]	1.21 [1.13; 1.29]	0.02 [-0.07; 0.11]	54.0 [37.1; 78.4]	44.8 [30.8; 65.2]	0.96 [0.91; 1.01]	0.98 [0.91; 1.05]	0.02 [-0.06; 0.10]

Table 16: **Estimates and 90% uncertainty intervals for sex ratios for 5q5 in 1990 and 2021, the change in sex ratios from 1990 to 2021, sex-specific 5q5 in 2021, and ratios of estimated to expected female 5q5 and their change from 1990 to 2021 for the world, UNICEF regions, and all countries.** ¶: Sex ratio is outlying in 1990. §: Sex ratio is outlying in 2021. *: The ratio of estimated to expected female mortality is significantly different from one. †: Change is significantly different from zero.

	Sex ratio 5q5			Sex-specific 5q5 in 2021 (per 1,000)		Estimated/Expected female 5q5		
	1990	2021	Change 1990–2021	Male	Female	1990	2021	Change 1990–2021
World	1.04 [1.01; 1.07]	1.13 [1.07; 1.17]	0.08 [0.03; 0.12]†	3.7 [3.5; 3.9]	3.3 [3.1; 3.5]	1.04 [1.01; 1.07]*	1.02 [0.97; 1.06]	-0.03 [-0.06; 0.03]
South Asia¶	0.92 [0.87; 0.98]	1.23 [1.10; 1.37]	0.31 [0.16; 0.47]†	2.4 [2.1; 2.7]	2.0 [1.7; 2.2]	1.13 [1.06; 1.21]*	1.13 [1.00; 1.27]*	0.00 [-0.15; 0.16]
Europe and Central Asia	1.41 [1.35; 1.46]	1.31 [1.27; 1.35]	-0.10 [-0.17; -0.03]†	0.7 [0.7; 0.8]	0.6 [0.6; 0.6]	0.93 [0.89; 0.97]*	1.03 [1.00; 1.06]*	0.10 [0.05; 0.16]†
Middle East and North Africa	1.15 [1.09; 1.20]	1.35 [1.25; 1.45]	0.20 [0.08; 0.32]†	2.3 [2.0; 2.5]	1.7 [1.5; 1.9]	0.99 [0.94; 1.05]	1.02 [0.94; 1.11]	0.03 [-0.07; 0.13]
Sub-Saharan Africa	1.07 [1.03; 1.12]	1.09 [1.02; 1.14]	0.01 [-0.07; 0.08]	9.9 [9.1; 10.6]	9.1 [8.4; 9.7]	0.99 [0.95; 1.02]	0.99 [0.94; 1.05]	0.00 [-0.06; 0.09]
Latin America and Caribbean	1.25 [1.22; 1.29]	1.24 [1.17; 1.31]	-0.01 [-0.09; 0.07]	1.3 [1.3; 1.4]	1.1 [1.0; 1.2]	1.04 [1.01; 1.07]*	1.09 [1.02; 1.15]*	0.05 [-0.03; 0.12]
East Asia and Pacific	1.17 [1.10; 1.25]	1.41 [1.27; 1.56]	0.24 [0.07; 0.42]†	1.6 [1.3; 1.8]	1.1 [1.0; 1.3]	1.01 [0.93; 1.09]	0.98 [0.88; 1.08]	-0.04 [-0.17; 0.10]
North America	1.36 [1.32; 1.41]	1.19 [1.10; 1.29]	-0.17 [-0.27; -0.06]†	0.6 [0.6; 0.6]	0.5 [0.5; 0.5]	1.03 [0.99; 1.07]	1.08 [1.00; 1.17]*	0.05 [-0.04; 0.15]
Afghanistan	1.04 [0.92; 1.19]	1.49 [1.19; 1.84]	0.44 [0.09; 0.84]†	2.7 [1.9; 3.4]	1.8 [1.3; 2.3]	1.00 [0.88; 1.15]	0.97 [0.78; 1.21]	-0.03 [-0.28; 0.25]
Albania	1.37 [1.26; 1.49]	1.09 [0.90; 1.31]	-0.28 [-0.51; -0.03]†	0.9 [0.8; 1.0]	0.8 [0.7; 0.9]	0.98 [0.90; 1.07]	1.24 [1.04; 1.48]*	0.26 [0.03; 0.53]†
Algeria	1.33 [1.21; 1.45]	1.17 [1.08; 1.27]	-0.15 [-0.31; 0.00]†	1.5 [1.5; 1.6]	1.3 [1.2; 1.4]	0.91 [0.82; 1.02]	1.21 [1.11; 1.31]*	0.30 [0.16; 0.43]†
Andorra	1.43 [1.26; 1.62]	1.30 [1.02; 1.65]	-0.13 [-0.49; 0.28]	0.4 [0.3; 0.5]	0.3 [0.2; 0.4]	0.99 [0.87; 1.12]	0.98 [0.77; 1.24]	-0.01 [-0.27; 0.29]
Angola	1.03 [0.91; 1.17]	1.10 [0.88; 1.38]	0.07 [-0.21; 0.39]	9.9 [7.5; 12.9]	9.0 [6.8; 11.8]	1.00 [0.88; 1.13]	0.97 [0.76; 1.25]	-0.03 [-0.29; 0.29]
Anguilla	1.42 [1.25; 1.61]	1.27 [1.00; 1.62]	-0.15 [-0.51; 0.26]	0.5 [0.4; 0.7]	0.4 [0.3; 0.5]	1.00 [0.88; 1.13]	1.00 [0.79; 1.27]	0.00 [-0.26; 0.31]
Antigua and Barbuda	1.42 [1.25; 1.61]	1.39 [1.09; 1.76]	-0.03 [-0.41; 0.39]	0.7 [0.6; 0.9]	0.5 [0.4; 0.7]	0.99 [0.88; 1.12]	0.95 [0.76; 1.20]	-0.04 [-0.28; 0.24]
Argentina	1.38 [1.31; 1.46]	1.16 [1.05; 1.27]	-0.23 [-0.36; -0.09]†	0.7 [0.6; 0.7]	0.6 [0.5; 0.6]	1.03 [0.98; 1.09]	1.13 [1.03; 1.24]*	0.10 [-0.02; 0.22]
Armenia	1.44 [1.31; 1.58]	1.29 [1.09; 1.53]	-0.15 [-0.41; 0.14]	1.1 [0.9; 1.2]	0.8 [0.7; 0.9]	1.00 [0.91; 1.10]	1.07 [0.91; 1.26]	0.07 [-0.13; 0.29]
Australia	1.38 [1.28; 1.48]	1.27 [1.12; 1.45]	-0.11 [-0.29; 0.10]	0.4 [0.3; 0.4]	0.3 [0.3; 0.3]	1.00 [0.93; 1.07]	1.00 [0.87; 1.13]	0.00 [-0.15; 0.16]
Austria	1.37 [1.25; 1.51]	1.15 [0.97; 1.35]	-0.22 [-0.46; 0.04]	0.3 [0.3; 0.4]	0.3 [0.3; 0.3]	1.00 [0.92; 1.10]	1.10 [0.94; 1.31]	0.10 [-0.10; 0.33]
Azerbaijan	1.30 [1.22; 1.39]	1.29 [1.16; 1.44]	-0.01 [-0.17; 0.16]	1.7 [1.6; 1.8]	1.3 [1.2; 1.4]	1.06 [0.98; 1.15]	1.10 [0.99; 1.23]	0.04 [-0.11; 0.19]
Bahamas	1.43 [1.26; 1.62]	1.35 [1.07; 1.70]	-0.09 [-0.45; 0.33]	1.1 [0.8; 1.5]	0.8 [0.6; 1.1]	1.00 [0.88; 1.14]	1.03 [0.82; 1.28]	0.02 [-0.24; 0.32]
Bahrain	1.44 [1.28; 1.63]	1.33 [1.12; 1.57]	-0.11 [-0.40; 0.19]	1.0 [0.8; 1.2]	0.7 [0.6; 0.9]	1.00 [0.89; 1.12]	1.02 [0.87; 1.21]	0.02 [-0.18; 0.24]
Bangladesh	0.98 [0.89; 1.08]	1.46 [1.24; 1.72]	0.49 [0.24; 0.76]†	2.9 [2.6; 3.2]	2.0 [1.7; 2.3]	1.05 [0.95; 1.16]	0.99 [0.84; 1.16]	-0.06 [-0.26; 0.15]
Barbados	1.43 [1.26; 1.62]	1.28 [1.00; 1.64]	-0.15 [-0.50; 0.27]	0.5 [0.3; 0.9]	0.4 [0.2; 0.7]	0.99 [0.88; 1.12]	1.00 [0.79; 1.28]	0.01 [-0.26; 0.32]
Belarus	1.66 [1.55; 1.78]	1.33 [1.13; 1.57]	-0.33 [-0.56; -0.07]†	0.6 [0.5; 0.6]	0.4 [0.4; 0.5]	0.87 [0.81; 0.94]*	0.96 [0.83; 1.12]	0.09 [-0.06; 0.26]
Belgium	1.33 [1.22; 1.46]	1.29 [1.11; 1.50]	-0.04 [-0.27; 0.20]	0.4 [0.4; 0.4]	0.3 [0.3; 0.3]	1.04 [0.96; 1.13]	0.99 [0.85; 1.15]	-0.06 [-0.23; 0.13]
Belize	1.44 [1.27; 1.63]	1.32 [1.05; 1.67]	-0.12 [-0.47; 0.28]	1.4 [1.1; 1.7]	1.0 [0.8; 1.3]	1.00 [0.88; 1.14]	1.07 [0.85; 1.33]	0.06 [-0.20; 0.37]
Benin	1.03 [0.92; 1.16]	1.05 [0.87; 1.28]	0.02 [-0.22; 0.28]	12.2 [9.4; 15.4]	11.6 [8.9; 14.7]	1.01 [0.91; 1.12]	0.99 [0.81; 1.21]	-0.02 [-0.24; 0.24]
Bhutan	1.03 [0.91; 1.18]	1.46 [1.16; 1.86]	0.43 [0.07; 0.86]†	3.0 [1.1; 6.3]	2.1 [0.8; 4.3]	1.00 [0.88; 1.16]	0.95 [0.71; 1.22]	-0.06 [-0.37; 0.25]
Bolivia (Plurinational State of)	1.09 [0.96; 1.23]	1.42 [1.12; 1.81]	0.33 [-0.02; 0.75]	2.4 [1.3; 3.5]	1.7 [0.9; 2.5]	1.03 [0.90; 1.19]	1.01 [0.79; 1.28]	-0.02 [-0.31; 0.29]
Bosnia and Herzegovina	1.51 [1.35; 1.69]	1.31 [1.12; 1.54]	-0.20 [-0.48; 0.09]	0.6 [0.5; 0.7]	0.4 [0.4; 0.5]	0.94 [0.84; 1.05]	0.98 [0.84; 1.14]	0.04 [-0.15; 0.24]
Botswana	1.04 [0.91; 1.18]	1.43 [1.14; 1.80]	0.39 [0.05; 0.80]†	4.7 [2.9; 6.6]	3.3 [2.0; 4.7]	1.00 [0.88; 1.14]	0.92 [0.70; 1.20]	-0.08 [-0.36; 0.24]
Brazil	1.43 [1.30; 1.58]	1.28 [1.11; 1.48]	-0.15 [-0.39; 0.10]	1.0 [1.0; 1.1]	0.8 [0.7; 0.9]	1.01 [0.92; 1.11]	1.07 [0.93; 1.23]	0.06 [-0.12; 0.25]
British Virgin Islands	1.43 [1.26; 1.62]	1.38 [1.08; 1.77]	-0.05 [-0.41; 0.39]	1.1 [0.9; 1.5]	0.8 [0.6; 1.1]	1.00 [0.88; 1.13]	1.00 [0.78; 1.26]	0.00 [-0.26; 0.29]
Brunei	1.46 [1.29; 1.65]	1.36 [1.08; 1.72]	-0.10 [-0.46; 0.32]	1.2 [0.9; 1.5]	0.9 [0.7; 1.1]	0.99 [0.88; 1.12]	1.02 [0.81; 1.28]	0.04 [-0.23; 0.33]
Bulgaria	1.43 [1.33; 1.54]	1.23 [1.07; 1.43]	-0.20 [-0.41; 0.02]	0.8 [0.7; 0.9]	0.7 [0.6; 0.7]	1.01 [0.94; 1.08]	1.09 [0.94; 1.25]	0.08 [-0.09; 0.26]
Burkina Faso	1.07 [0.96; 1.19]	1.10 [0.88; 1.37]	0.03 [-0.24; 0.34]	11.9 [7.4; 15.5]	10.8 [6.7; 14.2]	0.98 [0.88; 1.08]	0.96 [0.76; 1.22]	-0.02 [-0.26; 0.28]
Burundi	1.07 [0.94; 1.22]	1.07 [0.87; 1.32]	0.00 [-0.27; 0.30]	11.4 [8.0; 14.8]	10.6 [7.4; 13.9]	1.01 [0.90; 1.14]	0.98 [0.79; 1.24]	-0.03 [-0.27; 0.26]
Cambodia	0.99 [0.88; 1.11]	1.49 [1.17; 1.88]	0.50 [0.14; 0.92]†	3.2 [1.6; 4.6]	2.2 [1.1; 3.1]	1.04 [0.93; 1.16]	0.95 [0.74; 1.22]	-0.09 [-0.36; 0.22]
Cameroon	1.03 [0.92; 1.16]	1.15 [0.94; 1.40]	0.12 [-0.14; 0.41]	14.2 [10.4; 17.8]	12.3 [9.0; 15.5]	1.00 [0.89; 1.12]	0.90 [0.73; 1.11]	-0.10 [-0.31; 0.15]
Canada	1.31 [1.22; 1.40]	1.14 [1.01; 1.29]	-0.17 [-0.32; 0.00]	0.4 [0.3; 0.4]	0.3 [0.3; 0.4]	1.06 [0.99; 1.13]	1.11 [0.99; 1.26]	0.05 [-0.10; 0.21]

¶: Sex ratio is outlying in 1990. §: Sex ratio is outlying in 2021. *: The ratio of estimated to expected female mortality is significantly different from one. †: Change is significantly different from zero.

Continued on next page

Table 16 – continued from previous page

	Sex ratio 5q5			Sex-specific 5q5 in 2021 (per 1,000)		Estimated/Expected female 5q5		
	1990	2021	Change 1990–2021	Male	Female	1990	2021	Change 1990–2021
Cape Verde	1.40 [1.24; 1.58]	1.08 [0.89; 1.32]	-0.32 [-0.59; -0.02]†	0.9 [0.7; 1.1]	0.8 [0.7; 1.0]	0.99 [0.87; 1.14]	1.25 [1.03; 1.50]*	0.25 [-0.02; 0.54]
Central African Republic	1.04 [0.92; 1.17]	0.93 [0.75; 1.16]	-0.11 [-0.35; 0.16]	12.6 [8.5; 17.0]	13.5 [9.1; 18.2]	0.99 [0.88; 1.11]	1.12 [0.89; 1.43]	0.13 [-0.14; 0.48]
Chad	1.06 [0.95; 1.20]	1.03 [0.85; 1.24]	-0.04 [-0.27; 0.21]	12.5 [9.2; 16.2]	12.2 [9.0; 15.8]	1.00 [0.90; 1.12]	1.01 [0.84; 1.24]	0.01 [-0.21; 0.28]
Chile	1.43 [1.34; 1.52]	1.20 [1.04; 1.37]	-0.23 [-0.42; -0.03]†	0.6 [0.6; 0.6]	0.5 [0.5; 0.5]	1.00 [0.94; 1.07]	1.07 [0.94; 1.22]	0.07 [-0.08; 0.24]
China	1.29 [1.14; 1.46]	1.51 [1.22; 1.88]	0.23 [-0.14; 0.65]	1.0 [0.7; 1.4]	0.7 [0.5; 0.9]	1.00 [0.86; 1.16]	0.90 [0.73; 1.11]	-0.10 [-0.35; 0.17]
Colombia	1.43 [1.28; 1.60]	1.33 [1.14; 1.54]	-0.10 [-0.36; 0.17]	1.2 [1.1; 1.2]	0.9 [0.8; 1.0]	1.01 [0.91; 1.13]	1.04 [0.91; 1.21]	0.03 [-0.16; 0.23]
Comoros	1.01 [0.89; 1.14]	1.27 [1.01; 1.60]	0.27 [-0.05; 0.63]	4.0 [2.5; 5.5]	3.2 [1.9; 4.3]	1.04 [0.91; 1.19]	1.08 [0.82; 1.39]	0.04 [-0.27; 0.39]
Congo	1.03 [0.91; 1.17]	1.39 [1.09; 1.75]	0.36 [0.02; 0.76]†	4.0 [2.1; 6.2]	2.9 [1.5; 4.5]	1.00 [0.89; 1.14]	0.98 [0.75; 1.28]	-0.02 [-0.32; 0.31]
Democratic Republic of the Congo	1.04 [0.91; 1.18]	1.03 [0.84; 1.27]	0.00 [-0.26; 0.28]	11.0 [7.4; 14.8]	10.7 [7.1; 14.3]	1.00 [0.88; 1.13]	1.02 [0.82; 1.29]	0.02 [-0.23; 0.33]
Cook Islands	1.46 [1.29; 1.66]	1.38 [1.08; 1.77]	-0.08 [-0.46; 0.37]	1.0 [0.8; 1.4]	0.7 [0.6; 1.0]	0.97 [0.85; 1.11]	0.99 [0.78; 1.25]	0.02 [-0.26; 0.33]
Costa Rica	1.34 [1.21; 1.49]	1.23 [1.05; 1.46]	-0.11 [-0.36; 0.17]	0.8 [0.7; 0.9]	0.6 [0.5; 0.7]	1.06 [0.96; 1.18]	1.08 [0.92; 1.26]	0.02 [-0.19; 0.24]
Cote d'Ivoire	1.05 [0.93; 1.18]	0.96 [0.78; 1.20]	-0.08 [-0.32; 0.19]	13.2 [9.3; 17.6]	13.7 [9.7; 18.4]	0.98 [0.87; 1.10]	1.07 [0.86; 1.35]	0.10 [-0.16; 0.41]
Croatia	1.42 [1.29; 1.56]	1.36 [1.14; 1.64]	-0.06 [-0.33; 0.26]	0.8 [0.7; 0.9]	0.6 [0.5; 0.7]	1.00 [0.91; 1.10]	0.98 [0.82; 1.17]	-0.02 [-0.22; 0.19]
Cuba	1.45 [1.34; 1.58]	1.27 [1.11; 1.45]	-0.18 [-0.39; 0.04]	0.9 [0.9; 1.0]	0.7 [0.7; 0.8]	0.99 [0.91; 1.07]	1.07 [0.94; 1.21]	0.08 [-0.08; 0.24]
Cyprus	1.39 [1.23; 1.57]	1.24 [0.98; 1.57]	-0.15 [-0.49; 0.24]	0.6 [0.5; 0.8]	0.5 [0.4; 0.6]	1.00 [0.89; 1.13]	1.04 [0.83; 1.30]	0.04 [-0.23; 0.34]
Czech Republic	1.46 [1.35; 1.59]	1.11 [0.96; 1.29]	-0.35 [-0.55; -0.13]†	0.4 [0.4; 0.5]	0.4 [0.3; 0.4]	0.97 [0.89; 1.05]	1.14 [0.98; 1.32]	0.17 [-0.02; 0.37]
Denmark	1.42 [1.28; 1.58]	1.17 [0.97; 1.40]	-0.25 [-0.52; 0.04]	0.3 [0.3; 0.4]	0.3 [0.2; 0.3]	0.97 [0.88; 1.07]	1.08 [0.90; 1.30]	0.12 [-0.10; 0.37]
Djibouti	1.03 [0.91; 1.17]	1.12 [0.87; 1.44]	0.09 [-0.21; 0.44]	7.7 [5.8; 10.1]	6.9 [5.2; 9.1]	1.00 [0.88; 1.13]	1.01 [0.76; 1.35]	0.01 [-0.29; 0.38]
Dominica	1.46 [1.29; 1.65]	1.42 [1.11; 1.80]	-0.04 [-0.42; 0.39]	1.7 [0.9; 2.6]	1.2 [0.6; 1.9]	0.98 [0.87; 1.11]	1.00 [0.79; 1.27]	0.02 [-0.24; 0.32]
Dominican Republic	1.29 [1.14; 1.46]	1.43 [1.13; 1.79]	0.14 [-0.22; 0.55]	1.8 [1.2; 2.6]	1.3 [0.8; 1.8]	1.02 [0.88; 1.17]	1.00 [0.80; 1.26]	-0.02 [-0.29; 0.29]
Ecuador	1.25 [1.17; 1.33]	1.23 [1.11; 1.36]	-0.02 [-0.17; 0.13]	1.2 [1.1; 1.2]	0.9 [0.9; 1.0]	1.09 [1.01; 1.17]*	1.13 [1.03; 1.25]*	0.04 [-0.09; 0.18]
Egypt	1.16 [1.06; 1.28]	1.52 [1.30; 1.79]	0.36 [0.11; 0.65]†	2.5 [2.4; 2.7]	1.7 [1.5; 1.8]	1.03 [0.92; 1.14]	0.95 [0.81; 1.11]	-0.07 [-0.26; 0.12]
El Salvador	1.33 [1.23; 1.44]	1.27 [1.10; 1.47]	-0.06 [-0.27; 0.17]	1.4 [1.3; 1.5]	1.1 [1.0; 1.2]	1.06 [0.98; 1.16]	1.11 [0.96; 1.28]	0.05 [-0.13; 0.24]
Equatorial Guinea	1.03 [0.90; 1.17]	1.07 [0.84; 1.38]	0.05 [-0.25; 0.39]	9.3 [7.0; 12.2]	8.7 [6.5; 11.4]	1.00 [0.88; 1.13]	1.00 [0.77; 1.33]	0.00 [-0.29; 0.36]
Eritrea	1.06 [0.94; 1.20]	1.44 [1.14; 1.84]	0.38 [0.04; 0.80]†	4.1 [2.2; 6.3]	2.8 [1.5; 4.4]	0.98 [0.88; 1.10]	0.94 [0.70; 1.22]	-0.04 [-0.32; 0.27]
Estonia	1.47 [1.32; 1.63]	1.20 [0.98; 1.47]	-0.27 [-0.56; 0.06]	0.5 [0.4; 0.6]	0.4 [0.3; 0.5]	0.99 [0.89; 1.10]	1.06 [0.87; 1.29]	0.07 [-0.16; 0.33]
Ethiopia	1.14 [1.00; 1.30]	1.37 [1.09; 1.71]	0.23 [-0.11; 0.61]	4.8 [3.1; 6.4]	3.5 [2.3; 4.7]	0.98 [0.87; 1.09]	0.96 [0.74; 1.25]	-0.02 [-0.28; 0.30]
Federated States of Micronesia	1.23 [1.08; 1.39]	1.43 [1.11; 1.83]	0.20 [-0.18; 0.64]	3.3 [2.5; 4.3]	2.3 [1.7; 3.1]	1.00 [0.85; 1.18]	0.99 [0.76; 1.29]	0.00 [-0.30; 0.34]
Fiji	1.15 [1.01; 1.30]	1.43 [1.20; 1.70]	0.28 [-0.01; 0.59]	2.5 [2.2; 2.9]	1.8 [1.5; 2.1]	1.01 [0.88; 1.18]	1.02 [0.86; 1.20]	0.00 [-0.24; 0.25]
Finland	1.39 [1.26; 1.54]	1.06 [0.89; 1.28]	-0.33 [-0.57; -0.06]†	0.3 [0.3; 0.4]	0.3 [0.3; 0.3]	0.99 [0.90; 1.09]	1.19 [0.99; 1.43]	0.20 [-0.04; 0.46]
France	1.32 [1.25; 1.39]	1.32 [1.18; 1.47]	0.00 [-0.16; 0.17]	0.4 [0.4; 0.4]	0.3 [0.3; 0.3]	1.05 [0.99; 1.11]	0.96 [0.86; 1.08]	-0.08 [-0.21; 0.04]
Gabon	1.08 [0.95; 1.22]	1.24 [0.98; 1.58]	0.17 [-0.15; 0.54]	5.6 [2.3; 9.9]	4.5 [1.8; 8.0]	0.99 [0.86; 1.17]	1.01 [0.73; 1.37]	0.02 [-0.33; 0.42]
The Gambia	1.03 [0.91; 1.17]	1.23 [0.99; 1.53]	0.20 [-0.10; 0.54]	5.8 [4.5; 7.6]	4.7 [3.6; 6.2]	1.00 [0.88; 1.13]	1.00 [0.77; 1.29]	0.00 [-0.28; 0.33]
Georgia	1.45 [1.30; 1.61]	1.34 [1.12; 1.62]	-0.10 [-0.40; 0.23]	0.9 [0.8; 1.0]	0.7 [0.6; 0.8]	1.00 [0.89; 1.11]	1.01 [0.84; 1.20]	0.01 [-0.20; 0.24]
Germany	1.33 [1.26; 1.40]	1.29 [1.17; 1.42]	-0.04 [-0.18; 0.10]	0.4 [0.4; 0.4]	0.3 [0.3; 0.3]	1.04 [0.99; 1.10]	0.99 [0.89; 1.09]	-0.05 [-0.16; 0.06]
Ghana	1.09 [0.97; 1.22]	1.16 [0.94; 1.43]	0.07 [-0.20; 0.38]	6.0 [4.5; 7.7]	5.2 [3.9; 6.6]	0.94 [0.84; 1.06]	1.05 [0.81; 1.35]	0.10 [-0.17; 0.44]
Greece	1.32 [1.21; 1.44]	1.34 [1.14; 1.58]	0.02 [-0.22; 0.29]	0.4 [0.4; 0.4]	0.3 [0.3; 0.3]	1.04 [0.96; 1.13]	0.95 [0.80; 1.11]	-0.09 [-0.27; 0.10]
Grenada	1.43 [1.26; 1.62]	1.46 [1.14; 1.84]	0.02 [-0.37; 0.46]	2.1 [1.4; 2.9]	1.4 [1.0; 2.1]	1.00 [0.87; 1.14]	0.99 [0.78; 1.26]	-0.01 [-0.28; 0.30]
Guatemala¶	0.97 [0.93; 1.01]	1.14 [1.01; 1.28]	0.17 [0.04; 0.32]†	1.5 [1.4; 1.6]	1.4 [1.3; 1.5]	1.17 [1.11; 1.25]*	1.25 [1.11; 1.40]*	0.07 [-0.08; 0.24]
Guinea	1.03 [0.91; 1.16]	1.02 [0.82; 1.25]	-0.01 [-0.26; 0.27]	12.0 [8.7; 15.0]	11.9 [8.6; 14.8]	1.02 [0.91; 1.14]	1.03 [0.83; 1.29]	0.01 [-0.23; 0.31]
Guinea-Bissau	1.08 [0.94; 1.23]	1.15 [0.92; 1.44]	0.08 [-0.21; 0.40]	8.1 [4.6; 11.9]	7.0 [4.0; 10.4]	0.99 [0.87; 1.13]	0.98 [0.76; 1.30]	-0.02 [-0.29; 0.31]
Guyana	1.39 [1.25; 1.56]	1.33 [1.08; 1.65]	-0.07 [-0.39; 0.30]	2.2 [1.7; 2.7]	1.6 [1.3; 2.1]	1.03 [0.92; 1.16]	1.08 [0.88; 1.33]	0.05 [-0.21; 0.33]
Haiti	1.02 [0.91; 1.16]	1.01 [0.79; 1.27]	-0.02 [-0.28; 0.29]	6.8 [4.5; 9.2]	6.8 [4.4; 9.2]	1.00 [0.89; 1.12]	1.13 [0.85; 1.53]	0.14 [-0.19; 0.55]
Honduras	1.17 [1.04; 1.32]	1.39 [1.11; 1.75]	0.22 [-0.13; 0.62]	2.1 [1.3; 3.1]	1.5 [1.0; 2.3]	1.03 [0.89; 1.20]	1.03 [0.82; 1.29]	0.00 [-0.28; 0.32]
Hungary	1.39 [1.28; 1.50]	1.24 [1.07; 1.46]	-0.14 [-0.36; 0.10]	0.5 [0.4; 0.5]	0.4 [0.3; 0.4]	1.02 [0.94; 1.10]	1.02 [0.87; 1.19]	0.00 [-0.17; 0.19]
Iceland	1.40 [1.23; 1.58]	1.29 [1.02; 1.64]	-0.11 [-0.46; 0.29]	0.2 [0.1; 0.3]	0.2 [0.1; 0.2]	0.99 [0.88; 1.12]	0.98 [0.78; 1.24]	-0.01 [-0.26; 0.29]

¶: Sex ratio is outlying in 1990. §: Sex ratio is outlying in 2021. *: The ratio of estimated to expected female mortality is significantly different from one. †: Change is significantly different from zero.

Continued on next page

Table 16 – continued from previous page

	Sex ratio 5q5			Sex-specific 5q5 in 2021 (per 1,000)		Estimated/Expected female 5q5		
	1990	2021	Change 1990–2021	Male	Female	1990	2021	Change 1990–2021
India¶	0.90 [0.83; 0.97]	1.23 [1.05; 1.44]	0.33 [0.13; 0.54]†	1.9 [1.5; 2.2]	1.5 [1.2; 1.8]	1.15 [1.06; 1.25]*	1.16 [1.00; 1.36]	0.02 [-0.18; 0.23]
Indonesia	1.03 [0.93; 1.15]	1.39 [1.13; 1.72]	0.36 [0.06; 0.71]†	3.2 [2.3; 4.1]	2.3 [1.6; 3.0]	1.04 [0.93; 1.17]	1.02 [0.82; 1.28]	-0.02 [-0.27; 0.26]
Iran (Islamic Republic of)	1.05 [0.93; 1.19]	1.29 [1.08; 1.54]	0.24 [-0.03; 0.53]	1.7 [1.5; 1.9]	1.3 [1.2; 1.5]	0.99 [0.86; 1.14]	1.11 [0.93; 1.32]	0.12 [-0.13; 0.38]
Iraq	1.18 [1.04; 1.35]	1.37 [1.09; 1.72]	0.19 [-0.15; 0.59]	2.9 [1.9; 4.1]	2.1 [1.4; 3.0]	0.99 [0.85; 1.16]	1.05 [0.82; 1.32]	0.06 [-0.24; 0.38]
Ireland	1.48 [1.33; 1.64]	1.40 [1.19; 1.64]	-0.08 [-0.35; 0.22]	0.3 [0.3; 0.4]	0.2 [0.2; 0.3]	0.94 [0.85; 1.03]	0.91 [0.77; 1.07]	-0.03 [-0.20; 0.16]
Israel	1.26 [1.15; 1.37]	1.36 [1.18; 1.57]	0.10 [-0.12; 0.35]	0.4 [0.4; 0.5]	0.3 [0.3; 0.3]	1.10 [1.01; 1.20]*	0.93 [0.81; 1.08]	-0.17 [-0.33; 0.01]
Italy	1.33 [1.25; 1.41]	1.20 [1.07; 1.35]	-0.13 [-0.29; 0.04]	0.3 [0.3; 0.3]	0.2 [0.2; 0.3]	1.03 [0.97; 1.09]	1.06 [0.94; 1.19]	0.03 [-0.11; 0.18]
Jamaica	1.45 [1.27; 1.64]	1.36 [1.06; 1.72]	-0.10 [-0.47; 0.33]	1.3 [1.0; 1.6]	0.9 [0.7; 1.2]	0.99 [0.88; 1.13]	1.03 [0.82; 1.30]	0.04 [-0.23; 0.34]
Japan	1.48 [1.41; 1.55]	1.19 [1.09; 1.30]	-0.29 [-0.41; -0.16]†	0.4 [0.3; 0.4]	0.3 [0.3; 0.3]	0.93 [0.89; 0.98]*	1.06 [0.97; 1.17]	0.13 [0.02; 0.25]†
Jordan	1.45 [1.28; 1.64]	1.41 [1.10; 1.80]	-0.04 [-0.42; 0.40]	1.6 [0.8; 2.3]	1.1 [0.6; 1.7]	0.98 [0.86; 1.12]	1.00 [0.79; 1.27]	0.02 [-0.25; 0.33]
Kazakhstan	1.66 [1.57; 1.76]	1.45 [1.34; 1.58]	-0.21 [-0.36; -0.06]†	1.4 [1.4; 1.5]	1.0 [0.9; 1.0]	0.84 [0.79; 0.89]*	0.97 [0.89; 1.05]	0.14 [0.04; 0.23]†
Kenya	1.07 [0.95; 1.20]	1.16 [0.93; 1.44]	0.09 [-0.20; 0.41]	5.3 [3.4; 7.3]	4.6 [2.9; 6.4]	0.97 [0.87; 1.10]	1.10 [0.84; 1.45]	0.13 [-0.18; 0.50]
Kiribati	1.07 [0.94; 1.21]	1.24 [0.96; 1.57]	0.18 [-0.16; 0.55]	5.8 [4.4; 7.5]	4.6 [3.5; 6.2]	1.00 [0.87; 1.16]	0.99 [0.75; 1.34]	-0.01 [-0.31; 0.36]
Democratic People's Republic of Korea	1.27 [1.12; 1.44]	1.44 [1.13; 1.83]	0.17 [-0.21; 0.61]	2.1 [1.6; 2.8]	1.5 [1.1; 2.0]	1.00 [0.85; 1.17]	1.00 [0.79; 1.27]	0.00 [-0.29; 0.32]
Republic of Korea	1.43 [1.29; 1.58]	1.31 [1.12; 1.52]	-0.12 [-0.37; 0.14]	0.4 [0.4; 0.4]	0.3 [0.3; 0.3]	1.01 [0.91; 1.12]	0.97 [0.84; 1.13]	-0.04 [-0.23; 0.16]
Kosovo	1.32 [1.16; 1.49]	1.39 [1.08; 1.76]	0.07 [-0.30; 0.50]	1.2 [0.9; 1.5]	0.8 [0.6; 1.1]	1.00 [0.85; 1.17]	1.00 [0.79; 1.27]	0.00 [-0.28; 0.32]
Kuwait	1.29 [1.16; 1.44]	1.35 [1.14; 1.60]	0.06 [-0.21; 0.35]	1.0 [0.9; 1.1]	0.7 [0.6; 0.8]	0.99 [0.89; 1.11]	1.01 [0.86; 1.18]	0.02 [-0.19; 0.23]
Kyrgyzstan	1.41 [1.31; 1.52]	1.67 [1.49; 1.86]	0.26 [0.04; 0.48]†	1.5 [1.4; 1.6]	0.9 [0.8; 1.0]	1.00 [0.92; 1.08]	0.85 [0.76; 0.95]*	-0.15 [-0.27; -0.02]†
Lao People's Democratic Republic	1.06 [0.94; 1.21]	1.43 [1.14; 1.78]	0.37 [0.02; 0.76]†	3.0 [1.8; 4.6]	2.1 [1.2; 3.2]	0.99 [0.87; 1.11]	0.99 [0.78; 1.26]	0.01 [-0.25; 0.31]
Latvia	1.63 [1.49; 1.79]	1.12 [0.92; 1.38]	-0.51 [-0.78; -0.21]†	0.5 [0.4; 0.6]	0.5 [0.4; 0.5]	0.86 [0.78; 0.95]*	1.13 [0.93; 1.37]	0.27 [0.04; 0.54]†
Lebanon	1.41 [1.25; 1.60]	1.40 [1.09; 1.78]	-0.01 [-0.39; 0.42]	1.2 [0.9; 1.5]	0.8 [0.6; 1.1]	1.00 [0.87; 1.14]	0.99 [0.78; 1.26]	-0.01 [-0.28; 0.31]
Lesotho	1.06 [0.94; 1.21]	1.26 [1.00; 1.57]	0.19 [-0.12; 0.55]	5.0 [3.5; 6.8]	4.0 [2.8; 5.4]	1.01 [0.88; 1.17]	1.03 [0.78; 1.34]	0.01 [-0.29; 0.37]
Liberia	1.01 [0.89; 1.15]	1.23 [0.99; 1.55]	0.23 [-0.08; 0.57]	10.3 [7.2; 13.6]	8.3 [5.8; 11.2]	1.02 [0.90; 1.15]	0.86 [0.68; 1.11]	-0.16 [-0.40; 0.14]
Libya	1.27 [1.13; 1.44]	1.41 [1.10; 1.80]	0.13 [-0.23; 0.56]	1.4 [0.4; 2.7]	1.0 [0.3; 1.9]	0.98 [0.82; 1.15]	0.98 [0.77; 1.25]	0.01 [-0.28; 0.32]
Lithuania	1.58 [1.45; 1.72]	1.22 [1.02; 1.46]	-0.36 [-0.62; -0.08]†	0.7 [0.6; 0.7]	0.5 [0.5; 0.6]	0.92 [0.84; 1.00]*	1.06 [0.90; 1.26]	0.15 [-0.04; 0.37]
Luxembourg	1.40 [1.24; 1.59]	1.28 [1.01; 1.63]	-0.12 [-0.48; 0.28]	0.2 [0.1; 0.3]	0.2 [0.1; 0.2]	0.98 [0.87; 1.10]	0.99 [0.78; 1.26]	0.01 [-0.25; 0.31]
Macedonia	1.43 [1.28; 1.60]	1.26 [1.01; 1.56]	-0.18 [-0.50; 0.18]	0.8 [0.6; 0.9]	0.6 [0.5; 0.7]	1.00 [0.90; 1.12]	1.06 [0.86; 1.30]	0.05 [-0.19; 0.33]
Madagascar	1.05 [0.94; 1.19]	1.02 [0.85; 1.22]	-0.04 [-0.26; 0.21]	13.3 [10.6; 16.2]	13.1 [10.5; 15.9]	0.98 [0.87; 1.09]	1.02 [0.85; 1.23]	0.04 [-0.18; 0.29]
Malawi	1.03 [0.92; 1.15]	1.22 [1.01; 1.46]	0.19 [-0.07; 0.47]	7.9 [6.3; 9.7]	6.5 [5.1; 8.0]	1.00 [0.90; 1.12]	0.92 [0.74; 1.14]	-0.09 [-0.31; 0.16]
Malaysia	1.39 [1.29; 1.50]	1.30 [1.19; 1.41]	-0.09 [-0.24; 0.07]	0.9 [0.8; 0.9]	0.7 [0.6; 0.7]	1.04 [0.97; 1.13]	1.04 [0.95; 1.13]	-0.01 [-0.13; 0.11]
Maldives	1.12 [0.99; 1.26]	1.26 [0.99; 1.58]	0.14 [-0.18; 0.50]	0.7 [0.5; 0.9]	0.5 [0.4; 0.7]	1.10 [0.96; 1.27]	1.04 [0.83; 1.30]	-0.06 [-0.34; 0.25]
Mali	1.05 [0.94; 1.17]	1.09 [0.89; 1.33]	0.04 [-0.20; 0.31]	13.5 [10.1; 17.0]	12.3 [9.2; 15.7]	0.99 [0.89; 1.10]	0.95 [0.77; 1.17]	-0.04 [-0.25; 0.21]
Malta	1.39 [1.23; 1.58]	1.31 [1.04; 1.66]	-0.08 [-0.43; 0.33]	0.4 [0.2; 0.6]	0.3 [0.2; 0.4]	0.94 [0.82; 1.07]	0.97 [0.77; 1.23]	0.03 [-0.22; 0.32]
Marshall Islands	1.24 [1.10; 1.41]	1.38 [1.08; 1.78]	0.14 [-0.22; 0.58]	3.8 [2.9; 5.0]	2.8 [2.1; 3.7]	1.00 [0.85; 1.17]	1.00 [0.75; 1.31]	0.00 [-0.30; 0.35]
Mauritania	1.03 [0.91; 1.16]	1.33 [1.07; 1.64]	0.30 [-0.01; 0.64]	5.3 [3.2; 6.9]	4.0 [2.4; 5.3]	1.01 [0.89; 1.14]	0.96 [0.75; 1.24]	-0.04 [-0.31; 0.27]
Mauritius	1.37 [1.22; 1.53]	1.34 [1.07; 1.67]	-0.02 [-0.36; 0.35]	0.9 [0.7; 1.1]	0.7 [0.5; 0.8]	1.04 [0.93; 1.17]	1.00 [0.81; 1.24]	-0.04 [-0.28; 0.24]
Mexico	1.26 [1.22; 1.30]	1.25 [1.16; 1.34]	-0.02 [-0.11; 0.09]	1.2 [1.1; 1.2]	0.9 [0.9; 1.0]	1.15 [1.11; 1.19]*	1.11 [1.03; 1.19]*	-0.03 [-0.12; 0.05]
Republic of Moldova	1.59 [1.46; 1.73]	1.39 [1.17; 1.64]	-0.20 [-0.47; 0.10]	1.3 [1.1; 1.4]	0.9 [0.8; 1.0]	0.88 [0.80; 0.97]*	1.00 [0.85; 1.19]	0.13 [-0.06; 0.34]
Monaco	1.37 [1.20; 1.55]	1.27 [0.99; 1.63]	-0.09 [-0.45; 0.31]	0.4 [0.3; 0.5]	0.3 [0.2; 0.4]	1.00 [0.88; 1.13]	1.00 [0.78; 1.28]	0.00 [-0.26; 0.32]
Mongolia	1.16 [1.04; 1.30]	1.57 [1.33; 1.87]	0.41 [0.11; 0.74]†	1.7 [1.6; 1.9]	1.1 [1.0; 1.2]	0.99 [0.88; 1.13]	0.91 [0.76; 1.07]	-0.08 [-0.29; 0.12]
Montenegro	1.41 [1.25; 1.60]	1.31 [1.03; 1.65]	-0.10 [-0.46; 0.29]	0.4 [0.3; 0.6]	0.3 [0.2; 0.4]	1.00 [0.89; 1.13]	0.97 [0.77; 1.23]	-0.03 [-0.28; 0.26]
Montserrat	1.45 [1.28; 1.64]	1.51 [1.19; 1.91]	0.05 [-0.34; 0.52]	0.7 [0.6; 0.9]	0.5 [0.4; 0.6]	0.99 [0.87; 1.12]	0.87 [0.70; 1.10]	-0.11 [-0.35; 0.15]
Morocco	1.18 [1.04; 1.33]	1.43 [1.14; 1.82]	0.26 [-0.09; 0.68]	1.7 [1.0; 2.4]	1.2 [0.7; 1.7]	1.01 [0.88; 1.17]	0.99 [0.78; 1.25]	-0.02 [-0.30; 0.28]
Mozambique	1.08 [0.95; 1.24]	1.16 [0.93; 1.47]	0.08 [-0.23; 0.43]	8.1 [4.4; 11.7]	6.9 [3.7; 10.1]	1.00 [0.89; 1.13]	0.97 [0.74; 1.32]	-0.03 [-0.31; 0.33]
Myanmar	1.03 [0.90; 1.17]	1.47 [1.17; 1.85]	0.44 [0.10; 0.86]†	2.6 [1.3; 4.2]	1.8 [0.9; 2.9]	1.00 [0.88; 1.14]	0.97 [0.76; 1.22]	-0.03 [-0.30; 0.26]
Namibia	1.09 [0.96; 1.23]	1.13 [0.89; 1.41]	0.04 [-0.25; 0.37]	6.9 [4.4; 9.9]	6.1 [3.8; 8.9]	0.99 [0.86; 1.14]	1.04 [0.79; 1.40]	0.06 [-0.26; 0.44]

¶: Sex ratio is outlying in 1990. §: Sex ratio is outlying in 2021. *: The ratio of estimated to expected female mortality is significantly different from one. †: Change is significantly different from zero.

Continued on next page

Table 16 – continued from previous page

	Sex ratio 5q5			Sex-specific 5q5 in 2021 (per 1,000)		Estimated/Expected female 5q5		
	1990	2021	Change 1990–2021	Male	Female	1990	2021	Change 1990–2021
Nauru	1.12 [0.99; 1.28]	1.40 [1.10; 1.80]	0.28 [-0.08; 0.70]	3.6 [2.8; 4.7]	2.6 [1.9; 3.4]	1.01 [0.87; 1.19]	1.00 [0.76; 1.29]	-0.01 [-0.31; 0.32]
Nepal	0.98 [0.88; 1.08]	1.43 [1.15; 1.79]	0.46 [0.15; 0.83]†	3.2 [2.2; 4.2]	2.2 [1.5; 2.9]	1.05 [0.95; 1.16]	0.99 [0.78; 1.25]	-0.05 [-0.30; 0.22]
Netherlands	1.42 [1.31; 1.54]	1.04 [0.90; 1.20]	-0.38 [-0.57; -0.19]†	0.3 [0.3; 0.3]	0.3 [0.3; 0.3]	0.97 [0.89; 1.05]	1.22 [1.06; 1.41]*	0.26 [0.07; 0.46]†
New Zealand	1.33 [1.21; 1.47]	1.40 [1.21; 1.61]	0.07 [-0.17; 0.32]	0.5 [0.4; 0.5]	0.3 [0.3; 0.4]	1.06 [0.96; 1.17]	0.91 [0.79; 1.05]	-0.15 [-0.32; 0.03]
Nicaragua	1.27 [1.12; 1.43]	1.39 [1.10; 1.77]	0.13 [-0.24; 0.56]	1.5 [0.6; 2.7]	1.1 [0.4; 1.9]	1.00 [0.85; 1.17]	1.00 [0.79; 1.28]	0.00 [-0.28; 0.33]
Niger	1.15 [1.03; 1.28]	0.99 [0.82; 1.20]	-0.15 [-0.38; 0.09]	20.8 [15.6; 27.3]	20.9 [15.5; 27.6]	0.97 [0.87; 1.07]	1.03 [0.86; 1.24]	0.07 [-0.15; 0.31]
Nigeria	1.05 [0.94; 1.19]	1.07 [0.91; 1.27]	0.02 [-0.20; 0.26]	13.3 [10.5; 16.1]	12.4 [9.8; 15.0]	0.98 [0.88; 1.09]	0.97 [0.81; 1.15]	-0.01 [-0.22; 0.19]
Niue	1.46 [1.29; 1.65]	1.44 [1.13; 1.84]	-0.01 [-0.40; 0.43]	3.3 [2.5; 4.3]	2.3 [1.7; 3.0]	0.97 [0.84; 1.11]	0.99 [0.76; 1.27]	0.02 [-0.26; 0.33]
Norway	1.36 [1.22; 1.51]	1.09 [0.91; 1.31]	-0.27 [-0.53; 0.00]	0.3 [0.2; 0.3]	0.3 [0.2; 0.3]	1.01 [0.92; 1.12]	1.17 [0.97; 1.40]	0.16 [-0.08; 0.42]
Oman	1.38 [1.22; 1.56]	1.41 [1.10; 1.82]	0.03 [-0.35; 0.48]	1.4 [1.1; 1.8]	1.0 [0.7; 1.3]	1.00 [0.86; 1.15]	1.00 [0.78; 1.27]	0.00 [-0.28; 0.33]
Pakistan¶	0.97 [0.88; 1.07]	1.16 [0.93; 1.44]	0.19 [-0.07; 0.50]	4.4 [3.4; 5.6]	3.8 [2.9; 4.8]	1.14 [1.02; 1.27]*	1.15 [0.89; 1.48]	0.01 [-0.30; 0.38]
Palau	1.37 [1.21; 1.55]	1.45 [1.14; 1.86]	0.09 [-0.30; 0.53]	2.2 [1.7; 2.9]	1.5 [1.1; 2.0]	0.97 [0.83; 1.13]	0.99 [0.78; 1.26]	0.02 [-0.26; 0.34]
Panama	1.39 [1.25; 1.56]	1.21 [1.05; 1.42]	-0.17 [-0.42; 0.09]	1.5 [1.4; 1.7]	1.3 [1.1; 1.4]	1.04 [0.93; 1.16]	1.17 [1.00; 1.35]	0.13 [-0.09; 0.35]
Papua New Guinea	1.08 [0.95; 1.23]	1.23 [0.97; 1.57]	0.15 [-0.16; 0.53]	5.1 [3.9; 6.7]	4.2 [3.1; 5.5]	1.00 [0.87; 1.17]	1.03 [0.78; 1.37]	0.03 [-0.27; 0.40]
Paraguay	1.32 [1.16; 1.50]	1.43 [1.12; 1.81]	0.11 [-0.27; 0.54]	1.0 [0.4; 1.8]	0.7 [0.3; 1.3]	0.97 [0.83; 1.13]	0.95 [0.75; 1.20]	-0.03 [-0.31; 0.28]
Peru	1.21 [1.09; 1.36]	1.40 [1.12; 1.74]	0.18 [-0.15; 0.56]	1.4 [1.1; 1.9]	1.0 [0.8; 1.4]	0.97 [0.85; 1.11]	1.01 [0.81; 1.25]	0.04 [-0.22; 0.32]
Philippines	1.30 [1.17; 1.44]	1.34 [1.14; 1.56]	0.04 [-0.22; 0.31]	2.6 [2.5; 2.8]	2.0 [1.8; 2.2]	0.99 [0.88; 1.11]	1.09 [0.93; 1.27]	0.10 [-0.12; 0.32]
Poland	1.46 [1.38; 1.54]	1.19 [1.07; 1.33]	-0.27 [-0.41; -0.11]†	0.4 [0.4; 0.5]	0.4 [0.3; 0.4]	0.97 [0.92; 1.03]	1.06 [0.95; 1.19]	0.09 [-0.04; 0.23]
Portugal	1.47 [1.37; 1.58]	1.09 [0.93; 1.26]	-0.38 [-0.58; -0.18]†	0.4 [0.3; 0.4]	0.3 [0.3; 0.4]	0.98 [0.91; 1.06]	1.17 [1.01; 1.36]*	0.19 [0.00; 0.40]†
Qatar	1.46 [1.29; 1.66]	1.26 [1.02; 1.55]	-0.20 [-0.53; 0.15]	0.8 [0.6; 0.9]	0.6 [0.5; 0.7]	0.98 [0.87; 1.11]	1.05 [0.86; 1.29]	0.07 [-0.17; 0.34]
Romania	1.47 [1.39; 1.56]	1.32 [1.18; 1.48]	-0.15 [-0.32; 0.02]	0.8 [0.7; 0.9]	0.6 [0.5; 0.7]	0.96 [0.90; 1.02]	1.01 [0.91; 1.13]	0.06 [-0.07; 0.19]
Russian Federation	1.85 [1.79; 1.91]	1.36 [1.25; 1.46]	-0.50 [-0.61; -0.38]†	0.8 [0.8; 0.9]	0.6 [0.6; 0.7]	0.76 [0.73; 0.79]*	0.99 [0.92; 1.07]	0.23 [0.15; 0.31]†
Rwanda	1.12 [1.01; 1.24]	1.17 [0.96; 1.43]	0.05 [-0.21; 0.35]	9.2 [7.0; 11.5]	7.9 [5.9; 10.0]	0.97 [0.88; 1.07]	0.93 [0.74; 1.17]	-0.05 [-0.27; 0.22]
Saint Kitts and Nevis	1.50 [1.33; 1.70]	1.64 [1.30; 2.09]	0.14 [-0.28; 0.64]	1.6 [1.2; 2.0]	1.0 [0.7; 1.3]	0.96 [0.84; 1.08]	0.86 [0.68; 1.08]	-0.10 [-0.33; 0.17]
Saint Lucia	1.45 [1.28; 1.65]	1.45 [1.14; 1.84]	-0.01 [-0.39; 0.44]	2.0 [1.4; 2.6]	1.3 [0.9; 1.9]	0.99 [0.87; 1.12]	0.99 [0.78; 1.25]	0.00 [-0.26; 0.30]
Samoa	1.31 [1.15; 1.49]	1.38 [1.09; 1.74]	0.07 [-0.30; 0.49]	1.4 [0.7; 2.3]	1.0 [0.5; 1.7]	1.03 [0.87; 1.21]	1.01 [0.80; 1.28]	-0.02 [-0.30; 0.31]
San Marino	1.41 [1.24; 1.59]	1.27 [1.00; 1.63]	-0.13 [-0.48; 0.28]	0.3 [0.2; 0.3]	0.2 [0.1; 0.3]	1.01 [0.90; 1.14]	1.00 [0.78; 1.27]	-0.01 [-0.28; 0.29]
Sao Tome and Principe	1.05 [0.92; 1.19]	1.32 [1.05; 1.68]	0.28 [-0.06; 0.67]	1.7 [1.3; 2.2]	1.3 [1.0; 1.7]	0.99 [0.87; 1.13]	1.08 [0.85; 1.35]	0.09 [-0.19; 0.41]
Saudi Arabia	1.35 [1.18; 1.53]	1.36 [1.06; 1.75]	0.02 [-0.35; 0.45]	1.0 [0.8; 1.3]	0.7 [0.5; 1.0]	1.00 [0.85; 1.17]	1.00 [0.79; 1.27]	0.00 [-0.29; 0.32]
Senegal	1.04 [0.94; 1.15]	1.52 [1.26; 1.83]	0.48 [0.19; 0.82]†	5.3 [4.2; 6.5]	3.5 [2.7; 4.3]	0.99 [0.90; 1.10]	0.83 [0.67; 1.04]	-0.16 [-0.36; 0.07]
Serbia	1.36 [1.23; 1.51]	1.36 [1.13; 1.63]	-0.01 [-0.30; 0.31]	0.6 [0.5; 0.6]	0.4 [0.4; 0.5]	1.05 [0.95; 1.16]	0.94 [0.79; 1.12]	-0.11 [-0.31; 0.11]
Seychelles	1.46 [1.30; 1.66]	1.52 [1.20; 1.91]	0.06 [-0.35; 0.51]	1.2 [0.7; 1.7]	0.8 [0.5; 1.2]	0.99 [0.87; 1.11]	0.91 [0.73; 1.15]	-0.08 [-0.32; 0.21]
Sierra Leone	1.06 [0.93; 1.21]	0.95 [0.79; 1.13]	-0.12 [-0.35; 0.12]	14.9 [10.9; 19.0]	15.7 [11.6; 20.1]	0.98 [0.87; 1.11]	1.09 [0.91; 1.32]	0.11 [-0.13; 0.37]
Singapore	1.36 [1.21; 1.53]	1.16 [0.93; 1.44]	-0.20 [-0.51; 0.14]	0.3 [0.2; 0.3]	0.3 [0.2; 0.3]	1.02 [0.91; 1.14]	1.10 [0.88; 1.37]	0.08 [-0.19; 0.39]
Slovakia	1.38 [1.27; 1.51]	1.23 [1.05; 1.46]	-0.15 [-0.39; 0.11]	0.6 [0.6; 0.7]	0.5 [0.5; 0.6]	1.03 [0.94; 1.12]	1.05 [0.90; 1.22]	0.03 [-0.16; 0.23]
Slovenia	1.36 [1.22; 1.53]	1.17 [0.93; 1.48]	-0.19 [-0.50; 0.16]	0.3 [0.3; 0.4]	0.3 [0.2; 0.3]	1.03 [0.93; 1.15]	1.08 [0.86; 1.36]	0.05 [-0.21; 0.36]
Solomon Islands	1.31 [1.15; 1.48]	1.42 [1.11; 1.80]	0.11 [-0.26; 0.54]	2.5 [2.0; 3.3]	1.8 [1.4; 2.4]	1.00 [0.85; 1.16]	1.02 [0.80; 1.29]	0.02 [-0.27; 0.35]
Somalia	1.03 [0.91; 1.17]	1.02 [0.80; 1.30]	-0.01 [-0.29; 0.32]	15.8 [11.9; 20.8]	15.5 [11.8; 20.4]	1.01 [0.89; 1.13]	1.01 [0.79; 1.29]	0.00 [-0.28; 0.32]
South Africa	1.25 [1.10; 1.41]	1.39 [1.20; 1.62]	0.15 [-0.12; 0.42]	2.9 [2.6; 3.3]	2.1 [1.9; 2.4]	1.00 [0.86; 1.17]	1.04 [0.89; 1.20]	0.04 [-0.20; 0.26]
South Sudan	1.07 [0.94; 1.22]	1.04 [0.82; 1.33]	-0.03 [-0.32; 0.30]	14.0 [10.6; 18.4]	13.5 [10.1; 17.6]	1.00 [0.88; 1.13]	0.99 [0.77; 1.28]	0.00 [-0.27; 0.31]
Spain	1.38 [1.30; 1.47]	1.18 [1.04; 1.32]	-0.20 [-0.36; -0.03]†	0.3 [0.3; 0.4]	0.3 [0.3; 0.3]	1.01 [0.95; 1.08]	1.08 [0.96; 1.22]	0.06 [-0.08; 0.22]
Sri Lanka	1.20 [1.09; 1.33]	1.21 [1.01; 1.47]	0.01 [-0.25; 0.30]	0.7 [0.6; 0.9]	0.6 [0.5; 0.7]	1.16 [1.04; 1.30]*	1.08 [0.91; 1.30]	-0.08 [-0.32; 0.18]
Saint Vincent and the Grenadines	1.44 [1.27; 1.64]	1.53 [1.21; 1.94]	0.09 [-0.32; 0.55]	1.8 [1.1; 2.7]	1.2 [0.7; 1.8]	1.00 [0.88; 1.14]	0.93 [0.74; 1.17]	-0.07 [-0.33; 0.22]
State of Palestine	1.38 [1.22; 1.56]	1.37 [1.08; 1.73]	-0.02 [-0.38; 0.39]	1.9 [1.2; 2.8]	1.4 [0.9; 2.1]	1.01 [0.88; 1.16]	1.04 [0.83; 1.32]	0.03 [-0.24; 0.36]
Sudan	0.99 [0.89; 1.12]	1.24 [0.99; 1.54]	0.24 [-0.05; 0.58]	5.3 [3.4; 7.0]	4.3 [2.7; 5.8]	1.03 [0.92; 1.16]	1.03 [0.78; 1.35]	0.00 [-0.29; 0.36]
Suriname	1.43 [1.27; 1.61]	1.00 [0.84; 1.18]	-0.43 [-0.68; -0.17]†	1.9 [1.6; 2.2]	1.9 [1.6; 2.2]	1.01 [0.89; 1.14]	1.43 [1.22; 1.69]*	0.42 [0.16; 0.71]†

¶: Sex ratio is outlying in 1990. §: Sex ratio is outlying in 2021. *: The ratio of estimated to expected female mortality is significantly different from one. †: Change is significantly different from zero.

Continued on next page

Table 16 – continued from previous page

	Sex ratio 5q5			Sex-specific 5q5 in 2021 (per 1,000)		Estimated/Expected female 5q5		
	1990	2021	Change 1990–2021	Male	Female	1990	2021	Change 1990–2021
Eswatini	1.24 [1.10; 1.41]	1.11 [0.87; 1.43]	-0.14 [-0.44; 0.23]	10.0 [3.4; 12.3]	9.0 [3.0; 11.2]	1.00 [0.79; 1.24]	1.01 [0.76; 1.41]	0.01 [-0.38; 0.53]
Sweden	1.41 [1.28; 1.56]	1.10 [0.94; 1.30]	-0.31 [-0.55; -0.06]†	0.3 [0.3; 0.3]	0.3 [0.2; 0.3]	0.95 [0.86; 1.04]	1.15 [0.98; 1.35]	0.21 [0.00; 0.43]
Switzerland	1.43 [1.30; 1.58]	1.17 [0.99; 1.37]	-0.27 [-0.52; 0.00]†	0.3 [0.3; 0.4]	0.3 [0.3; 0.3]	0.96 [0.88; 1.06]	1.09 [0.92; 1.28]	0.12 [-0.08; 0.35]
Syria	1.17 [1.04; 1.31]	1.20 [1.00; 1.43]	0.03 [-0.22; 0.31]	2.8 [2.0; 3.5]	2.3 [1.7; 3.0]	1.03 [0.90; 1.17]	1.11 [0.93; 1.32]	0.08 [-0.16; 0.34]
Tajikistan	1.43 [1.33; 1.53]	1.35 [1.22; 1.49]	-0.08 [-0.25; 0.09]	1.2 [1.1; 1.2]	0.9 [0.8; 0.9]	0.89 [0.82; 0.97]*	1.03 [0.93; 1.13]	0.14 [0.01; 0.27]†
Tanzania	1.05 [0.94; 1.17]	1.03 [0.83; 1.28]	-0.02 [-0.27; 0.26]	9.1 [6.5; 11.2]	8.8 [6.4; 11.0]	0.98 [0.87; 1.09]	1.06 [0.84; 1.36]	0.08 [-0.18; 0.41]
Thailand	1.38 [1.24; 1.53]	1.48 [1.26; 1.74]	0.11 [-0.18; 0.40]	2.0 [1.9; 2.2]	1.4 [1.2; 1.5]	0.99 [0.88; 1.11]	0.97 [0.83; 1.13]	-0.02 [-0.21; 0.19]
Timor Leste	1.01 [0.89; 1.15]	1.21 [0.95; 1.54]	0.20 [-0.11; 0.56]	6.0 [4.6; 7.7]	4.9 [3.7; 6.4]	1.02 [0.90; 1.15]	1.01 [0.76; 1.33]	-0.01 [-0.31; 0.35]
Togo	1.07 [0.95; 1.21]	1.04 [0.84; 1.30]	-0.03 [-0.29; 0.26]	6.9 [4.6; 9.3]	6.6 [4.4; 9.0]	0.96 [0.86; 1.08]	1.12 [0.86; 1.48]	0.16 [-0.14; 0.54]
Tonga	1.42 [1.25; 1.61]	1.54 [1.23; 1.93]	0.12 [-0.27; 0.57]	1.6 [1.0; 2.2]	1.1 [0.7; 1.5]	1.01 [0.89; 1.14]	0.92 [0.74; 1.15]	-0.09 [-0.33; 0.18]
Trinidad and Tobago	1.36 [1.22; 1.51]	1.32 [1.10; 1.56]	-0.05 [-0.32; 0.25]	1.0 [0.8; 1.2]	0.8 [0.6; 0.9]	1.06 [0.95; 1.18]	1.04 [0.88; 1.23]	-0.02 [-0.23; 0.21]
Tunisia	1.30 [1.16; 1.46]	1.35 [1.11; 1.63]	0.05 [-0.25; 0.38]	1.8 [1.5; 2.0]	1.3 [1.1; 1.5]	1.03 [0.91; 1.17]	1.06 [0.88; 1.28]	0.02 [-0.22; 0.29]
Turkey	1.14 [1.00; 1.29]	1.24 [1.12; 1.36]	0.10 [-0.09; 0.28]	0.8 [0.8; 0.9]	0.7 [0.6; 0.7]	0.99 [0.86; 1.13]	1.09 [0.99; 1.19]	0.10 [-0.08; 0.26]
Turkmenistan	1.29 [1.16; 1.42]	1.39 [1.16; 1.67]	0.10 [-0.19; 0.42]	2.1 [1.7; 2.4]	1.5 [1.2; 1.8]	1.03 [0.92; 1.15]	1.03 [0.86; 1.24]	0.01 [-0.22; 0.25]
Turks and Caicos Islands	1.52 [1.34; 1.72]	1.25 [0.97; 1.59]	-0.27 [-0.63; 0.12]	0.5 [0.4; 0.7]	0.4 [0.3; 0.6]	0.93 [0.82; 1.05]	1.03 [0.81; 1.30]	0.10 [-0.15; 0.41]
Tuvalu	1.21 [1.07; 1.37]	1.49 [1.18; 1.89]	0.28 [-0.09; 0.72]	2.9 [2.2; 3.8]	2.0 [1.5; 2.6]	0.99 [0.84; 1.17]	0.97 [0.75; 1.23]	-0.03 [-0.31; 0.29]
Uganda	1.03 [0.92; 1.16]	1.08 [0.88; 1.33]	0.05 [-0.21; 0.33]	8.2 [5.6; 10.8]	7.5 [5.1; 10.0]	0.99 [0.89; 1.11]	1.03 [0.81; 1.34]	0.04 [-0.23; 0.38]
Ukraine	1.67 [1.59; 1.75]	1.26 [1.16; 1.38]	-0.40 [-0.54; -0.26]†	0.8 [0.8; 0.9]	0.7 [0.6; 0.7]	0.87 [0.82; 0.91]*	1.06 [0.97; 1.16]	0.19 [0.09; 0.30]†
United Arab Emirates	1.43 [1.27; 1.63]	1.35 [1.06; 1.73]	-0.08 [-0.46; 0.34]	0.9 [0.7; 1.2]	0.7 [0.5; 0.9]	1.00 [0.88; 1.13]	1.00 [0.79; 1.27]	0.00 [-0.26; 0.31]
United Kingdom	1.39 [1.31; 1.47]	1.24 [1.11; 1.37]	-0.16 [-0.31; 0.00]	0.3 [0.3; 0.4]	0.3 [0.2; 0.3]	0.98 [0.93; 1.04]	1.03 [0.93; 1.14]	0.04 [-0.08; 0.18]
United States of America	1.37 [1.32; 1.41]	1.19 [1.10; 1.30]	-0.17 [-0.28; -0.05]†	0.6 [0.6; 0.6]	0.5 [0.5; 0.5]	1.03 [0.99; 1.07]	1.08 [0.99; 1.17]	0.05 [-0.04; 0.16]
Uruguay	1.43 [1.29; 1.58]	1.13 [0.95; 1.36]	-0.29 [-0.55; -0.01]†	0.7 [0.6; 0.8]	0.6 [0.5; 0.7]	1.00 [0.90; 1.11]	1.15 [0.97; 1.36]	0.15 [-0.07; 0.39]
Uzbekistan	1.36 [1.23; 1.50]	1.33 [1.16; 1.52]	-0.03 [-0.26; 0.20]	1.7 [1.6; 1.8]	1.3 [1.2; 1.4]	0.99 [0.89; 1.11]	1.07 [0.94; 1.22]	0.08 [-0.10; 0.27]
Vanuatu	1.33 [1.17; 1.51]	1.42 [1.11; 1.81]	0.09 [-0.29; 0.52]	3.1 [2.4; 4.0]	2.2 [1.6; 2.9]	1.00 [0.85; 1.16]	1.01 [0.78; 1.29]	0.01 [-0.28; 0.34]
Venezuela (Bolivarian Republic of)	1.43 [1.29; 1.58]	1.30 [1.08; 1.55]	-0.13 [-0.42; 0.17]	1.7 [1.5; 2.0]	1.3 [1.1; 1.5]	1.01 [0.92; 1.12]	1.10 [0.92; 1.31]	0.08 [-0.14; 0.33]
Vietnam	1.13 [0.99; 1.28]	1.68 [1.37; 2.06]	0.55 [0.20; 0.97]†	1.4 [1.1; 1.9]	0.8 [0.6; 1.1]	0.98 [0.85; 1.13]	0.84 [0.68; 1.02]	-0.14 [-0.37; 0.09]
Yemen	1.06 [0.95; 1.19]	1.25 [0.98; 1.58]	0.18 [-0.13; 0.55]	4.9 [2.9; 6.5]	3.9 [2.3; 5.3]	0.98 [0.87; 1.10]	0.99 [0.76; 1.28]	0.01 [-0.27; 0.34]
Zambia	1.01 [0.91; 1.13]	1.18 [0.96; 1.45]	0.17 [-0.10; 0.47]	7.0 [3.7; 9.4]	5.9 [3.1; 8.0]	1.01 [0.91; 1.13]	1.00 [0.77; 1.33]	-0.01 [-0.28; 0.35]
Zimbabwe	1.06 [0.94; 1.20]	1.21 [0.98; 1.50]	0.14 [-0.14; 0.47]	4.9 [2.6; 6.8]	4.1 [2.2; 5.6]	1.03 [0.90; 1.18]	1.08 [0.82; 1.39]	0.05 [-0.26; 0.40]

Table 17: **Estimates and 90% uncertainty intervals for sex ratios for 5q10 in 1990 and 2021, the change in sex ratios from 1990 to 2021, sex-specific 5q10 in 2021, and ratios of estimated to expected female 5q10 and their change from 1990 to 2021 for the world, UNICEF regions, and all countries.** ¶: Sex ratio is outlying in 1990. §: Sex ratio is outlying in 2021. *: The ratio of estimated to expected female mortality is significantly different from one. †: Change is significantly different from zero.

	Sex ratio 5q10			Sex-specific 5q10 in 2021 (per 1,000)		Estimated/Expected female 5q10		
	1990	2021	Change 1990–2021	Male	Female	1990	2021	Change 1990–2021
World	1.11 [1.06; 1.15]	1.22 [1.14; 1.28]	0.11 [0.01; 0.19]†	2.9 [2.8; 3.6]	2.4 [2.2; 3.0]	1.01 [0.96; 1.06]	0.99 [0.92; 1.05]	-0.02 [-0.08; 0.05]
South Asia	0.98 [0.90; 1.05]	1.30 [1.15; 1.48]	0.33 [0.15; 0.52]†	2.8 [2.2; 3.7]	2.1 [1.7; 3.0]	1.06 [0.97; 1.17]	1.13 [0.95; 1.31]	0.07 [-0.15; 0.28]
Europe and Central Asia	1.67 [1.62; 1.70]	1.41 [1.36; 1.45]	-0.26 [-0.31; -0.20]†	0.9 [0.9; 0.9]	0.6 [0.6; 0.7]	0.91 [0.87; 0.94]*	1.05 [1.02; 1.08]*	0.14 [0.10; 0.19]†
Middle East and North Africa	1.15 [1.09; 1.22]	1.54 [1.41; 1.67]	0.38 [0.23; 0.53]†	2.1 [1.8; 3.1]	1.4 [1.1; 2.0]	0.98 [0.87; 1.07]	0.95 [0.78; 1.07]	-0.03 [-0.22; 0.13]
Sub-Saharan Africa	1.00 [0.94; 1.07]	1.11 [1.03; 1.19]	0.10 [0.00; 0.21]	6.4 [5.6; 8.8]	5.8 [5.0; 7.9]	1.00 [0.96; 1.05]	0.95 [0.87; 1.04]	-0.05 [-0.14; 0.05]
Latin America and Caribbean	1.45 [1.40; 1.51]	1.38 [1.30; 1.46]	-0.07 [-0.17; 0.03]	1.7 [1.6; 2.0]	1.3 [1.2; 1.5]	0.99 [0.95; 1.04]	1.07 [0.98; 1.15]	0.08 [-0.02; 0.17]
East Asia and Pacific	1.36 [1.23; 1.47]	1.57 [1.40; 1.75]	0.21 [0.00; 0.45]	1.6 [1.2; 2.3]	1.0 [0.8; 1.5]	1.00 [0.83; 1.11]	0.93 [0.80; 1.06]	-0.07 [-0.24; 0.14]
North America	1.63 [1.58; 1.69]	1.39 [1.28; 1.51]	-0.24 [-0.37; -0.11]†	0.9 [0.8; 1.0]	0.6 [0.6; 0.7]	0.97 [0.94; 1.01]	1.03 [0.95; 1.11]	0.06 [-0.03; 0.15]
Afghanistan	1.01 [0.88; 1.15]	1.78 [1.41; 2.26]	0.77 [0.36; 1.28]†	2.2 [0.3; 7.0]	1.2 [0.2; 3.9]	1.04 [0.86; 1.61]	0.79 [0.52; 1.06]	-0.25 [-0.88; 0.08]
Albania	1.56 [1.42; 1.71]	1.20 [1.00; 1.44]	-0.36 [-0.62; -0.07]†	1.1 [0.8; 1.4]	0.9 [0.7; 1.2]	0.99 [0.89; 1.10]	1.24 [1.04; 1.47]*	0.25 [0.01; 0.51]†
Algeria	1.36 [1.23; 1.50]	1.29 [1.18; 1.41]	-0.07 [-0.25; 0.10]	1.6 [1.5; 1.8]	1.2 [1.1; 1.4]	0.96 [0.81; 1.14]	1.23 [1.13; 1.34]*	0.27 [0.06; 0.46]†
Andorra	1.59 [1.39; 1.82]	1.27 [0.98; 1.65]	-0.32 [-0.72; 0.13]	0.5 [0.3; 0.6]	0.4 [0.3; 0.5]	1.00 [0.87; 1.13]	1.01 [0.79; 1.31]	0.02 [-0.26; 0.35]
Angola	1.03 [0.88; 1.20]	1.00 [0.78; 1.29]	-0.03 [-0.33; 0.31]	6.3 [4.7; 8.2]	6.3 [4.7; 8.2]	1.00 [0.87; 1.15]	1.06 [0.80; 1.47]	0.06 [-0.26; 0.51]
Anguilla	1.60 [1.40; 1.83]	1.41 [1.09; 1.84]	-0.18 [-0.61; 0.31]	0.8 [0.6; 1.1]	0.6 [0.4; 0.8]	1.00 [0.87; 1.14]	1.00 [0.78; 1.27]	0.00 [-0.28; 0.31]
Antigua and Barbuda	1.60 [1.40; 1.82]	1.49 [1.15; 1.93]	-0.10 [-0.54; 0.40]	1.1 [0.8; 1.4]	0.7 [0.5; 1.0]	1.00 [0.87; 1.14]	1.00 [0.79; 1.27]	0.00 [-0.27; 0.32]
Argentina	1.61 [1.52; 1.70]	1.22 [1.11; 1.35]	-0.38 [-0.53; -0.23]†	0.9 [0.8; 1.1]	0.8 [0.6; 0.9]	1.00 [0.94; 1.05]	1.18 [1.07; 1.29]*	0.18 [0.06; 0.31]†
Armenia	1.82 [1.63; 2.02]	1.49 [1.25; 1.77]	-0.33 [-0.66; 0.01]	1.1 [0.9; 1.4]	0.8 [0.6; 1.0]	0.88 [0.79; 0.98]*	1.01 [0.85; 1.20]	0.13 [-0.06; 0.35]
Australia	1.56 [1.44; 1.68]	1.25 [1.11; 1.43]	-0.31 [-0.51; -0.09]†	0.5 [0.4; 0.5]	0.4 [0.3; 0.4]	0.97 [0.91; 1.05]	1.03 [0.90; 1.17]	0.06 [-0.10; 0.22]
Austria	1.51 [1.37; 1.66]	1.26 [1.07; 1.49]	-0.25 [-0.51; 0.04]	0.5 [0.4; 0.6]	0.4 [0.3; 0.5]	0.99 [0.91; 1.09]	1.02 [0.86; 1.21]	0.03 [-0.17; 0.24]
Azerbaijan	1.48 [1.37; 1.61]	1.45 [1.30; 1.62]	-0.03 [-0.23; 0.18]	1.9 [1.7; 2.1]	1.3 [1.2; 1.5]	1.07 [0.98; 1.17]	1.10 [0.98; 1.23]	0.03 [-0.13; 0.19]
Bahamas	1.60 [1.40; 1.83]	1.53 [1.19; 1.98]	-0.06 [-0.49; 0.44]	1.5 [0.8; 2.4]	1.0 [0.5; 1.6]	0.99 [0.85; 1.13]	1.00 [0.78; 1.29]	0.02 [-0.26; 0.34]
Bahrain	1.58 [1.39; 1.80]	1.42 [1.19; 1.68]	-0.17 [-0.50; 0.18]	1.1 [0.7; 1.6]	0.8 [0.5; 1.2]	1.01 [0.88; 1.14]	1.05 [0.88; 1.25]	0.04 [-0.19; 0.30]
Bangladesh	0.99 [0.88; 1.11]	1.42 [1.18; 1.71]	0.43 [0.15; 0.75]†	2.4 [1.4; 3.6]	1.7 [1.0; 2.6]	1.02 [0.90; 1.21]	1.10 [0.89; 1.33]	0.08 [-0.22; 0.36]
Barbados	1.58 [1.38; 1.80]	1.66 [1.29; 2.14]	0.08 [-0.38; 0.62]	1.4 [0.7; 2.5]	0.9 [0.4; 1.5]	0.98 [0.83; 1.13]	0.92 [0.71; 1.19]	-0.06 [-0.34; 0.26]
Belarus	1.76 [1.63; 1.90]	1.15 [0.98; 1.35]	-0.61 [-0.83; -0.37]†	0.6 [0.4; 0.8]	0.5 [0.4; 0.7]	0.91 [0.84; 0.98]*	1.15 [0.98; 1.35]	0.24 [0.06; 0.45]†
Belgium	1.42 [1.30; 1.55]	1.29 [1.10; 1.51]	-0.13 [-0.37; 0.13]	0.5 [0.4; 0.6]	0.4 [0.3; 0.4]	1.07 [0.99; 1.17]	1.00 [0.86; 1.18]	-0.07 [-0.25; 0.13]
Belize	1.56 [1.37; 1.78]	1.54 [1.21; 1.96]	-0.02 [-0.44; 0.47]	1.9 [1.0; 2.9]	1.2 [0.7; 1.9]	1.00 [0.84; 1.16]	1.02 [0.80; 1.30]	0.02 [-0.27; 0.37]
Benin	0.99 [0.86; 1.14]	1.09 [0.87; 1.37]	0.10 [-0.19; 0.42]	7.0 [1.8; 14.2]	6.4 [1.7; 13.1]	1.00 [0.87; 1.19]	0.99 [0.75; 1.56]	-0.01 [-0.35; 0.57]
Bhutan	1.58 [1.38; 1.81]	1.15 [0.89; 1.50]	-0.43 [-0.80; 0.00]†	5.1 [1.3; 10.9]	4.4 [1.2; 9.4]	0.72 [0.56; 1.06]	1.04 [0.71; 1.59]	0.32 [-0.20; 0.93]
Bolivia (Plurinational State of)	1.18 [1.03; 1.35]	1.56 [1.21; 2.00]	0.37 [-0.04; 0.86]	2.4 [0.5; 5.8]	1.5 [0.4; 3.7]	1.03 [0.81; 1.41]	0.95 [0.65; 1.27]	-0.08 [-0.59; 0.33]
Bosnia and Herzegovina	1.62 [1.45; 1.82]	1.21 [1.03; 1.42]	-0.40 [-0.69; -0.12]†	0.7 [0.5; 1.0]	0.6 [0.4; 0.8]	0.97 [0.87; 1.09]	1.13 [0.97; 1.33]	0.16 [-0.05; 0.39]
Botswana	1.04 [0.90; 1.19]	1.50 [1.17; 1.93]	0.47 [0.07; 0.94]†	3.4 [0.8; 7.8]	2.3 [0.5; 5.2]	1.04 [0.85; 1.57]	0.91 [0.60; 1.28]	-0.13 [-0.77; 0.30]
Brazil	1.62 [1.46; 1.80]	1.50 [1.29; 1.74]	-0.12 [-0.40; 0.18]	1.5 [1.4; 1.7]	1.0 [0.9; 1.1]	0.98 [0.88; 1.09]	1.05 [0.91; 1.21]	0.07 [-0.11; 0.27]
British Virgin Islands	1.60 [1.40; 1.83]	1.58 [1.21; 2.05]	-0.02 [-0.48; 0.52]	1.5 [1.2; 2.0]	1.0 [0.7; 1.3]	1.00 [0.87; 1.14]	0.99 [0.77; 1.29]	0.00 [-0.28; 0.33]
Brunei	1.59 [1.39; 1.82]	1.54 [1.20; 1.97]	-0.05 [-0.48; 0.44]	1.1 [0.5; 1.9]	0.7 [0.3; 1.2]	0.99 [0.84; 1.13]	0.96 [0.75; 1.24]	-0.02 [-0.29; 0.30]
Bulgaria	1.62 [1.50; 1.75]	1.42 [1.22; 1.65]	-0.20 [-0.44; 0.07]	1.0 [0.8; 1.2]	0.7 [0.6; 0.9]	0.99 [0.91; 1.07]	1.03 [0.89; 1.19]	0.04 [-0.12; 0.22]
Burkina Faso	0.99 [0.87; 1.13]	1.10 [0.85; 1.41]	0.11 [-0.19; 0.46]	5.8 [1.0; 17.2]	5.3 [0.9; 15.7]	1.03 [0.88; 1.54]	1.01 [0.73; 1.61]	-0.02 [-0.53; 0.59]
Burundi	0.99 [0.83; 1.18]	1.08 [0.85; 1.38]	0.09 [-0.23; 0.45]	8.3 [1.5; 26.1]	7.7 [1.4; 24.1]	1.01 [0.88; 1.19]	0.99 [0.73; 1.57]	-0.02 [-0.36; 0.58]
Cambodia	1.00 [0.88; 1.15]	1.49 [1.15; 1.92]	0.49 [0.10; 0.95]†	2.0 [0.4; 5.6]	1.3 [0.2; 3.8]	1.03 [0.87; 1.59]	0.98 [0.68; 1.32]	-0.05 [-0.70; 0.33]
Cameroon	0.99 [0.86; 1.13]	1.15 [0.90; 1.46]	0.16 [-0.15; 0.51]	8.4 [2.1; 16.7]	7.4 [1.8; 14.6]	1.01 [0.87; 1.22]	0.92 [0.69; 1.42]	-0.10 [-0.42; 0.43]
Canada	1.56 [1.46; 1.67]	1.20 [1.07; 1.35]	-0.36 [-0.53; -0.18]†	0.6 [0.5; 0.7]	0.5 [0.4; 0.6]	0.99 [0.93; 1.06]	1.09 [0.97; 1.22]	0.09 [-0.04; 0.24]

¶: Sex ratio is outlying in 1990. §: Sex ratio is outlying in 2021. *: The ratio of estimated to expected female mortality is significantly different from one. †: Change is significantly different from zero.

Continued on next page

Table 17 – continued from previous page

	Sex ratio 5q10			Sex-specific 5q10 in 2021 (per 1,000)		Estimated/Expected female 5q10		
	1990	2021	Change 1990–2021	Male	Female	1990	2021	Change 1990–2021
Cape Verde	1.47 [1.29; 1.68]	1.49 [1.21; 1.82]	0.02 [-0.35; 0.41]	1.2 [0.6; 1.8]	0.8 [0.4; 1.2]	1.00 [0.79; 1.19]	1.01 [0.81; 1.24]	0.01 [-0.27; 0.33]
Central African Republic	1.00 [0.87; 1.14]	0.89 [0.69; 1.13]	-0.11 [-0.36; 0.17]	9.9 [2.3; 22.1]	11.2 [2.6; 24.6]	1.00 [0.87; 1.29]	1.17 [0.88; 1.79]	0.16 [-0.23; 0.79]
Chad	0.99 [0.86; 1.14]	1.00 [0.80; 1.24]	0.01 [-0.25; 0.29]	10.5 [4.3; 18.1]	10.5 [4.2; 18.2]	1.02 [0.87; 1.41]	1.01 [0.80; 1.39]	-0.01 [-0.43; 0.41]
Chile	1.61 [1.50; 1.73]	1.23 [1.08; 1.42]	-0.38 [-0.58; -0.16]†	0.8 [0.7; 0.9]	0.6 [0.5; 0.7]	0.99 [0.93; 1.06]	1.13 [0.99; 1.29]	0.14 [-0.02; 0.31]
China	1.54 [1.35; 1.76]	1.63 [1.29; 2.04]	0.09 [-0.33; 0.57]	1.1 [0.6; 2.1]	0.7 [0.4; 1.3]	0.98 [0.73; 1.15]	0.92 [0.72; 1.16]	-0.06 [-0.34; 0.28]
Colombia	1.61 [1.43; 1.81]	1.45 [1.24; 1.68]	-0.16 [-0.47; 0.15]	1.5 [1.4; 1.7]	1.1 [0.9; 1.2]	0.96 [0.83; 1.09]	1.09 [0.94; 1.27]	0.14 [-0.07; 0.36]
Comoros	1.17 [1.01; 1.34]	1.59 [1.24; 2.06]	0.42 [0.01; 0.93]†	2.1 [0.3; 5.6]	1.3 [0.2; 3.5]	1.00 [0.77; 1.46]	0.91 [0.63; 1.23]	-0.08 [-0.65; 0.33]
Congo	1.01 [0.88; 1.17]	1.43 [1.12; 1.86]	0.42 [0.05; 0.88]†	3.8 [0.9; 8.9]	2.6 [0.6; 6.2]	1.02 [0.85; 1.56]	0.92 [0.60; 1.33]	-0.10 [-0.75; 0.36]
Democratic Republic of the Congo	0.98 [0.85; 1.13]	1.04 [0.81; 1.32]	0.05 [-0.23; 0.37]	9.4 [3.2; 17.8]	9.1 [3.2; 17.1]	1.04 [0.88; 1.58]	0.99 [0.75; 1.48]	-0.05 [-0.62; 0.45]
Cook Islands	1.58 [1.38; 1.80]	1.51 [1.17; 1.96]	-0.06 [-0.51; 0.43]	1.1 [0.9; 1.5]	0.8 [0.6; 1.0]	0.99 [0.85; 1.14]	0.99 [0.78; 1.26]	0.00 [-0.28; 0.32]
Costa Rica	1.50 [1.34; 1.68]	1.26 [1.07; 1.49]	-0.24 [-0.51; 0.05]	1.1 [0.9; 1.2]	0.8 [0.7; 1.0]	1.06 [0.95; 1.18]	1.17 [1.00; 1.37]*	0.11 [-0.11; 0.34]
Cote d'Ivoire	1.00 [0.87; 1.14]	0.94 [0.73; 1.21]	-0.06 [-0.32; 0.25]	10.0 [2.5; 23.7]	10.6 [2.7; 25.5]	1.00 [0.87; 1.23]	1.10 [0.83; 1.70]	0.10 [-0.29; 0.71]
Croatia	1.69 [1.53; 1.88]	1.32 [1.08; 1.62]	-0.37 [-0.68; -0.02]†	0.6 [0.4; 0.9]	0.5 [0.3; 0.7]	0.94 [0.85; 1.04]	1.01 [0.83; 1.23]	0.07 [-0.14; 0.32]
Cuba	1.55 [1.43; 1.68]	1.43 [1.24; 1.63]	-0.13 [-0.35; 0.11]	1.1 [0.9; 1.4]	0.8 [0.6; 1.0]	1.03 [0.95; 1.12]	1.05 [0.92; 1.20]	0.02 [-0.14; 0.20]
Cyprus	1.42 [1.24; 1.61]	1.46 [1.14; 1.88]	0.04 [-0.35; 0.51]	0.7 [0.4; 1.1]	0.5 [0.3; 0.8]	0.99 [0.85; 1.14]	0.94 [0.73; 1.21]	-0.04 [-0.32; 0.26]
Czech Republic	1.66 [1.53; 1.80]	1.27 [1.09; 1.49]	-0.39 [-0.63; -0.13]†	0.5 [0.4; 0.6]	0.4 [0.3; 0.5]	0.94 [0.87; 1.02]	1.02 [0.87; 1.19]	0.08 [-0.09; 0.27]
Denmark	1.49 [1.34; 1.66]	1.32 [1.10; 1.58]	-0.17 [-0.47; 0.14]	0.4 [0.3; 0.5]	0.3 [0.2; 0.4]	1.01 [0.91; 1.11]	0.98 [0.82; 1.18]	-0.03 [-0.23; 0.20]
Djibouti	0.99 [0.86; 1.14]	1.12 [0.87; 1.46]	0.13 [-0.18; 0.51]	5.4 [4.1; 7.1]	4.8 [3.6; 6.4]	1.00 [0.87; 1.16]	1.01 [0.73; 1.45]	0.01 [-0.33; 0.48]
Dominica	1.57 [1.37; 1.80]	1.63 [1.26; 2.12]	0.06 [-0.40; 0.60]	1.9 [0.5; 4.4]	1.2 [0.3; 2.7]	0.97 [0.75; 1.14]	0.92 [0.67; 1.22]	-0.05 [-0.37; 0.33]
Dominican Republic	1.50 [1.31; 1.70]	1.58 [1.23; 2.04]	0.08 [-0.35; 0.60]	2.0 [0.7; 4.3]	1.2 [0.4; 2.8]	0.99 [0.75; 1.18]	0.96 [0.70; 1.25]	-0.03 [-0.37; 0.36]
Ecuador	1.33 [1.25; 1.42]	1.26 [1.14; 1.40]	-0.07 [-0.22; 0.09]	1.5 [1.3; 1.7]	1.2 [1.0; 1.3]	1.02 [0.93; 1.13]	1.24 [1.13; 1.37]*	0.22 [0.06; 0.38]†
Egypt	1.14 [1.03; 1.26]	1.75 [1.48; 2.05]	0.61 [0.30; 0.95]†	2.5 [2.2; 2.9]	1.4 [1.2; 1.7]	1.00 [0.88; 1.13]	0.91 [0.77; 1.08]	-0.08 [-0.29; 0.13]
El Salvador	1.40 [1.29; 1.52]	1.49 [1.28; 1.72]	0.08 [-0.16; 0.35]	3.0 [2.5; 3.4]	2.0 [1.7; 2.3]	1.02 [0.91; 1.13]	1.05 [0.89; 1.23]	0.03 [-0.17; 0.25]
Equatorial Guinea	0.98 [0.85; 1.14]	1.03 [0.80; 1.35]	0.05 [-0.25; 0.41]	6.7 [5.0; 8.8]	6.4 [4.8; 8.6]	1.00 [0.87; 1.15]	1.01 [0.75; 1.39]	0.01 [-0.30; 0.42]
Eritrea	0.99 [0.86; 1.14]	1.34 [1.03; 1.73]	0.34 [-0.01; 0.77]	4.1 [0.9; 12.4]	3.1 [0.6; 9.3]	1.01 [0.87; 1.40]	0.96 [0.62; 1.43]	-0.06 [-0.55; 0.44]
Estonia	1.68 [1.51; 1.89]	1.15 [0.93; 1.43]	-0.53 [-0.85; -0.18]†	0.6 [0.4; 0.9]	0.5 [0.3; 0.8]	0.94 [0.83; 1.05]	1.15 [0.93; 1.41]	0.21 [-0.05; 0.51]
Ethiopia	1.04 [0.88; 1.24]	1.47 [1.15; 1.88]	0.43 [0.05; 0.88]†	3.5 [0.6; 8.4]	2.4 [0.4; 5.8]	0.97 [0.84; 1.21]	0.90 [0.59; 1.29]	-0.06 [-0.47; 0.35]
Federated States of Micronesia	1.29 [1.13; 1.47]	1.57 [1.22; 2.04]	0.28 [-0.14; 0.79]	2.9 [2.2; 3.8]	1.8 [1.4; 2.5]	1.00 [0.80; 1.25]	0.99 [0.74; 1.30]	-0.01 [-0.36; 0.37]
Fiji	1.10 [0.96; 1.26]	1.43 [1.20; 1.72]	0.34 [0.04; 0.67]†	3.3 [2.4; 4.2]	2.3 [1.7; 3.0]	1.03 [0.83; 1.50]	1.05 [0.81; 1.30]	0.01 [-0.51; 0.35]
Finland	1.53 [1.38; 1.69]	1.12 [0.94; 1.34]	-0.41 [-0.67; -0.13]†	0.4 [0.3; 0.5]	0.4 [0.3; 0.5]	0.98 [0.88; 1.08]	1.16 [0.97; 1.38]	0.18 [-0.05; 0.43]
France	1.42 [1.34; 1.50]	1.30 [1.17; 1.45]	-0.12 [-0.28; 0.06]	0.4 [0.4; 0.5]	0.3 [0.3; 0.4]	1.07 [1.01; 1.13]*	0.99 [0.89; 1.11]	-0.08 [-0.20; 0.06]
Gabon	1.00 [0.87; 1.15]	1.04 [0.81; 1.35]	0.04 [-0.25; 0.38]	6.6 [1.4; 18.6]	6.4 [1.4; 18.0]	1.05 [0.87; 1.62]	1.06 [0.76; 1.69]	0.01 [-0.67; 0.70]
The Gambia	0.98 [0.85; 1.13]	1.25 [0.98; 1.59]	0.27 [-0.05; 0.65]	5.0 [3.8; 6.6]	4.0 [3.1; 5.3]	1.01 [0.88; 1.16]	0.94 [0.69; 1.35]	-0.07 [-0.38; 0.36]
Georgia	1.70 [1.51; 1.93]	1.53 [1.27; 1.85]	-0.17 [-0.54; 0.22]	1.2 [1.0; 1.4]	0.8 [0.6; 1.0]	0.94 [0.83; 1.06]	0.99 [0.83; 1.19]	0.06 [-0.16; 0.29]
Germany	1.49 [1.40; 1.57]	1.17 [1.06; 1.28]	-0.32 [-0.46; -0.18]†	0.4 [0.4; 0.5]	0.4 [0.3; 0.4]	1.01 [0.95; 1.07]	1.11 [1.00; 1.22]*	0.10 [-0.02; 0.23]
Ghana	1.00 [0.88; 1.15]	1.16 [0.91; 1.47]	0.15 [-0.15; 0.50]	4.7 [1.9; 8.6]	4.1 [1.7; 7.4]	1.01 [0.87; 1.31]	1.06 [0.74; 1.58]	0.05 [-0.41; 0.61]
Greece	1.51 [1.39; 1.65]	1.38 [1.18; 1.62]	-0.13 [-0.39; 0.14]	0.5 [0.4; 0.6]	0.3 [0.3; 0.4]	1.00 [0.92; 1.08]	0.93 [0.80; 1.10]	-0.06 [-0.23; 0.12]
Grenada	1.64 [1.43; 1.87]	1.67 [1.30; 2.16]	0.04 [-0.44; 0.59]	2.6 [1.1; 4.5]	1.5 [0.6; 2.8]	0.95 [0.79; 1.10]	0.91 [0.66; 1.20]	-0.04 [-0.35; 0.31]
Guatemala	1.06 [1.01; 1.12]	1.25 [1.11; 1.42]	0.19 [0.04; 0.36]†	2.2 [2.0; 2.4]	1.7 [1.5; 1.9]	1.07 [0.98; 1.16]	1.28 [1.13; 1.44]*	0.21 [0.03; 0.39]†
Guinea	1.01 [0.87; 1.17]	1.04 [0.82; 1.32]	0.03 [-0.27; 0.35]	6.0 [1.5; 13.8]	5.8 [1.4; 13.3]	0.99 [0.85; 1.18]	1.08 [0.79; 1.69]	0.09 [-0.29; 0.73]
Guinea-Bissau	1.02 [0.89; 1.18]	1.07 [0.83; 1.37]	0.04 [-0.25; 0.38]	5.8 [1.2; 13.5]	5.5 [1.1; 12.7]	1.00 [0.82; 1.49]	1.06 [0.76; 1.67]	0.06 [-0.49; 0.71]
Guyana	1.48 [1.32; 1.67]	1.44 [1.16; 1.77]	-0.05 [-0.40; 0.35]	3.2 [2.1; 4.9]	2.2 [1.4; 3.4]	1.03 [0.89; 1.18]	1.04 [0.74; 1.34]	0.01 [-0.33; 0.35]
Haiti	0.99 [0.86; 1.14]	1.13 [0.85; 1.44]	0.14 [-0.21; 0.52]	5.4 [1.3; 12.1]	4.8 [1.2; 10.6]	1.01 [0.87; 1.27]	1.02 [0.68; 1.56]	0.00 [-0.44; 0.57]
Honduras	1.41 [1.23; 1.61]	1.28 [1.00; 1.63]	-0.12 [-0.50; 0.28]	3.3 [1.4; 6.9]	2.6 [1.1; 5.5]	0.99 [0.72; 1.23]	1.11 [0.72; 1.53]	0.12 [-0.35; 0.64]
Hungary	1.54 [1.42; 1.67]	1.41 [1.20; 1.66]	-0.13 [-0.38; 0.15]	0.6 [0.5; 0.7]	0.4 [0.3; 0.5]	1.03 [0.95; 1.11]	0.93 [0.80; 1.09]	-0.09 [-0.25; 0.09]
Iceland	1.49 [1.31; 1.70]	1.27 [0.99; 1.65]	-0.22 [-0.59; 0.22]	0.4 [0.2; 0.7]	0.3 [0.1; 0.5]	0.99 [0.86; 1.13]	1.02 [0.79; 1.31]	0.03 [-0.26; 0.36]

¶: Sex ratio is outlying in 1990. §: Sex ratio is outlying in 2021. *: The ratio of estimated to expected female mortality is significantly different from one. †: Change is significantly different from zero.

Continued on next page

Table 17 – continued from previous page

	Sex ratio 5q10			Sex-specific 5q10 in 2021 (per 1,000)		Estimated/Expected female 5q10		
	1990	2021	Change 1990–2021	Male	Female	1990	2021	Change 1990–2021
India	0.96<br>[0.87; 1.06]	1.34<br>[1.14; 1.58]	0.38<br>[0.16; 0.64]†	2.7<br>[2.1; 3.4]	2.0<br>[1.5; 2.5]	1.08<br>[0.96; 1.22]	1.18<br>[0.98; 1.39]	0.10<br>[-0.15; 0.35]
Indonesia	1.09<br>[0.96; 1.24]	1.63<br>[1.29; 2.07]	0.54<br>[0.16; 1.01]†	2.5<br>[0.9; 5.1]	1.5<br>[0.5; 3.2]	1.13<br>[0.90; 1.51]	0.92<br>[0.65; 1.19]	-0.21<br>[-0.67; 0.17]
Iran (Islamic Republic of)	1.03<br>[0.90; 1.17]	1.39<br>[1.16; 1.68]	0.37<br>[0.08; 0.69]†	1.9<br>[1.5; 2.4]	1.4<br>[1.1; 1.7]	0.99<br>[0.75; 1.26]	1.15<br>[0.95; 1.38]	0.15<br>[-0.18; 0.50]
Iraq	1.20<br>[1.04; 1.37]	1.51<br>[1.18; 1.93]	0.31<br>[-0.08; 0.78]	3.2<br>[0.9; 9.0]	2.1<br>[0.6; 6.0]	1.02<br>[0.77; 1.42]	0.93<br>[0.59; 1.28]	-0.09<br>[-0.66; 0.36]
Ireland	1.51<br>[1.36; 1.67]	1.59<br>[1.34; 1.88]	0.08<br>[-0.23; 0.41]	0.4<br>[0.3; 0.5]	0.3<br>[0.2; 0.3]	1.01<br>[0.92; 1.11]	0.81<br>[0.69; 0.96]*	-0.19<br>[-0.36; -0.01]†
Israel	1.47<br>[1.33; 1.62]	1.33<br>[1.16; 1.54]	-0.14<br>[-0.37; 0.12]	0.5<br>[0.4; 0.6]	0.4<br>[0.3; 0.4]	1.02<br>[0.93; 1.12]	0.97<br>[0.84; 1.12]	-0.05<br>[-0.22; 0.13]
Italy	1.59<br>[1.50; 1.69]	1.32<br>[1.18; 1.49]	-0.27<br>[-0.45; -0.08]†	0.4<br>[0.3; 0.5]	0.3<br>[0.2; 0.4]	0.95<br>[0.90; 1.01]	0.97<br>[0.86; 1.10]	0.02<br>[-0.10; 0.16]
Jamaica	1.58<br>[1.38; 1.80]	1.58<br>[1.23; 2.05]	0.00<br>[-0.43; 0.52]	1.7<br>[1.3; 2.2]	1.1<br>[0.8; 1.5]	0.99<br>[0.85; 1.14]	1.00<br>[0.78; 1.28]	0.01<br>[-0.27; 0.34]
Japan	1.49<br>[1.42; 1.57]	1.26<br>[1.15; 1.37]	-0.24<br>[-0.37; -0.10]†	0.4<br>[0.4; 0.5]	0.3<br>[0.3; 0.4]	0.95<br>[0.91; 1.01]	1.03<br>[0.93; 1.13]	0.07<br>[-0.03; 0.19]
Jordan	1.59<br>[1.40; 1.82]	1.56<br>[1.20; 2.03]	-0.03<br>[-0.47; 0.50]	1.2<br>[0.2; 3.3]	0.8<br>[0.1; 2.1]	0.97<br>[0.79; 1.12]	0.95<br>[0.70; 1.25]	-0.02<br>[-0.33; 0.34]
Kazakhstan	1.88<br>[1.77; 2.00]	1.58<br>[1.45; 1.72]	-0.30<br>[-0.47; -0.11]†	1.7<br>[1.5; 1.8]	1.0<br>[1.0; 1.1]	0.79<br>[0.73; 0.85]*	1.00<br>[0.92; 1.10]	0.22<br>[0.11; 0.33]†
Kenya	1.12<br>[0.98; 1.28]	1.27<br>[0.99; 1.62]	0.15<br>[-0.19; 0.54]	4.7<br>[0.9; 12.8]	3.7<br>[0.7; 10.3]	1.03<br>[0.82; 1.48]	0.96<br>[0.66; 1.45]	-0.07<br>[-0.64; 0.48]
Kiribati	1.06<br>[0.92; 1.22]	1.26<br>[0.97; 1.63]	0.20<br>[-0.14; 0.61]	4.3<br>[3.2; 5.6]	3.4<br>[2.5; 4.5]	1.01<br>[0.85; 1.23]	1.04<br>[0.72; 1.48]	0.03<br>[-0.33; 0.48]
Democratic People’s Republic of Korea	1.36<br>[1.19; 1.56]	1.58<br>[1.22; 2.06]	0.22<br>[-0.21; 0.74]	2.0<br>[1.5; 2.6]	1.3<br>[1.0; 1.7]	0.99<br>[0.78; 1.22]	1.01<br>[0.77; 1.30]	0.02<br>[-0.33; 0.39]
Republic of Korea	1.53<br>[1.37; 1.70]	1.33<br>[1.14; 1.56]	-0.20<br>[-0.46; 0.09]	0.5<br>[0.4; 0.5]	0.3<br>[0.3; 0.4]	1.05<br>[0.94; 1.16]	0.97<br>[0.83; 1.13]	-0.08<br>[-0.27; 0.12]
Kosovo	1.41<br>[1.23; 1.61]	1.53<br>[1.18; 1.99]	0.12<br>[-0.30; 0.63]	1.3<br>[1.0; 1.7]	0.9<br>[0.6; 1.1]	1.00<br>[0.79; 1.21]	1.01<br>[0.78; 1.29]	0.01<br>[-0.33; 0.39]
Kuwait	1.61<br>[1.42; 1.84]	1.30<br>[1.09; 1.55]	-0.31<br>[-0.64; 0.02]	1.0<br>[0.8; 1.4]	0.8<br>[0.6; 1.1]	0.96<br>[0.84; 1.10]	1.13<br>[0.95; 1.35]	0.17<br>[-0.06; 0.43]
Kyrgyzstan	1.71<br>[1.57; 1.86]	1.55<br>[1.39; 1.73]	-0.16<br>[-0.38; 0.07]	1.9<br>[1.7; 2.0]	1.2<br>[1.1; 1.3]	0.90<br>[0.81; 0.99]*	1.03<br>[0.92; 1.15]	0.13<br>[-0.01; 0.29]
Lao People’s Democratic Republic	0.98<br>[0.85; 1.13]	1.31<br>[1.03; 1.65]	0.33<br>[0.00; 0.72]	4.8<br>[1.7; 8.6]	3.7<br>[1.3; 6.6]	1.04<br>[0.87; 1.56]	0.94<br>[0.65; 1.39]	-0.10<br>[-0.69; 0.39]
Latvia	1.79<br>[1.62; 1.98]	1.06<br>[0.86; 1.30]	-0.73<br>[-1.02; -0.41]†	0.5<br>[0.3; 0.7]	0.4<br>[0.3; 0.7]	0.85<br>[0.74; 0.95]*	1.22<br>[1.00; 1.50]*	0.38<br>[0.12; 0.69]†
Lebanon	1.36<br>[1.19; 1.56]	1.50<br>[1.16; 1.95]	0.14<br>[-0.28; 0.65]	1.1<br>[0.9; 1.5]	0.8<br>[0.6; 1.0]	1.00<br>[0.79; 1.23]	1.00<br>[0.78; 1.27]	0.00<br>[-0.36; 0.38]
Lesotho	1.03<br>[0.89; 1.18]	1.27<br>[0.98; 1.65]	0.24<br>[-0.09; 0.66]	4.3<br>[1.5; 8.1]	3.4<br>[1.1; 6.4]	1.01<br>[0.85; 1.45]	1.01<br>[0.68; 1.49]	0.00<br>[-0.53; 0.51]
Liberia	0.99<br>[0.86; 1.13]	1.09<br>[0.86; 1.39]	0.11<br>[-0.18; 0.45]	8.1<br>[2.0; 19.2]	7.4<br>[1.8; 17.6]	1.02<br>[0.87; 1.36]	0.97<br>[0.72; 1.52]	-0.05<br>[-0.46; 0.51]
Libya	1.55<br>[1.36; 1.77]	1.62<br>[1.25; 2.10]	0.07<br>[-0.39; 0.61]	1.8<br>[0.3; 6.9]	1.1<br>[0.2; 4.4]	0.91<br>[0.63; 1.12]	0.90<br>[0.60; 1.21]	-0.01<br>[-0.37; 0.40]
Lithuania	1.66<br>[1.51; 1.82]	1.30<br>[1.08; 1.56]	-0.35<br>[-0.63; -0.04]†	0.8<br>[0.6; 1.0]	0.6<br>[0.4; 0.8]	0.96<br>[0.87; 1.06]	1.07<br>[0.90; 1.27]	0.11<br>[-0.10; 0.33]
Luxembourg	1.45<br>[1.26; 1.65]	1.34<br>[1.04; 1.73]	-0.11<br>[-0.49; 0.34]	0.2<br>[0.0; 0.4]	0.1<br>[0.0; 0.3]	0.99<br>[0.86; 1.14]	0.96<br>[0.75; 1.24]	-0.03<br>[-0.30; 0.30]
Macedonia	1.54<br>[1.37; 1.74]	1.38<br>[1.11; 1.72]	-0.17<br>[-0.53; 0.23]	1.0<br>[0.7; 1.3]	0.7<br>[0.5; 1.0]	1.03<br>[0.92; 1.16]	1.05<br>[0.85; 1.30]	0.02<br>[-0.23; 0.31]
Madagascar	0.97<br>[0.85; 1.12]	1.09<br>[0.88; 1.36]	0.12<br>[-0.15; 0.43]	8.5<br>[2.0; 20.4]	7.7<br>[1.7; 18.6]	1.02<br>[0.89; 1.19]	0.97<br>[0.74; 1.50]	-0.05<br>[-0.36; 0.51]
Malawi	0.97<br>[0.84; 1.11]	1.20<br>[0.96; 1.49]	0.22<br>[-0.06; 0.57]	5.4<br>[2.5; 9.2]	4.5<br>[2.1; 7.7]	1.03<br>[0.90; 1.21]	0.97<br>[0.71; 1.47]	-0.05<br>[-0.41; 0.46]
Malaysia	1.56<br>[1.44; 1.70]	1.44<br>[1.32; 1.56]	-0.13<br>[-0.30; 0.05]	1.2<br>[1.1; 1.3]	0.8<br>[0.7; 0.9]	0.99<br>[0.90; 1.08]	1.05<br>[0.97; 1.14]	0.07<br>[-0.06; 0.19]
Maldives	1.29<br>[1.13; 1.47]	1.44<br>[1.12; 1.83]	0.14<br>[-0.24; 0.59]	1.0<br>[0.5; 1.5]	0.7<br>[0.4; 1.0]	1.06<br>[0.83; 1.30]	1.01<br>[0.78; 1.29]	-0.05<br>[-0.39; 0.33]
Mali	0.95<br>[0.84; 1.08]	0.93<br>[0.74; 1.17]	-0.02<br>[-0.26; 0.25]	8.1<br>[2.5; 15.6]	8.7<br>[2.7; 16.8]	1.05<br>[0.91; 1.25]	1.12<br>[0.86; 1.73]	0.08<br>[-0.28; 0.69]
Malta	1.39<br>[1.22; 1.60]	1.35<br>[1.05; 1.75]	-0.04<br>[-0.43; 0.41]	0.7<br>[0.3; 1.1]	0.5<br>[0.2; 0.8]	1.00<br>[0.85; 1.19]	1.00<br>[0.77; 1.29]	0.00<br>[-0.32; 0.34]
Marshall Islands	1.31<br>[1.15; 1.50]	1.52<br>[1.18; 1.97]	0.21<br>[-0.20; 0.71]	3.2<br>[2.5; 4.2]	2.1<br>[1.6; 2.8]	0.99<br>[0.79; 1.24]	0.99<br>[0.72; 1.32]	0.00<br>[-0.35; 0.38]
Mauritania	1.03<br>[0.90; 1.18]	1.35<br>[1.07; 1.69]	0.32<br>[-0.01; 0.71]	4.0<br>[0.8; 9.0]	3.0<br>[0.6; 6.7]	1.02<br>[0.86; 1.54]	0.96<br>[0.64; 1.38]	-0.07<br>[-0.65; 0.38]
Mauritius	1.48<br>[1.31; 1.67]	1.33<br>[1.07; 1.65]	-0.16<br>[-0.49; 0.23]	1.1<br>[0.8; 1.4]	0.8<br>[0.6; 1.1]	1.08<br>[0.95; 1.22]	1.12<br>[0.91; 1.37]	0.04<br>[-0.23; 0.34]
Mexico	1.53<br>[1.48; 1.58]	1.36<br>[1.27; 1.47]	-0.16<br>[-0.27; -0.05]†	1.6<br>[1.5; 1.8]	1.2<br>[1.1; 1.3]	1.02<br>[0.97; 1.06]	1.16<br>[1.08; 1.25]*	0.15<br>[0.05; 0.25]†
Republic of Moldova	1.57<br>[1.43; 1.72]	1.43<br>[1.20; 1.70]	-0.14<br>[-0.42; 0.18]	1.6<br>[1.2; 1.9]	1.1<br>[0.9; 1.4]	1.00<br>[0.91; 1.11]	1.10<br>[0.93; 1.30]	0.10<br>[-0.11; 0.33]
Monaco	1.50<br>[1.31; 1.71]	1.29<br>[0.99; 1.68]	-0.21<br>[-0.60; 0.25]	0.5<br>[0.4; 0.6]	0.4<br>[0.3; 0.5]	1.00<br>[0.87; 1.14]	1.00<br>[0.78; 1.30]	0.00<br>[-0.27; 0.34]
Mongolia	1.20<br>[1.06; 1.34]	1.77<br>[1.48; 2.10]	0.57<br>[0.23; 0.95]†	2.3<br>[1.8; 2.7]	1.3<br>[1.0; 1.6]	0.98<br>[0.83; 1.18]	0.90<br>[0.76; 1.08]	-0.07<br>[-0.33; 0.17]
Montenegro	1.55<br>[1.36; 1.77]	1.36<br>[1.05; 1.76]	-0.19<br>[-0.59; 0.27]	0.5<br>[0.2; 0.9]	0.4<br>[0.2; 0.7]	1.00<br>[0.86; 1.14]	0.97<br>[0.75; 1.25]	-0.03<br>[-0.30; 0.29]
Montserrat	1.62<br>[1.42; 1.85]	1.58<br>[1.22; 2.03]	-0.04<br>[-0.49; 0.48]	0.8<br>[0.6; 1.1]	0.5<br>[0.4; 0.7]	0.99<br>[0.87; 1.12]	0.90<br>[0.71; 1.14]	-0.09<br>[-0.34; 0.19]
Morocco	1.34<br>[1.17; 1.52]	1.42<br>[1.11; 1.84]	0.08<br>[-0.30; 0.55]	1.1<br>[0.2; 2.8]	0.8<br>[0.2; 2.0]	1.01<br>[0.76; 1.27]	1.03<br>[0.77; 1.35]	0.02<br>[-0.36; 0.45]
Mozambique	0.99<br>[0.84; 1.17]	1.04<br>[0.80; 1.35]	0.05<br>[-0.27; 0.42]	6.0<br>[1.3; 15.4]	5.8<br>[1.2; 14.8]	1.02<br>[0.88; 1.40]	1.08<br>[0.77; 1.73]	0.06<br>[-0.42; 0.73]
Myanmar	1.05<br>[0.91; 1.20]	1.57<br>[1.21; 2.02]	0.52<br>[0.11; 1.00]†	2.4<br>[0.4; 6.3]	1.5<br>[0.3; 4.1]	1.01<br>[0.82; 1.56]	0.92<br>[0.61; 1.25]	-0.09<br>[-0.73; 0.30]
Namibia	1.14<br>[0.99; 1.30]	1.01<br>[0.79; 1.30]	-0.13<br>[-0.41; 0.22]	6.4<br>[1.9; 13.0]	6.4<br>[1.9; 12.9]	1.00<br>[0.80; 1.46]	1.09<br>[0.79; 1.72]	0.09<br>[-0.51; 0.76]

¶: Sex ratio is outlying in 1990. §: Sex ratio is outlying in 2021. *: The ratio of estimated to expected female mortality is significantly different from one. †: Change is significantly different from zero.

Continued on next page

Table 17 – continued from previous page

	Sex ratio 5q10			Sex-specific 5q10 in 2021 (per 1,000)		Estimated/Expected female 5q10		
	**1990**	**2021**	**Change 1990–2021**	**Male**	**Female**	**1990**	**2021**	**Change 1990–2021**
Nauru	1.16 [1.00; 1.33]	1.55 [1.20; 2.00]	0.40 [-0.02; 0.90]	3.1 [2.3; 4.0]	2.0 [1.5; 2.6]	1.00 [0.82; 1.27]	0.99 [0.73; 1.31]	-0.01 [-0.37; 0.35]
Nepal	1.03 [0.91; 1.18]	1.43 [1.12; 1.81]	0.40 [0.04; 0.81]†	2.4 [0.7; 5.9]	1.7 [0.5; 4.2]	1.00 [0.86; 1.47]	1.04 [0.70; 1.37]	0.04 [-0.49; 0.40]
Netherlands	1.30 [1.19; 1.41]	1.34 [1.17; 1.54]	0.04 [-0.16; 0.27]	0.5 [0.4; 0.6]	0.4 [0.3; 0.4]	1.14 [1.06; 1.23]*	0.96 [0.84; 1.10]	-0.18 [-0.34; -0.01]†
New Zealand	1.58 [1.43; 1.75]	1.29 [1.12; 1.49]	-0.29 [-0.53; -0.03]†	0.7 [0.5; 0.8]	0.5 [0.4; 0.6]	1.01 [0.91; 1.11]	1.05 [0.91; 1.20]	0.04 [-0.14; 0.23]
Nicaragua	1.52 [1.33; 1.74]	1.58 [1.22; 2.05]	0.06 [-0.38; 0.59]	2.5 [0.7; 6.0]	1.6 [0.4; 3.8]	0.95 [0.68; 1.15]	0.93 [0.62; 1.25]	-0.02 [-0.41; 0.41]
Niger	0.98 [0.85; 1.14]	0.98 [0.78; 1.23]	0.00 [-0.27; 0.29]	11.9 [3.0; 23.5]	12.2 [3.1; 23.9]	1.02 [0.88; 1.23]	1.05 [0.81; 1.53]	0.02 [-0.31; 0.53]
Nigeria	0.98 [0.85; 1.12]	1.11 [0.91; 1.36]	0.13 [-0.13; 0.43]	7.2 [2.0; 14.3]	6.5 [1.8; 13.0]	1.02 [0.89; 1.21]	0.97 [0.75; 1.49]	-0.05 [-0.36; 0.49]
Niue	1.76 [1.55; 2.01]	1.65 [1.27; 2.14]	-0.11 [-0.58; 0.45]	2.9 [2.2; 3.7]	1.7 [1.3; 2.3]	0.88 [0.73; 1.01]	0.94 [0.70; 1.24]	0.07 [-0.21; 0.40]
Norway	1.60 [1.43; 1.78]	1.04 [0.87; 1.25]	-0.55 [-0.82; -0.28]†	0.4 [0.3; 0.5]	0.4 [0.3; 0.5]	0.94 [0.84; 1.04]	1.24 [1.04; 1.48]*	0.30 [0.07; 0.57]†
Oman	1.23 [1.07; 1.42]	1.57 [1.20; 2.04]	0.34 [-0.10; 0.86]	1.4 [1.1; 1.8]	0.9 [0.7; 1.2]	1.01 [0.81; 1.28]	0.99 [0.77; 1.28]	-0.01 [-0.39; 0.36]
Pakistan	1.07 [0.97; 1.19]	1.13 [0.89; 1.43]	0.05 [-0.22; 0.38]	3.7 [1.1; 8.9]	3.3 [1.0; 8.0]	1.02 [0.89; 1.19]	1.20 [0.79; 1.67]	0.18 [-0.28; 0.68]
Palau	1.50 [1.31; 1.71]	1.65 [1.27; 2.14]	0.15 [-0.30; 0.70]	2.2 [1.7; 2.8]	1.3 [1.0; 1.8]	0.96 [0.76; 1.14]	0.97 [0.74; 1.25]	0.01 [-0.29; 0.37]
Panama	1.46 [1.30; 1.64]	1.39 [1.19; 1.63]	-0.07 [-0.35; 0.23]	1.9 [1.6; 2.2]	1.4 [1.2; 1.6]	1.09 [0.96; 1.23]	1.15 [0.98; 1.34]	0.06 [-0.16; 0.30]
Papua New Guinea	1.08 [0.94; 1.24]	1.26 [0.97; 1.63]	0.18 [-0.17; 0.59]	3.9 [3.0; 5.1]	3.1 [2.3; 4.1]	1.01 [0.85; 1.23]	1.09 [0.76; 1.53]	0.09 [-0.29; 0.55]
Paraguay	1.52 [1.32; 1.74]	1.59 [1.23; 2.07]	0.07 [-0.37; 0.61]	2.2 [0.7; 4.8]	1.4 [0.4; 3.0]	0.97 [0.70; 1.16]	0.95 [0.67; 1.26]	-0.02 [-0.37; 0.40]
Peru	1.31 [1.15; 1.49]	1.27 [1.01; 1.61]	-0.03 [-0.37; 0.35]	1.4 [0.6; 2.5]	1.1 [0.5; 2.0]	1.00 [0.78; 1.28]	1.20 [0.94; 1.52]	0.20 [-0.19; 0.60]
Philippines	1.27 [1.13; 1.43]	1.49 [1.26; 1.75]	0.22 [-0.07; 0.53]	2.7 [2.3; 3.1]	1.8 [1.5; 2.1]	1.04 [0.87; 1.25]	1.06 [0.89; 1.26]	0.02 [-0.26; 0.30]
Poland	1.71 [1.61; 1.81]	1.11 [1.00; 1.24]	-0.60 [-0.75; -0.44]†	0.6 [0.5; 0.6]	0.5 [0.5; 0.6]	0.93 [0.88; 0.99]*	1.17 [1.06; 1.30]*	0.24 [0.11; 0.38]†
Portugal	1.68 [1.56; 1.80]	1.19 [1.02; 1.38]	-0.49 [-0.71; -0.26]†	0.5 [0.4; 0.6]	0.4 [0.3; 0.5]	0.95 [0.88; 1.03]	1.09 [0.94; 1.27]	0.13 [-0.04; 0.33]
Qatar	1.59 [1.39; 1.82]	1.39 [1.10; 1.78]	-0.20 [-0.60; 0.26]	0.7 [0.4; 1.0]	0.5 [0.3; 0.8]	0.99 [0.85; 1.14]	0.98 [0.77; 1.24]	-0.01 [-0.28; 0.30]
Romania	1.72 [1.62; 1.82]	1.43 [1.28; 1.59]	-0.29 [-0.48; -0.10]†	1.1 [0.9; 1.3]	0.8 [0.6; 0.9]	0.91 [0.85; 0.97]*	1.04 [0.94; 1.16]	0.13 [0.00; 0.26]†
Russian Federation	1.99 [1.92; 2.06]	1.49 [1.38; 1.61]	-0.50 [-0.64; -0.36]†	1.2 [1.1; 1.4]	0.8 [0.7; 0.9]	0.77 [0.74; 0.81]*	1.02 [0.95; 1.11]	0.25 [0.16; 0.34]†
Rwanda	1.02 [0.86; 1.19]	1.05 [0.83; 1.33]	0.03 [-0.27; 0.36]	7.0 [1.1; 23.1]	6.6 [1.0; 21.7]	0.98 [0.86; 1.14]	1.04 [0.76; 1.65]	0.05 [-0.28; 0.69]
Saint Kitts and Nevis	1.65 [1.44; 1.88]	1.60 [1.22; 2.06]	-0.05 [-0.52; 0.48]	1.9 [1.5; 2.5]	1.2 [0.9; 1.6]	0.94 [0.79; 1.09]	1.00 [0.77; 1.29]	0.06 [-0.23; 0.40]
Saint Lucia	1.60 [1.40; 1.83]	1.59 [1.23; 2.05]	-0.02 [-0.46; 0.51]	1.5 [0.4; 3.0]	0.9 [0.2; 1.9]	0.96 [0.76; 1.12]	0.95 [0.71; 1.24]	0.00 [-0.30; 0.35]
Samoa	1.50 [1.31; 1.72]	1.51 [1.17; 1.95]	0.01 [-0.41; 0.51]	1.5 [0.4; 3.0]	1.0 [0.2; 2.0]	0.96 [0.68; 1.17]	1.00 [0.75; 1.31]	0.04 [-0.30; 0.44]
San Marino	1.59 [1.39; 1.81]	1.29 [1.00; 1.67]	-0.30 [-0.69; 0.16]	0.3 [0.2; 0.4]	0.2 [0.2; 0.3]	1.00 [0.88; 1.14]	1.00 [0.77; 1.29]	0.00 [-0.29; 0.33]
Sao Tome and Principe	1.00 [0.87; 1.15]	1.51 [1.17; 1.95]	0.51 [0.12; 0.97]†	2.2 [1.7; 2.8]	1.4 [1.1; 1.9]	1.00 [0.87; 1.16]	1.06 [0.82; 1.36]	0.06 [-0.25; 0.40]
Saudi Arabia	1.17 [1.02; 1.35]	1.43 [1.10; 1.86]	0.26 [-0.14; 0.73]	0.9 [0.7; 1.2]	0.6 [0.5; 0.8]	1.00 [0.82; 1.27]	1.00 [0.78; 1.28]	0.00 [-0.38; 0.37]
Senegal	1.00 [0.87; 1.13]	1.41 [1.13; 1.76]	0.42 [0.08; 0.81]†	4.2 [1.2; 8.3]	3.0 [0.8; 5.9]	1.01 [0.87; 1.30]	0.91 [0.63; 1.30]	-0.10 [-0.50; 0.33]
Serbia	1.54 [1.39; 1.72]	1.49 [1.23; 1.79]	-0.05 [-0.38; 0.31]	0.8 [0.6; 1.0]	0.5 [0.4; 0.7]	1.03 [0.93; 1.15]	0.94 [0.79; 1.12]	-0.10 [-0.30; 0.13]
Seychelles	1.60 [1.39; 1.82]	1.69 [1.32; 2.16]	0.09 [-0.37; 0.62]	1.6 [0.5; 3.4]	1.0 [0.3; 2.0]	0.97 [0.81; 1.12]	0.90 [0.68; 1.17]	-0.07 [-0.36; 0.26]
Sierra Leone	0.99 [0.86; 1.15]	0.91 [0.74; 1.14]	-0.08 [-0.32; 0.19]	9.5 [2.7; 20.5]	10.4 [2.9; 22.5]	1.03 [0.86; 1.53]	1.13 [0.88; 1.72]	0.10 [-0.44; 0.73]
Singapore	1.54 [1.37; 1.74]	1.28 [1.02; 1.60]	-0.27 [-0.62; 0.12]	0.4 [0.3; 0.5]	0.3 [0.2; 0.4]	1.03 [0.91; 1.15]	1.01 [0.81; 1.27]	-0.02 [-0.27; 0.28]
Slovakia	1.61 [1.47; 1.76]	1.27 [1.07; 1.50]	-0.34 [-0.59; -0.06]†	0.7 [0.6; 0.9]	0.6 [0.4; 0.7]	0.98 [0.90; 1.07]	1.08 [0.92; 1.26]	0.10 [-0.10; 0.31]
Slovenia	1.60 [1.41; 1.80]	1.30 [1.02; 1.66]	-0.30 [-0.66; 0.11]	0.4 [0.3; 0.6]	0.3 [0.2; 0.4]	0.97 [0.86; 1.09]	0.99 [0.78; 1.26]	0.02 [-0.23; 0.32]
Solomon Islands	1.42 [1.24; 1.62]	1.56 [1.21; 2.02]	0.14 [-0.29; 0.67]	2.3 [1.8; 3.0]	1.5 [1.1; 2.0]	1.00 [0.79; 1.21]	1.02 [0.78; 1.32]	0.03 [-0.31; 0.40]
Somalia	0.99 [0.85; 1.14]	0.99 [0.76; 1.28]	0.00 [-0.28; 0.33]	9.2 [6.9; 12.2]	9.3 [7.0; 12.3]	1.00 [0.87; 1.14]	1.01 [0.77; 1.32]	0.01 [-0.28; 0.36]
South Africa	1.58 [1.39; 1.80]	1.39 [1.19; 1.61]	-0.19 [-0.50; 0.12]	3.5 [2.7; 4.5]	2.5 [2.0; 3.2]	0.90 [0.63; 1.10]	1.05 [0.83; 1.28]	0.15 [-0.16; 0.53]
South Sudan	1.00 [0.85; 1.17]	1.00 [0.77; 1.31]	0.01 [-0.30; 0.36]	8.4 [6.3; 11.0]	8.3 [6.2; 11.0]	1.00 [0.87; 1.15]	1.00 [0.76; 1.34]	0.00 [-0.30; 0.38]
Spain	1.50 [1.42; 1.59]	1.28 [1.13; 1.44]	-0.22 [-0.40; -0.04]†	0.4 [0.4; 0.5]	0.3 [0.3; 0.4]	1.03 [0.98; 1.10]	1.01 [0.89; 1.15]	-0.03 [-0.16; 0.12]
Sri Lanka	1.39 [1.25; 1.54]	1.24 [1.02; 1.52]	-0.15 [-0.43; 0.18]	0.9 [0.7; 1.3]	0.7 [0.5; 1.0]	1.07 [0.94; 1.21]	1.16 [0.96; 1.40]	0.09 [-0.17; 0.38]
Saint Vincent and the Grenadines	1.58 [1.39; 1.81]	1.43 [1.10; 1.85]	-0.16 [-0.57; 0.33]	3.7 [1.9; 6.0]	2.6 [1.3; 4.2]	0.98 [0.83; 1.13]	0.99 [0.65; 1.38]	0.00 [-0.37; 0.43]
State of Palestine	1.60 [1.40; 1.82]	1.60 [1.25; 2.07]	0.00 [-0.43; 0.54]	2.2 [0.6; 4.8]	1.4 [0.4; 3.1]	0.95 [0.73; 1.11]	0.94 [0.67; 1.24]	-0.01 [-0.34; 0.36]
Sudan	1.01 [0.89; 1.16]	1.47 [1.15; 1.88]	0.46 [0.08; 0.90]†	3.3 [0.6; 9.3]	2.3 [0.4; 6.4]	1.04 [0.87; 1.59]	0.91 [0.59; 1.28]	-0.13 [-0.77; 0.29]
Suriname§	1.54 [1.35; 1.75]	0.71 [0.60; 0.85]	-0.83 [-1.08; -0.58]†	1.7 [1.1; 2.4]	2.4 [1.6; 3.3]	0.98 [0.81; 1.14]	2.21 [1.85; 2.63]*	1.23 [0.82; 1.69]†

¶: Sex ratio is outlying in 1990. §: Sex ratio is outlying in 2021. *: The ratio of estimated to expected female mortality is significantly different from one. †: Change is significantly different from zero.

Continued on next page

Table 17 – continued from previous page

	Sex ratio 5q10			Sex-specific 5q10 in 2021 (per 1,000)		Estimated/Expected female 5q10		
	1990	2021	Change 1990–2021	Male	Female	1990	2021	Change 1990–2021
Eswatini	1.07 [0.93; 1.22]	1.60 [1.24; 2.05]	0.53 [0.11; 1.03]†	2.0 [0.6; 18.6]	1.3 [0.4; 11.7]	1.02 [0.82; 1.55]	0.73 [0.51; 1.14]	-0.29 [-0.92; 0.19]
Sweden	1.35 [1.23; 1.49]	1.24 [1.07; 1.45]	-0.11 [-0.35; 0.15]	0.4 [0.4; 0.5]	0.4 [0.3; 0.4]	1.05 [0.96; 1.15]	1.04 [0.89; 1.21]	-0.01 [-0.20; 0.19]
Switzerland	1.57 [1.42; 1.74]	1.11 [0.94; 1.31]	-0.46 [-0.71; -0.20]†	0.4 [0.3; 0.5]	0.4 [0.3; 0.5]	0.97 [0.88; 1.06]	1.16 [0.98; 1.38]	0.19 [-0.02; 0.44]
Syria	1.26 [1.12; 1.43]	1.55 [1.26; 1.96]	0.29 [-0.07; 0.73]	2.7 [0.8; 5.5]	1.7 [0.5; 3.6]	1.06 [0.82; 1.33]	0.96 [0.68; 1.21]	-0.10 [-0.49; 0.27]
Tajikistan	1.68 [1.54; 1.82]	1.36 [1.21; 1.53]	-0.31 [-0.53; -0.09]†	1.2 [1.1; 1.3]	0.9 [0.8; 1.0]	0.78 [0.69; 0.88]*	1.12 [1.00; 1.25]*	0.34 [0.18; 0.50]†
Tanzania	0.98 [0.86; 1.12]	1.39 [1.08; 1.79]	0.41 [0.05; 0.83]†	4.2 [0.7; 17.2]	3.0 [0.5; 12.4]	1.05 [0.89; 1.50]	0.86 [0.59; 1.33]	-0.18 [-0.67; 0.31]
Thailand	1.51 [1.35; 1.69]	1.76 [1.49; 2.09]	0.26 [-0.09; 0.63]	3.2 [2.8; 3.6]	1.8 [1.6; 2.1]	1.00 [0.87; 1.13]	0.87 [0.71; 1.05]	-0.13 [-0.34; 0.10]
Timor Leste	0.98 [0.85; 1.13]	1.22 [0.94; 1.59]	0.24 [-0.10; 0.64]	4.3 [3.3; 5.6]	3.5 [2.6; 4.7]	1.01 [0.88; 1.17]	1.06 [0.74; 1.53]	0.05 [-0.32; 0.54]
Togo	1.00 [0.88; 1.15]	1.11 [0.87; 1.41]	0.10 [-0.20; 0.45]	5.1 [1.3; 10.1]	4.6 [1.2; 9.1]	1.03 [0.87; 1.55]	1.07 [0.75; 1.64]	0.05 [-0.58; 0.65]
Tonga	1.63 [1.43; 1.86]	1.43 [1.11; 1.86]	-0.20 [-0.61; 0.30]	0.9 [0.2; 2.1]	0.6 [0.1; 1.5]	0.95 [0.77; 1.10]	1.01 [0.76; 1.33]	0.06 [-0.25; 0.44]
Trinidad and Tobago	1.45 [1.29; 1.62]	1.32 [1.10; 1.60]	-0.12 [-0.42; 0.20]	1.5 [1.0; 2.0]	1.1 [0.8; 1.5]	1.10 [0.98; 1.24]	1.17 [0.98; 1.40]	0.07 [-0.18; 0.34]
Tunisia	1.46 [1.28; 1.66]	1.52 [1.24; 1.86]	0.06 [-0.30; 0.46]	1.9 [1.5; 2.5]	1.3 [0.9; 1.7]	1.00 [0.83; 1.18]	1.05 [0.86; 1.29]	0.05 [-0.23; 0.36]
Turkey	1.54 [1.35; 1.75]	1.48 [1.34; 1.64]	-0.06 [-0.32; 0.19]	1.2 [1.0; 1.3]	0.8 [0.7; 0.9]	0.98 [0.75; 1.15]	1.02 [0.93; 1.13]	0.04 [-0.15; 0.30]
Turkmenistan	1.36 [1.22; 1.51]	1.44 [1.19; 1.75]	0.08 [-0.22; 0.44]	2.2 [1.6; 3.0]	1.5 [1.1; 2.1]	1.05 [0.91; 1.21]	1.11 [0.90; 1.34]	0.05 [-0.22; 0.34]
Turks and Caicos Islands	1.61 [1.41; 1.83]	1.41 [1.10; 1.83]	-0.19 [-0.61; 0.29]	0.9 [0.7; 1.2]	0.6 [0.5; 0.8]	0.99 [0.87; 1.13]	1.01 [0.79; 1.28]	0.02 [-0.25; 0.33]
Tuvalu	1.25 [1.09; 1.43]	1.59 [1.22; 2.06]	0.34 [-0.09; 0.85]	2.6 [2.0; 3.3]	1.6 [1.2; 2.2]	1.00 [0.80; 1.27]	0.99 [0.75; 1.30]	-0.01 [-0.37; 0.37]
Uganda	0.98 [0.85; 1.12]	1.13 [0.89; 1.44]	0.15 [-0.15; 0.50]	5.4 [1.3; 11.5]	4.8 [1.2; 10.2]	1.03 [0.89; 1.40]	1.02 [0.73; 1.59]	-0.01 [-0.47; 0.60]
Ukraine	1.79 [1.71; 1.88]	1.54 [1.41; 1.68]	-0.26 [-0.41; -0.09]†	1.1 [1.0; 1.2]	0.7 [0.6; 0.8]	0.89 [0.84; 0.94]*	0.97 [0.89; 1.06]	0.08 [-0.01; 0.18]
United Arab Emirates	1.60 [1.39; 1.82]	1.42 [1.10; 1.85]	-0.18 [-0.60; 0.33]	0.9 [0.7; 1.1]	0.6 [0.4; 0.8]	1.00 [0.87; 1.14]	1.00 [0.78; 1.27]	0.00 [-0.28; 0.32]
United Kingdom	1.50 [1.42; 1.59]	1.37 [1.24; 1.52]	-0.13 [-0.30; 0.05]	0.5 [0.4; 0.5]	0.3 [0.3; 0.4]	1.01 [0.95; 1.06]	0.94 [0.84; 1.05]	-0.07 [-0.18; 0.06]
United States of America	1.64 [1.58; 1.70]	1.40 [1.28; 1.54]	-0.24 [-0.37; -0.09]†	0.9 [0.8; 1.0]	0.6 [0.6; 0.7]	0.97 [0.93; 1.01]	1.02 [0.94; 1.12]	0.06 [-0.04; 0.15]
Uruguay	1.68 [1.51; 1.87]	1.36 [1.14; 1.62]	-0.33 [-0.63; 0.01]	1.0 [0.7; 1.2]	0.7 [0.5; 0.9]	0.95 [0.85; 1.06]	1.07 [0.90; 1.27]	0.12 [-0.09; 0.35]
Uzbekistan	1.45 [1.31; 1.61]	1.35 [1.18; 1.55]	-0.10 [-0.35; 0.16]	1.9 [1.8; 2.1]	1.4 [1.3; 1.6]	1.00 [0.87; 1.13]	1.18 [1.03; 1.36]*	0.19 [-0.03; 0.41]
Vanuatu	1.45 [1.27; 1.67]	1.60 [1.23; 2.08]	0.15 [-0.31; 0.68]	2.8 [2.1; 3.6]	1.7 [1.3; 2.3]	0.99 [0.79; 1.19]	0.98 [0.74; 1.29]	-0.01 [-0.33; 0.37]
Venezuela (Bolivarian Republic of)	1.58 [1.41; 1.76]	1.54 [1.27; 1.85]	-0.04 [-0.38; 0.33]	2.5 [1.8; 3.3]	1.6 [1.2; 2.2]	1.00 [0.89; 1.12]	1.03 [0.84; 1.25]	0.03 [-0.20; 0.29]
Vietnam	1.49 [1.31; 1.70]	1.81 [1.42; 2.30]	0.32 [-0.14; 0.86]	2.1 [1.2; 3.2]	1.1 [0.7; 1.8]	0.97 [0.68; 1.18]	0.87 [0.68; 1.11]	-0.10 [-0.40; 0.28]
Yemen	1.03 [0.90; 1.17]	1.54 [1.14; 2.21]	0.51 [-0.04; 1.20]	3.0 [0.5; 8.8]	2.0 [0.2; 5.9]	1.02 [0.87; 1.41]	0.90 [0.55; 1.28]	-0.12 [-0.64; 0.29]
Zambia	1.00 [0.88; 1.14]	1.37 [1.07; 1.76]	0.36 [0.01; 0.78]†	4.3 [0.8; 10.6]	3.2 [0.6; 7.8]	1.01 [0.87; 1.33]	0.90 [0.61; 1.34]	-0.11 [-0.55; 0.37]
Zimbabwe	1.14 [1.00; 1.30]	1.01 [0.80; 1.28]	-0.13 [-0.41; 0.19]	6.7 [2.4; 12.2]	6.6 [2.4; 12.1]	1.03 [0.81; 1.46]	1.08 [0.80; 1.67]	0.05 [-0.46; 0.66]

Table 18: **Estimates and 90% uncertainty intervals for sex ratios for 10q5 in 1990 and 2021, the change in sex ratios from 1990 to 2021, sex-specific 10q5 in 2021, and ratios of estimated to expected female 10q5 and their change from 1990 to 2021 for the world, UNICEF regions, and all countries.** ¶: Sex ratio is outlying in 1990. §: Sex ratio is outlying in 2021. *: The ratio of estimated to expected female mortality is significantly different from one. †: Change is significantly different from zero.

	Sex ratio 10q5			Sex-specific 10q5 in 2021 (per 1,000)		Estimated/Expected female 10q5		
	1990	2021	Change 1990–2021	Male	Female	1990	2021	Change 1990–2021
World	1.06 [1.04; 1.09]	1.16 [1.12; 1.20]	0.10 [0.06; 0.13]†	6.6 [6.4; 7.3]	5.7 [5.5; 6.3]	1.03 [1.00; 1.06]*	1.00 [0.96; 1.04]	-0.03 [-0.06; 0.01]
South Asia¶	0.94 [0.89; 0.98]	1.27 [1.16; 1.38]	0.33 [0.21; 0.45]†	5.2 [4.6; 6.1]	4.1 [3.6; 4.9]	1.10 [1.05; 1.17]*	1.13 [1.01; 1.24]*	0.02 [-0.11; 0.15]
Europe and Central Asia	1.51 [1.47; 1.54]	1.36 [1.33; 1.39]	-0.14 [-0.19; -0.10]†	1.7 [1.6; 1.7]	1.2 [1.2; 1.2]	0.92 [0.89; 0.95]*	1.04 [1.02; 1.06]*	0.12 [0.08; 0.16]†
Middle East and North Africa	1.15 [1.11; 1.19]	1.43 [1.35; 1.52]	0.28 [0.19; 0.38]†	4.4 [4.0; 5.4]	3.1 [2.8; 3.7]	0.99 [0.93; 1.03]	0.98 [0.87; 1.06]	0.00 [-0.12; 0.10]
Sub-Saharan Africa	1.05 [1.01; 1.09]	1.09 [1.04; 1.14]	0.04 [-0.03; 0.10]	16.2 [15.4; 18.6]	14.8 [14.1; 16.9]	0.99 [0.97; 1.02]	0.97 [0.93; 1.03]	-0.02 [-0.08; 0.05]
Latin America and Caribbean	1.34 [1.31; 1.37]	1.32 [1.27; 1.37]	-0.02 [-0.09; 0.04]	3.1 [2.9; 3.3]	2.3 [2.2; 2.5]	1.02 [0.99; 1.04]	1.08 [1.02; 1.13]*	0.06 [0.00; 0.12]
East Asia and Pacific	1.23 [1.16; 1.30]	1.49 [1.37; 1.61]	0.25 [0.12; 0.40]†	3.2 [2.7; 4.0]	2.1 [1.8; 2.7]	1.00 [0.93; 1.07]	0.95 [0.87; 1.04]	-0.05 [-0.16; 0.07]
North America	1.50 [1.46; 1.53]	1.30 [1.23; 1.38]	-0.20 [-0.28; -0.11]†	1.5 [1.4; 1.5]	1.1 [1.1; 1.2]	1.00 [0.97; 1.02]	1.05 [0.99; 1.11]	0.05 [-0.01; 0.12]
Afghanistan	1.03 [0.93; 1.14]	1.60 [1.34; 1.92]	0.58 [0.14; 0.92]†	4.9 [2.8; 9.7]	3.0 [1.8; 5.9]	1.01 [0.90; 1.14]	0.89 [0.63; 1.09]	-0.12 [-0.42; 0.11]
Albania	1.44 [1.36; 1.53]	1.15 [1.01; 1.31]	-0.29 [-0.47; -0.11]†	2.0 [1.7; 2.3]	1.7 [1.5; 2.0]	0.98 [0.92; 1.05]	1.24 [1.10; 1.40]*	0.26 [0.09; 0.43]†
Algeria	1.34 [1.25; 1.43]	1.23 [1.16; 1.30]	-0.11 [-0.23; 0.00]	3.1 [3.0; 3.3]	2.5 [2.4; 2.7]	0.93 [0.85; 1.02]	1.22 [1.15; 1.29]*	0.29 [0.18; 0.40]†
Andorra	1.51 [1.38; 1.65]	1.28 [1.08; 1.53]	-0.22 [-0.50; 0.08]	0.8 [0.7; 1.1]	0.7 [0.5; 0.9]	0.99 [0.91; 1.09]	1.00 [0.84; 1.19]	0.00 [-0.19; 0.22]
Angola	1.03 [0.93; 1.14]	1.06 [0.90; 1.26]	0.03 [-0.18; 0.26]	16.2 [12.3; 20.8]	15.2 [11.6; 19.7]	1.00 [0.91; 1.10]	1.01 [0.83; 1.23]	0.01 [-0.20; 0.27]
Anguilla	1.51 [1.38; 1.66]	1.36 [1.13; 1.63]	-0.16 [-0.45; 0.16]	1.3 [1.0; 1.7]	1.0 [0.8; 1.3]	1.00 [0.91; 1.09]	1.00 [0.84; 1.19]	0.00 [-0.19; 0.22]
Antigua and Barbuda	1.51 [1.38; 1.66]	1.45 [1.20; 1.73]	-0.07 [-0.37; 0.26]	1.8 [1.4; 2.3]	1.3 [1.0; 1.6]	1.00 [0.91; 1.09]	0.98 [0.82; 1.16]	-0.02 [-0.21; 0.20]
Argentina	1.49 [1.44; 1.55]	1.20 [1.12; 1.28]	-0.30 [-0.40; -0.20]†	1.6 [1.5; 1.7]	1.3 [1.2; 1.5]	1.01 [0.97; 1.06]	1.16 [1.08; 1.23]*	0.14 [0.06; 0.23]†
Armenia	1.60 [1.49; 1.72]	1.38 [1.22; 1.56]	-0.22 [-0.43; 0.00]	2.2 [2.0; 2.5]	1.6 [1.4; 1.8]	0.94 [0.88; 1.01]	1.04 [0.93; 1.17]	0.10 [-0.04; 0.25]
Australia	1.47 [1.39; 1.55]	1.26 [1.15; 1.39]	-0.21 [-0.34; -0.06]†	0.8 [0.8; 0.9]	0.7 [0.6; 0.7]	0.99 [0.94; 1.04]	1.02 [0.92; 1.11]	0.03 [-0.08; 0.14]
Austria	1.44 [1.34; 1.54]	1.21 [1.08; 1.36]	-0.23 [-0.40; -0.04]†	0.8 [0.7; 0.9]	0.7 [0.6; 0.8]	1.00 [0.94; 1.06]	1.06 [0.94; 1.19]	0.06 [-0.08; 0.21]
Azerbaijan	1.37 [1.30; 1.44]	1.37 [1.27; 1.48]	0.00 [-0.12; 0.13]	3.6 [3.4; 3.8]	2.6 [2.4; 2.8]	1.06 [1.00; 1.13]*	1.10 [1.02; 1.19]*	0.04 [-0.07; 0.14]
Bahamas	1.49 [1.35; 1.64]	1.45 [1.21; 1.73]	-0.04 [-0.33; 0.28]	2.6 [1.9; 3.6]	1.8 [1.4; 2.5]	1.00 [0.90; 1.10]	1.02 [0.86; 1.21]	0.02 [-0.18; 0.24]
Bahrain	1.50 [1.38; 1.64]	1.37 [1.22; 1.55]	-0.13 [-0.35; 0.10]	2.1 [1.7; 2.6]	1.5 [1.2; 1.9]	1.00 [0.92; 1.10]	1.04 [0.92; 1.17]	0.04 [-0.12; 0.20]
Bangladesh	0.98 [0.91; 1.06]	1.44 [1.28; 1.63]	0.46 [0.27; 0.67]†	5.3 [4.3; 6.5]	3.7 [2.9; 4.6]	1.04 [0.96; 1.13]	1.04 [0.91; 1.18]	0.00 [-0.17; 0.16]
Barbados	1.50 [1.37; 1.64]	1.54 [1.27; 1.86]	0.04 [-0.28; 0.41]	1.9 [1.2; 3.1]	1.3 [0.8; 2.0]	0.99 [0.90; 1.09]	0.95 [0.78; 1.15]	-0.04 [-0.25; 0.19]
Belarus	1.71 [1.62; 1.79]	1.23 [1.10; 1.38]	-0.47 [-0.63; -0.30]†	1.2 [1.0; 1.4]	1.0 [0.8; 1.2]	0.89 [0.84; 0.94]*	1.06 [0.94; 1.18]	0.17 [0.04; 0.31]†
Belgium	1.38 [1.29; 1.46]	1.29 [1.15; 1.43]	-0.09 [-0.25; 0.08]	0.9 [0.8; 1.0]	0.7 [0.6; 0.7]	1.06 [1.00; 1.12]	1.00 [0.89; 1.11]	-0.06 [-0.19; 0.08]
Belize	1.49 [1.36; 1.64]	1.44 [1.22; 1.69]	-0.06 [-0.33; 0.25]	3.3 [2.4; 4.3]	2.3 [1.7; 3.0]	1.00 [0.89; 1.10]	1.05 [0.88; 1.23]	0.05 [-0.16; 0.27]
Benin	1.02 [0.93; 1.12]	1.06 [0.92; 1.24]	0.04 [-0.14; 0.24]	19.2 [14.4; 26.3]	18.0 [13.6; 24.6]	1.01 [0.92; 1.10]	0.98 [0.83; 1.17]	-0.03 [-0.21; 0.19]
Bhutan	1.09 [1.00; 1.37]	1.25 [1.02; 1.55]	0.16 [-0.18; 0.54]	8.1 [4.7; 14.4]	6.5 [3.6; 11.7]	0.90 [0.74; 1.08]	0.98 [0.74; 1.29]	0.07 [-0.23; 0.43]
Bolivia (Plurinational State of)	1.12 [1.02; 1.23]	1.48 [1.25; 1.76]	0.36 [0.09; 0.67]†	4.7 [2.8; 8.4]	3.2 [1.9; 5.6]	1.02 [0.90; 1.16]	0.98 [0.74; 1.19]	-0.04 [-0.33; 0.22]
Bosnia and Herzegovina	1.56 [1.44; 1.69]	1.26 [1.12; 1.41]	-0.31 [-0.51; -0.11]†	1.3 [1.1; 1.6]	1.1 [0.9; 1.3]	0.96 [0.88; 1.03]	1.06 [0.95; 1.19]	0.11 [-0.03; 0.27]
Botswana	1.04 [0.94; 1.14]	1.46 [1.22; 1.74]	0.42 [0.14; 0.74]†	8.1 [5.8; 12.2]	5.5 [4.0; 8.4]	1.01 [0.90; 1.13]	0.89 [0.68; 1.13]	-0.12 [-0.37; 0.15]
Brazil	1.53 [1.42; 1.64]	1.40 [1.26; 1.56]	-0.12 [-0.31; 0.07]	2.6 [2.4; 2.7]	1.8 [1.7; 2.0]	0.99 [0.92; 1.07]	1.06 [0.96; 1.17]	0.07 [-0.06; 0.20]
British Virgin Islands	1.52 [1.38; 1.66]	1.49 [1.24; 1.79]	-0.03 [-0.34; 0.31]	2.7 [2.0; 3.4]	1.8 [1.4; 2.4]	1.00 [0.91; 1.09]	1.00 [0.83; 1.19]	0.00 [-0.20; 0.22]
Brunei	1.52 [1.39; 1.66]	1.44 [1.22; 1.71]	-0.08 [-0.37; 0.23]	2.3 [1.8; 3.1]	1.6 [1.2; 2.1]	0.99 [0.89; 1.09]	1.00 [0.85; 1.19]	0.02 [-0.18; 0.23]
Bulgaria	1.52 [1.44; 1.60]	1.33 [1.20; 1.47]	-0.19 [-0.35; -0.02]†	1.8 [1.6; 2.0]	1.4 [1.2; 1.5]	1.00 [0.95; 1.05]	1.06 [0.96; 1.17]	0.06 [-0.06; 0.18]
Burkina Faso	1.05 [0.96; 1.15]	1.10 [0.92; 1.30]	0.05 [-0.16; 0.28]	17.6 [11.3; 29.9]	16.0 [10.2; 27.3]	0.99 [0.90; 1.08]	0.96 [0.79; 1.19]	-0.03 [-0.23; 0.23]
Burundi	1.04 [0.94; 1.16]	1.07 [0.91; 1.27]	0.03 [-0.18; 0.27]	19.6 [12.4; 37.5]	18.3 [11.6; 35.2]	1.01 [0.92; 1.11]	0.97 [0.81; 1.18]	-0.04 [-0.24; 0.19]
Cambodia	0.99 [0.91; 1.09]	1.49 [1.25; 1.77]	0.49 [0.22; 0.80]†	5.2 [3.3; 8.9]	3.5 [2.2; 6.0]	1.03 [0.94; 1.14]	0.96 [0.74; 1.17]	-0.07 [-0.33; 0.18]
Cameroon	1.02 [0.93; 1.11]	1.15 [0.98; 1.34]	0.13 [-0.07; 0.35]	22.5 [16.4; 30.4]	19.6 [14.1; 26.5]	1.00 [0.91; 1.09]	0.90 [0.76; 1.07]	-0.10 [-0.28; 0.10]
Canada	1.43 [1.37; 1.50]	1.18 [1.08; 1.28]	-0.26 [-0.38; -0.14]†	1.0 [0.9; 1.0]	0.8 [0.8; 0.9]	1.03 [0.98; 1.07]	1.10 [1.01; 1.20]*	0.07 [-0.03; 0.18]

¶: Sex ratio is outlying in 1990. §: Sex ratio is outlying in 2021. *: The ratio of estimated to expected female mortality is significantly different from one. †: Change is significantly different from zero.

Continued on next page

Table 18 – continued from previous page

	Sex ratio 10q5			Sex-specific 10q5 in 2021 (per 1,000)		Estimated/Expected female 10q5		
	1990	2021	Change 1990–2021	Male	Female	1990	2021	Change 1990–2021
Cape Verde	1.43 [1.31; 1.56]	1.28 [1.10; 1.48]	-0.15 [-0.39; 0.09]	2.1 [1.6; 2.7]	1.6 [1.3; 2.1]	0.99 [0.87; 1.11]	1.13 [0.98; 1.29]	0.14 [-0.06; 0.35]
Central African Republic	1.02 [0.94; 1.12]	0.91 [0.77; 1.07]	-0.11 [-0.29; 0.08]	22.4 [15.3; 34.1]	24.5 [16.8; 37.8]	0.99 [0.90; 1.09]	1.13 [0.94; 1.37]	0.14 [-0.08; 0.40]
Chad	1.04 [0.95; 1.14]	1.01 [0.88; 1.16]	-0.03 [-0.21; 0.16]	22.8 [17.4; 30.1]	22.5 [17.1; 29.8]	1.01 [0.92; 1.10]	1.01 [0.87; 1.18]	0.00 [-0.17; 0.20]
Chile	1.52 [1.45; 1.59]	1.22 [1.11; 1.34]	-0.30 [-0.44; -0.15]†	1.4 [1.3; 1.5]	1.1 [1.0; 1.2]	1.00 [0.95; 1.04]	1.11 [1.01; 1.21]*	0.11 [0.00; 0.23]
China	1.37 [1.24; 1.51]	1.57 [1.34; 1.85]	0.20 [-0.09; 0.52]	2.2 [1.4; 3.4]	1.4 [0.9; 2.1]	0.98 [0.84; 1.10]	0.91 [0.77; 1.08]	-0.07 [-0.26; 0.16]
Colombia	1.52 [1.40; 1.65]	1.39 [1.25; 1.55]	-0.13 [-0.33; 0.07]	2.7 [2.5; 2.9]	1.9 [1.8; 2.1]	0.98 [0.89; 1.07]	1.07 [0.97; 1.19]	0.09 [-0.06; 0.24]
Comoros	1.05 [0.95; 1.16]	1.36 [1.14; 1.63]	0.32 [0.05; 0.61]†	6.1 [4.3; 9.3]	4.5 [3.3; 6.5]	1.01 [0.89; 1.16]	1.01 [0.77; 1.26]	0.00 [-0.29; 0.30]
Congo	1.02 [0.92; 1.13]	1.41 [1.17; 1.69]	0.38 [0.09; 0.70]†	7.8 [4.7; 13.1]	5.5 [3.4; 9.3]	1.00 [0.90; 1.13]	0.92 [0.69; 1.18]	-0.08 [-0.34; 0.22]
Democratic Republic of the Congo	1.02 [0.92; 1.13]	1.03 [0.88; 1.22]	0.01 [-0.19; 0.23]	20.3 [14.3; 29.0]	19.6 [13.8; 27.9]	1.01 [0.91; 1.12]	1.00 [0.84; 1.20]	-0.01 [-0.21; 0.22]
Cook Islands	1.51 [1.38; 1.65]	1.45 [1.21; 1.72]	-0.06 [-0.36; 0.26]	2.2 [1.7; 2.8]	1.5 [1.1; 2.0]	0.98 [0.88; 1.08]	0.99 [0.84; 1.18]	0.01 [-0.19; 0.24]
Costa Rica	1.42 [1.32; 1.53]	1.25 [1.11; 1.41]	-0.17 [-0.36; 0.03]	1.8 [1.7; 2.0]	1.5 [1.3; 1.6]	1.06 [0.98; 1.14]	1.13 [1.01; 1.26]*	0.07 [-0.08; 0.23]
Cote d'Ivoire	1.03 [0.94; 1.13]	0.96 [0.81; 1.13]	-0.07 [-0.25; 0.12]	23.1 [15.6; 37.3]	24.2 [16.3; 39.5]	0.99 [0.90; 1.08]	1.08 [0.90; 1.30]	0.09 [-0.12; 0.33]
Croatia	1.54 [1.44; 1.66]	1.34 [1.17; 1.54]	-0.20 [-0.41; 0.03]	1.4 [1.2; 1.6]	1.1 [0.9; 1.2]	0.97 [0.91; 1.04]	1.00 [0.87; 1.13]	0.02 [-0.13; 0.18]
Cuba	1.50 [1.42; 1.59]	1.35 [1.23; 1.49]	-0.15 [-0.31; 0.01]	2.1 [1.8; 2.3]	1.5 [1.4; 1.7]	1.01 [0.95; 1.07]	1.06 [0.97; 1.16]	0.05 [-0.06; 0.17]
Cyprus	1.40 [1.28; 1.53]	1.36 [1.14; 1.61]	-0.05 [-0.31; 0.25]	1.3 [1.0; 1.7]	1.0 [0.8; 1.3]	1.00 [0.91; 1.10]	0.99 [0.84; 1.17]	-0.01 [-0.20; 0.20]
Czech Republic	1.55 [1.46; 1.64]	1.19 [1.07; 1.33]	-0.36 [-0.52; -0.19]†	0.9 [0.8; 1.0]	0.8 [0.7; 0.9]	0.96 [0.90; 1.01]	1.08 [0.97; 1.20]	0.12 [-0.01; 0.26]
Denmark	1.46 [1.35; 1.57]	1.25 [1.10; 1.42]	-0.21 [-0.41; 0.00]	0.7 [0.6; 0.9]	0.6 [0.5; 0.7]	0.99 [0.92; 1.06]	1.03 [0.91; 1.17]	0.04 [-0.11; 0.21]
Djibouti	1.01 [0.92; 1.11]	1.12 [0.94; 1.34]	0.11 [-0.11; 0.35]	13.1 [10.0; 17.0]	11.7 [9.0; 15.2]	1.00 [0.91; 1.10]	1.01 [0.81; 1.27]	0.01 [-0.22; 0.29]
Dominica	1.52 [1.39; 1.67]	1.52 [1.28; 1.81]	0.00 [-0.30; 0.33]	3.6 [2.2; 6.1]	2.4 [1.5; 3.9]	0.97 [0.81; 1.08]	0.97 [0.77; 1.17]	-0.01 [-0.24; 0.26]
Dominican Republic	1.36 [1.25; 1.49]	1.50 [1.26; 1.77]	0.14 [-0.14; 0.44]	3.8 [2.5; 6.3]	2.5 [1.7; 4.1]	0.99 [0.86; 1.12]	0.99 [0.79; 1.18]	-0.01 [-0.25; 0.23]
Ecuador	1.29 [1.23; 1.35]	1.25 [1.16; 1.34]	-0.04 [-0.15; 0.07]	2.6 [2.5; 2.8]	2.1 [2.0; 2.3]	1.06 [0.99; 1.12]	1.19 [1.11; 1.28]*	0.13 [0.03; 0.24]†
Egypt	1.15 [1.07; 1.23]	1.63 [1.45; 1.82]	0.47 [0.28; 0.69]†	5.0 [4.6; 5.5]	3.1 [2.8; 3.4]	1.01 [0.93; 1.10]	0.93 [0.83; 1.04]	-0.08 [-0.22; 0.06]
El Salvador	1.36 [1.29; 1.44]	1.41 [1.26; 1.57]	0.04 [-0.13; 0.22]	4.4 [3.9; 4.8]	3.1 [2.8; 3.5]	1.04 [0.97; 1.11]	1.07 [0.95; 1.20]	0.03 [-0.11; 0.18]
Equatorial Guinea	1.01 [0.92; 1.12]	1.06 [0.88; 1.27]	0.04 [-0.17; 0.29]	15.9 [12.3; 20.7]	15.0 [11.5; 19.6]	1.00 [0.91; 1.10]	1.01 [0.82; 1.25]	0.01 [-0.22; 0.28]
Eritrea	1.04 [0.95; 1.14]	1.39 [1.17; 1.65]	0.35 [0.09; 0.64]†	8.2 [4.5; 17.0]	5.9 [3.2; 12.6]	0.99 [0.90; 1.09]	0.92 [0.69; 1.20]	-0.07 [-0.32; 0.23]
Estonia	1.56 [1.45; 1.69]	1.17 [1.01; 1.36]	-0.39 [-0.61; -0.16]†	1.1 [0.9; 1.4]	0.9 [0.7; 1.2]	0.96 [0.89; 1.04]	1.11 [0.96; 1.29]	0.15 [-0.04; 0.35]
Ethiopia	1.11 [1.00; 1.23]	1.41 [1.19; 1.66]	0.30 [0.04; 0.58]†	8.3 [5.3; 13.1]	5.9 [3.8; 9.2]	0.98 [0.89; 1.07]	0.91 [0.69; 1.16]	-0.07 [-0.31; 0.21]
Federated States of Micronesia	1.25 [1.14; 1.38]	1.49 [1.25; 1.78]	0.24 [-0.05; 0.55]	6.2 [4.8; 8.0]	4.2 [3.1; 5.4]	1.00 [0.85; 1.16]	0.99 [0.81; 1.20]	-0.01 [-0.24; 0.25]
Fiji	1.12 [1.02; 1.24]	1.43 [1.26; 1.62]	0.31 [0.09; 0.54]†	5.8 [5.0; 6.8]	4.1 [3.5; 4.8]	1.02 [0.89; 1.18]	1.03 [0.87; 1.19]	0.01 [-0.22; 0.22]
Finland	1.45 [1.35; 1.57]	1.09 [0.96; 1.24]	-0.36 [-0.54; -0.17]†	0.7 [0.6; 0.9]	0.7 [0.6; 0.8]	0.98 [0.92; 1.05]	1.17 [1.03; 1.33]*	0.19 [0.02; 0.36]†
France	1.37 [1.31; 1.42]	1.31 [1.21; 1.41]	-0.06 [-0.17; 0.06]	0.8 [0.8; 0.9]	0.6 [0.6; 0.7]	1.06 [1.02; 1.10]*	0.98 [0.90; 1.06]	-0.08 [-0.17; 0.01]
Gabon	1.04 [0.95; 1.15]	1.13 [0.94; 1.34]	0.08 [-0.14; 0.33]	12.2 [6.3; 25.4]	10.9 [5.5; 23.4]	1.00 [0.90; 1.13]	1.00 [0.79; 1.34]	0.00 [-0.25; 0.36]
The Gambia	1.01 [0.92; 1.11]	1.24 [1.05; 1.45]	0.23 [0.00; 0.47]†	10.8 [8.4; 14.0]	8.8 [6.8; 11.4]	1.00 [0.91; 1.10]	0.97 [0.79; 1.22]	-0.03 [-0.25; 0.24]
Georgia	1.56 [1.44; 1.69]	1.44 [1.26; 1.65]	-0.11 [-0.35; 0.14]	2.1 [1.9; 2.4]	1.5 [1.3; 1.7]	0.97 [0.90; 1.05]	1.00 [0.88; 1.13]	0.03 [-0.12; 0.19]
Germany	1.40 [1.35; 1.46]	1.22 [1.14; 1.31]	-0.18 [-0.28; -0.08]†	0.8 [0.8; 0.9]	0.7 [0.6; 0.7]	1.02 [0.98; 1.06]	1.05 [0.98; 1.12]	0.02 [-0.06; 0.11]
Ghana	1.06 [0.97; 1.16]	1.16 [0.99; 1.35]	0.10 [-0.11; 0.32]	10.8 [8.1; 14.6]	9.3 [6.9; 12.7]	0.96 [0.88; 1.06]	1.03 [0.83; 1.28]	0.07 [-0.16; 0.34]
Greece	1.41 [1.33; 1.50]	1.36 [1.22; 1.53]	-0.05 [-0.23; 0.14]	0.9 [0.8; 1.0]	0.6 [0.6; 0.7]	1.02 [0.96; 1.08]	0.94 [0.84; 1.06]	-0.08 [-0.20; 0.06]
Grenada	1.51 [1.37; 1.65]	1.57 [1.31; 1.87]	0.06 [-0.26; 0.41]	4.7 [3.3; 6.6]	3.0 [2.1; 4.2]	0.98 [0.87; 1.08]	0.95 [0.75; 1.15]	-0.03 [-0.27; 0.20]
Guatemala¶	1.00 [0.97; 1.04]	1.20 [1.10; 1.31]	0.20 [0.09; 0.31]†	3.7 [3.5; 3.9]	3.1 [2.9; 3.3]	1.13 [1.08; 1.19]*	1.26 [1.16; 1.37]*	0.13 [0.01; 0.26]†
Guinea	1.02 [0.93; 1.12]	1.02 [0.87; 1.20]	0.00 [-0.19; 0.21]	18.0 [13.6; 25.3]	17.6 [13.3; 24.7]	1.01 [0.92; 1.10]	1.03 [0.86; 1.24]	0.02 [-0.18; 0.26]
Guinea-Bissau	1.07 [0.95; 1.18]	1.11 [0.93; 1.33]	0.05 [-0.24; 0.34]	13.9 [9.5; 21.2]	12.4 [8.4; 19.4]	0.99 [0.88; 1.11]	0.99 [0.81; 1.23]	0.00 [-0.23; 0.27]
Guyana	1.44 [1.33; 1.56]	1.39 [1.19; 1.62]	-0.05 [-0.29; 0.22]	5.4 [4.0; 7.4]	3.9 [2.9; 5.3]	1.03 [0.94; 1.12]	1.05 [0.83; 1.25]	0.02 [-0.22; 0.25]
Haiti	1.01 [0.92; 1.11]	1.06 [0.88; 1.27]	0.04 [-0.18; 0.28]	12.2 [8.0; 19.0]	11.5 [7.9; 17.5]	1.00 [0.91; 1.10]	1.04 [0.81; 1.37]	0.04 [-0.22; 0.39]
Honduras	1.25 [1.13; 1.37]	1.32 [1.10; 1.58]	0.07 [-0.19; 0.37]	5.5 [3.6; 9.1]	4.1 [2.7; 7.0]	0.99 [0.86; 1.14]	1.07 [0.78; 1.33]	0.08 [-0.25; 0.38]
Hungary	1.46 [1.38; 1.55]	1.34 [1.19; 1.49]	-0.13 [-0.30; 0.05]	1.1 [1.0; 1.2]	0.8 [0.7; 0.9]	1.02 [0.97; 1.08]	0.97 [0.87; 1.08]	-0.05 [-0.17; 0.08]
Iceland	1.44 [1.31; 1.57]	1.28 [1.06; 1.53]	-0.16 [-0.42; 0.14]	0.6 [0.4; 0.9]	0.5 [0.3; 0.7]	0.99 [0.91; 1.09]	1.01 [0.84; 1.21]	0.01 [-0.19; 0.24]

¶: Sex ratio is outlying in 1990. §: Sex ratio is outlying in 2021. *: The ratio of estimated to expected female mortality is significantly different from one. †: Change is significantly different from zero.

Continued on next page

Table 18 – continued from previous page

	Sex ratio 10q5			Sex-specific 10q5 in 2021 (per 1,000)		Estimated/Expected female 10q5		
	1990	2021	Change 1990–2021	Male	Female	1990	2021	Change 1990–2021
India¶	0.92 [0.87; 0.98]	1.29 [1.15; 1.44]	0.37 [0.22; 0.54]†	4.5 [4.0; 5.1]	3.5 [3.1; 4.0]	1.12 [1.05; 1.21]*	1.17 [1.04; 1.32]*	0.05 [-0.12; 0.21]
Indonesia	1.05 [0.97; 1.14]	1.49 [1.26; 1.75]	0.44 [0.18; 0.72]†	5.6 [4.1; 8.2]	3.8 [2.8; 5.4]	1.06 [0.96; 1.18]	0.98 [0.77; 1.17]	-0.09 [-0.33; 0.14]
Iran (Islamic Republic of)	1.04 [0.95; 1.14]	1.34 [1.18; 1.52]	0.30 [0.10; 0.52]†	3.6 [3.1; 4.2]	2.7 [2.3; 3.2]	0.98 [0.84; 1.12]	1.13 [0.99; 1.28]	0.14 [-0.06; 0.36]
Iraq	1.19 [1.08; 1.30]	1.44 [1.22; 1.70]	0.25 [-0.01; 0.54]	6.1 [3.6; 12.1]	4.2 [2.5; 8.3]	0.98 [0.85; 1.14]	0.97 [0.69; 1.21]	-0.01 [-0.34; 0.28]
Ireland	1.49 [1.39; 1.60]	1.50 [1.34; 1.69]	0.01 [-0.20; 0.22]	0.7 [0.6; 0.8]	0.5 [0.4; 0.5]	0.97 [0.91; 1.04]	0.85 [0.76; 0.96]*	-0.12 [-0.24; 0.01]
Israel	1.35 [1.26; 1.45]	1.34 [1.22; 1.49]	-0.01 [-0.17; 0.17]	0.9 [0.8; 1.0]	0.7 [0.6; 0.7]	1.06 [1.00; 1.13]	0.95 [0.86; 1.06]	-0.11 [-0.23; 0.01]
Italy	1.46 [1.40; 1.53]	1.27 [1.17; 1.38]	-0.20 [-0.32; -0.07]†	0.7 [0.6; 0.8]	0.5 [0.5; 0.6]	0.99 [0.95; 1.03]	1.01 [0.93; 1.10]	0.02 [-0.07; 0.13]
Jamaica	1.52 [1.38; 1.66]	1.48 [1.24; 1.76]	-0.04 [-0.33; 0.28]	3.0 [2.3; 3.8]	2.0 [1.6; 2.6]	0.99 [0.90; 1.09]	1.01 [0.86; 1.21]	0.02 [-0.17; 0.25]
Japan	1.49 [1.44; 1.54]	1.23 [1.15; 1.30]	-0.26 [-0.35; -0.17]†	0.8 [0.8; 0.8]	0.7 [0.6; 0.7]	0.94 [0.91; 0.98]*	1.04 [0.98; 1.12]	0.10 [0.03; 0.18]†
Jordan	1.50 [1.38; 1.65]	1.47 [1.23; 1.76]	-0.03 [-0.33; 0.29]	2.8 [1.7; 5.0]	1.9 [1.2; 3.3]	0.97 [0.85; 1.08]	0.99 [0.81; 1.19]	0.01 [-0.20; 0.26]
Kazakhstan	1.76 [1.69; 1.83]	1.52 [1.43; 1.61]	-0.24 [-0.36; -0.12]†	3.1 [3.0; 3.2]	2.0 [1.9; 2.1]	0.81 [0.77; 0.86]*	0.99 [0.93; 1.05]	0.17 [0.10; 0.25]†
Kenya	1.08 [0.99; 1.19]	1.21 [1.02; 1.42]	0.12 [-0.10; 0.36]	9.9 [6.0; 18.4]	8.2 [5.0; 15.2]	0.98 [0.88; 1.10]	1.00 [0.77; 1.32]	0.02 [-0.25; 0.36]
Kiribati	1.06 [0.97; 1.17]	1.25 [1.04; 1.49]	0.19 [-0.06; 0.45]	10.0 [7.7; 12.9]	8.0 [6.1; 10.5]	1.00 [0.90; 1.14]	1.01 [0.80; 1.29]	0.01 [-0.23; 0.29]
Democratic People's Republic of Korea	1.31 [1.19; 1.43]	1.50 [1.26; 1.79]	0.20 [-0.09; 0.52]	4.2 [3.2; 5.4]	2.8 [2.1; 3.6]	0.99 [0.85; 1.15]	1.00 [0.84; 1.20]	0.01 [-0.23; 0.26]
Republic of Korea	1.47 [1.36; 1.58]	1.32 [1.18; 1.47]	-0.15 [-0.33; 0.04]	0.9 [0.8; 0.9]	0.6 [0.6; 0.7]	1.03 [0.95; 1.11]	0.97 [0.87; 1.08]	-0.05 [-0.19; 0.09]
Kosovo	1.36 [1.23; 1.48]	1.46 [1.22; 1.73]	0.10 [-0.18; 0.41]	2.5 [1.9; 3.2]	1.7 [1.3; 2.2]	1.00 [0.85; 1.14]	1.00 [0.85; 1.19]	0.00 [-0.23; 0.27]
Kuwait	1.43 [1.32; 1.55]	1.32 [1.17; 1.50]	-0.10 [-0.31; 0.11]	2.0 [1.8; 2.3]	1.5 [1.3; 1.8]	0.98 [0.90; 1.06]	1.07 [0.95; 1.21]	0.09 [-0.06; 0.26]
Kyrgyzstan	1.54 [1.45; 1.63]	1.60 [1.48; 1.73]	0.06 [-0.09; 0.22]	3.3 [3.1; 3.5]	2.1 [1.9; 2.2]	0.95 [0.89; 1.02]	0.94 [0.87; 1.02]	-0.01 [-0.10; 0.09]
Lao People's Democratic Republic	1.04 [0.94; 1.15]	1.35 [1.14; 1.60]	0.31 [0.05; 0.58]†	7.8 [5.0; 11.5]	5.8 [3.6; 8.7]	1.00 [0.90; 1.11]	0.94 [0.72; 1.21]	-0.05 [-0.31; 0.24]
Latvia	1.70 [1.59; 1.82]	1.09 [0.95; 1.26]	-0.61 [-0.81; -0.39]†	1.0 [0.8; 1.2]	0.9 [0.7; 1.1]	0.85 [0.79; 0.92]*	1.18 [1.02; 1.36]*	0.32 [0.14; 0.52]†
Lebanon	1.38 [1.26; 1.52]	1.45 [1.21; 1.73]	0.06 [-0.23; 0.38]	2.3 [1.8; 3.0]	1.6 [1.2; 2.1]	1.00 [0.85; 1.14]	1.00 [0.84; 1.18]	0.00 [-0.24; 0.26]
Lesotho	1.05 [0.95; 1.15]	1.26 [1.06; 1.49]	0.22 [-0.02; 0.48]	9.3 [6.5; 13.1]	7.4 [5.2; 10.5]	1.01 [0.91; 1.14]	1.00 [0.78; 1.27]	-0.01 [-0.28; 0.29]
Liberia	1.00 [0.91; 1.10]	1.17 [0.98; 1.39]	0.17 [-0.06; 0.43]	18.3 [12.5; 29.4]	15.6 [10.4; 25.8]	1.02 [0.92; 1.12]	0.90 [0.74; 1.09]	-0.12 [-0.32; 0.10]
Libya	1.36 [1.22; 1.51]	1.52 [1.25; 1.87]	0.16 [-0.20; 0.56]	3.2 [1.5; 8.8]	2.1 [1.0; 5.6]	0.92 [0.75; 1.07]	0.95 [0.67; 1.16]	0.02 [-0.28; 0.29]
Lithuania	1.61 [1.51; 1.72]	1.27 [1.11; 1.44]	-0.35 [-0.55; -0.14]†	1.4 [1.2; 1.7]	1.1 [1.0; 1.3]	0.94 [0.88; 1.00]	1.07 [0.95; 1.21]	0.13 [-0.01; 0.29]
Luxembourg	1.42 [1.30; 1.56]	1.31 [1.10; 1.56]	-0.11 [-0.37; 0.17]	0.4 [0.2; 0.6]	0.3 [0.2; 0.5]	0.99 [0.90; 1.08]	0.98 [0.82; 1.17]	-0.01 [-0.20; 0.21]
Macedonia	1.48 [1.37; 1.61]	1.32 [1.13; 1.54]	-0.16 [-0.41; 0.10]	1.7 [1.4; 2.0]	1.3 [1.1; 1.6]	1.02 [0.94; 1.10]	1.06 [0.91; 1.23]	0.04 [-0.14; 0.24]
Madagascar	1.02 [0.94; 1.12]	1.04 [0.91; 1.20]	0.02 [-0.15; 0.21]	21.6 [15.4; 33.3]	20.7 [15.0; 31.2]	0.99 [0.91; 1.08]	0.99 [0.84; 1.17]	0.00 [-0.19; 0.20]
Malawi	1.01 [0.93; 1.11]	1.21 [1.05; 1.39]	0.19 [0.00; 0.41]	13.2 [10.7; 16.9]	10.9 [8.8; 13.9]	1.01 [0.93; 1.10]	0.93 [0.78; 1.11]	-0.08 [-0.27; 0.13]
Malaysia	1.48 [1.40; 1.56]	1.37 [1.29; 1.46]	-0.10 [-0.22; 0.01]	2.0 [1.9; 2.1]	1.5 [1.4; 1.6]	1.01 [0.95; 1.08]	1.05 [0.99; 1.11]	0.03 [-0.05; 0.12]
Maldives	1.18 [1.08; 1.30]	1.36 [1.13; 1.61]	0.17 [-0.09; 0.46]	1.6 [1.2; 2.1]	1.2 [0.9; 1.6]	1.08 [0.95; 1.21]	1.03 [0.87; 1.22]	-0.05 [-0.26; 0.19]
Mali	1.02 [0.94; 1.11]	1.03 [0.88; 1.19]	0.00 [-0.17; 0.19]	21.5 [16.3; 28.6]	20.9 [15.5; 28.5]	1.00 [0.93; 1.09]	1.01 [0.86; 1.19]	0.00 [-0.18; 0.20]
Malta	1.39 [1.27; 1.53]	1.34 [1.11; 1.61]	-0.06 [-0.33; 0.25]	1.0 [0.7; 1.4]	0.8 [0.5; 1.1]	0.98 [0.88; 1.11]	0.99 [0.83; 1.20]	0.01 [-0.21; 0.26]
Marshall Islands	1.27 [1.16; 1.39]	1.44 [1.20; 1.73]	0.17 [-0.12; 0.49]	7.0 [5.4; 9.1]	4.9 [3.7; 6.4]	0.99 [0.85; 1.16]	0.99 [0.80; 1.22]	0.00 [-0.23; 0.26]
Mauritania	1.03 [0.94; 1.13]	1.34 [1.15; 1.56]	0.31 [0.08; 0.56]†	9.2 [6.2; 13.8]	6.9 [4.6; 10.4]	1.01 [0.91; 1.13]	0.93 [0.73; 1.17]	-0.08 [-0.31; 0.18]
Mauritius	1.43 [1.31; 1.55]	1.33 [1.14; 1.55]	-0.09 [-0.34; 0.16]	2.0 [1.7; 2.3]	1.5 [1.2; 1.8]	1.06 [0.98; 1.15]	1.07 [0.92; 1.24]	0.01 [-0.18; 0.21]
Mexico	1.38 [1.35; 1.42]	1.31 [1.25; 1.38]	-0.07 [-0.14; 0.01]	2.8 [2.7; 2.9]	2.1 [2.0; 2.2]	1.08 [1.05; 1.11]*	1.14 [1.08; 1.20]*	0.06 [-0.01; 0.13]
Republic of Moldova	1.58 [1.48; 1.68]	1.41 [1.25; 1.60]	-0.16 [-0.36; 0.05]	2.8 [2.5; 3.2]	2.0 [1.8; 2.3]	0.93 [0.87; 0.99]*	1.06 [0.94; 1.19]	0.13 [-0.02; 0.28]
Monaco	1.43 [1.30; 1.57]	1.28 [1.07; 1.54]	-0.15 [-0.42; 0.14]	0.9 [0.7; 1.1]	0.7 [0.5; 0.9]	1.00 [0.91; 1.10]	1.00 [0.84; 1.20]	0.00 [-0.19; 0.22]
Mongolia	1.18 [1.09; 1.27]	1.68 [1.48; 1.90]	0.50 [0.27; 0.74]†	4.0 [3.5; 4.5]	2.4 [2.1; 2.7]	0.98 [0.89; 1.09]	0.91 [0.80; 1.02]	-0.08 [-0.24; 0.08]
Montenegro	1.48 [1.35; 1.61]	1.34 [1.12; 1.60]	-0.14 [-0.41; 0.16]	0.9 [0.7; 1.3]	0.7 [0.5; 0.9]	1.00 [0.91; 1.10]	0.97 [0.82; 1.16]	-0.03 [-0.22; 0.18]
Montserrat	1.53 [1.40; 1.68]	1.54 [1.29; 1.84]	0.01 [-0.30; 0.35]	1.6 [1.2; 2.0]	1.0 [0.8; 1.3]	0.99 [0.90; 1.08]	0.89 [0.75; 1.05]	-0.10 [-0.28; 0.09]
Morocco	1.24 [1.13; 1.35]	1.43 [1.20; 1.70]	0.19 [-0.07; 0.50]	2.8 [1.8; 4.7]	2.0 [1.2; 3.3]	1.00 [0.87; 1.13]	1.02 [0.84; 1.23]	0.02 [-0.21; 0.27]
Mozambique	1.05 [0.95; 1.17]	1.11 [0.93; 1.32]	0.05 [-0.17; 0.31]	14.0 [8.6; 24.4]	12.6 [7.6; 22.4]	1.01 [0.92; 1.11]	0.99 [0.80; 1.27]	-0.01 [-0.25; 0.27]
Myanmar	1.03 [0.93; 1.15]	1.51 [1.26; 1.83]	0.48 [0.15; 0.84]†	5.1 [2.9; 8.9]	3.3 [1.9; 5.9]	1.00 [0.89; 1.12]	0.94 [0.69; 1.16]	-0.05 [-0.33; 0.20]
Namibia	1.10 [1.00; 1.21]	1.07 [0.90; 1.26]	-0.04 [-0.24; 0.19]	13.3 [9.2; 19.8]	12.4 [8.4; 19.0]	0.98 [0.87; 1.12]	1.04 [0.85; 1.31]	0.06 [-0.19; 0.36]

¶: Sex ratio is outlying in 1990. §: Sex ratio is outlying in 2021. *: The ratio of estimated to expected female mortality is significantly different from one. †: Change is significantly different from zero.

Continued on next page

Table 18 – continued from previous page

	Sex ratio 10q5			Sex-specific 10q5 in 2021 (per 1,000)		Estimated/Expected female 10q5		
	**1990**	**2021**	**Change 1990–2021**	**Male**	**Female**	**1990**	**2021**	**Change 1990–2021**
Nauru	1.14 [1.03; 1.25]	1.47 [1.23; 1.75]	0.33 [0.05; 0.64]†	6.7 [5.2; 8.6]	4.5 [3.5; 6.0]	1.00 [0.88; 1.17]	0.99 [0.80; 1.21]	-0.01 [-0.25; 0.23]
Nepal	0.99 [0.91; 1.08]	1.43 [1.20; 1.70]	0.44 [0.19; 0.72]†	5.5 [3.8; 9.3]	3.9 [2.6; 6.5]	1.03 [0.95; 1.14]	1.01 [0.78; 1.22]	-0.02 [-0.28; 0.21]
Netherlands	1.36 [1.28; 1.44]	1.20 [1.09; 1.32]	-0.15 [-0.29; -0.01]†	0.8 [0.7; 0.9]	0.6 [0.6; 0.7]	1.05 [0.99; 1.11]	1.07 [0.97; 1.18]	0.02 [-0.10; 0.14]
New Zealand	1.45 [1.36; 1.57]	1.33 [1.21; 1.48]	-0.12 [-0.30; 0.06]	1.2 [1.0; 1.3]	0.9 [0.8; 1.0]	1.03 [0.96; 1.10]	0.99 [0.89; 1.09]	-0.04 [-0.17; 0.09]
Nicaragua	1.35 [1.21; 1.49]	1.51 [1.24; 1.83]	0.16 [-0.17; 0.53]	3.9 [2.0; 8.1]	2.6 [1.4; 5.3]	0.96 [0.81; 1.10]	0.96 [0.70; 1.19]	0.00 [-0.30; 0.28]
Niger	1.10 [1.00; 1.20]	0.99 [0.86; 1.14]	-0.11 [-0.29; 0.08]	32.4 [25.2; 42.9]	32.9 [25.5; 43.6]	0.98 [0.90; 1.07]	1.03 [0.89; 1.20]	0.05 [-0.13; 0.25]
Nigeria	1.03 [0.94; 1.12]	1.08 [0.95; 1.23]	0.06 [-0.11; 0.24]	20.4 [15.6; 27.5]	18.8 [14.5; 25.1]	0.99 [0.91; 1.08]	0.96 [0.83; 1.11]	-0.04 [-0.20; 0.14]
Niue	1.58 [1.45; 1.73]	1.53 [1.28; 1.82]	-0.05 [-0.36; 0.29]	6.1 [4.7; 7.9]	4.0 [3.1; 5.2]	0.93 [0.82; 1.03]	0.97 [0.79; 1.17]	0.04 [-0.16; 0.26]
Norway	1.47 [1.36; 1.58]	1.06 [0.93; 1.21]	-0.41 [-0.60; -0.22]†	0.7 [0.6; 0.8]	0.6 [0.6; 0.7]	0.97 [0.91; 1.05]	1.21 [1.06; 1.38]*	0.23 [0.06; 0.43]†
Oman	1.29 [1.18; 1.43]	1.49 [1.24; 1.77]	0.19 [-0.11; 0.52]	2.8 [2.1; 3.6]	1.9 [1.4; 2.4]	1.00 [0.86; 1.18]	1.00 [0.83; 1.19]	-0.01 [-0.26; 0.26]
Pakistan	1.01 [0.94; 1.09]	1.14 [0.97; 1.35]	0.13 [-0.06; 0.36]	8.1 [5.4; 13.6]	7.1 [4.6; 11.9]	1.09 [1.00; 1.19]	1.14 [0.88; 1.43]	0.06 [-0.24; 0.37]
Palau	1.42 [1.30; 1.56]	1.54 [1.29; 1.84]	0.12 [-0.19; 0.46]	4.4 [3.4; 5.7]	2.8 [2.2; 3.7]	0.96 [0.83; 1.10]	0.98 [0.82; 1.17]	0.02 [-0.21; 0.26]
Panama	1.42 [1.31; 1.54]	1.31 [1.17; 1.46]	-0.11 [-0.30; 0.08]	3.4 [3.2; 3.7]	2.6 [2.4; 2.9]	1.06 [0.97; 1.15]	1.16 [1.04; 1.29]*	0.09 [-0.06; 0.26]
Papua New Guinea	1.08 [0.98; 1.19]	1.24 [1.04; 1.48]	0.17 [-0.07; 0.43]	9.0 [7.0; 11.6]	7.2 [5.6; 9.4]	1.00 [0.90; 1.15]	1.06 [0.84; 1.33]	0.05 [-0.19; 0.33]
Paraguay	1.39 [1.27; 1.53]	1.54 [1.27; 1.87]	0.14 [-0.17; 0.51]	3.2 [1.7; 5.9]	2.1 [1.1; 3.8]	0.95 [0.81; 1.08]	0.96 [0.73; 1.17]	0.00 [-0.26; 0.27]
Peru	1.25 [1.15; 1.36]	1.33 [1.14; 1.57]	0.09 [-0.15; 0.35]	2.8 [2.0; 4.0]	2.1 [1.5; 3.1]	0.97 [0.86; 1.09]	1.10 [0.93; 1.30]	0.13 [-0.09; 0.36]
Philippines	1.28 [1.19; 1.39]	1.41 [1.25; 1.58]	0.12 [-0.07; 0.33]	5.3 [4.9; 5.8]	3.8 [3.5; 4.2]	1.01 [0.91; 1.12]	1.08 [0.96; 1.21]	0.07 [-0.11; 0.24]
Poland	1.57 [1.52; 1.64]	1.14 [1.06; 1.23]	-0.43 [-0.54; -0.32]†	1.0 [0.9; 1.1]	0.9 [0.8; 0.9]	0.96 [0.92; 0.99]*	1.13 [1.04; 1.22]*	0.17 [0.08; 0.27]†
Portugal	1.57 [1.49; 1.65]	1.14 [1.02; 1.27]	-0.42 [-0.58; -0.27]†	0.9 [0.8; 1.0]	0.8 [0.7; 0.9]	0.97 [0.92; 1.02]	1.12 [1.01; 1.25]*	0.15 [0.03; 0.30]†
Qatar	1.53 [1.40; 1.68]	1.32 [1.12; 1.55]	-0.21 [-0.47; 0.07]	1.5 [1.2; 1.8]	1.1 [0.9; 1.4]	0.99 [0.89; 1.08]	1.02 [0.88; 1.20]	0.04 [-0.15; 0.25]
Romania	1.57 [1.51; 1.64]	1.38 [1.27; 1.49]	-0.20 [-0.32; -0.07]†	1.9 [1.7; 2.1]	1.4 [1.3; 1.5]	0.94 [0.90; 0.98]*	1.03 [0.95; 1.11]	0.09 [0.00; 0.18]†
Russian Federation¶	1.91 [1.87; 1.96]	1.43 [1.35; 1.51]	-0.48 [-0.57; -0.39]†	2.1 [1.9; 2.2]	1.4 [1.3; 1.5]	0.77 [0.74; 0.79]*	1.01 [0.96; 1.07]	0.24 [0.18; 0.31]†
Rwanda	1.08 [0.99; 1.18]	1.11 [0.94; 1.31]	0.03 [-0.18; 0.28]	16.1 [10.1; 32.2]	14.5 [8.7; 29.7]	0.98 [0.90; 1.06]	0.96 [0.79; 1.16]	-0.02 [-0.21; 0.21]
Saint Kitts and Nevis	1.57 [1.44; 1.72]	1.62 [1.35; 1.93]	0.05 [-0.27; 0.40]	3.5 [2.7; 4.5]	2.1 [1.6; 2.8]	0.95 [0.85; 1.05]	0.93 [0.78; 1.11]	-0.02 [-0.21; 0.19]
Saint Lucia	1.53 [1.39; 1.67]	1.50 [1.26; 1.79]	-0.02 [-0.32; 0.31]	3.4 [2.4; 4.9]	2.3 [1.6; 3.2]	0.97 [0.84; 1.08]	0.98 [0.81; 1.18]	0.01 [-0.20; 0.26]
Samoa	1.34 [1.22; 1.53]	1.45 [1.20; 1.74]	0.10 [-0.24; 0.46]	2.9 [2.0; 4.3]	2.0 [1.4; 3.0]	1.00 [0.77; 1.15]	1.02 [0.85; 1.23]	0.02 [-0.22; 0.32]
San Marino	1.49 [1.36; 1.63]	1.28 [1.07; 1.54]	-0.21 [-0.48; 0.09]	0.6 [0.4; 0.7]	0.4 [0.3; 0.6]	1.01 [0.92; 1.10]	1.00 [0.83; 1.19]	-0.01 [-0.21; 0.21]
Sao Tome and Principe	1.03 [0.93; 1.13]	1.42 [1.19; 1.69]	0.39 [0.13; 0.69]†	3.9 [3.0; 5.0]	2.7 [2.1; 3.6]	0.99 [0.90; 1.10]	1.07 [0.90; 1.27]	0.07 [-0.14; 0.30]
Saudi Arabia	1.25 [1.13; 1.37]	1.40 [1.17; 1.66]	0.15 [-0.12; 0.45]	1.9 [1.5; 2.4]	1.4 [1.0; 1.8]	1.00 [0.86; 1.18]	1.00 [0.84; 1.18]	0.00 [-0.26; 0.25]
Senegal	1.03 [0.95; 1.11]	1.47 [1.27; 1.69]	0.44 [0.22; 0.69]†	9.5 [6.5; 13.6]	6.4 [4.3; 9.4]	1.00 [0.92; 1.08]	0.85 [0.69; 1.04]	-0.15 [-0.34; 0.06]
Serbia	1.44 [1.34; 1.56]	1.43 [1.25; 1.64]	-0.01 [-0.24; 0.22]	1.3 [1.2; 1.5]	0.9 [0.8; 1.1]	1.04 [0.97; 1.12]	0.94 [0.83; 1.07]	-0.10 [-0.25; 0.05]
Seychelles	1.52 [1.39; 1.67]	1.61 [1.36; 1.91]	0.09 [-0.22; 0.43]	2.8 [1.7; 4.5]	1.8 [1.1; 2.8]	0.98 [0.88; 1.08]	0.91 [0.76; 1.09]	-0.07 [-0.26; 0.15]
Sierra Leone	1.04 [0.94; 1.14]	0.93 [0.81; 1.07]	-0.11 [-0.27; 0.07]	24.2 [18.0; 34.7]	25.9 [19.2; 37.3]	0.99 [0.90; 1.11]	1.10 [0.94; 1.28]	0.10 [-0.09; 0.32]
Singapore	1.46 [1.34; 1.59]	1.22 [1.04; 1.43]	-0.24 [-0.48; 0.02]	0.7 [0.6; 0.8]	0.6 [0.5; 0.7]	1.02 [0.94; 1.11]	1.05 [0.89; 1.23]	0.03 [-0.17; 0.23]
Slovakia	1.48 [1.39; 1.58]	1.25 [1.11; 1.41]	-0.23 [-0.41; -0.04]†	1.3 [1.2; 1.5]	1.1 [0.9; 1.2]	1.00 [0.94; 1.07]	1.06 [0.95; 1.19]	0.06 [-0.08; 0.20]
Slovenia	1.47 [1.35; 1.59]	1.24 [1.05; 1.47]	-0.23 [-0.47; 0.04]	0.7 [0.6; 0.9]	0.6 [0.5; 0.7]	1.00 [0.93; 1.09]	1.03 [0.88; 1.22]	0.03 [-0.16; 0.24]
Solomon Islands	1.36 [1.23; 1.48]	1.48 [1.24; 1.76]	0.13 [-0.16; 0.45]	4.9 [3.8; 6.3]	3.3 [2.5; 4.3]	1.00 [0.85; 1.14]	1.02 [0.86; 1.21]	0.02 [-0.21; 0.27]
Somalia	1.01 [0.92; 1.12]	1.01 [0.84; 1.20]	-0.01 [-0.22; 0.23]	24.8 [19.0; 32.3]	24.6 [19.0; 32.0]	1.00 [0.92; 1.10]	1.01 [0.84; 1.21]	0.00 [-0.21; 0.24]
South Africa	1.33 [1.21; 1.47]	1.39 [1.25; 1.55]	0.06 [-0.14; 0.26]	6.4 [5.7; 7.4]	4.6 [4.1; 5.3]	0.95 [0.77; 1.10]	1.04 [0.90; 1.19]	0.09 [-0.12; 0.32]
South Sudan	1.05 [0.94; 1.16]	1.03 [0.86; 1.23]	-0.02 [-0.24; 0.22]	22.3 [17.0; 28.8]	21.7 [16.6; 28.2]	1.00 [0.91; 1.10]	1.00 [0.83; 1.20]	0.00 [-0.21; 0.23]
Spain	1.44 [1.38; 1.50]	1.23 [1.13; 1.34]	-0.21 [-0.33; -0.08]†	0.7 [0.7; 0.8]	0.6 [0.5; 0.7]	1.02 [0.98; 1.07]	1.04 [0.95; 1.14]	0.02 [-0.08; 0.13]
Sri Lanka	1.29 [1.20; 1.38]	1.23 [1.07; 1.41]	-0.06 [-0.25; 0.16]	1.7 [1.3; 2.1]	1.3 [1.0; 1.7]	1.12 [1.03; 1.21]*	1.13 [0.99; 1.29]	0.01 [-0.17; 0.20]
Saint Vincent and the Grenadines	1.49 [1.36; 1.64]	1.46 [1.20; 1.76]	-0.04 [-0.35; 0.31]	5.5 [3.8; 7.8]	3.8 [2.6; 5.5]	1.00 [0.90; 1.10]	0.96 [0.71; 1.21]	-0.04 [-0.31; 0.24]
State of Palestine	1.47 [1.33; 1.61]	1.48 [1.23; 1.78]	0.01 [-0.30; 0.36]	4.1 [2.6; 6.8]	2.8 [1.8; 4.5]	0.98 [0.83; 1.10]	1.00 [0.78; 1.21]	0.02 [-0.24; 0.28]
Sudan	1.00 [0.91; 1.10]	1.32 [1.09; 1.58]	0.32 [0.04; 0.60]†	8.6 [5.6; 14.8]	6.5 [4.4; 10.8]	1.03 [0.93; 1.15]	0.95 [0.72; 1.23]	-0.07 [-0.34; 0.23]
Suriname§	1.48 [1.36; 1.62]	0.84 [0.74; 0.94]	-0.65 [-0.82; -0.47]†	3.6 [3.0; 4.3]	4.3 [3.5; 5.2]	0.99 [0.88; 1.10]	1.79 [1.57; 2.03]*	0.80 [0.55; 1.07]†

¶: Sex ratio is outlying in 1990. §: Sex ratio is outlying in 2021. *: The ratio of estimated to expected female mortality is significantly different from one. †: Change is significantly different from zero.

Continued on next page

Table 18 – continued from previous page

	Sex ratio 10q5			Sex-specific 10q5 in 2021 (per 1,000)		Estimated/Expected female 10q5		
	1990	2021	Change 1990–2021	Male	Female	1990	2021	Change 1990–2021
Eswatini	1.15 [1.04; 1.26]	1.17 [0.95; 1.45]	0.02 [-0.23; 0.33]	12.0 [8.0; 26.9]	10.2 [6.4; 19.7]	0.97 [0.83; 1.18]	0.86 [0.65; 1.17]	-0.11 [-0.41; 0.25]
Sweden	1.38 [1.29; 1.48]	1.18 [1.06; 1.32]	-0.20 [-0.37; -0.02]†	0.7 [0.7; 0.8]	0.6 [0.6; 0.7]	1.00 [0.93; 1.07]	1.08 [0.97; 1.22]	0.09 [-0.05; 0.24]
Switzerland	1.50 [1.40; 1.61]	1.14 [1.01; 1.28]	-0.36 [-0.54; -0.18]†	0.7 [0.7; 0.8]	0.7 [0.6; 0.7]	0.97 [0.90; 1.03]	1.13 [1.00; 1.27]*	0.16 [0.01; 0.33]†
Syria	1.20 [1.10; 1.31]	1.35 [1.16; 1.55]	0.14 [-0.08; 0.38]	5.4 [3.7; 8.2]	4.0 [2.8; 5.9]	1.03 [0.90; 1.16]	1.04 [0.81; 1.21]	0.01 [-0.26; 0.23]
Tajikistan¶	1.53 [1.45; 1.61]	1.36 [1.25; 1.46]	-0.17 [-0.30; -0.04]†	2.4 [2.2; 2.5]	1.7 [1.6; 1.9]	0.84 [0.79; 0.90]*	1.07 [0.99; 1.15]	0.23 [0.13; 0.33]†
Tanzania	1.03 [0.94; 1.12]	1.12 [0.95; 1.33]	0.09 [-0.12; 0.33]	13.3 [9.2; 26.3]	11.8 [8.6; 21.4]	1.00 [0.91; 1.09]	0.96 [0.75; 1.25]	-0.03 [-0.28; 0.27]
Thailand	1.43 [1.32; 1.55]	1.64 [1.46; 1.85]	0.21 [-0.02; 0.45]	5.2 [4.8; 5.6]	3.2 [2.9; 3.5]	0.99 [0.91; 1.08]	0.91 [0.80; 1.03]	-0.08 [-0.23; 0.07]
Timor Leste	1.00 [0.91; 1.10]	1.22 [1.02; 1.45]	0.22 [-0.02; 0.48]	10.2 [7.9; 13.2]	8.4 [6.4; 10.9]	1.01 [0.92; 1.12]	1.03 [0.81; 1.30]	0.02 [-0.24; 0.31]
Togo	1.05 [0.96; 1.16]	1.07 [0.91; 1.26]	0.02 [-0.19; 0.24]	12.0 [8.5; 16.7]	11.2 [8.0; 15.6]	0.98 [0.89; 1.08]	1.07 [0.86; 1.35]	0.09 [-0.15; 0.40]
Tonga	1.52 [1.38; 1.66]	1.50 [1.26; 1.78]	-0.02 [-0.31; 0.31]	2.5 [1.9; 3.6]	1.7 [1.2; 2.5]	0.98 [0.86; 1.09]	0.96 [0.80; 1.16]	-0.02 [-0.22; 0.22]
Trinidad and Tobago	1.40 [1.30; 1.52]	1.32 [1.16; 1.50]	-0.08 [-0.29; 0.14]	2.5 [2.0; 3.0]	1.9 [1.5; 2.3]	1.08 [1.00; 1.17]*	1.12 [0.98; 1.27]	0.04 [-0.13; 0.22]
Tunisia	1.37 [1.25; 1.49]	1.43 [1.24; 1.64]	0.07 [-0.18; 0.32]	3.7 [3.1; 4.3]	2.6 [2.2; 3.0]	1.02 [0.91; 1.13]	1.05 [0.92; 1.21]	0.04 [-0.15; 0.24]
Turkey	1.21 [1.08; 1.35]	1.37 [1.27; 1.47]	0.16 [-0.01; 0.33]	2.0 [1.9; 2.2]	1.5 [1.4; 1.6]	0.98 [0.86; 1.11]	1.05 [0.98; 1.12]	0.07 [-0.08; 0.21]
Turkmenistan	1.32 [1.22; 1.42]	1.41 [1.24; 1.62]	0.09 [-0.12; 0.33]	4.3 [3.4; 5.3]	3.0 [2.4; 3.8]	1.04 [0.95; 1.13]	1.07 [0.93; 1.22]	0.03 [-0.14; 0.22]
Turks and Caicos Islands	1.57 [1.43; 1.72]	1.34 [1.12; 1.62]	-0.22 [-0.51; 0.09]	1.4 [1.1; 1.9]	1.1 [0.8; 1.4]	0.96 [0.88; 1.05]	1.02 [0.85; 1.20]	0.06 [-0.14; 0.27]
Tuvalu	1.23 [1.12; 1.35]	1.53 [1.29; 1.82]	0.31 [0.02; 0.63]†	5.5 [4.2; 7.0]	3.6 [2.7; 4.7]	1.00 [0.85; 1.17]	0.98 [0.81; 1.17]	-0.02 [-0.26; 0.22]
Uganda	1.02 [0.93; 1.11]	1.10 [0.94; 1.29]	0.08 [-0.11; 0.30]	13.5 [9.5; 19.6]	12.3 [8.7; 17.8]	1.00 [0.92; 1.10]	1.00 [0.83; 1.25]	0.00 [-0.21; 0.27]
Ukraine	1.72 [1.67; 1.78]	1.40 [1.32; 1.50]	-0.32 [-0.42; -0.21]†	1.9 [1.8; 2.0]	1.4 [1.3; 1.4]	0.88 [0.84; 0.91]*	1.01 [0.95; 1.07]	0.14 [0.07; 0.21]†
United Arab Emirates	1.52 [1.38; 1.66]	1.39 [1.16; 1.65]	-0.13 [-0.42; 0.19]	1.8 [1.4; 2.3]	1.3 [1.0; 1.7]	1.00 [0.91; 1.09]	1.00 [0.84; 1.19]	0.00 [-0.19; 0.22]
United Kingdom	1.45 [1.39; 1.51]	1.31 [1.22; 1.41]	-0.14 [-0.26; -0.02]†	0.8 [0.7; 0.9]	0.6 [0.6; 0.7]	0.99 [0.96; 1.03]	0.98 [0.91; 1.06]	-0.02 [-0.10; 0.08]
United States of America	1.51 [1.47; 1.54]	1.31 [1.23; 1.40]	-0.20 [-0.28; -0.10]†	1.5 [1.4; 1.6]	1.2 [1.1; 1.2]	1.00 [0.97; 1.02]	1.05 [0.99; 1.11]	0.05 [-0.02; 0.12]
Uruguay	1.55 [1.44; 1.67]	1.26 [1.11; 1.43]	-0.30 [-0.50; -0.08]†	1.6 [1.4; 1.8]	1.3 [1.1; 1.5]	0.98 [0.91; 1.05]	1.11 [0.98; 1.25]	0.13 [-0.02; 0.30]
Uzbekistan	1.40 [1.30; 1.51]	1.34 [1.22; 1.48]	-0.06 [-0.23; 0.11]	3.6 [3.5; 3.8]	2.7 [2.5; 2.9]	0.99 [0.91; 1.08]	1.13 [1.02; 1.24]*	0.14 [0.00; 0.28]
Vanuatu	1.38 [1.26; 1.51]	1.50 [1.25; 1.78]	0.12 [-0.18; 0.44]	5.8 [4.5; 7.5]	3.9 [3.0; 5.1]	0.99 [0.86; 1.13]	1.00 [0.82; 1.20]	0.00 [-0.22; 0.25]
Venezuela (Bolivarian Republic of)	1.50 [1.39; 1.62]	1.43 [1.25; 1.63]	-0.08 [-0.30; 0.17]	4.2 [3.5; 5.0]	2.9 [2.5; 3.5]	1.01 [0.93; 1.09]	1.06 [0.92; 1.21]	0.05 [-0.12; 0.23]
Vietnam	1.21 [1.09; 1.33]	1.75 [1.49; 2.05]	0.54 [0.24; 0.88]†	3.4 [2.8; 4.5]	2.0 [1.6; 2.6]	0.96 [0.83; 1.09]	0.86 [0.73; 1.01]	-0.10 [-0.30; 0.10]
Yemen	1.05 [0.96; 1.15]	1.34 [1.12; 1.62]	0.29 [0.02; 0.60]†	7.9 [4.7; 14.3]	5.9 [3.5; 10.4]	0.99 [0.90; 1.10]	0.95 [0.70; 1.18]	-0.04 [-0.31; 0.22]
Zambia	1.01 [0.93; 1.10]	1.24 [1.05; 1.46]	0.23 [0.02; 0.47]†	11.3 [7.8; 16.7]	9.1 [6.4; 13.1]	1.01 [0.92; 1.11]	0.92 [0.73; 1.18]	-0.09 [-0.30; 0.19]
Zimbabwe	1.09 [0.99; 1.20]	1.08 [0.92; 1.28]	-0.01 [-0.21; 0.22]	11.5 [7.3; 17.0]	10.7 [6.5; 15.9]	1.02 [0.90; 1.16]	1.06 [0.86; 1.34]	0.04 [-0.21; 0.35]

Table 19: **Estimates and 90% uncertainty intervals for sex ratios for 5q15 in 1990 and 2021, the change in sex ratios from 1990 to 2021, sex-specific 5q15 in 2021, and ratios of estimated to expected female 5q15 and their change from 1990 to 2021 for the world, UNICEF regions, and all countries.** ¶: Sex ratio is outlying in 1990. §: Sex ratio is outlying in 2021. *: The ratio of estimated to expected female mortality is significantly different from one. †: Change is significantly different from zero.

	Sex ratio 5q15			Sex-specific 5q15 in 2021 (per 1,000)		Estimated/Expected female 5q15		
	1990	2021	Change 1990–2021	Male	Female	1990	2021	Change 1990–2021
World¶	1.22 [1.15; 1.30]	1.55 [1.43; 1.66]	0.32 [0.18; 0.46]†	5.5 [5.2; 5.9]	3.5 [3.3; 3.9]	1.26 [1.15; 1.36]*	1.07 [0.94; 1.21]	-0.19 [-0.35; -0.02]†
South Asia¶§	0.81 [0.73; 0.90]	1.30 [1.07; 1.57]	0.49 [0.24; 0.77]†	4.4 [3.8; 5.0]	3.4 [2.9; 3.9]	2.25 [1.93; 2.60]*	1.68 [1.34; 2.08]*	-0.57 [-1.06; -0.06]†
Europe and Central Asia	2.23 [2.06; 2.39]	1.91 [1.82; 2.00]	-0.32 [-0.51; -0.13]†	2.4 [2.3; 2.4]	1.2 [1.2; 1.3]	0.94 [0.74; 1.02]	1.19 [1.12; 1.25]*	0.25 [0.15; 0.46]†
Middle East and North Africa	1.64 [1.45; 1.83]	2.43 [2.05; 2.81]	0.79 [0.35; 1.22]†	5.5 [5.0; 6.1]	2.3 [2.0; 2.6]	1.12 [0.89; 1.32]	1.00 [0.85; 1.18]	-0.12 [-0.38; 0.17]
Sub-Saharan Africa	0.97 [0.90; 1.05]	1.33 [1.19; 1.48]	0.36 [0.19; 0.53]†	11.0 [9.9; 12.4]	8.2 [7.4; 9.4]	0.94 [0.81; 1.07]	0.97 [0.78; 1.20]	0.03 [-0.19; 0.29]
Latin America and Caribbean§	2.09 [1.96; 2.21]	2.96 [2.68; 3.21]	0.87 [0.57; 1.15]†	6.7 [6.4; 7.0]	2.3 [2.1; 2.5]	0.87 [0.79; 0.96]*	0.61 [0.54; 0.70]*	-0.26 [-0.38; -0.14]†
East Asia and Pacific	1.74 [1.47; 2.06]	2.11 [1.71; 2.55]	0.36 [-0.17; 0.90]	3.2 [2.7; 3.8]	1.5 [1.2; 1.9]	1.13 [0.94; 1.37]	1.03 [0.84; 1.27]	-0.10 [-0.42; 0.21]
North America	2.73 [2.65; 2.80]	2.37 [2.02; 2.79]	-0.35 [-0.71; 0.07]	4.0 [3.7; 4.2]	1.7 [1.5; 1.9]	0.81 [0.78; 0.84]*	0.97 [0.82; 1.15]	0.16 [0.01; 0.34]†
Afghanistan§	0.84 [0.62; 1.13]	0.61 [0.40; 0.93]	-0.23 [-0.60; 0.18]	9.7 [5.8; 15.1]	15.9 [9.8; 23.9]	1.05 [0.63; 2.33]	3.03 [1.30; 5.32]*	1.98 [-0.22; 4.30]
Albania	2.40 [2.09; 2.75]	1.52 [1.18; 1.93]	-0.88 [-1.37; -0.35]†	1.9 [1.6; 2.2]	1.2 [1.0; 1.5]	0.95 [0.83; 1.10]	1.47 [1.16; 1.88]*	0.52 [0.17; 0.94]†
Algeria	1.79 [1.45; 2.19]	1.72 [1.49; 1.99]	-0.06 [-0.53; 0.37]	3.0 [2.8; 3.3]	1.8 [1.6; 2.0]	1.22 [0.98; 1.53]	1.35 [1.16; 1.56]*	0.13 [-0.23; 0.45]
Andorra	2.43 [1.79; 3.29]	3.06 [1.89; 4.99]	0.63 [-0.93; 2.65]	1.9 [1.4; 2.4]	0.6 [0.4; 0.9]	0.93 [0.68; 1.28]	0.73 [0.45; 1.17]	-0.20 [-0.66; 0.34]
Angola	0.70 [0.51; 0.97]	1.88 [1.20; 2.93]	1.18 [0.42; 2.26]†	15.8 [11.6; 20.9]	8.4 [5.7; 12.1]	1.00 [0.69; 1.78]	0.53 [0.27; 1.22]	-0.47 [-1.22; 0.25]
Anguilla	2.24 [1.65; 3.06]	2.30 [1.27; 4.23]	0.06 [-1.31; 2.08]	2.7 [2.0; 3.6]	1.2 [0.7; 1.8]	1.00 [0.72; 1.38]	1.00 [0.55; 1.77]	0.00 [-0.59; 0.84]
Antigua and Barbuda	2.03 [1.48; 2.75]	2.52 [1.45; 4.30]	0.49 [-0.85; 2.45]	3.6 [2.2; 5.4]	1.4 [0.8; 2.5]	0.96 [0.53; 1.42]	0.91 [0.53; 1.58]	-0.05 [-0.68; 0.75]
Argentina	1.98 [1.85; 2.11]	2.18 [1.89; 2.52]	0.21 [-0.12; 0.57]	3.5 [3.3; 3.8]	1.6 [1.5; 1.8]	1.16 [1.08; 1.24]*	1.06 [0.92; 1.24]	-0.09 [-0.26; 0.09]
Armenia	2.26 [1.95; 2.63]	2.86 [2.22; 3.66]	0.59 [-0.13; 1.46]	3.1 [2.5; 3.7]	1.1 [0.8; 1.4]	1.03 [0.88; 1.20]	0.81 [0.63; 1.04]	-0.22 [-0.46; 0.05]
Australia	2.45 [2.26; 2.66]	2.09 [1.76; 2.47]	-0.36 [-0.75; 0.07]	2.2 [2.0; 2.4]	1.1 [0.9; 1.2]	0.93 [0.86; 1.02]	1.09 [0.91; 1.29]	0.15 [-0.04; 0.37]
Austria	3.12 [2.78; 3.50]	1.98 [1.58; 2.47]	-1.14 [-1.68; -0.55]†	1.7 [1.5; 1.9]	0.9 [0.7; 1.0]	0.73 [0.64; 0.82]*	1.11 [0.90; 1.40]	0.39 [0.15; 0.68]†
Azerbaijan	2.39 [2.15; 2.65]	2.02 [1.76; 2.32]	-0.37 [-0.75; 0.01]	5.0 [4.6; 5.4]	2.5 [2.2; 2.8]	0.97 [0.87; 1.08]	1.13 [0.98; 1.30]	0.15 [-0.03; 0.36]
Bahamas	2.09 [1.61; 2.69]	2.39 [1.60; 3.64]	0.30 [-0.75; 1.67]	4.4 [3.3; 5.7]	1.9 [1.2; 2.7]	1.08 [0.83; 1.42]	0.96 [0.62; 1.44]	-0.12 [-0.61; 0.45]
Bahrain	2.07 [1.59; 2.71]	1.80 [1.40; 2.31]	-0.27 [-1.02; 0.43]	2.2 [1.7; 2.6]	1.2 [0.9; 1.5]	1.11 [0.85; 1.47]	1.26 [0.99; 1.60]	0.14 [-0.30; 0.58]
Bangladesh¶§	0.96 [0.80; 1.16]	1.20 [0.89; 1.61]	0.24 [-0.13; 0.68]	2.7 [1.7; 3.9]	2.3 [1.4; 3.3]	2.00 [1.50; 2.56]*	1.90 [1.42; 2.57]*	-0.10 [-0.84; 0.75]
Barbados	1.90 [1.46; 2.47]	2.24 [1.29; 3.82]	0.34 [-0.84; 2.01]	2.9 [1.8; 4.4]	1.3 [0.7; 2.2]	1.18 [0.89; 1.55]	1.03 [0.60; 1.78]	-0.15 [-0.75; 0.68]
Belarus	2.40 [2.19; 2.63]	2.10 [1.65; 2.69]	-0.30 [-0.81; 0.31]	2.1 [1.8; 2.4]	1.0 [0.8; 1.2]	0.93 [0.84; 1.03]	1.07 [0.84; 1.36]	0.15 [-0.10; 0.45]
Belgium	2.61 [2.34; 2.90]	1.66 [1.33; 2.08]	-0.95 [-1.39; -0.45]†	1.2 [1.1; 1.4]	0.7 [0.6; 0.9]	0.88 [0.79; 0.99]*	1.28 [1.02; 1.62]*	0.40 [0.11; 0.75]†
Belize	2.40 [1.80; 3.24]	2.15 [1.48; 3.14]	-0.25 [-1.33; 0.95]	5.3 [3.9; 7.0]	2.5 [1.7; 3.5]	0.96 [0.71; 1.29]	1.05 [0.70; 1.54]	0.08 [-0.40; 0.64]
Benin	0.96 [0.75; 1.23]	1.09 [0.74; 1.60]	0.13 [-0.33; 0.68]	11.4 [7.1; 17.0]	10.5 [6.5; 15.9]	1.29 [0.73; 2.29]	1.50 [0.64; 2.67]	0.21 [-1.14; 1.53]
Bhutan	0.93 [0.68; 1.26]	1.70 [1.01; 2.91]	0.77 [-0.03; 1.99]	6.5 [3.3; 10.5]	3.8 [1.9; 6.6]	1.08 [0.59; 2.42]	1.27 [0.68; 2.22]	0.19 [-1.31; 1.31]
Bolivia (Plurinational State of)	1.12 [0.88; 1.43]	1.71 [0.99; 2.90]	0.58 [-0.22; 1.80]	5.5 [2.7; 8.8]	3.2 [1.6; 5.6]	1.39 [0.82; 2.10]	1.31 [0.73; 2.31]	-0.09 [-1.03; 1.10]
Bosnia and Herzegovina	2.13 [1.83; 2.47]	2.42 [1.70; 3.44]	0.29 [-0.52; 1.35]	2.3 [1.7; 2.9]	0.9 [0.6; 1.3]	1.09 [0.94; 1.28]	0.94 [0.67; 1.33]	-0.15 [-0.49; 0.27]
Botswana	1.16 [0.87; 1.54]	2.00 [1.24; 3.23]	0.84 [-0.04; 2.12]	6.0 [3.4; 9.6]	3.0 [1.6; 5.1]	1.07 [0.56; 2.03]	1.10 [0.65; 1.83]	0.02 [-1.03; 0.94]
Brazil§	2.68 [2.29; 3.14]	4.10 [3.26; 5.18]	1.42 [0.45; 2.57]†	7.6 [7.0; 8.1]	1.8 [1.5; 2.2]	0.77 [0.65; 0.92]*	0.51 [0.40; 0.66]*	-0.26 [-0.45; -0.07]†
British Virgin Islands	2.24 [1.62; 3.06]	2.31 [1.26; 4.17]	0.07 [-1.29; 2.05]	4.4 [3.2; 5.9]	1.9 [1.2; 3.0]	0.99 [0.71; 1.39]	0.99 [0.54; 1.84]	0.00 [-0.62; 0.92]
Brunei	2.18 [1.63; 2.93]	1.66 [1.02; 2.74]	-0.52 [-1.52; 0.75]	1.3 [0.8; 1.8]	0.8 [0.5; 1.1]	0.95 [0.68; 1.31]	1.29 [0.80; 2.07]	0.34 [-0.29; 1.19]
Bulgaria	2.09 [1.90; 2.31]	2.10 [1.76; 2.52]	0.01 [-0.39; 0.46]	3.2 [2.9; 3.5]	1.5 [1.3; 1.8]	1.09 [0.99; 1.21]	1.11 [0.92; 1.33]	0.01 [-0.20; 0.25]
Burkina Faso	0.81 [0.65; 1.02]	1.58 [0.97; 2.58]	0.77 [0.09; 1.80]†	8.6 [4.8; 13.3]	5.4 [2.9; 9.0]	1.22 [0.81; 2.05]	1.25 [0.60; 2.21]	0.02 [-1.04; 1.12]
Burundi	0.67 [0.49; 0.92]	1.53 [1.01; 2.35]	0.86 [0.27; 1.69]†	9.8 [6.1; 14.2]	6.4 [3.8; 9.6]	1.02 [0.73; 1.77]	1.21 [0.58; 2.04]	0.20 [-0.78; 1.07]
Cambodia	1.05 [0.82; 1.34]	2.02 [1.20; 3.36]	0.97 [0.08; 2.32]†	4.5 [2.5; 7.4]	2.2 [1.1; 3.9]	0.80 [0.52; 1.43]	1.12 [0.66; 1.91]	0.32 [-0.47; 1.16]
Cameroon	1.01 [0.80; 1.29]	1.20 [0.80; 1.78]	0.19 [-0.32; 0.81]	12.9 [8.1; 19.7]	10.7 [6.7; 16.6]	1.27 [0.77; 2.12]	1.14 [0.49; 2.31]	-0.13 [-1.26; 1.18]
Canada	2.73 [2.53; 2.94]	1.77 [1.51; 2.08]	-0.96 [-1.29; -0.59]†	2.2 [2.1; 2.4]	1.2 [1.1; 1.4]	0.83 [0.77; 0.90]*	1.28 [1.09; 1.50]*	0.45 [0.24; 0.68]†

¶: Sex ratio is outlying in 1990. §: Sex ratio is outlying in 2021. *: The ratio of estimated to expected female mortality is significantly different from one. †: Change is significantly different from zero.

Continued on next page

Table 19 – continued from previous page

	Sex ratio 5q15			Sex-specific 5q15 in 2021 (per 1,000)		Estimated/Expected female 5q15		
	1990	2021	Change 1990–2021	Male	Female	1990	2021	Change 1990–2021
Cape Verde	2.09 [1.58; 2.75]	1.25 [0.95; 1.66]	-0.83 [-1.57; -0.18]†	2.0 [1.4; 2.7]	1.6 [1.1; 2.2]	1.09 [0.82; 1.45]	1.79 [1.36; 2.34]*	0.69 [0.13; 1.32]†
Central African Republic	0.84 [0.67; 1.07]	1.52 [0.88; 2.67]	0.68 [-0.02; 1.84]	14.4 [6.4; 27.1]	9.5 [4.1; 18.4]	1.11 [0.72; 2.00]	1.09 [0.37; 2.57]	-0.02 [-1.16; 1.46]
Chad	1.05 [0.85; 1.31]	1.20 [0.79; 1.84]	0.15 [-0.35; 0.81]	16.3 [9.6; 25.0]	13.5 [7.8; 21.0]	1.05 [0.73; 1.70]	0.82 [0.40; 2.05]	-0.23 [-1.04; 1.04]
Chile	2.36 [2.17; 2.57]	2.24 [1.82; 2.76]	-0.12 [-0.59; 0.44]	3.1 [2.8; 3.3]	1.4 [1.2; 1.6]	0.97 [0.89; 1.06]	1.04 [0.84; 1.28]	0.07 [-0.15; 0.32]
China	1.82 [1.39; 2.40]	2.23 [1.45; 3.47]	0.41 [-0.62; 1.78]	1.7 [1.1; 2.5]	0.8 [0.5; 1.2]	1.20 [0.89; 1.61]	0.99 [0.64; 1.51]	-0.21 [-0.77; 0.43]
Colombia¶	2.55 [1.99; 3.24]	3.28 [2.57; 4.19]	0.73 [-0.25; 1.79]	6.1 [5.7; 6.6]	1.9 [1.5; 2.3]	0.48 [0.32; 0.73]*	0.67 [0.52; 0.87]*	0.19 [-0.11; 0.45]
Comoros	1.15 [0.85; 1.55]	1.83 [1.06; 3.15]	0.68 [-0.25; 2.08]	4.6 [2.8; 6.9]	2.5 [1.4; 4.1]	1.15 [0.59; 2.13]	1.25 [0.71; 2.17]	0.10 [-1.08; 1.22]
Congo	0.87 [0.65; 1.17]	1.72 [1.04; 2.80]	0.85 [0.07; 1.99]†	5.8 [2.8; 10.4]	3.4 [1.5; 6.3]	0.82 [0.57; 1.90]	1.27 [0.71; 2.18]	0.45 [-0.75; 1.41]
Democratic Republic of the Congo	0.93 [0.69; 1.27]	1.04 [0.67; 1.62]	0.11 [-0.42; 0.76]	17.4 [9.9; 26.9]	16.7 [9.6; 25.7]	0.87 [0.54; 2.12]	0.87 [0.43; 2.39]	0.00 [-1.34; 1.54]
Cook Islands¶	2.83 [2.17; 3.71]	2.54 [1.43; 4.47]	-0.29 [-1.78; 1.81]	7.8 [3.9; 11.4]	3.1 [1.4; 5.1]	0.58 [0.31; 0.87]*	0.82 [0.42; 1.56]	0.24 [-0.28; 1.05]
Costa Rica	1.96 [1.64; 2.32]	2.66 [2.10; 3.41]	0.71 [0.02; 1.52]†	3.5 [3.1; 3.8]	1.3 [1.1; 1.6]	1.19 [1.00; 1.42]	0.87 [0.68; 1.11]	-0.31 [-0.62; -0.01]†
Cote d'Ivoire	1.20 [0.94; 1.52]	1.35 [0.82; 2.23]	0.15 [-0.50; 1.08]	11.8 [6.1; 20.0]	8.7 [4.4; 15.4]	1.03 [0.61; 1.80]	1.15 [0.41; 2.40]	0.12 [-1.03; 1.43]
Croatia	2.37 [2.08; 2.70]	2.89 [2.29; 3.63]	0.51 [-0.16; 1.32]	2.9 [2.6; 3.3]	1.0 [0.8; 1.2]	0.96 [0.84; 1.10]	0.81 [0.64; 1.02]	-0.16 [-0.37; 0.09]
Cuba¶	1.52 [1.41; 1.65]	1.65 [1.37; 1.99]	0.12 [-0.18; 0.48]	2.5 [2.2; 2.7]	1.5 [1.3; 1.7]	1.48 [1.35; 1.61]*	1.40 [1.16; 1.68]*	-0.08 [-0.36; 0.23]
Cyprus	2.49 [1.89; 3.29]	2.17 [1.35; 3.54]	-0.32 [-1.54; 1.23]	1.4 [1.1; 1.9]	0.7 [0.4; 1.0]	0.93 [0.70; 1.23]	1.00 [0.62; 1.58]	0.07 [-0.43; 0.72]
Czech Republic	2.18 [1.98; 2.41]	2.19 [1.81; 2.64]	0.00 [-0.44; 0.50]	1.8 [1.6; 1.9]	0.8 [0.7; 0.9]	1.06 [0.95; 1.18]	1.02 [0.84; 1.23]	-0.04 [-0.25; 0.20]
Denmark	2.22 [1.94; 2.54]	1.82 [1.46; 2.28]	-0.40 [-0.88; 0.14]	1.2 [1.0; 1.4]	0.7 [0.5; 0.8]	1.05 [0.91; 1.20]	1.17 [0.92; 1.49]	0.13 [-0.18; 0.47]
Djibouti	0.91 [0.66; 1.25]	1.50 [0.82; 2.73]	0.58 [-0.21; 1.84]	12.2 [8.3; 16.8]	8.2 [5.1; 12.3]	1.02 [0.59; 2.06]	1.00 [0.37; 2.40]	-0.02 [-1.10; 1.42]
Dominica	2.71 [2.03; 3.61]	2.97 [1.76; 5.02]	0.26 [-1.30; 2.40]	2.6 [1.3; 4.7]	0.9 [0.4; 1.8]	0.84 [0.63; 1.13]	0.76 [0.46; 1.27]	-0.08 [-0.51; 0.49]
Dominican Republic	2.07 [1.56; 2.73]	2.41 [1.41; 4.12]	0.34 [-0.91; 2.18]	5.6 [3.1; 9.0]	2.3 [1.2; 4.1]	0.97 [0.65; 1.37]	0.91 [0.51; 1.60]	-0.06 [-0.66; 0.74]
Ecuador	1.70 [1.57; 1.84]	1.96 [1.68; 2.28]	0.26 [-0.06; 0.60]	4.0 [3.7; 4.4]	2.1 [1.8; 2.3]	1.24 [1.13; 1.37]*	1.18 [1.01; 1.38]*	-0.06 [-0.27; 0.17]
Egypt¶	1.33 [1.13; 1.55]	2.27 [1.72; 2.98]	0.95 [0.34; 1.68]†	5.1 [4.5; 5.6]	2.2 [1.8; 2.7]	1.63 [1.38; 1.95]*	1.00 [0.76; 1.33]	-0.63 [-1.03; -0.21]†
El Salvador¶§	3.31 [2.95; 3.71]	4.80 [3.71; 6.21]	1.48 [0.33; 2.98]†	6.9 [6.1; 7.6]	1.4 [1.1; 1.8]	0.52 [0.43; 0.64]*	0.45 [0.34; 0.59]*	-0.07 [-0.23; 0.09]
Equatorial Guinea	0.86 [0.63; 1.18]	1.41 [0.78; 2.57]	0.55 [-0.18; 1.75]	12.6 [8.7; 17.3]	8.9 [5.4; 13.3]	1.00 [0.59; 2.00]	1.01 [0.37; 2.45]	0.00 [-1.06; 1.47]
Eritrea	0.80 [0.59; 1.11]	1.84 [1.01; 3.39]	1.04 [0.13; 2.59]†	10.3 [7.3; 13.9]	5.6 [3.4; 8.6]	1.01 [0.62; 1.95]	0.98 [0.40; 2.02]	-0.04 [-1.06; 1.04]
Estonia	2.62 [2.26; 3.04]	1.51 [1.08; 2.12]	-1.11 [-1.73; -0.39]†	2.0 [1.5; 2.4]	1.3 [1.0; 1.7]	0.80 [0.68; 0.94]*	1.49 [1.07; 2.05]*	0.69 [0.23; 1.27]†
Ethiopia¶	1.07 [0.87; 1.33]	1.89 [1.19; 2.98]	0.81 [0.04; 1.93]†	9.9 [5.6; 14.4]	5.2 [2.8; 8.3]	0.71 [0.56; 0.95]*	0.98 [0.46; 1.74]	0.26 [-0.32; 1.05]
Federated States of Micronesia	1.94 [1.42; 2.70]	2.18 [1.18; 4.00]	0.24 [-1.04; 2.11]	6.6 [4.7; 8.8]	3.0 [1.8; 4.8]	0.99 [0.64; 1.45]	1.00 [0.51; 1.91]	0.01 [-0.66; 0.99]
Fiji§	1.86 [1.37; 2.51]	1.35 [0.94; 1.94]	-0.51 [-1.30; 0.30]	4.6 [3.6; 5.8]	3.4 [2.5; 4.4]	1.08 [0.68; 1.56]	1.70 [1.16; 2.45]*	0.61 [-0.14; 1.48]
Finland	2.61 [2.29; 2.96]	2.15 [1.77; 2.62]	-0.45 [-0.99; 0.12]	2.5 [2.2; 2.7]	1.1 [1.0; 1.3]	0.87 [0.77; 1.00]*	1.07 [0.88; 1.30]	0.19 [-0.03; 0.45]
France	2.41 [2.27; 2.55]	2.15 [1.83; 2.52]	-0.26 [-0.60; 0.13]	1.4 [1.3; 1.5]	0.6 [0.6; 0.7]	0.96 [0.90; 1.03]	0.99 [0.84; 1.19]	0.03 [-0.14; 0.24]
Gabon	1.45 [1.08; 1.93]	1.58 [0.93; 2.73]	0.13 [-0.72; 1.36]	8.2 [3.3; 14.4]	5.2 [2.0; 9.7]	1.21 [0.56; 1.85]	1.25 [0.53; 2.34]	0.04 [-0.99; 1.39]
The Gambia	0.91 [0.67; 1.24]	1.92 [1.25; 2.97]	1.01 [0.23; 2.10]†	10.3 [7.6; 13.6]	5.4 [3.6; 7.6]	0.97 [0.58; 1.94]	0.93 [0.48; 1.61]	-0.04 [-0.99; 0.72]
Georgia	2.47 [2.02; 3.01]	2.23 [1.74; 2.86]	-0.23 [-0.97; 0.56]	4.1 [3.6; 4.6]	1.8 [1.5; 2.2]	0.93 [0.75; 1.14]	1.03 [0.80; 1.34]	0.11 [-0.21; 0.46]
Germany	2.46 [2.32; 2.61]	2.08 [1.89; 2.30]	-0.37 [-0.62; -0.12]†	1.5 [1.4; 1.5]	0.7 [0.7; 0.8]	0.94 [0.88; 1.01]	1.04 [0.92; 1.17]	0.10 [-0.04; 0.25]
Ghana	1.23 [0.93; 1.64]	1.57 [0.95; 2.57]	0.34 [-0.46; 1.42]	8.6 [4.8; 13.0]	5.5 [2.9; 8.8]	1.08 [0.50; 2.04]	1.26 [0.63; 2.27]	0.18 [-1.06; 1.42]
Greece	2.78 [2.51; 3.10]	2.08 [1.66; 2.62]	-0.70 [-1.23; -0.11]†	1.4 [1.2; 1.5]	0.7 [0.5; 0.8]	0.83 [0.74; 0.93]*	1.03 [0.82; 1.29]	0.20 [-0.03; 0.48]
Grenada	1.99 [1.49; 2.67]	3.37 [2.09; 5.44]	1.38 [-0.12; 3.55]	3.6 [2.2; 5.2]	1.1 [0.6; 1.8]	1.13 [0.83; 1.54]	0.68 [0.42; 1.10]	-0.45 [-0.94; 0.08]
Guatemala¶	1.44 [1.35; 1.53]	2.31 [1.78; 3.00]	0.87 [0.35; 1.57]†	8.5 [7.2; 9.8]	3.7 [2.9; 4.6]	1.37 [1.25; 1.49]*	0.87 [0.65; 1.17]	-0.50 [-0.74; -0.18]†
Guinea	0.90 [0.70; 1.15]	1.08 [0.68; 1.73]	0.18 [-0.31; 0.87]	13.4 [7.7; 20.7]	12.4 [7.0; 19.7]	1.02 [0.65; 1.89]	1.22 [0.49; 2.74]	0.20 [-1.00; 1.81]
Guinea-Bissau	0.78 [0.56; 1.07]	1.18 [0.73; 1.92]	0.40 [-0.16; 1.18]	11.4 [8.0; 15.6]	9.7 [6.6; 13.6]	1.01 [0.63; 1.94]	1.38 [0.58; 2.72]	0.37 [-0.77; 1.71]
Guyana¶	1.63 [1.39; 1.92]	1.64 [1.12; 2.40]	0.01 [-0.60; 0.82]	6.8 [4.7; 9.2]	4.1 [2.7; 5.9]	1.33 [1.11; 1.59]*	1.32 [0.85; 2.01]	-0.02 [-0.56; 0.71]
Haiti	0.81 [0.62; 1.06]	1.39 [0.87; 2.19]	0.58 [-0.02; 1.41]	7.4 [4.0; 12.2]	5.3 [2.8; 9.0]	1.21 [0.70; 2.47]	1.49 [0.81; 2.56]	0.28 [-1.17; 1.50]
Honduras	1.88 [1.36; 2.56]	2.30 [1.27; 4.13]	0.41 [-0.89; 2.38]	5.6 [4.0; 7.4]	2.4 [1.5; 3.8]	0.97 [0.57; 1.45]	0.97 [0.52; 1.80]	0.00 [-0.68; 0.93]
Hungary	2.36 [2.14; 2.59]	2.30 [1.82; 2.88]	-0.06 [-0.59; 0.57]	1.8 [1.6; 2.0]	0.8 [0.6; 0.9]	0.97 [0.88; 1.07]	0.97 [0.78; 1.22]	0.00 [-0.23; 0.27]
Iceland	2.34 [1.76; 3.11]	3.31 [2.17; 5.04]	0.97 [-0.43; 2.81]	1.9 [1.4; 2.6]	0.6 [0.4; 0.9]	0.98 [0.73; 1.31]	0.68 [0.45; 1.02]	-0.30 [-0.70; 0.13]

¶: Sex ratio is outlying in 1990. §: Sex ratio is outlying in 2021. *: The ratio of estimated to expected female mortality is significantly different from one. †: Change is significantly different from zero.

Continued on next page

Table 19 – continued from previous page

	Sex ratio 5q15			Sex-specific 5q15 in 2021 (per 1,000)		Estimated/Expected female 5q15		
	1990	2021	Change 1990–2021	Male	Female	1990	2021	Change 1990–2021
India¶§	0.75 [0.66; 0.86]	1.25 [0.98; 1.62]	0.50 [0.20; 0.88]†	3.9 [3.3; 4.4]	3.1 [2.6; 3.6]	2.55 [2.12; 3.04]*	1.84 [1.42; 2.38]*	-0.70 [-1.34; -0.01]†
Indonesia¶	1.53 [1.20; 1.94]	2.08 [1.30; 3.37]	0.56 [-0.37; 1.96]	6.1 [3.6; 9.0]	2.9 [1.6; 4.7]	1.36 [1.02; 1.80]*	1.05 [0.62; 1.74]	-0.31 [-0.97; 0.50]
Iran (Islamic Republic of)	1.41 [1.03; 1.89]	2.30 [1.66; 3.20]	0.89 [0.05; 1.88]†	6.2 [5.3; 7.2]	2.7 [2.1; 3.5]	1.00 [0.61; 1.48]	0.96 [0.68; 1.36]	-0.05 [-0.61; 0.53]
Iraq	2.11 [1.54; 2.89]	2.17 [1.22; 3.92]	0.06 [-1.22; 1.95]	5.2 [3.8; 7.0]	2.4 [1.5; 3.7]	1.00 [0.70; 1.41]	1.04 [0.56; 1.89]	0.04 [-0.62; 0.97]
Ireland	2.45 [2.15; 2.80]	1.76 [1.41; 2.19]	-0.70 [-1.18; -0.18]†	1.1 [0.9; 1.2]	0.6 [0.5; 0.7]	0.95 [0.83; 1.08]	1.21 [0.96; 1.54]	0.27 [-0.03; 0.62]
Israel	2.47 [2.17; 2.81]	2.46 [2.06; 2.96]	-0.01 [-0.53; 0.57]	1.6 [1.4; 1.7]	0.6 [0.5; 0.7]	0.94 [0.83; 1.08]	0.88 [0.74; 1.07]	-0.06 [-0.26; 0.16]
Italy	3.15 [2.96; 3.35]	2.14 [1.81; 2.54]	-1.01 [-1.40; -0.57]†	1.1 [1.0; 1.2]	0.5 [0.4; 0.6]	0.73 [0.68; 0.78]*	0.99 [0.82; 1.21]	0.26 [0.08; 0.49]†
Jamaica	2.14 [1.58; 2.94]	2.49 [1.42; 4.35]	0.34 [-1.06; 2.30]	4.9 [3.6; 6.5]	2.0 [1.2; 3.1]	0.99 [0.69; 1.38]	0.92 [0.51; 1.63]	-0.07 [-0.65; 0.72]
Japan	2.67 [2.55; 2.80]	1.65 [1.52; 1.79]	-1.02 [-1.21; -0.84]†	1.4 [1.3; 1.4]	0.8 [0.8; 0.9]	0.87 [0.82; 0.92]*	1.30 [1.16; 1.48]*	0.43 [0.28; 0.62]†
Jordan	2.32 [1.74; 3.10]	2.15 [1.27; 3.67]	-0.17 [-1.35; 1.54]	4.2 [3.1; 5.6]	2.0 [1.2; 3.0]	0.95 [0.69; 1.28]	1.07 [0.62; 1.82]	0.12 [-0.47; 0.92]
Kazakhstan	1.91 [1.78; 2.04]	1.83 [1.65; 2.02]	-0.08 [-0.30; 0.15]	3.8 [3.6; 4.0]	2.1 [1.9; 2.2]	1.12 [1.04; 1.22]*	1.27 [1.14; 1.41]*	0.15 [-0.01; 0.31]
Kenya¶	1.17 [0.92; 1.49]	1.63 [1.00; 2.62]	0.46 [-0.27; 1.52]	7.4 [4.2; 11.5]	4.5 [2.5; 7.5]	1.63 [1.06; 2.23]*	1.28 [0.70; 2.20]	-0.35 [-1.23; 0.76]
Kiribati	1.55 [1.14; 2.13]	1.95 [1.05; 3.58]	0.40 [-0.68; 2.08]	9.2 [6.5; 12.4]	4.7 [2.9; 7.3]	0.99 [0.52; 1.61]	0.99 [0.46; 2.01]	0.00 [-0.79; 1.08]
Democratic People's Republic of Korea	1.97 [1.44; 2.71]	1.98 [1.11; 3.45]	0.01 [-1.16; 1.59]	4.9 [3.5; 6.5]	2.5 [1.5; 3.8]	1.01 [0.68; 1.44]	1.14 [0.64; 2.08]	0.14 [-0.53; 1.13]
Republic of Korea	2.41 [2.04; 2.84]	1.67 [1.33; 2.08]	-0.75 [-1.29; -0.18]†	1.4 [1.3; 1.5]	0.8 [0.7; 1.0]	0.94 [0.79; 1.12]	1.29 [1.03; 1.64]*	0.35 [0.03; 0.73]†
Kosovo	2.16 [1.59; 2.95]	2.31 [1.26; 4.25]	0.15 [-1.22; 2.18]	3.3 [2.4; 4.4]	1.4 [0.8; 2.3]	1.00 [0.70; 1.39]	1.00 [0.54; 1.82]	0.01 [-0.60; 0.89]
Kuwait	4.34 [3.48; 5.45]	3.31 [2.49; 4.45]	-1.02 [-2.45; 0.43]	2.9 [2.4; 3.5]	0.9 [0.7; 1.2]	0.83 [0.65; 1.03]	0.70 [0.52; 0.93]*	-0.13 [-0.40; 0.16]
Kyrgyzstan§	1.71 [1.53; 1.91]	1.33 [1.18; 1.51]	-0.37 [-0.63; -0.12]†	3.8 [3.5; 4.1]	2.9 [2.6; 3.1]	1.33 [1.18; 1.49]*	1.74 [1.52; 1.99]*	0.41 [0.14; 0.70]†
Lao People's Democratic Republic	1.00 [0.74; 1.34]	1.51 [0.90; 2.55]	0.52 [-0.21; 1.60]	5.4 [2.7; 10.1]	3.6 [1.7; 6.9]	1.19 [0.56; 2.65]	1.46 [0.80; 2.52]	0.26 [-1.43; 1.56]
Latvia¶	2.75 [2.42; 3.13]	2.30 [1.75; 3.07]	-0.45 [-1.13; 0.39]	3.0 [2.5; 3.5]	1.3 [1.0; 1.6]	0.73 [0.63; 0.85]*	1.01 [0.76; 1.33]	0.27 [-0.01; 0.60]
Lebanon	7.46 [5.47; 10.21]	2.33 [1.27; 4.18]	-5.12 [-8.12; -2.31]†	3.4 [2.4; 4.4]	1.4 [0.9; 2.3]	1.00 [0.71; 1.38]	0.99 [0.55; 1.82]	0.00 [-0.62; 0.88]
Lesotho	1.22 [0.92; 1.62]	1.17 [0.74; 1.88]	-0.05 [-0.65; 0.74]	10.6 [5.6; 17.8]	9.0 [4.7; 15.2]	0.97 [0.49; 1.92]	1.47 [0.56; 2.78]	0.51 [-0.88; 1.92]
Liberia	3.32 [2.50; 4.52]	1.27 [0.81; 1.98]	-2.06 [-3.36; -0.91]†	14.0 [8.2; 21.9]	11.1 [6.3; 17.6]	1.06 [0.57; 2.26]	0.95 [0.41; 2.24]	-0.11 [-1.51; 1.32]
Libya	2.17 [1.59; 2.99]	2.34 [1.28; 4.26]	0.17 [-1.20; 2.16]	3.8 [2.8; 5.0]	1.6 [1.0; 2.6]	1.00 [0.71; 1.41]	0.99 [0.54; 1.81]	-0.01 [-0.63; 0.86]
Lithuania	2.49 [2.22; 2.79]	2.37 [1.87; 3.03]	-0.12 [-0.71; 0.59]	2.9 [2.5; 3.4]	1.2 [1.0; 1.5]	0.86 [0.76; 0.98]*	0.98 [0.77; 1.24]	0.11 [-0.13; 0.39]
Luxembourg	2.39 [1.82; 3.12]	2.17 [1.37; 3.46]	-0.22 [-1.35; 1.20]	1.7 [1.2; 2.2]	0.8 [0.5; 1.1]	0.93 [0.70; 1.24]	1.01 [0.65; 1.58]	0.08 [-0.41; 0.70]
Macedonia	2.25 [1.84; 2.76]	1.63 [1.19; 2.24]	-0.62 [-1.31; 0.12]	1.8 [1.5; 2.1]	1.1 [0.8; 1.4]	1.03 [0.84; 1.27]	1.36 [1.00; 1.84]	0.32 [-0.11; 0.84]
Madagascar	0.97 [0.77; 1.21]	1.28 [0.77; 2.17]	0.31 [-0.28; 1.23]	11.5 [5.7; 18.6]	8.9 [4.4; 14.9]	1.24 [0.75; 2.13]	1.25 [0.44; 2.62]	0.01 [-1.27; 1.50]
Malawi	0.86 [0.71; 1.05]	1.61 [1.08; 2.40]	0.74 [0.16; 1.54]†	8.7 [5.8; 12.3]	5.4 [3.4; 8.1]	1.04 [0.72; 1.68]	1.24 [0.70; 1.98]	0.20 [-0.64; 1.00]
Malaysia	2.46 [2.14; 2.84]	2.59 [2.37; 2.82]	0.12 [-0.31; 0.51]	3.1 [3.0; 3.3]	1.2 [1.1; 1.3]	0.89 [0.76; 1.03]	0.90 [0.82; 0.99]*	0.01 [-0.15; 0.16]
Maldives¶	1.47 [1.15; 1.90]	2.01 [1.26; 3.20]	0.55 [-0.36; 1.79]	2.7 [2.0; 3.4]	1.3 [0.9; 1.9]	1.48 [1.12; 1.95]*	1.14 [0.72; 1.82]	-0.34 [-0.97; 0.45]
Mali	0.74 [0.59; 0.92]	1.27 [0.85; 1.90]	0.53 [0.06; 1.19]†	10.7 [6.7; 16.1]	8.4 [5.2; 13.0]	1.40 [0.89; 2.50]	1.37 [0.60; 2.37]	-0.03 [-1.38; 1.13]
Malta	2.58 [1.92; 3.47]	2.22 [1.35; 3.64]	-0.35 [-1.66; 1.28]	1.3 [0.9; 1.8]	0.6 [0.3; 0.9]	0.90 [0.67; 1.20]	0.97 [0.60; 1.58]	0.07 [-0.43; 0.73]
Marshall Islands	1.96 [1.43; 2.68]	2.13 [1.16; 3.95]	0.17 [-1.07; 2.07]	7.1 [5.1; 9.6]	3.4 [2.0; 5.3]	0.99 [0.65; 1.44]	0.99 [0.50; 1.93]	0.00 [-0.66; 1.00]
Mauritania	1.57 [1.16; 2.14]	1.39 [0.90; 2.14]	-0.18 [-0.93; 0.68]	7.8 [4.5; 12.1]	5.6 [3.1; 9.1]	1.06 [0.54; 1.66]	1.49 [0.81; 2.44]	0.43 [-0.49; 1.52]
Mauritius¶	1.46 [1.23; 1.74]	1.99 [1.53; 2.61]	0.53 [-0.01; 1.20]	4.1 [3.5; 4.8]	2.1 [1.7; 2.6]	1.58 [1.32; 1.89]*	1.16 [0.88; 1.52]	-0.42 [-0.85; 0.02]
Mexico	2.28 [2.19; 2.36]	2.54 [2.23; 2.89]	0.26 [-0.06; 0.63]	6.3 [6.0; 6.6]	2.5 [2.2; 2.7]	0.96 [0.92; 1.01]	0.87 [0.75; 1.00]*	-0.10 [-0.22; 0.04]
Republic of Moldova	2.04 [1.81; 2.31]	2.20 [1.72; 2.80]	0.15 [-0.41; 0.82]	4.0 [3.5; 4.5]	1.8 [1.5; 2.2]	1.08 [0.95; 1.23]	1.05 [0.82; 1.35]	-0.03 [-0.31; 0.30]
Monaco	2.32 [1.70; 3.15]	2.23 [1.20; 4.17]	-0.09 [-1.45; 1.94]	1.8 [1.3; 2.4]	0.8 [0.5; 1.3]	1.00 [0.74; 1.37]	0.99 [0.54; 1.79]	0.00 [-0.60; 0.83]
Mongolia	1.70 [1.37; 2.10]	2.01 [1.51; 2.66]	0.31 [-0.34; 1.05]	4.2 [3.6; 4.9]	2.1 [1.7; 2.6]	1.25 [0.98; 1.59]	1.14 [0.86; 1.53]	-0.10 [-0.56; 0.36]
Montenegro	2.04 [1.54; 2.68]	1.87 [1.19; 2.93]	-0.17 [-1.16; 1.03]	2.1 [1.5; 2.7]	1.1 [0.8; 1.6]	1.14 [0.87; 1.50]	1.21 [0.78; 1.86]	0.07 [-0.52; 0.81]
Montserrat	2.23 [1.63; 3.04]	2.34 [1.26; 4.30]	0.11 [-1.25; 2.15]	2.8 [2.0; 3.8]	1.2 [0.7; 1.9]	0.99 [0.71; 1.39]	0.99 [0.54; 1.78]	0.00 [-0.62; 0.84]
Morocco	1.69 [1.33; 2.15]	2.02 [1.19; 3.43]	0.33 [-0.66; 1.80]	2.8 [1.4; 4.5]	1.4 [0.7; 2.5]	1.28 [0.97; 1.66]	1.13 [0.67; 1.91]	-0.15 [-0.77; 0.70]
Mozambique	1.08 [0.85; 1.37]	1.65 [0.99; 2.75]	0.57 [-0.19; 1.69]	11.2 [5.1; 20.4]	6.8 [3.0; 12.9]	0.73 [0.49; 1.24]	0.98 [0.34; 2.05]	0.25 [-0.62; 1.35]
Myanmar	1.08 [0.78; 1.48]	2.34 [1.40; 3.92]	1.26 [0.21; 2.87]†	5.2 [2.9; 8.5]	2.2 [1.2; 3.9]	1.04 [0.53; 2.18]	1.17 [0.70; 1.98]	0.13 [-1.11; 1.13]
Namibia	1.41 [1.10; 1.79]	1.41 [0.85; 2.29]	0.00 [-0.70; 0.95]	8.3 [4.6; 13.1]	5.9 [3.2; 9.8]	0.93 [0.56; 1.53]	1.42 [0.69; 2.55]	0.49 [-0.48; 1.68]

¶: Sex ratio is outlying in 1990. §: Sex ratio is outlying in 2021. *: The ratio of estimated to expected female mortality is significantly different from one. †: Change is significantly different from zero.

Continued on next page

Table 19 – continued from previous page

	Sex ratio 5q15			Sex-specific 5q15 in 2021 (per 1,000)		Estimated/Expected female 5q15		
	1990	2021	Change 1990–2021	Male	Female	1990	2021	Change 1990–2021
Nauru	1.80 [1.30; 2.48]	2.15 [1.16; 3.91]	0.35 [-0.86; 2.20]	6.9 [4.9; 9.2]	3.2 [1.9; 5.0]	0.97 [0.54; 1.49]	1.00 [0.51; 1.94]	0.02 [-0.69; 1.03]
Nepal¶	0.91 [0.71; 1.15]	1.45 [0.90; 2.34]	0.55 [-0.08; 1.46]	5.1 [2.9; 8.1]	3.5 [1.9; 5.8]	2.05 [1.31; 2.85]*	1.55 [0.93; 2.54]	-0.51 [-1.54; 0.77]
Netherlands	2.02 [1.83; 2.23]	1.80 [1.54; 2.12]	-0.22 [-0.56; 0.15]	1.2 [1.1; 1.3]	0.7 [0.6; 0.8]	1.14 [1.03; 1.27]*	1.19 [0.98; 1.42]	0.04 [-0.20; 0.31]
New Zealand	2.50 [2.24; 2.80]	1.62 [1.37; 1.92]	-0.88 [-1.27; -0.48]†	2.4 [2.2; 2.7]	1.5 [1.3; 1.7]	0.86 [0.76; 0.97]*	1.41 [1.20; 1.68]*	0.56 [0.31; 0.84]†
Nicaragua	1.93 [1.42; 2.66]	2.28 [1.24; 4.21]	0.35 [-0.94; 2.32]	4.9 [3.6; 6.7]	2.2 [1.3; 3.5]	0.99 [0.56; 1.49]	1.00 [0.53; 1.87]	0.01 [-0.70; 0.96]
Niger	0.83 [0.66; 1.07]	1.27 [0.79; 2.05]	0.43 [-0.12; 1.25]	11.3 [5.9; 18.5]	8.9 [4.6; 15.1]	1.05 [0.67; 1.82]	1.29 [0.48; 2.54]	0.24 [-0.88; 1.56]
Nigeria	0.92 [0.69; 1.22]	1.26 [0.90; 1.78]	0.34 [-0.15; 0.93]	8.9 [5.9; 13.0]	7.0 [4.6; 10.4]	1.02 [0.60; 2.17]	1.54 [0.88; 2.36]	0.52 [-0.83; 1.46]
Niue	2.17 [1.58; 2.96]	2.20 [1.21; 4.02]	0.02 [-1.26; 1.95]	6.9 [4.8; 9.5]	3.2 [2.0; 4.8]	1.00 [0.72; 1.40]	0.99 [0.50; 1.88]	-0.01 [-0.65; 0.93]
Norway	2.79 [2.44; 3.19]	1.97 [1.60; 2.45]	-0.81 [-1.36; -0.22]†	1.7 [1.5; 1.9]	0.8 [0.7; 1.0]	0.83 [0.72; 0.95]*	1.11 [0.90; 1.38]	0.29 [0.03; 0.57]†
Oman	2.19 [1.60; 2.96]	2.31 [1.25; 4.30]	0.12 [-1.24; 2.21]	3.7 [2.7; 4.9]	1.6 [0.9; 2.5]	1.00 [0.72; 1.39]	1.00 [0.53; 1.85]	0.00 [-0.62; 0.89]
Pakistan	1.16 [0.93; 1.44]	2.19 [1.37; 3.50]	1.03 [0.12; 2.41]†	7.2 [4.3; 10.8]	3.3 [1.8; 5.5]	1.47 [0.95; 2.03]	0.96 [0.55; 1.61]	-0.51 [-1.24; 0.37]
Palau¶	2.82 [2.16; 3.66]	1.30 [0.97; 1.72]	-1.52 [-2.43; -0.75]†	8.2 [3.6; 17.2]	6.3 [2.8; 13.4]	0.68 [0.43; 0.95]*	1.51 [0.64; 2.21]	0.83 [-0.11; 1.59]
Panama	2.20 [1.80; 2.68]	2.35 [1.90; 2.94]	0.15 [-0.51; 0.87]	5.0 [4.5; 5.4]	2.1 [1.8; 2.5]	1.00 [0.81; 1.24]	0.97 [0.77; 1.21]	-0.03 [-0.35; 0.27]
Papua New Guinea§	1.60 [1.17; 2.18]	1.11 [0.68; 1.81]	-0.49 [-1.23; 0.34]	6.8 [4.8; 9.3]	6.1 [4.2; 8.7]	1.00 [0.55; 1.58]	1.94 [1.10; 3.29]*	0.94 [-0.08; 2.36]
Paraguay	1.87 [1.39; 2.50]	2.15 [1.19; 3.92]	0.28 [-0.92; 2.13]	5.8 [4.1; 7.7]	2.7 [1.6; 4.2]	1.05 [0.72; 1.49]	1.04 [0.55; 1.93]	-0.01 [-0.70; 0.95]
Peru	1.48 [1.24; 1.78]	1.82 [1.08; 3.02]	0.33 [-0.48; 1.57]	2.9 [1.4; 4.7]	1.6 [0.8; 2.8]	1.23 [0.90; 1.55]	1.26 [0.76; 2.09]	0.03 [-0.60; 0.92]
Philippines¶	1.61 [1.33; 1.97]	2.04 [1.55; 2.68]	0.43 [-0.17; 1.11]	5.0 [4.4; 5.5]	2.5 [2.0; 3.0]	1.35 [1.09; 1.67]*	1.12 [0.84; 1.48]	-0.23 [-0.64; 0.20]
Poland	2.90 [2.71; 3.09]	2.13 [1.92; 2.36]	-0.77 [-1.05; -0.46]†	2.5 [2.4; 2.6]	1.2 [1.1; 1.3]	0.79 [0.73; 0.84]*	1.08 [0.97; 1.21]	0.30 [0.17; 0.44]†
Portugal¶	3.51 [3.20; 3.84]	2.10 [1.75; 2.53]	-1.41 [-1.89; -0.87]†	1.5 [1.3; 1.7]	0.7 [0.6; 0.8]	0.61 [0.55; 0.67]*	1.03 [0.85; 1.25]	0.43 [0.23; 0.66]†
Qatar	2.35 [1.76; 3.13]	2.92 [1.99; 4.33]	0.57 [-0.68; 2.13]	2.8 [2.2; 3.3]	0.9 [0.7; 1.3]	0.97 [0.72; 1.29]	0.79 [0.53; 1.15]	-0.18 [-0.61; 0.27]
Romania	2.09 [1.94; 2.24]	2.34 [2.06; 2.64]	0.25 [-0.07; 0.59]	2.8 [2.6; 3.0]	1.2 [1.1; 1.3]	1.09 [1.01; 1.18]*	0.99 [0.87; 1.13]	-0.10 [-0.25; 0.06]
Russian Federation	2.56 [2.47; 2.64]	2.12 [1.85; 2.43]	-0.44 [-0.71; -0.12]†	3.9 [3.7; 4.1]	1.8 [1.7; 2.0]	0.81 [0.77; 0.85]*	1.09 [0.95; 1.26]	0.28 [0.13; 0.45]†
Rwanda¶	1.03 [0.82; 1.30]	1.84 [1.22; 2.79]	0.81 [0.11; 1.78]†	4.6 [2.7; 6.8]	2.5 [1.4; 3.9]	0.63 [0.49; 0.80]*	1.24 [0.80; 1.89]	0.61 [0.13; 1.29]†
Saint Kitts and Nevis	2.27 [1.68; 3.04]	2.63 [1.57; 4.50]	0.37 [-1.02; 2.32]	8.4 [5.0; 12.6]	3.2 [1.7; 5.4]	1.00 [0.74; 1.37]	0.76 [0.38; 1.37]	-0.24 [-0.80; 0.44]
Saint Lucia	2.07 [1.57; 2.77]	2.47 [1.51; 4.19]	0.40 [-0.83; 2.17]	5.5 [3.8; 7.5]	2.2 [1.3; 3.5]	1.08 [0.79; 1.44]	0.91 [0.52; 1.52]	-0.17 [-0.71; 0.52]
Samoa	1.69 [1.24; 2.30]	1.61 [1.00; 2.62]	-0.07 [-0.97; 1.04]	4.0 [2.1; 6.2]	2.5 [1.3; 4.0]	1.06 [0.39; 1.68]	1.42 [0.86; 2.29]	0.37 [-0.51; 1.50]
San Marino	2.27 [1.66; 3.10]	2.13 [1.14; 3.99]	-0.13 [-1.45; 1.83]	1.2 [0.9; 1.6]	0.6 [0.3; 0.9]	1.00 [0.72; 1.37]	1.00 [0.55; 1.84]	0.01 [-0.60; 0.90]
Sao Tome and Principe	1.47 [1.08; 1.99]	2.19 [1.29; 3.71]	0.73 [-0.35; 2.32]	7.9 [3.8; 13.4]	3.6 [1.6; 6.7]	1.14 [0.41; 1.92]	0.92 [0.43; 1.69]	-0.22 [-1.21; 0.93]
Saudi Arabia	1.86 [1.37; 2.54]	3.09 [1.91; 4.96]	1.23 [-0.18; 3.22]	6.4 [3.8; 10.0]	2.1 [1.1; 3.6]	1.02 [0.52; 1.53]	0.70 [0.41; 1.16]	-0.32 [-0.95; 0.38]
Senegal	1.05 [0.81; 1.36]	1.54 [1.00; 2.36]	0.49 [-0.16; 1.36]	7.6 [4.7; 10.8]	5.0 [2.9; 7.3]	1.48 [0.81; 2.28]	1.36 [0.80; 2.20]	-0.12 [-1.11; 0.99]
Serbia	2.31 [1.95; 2.75]	2.10 [1.64; 2.67]	-0.22 [-0.87; 0.49]	2.2 [2.0; 2.4]	1.1 [0.9; 1.3]	1.00 [0.83; 1.19]	1.08 [0.85; 1.39]	0.08 [-0.23; 0.44]
Seychelles	2.28 [1.70; 3.07]	3.42 [2.15; 5.37]	1.15 [-0.43; 3.29]	6.5 [4.0; 9.0]	1.9 [1.1; 3.1]	1.01 [0.75; 1.36]	0.63 [0.39; 1.04]	-0.38 [-0.83; 0.14]
Sierra Leone	0.70 [0.52; 0.96]	1.08 [0.75; 1.54]	0.37 [-0.05; 0.87]	16.8 [10.1; 24.2]	15.7 [9.3; 22.8]	1.06 [0.71; 2.85]	0.88 [0.47; 2.07]	-0.18 [-1.99; 1.03]
Singapore	2.12 [1.73; 2.58]	1.78 [1.29; 2.46]	-0.34 [-1.04; 0.46]	1.1 [0.9; 1.4]	0.6 [0.5; 0.8]	1.10 [0.90; 1.34]	1.20 [0.85; 1.69]	0.10 [-0.33; 0.63]
Slovakia	2.28 [2.03; 2.55]	1.95 [1.60; 2.40]	-0.33 [-0.77; 0.18]	2.3 [2.1; 2.5]	1.2 [1.0; 1.4]	1.02 [0.90; 1.15]	1.17 [0.95; 1.43]	0.15 [-0.10; 0.43]
Slovenia	2.47 [2.11; 2.90]	2.16 [1.49; 3.14]	-0.31 [-1.11; 0.74]	1.5 [1.3; 1.9]	0.7 [0.5; 1.0]	0.93 [0.78; 1.09]	1.01 [0.70; 1.44]	0.08 [-0.27; 0.54]
Solomon Islands	2.02 [1.46; 2.77]	1.82 [1.03; 3.19]	-0.20 [-1.32; 1.27]	5.3 [3.8; 7.1]	2.9 [1.8; 4.4]	1.00 [0.69; 1.45]	1.24 [0.69; 2.24]	0.24 [-0.49; 1.27]
Somalia	0.75 [0.55; 1.04]	0.94 [0.50; 1.74]	0.18 [-0.35; 1.02]	16.4 [10.5; 23.5]	17.4 [11.4; 24.9]	1.00 [0.64; 1.84]	1.01 [0.41; 3.38]	0.02 [-0.95; 2.35]
South Africa§	1.71 [1.32; 2.23]	1.42 [1.15; 1.78]	-0.29 [-0.88; 0.22]	8.7 [7.6; 9.9]	6.1 [5.1; 7.1]	1.07 [0.62; 1.50]	1.40 [1.08; 1.80]*	0.33 [-0.20; 0.92]
South Sudan	0.65 [0.47; 0.91]	1.00 [0.55; 1.87]	0.35 [-0.19; 1.23]	15.5 [10.1; 22.1]	15.5 [9.9; 22.3]	1.04 [0.73; 1.82]	1.01 [0.40; 3.17]	-0.03 [-0.95; 2.11]
Spain	2.62 [2.46; 2.79]	1.76 [1.48; 2.10]	-0.85 [-1.19; -0.48]†	1.0 [0.9; 1.1]	0.5 [0.5; 0.6]	0.87 [0.82; 0.94]*	1.21 [0.99; 1.48]	0.34 [0.10; 0.62]†
Sri Lanka	1.75 [1.49; 2.06]	2.04 [1.40; 2.99]	0.29 [-0.43; 1.30]	2.1 [1.6; 2.7]	1.0 [0.7; 1.4]	1.09 [0.89; 1.32]	1.10 [0.77; 1.59]	0.01 [-0.41; 0.54]
Saint Vincent and the Grenadines	2.25 [1.69; 3.03]	2.18 [1.29; 3.67]	-0.08 [-1.32; 1.59]	8.3 [5.4; 11.6]	3.8 [2.2; 6.0]	1.00 [0.74; 1.36]	0.92 [0.49; 1.66]	-0.08 [-0.67; 0.72]
State of Palestine	2.37 [1.72; 3.24]	2.42 [1.33; 4.42]	0.05 [-1.43; 2.17]	4.7 [3.4; 6.3]	2.0 [1.2; 3.1]	0.99 [0.70; 1.40]	1.00 [0.54; 1.83]	0.00 [-0.62; 0.91]
Sudan	0.89 [0.66; 1.20]	1.52 [0.83; 2.78]	0.62 [-0.15; 1.91]	12.4 [8.6; 17.0]	8.2 [5.0; 12.2]	0.95 [0.58; 1.78]	0.96 [0.36; 2.33]	0.00 [-0.92; 1.39]
Suriname¶§	1.56 [1.24; 1.95]	1.26 [0.99; 1.61]	-0.30 [-0.79; 0.18]	5.5 [4.4; 6.7]	4.4 [3.5; 5.4]	1.38 [1.07; 1.76]*	1.79 [1.37; 2.32]*	0.41 [-0.17; 1.02]

¶: Sex ratio is outlying in 1990. §: Sex ratio is outlying in 2021. *: The ratio of estimated to expected female mortality is significantly different from one. †: Change is significantly different from zero.

Continued on next page

Table 19 – continued from previous page

	Sex ratio 5q15			Sex-specific 5q15 in 2021 (per 1,000)		Estimated/Expected female 5q15		
	1990	2021	Change 1990–2021	Male	Female	1990	2021	Change 1990–2021
Eswatini	1.14<br>[0.84; 1.53]	1.05<br>[0.60; 1.85]	-0.08<br>[-0.71; 0.80]	9.7<br>[6.6; 13.8]	9.2<br>[6.1; 13.1]	1.27<br>[0.66; 2.16]	1.77<br>[0.73; 3.54]	0.50<br>[-0.82; 2.36]
Sweden	2.27<br>[2.02; 2.54]	2.19<br>[1.82; 2.63]	-0.08<br>[-0.54; 0.43]	1.6<br>[1.5; 1.7]	0.7<br>[0.6; 0.8]	1.02<br>[0.91; 1.16]	1.00<br>[0.83; 1.21]	-0.02<br>[-0.24; 0.21]
Switzerland	2.45<br>[2.18; 2.74]	2.09<br>[1.69; 2.58]	-0.35<br>[-0.86; 0.20]	1.5<br>[1.4; 1.7]	0.7<br>[0.6; 0.9]	0.94<br>[0.83; 1.06]	1.04<br>[0.84; 1.29]	0.10<br>[-0.13; 0.38]
Syria	1.54<br>[1.17; 2.03]	2.77<br>[1.83; 4.24]	1.23<br>[0.13; 2.77]†	5.3<br>[3.7; 7.0]	1.9<br>[1.2; 2.8]	1.19<br>[0.75; 1.70]	1.08<br>[0.70; 1.64]	-0.11<br>[-0.75; 0.62]
Tajikistan	1.65<br>[1.49; 1.84]	1.63<br>[1.41; 1.89]	-0.02<br>[-0.31; 0.28]	2.1<br>[2.0; 2.3]	1.3<br>[1.2; 1.5]	1.36<br>[1.22; 1.53]*	1.39<br>[1.19; 1.61]*	0.02<br>[-0.24; 0.30]
Tanzania	1.06<br>[0.85; 1.33]	1.23<br>[0.80; 1.91]	0.17<br>[-0.37; 0.89]	8.6<br>[5.6; 12.2]	7.0<br>[4.5; 10.3]	1.25<br>[0.76; 2.02]	1.62<br>[0.87; 2.70]	0.36<br>[-0.74; 1.59]
Thailand¶§	2.53<br>[2.08; 3.08]	3.29<br>[2.49; 4.35]	0.76<br>[-0.24; 1.91]	8.7<br>[7.9; 9.5]	2.7<br>[2.1; 3.3]	0.74<br>[0.58; 0.93]*	0.61<br>[0.44; 0.82]*	-0.14<br>[-0.39; 0.13]
Timor Leste	1.73<br>[1.29; 2.33]	0.67<br>[0.43; 1.04]	-1.05<br>[-1.74; -0.44]†	12.3<br>[7.2; 18.7]	18.3<br>[11.1; 26.8]	0.94<br>[0.42; 1.53]	2.23<br>[0.88; 4.63]	1.29<br>[-0.30; 3.78]
Togo	1.20<br>[0.93; 1.54]	1.56<br>[0.95; 2.57]	0.36<br>[-0.36; 1.42]	8.7<br>[4.8; 13.5]	5.6<br>[2.9; 9.1]	1.15<br>[0.65; 1.88]	1.26<br>[0.59; 2.24]	0.11<br>[-0.91; 1.24]
Tonga	2.46<br>[1.82; 3.31]	2.46<br>[1.50; 4.08]	0.00<br>[-1.31; 1.78]	4.2<br>[2.7; 6.1]	1.7<br>[1.0; 2.8]	0.94<br>[0.70; 1.27]	0.93<br>[0.56; 1.54]	-0.01<br>[-0.53; 0.64]
Trinidad and Tobago	1.86<br>[1.58; 2.21]	2.73<br>[2.04; 3.73]	0.87<br>[0.08; 1.88]†	7.2<br>[5.8; 8.6]	2.6<br>[1.9; 3.4]	1.22<br>[1.02; 1.44]*	0.78<br>[0.55; 1.07]	-0.44<br>[-0.75; -0.08]†
Tunisia	1.97<br>[1.52; 2.55]	2.31<br>[1.65; 3.22]	0.34<br>[-0.55; 1.37]	4.2<br>[3.4; 5.1]	1.8<br>[1.4; 2.4]	1.15<br>[0.88; 1.51]	1.00<br>[0.71; 1.40]	-0.16<br>[-0.62; 0.34]
Turkey	1.67<br>[1.23; 2.28]	2.21<br>[1.84; 2.68]	0.54<br>[-0.20; 1.20]	2.4<br>[2.2; 2.6]	1.1<br>[0.9; 1.2]	1.04<br>[0.48; 1.68]	1.03<br>[0.86; 1.24]	-0.01<br>[-0.66; 0.60]
Turkmenistan¶§	1.51<br>[1.28; 1.78]	1.18<br>[0.82; 1.70]	-0.33<br>[-0.79; 0.25]	4.7<br>[3.6; 6.1]	4.0<br>[3.0; 5.2]	1.46<br>[1.22; 1.75]*	1.93<br>[1.32; 2.81]*	0.48<br>[-0.22; 1.38]
Turks and Caicos Islands	2.42<br>[1.85; 3.17]	2.23<br>[1.20; 4.04]	-0.19<br>[-1.47; 1.71]	2.9<br>[2.1; 4.0]	1.3<br>[0.8; 2.0]	0.93<br>[0.70; 1.23]	1.04<br>[0.58; 1.89]	0.11<br>[-0.46; 0.98]
Tuvalu	1.87<br>[1.37; 2.55]	2.15<br>[1.17; 4.00]	0.28<br>[-0.94; 2.18]	5.9<br>[4.3; 7.9]	2.7<br>[1.6; 4.4]	1.01<br>[0.65; 1.49]	1.03<br>[0.53; 1.94]	0.02<br>[-0.67; 1.00]
Uganda	0.83<br>[0.68; 1.01]	1.32<br>[0.88; 1.97]	0.49<br>[0.01; 1.15]†	15.0<br>[9.6; 21.9]	11.4<br>[7.1; 17.0]	1.12<br>[0.76; 1.83]	0.82<br>[0.40; 1.92]	-0.29<br>[-1.12; 0.86]
Ukraine	2.19<br>[2.08; 2.31]	2.28<br>[2.07; 2.50]	0.08<br>[-0.15; 0.33]	3.5<br>[3.4; 3.7]	1.5<br>[1.4; 1.7]	1.01<br>[0.95; 1.07]	1.02<br>[0.92; 1.13]	0.01<br>[-0.10; 0.13]
United Arab Emirates	2.28<br>[1.67; 3.12]	2.33<br>[1.26; 4.29]	0.05<br>[-1.34; 2.13]	3.1<br>[2.2; 4.1]	1.3<br>[0.8; 2.1]	1.00<br>[0.73; 1.37]	1.00<br>[0.54; 1.81]	-0.01<br>[-0.62; 0.84]
United Kingdom	2.55<br>[2.39; 2.72]	1.80<br>[1.47; 2.19]	-0.76<br>[-1.12; -0.33]†	1.5<br>[1.4; 1.7]	0.9<br>[0.7; 1.0]	0.91<br>[0.85; 0.98]*	1.21<br>[0.99; 1.49]	0.30<br>[0.07; 0.59]†
United States of America	2.72<br>[2.65; 2.80]	2.41<br>[2.04; 2.86]	-0.31<br>[-0.69; 0.15]	4.1<br>[3.9; 4.4]	1.7<br>[1.5; 1.9]	0.81<br>[0.77; 0.84]*	0.95<br>[0.80; 1.14]	0.15<br>[-0.01; 0.34]
Uruguay	1.88<br>[1.65; 2.15]	2.78<br>[2.26; 3.46]	0.90<br>[0.31; 1.62]†	5.0<br>[4.4; 5.5]	1.8<br>[1.5; 2.1]	1.21<br>[1.05; 1.40]*	0.82<br>[0.65; 1.02]	-0.40<br>[-0.64; -0.15]†
Uzbekistan§	1.61<br>[1.36; 1.91]	1.14<br>[0.94; 1.39]	-0.47<br>[-0.83; -0.11]†	3.7<br>[3.3; 4.0]	3.2<br>[2.9; 3.6]	1.40<br>[1.17; 1.67]*	2.03<br>[1.66; 2.50]*	0.63<br>[0.16; 1.14]†
Vanuatu	2.06<br>[1.50; 2.83]	1.80<br>[1.03; 3.18]	-0.26<br>[-1.36; 1.22]	5.9<br>[4.2; 7.9]	3.3<br>[2.1; 5.0]	1.00<br>[0.69; 1.43]	1.23<br>[0.67; 2.22]	0.23<br>[-0.48; 1.27]
Venezuela (Bolivarian Republic of)§	2.51<br>[2.10; 2.99]	4.26<br>[3.00; 6.14]	1.75<br>[0.38; 3.67]†	17.9<br>[14.8; 21.2]	4.2<br>[2.9; 5.8]	0.84<br>[0.69; 1.02]	0.20<br>[0.13; 0.33]*	-0.64<br>[-0.84; -0.44]†
Vietnam	1.89<br>[1.41; 2.52]	2.78<br>[1.89; 4.14]	0.90<br>[-0.21; 2.36]	4.0<br>[3.0; 5.2]	1.4<br>[1.0; 2.1]	1.10<br>[0.77; 1.54]	0.83<br>[0.55; 1.23]	-0.27<br>[-0.80; 0.24]
Yemen	1.88<br>[1.37; 2.60]	3.07<br>[1.65; 5.66]	1.19<br>[-0.41; 3.87]	12.5<br>[9.0; 16.6]	4.1<br>[2.4; 6.5]	0.98<br>[0.59; 1.46]	0.99<br>[0.50; 1.95]	0.00<br>[-0.68; 1.04]
Zambia	0.79<br>[0.65; 0.96]	1.83<br>[1.24; 2.71]	1.04<br>[0.42; 1.94]†	11.1<br>[7.2; 16.5]	6.1<br>[3.8; 9.5]	1.20<br>[0.81; 1.96]	0.91<br>[0.41; 1.58]	-0.29<br>[-1.19; 0.49]
Zimbabwe¶	1.29<br>[1.03; 1.62]	1.44<br>[0.94; 2.18]	0.14<br>[-0.47; 0.96]	11.0<br>[7.2; 16.0]	7.6<br>[4.8; 11.6]	1.56<br>[1.17; 2.04]*	1.18<br>[0.52; 2.10]	-0.38<br>[-1.21; 0.65]

Table 20: **Estimates and 90% uncertainty intervals for sex ratios for 5q20 in 1990 and 2021, the change in sex ratios from 1990 to 2021, sex-specific 5q20 in 2021, and ratios of estimated to expected female 5q20 and their change from 1990 to 2021 for the world, UNICEF regions, and all countries.** ¶: Sex ratio is outlying in 1990. §: Sex ratio is outlying in 2021. *: The ratio of estimated to expected female mortality is significantly different from one. †: Change is significantly different from zero.

	Sex ratio 5q20			Sex-specific 5q20 in 2021 (per 1,000)		Estimated/Expected female 5q20		
	1990	2021	Change 1990–2021	Male	Female	1990	2021	Change 1990–2021
World¶	1.35 [1.24; 1.45]	1.73 [1.54; 1.89]	0.38 [0.15; 0.58]†	7.7 [7.3; 9.3]	4.4 [4.1; 5.5]	1.26 [1.10; 1.40]*	1.02 [0.81; 1.21]	-0.24 [-0.49; 0.01]
South Asia¶	0.83 [0.74; 0.92]	1.54 [1.20; 1.99]	0.72 [0.36; 1.17]†	6.6 [5.2; 9.0]	4.2 [3.3; 5.8]	2.70 [2.23; 3.18]*	1.59 [0.79; 2.09]	-1.12 [-2.01; -0.42]†
Europe and Central Asia	2.99 [2.78; 3.13]	2.45 [2.32; 2.58]	-0.54 [-0.74; -0.30]†	3.7 [3.5; 3.8]	1.5 [1.4; 1.6]	0.85 [0.61; 0.91]*	1.06 [1.00; 1.13]*	0.21 [0.13; 0.47]†
Middle East and North Africa	1.98 [1.69; 2.39]	3.00 [2.41; 3.56]	1.02 [0.25; 1.64]†	7.3 [6.3; 9.1]	2.4 [2.1; 3.2]	1.16 [0.52; 1.50]	1.05 [0.81; 1.29]	-0.10 [-0.53; 0.58]
Sub-Saharan Africa	1.13 [1.00; 1.24]	1.29 [1.10; 1.47]	0.15 [-0.07; 0.39]	14.4 [12.6; 19.5]	11.2 [9.8; 15.6]	0.88 [0.74; 1.06]	0.96 [0.71; 1.28]	0.08 [-0.21; 0.41]
Latin America and Caribbean¶§	2.54 [2.35; 2.74]	3.63 [3.18; 3.99]	1.08 [0.60; 1.50]†	11.0 [10.2; 12.2]	3.0 [2.8; 3.5]	0.72 [0.61; 0.89]*	0.57 [0.46; 0.72]*	-0.15 [-0.35; 0.03]
East Asia and Pacific	1.98 [1.64; 2.36]	2.43 [1.83; 3.07]	0.45 [-0.30; 1.21]	4.8 [3.4; 8.0]	2.0 [1.4; 3.4]	1.17 [0.89; 1.46]	1.06 [0.63; 1.40]	-0.11 [-0.61; 0.35]
North America	3.21 [3.14; 3.29]	2.66 [2.18; 3.23]	-0.55 [-1.04; 0.02]	7.7 [6.7; 8.8]	2.9 [2.4; 3.5]	0.83 [0.80; 0.87]*	1.01 [0.82; 1.23]	0.17 [-0.01; 0.40]
Afghanistan	0.82 [0.56; 1.21]	0.62 [0.37; 1.03]	-0.20 [-0.67; 0.30]	13.7 [3.2; 36.2]	22.0 [5.4; 57.4]	1.01 [0.63; 3.79]	3.38 [0.86; 6.75]	2.38 [-1.37; 5.56]
Albania	2.71 [2.35; 3.11]	1.91 [1.51; 2.42]	-0.80 [-1.38; -0.17]†	2.5 [1.9; 3.2]	1.3 [1.0; 1.8]	0.99 [0.86; 1.14]	1.32 [1.03; 1.69]*	0.33 [0.00; 0.73]
Algeria¶	1.56 [1.20; 2.02]	1.87 [1.57; 2.21]	0.31 [-0.23; 0.81]	3.9 [3.4; 4.4]	2.1 [1.8; 2.4]	1.72 [1.32; 2.24]*	1.42 [1.20; 1.68]*	-0.31 [-0.88; 0.18]
Andorra	2.71 [1.81; 3.98]	2.79 [1.36; 5.78]	0.08 [-1.91; 3.18]	2.7 [1.9; 3.5]	1.0 [0.5; 1.7]	0.99 [0.67; 1.48]	0.91 [0.45; 1.83]	-0.08 [-0.78; 0.91]
Angola	0.73 [0.49; 1.11]	1.66 [0.97; 2.83]	0.93 [0.11; 2.13]†	22.2 [15.7; 29.9]	13.4 [8.6; 19.8]	1.01 [0.65; 1.91]	0.59 [0.28; 2.03]	-0.42 [-1.30; 0.98]
Anguilla	2.66 [1.79; 3.93]	2.66 [1.27; 5.75]	0.00 [-1.94; 3.24]	4.2 [3.0; 5.7]	1.6 [0.8; 2.8]	1.00 [0.67; 1.49]	1.00 [0.47; 2.04]	0.01 [-0.75; 1.09]
Antigua and Barbuda	2.54 [1.71; 3.74]	2.82 [1.43; 5.59]	0.28 [-1.64; 3.15]	4.6 [1.3; 9.8]	1.6 [0.4; 3.9]	0.89 [0.25; 1.46]	0.93 [0.47; 1.83]	0.04 [-0.71; 1.17]
Argentina	2.10 [1.97; 2.22]	2.81 [2.37; 3.33]	0.72 [0.25; 1.26]†	5.9 [5.1; 6.7]	2.1 [1.8; 2.5]	1.28 [1.19; 1.37]*	0.96 [0.80; 1.14]	-0.32 [-0.50; -0.12]†
Armenia	2.42 [2.11; 2.79]	3.28 [2.55; 4.27]	0.85 [0.03; 1.88]†	4.7 [3.5; 5.9]	1.4 [1.0; 1.9]	1.11 [0.96; 1.29]	0.82 [0.63; 1.05]	-0.30 [-0.55; -0.02]†
Australia	2.97 [2.73; 3.22]	2.74 [2.26; 3.33]	-0.22 [-0.77; 0.40]	3.2 [2.8; 3.7]	1.2 [1.0; 1.4]	0.90 [0.83; 0.99]*	0.94 [0.77; 1.14]	0.04 [-0.15; 0.25]
Austria	3.47 [3.09; 3.89]	2.99 [2.32; 3.83]	-0.48 [-1.27; 0.45]	2.5 [2.0; 3.0]	0.8 [0.6; 1.1]	0.77 [0.68; 0.87]*	0.84 [0.65; 1.09]	0.07 [-0.15; 0.34]
Azerbaijan	2.10 [1.91; 2.32]	2.38 [2.07; 2.74]	0.28 [-0.10; 0.70]	5.9 [4.9; 7.0]	2.5 [2.0; 3.0]	1.29 [1.17; 1.43]*	1.13 [0.98; 1.31]	-0.16 [-0.36; 0.05]
Bahamas§	2.13 [1.61; 2.81]	4.10 [2.70; 6.27]	1.98 [0.39; 4.21]†	11.3 [8.0; 15.0]	2.8 [1.7; 4.2]	1.19 [0.88; 1.60]	0.62 [0.39; 0.96]*	-0.57 [-1.04; -0.10]†
Bahrain	2.29 [1.69; 3.12]	1.65 [1.24; 2.20]	-0.64 [-1.56; 0.18]	2.9 [2.0; 3.9]	1.8 [1.2; 2.5]	1.17 [0.86; 1.57]	1.55 [1.17; 2.06]*	0.39 [-0.18; 0.97]
Bangladesh¶§	0.76 [0.61; 0.94]	1.41 [0.99; 2.01]	0.65 [0.19; 1.26]†	6.6 [4.3; 8.7]	4.6 [3.0; 6.4]	3.25 [2.40; 4.19]*	1.90 [1.33; 2.73]*	-1.35 [-2.45; -0.14]†
Barbados	2.27 [1.68; 3.03]	1.74 [0.96; 3.16]	-0.53 [-1.67; 1.05]	4.6 [1.7; 10.0]	2.6 [0.9; 6.2]	1.18 [0.88; 1.59]	1.50 [0.82; 2.74]	0.32 [-0.51; 1.60]
Belarus¶	4.01 [3.66; 4.41]	3.41 [2.57; 4.55]	-0.60 [-1.54; 0.59]	3.5 [2.6; 4.6]	1.0 [0.7; 1.5]	0.63 [0.56; 0.70]*	0.76 [0.57; 1.01]	0.13 [-0.07; 0.38]
Belgium	2.80 [2.53; 3.10]	2.67 [2.11; 3.38]	-0.13 [-0.77; 0.63]	2.4 [2.0; 2.8]	0.9 [0.7; 1.1]	0.96 [0.86; 1.06]	0.94 [0.73; 1.21]	-0.02 [-0.25; 0.27]
Belize	2.58 [1.86; 3.61]	3.17 [2.08; 4.84]	0.59 [-0.95; 2.44]	11.5 [7.1; 16.8]	3.6 [2.1; 5.9]	1.03 [0.72; 1.44]	0.78 [0.46; 1.24]	-0.24 [-0.78; 0.31]
Benin	1.21 [0.90; 1.62]	1.46 [0.91; 2.30]	0.25 [-0.45; 1.16]	13.0 [3.3; 29.2]	9.0 [2.2; 20.9]	1.70 [0.60; 2.68]	1.51 [0.43; 2.75]	-0.19 [-1.70; 1.53]
Bhutan	1.50 [1.04; 2.19]	1.93 [1.01; 3.72]	0.42 [-0.80; 2.26]	8.8 [1.8; 23.8]	4.6 [0.9; 13.5]	1.10 [0.37; 2.31]	1.25 [0.38; 2.57]	0.14 [-1.38; 1.76]
Bolivia (Plurinational State of)	1.55 [1.18; 2.03]	2.32 [1.20; 4.54]	0.76 [-0.48; 3.09]	7.3 [1.2; 24.0]	3.1 [0.5; 10.8]	1.42 [0.58; 2.08]	1.05 [0.34; 2.16]	-0.37 [-1.37; 1.02]
Bosnia and Herzegovina	3.10 [2.68; 3.58]	3.70 [2.43; 5.68]	0.60 [-0.75; 2.60]	3.9 [2.2; 6.3]	1.1 [0.5; 1.9]	0.87 [0.75; 1.02]	0.71 [0.46; 1.07]	-0.16 [-0.45; 0.22]
Botswana	0.93 [0.65; 1.33]	2.11 [1.19; 3.84]	1.18 [0.14; 2.94]†	8.2 [2.1; 18.3]	3.9 [0.9; 9.3]	0.93 [0.56; 2.96]	1.19 [0.54; 2.20]	0.27 [-1.87; 1.30]
Brazil¶§	3.22 [2.71; 3.85]	4.51 [3.44; 5.94]	1.29 [0.03; 2.80]†	11.9 [10.6; 13.3]	2.6 [2.1; 3.4]	0.77 [0.63; 0.93]*	0.55 [0.41; 0.74]*	-0.22 [-0.43; 0.02]
British Virgin Islands	2.64 [1.78; 3.96]	2.68 [1.24; 5.75]	0.05 [-1.90; 3.18]	7.0 [4.9; 9.5]	2.6 [1.4; 4.5]	0.99 [0.65; 1.49]	1.00 [0.46; 2.18]	0.01 [-0.74; 1.19]
Brunei	3.08 [2.22; 4.31]	1.64 [1.01; 2.64]	-1.45 [-2.88; -0.07]†	2.5 [1.4; 3.9]	1.5 [0.8; 2.6]	0.84 [0.59; 1.19]	1.55 [0.97; 2.49]	0.70 [0.01; 1.70]†
Bulgaria	2.57 [2.32; 2.86]	2.49 [2.10; 2.96]	-0.08 [-0.57; 0.45]	4.8 [4.2; 5.4]	1.9 [1.6; 2.3]	1.04 [0.93; 1.16]	1.08 [0.91; 1.29]	0.04 [-0.17; 0.28]
Burkina Faso	0.82 [0.63; 1.06]	1.40 [0.77; 2.51]	0.58 [-0.12; 1.73]	12.6 [3.5; 33.2]	9.0 [2.4; 24.5]	2.09 [0.86; 3.63]	1.55 [0.40; 3.28]	-0.55 [-2.62; 1.69]
Burundi	0.77 [0.52; 1.16]	2.10 [1.27; 3.44]	1.33 [0.38; 2.70]†	12.0 [2.6; 31.5]	5.7 [1.1; 15.9]	0.99 [0.66; 3.46]	1.05 [0.29; 1.96]	0.06 [-2.50; 1.05]
Cambodia	1.57 [1.18; 2.09]	2.07 [1.14; 3.76]	0.50 [-0.61; 2.26]	6.2 [1.3; 16.6]	3.0 [0.6; 8.7]	0.58 [0.36; 1.75]	1.24 [0.60; 2.30]	0.66 [-0.71; 1.71]
Cameroon	1.24 [0.93; 1.65]	1.26 [0.79; 1.96]	0.02 [-0.62; 0.81]	19.4 [7.4; 35.3]	15.4 [5.8; 28.7]	1.09 [0.53; 2.35]	1.15 [0.42; 2.91]	0.06 [-1.50; 1.94]
Canada	3.08 [2.86; 3.33]	2.16 [1.81; 2.58]	-0.93 [-1.35; -0.45]†	4.1 [3.6; 4.6]	1.9 [1.6; 2.2]	0.87 [0.80; 0.95]*	1.23 [1.03; 1.47]*	0.36 [0.15; 0.61]†

¶: Sex ratio is outlying in 1990. §: Sex ratio is outlying in 2021. *: The ratio of estimated to expected female mortality is significantly different from one. †: Change is significantly different from zero.

Continued on next page

Table 20 – continued from previous page

	Sex ratio 5q20			Sex-specific 5q20 in 2021 (per 1,000)		Estimated/Expected female 5q20		
	1990	2021	Change 1990–2021	Male	Female	1990	2021	Change 1990–2021
Cape Verde	2.27 [1.66; 3.10]	2.93 [2.12; 4.02]	0.65 [-0.54; 1.91]	5.7 [3.6; 8.3]	1.9 [1.2; 3.0]	1.17 [0.84; 1.62]	0.92 [0.67; 1.26]	-0.25 [-0.77; 0.24]
Central African Republic	0.87 [0.68; 1.12]	1.02 [0.53; 2.06]	0.15 [-0.42; 1.22]	27.0 [6.2; 89.1]	26.6 [5.8; 89.0]	0.97 [0.66; 2.68]	1.51 [0.50; 5.90]	0.54 [-1.33; 4.84]
Chad	2.24 [1.73; 3.30]	1.36 [0.81; 2.30]	-0.88 [-2.09; 0.18]	21.8 [6.0; 51.6]	16.0 [4.3; 38.6]	2.24 [0.81; 3.36]	0.83 [0.34; 2.80]	-1.41 [-2.66; 1.14]
Chile	3.46 [3.17; 3.76]	2.77 [2.19; 3.48]	-0.69 [-1.34; 0.08]	5.0 [4.3; 5.7]	1.8 [1.5; 2.2]	0.78 [0.71; 0.85]*	0.98 [0.78; 1.23]	0.20 [-0.02; 0.46]
China	2.00 [1.43; 2.81]	2.64 [1.51; 4.54]	0.63 [-0.82; 2.64]	3.5 [1.4; 7.8]	1.3 [0.5; 3.1]	1.32 [0.94; 1.86]	0.99 [0.57; 1.72]	-0.33 [-1.04; 0.50]
Colombia¶§	2.59 [1.90; 3.51]	4.20 [3.19; 5.51]	1.61 [0.22; 3.12]†	10.5 [9.4; 11.8]	2.5 [2.0; 3.2]	0.35 [0.22; 0.78]*	0.61 [0.46; 0.82]*	0.27 [-0.17; 0.51]
Comoros	2.02 [1.38; 2.96]	1.80 [0.93; 3.40]	-0.21 [-1.58; 1.56]	5.3 [1.5; 11.2]	3.0 [0.8; 6.6]	0.97 [0.30; 1.75]	1.46 [0.76; 2.81]	0.49 [-0.64; 2.06]
Congo	0.77 [0.54; 1.10]	1.67 [0.90; 3.04]	0.90 [0.02; 2.28]†	13.3 [4.2; 32.0]	8.0 [2.4; 20.2]	1.09 [0.68; 4.02]	1.28 [0.33; 2.79]	0.19 [-3.03; 1.73]
Democratic Republic of the Congo	0.95 [0.65; 1.39]	0.98 [0.56; 1.71]	0.03 [-0.60; 0.83]	19.2 [4.4; 50.3]	19.5 [4.5; 52.3]	0.93 [0.53; 3.27]	1.43 [0.48; 4.15]	0.51 [-2.21; 3.18]
Cook Islands	2.58 [1.80; 3.72]	2.66 [1.23; 5.60]	0.07 [-1.81; 3.17]	5.0 [0.7; 23.8]	1.9 [0.3; 9.9]	0.88 [0.37; 1.38]	0.92 [0.29; 2.09]	0.04 [-0.77; 1.29]
Costa Rica	2.38 [1.97; 2.88]	3.19 [2.41; 4.19]	0.81 [-0.13; 1.89]	6.3 [5.2; 7.6]	2.0 [1.5; 2.6]	1.14 [0.94; 1.37]	0.84 [0.64; 1.12]	-0.29 [-0.60; 0.05]
Cote d'Ivoire	0.96 [0.74; 1.24]	1.21 [0.66; 2.24]	0.25 [-0.38; 1.31]	17.4 [4.5; 44.5]	14.4 [3.8; 38.4]	1.61 [0.71; 3.06]	1.34 [0.38; 3.59]	-0.27 [-2.21; 2.24]
Croatia¶	4.18 [3.65; 4.80]	3.10 [2.44; 3.93]	-1.08 [-1.99; -0.12]†	2.9 [2.3; 3.6]	1.0 [0.7; 1.3]	0.62 [0.54; 0.72]*	0.82 [0.65; 1.05]	0.20 [0.00; 0.45]†
Cuba¶	1.79 [1.66; 1.93]	2.12 [1.71; 2.61]	0.33 [-0.10; 0.84]	3.6 [3.1; 4.2]	1.7 [1.4; 2.1]	1.50 [1.38; 1.63]*	1.23 [1.01; 1.52]*	-0.27 [-0.53; 0.04]
Cyprus	2.79 [2.02; 3.82]	2.87 [1.74; 4.76]	0.08 [-1.56; 2.20]	2.8 [1.8; 3.9]	1.0 [0.5; 1.6]	0.97 [0.70; 1.33]	0.89 [0.54; 1.46]	-0.08 [-0.61; 0.56]
Czech Republic	3.09 [2.78; 3.44]	2.71 [2.28; 3.25]	-0.38 [-0.94; 0.24]	3.1 [2.7; 3.5]	1.1 [1.0; 1.4]	0.88 [0.78; 0.98]*	0.94 [0.78; 1.13]	0.07 [-0.12; 0.28]
Denmark	2.64 [2.31; 3.02]	2.41 [1.95; 2.96]	-0.23 [-0.82; 0.41]	2.1 [1.7; 2.4]	0.9 [0.7; 1.1]	1.02 [0.89; 1.17]	1.04 [0.83; 1.32]	0.02 [-0.23; 0.34]
Djibouti	0.90 [0.60; 1.35]	1.57 [0.75; 3.35]	0.67 [-0.30; 2.47]	18.6 [12.0; 26.2]	11.9 [6.6; 18.8]	1.01 [0.56; 3.12]	0.97 [0.26; 3.27]	-0.04 [-1.98; 2.19]
Dominica	2.53 [1.74; 3.70]	2.52 [1.26; 4.98]	0.00 [-1.80; 2.62]	6.6 [2.5; 13.6]	2.6 [0.9; 6.1]	1.05 [0.72; 1.52]	1.04 [0.50; 2.09]	-0.02 [-0.77; 1.10]
Dominican Republic	2.30 [1.65; 3.16]	2.58 [1.35; 4.87]	0.28 [-1.34; 2.75]	11.7 [3.5; 26.5]	4.5 [1.2; 11.6]	1.10 [0.67; 1.57]	0.88 [0.25; 1.85]	-0.22 [-1.05; 0.90]
Ecuador	2.12 [1.97; 2.29]	2.98 [2.49; 3.56]	0.85 [0.34; 1.45]†	8.2 [7.3; 9.1]	2.7 [2.3; 3.2]	1.19 [1.08; 1.31]*	0.90 [0.75; 1.08]	-0.29 [-0.48; -0.08]†
Egypt¶	1.40 [1.17; 1.67]	2.45 [1.77; 3.37]	1.05 [0.30; 2.01]†	5.6 [4.3; 7.1]	2.3 [1.6; 3.1]	1.92 [1.60; 2.31]*	1.10 [0.80; 1.53]	-0.82 [-1.31; -0.28]†
El Salvador¶§	6.22 [5.46; 7.11]	5.00 [3.76; 6.74]	-1.22 [-2.74; 0.66]	10.2 [8.3; 12.2]	2.0 [1.5; 2.8]	0.20 [0.16; 0.24]*	0.52 [0.38; 0.70]*	0.32 [0.17; 0.50]†
Equatorial Guinea	0.96 [0.65; 1.44]	1.80 [0.83; 3.82]	0.84 [-0.26; 2.86]	17.3 [11.5; 23.8]	9.6 [5.2; 16.1]	1.03 [0.54; 3.12]	0.98 [0.27; 2.95]	-0.05 [-2.13; 1.82]
Eritrea	0.81 [0.53; 1.20]	2.05 [0.93; 4.44]	1.24 [0.04; 3.62]†	16.6 [11.0; 22.7]	8.1 [4.3; 13.5]	1.01 [0.61; 2.96]	0.96 [0.24; 2.69]	-0.05 [-1.81; 1.65]
Estonia¶	3.57 [3.05; 4.19]	2.04 [1.40; 2.98]	-1.53 [-2.42; -0.47]†	2.5 [1.5; 3.5]	1.2 [0.7; 1.8]	0.69 [0.58; 0.82]*	1.24 [0.85; 1.79]	0.55 [0.14; 1.11]†
Ethiopia¶	1.43 [1.11; 1.84]	1.92 [1.12; 3.28]	0.49 [-0.43; 1.90]	11.2 [2.5; 27.2]	5.8 [1.3; 15.0]	0.62 [0.47; 0.84]*	1.20 [0.34; 2.27]	0.57 [-0.32; 1.65]
Federated States of Micronesia	2.49 [1.67; 3.68]	2.63 [1.23; 5.71]	0.14 [-1.76; 3.35]	8.9 [6.1; 11.9]	3.4 [1.7; 5.8]	0.99 [0.62; 1.53]	1.00 [0.45; 2.17]	0.01 [-0.77; 1.27]
Fiji§	2.02 [1.40; 2.94]	1.62 [1.07; 2.40]	-0.40 [-1.50; 0.62]	7.4 [5.2; 9.8]	4.6 [3.1; 6.5]	1.12 [0.36; 1.80]	1.66 [1.10; 2.52]*	0.53 [-0.35; 1.69]
Finland	3.29 [2.90; 3.73]	2.44 [2.02; 2.95]	-0.85 [-1.47; -0.21]†	4.1 [3.5; 4.7]	1.7 [1.4; 2.0]	0.81 [0.72; 0.93]*	1.09 [0.90; 1.31]	0.27 [0.06; 0.52]†
France	3.46 [3.27; 3.66]	2.70 [2.26; 3.23]	-0.75 [-1.24; -0.19]†	2.6 [2.3; 2.9]	1.0 [0.8; 1.1]	0.78 [0.73; 0.83]*	0.93 [0.77; 1.13]	0.15 [-0.02; 0.36]
Gabon	1.28 [0.94; 1.76]	1.77 [0.90; 3.45]	0.49 [-0.56; 2.24]	10.5 [1.7; 32.3]	5.9 [0.9; 20.1]	1.61 [0.53; 2.61]	1.22 [0.31; 2.77]	-0.38 [-1.88; 1.62]
The Gambia	0.97 [0.66; 1.44]	1.73 [1.08; 2.76]	0.76 [-0.08; 1.84]	13.1 [9.6; 17.5]	7.6 [5.1; 11.0]	1.09 [0.56; 3.21]	1.39 [0.73; 2.38]	0.30 [-1.88; 1.42]
Georgia	3.02 [2.40; 3.80]	3.03 [2.31; 3.98]	0.01 [-1.03; 1.16]	6.1 [5.0; 7.2]	2.0 [1.5; 2.6]	0.88 [0.70; 1.12]	0.89 [0.67; 1.17]	0.00 [-0.32; 0.34]
Germany	2.73 [2.59; 2.87]	2.39 [2.19; 2.61]	-0.34 [-0.58; -0.08]†	2.1 [1.9; 2.2]	0.9 [0.8; 0.9]	0.99 [0.94; 1.06]	1.05 [0.91; 1.22]	0.06 [-0.10; 0.24]
Ghana	1.09 [0.77; 1.55]	1.51 [0.84; 2.75]	0.42 [-0.45; 1.73]	8.9 [1.7; 25.0]	5.9 [1.1; 17.8]	1.39 [0.51; 3.10]	1.58 [0.47; 3.04]	0.19 [-1.95; 2.01]
Greece	3.42 [3.07; 3.80]	3.36 [2.64; 4.29]	-0.07 [-0.90; 0.93]	2.6 [2.2; 3.0]	0.8 [0.6; 1.0]	0.78 [0.70; 0.88]*	0.75 [0.58; 0.96]*	-0.03 [-0.23; 0.20]
Grenada	2.37 [1.65; 3.37]	2.09 [1.13; 3.93]	-0.28 [-1.73; 1.71]	4.5 [1.6; 8.5]	2.1 [0.7; 4.5]	1.09 [0.75; 1.60]	1.26 [0.66; 2.32]	0.16 [-0.64; 1.30]
Guatemala¶	1.85 [1.73; 1.97]	3.03 [2.19; 4.17]	1.19 [0.34; 2.32]†	12.7 [8.4; 18.3]	4.2 [2.6; 6.5]	1.14 [1.00; 1.28]*	0.79 [0.46; 1.16]	-0.36 [-0.70; 0.03]
Guinea	1.11 [0.83; 1.49]	1.08 [0.61; 1.92]	-0.03 [-0.66; 0.86]	19.0 [5.6; 43.9]	17.6 [5.1; 40.4]	1.77 [0.61; 2.92]	1.33 [0.43; 3.84]	-0.43 [-2.06; 2.56]
Guinea-Bissau	0.87 [0.58; 1.30]	1.34 [0.73; 2.49]	0.47 [-0.32; 1.68]	15.3 [10.2; 21.3]	11.4 [7.1; 17.0]	1.03 [0.57; 3.34]	1.62 [0.47; 3.51]	0.59 [-1.79; 2.45]
Guyana	2.12 [1.81; 2.48]	2.54 [1.60; 4.05]	0.42 [-0.58; 1.91]	16.0 [8.4; 28.5]	6.3 [3.1; 12.1]	1.18 [0.98; 1.40]	0.77 [0.24; 1.49]	-0.40 [-1.00; 0.34]
Haiti	0.73 [0.55; 0.98]	1.50 [0.88; 2.54]	0.77 [0.09; 1.82]†	13.2 [4.5; 27.6]	8.8 [2.9; 18.9]	1.74 [0.83; 4.24]	1.49 [0.41; 2.88]	-0.25 [-3.09; 1.45]
Honduras	2.22 [1.51; 3.28]	2.77 [1.31; 5.82]	0.55 [-1.36; 3.66]	8.7 [6.2; 11.7]	3.2 [1.7; 5.5]	0.95 [0.40; 1.58]	0.96 [0.44; 2.04]	0.01 [-0.82; 1.25]
Hungary	2.73 [2.48; 3.02]	2.23 [1.78; 2.82]	-0.50 [-1.04; 0.13]	2.8 [2.3; 3.3]	1.2 [1.0; 1.6]	0.98 [0.88; 1.09]	1.13 [0.89; 1.44]	0.15 [-0.11; 0.47]
Iceland	3.04 [2.16; 4.27]	2.23 [1.24; 4.02]	-0.80 [-2.47; 1.23]	1.9 [0.8; 3.1]	0.8 [0.3; 1.6]	0.89 [0.63; 1.24]	1.14 [0.63; 2.00]	0.25 [-0.39; 1.17]

¶: Sex ratio is outlying in 1990. §: Sex ratio is outlying in 2021. *: The ratio of estimated to expected female mortality is significantly different from one. †: Change is significantly different from zero.

Continued on next page

Table 20 – continued from previous page

	Sex ratio 5q20			Sex-specific 5q20 in 2021 (per 1,000)		Estimated/Expected female 5q20		
	1990	2021	Change 1990–2021	Male	Female	1990	2021	Change 1990–2021
India¶§	0.78 [0.68; 0.89]	1.54 [1.16; 2.06]	0.76 [0.36; 1.29]†	6.0 [4.6; 7.5]	3.9 [2.9; 5.1]	3.12 [2.56; 3.73]*	1.75 [1.30; 2.33]*	-1.37 [-2.12; -0.55]†
Indonesia¶	1.35 [1.02; 1.76]	2.65 [1.47; 4.68]	1.29 [0.01; 3.38]†	7.3 [1.6; 20.4]	2.8 [0.6; 8.5]	1.97 [1.51; 2.62]*	0.94 [0.36; 1.77]	-1.04 [-1.91; -0.05]†
Iran (Islamic Republic of)	1.77 [1.18; 2.63]	2.78 [1.86; 4.12]	1.01 [-0.29; 2.54]	7.6 [4.8; 11.3]	2.7 [1.6; 4.4]	1.00 [0.33; 1.68]	0.96 [0.64; 1.45]	-0.05 [-0.80; 0.77]
Iraq	2.64 [1.79; 3.91]	2.44 [1.16; 5.16]	-0.20 [-2.08; 2.66]	6.1 [4.3; 8.2]	2.5 [1.3; 4.3]	1.01 [0.67; 1.50]	1.10 [0.52; 2.31]	0.09 [-0.70; 1.37]
Ireland	3.17 [2.74; 3.68]	3.29 [2.59; 4.17]	0.12 [-0.73; 1.09]	2.0 [1.5; 2.4]	0.6 [0.4; 0.8]	0.86 [0.73; 0.99]*	0.76 [0.59; 0.99]*	-0.09 [-0.31; 0.16]
Israel	2.56 [2.24; 2.95]	3.03 [2.55; 3.63]	0.47 [-0.15; 1.13]	2.4 [2.1; 2.8]	0.8 [0.7; 1.0]	1.04 [0.91; 1.20]	0.82 [0.68; 1.01]	-0.22 [-0.43; 0.01]
Italy	3.41 [3.21; 3.63]	2.78 [2.28; 3.37]	-0.63 [-1.17; -0.01]†	1.7 [1.5; 1.9]	0.6 [0.5; 0.7]	0.79 [0.74; 0.85]*	0.91 [0.72; 1.14]	0.11 [-0.08; 0.35]
Jamaica	2.55 [1.72; 3.76]	3.17 [1.58; 6.23]	0.62 [-1.43; 3.80]	7.9 [5.7; 10.3]	2.5 [1.3; 4.2]	0.99 [0.64; 1.52]	0.84 [0.42; 1.71]	-0.15 [-0.83; 0.77]
Japan	2.53 [2.41; 2.66]	1.89 [1.77; 2.02]	-0.64 [-0.82; -0.47]†	2.4 [2.2; 2.5]	1.3 [1.2; 1.3]	1.04 [0.97; 1.11]	1.32 [1.18; 1.49]*	0.28 [0.14; 0.45]†
Jordan	2.53 [1.74; 3.71]	2.57 [1.31; 4.96]	0.04 [-1.75; 2.59]	5.2 [3.7; 6.9]	2.0 [1.1; 3.4]	1.06 [0.72; 1.54]	1.05 [0.54; 2.03]	-0.01 [-0.73; 1.04]
Kazakhstan	2.28 [2.14; 2.44]	2.21 [2.01; 2.43]	-0.07 [-0.33; 0.20]	6.0 [5.5; 6.4]	2.7 [2.5; 3.0]	1.10 [1.01; 1.19]*	1.22 [1.10; 1.35]*	0.12 [-0.03; 0.28]
Kenya	1.15 [0.87; 1.52]	1.54 [0.89; 2.64]	0.39 [-0.39; 1.55]	11.8 [3.4; 28.1]	7.7 [2.2; 18.7]	1.88 [0.73; 2.82]	1.48 [0.40; 2.89]	-0.40 [-1.88; 1.41]
Kiribati	2.11 [1.42; 3.13]	2.48 [1.15; 5.23]	0.37 [-1.33; 3.25]	12.3 [8.5; 16.8]	5.0 [2.7; 8.5]	0.97 [0.39; 1.65]	0.99 [0.40; 2.29]	0.02 [-0.89; 1.41]
Democratic People's Republic of Korea	2.51 [1.68; 3.74]	2.30 [1.14; 4.61]	-0.21 [-1.90; 2.33]	6.7 [4.7; 9.0]	2.9 [1.6; 4.8]	1.00 [0.64; 1.53]	1.16 [0.58; 2.38]	0.17 [-0.68; 1.43]
Republic of Korea	2.26 [1.87; 2.74]	1.50 [1.18; 1.93]	-0.75 [-1.35; -0.18]†	2.1 [1.9; 2.4]	1.4 [1.2; 1.7]	1.18 [0.98; 1.44]	1.67 [1.27; 2.20]*	0.48 [0.00; 1.05]†
Kosovo	2.52 [1.70; 3.75]	2.70 [1.27; 5.80]	0.18 [-1.75; 3.40]	5.1 [3.6; 6.9]	1.9 [1.0; 3.3]	1.00 [0.65; 1.53]	1.00 [0.46; 2.10]	0.00 [-0.76; 1.14]
Kuwait	6.95 [5.53; 8.86]	2.47 [1.83; 3.32]	-4.48 [-6.45; -2.80]†	4.1 [3.0; 5.3]	1.7 [1.1; 2.3]	0.96 [0.76; 1.21]	1.08 [0.80; 1.45]	0.12 [-0.25; 0.54]
Kyrgyzstan¶	2.02 [1.82; 2.25]	1.85 [1.62; 2.11]	-0.17 [-0.50; 0.16]	5.3 [4.7; 5.9]	2.8 [2.5; 3.3]	1.29 [1.15; 1.46]*	1.46 [1.28; 1.68]*	0.17 [-0.08; 0.43]
Lao People's Democratic Republic	1.81 [1.24; 2.60]	1.33 [0.70; 2.51]	-0.47 [-1.53; 0.87]	8.2 [1.8; 20.7]	6.2 [1.3; 16.1]	0.87 [0.31; 1.89]	1.88 [0.66; 3.74]	1.01 [-0.68; 2.96]
Latvia¶	3.71 [3.24; 4.25]	2.51 [1.93; 3.28]	-1.19 [-1.99; -0.30]†	4.9 [3.9; 6.0]	1.9 [1.4; 2.5]	0.66 [0.56; 0.77]*	1.07 [0.82; 1.39]	0.41 [0.14; 0.74]†
Lebanon	16.02 [11.46; 22.85]	2.69 [1.25; 5.67]	-13.33 [-20.44; -7.73]†	4.3 [3.0; 5.7]	1.6 [0.8; 2.8]	0.99 [0.69; 1.43]	0.99 [0.48; 2.06]	0.00 [-0.70; 1.12]
Lesotho	1.20 [0.84; 1.69]	1.64 [0.96; 2.85]	0.45 [-0.43; 1.70]	21.1 [6.5; 45.5]	12.8 [4.0; 29.0]	1.23 [0.46; 2.80]	0.74 [0.28; 2.31]	-0.49 [-2.15; 1.32]
Liberia	5.66 [4.02; 8.52]	1.13 [0.70; 1.81]	-4.52 [-7.43; -2.64]†	16.4 [5.1; 33.4]	14.5 [4.6; 29.6]	0.90 [0.50; 2.89]	1.68 [0.48; 3.46]	0.78 [-1.82; 2.62]
Libya	2.70 [1.81; 4.03]	2.72 [1.28; 5.91]	0.03 [-1.98; 3.33]	4.7 [3.3; 6.2]	1.7 [0.9; 3.0]	0.99 [0.66; 1.49]	0.98 [0.46; 2.05]	-0.01 [-0.75; 1.12]
Lithuania¶	4.18 [3.68; 4.76]	2.99 [2.37; 3.78]	-1.20 [-2.05; -0.26]†	4.7 [3.9; 5.7]	1.6 [1.2; 2.0]	0.60 [0.52; 0.69]*	0.90 [0.71; 1.14]	0.30 [0.09; 0.55]†
Luxembourg	2.76 [2.09; 3.69]	2.46 [1.41; 4.24]	-0.30 [-1.75; 1.64]	1.4 [0.5; 2.6]	0.6 [0.2; 1.2]	0.97 [0.72; 1.28]	1.02 [0.59; 1.79]	0.05 [-0.51; 0.86]
Macedonia	2.00 [1.60; 2.47]	2.31 [1.62; 3.28]	0.31 [-0.54; 1.36]	2.3 [1.6; 3.2]	1.0 [0.7; 1.5]	1.33 [1.08; 1.64]*	1.09 [0.77; 1.57]	-0.24 [-0.70; 0.31]
Madagascar	1.05 [0.81; 1.36]	1.33 [0.70; 2.50]	0.28 [-0.45; 1.51]	13.5 [2.6; 43.1]	10.1 [1.9; 34.0]	1.67 [0.68; 2.86]	1.47 [0.37; 3.48]	-0.19 [-1.98; 2.11]
Malawi	0.92 [0.73; 1.15]	1.68 [1.05; 2.70]	0.76 [0.08; 1.81]†	14.0 [5.0; 27.2]	8.3 [2.8; 17.0]	1.13 [0.68; 2.93]	1.29 [0.37; 2.38]	0.16 [-1.95; 1.30]
Malaysia	2.56 [2.16; 3.03]	2.34 [2.17; 2.51]	-0.22 [-0.72; 0.21]	4.7 [4.3; 5.0]	2.0 [1.8; 2.2]	1.02 [0.85; 1.22]	1.15 [1.06; 1.25]*	0.13 [-0.09; 0.32]
Maldives¶	1.19 [0.90; 1.57]	1.85 [1.09; 3.11]	0.66 [-0.22; 1.95]	1.8 [0.6; 3.2]	1.0 [0.3; 1.9]	2.24 [1.68; 2.98]*	1.36 [0.82; 2.29]	-0.88 [-1.82; 0.23]
Mali	0.93 [0.71; 1.22]	1.23 [0.77; 1.93]	0.29 [-0.26; 1.05]	12.9 [4.3; 26.2]	10.6 [3.5; 21.7]	1.91 [0.73; 3.29]	1.86 [0.54; 3.30]	-0.05 [-2.07; 1.93]
Malta	2.68 [1.87; 3.81]	3.07 [1.80; 5.26]	0.39 [-1.38; 2.75]	1.8 [0.8; 3.0]	0.6 [0.2; 1.1]	0.98 [0.69; 1.39]	0.82 [0.48; 1.39]	-0.16 [-0.70; 0.50]
Marshall Islands	2.49 [1.67; 3.74]	2.61 [1.22; 5.59]	0.12 [-1.79; 3.22]	9.6 [6.8; 12.9]	3.7 [1.9; 6.4]	0.99 [0.61; 1.54]	1.00 [0.45; 2.18]	0.01 [-0.79; 1.26]
Mauritania	1.93 [1.32; 2.83]	1.24 [0.80; 1.96]	-0.69 [-1.69; 0.27]	8.5 [2.1; 19.8]	6.9 [1.6; 15.8]	1.09 [0.35; 1.85]	2.03 [0.87; 3.29]	0.94 [-0.42; 2.46]
Mauritius¶	1.40 [1.16; 1.68]	2.74 [2.07; 3.64]	1.34 [0.60; 2.28]†	7.1 [5.7; 8.7]	2.6 [1.9; 3.4]	1.93 [1.60; 2.33]*	0.98 [0.73; 1.31]	-0.95 [-1.42; -0.49]†
Mexico§	2.73 [2.64; 2.82]	3.30 [2.81; 3.88]	0.57 [0.08; 1.16]†	11.8 [10.5; 13.3]	3.6 [3.0; 4.2]	0.95 [0.90; 1.00]	0.76 [0.64; 0.91]*	-0.19 [-0.32; -0.04]†
Republic of Moldova¶	3.34 [2.95; 3.77]	2.80 [2.16; 3.63]	-0.54 [-1.30; 0.37]	5.9 [4.8; 7.1]	2.1 [1.6; 2.7]	0.74 [0.65; 0.85]*	0.96 [0.74; 1.25]	0.22 [-0.03; 0.52]
Monaco	2.72 [1.82; 4.02]	2.53 [1.16; 5.40]	-0.18 [-2.15; 2.79]	2.8 [1.9; 3.7]	1.1 [0.6; 1.9]	0.99 [0.67; 1.48]	1.00 [0.48; 2.16]	0.01 [-0.72; 1.20]
Mongolia	1.84 [1.43; 2.37]	2.34 [1.68; 3.24]	0.50 [-0.36; 1.50]	5.5 [4.1; 7.2]	2.4 [1.6; 3.3]	1.30 [0.95; 1.74]	1.15 [0.83; 1.60]	-0.15 [-0.71; 0.43]
Montenegro	2.16 [1.67; 2.80]	2.83 [1.70; 4.67]	0.66 [-0.67; 2.60]	3.3 [2.1; 4.7]	1.2 [0.7; 1.9]	1.25 [0.96; 1.62]	0.92 [0.56; 1.50]	-0.33 [-0.86; 0.33]
Montserrat	2.65 [1.79; 3.93]	2.66 [1.24; 5.72]	0.01 [-1.90; 3.22]	4.1 [2.9; 5.4]	1.5 [0.8; 2.7]	0.99 [0.66; 1.49]	1.00 [0.47; 2.06]	0.01 [-0.75; 1.12]
Morocco¶	1.83 [1.37; 2.48]	2.47 [1.27; 4.72]	0.63 [-0.78; 2.98]	4.3 [0.9; 12.2]	1.7 [0.4; 5.3]	1.43 [1.03; 1.95]*	1.05 [0.53; 2.05]	-0.38 [-1.15; 0.72]
Mozambique	1.06 [0.79; 1.44]	0.99 [0.54; 1.79]	-0.07 [-0.67; 0.79]	21.1 [6.3; 52.4]	21.3 [6.4; 53.5]	1.16 [0.56; 2.90]	1.26 [0.44; 4.27]	0.10 [-1.97; 3.09]
Myanmar	1.88 [1.27; 2.81]	2.05 [1.19; 3.75]	0.17 [-1.19; 2.00]	9.8 [3.3; 21.4]	4.8 [1.3; 11.3]	0.97 [0.31; 1.86]	1.76 [0.90; 3.16]	0.79 [-0.53; 2.41]
Namibia	1.41 [1.08; 1.84]	1.70 [0.96; 3.01]	0.29 [-0.59; 1.66]	17.9 [5.9; 41.6]	10.5 [3.3; 25.7]	1.01 [0.48; 2.06]	0.96 [0.28; 2.48]	-0.05 [-1.40; 1.59]

¶: Sex ratio is outlying in 1990. §: Sex ratio is outlying in 2021. *: The ratio of estimated to expected female mortality is significantly different from one. †: Change is significantly different from zero.

Continued on next page

Table 20 – continued from previous page

	**Sex ratio 5q20**			**Sex-specific 5q20 in 2021 (per 1,000)**		**Estimated/Expected female 5q20**		
	**1990**	**2021**	**Change 1990–2021**	**Male**	**Female**	**1990**	**2021**	**Change 1990–2021**
Nauru	2.35 [1.57; 3.45]	2.64 [1.21; 5.55]	0.30 [-1.53; 3.33]	9.4 [6.5; 12.6]	3.6 [1.9; 6.2]	0.98 [0.53; 1.57]	0.99 [0.46; 2.21]	0.01 [-0.82; 1.29]
Nepal¶	1.23 [0.93; 1.62]	1.41 [0.78; 2.51]	0.18 [-0.60; 1.32]	6.5 [1.7; 15.1]	4.6 [1.2; 10.9]	2.03 [1.34; 2.80]*	1.85 [0.95; 3.33]	-0.18 [-1.41; 1.49]
Netherlands	2.26 [2.04; 2.49]	2.02 [1.75; 2.33]	-0.24 [-0.60; 0.14]	1.9 [1.7; 2.1]	0.9 [0.8; 1.1]	1.16 [1.04; 1.29]*	1.25 [1.03; 1.50]*	0.09 [-0.15; 0.36]
New Zealand	3.40 [3.00; 3.85]	2.05 [1.72; 2.44]	-1.35 [-1.90; -0.78]†	3.6 [3.1; 4.2]	1.8 [1.5; 2.1]	0.77 [0.67; 0.88]*	1.28 [1.07; 1.52]*	0.51 [0.27; 0.77]†
Nicaragua	2.44 [1.66; 3.62]	2.62 [1.27; 5.68]	0.18 [-1.64; 3.34]	7.7 [5.4; 10.4]	2.9 [1.5; 5.0]	0.97 [0.38; 1.68]	0.99 [0.47; 2.12]	0.02 [-0.86; 1.31]
Niger	1.01 [0.76; 1.34]	0.92 [0.52; 1.67]	-0.09 [-0.63; 0.71]	15.9 [4.2; 40.9]	17.3 [4.5; 44.2]	1.13 [0.59; 2.94]	2.01 [0.52; 4.75]	0.88 [-1.70; 3.58]
Nigeria§	1.06 [0.75; 1.51]	1.10 [0.72; 1.65]	0.04 [-0.57; 0.67]	10.7 [4.3; 19.5]	9.7 [3.9; 18.0]	1.01 [0.52; 2.96]	2.27 [1.02; 3.62]*	1.26 [-1.12; 2.69]
Niue	2.64 [1.79; 3.91]	2.68 [1.24; 5.78]	0.04 [-1.93; 3.21]	11.4 [6.7; 17.1]	4.2 [2.6; 6.3]	1.00 [0.67; 1.50]	0.99 [0.41; 2.18]	-0.01 [-0.79; 1.22]
Norway	3.31 [2.85; 3.83]	2.43 [1.96; 3.03]	-0.87 [-1.57; -0.13]†	2.6 [2.2; 3.0]	1.1 [0.8; 1.3]	0.82 [0.70; 0.95]*	1.03 [0.82; 1.30]	0.22 [-0.04; 0.50]
Oman	2.67 [1.79; 3.98]	2.69 [1.25; 5.79]	0.03 [-2.00; 3.23]	4.3 [3.1; 5.7]	1.6 [0.8; 3.0]	1.00 [0.67; 1.50]	0.99 [0.47; 2.10]	-0.01 [-0.76; 1.16]
Pakistan¶	1.29 [1.02; 1.65]	3.16 [1.76; 5.69]	1.87 [0.37; 4.46]†	8.9 [2.0; 25.2]	2.8 [0.6; 8.7]	1.90 [1.33; 2.53]*	0.75 [0.24; 1.44]	-1.14 [-1.97; -0.19]†
Palau	2.57 [1.80; 3.63]	0.89 [0.64; 1.24]	-1.68 [-2.78; -0.82]†	19.9 [7.5; 38.3]	22.5 [8.5; 42.7]	0.89 [0.42; 1.37]	1.52 [0.64; 3.66]	0.63 [-0.44; 2.84]
Panama	2.62 [2.09; 3.31]	3.05 [2.38; 3.90]	0.43 [-0.54; 1.43]	8.9 [8.0; 9.9]	2.9 [2.4; 3.6]	1.00 [0.78; 1.27]	0.87 [0.67; 1.12]	-0.13 [-0.47; 0.20]
Papua New Guinea	2.12 [1.44; 3.16]	1.46 [0.86; 2.51]	-0.66 [-1.87; 0.64]	9.6 [6.8; 12.9]	6.5 [4.3; 9.5]	1.01 [0.46; 1.66]	1.78 [0.99; 3.12]	0.77 [-0.28; 2.24]
Paraguay	2.20 [1.52; 3.18]	2.61 [1.26; 5.54]	0.42 [-1.33; 3.41]	9.0 [6.3; 12.0]	3.4 [1.8; 5.9]	1.06 [0.61; 1.64]	1.00 [0.46; 2.14]	-0.06 [-0.89; 1.20]
Peru	2.13 [1.74; 2.59]	3.45 [1.85; 6.41]	1.31 [-0.40; 4.34]	6.5 [1.9; 16.5]	1.9 [0.5; 5.3]	1.13 [0.81; 1.45]	0.75 [0.35; 1.41]	-0.38 [-0.90; 0.37]
Philippines	1.98 [1.60; 2.44]	2.39 [1.71; 3.29]	0.41 [-0.41; 1.37]	7.8 [6.3; 9.5]	3.3 [2.4; 4.4]	1.26 [1.00; 1.60]	1.12 [0.81; 1.57]	-0.14 [-0.60; 0.37]
Poland¶	4.27 [3.99; 4.58]	3.32 [3.02; 3.66]	-0.95 [-1.37; -0.51]†	4.6 [4.3; 4.9]	1.4 [1.2; 1.5]	0.63 [0.58; 0.68]*	0.81 [0.73; 0.90]*	0.18 [0.09; 0.28]†
Portugal	3.55 [3.23; 3.88]	2.42 [2.01; 2.91]	-1.12 [-1.66; -0.54]†	2.4 [2.0; 2.7]	1.0 [0.8; 1.2]	0.74 [0.67; 0.82]*	1.03 [0.85; 1.28]	0.29 [0.09; 0.55]†
Qatar	2.77 [1.94; 3.94]	2.13 [1.37; 3.26]	-0.64 [-2.07; 0.80]	1.5 [0.8; 2.3]	0.7 [0.3; 1.3]	0.97 [0.67; 1.37]	1.18 [0.77; 1.82]	0.22 [-0.38; 0.93]
Romania	2.36 [2.20; 2.53]	2.82 [2.50; 3.18]	0.46 [0.10; 0.86]†	4.2 [3.8; 4.6]	1.5 [1.3; 1.7]	1.14 [1.05; 1.23]*	0.95 [0.83; 1.07]	-0.19 [-0.33; -0.03]†
Russian Federation¶	3.62 [3.52; 3.73]	2.85 [2.42; 3.34]	-0.78 [-1.22; -0.27]†	7.0 [6.1; 8.0]	2.5 [2.1; 2.9]	0.67 [0.63; 0.71]*	0.94 [0.80; 1.11]	0.27 [0.12; 0.45]†
Rwanda¶	1.38 [1.07; 1.76]	1.62 [1.03; 2.56]	0.25 [-0.48; 1.26]	7.0 [1.7; 16.3]	4.3 [1.1; 10.3]	0.54 [0.39; 0.90]*	1.59 [0.90; 2.54]	1.05 [0.16; 2.02]†
Saint Kitts and Nevis	2.51 [1.73; 3.67]	2.77 [1.41; 5.53]	0.26 [-1.59; 3.16]	14.7 [5.7; 29.0]	5.3 [1.8; 12.1]	1.05 [0.70; 1.53]	0.73 [0.20; 1.74]	-0.32 [-1.07; 0.76]
Saint Lucia	2.45 [1.75; 3.43]	2.18 [1.28; 3.71]	-0.26 [-1.65; 1.47]	6.9 [3.1; 11.9]	3.2 [1.3; 6.0]	1.06 [0.74; 1.51]	1.21 [0.70; 2.08]	0.15 [-0.56; 1.10]
Samoa	1.30 [0.88; 1.93]	1.68 [0.93; 3.02]	0.38 [-0.63; 1.82]	5.0 [1.1; 11.8]	3.0 [0.7; 7.4]	0.89 [0.40; 2.60]	1.55 [0.84; 2.83]	0.66 [-1.25; 1.97]
San Marino	2.68 [1.81; 3.99]	2.53 [1.20; 5.52]	-0.15 [-2.08; 2.94]	1.9 [1.4; 2.6]	0.8 [0.4; 1.3]	0.99 [0.66; 1.48]	0.99 [0.46; 2.09]	0.00 [-0.75; 1.15]
Sao Tome and Principe	1.60 [1.09; 2.36]	1.80 [0.94; 3.39]	0.19 [-1.03; 1.89]	15.8 [4.0; 40.2]	8.8 [2.1; 24.2]	0.67 [0.33; 2.12]	1.02 [0.27; 2.59]	0.35 [-1.47; 1.86]
Saudi Arabia	2.51 [1.73; 3.70]	3.05 [1.75; 5.27]	0.54 [-1.27; 2.90]	7.7 [1.9; 17.0]	2.5 [0.6; 6.0]	0.88 [0.25; 1.44]	0.83 [0.43; 1.49]	-0.04 [-0.75; 0.91]
Senegal	1.30 [0.96; 1.75]	1.54 [0.93; 2.55]	0.24 [-0.55; 1.31]	6.6 [1.5; 15.1]	4.3 [1.0; 10.2]	1.63 [0.61; 2.51]	1.68 [0.93; 2.86]	0.05 [-1.13; 1.60]
Serbia	2.67 [2.20; 3.22]	3.12 [2.38; 4.07]	0.45 [-0.47; 1.50]	3.6 [3.0; 4.2]	1.1 [0.9; 1.5]	1.01 [0.83; 1.23]	0.84 [0.64; 1.10]	-0.17 [-0.47; 0.13]
Seychelles	2.67 [1.88; 3.81]	3.31 [1.89; 5.81]	0.64 [-1.27; 3.31]	5.5 [1.2; 13.0]	1.6 [0.3; 4.4]	0.99 [0.69; 1.43]	0.79 [0.43; 1.39]	-0.21 [-0.80; 0.50]
Sierra Leone	0.84 [0.57; 1.21]	1.31 [0.88; 2.00]	0.48 [-0.12; 1.23]	20.0 [4.6; 40.7]	15.2 [3.4; 31.1]	1.05 [0.62; 3.74]	0.97 [0.39; 2.64]	-0.08 [-2.92; 1.64]
Singapore	2.27 [1.84; 2.80]	2.23 [1.56; 3.17]	-0.04 [-0.87; 1.03]	1.5 [1.0; 2.0]	0.7 [0.4; 1.0]	1.19 [0.97; 1.46]	1.12 [0.78; 1.65]	-0.07 [-0.52; 0.49]
Slovakia	3.27 [2.86; 3.71]	2.80 [2.28; 3.46]	-0.47 [-1.16; 0.30]	3.5 [3.0; 4.0]	1.2 [1.0; 1.5]	0.82 [0.72; 0.94]*	0.93 [0.75; 1.14]	0.10 [-0.11; 0.34]
Slovenia	3.49 [2.98; 4.12]	2.59 [1.72; 3.87]	-0.90 [-1.98; 0.47]	2.1 [1.6; 2.7]	0.8 [0.5; 1.2]	0.77 [0.65; 0.91]*	0.97 [0.65; 1.46]	0.20 [-0.15; 0.70]
Solomon Islands	2.55 [1.71; 3.79]	2.27 [1.13; 4.58]	-0.28 [-2.02; 2.17]	7.4 [5.2; 9.9]	3.2 [1.8; 5.3]	1.00 [0.65; 1.53]	1.18 [0.58; 2.39]	0.18 [-0.64; 1.43]
Somalia	0.77 [0.51; 1.15]	0.91 [0.42; 1.96]	0.14 [-0.49; 1.22]	22.8 [13.3; 34.8]	25.0 [15.1; 37.0]	1.01 [0.63; 2.37]	1.03 [0.37; 5.57]	0.02 [-1.25; 4.41]
South Africa	2.37 [1.74; 3.23]	1.72 [1.36; 2.17]	-0.65 [-1.59; 0.12]	16.0 [12.8; 19.5]	9.3 [7.2; 11.7]	0.63 [0.27; 1.29]	1.23 [0.66; 1.72]	0.59 [-0.28; 1.23]
South Sudan	0.72 [0.47; 1.10]	0.95 [0.44; 2.06]	0.23 [-0.42; 1.37]	21.6 [12.8; 32.2]	22.7 [13.4; 34.3]	1.02 [0.67; 1.85]	1.06 [0.36; 5.35]	0.04 [-1.00; 4.24]
Spain	3.56 [3.35; 3.77]	2.67 [2.19; 3.27]	-0.89 [-1.42; -0.26]†	1.8 [1.5; 2.0]	0.7 [0.5; 0.8]	0.76 [0.71; 0.81]*	0.94 [0.75; 1.19]	0.19 [-0.02; 0.43]
Sri Lanka¶	2.58 [2.13; 3.12]	2.40 [1.51; 3.85]	-0.18 [-1.24; 1.33]	3.4 [1.8; 6.2]	1.4 [0.7; 2.8]	0.53 [0.34; 0.80]*	1.08 [0.68; 1.71]	0.55 [0.06; 1.21]†
Saint Vincent and the Grenadines	2.70 [1.91; 3.76]	2.31 [1.27; 4.28]	-0.38 [-1.98; 1.81]	10.7 [3.9; 20.3]	4.6 [1.6; 9.5]	0.99 [0.71; 1.40]	1.05 [0.39; 2.05]	0.06 [-0.73; 1.12]
State of Palestine	3.37 [2.28; 4.96]	3.03 [1.43; 6.45]	-0.33 [-2.70; 3.26]	6.2 [4.4; 8.3]	2.1 [1.1; 3.6]	0.99 [0.66; 1.48]	0.99 [0.46; 2.10]	0.01 [-0.74; 1.15]
Sudan	0.89 [0.61; 1.30]	1.57 [0.73; 3.36]	0.68 [-0.29; 2.48]	18.8 [12.0; 26.5]	12.0 [6.7; 19.2]	0.95 [0.57; 2.59]	0.94 [0.26; 3.34]	-0.01 [-1.48; 2.32]
Suriname§	1.99 [1.54; 2.56]	1.38 [1.05; 1.83]	-0.61 [-1.27; 0.03]	7.5 [5.0; 10.4]	5.5 [3.6; 7.7]	1.27 [0.95; 1.66]	1.93 [1.44; 2.56]*	0.67 [0.03; 1.37]†

¶: Sex ratio is outlying in 1990. §: Sex ratio is outlying in 2021. *: The ratio of estimated to expected female mortality is significantly different from one. †: Change is significantly different from zero.

Continued on next page

Table 20 – continued from previous page

	Sex ratio 5q20			Sex-specific 5q20 in 2021 (per 1,000)		Estimated/Expected female 5q20		
	**1990**	**2021**	**Change 1990–2021**	**Male**	**Female**	**1990**	**2021**	**Change 1990–2021**
Eswatini	1.12<br>[0.79; 1.61]	0.81<br>[0.42; 1.60]	-0.31<br>[-0.99; 0.59]	12.8<br>[7.8; 19.0]	15.7<br>[10.1; 22.6]	1.48<br>[0.57; 2.94]	2.99<br>[0.98; 6.41]	1.51<br>[-0.94; 5.13]
Sweden	2.68<br>[2.37; 3.03]	2.25<br>[1.91; 2.65]	-0.43<br>[-0.92; 0.07]	2.9<br>[2.6; 3.3]	1.3<br>[1.1; 1.5]	1.00<br>[0.88; 1.13]	1.13<br>[0.96; 1.34]	0.13<br>[-0.08; 0.37]
Switzerland	3.31<br>[2.96; 3.69]	2.74<br>[2.24; 3.35]	-0.57<br>[-1.21; 0.14]	2.3<br>[1.9; 2.6]	0.8<br>[0.7; 1.0]	0.81<br>[0.72; 0.92]*	0.91<br>[0.73; 1.15]	0.10<br>[-0.11; 0.36]
Syria	1.76<br>[1.25; 2.49]	3.64<br>[2.14; 8.04]	1.88<br>[-1.42; 6.06]	8.3<br>[1.9; 27.7]	2.3<br>[0.3; 8.8]	1.44<br>[0.74; 2.09]	1.23<br>[0.66; 2.11]	-0.21<br>[-1.13; 0.92]
Tajikistan¶	1.23<br>[1.10; 1.36]	1.42<br>[1.25; 1.60]	0.19<br>[-0.03; 0.40]	3.0<br>[2.7; 3.3]	2.1<br>[1.9; 2.4]	2.19<br>[1.97; 2.44]*	1.80<br>[1.57; 2.06]*	-0.39<br>[-0.72; -0.04]†
Tanzania	1.17<br>[0.91; 1.50]	1.48<br>[0.88; 2.48]	0.32<br>[-0.40; 1.36]	11.5<br>[2.7; 31.5]	7.8<br>[1.8; 21.6]	1.71<br>[0.64; 2.65]	1.53<br>[0.40; 2.87]	-0.18<br>[-1.72; 1.63]
Thailand¶	3.24<br>[2.57; 4.09]	3.33<br>[2.38; 4.65]	0.10<br>[-1.19; 1.57]	9.8<br>[7.9; 12.0]	2.9<br>[2.1; 4.0]	0.74<br>[0.56; 0.95]*	0.78<br>[0.55; 1.11]	0.05<br>[-0.27; 0.42]
Timor Leste	1.95<br>[1.36; 2.80]	0.65<br>[0.39; 1.08]	-1.30<br>[-2.19; -0.54]†	12.6<br>[2.0; 43.7]	19.4<br>[3.2; 66.7]	0.93<br>[0.29; 1.76]	3.18<br>[0.81; 6.45]	2.26<br>[-0.45; 5.68]
Togo	1.25<br>[0.94; 1.70]	1.20<br>[0.68; 2.12]	-0.06<br>[-0.78; 0.93]	10.9<br>[2.6; 27.7]	9.1<br>[2.2; 23.7]	1.79<br>[0.66; 2.67]	1.94<br>[0.51; 3.80]	0.15<br>[-1.57; 2.35]
Tonga	2.75<br>[1.89; 4.02]	2.95<br>[1.56; 5.47]	0.19<br>[-1.80; 2.88]	8.2<br>[4.0; 13.6]	2.8<br>[1.2; 5.4]	0.96<br>[0.65; 1.41]	0.89<br>[0.46; 1.70]	-0.08<br>[-0.73; 0.82]
Trinidad and Tobago¶	2.02<br>[1.72; 2.36]	3.52<br>[2.45; 5.03]	1.50<br>[0.36; 3.01]†	13.9<br>[8.7; 22.0]	3.9<br>[2.3; 6.7]	1.31<br>[1.11; 1.55]*	0.64<br>[0.25; 1.00]	-0.67<br>[-1.11; -0.26]†
Tunisia	2.22<br>[1.63; 3.04]	3.07<br>[2.05; 4.60]	0.85<br>[-0.50; 2.52]	6.8<br>[4.3; 9.9]	2.2<br>[1.3; 3.5]	1.20<br>[0.87; 1.66]	0.87<br>[0.58; 1.31]	-0.33<br>[-0.88; 0.23]
Turkey	2.47<br>[1.67; 3.65]	2.71<br>[2.14; 3.41]	0.23<br>[-1.09; 1.30]	3.0<br>[2.4; 3.7]	1.1<br>[0.9; 1.4]	0.97<br>[0.32; 1.52]	0.94<br>[0.75; 1.20]	-0.02<br>[-0.61; 0.63]
Turkmenistan¶§	1.56<br>[1.30; 1.88]	1.50<br>[0.96; 2.32]	-0.07<br>[-0.70; 0.81]	6.8<br>[3.5; 12.1]	4.6<br>[2.3; 8.3]	1.68<br>[1.37; 2.04]*	1.77<br>[1.11; 2.77]*	0.09<br>[-0.67; 1.13]
Turks and Caicos Islands	2.59<br>[1.76; 3.80]	2.66<br>[1.23; 5.59]	0.07<br>[-1.84; 3.11]	4.6<br>[3.1; 6.2]	1.7<br>[0.9; 3.0]	1.03<br>[0.70; 1.52]	1.01<br>[0.48; 2.09]	-0.02<br>[-0.77; 1.12]
Tuvalu	2.72<br>[1.87; 3.97]	3.06<br>[1.47; 6.49]	0.34<br>[-1.80; 3.94]	8.6<br>[6.1; 11.4]	2.8<br>[1.5; 4.9]	0.88<br>[0.55; 1.34]	0.87<br>[0.40; 1.83]	-0.01<br>[-0.70; 1.03]
Uganda	0.90<br>[0.73; 1.10]	1.47<br>[0.91; 2.35]	0.57<br>[-0.03; 1.47]	16.8<br>[4.0; 40.5]	11.4<br>[2.6; 28.4]	0.87<br>[0.64; 1.48]	1.20<br>[0.36; 2.65]	0.33<br>[-0.72; 1.79]
Ukraine¶	3.69<br>[3.49; 3.88]	3.03<br>[2.77; 3.31]	-0.66<br>[-0.98; -0.33]†	5.7<br>[5.3; 6.1]	1.9<br>[1.7; 2.1]	0.69<br>[0.64; 0.74]*	0.89<br>[0.81; 0.98]*	0.20<br>[0.11; 0.30]†
United Arab Emirates	2.69<br>[1.80; 3.97]	2.61<br>[1.23; 5.55]	-0.08<br>[-2.09; 3.02]	3.2<br>[2.4; 4.1]	1.2<br>[0.6; 2.4]	1.00<br>[0.68; 1.49]	1.01<br>[0.48; 2.12]	0.01<br>[-0.75; 1.18]
United Kingdom	2.92<br>[2.74; 3.11]	2.39<br>[1.88; 3.02]	-0.53<br>[-1.06; 0.13]	2.7<br>[2.2; 3.2]	1.1<br>[0.9; 1.4]	0.92<br>[0.86; 0.99]*	1.05<br>[0.83; 1.35]	0.13<br>[-0.10; 0.43]
United States of America	3.22<br>[3.14; 3.31]	2.70<br>[2.19; 3.32]	-0.53<br>[-1.05; 0.11]	8.1<br>[7.0; 9.3]	3.0<br>[2.4; 3.6]	0.83<br>[0.79; 0.87]*	0.99<br>[0.80; 1.23]	0.16<br>[-0.03; 0.40]
Uruguay§	2.51<br>[2.16; 2.91]	3.76<br>[3.00; 4.75]	1.26<br>[0.40; 2.29]†	9.0<br>[7.5; 10.6]	2.4<br>[1.9; 3.0]	1.07<br>[0.92; 1.25]	0.70<br>[0.55; 0.89]*	-0.37<br>[-0.61; -0.13]†
Uzbekistan¶§	1.55<br>[1.29; 1.88]	1.27<br>[1.02; 1.58]	-0.28<br>[-0.70; 0.13]	4.3<br>[3.8; 4.8]	3.3<br>[2.9; 3.9]	1.73<br>[1.41; 2.10]*	2.10<br>[1.70; 2.60]*	0.38<br>[-0.18; 0.97]
Vanuatu	2.58<br>[1.74; 3.86]	2.65<br>[1.32; 5.34]	0.07<br>[-1.85; 2.91]	8.7<br>[6.2; 11.6]	3.3<br>[1.8; 5.5]	0.99<br>[0.64; 1.52]	1.00<br>[0.48; 2.03]	0.01<br>[-0.72; 1.11]
Venezuela (Bolivarian Republic of)§	2.98<br>[2.43; 3.66]	5.75<br>[3.72; 9.09]	2.77<br>[0.59; 6.14]†	26.7<br>[14.2; 43.5]	4.6<br>[2.2; 8.6]	0.87<br>[0.70; 1.08]	0.15<br>[0.08; 0.45]*	-0.71<br>[-0.93; -0.39]†
Vietnam	2.35<br>[1.63; 3.40]	2.42<br>[1.49; 3.94]	0.07<br>[-1.37; 1.78]	4.8<br>[2.6; 7.6]	2.0<br>[1.0; 3.4]	1.09<br>[0.68; 1.62]	1.10<br>[0.68; 1.78]	0.01<br>[-0.68; 0.82]
Yemen	2.56<br>[1.72; 3.74]	5.51<br>[2.70; 11.30]	2.95<br>[-0.14; 8.84]	22.3<br>[16.0; 29.8]	4.0<br>[2.1; 7.0]	0.99<br>[0.64; 1.51]	0.99<br>[0.47; 2.15]	0.00<br>[-0.76; 1.21]
Zambia	0.76<br>[0.62; 0.92]	1.44<br>[0.91; 2.29]	0.68<br>[0.12; 1.55]†	14.1<br>[4.8; 27.6]	9.8<br>[3.2; 19.5]	1.06<br>[0.77; 2.10]	1.50<br>[0.43; 2.76]	0.44<br>[-0.95; 1.72]
Zimbabwe	1.18<br>[0.92; 1.51]	1.20<br>[0.73; 2.01]	0.02<br>[-0.57; 0.86]	16.0<br>[5.6; 33.0]	13.3<br>[4.7; 27.2]	1.85<br>[0.79; 2.64]	1.64<br>[0.44; 3.40]	-0.21<br>[-1.76; 1.86]

Table 21: **Estimates and 90% uncertainty intervals for sex ratios for 10q15 in 1990 and 2021, the change in sex ratios from 1990 to 2021, sex-specific 10q15 in 2021, and ratios of estimated to expected female 10q15 and their change from 1990 to 2021 for the world, UNICEF regions, and all countries.** ¶: Sex ratio is outlying in 1990. §: Sex ratio is outlying in 2021. *: The ratio of estimated to expected female mortality is significantly different from one. †: Change is significantly different from zero.

	Sex ratio 10q15			Sex-specific 10q15 in 2021 (per 1,000)		Estimated/Expected female 10q15		
	1990	2021	Change 1990–2021	Male	Female	1990	2021	Change 1990–2021
World¶	1.29 [1.23; 1.35]	1.65 [1.52; 1.75]	0.36 [0.22; 0.49]†	13.2 [12.7; 14.9]	8.0 [7.7; 9.2]	1.25 [1.16; 1.34]*	1.04 [0.89; 1.16]	-0.21 [-0.38; -0.07]†
South Asia¶	0.82 [0.76; 0.89]	1.44 [1.21; 1.70]	0.61 [0.38; 0.89]†	10.9 [9.3; 13.7]	7.6 [6.6; 9.3]	2.47 [2.18; 2.75]*	1.62 [0.99; 1.96]	-0.85 [-1.51; -0.39]†
Europe and Central Asia	2.62 [2.49; 2.75]	2.20 [2.12; 2.28]	-0.42 [-0.57; -0.27]†	6.0 [5.9; 6.2]	2.7 [2.7; 2.8]	0.88 [0.71; 0.94]*	1.12 [1.07; 1.17]*	0.23 [0.16; 0.41]†
Middle East and North Africa	1.81 [1.64; 2.04]	2.72 [2.35; 3.04]	0.91 [0.44; 1.27]†	12.7 [11.6; 14.8]	4.7 [4.3; 5.6]	1.11 [0.67; 1.32]	1.03 [0.87; 1.18]	-0.09 [-0.35; 0.37]
Sub-Saharan Africa	1.06 [0.98; 1.13]	1.30 [1.17; 1.42]	0.24 [0.09; 0.40]†	25.2 [23.4; 30.7]	19.3 [17.8; 24.2]	0.90 [0.80; 1.01]	0.96 [0.78; 1.16]	0.06 [-0.15; 0.27]
Latin America and Caribbean¶§	2.34 [2.22; 2.45]	3.33 [3.05; 3.54]	0.99 [0.69; 1.24]†	17.6 [16.8; 18.9]	5.3 [5.0; 5.9]	0.78 [0.70; 0.88]*	0.59 [0.51; 0.68]*	-0.19 [-0.32; -0.08]†
East Asia and Pacific	1.85 [1.62; 2.09]	2.29 [1.88; 2.70]	0.44 [-0.05; 0.93]	8.0 [6.2; 11.5]	3.5 [2.8; 5.1]	1.14 [0.98; 1.33]	1.05 [0.73; 1.27]	-0.09 [-0.44; 0.19]
North America¶	2.98 [2.92; 3.03]	2.55 [2.21; 2.91]	-0.43 [-0.77; -0.06]†	11.6 [10.6; 12.7]	4.5 [4.0; 5.2]	0.82 [0.80; 0.85]*	0.99 [0.87; 1.14]	0.17 [0.04; 0.32]†
Afghanistan§	0.83 [0.63; 1.08]	0.62 [0.44; 0.88]	-0.21 [-0.52; 0.12]	23.3 [11.9; 47.5]	37.5 [19.6; 74.2]	0.99 [0.69; 1.79]	2.85 [1.07; 5.06]*	1.85 [-0.10; 4.03]
Albania	2.57 [2.33; 2.84]	1.72 [1.44; 2.03]	-0.85 [-1.23; -0.45]†	4.4 [3.9; 5.1]	2.6 [2.2; 3.0]	0.97 [0.88; 1.08]	1.39 [1.17; 1.66]*	0.42 [0.17; 0.70]†
Algeria¶§	1.66 [1.40; 1.96]	1.80 [1.60; 2.02]	0.14 [-0.22; 0.49]	6.9 [6.4; 7.5]	3.8 [3.5; 4.2]	1.46 [1.21; 1.74]*	1.38 [1.23; 1.55]*	-0.07 [-0.39; 0.22]
Andorra	2.58 [1.97; 3.31]	2.89 [1.77; 4.55]	0.31 [-1.08; 2.11]	4.5 [3.4; 5.9]	1.6 [1.0; 2.4]	0.97 [0.75; 1.26]	0.84 [0.53; 1.33]	-0.12 [-0.57; 0.43]
Angola	0.72 [0.55; 0.95]	1.74 [1.21; 2.48]	1.01 [0.41; 1.79]†	37.7 [28.1; 49.1]	21.7 [15.4; 30.1]	1.01 [0.75; 1.55]	0.57 [0.32; 1.27]	-0.44 [-0.95; 0.25]
Anguilla	2.47 [1.88; 3.19]	2.50 [1.50; 4.07]	0.03 [-1.23; 1.75]	6.9 [5.2; 9.1]	2.8 [1.8; 4.2]	1.00 [0.76; 1.32]	1.02 [0.62; 1.64]	0.02 [-0.51; 0.68]
Antigua and Barbuda	2.26 [1.75; 2.87]	2.67 [1.71; 4.07]	0.41 [-0.77; 1.89]	8.3 [5.0; 13.5]	3.1 [1.8; 5.5]	0.86 [0.31; 1.27]	0.93 [0.59; 1.49]	0.08 [-0.48; 0.86]
Argentina¶	2.04 [1.95; 2.13]	2.54 [2.25; 2.84]	0.49 [0.20; 0.81]†	9.5 [8.7; 10.2]	3.7 [3.3; 4.2]	1.22 [1.16; 1.28]*	1.00 [0.89; 1.13]	-0.22 [-0.34; -0.08]†
Armenia	2.36 [2.12; 2.61]	3.09 [2.57; 3.72]	0.74 [0.15; 1.41]†	7.7 [6.7; 8.8]	2.5 [2.1; 3.0]	1.08 [0.97; 1.20]	0.82 [0.68; 0.98]*	-0.26 [-0.45; -0.07]†
Australia	2.73 [2.57; 2.89]	2.43 [2.13; 2.77]	-0.29 [-0.64; 0.07]	5.4 [5.0; 5.9]	2.2 [2.0; 2.5]	0.92 [0.86; 0.98]*	1.00 [0.88; 1.15]	0.09 [-0.05; 0.24]
Austria¶	3.30 [3.04; 3.57]	2.47 [2.09; 2.91]	-0.83 [-1.30; -0.31]†	4.2 [3.7; 4.7]	1.7 [1.4; 2.0]	0.75 [0.69; 0.82]*	0.96 [0.81; 1.14]	0.21 [0.05; 0.40]†
Azerbaijan	2.21 [2.05; 2.37]	2.20 [1.99; 2.43]	-0.01 [-0.28; 0.27]	10.9 [9.9; 11.9]	5.0 [4.5; 5.5]	1.15 [1.06; 1.23]*	1.13 [1.02; 1.25]*	-0.02 [-0.15; 0.13]
Bahamas	2.11 [1.72; 2.58]	3.41 [2.47; 4.57]	1.30 [0.22; 2.55]†	15.7 [12.6; 19.2]	4.6 [3.5; 6.1]	1.15 [0.92; 1.43]	0.72 [0.51; 1.01]	-0.43 [-0.79; -0.06]†
Bahrain	2.18 [1.78; 2.66]	1.71 [1.41; 2.08]	-0.47 [-1.04; 0.08]	5.1 [4.2; 6.0]	3.0 [2.4; 3.7]	1.14 [0.93; 1.40]	1.42 [1.17; 1.72]*	0.28 [-0.08; 0.65]
Bangladesh¶§	0.84 [0.72; 0.97]	1.34 [1.03; 1.75]	0.51 [0.16; 0.92]†	9.3 [7.3; 11.2]	6.9 [5.4; 8.6]	2.59 [2.07; 3.13]*	1.91 [1.47; 2.48]*	-0.68 [-1.39; 0.10]
Barbados	2.09 [1.71; 2.55]	1.90 [1.22; 2.89]	-0.19 [-1.04; 0.91]	7.5 [4.1; 13.6]	3.9 [2.0; 7.7]	1.18 [0.96; 1.45]	1.31 [0.85; 2.04]	0.13 [-0.44; 0.92]
Belarus¶	3.25 [3.05; 3.48]	2.77 [2.29; 3.33]	-0.49 [-1.01; 0.12]	5.6 [4.6; 6.7]	2.0 [1.7; 2.5]	0.74 [0.69; 0.80]*	0.89 [0.74; 1.07]	0.15 [-0.01; 0.34]
Belgium	2.72 [2.52; 2.92]	2.21 [1.88; 2.60]	-0.50 [-0.90; -0.07]†	3.6 [3.2; 4.0]	1.6 [1.4; 1.9]	0.92 [0.85; 1.00]*	1.07 [0.90; 1.27]	0.15 [-0.04; 0.36]
Belize	2.51 [1.98; 3.20]	2.75 [2.03; 3.65]	0.23 [-0.77; 1.31]	16.7 [12.5; 22.2]	6.1 [4.4; 8.6]	1.00 [0.78; 1.28]	0.87 [0.59; 1.21]	-0.13 [-0.54; 0.28]
Benin	1.08 [0.89; 1.30]	1.26 [0.93; 1.68]	0.18 [-0.24; 0.64]	24.3 [14.8; 40.9]	19.3 [12.5; 31.6]	1.29 [0.76; 2.03]	1.33 [0.58; 2.30]	0.04 [-1.05; 1.17]
Bhutan	1.16 [0.92; 1.45]	1.82 [1.17; 2.77]	0.66 [-0.07; 1.66]	15.2 [7.7; 31.3]	8.3 [4.2; 18.1]	0.88 [0.52; 1.76]	1.24 [0.47; 2.06]	0.36 [-0.76; 1.28]
Bolivia (Plurinational State of)	1.33 [1.11; 1.58]	2.01 [1.30; 3.03]	0.68 [-0.09; 1.76]	12.7 [5.5; 30.9]	6.4 [2.9; 15.0]	1.32 [0.77; 1.83]	1.17 [0.45; 1.91]	-0.15 [-1.02; 0.79]
Bosnia and Herzegovina	2.64 [2.38; 2.93]	3.09 [2.32; 4.07]	0.45 [-0.38; 1.46]	6.2 [4.2; 8.9]	2.0 [1.3; 3.0]	0.96 [0.86; 1.07]	0.81 [0.61; 1.07]	-0.15 [-0.38; 0.12]
Botswana	1.00 [0.77; 1.29]	2.06 [1.38; 3.00]	1.06 [0.31; 2.04]†	14.2 [8.1; 24.4]	6.9 [3.9; 12.6]	0.94 [0.63; 1.78]	1.14 [0.63; 1.76]	0.20 [-0.76; 0.90]
Brazil¶§	2.97 [2.64; 3.36]	4.33 [3.58; 5.20]	1.35 [0.51; 2.28]†	19.4 [18.0; 20.8]	4.5 [3.8; 5.3]	0.77 [0.68; 0.88]*	0.54 [0.44; 0.66]*	-0.24 [-0.38; -0.09]†
British Virgin Islands	2.46 [1.87; 3.17]	2.52 [1.49; 4.08]	0.06 [-1.23; 1.72]	11.4 [8.4; 15.0]	4.5 [2.9; 7.0]	1.00 [0.76; 1.31]	1.02 [0.61; 1.69]	0.02 [-0.50; 0.73]
Brunei	2.59 [2.08; 3.21]	1.64 [1.14; 2.36]	-0.94 [-1.77; -0.03]†	3.8 [2.7; 5.2]	2.3 [1.6; 3.3]	0.90 [0.70; 1.14]	1.46 [1.02; 2.08]*	0.56 [0.05; 1.23]†
Bulgaria	2.34 [2.18; 2.52]	2.32 [2.05; 2.62]	-0.03 [-0.35; 0.31]	8.0 [7.4; 8.6]	3.4 [3.1; 3.8]	1.06 [0.99; 1.15]	1.09 [0.97; 1.24]	0.03 [-0.12; 0.19]
Burkina Faso	0.81 [0.69; 0.97]	1.47 [0.97; 2.18]	0.65 [0.13; 1.38]†	21.1 [10.1; 43.8]	14.4 [6.6; 31.3]	1.42 [0.96; 2.17]	1.36 [0.48; 2.32]	-0.07 [-1.23; 1.02]
Burundi	0.74 [0.56; 0.97]	1.79 [1.26; 2.53]	1.06 [0.44; 1.82]†	21.7 [12.0; 41.8]	12.1 [7.1; 22.9]	1.00 [0.75; 1.45]	1.06 [0.39; 1.72]	0.06 [-0.76; 0.79]
Cambodia	1.29 [1.07; 1.55]	2.05 [1.35; 3.01]	0.75 [-0.01; 1.74]	10.7 [5.5; 21.8]	5.2 [2.6; 11.3]	0.67 [0.48; 1.04]	1.19 [0.70; 1.84]	0.52 [-0.13; 1.20]
Cameroon	1.14 [0.94; 1.37]	1.24 [0.90; 1.68]	0.10 [-0.32; 0.59]	32.1 [20.5; 48.4]	26.0 [16.4; 40.0]	1.10 [0.70; 1.80]	0.96 [0.51; 2.03]	-0.14 [-0.97; 1.01]
Canada	2.91 [2.76; 3.07]	2.00 [1.77; 2.27]	-0.91 [-1.19; -0.60]†	6.3 [5.8; 6.8]	3.1 [2.8; 3.5]	0.85 [0.81; 0.90]*	1.25 [1.10; 1.42]*	0.40 [0.24; 0.57]†

¶: Sex ratio is outlying in 1990. §: Sex ratio is outlying in 2021. *: The ratio of estimated to expected female mortality is significantly different from one. †: Change is significantly different from zero.

Continued on next page

Table 21 – continued from previous page

	Sex ratio 10q15			Sex-specific 10q15 in 2021 (per 1,000)		Estimated/Expected female 10q15		
	1990	2021	Change 1990–2021	Male	Female	1990	2021	Change 1990–2021
Cape Verde	2.20 [1.75; 2.74]	2.17 [1.69; 2.74]	-0.03 [-0.78; 0.70]	7.7 [5.6; 10.3]	3.5 [2.7; 4.7]	1.14 [0.90; 1.43]	1.19 [0.93; 1.49]	0.05 [-0.34; 0.44]
Central African Republic	0.86 [0.72; 1.03]	1.15 [0.70; 1.92]	0.29 [-0.20; 1.09]	41.0 [15.8; 109.0]	35.8 [12.7; 101.3]	1.01 [0.75; 1.55]	1.19 [0.53; 3.27]	0.17 [-0.69; 2.29]
Chad	1.54 [1.30; 1.84]	1.28 [0.89; 1.83]	-0.26 [-0.77; 0.35]	37.7 [20.9; 69.3]	29.3 [16.6; 53.9]	1.22 [0.86; 1.80]	0.74 [0.42; 1.80]	-0.48 [-1.13; 0.65]
Chile	2.94 [2.77; 3.12]	2.54 [2.16; 2.97]	-0.41 [-0.83; 0.04]	8.1 [7.4; 8.8]	3.2 [2.8; 3.6]	0.86 [0.80; 0.92]*	1.00 [0.86; 1.18]	0.15 [-0.01; 0.33]
China	1.89 [1.53; 2.35]	2.48 [1.67; 3.56]	0.59 [-0.38; 1.75]	5.2 [2.7; 10.1]	2.1 [1.1; 4.2]	1.25 [0.98; 1.57]	1.00 [0.69; 1.46]	-0.25 [-0.72; 0.30]
Colombia¶§	2.56 [2.05; 3.14]	3.80 [3.13; 4.57]	1.24 [0.32; 2.16]†	16.6 [15.4; 17.9]	4.4 [3.7; 5.2]	0.38 [0.28; 0.62]*	0.64 [0.53; 0.78]*	0.26 [0.00; 0.44]
Comoros	1.50 [1.18; 1.89]	1.81 [1.18; 2.74]	0.31 [-0.48; 1.30]	9.9 [6.2; 15.6]	5.5 [3.2; 9.2]	0.90 [0.45; 1.60]	1.35 [0.86; 2.13]	0.45 [-0.42; 1.38]
Congo	0.81 [0.63; 1.05]	1.68 [1.06; 2.61]	0.87 [0.16; 1.82]†	19.0 [9.4; 38.8]	11.3 [5.4; 24.5]	0.94 [0.69; 1.40]	1.24 [0.39; 2.23]	0.31 [-0.68; 1.33]
Democratic Republic of the Congo	0.94 [0.73; 1.23]	1.01 [0.70; 1.46]	0.07 [-0.38; 0.59]	36.2 [19.6; 69.3]	35.9 [18.9; 70.3]	0.87 [0.60; 1.46]	0.94 [0.53; 2.33]	0.07 [-0.67; 1.46]
Cook Islands¶	2.68 [2.10; 3.38]	2.58 [1.62; 3.97]	-0.10 [-1.33; 1.46]	12.8 [6.7; 32.2]	4.9 [2.4; 13.5]	0.67 [0.38; 0.97]*	0.85 [0.36; 1.49]	0.18 [-0.39; 0.88]
Costa Rica	2.18 [1.92; 2.49]	2.97 [2.44; 3.58]	0.79 [0.17; 1.46]†	9.8 [8.6; 11.1]	3.3 [2.7; 4.0]	1.16 [1.02; 1.32]*	0.86 [0.71; 1.04]	-0.30 [-0.53; -0.07]†
Cote d’Ivoire	1.05 [0.87; 1.25]	1.26 [0.83; 1.90]	0.21 [-0.27; 0.90]	28.9 [14.4; 58.6]	22.9 [11.0; 49.0]	1.21 [0.77; 1.95]	1.04 [0.45; 2.42]	-0.17 [-1.13; 1.28]
Croatia¶	3.34 [3.03; 3.68]	2.99 [2.52; 3.50]	-0.36 [-0.93; 0.23]	5.9 [5.3; 6.5]	2.0 [1.7; 2.3]	0.74 [0.67; 0.82]*	0.82 [0.69; 0.97]*	0.07 [-0.07; 0.24]
Cuba¶	1.67 [1.58; 1.76]	1.90 [1.64; 2.19]	0.23 [-0.04; 0.53]	6.1 [5.6; 6.7]	3.2 [2.9; 3.6]	1.49 [1.40; 1.58]*	1.31 [1.14; 1.50]*	-0.18 [-0.38; 0.03]
Cyprus	2.65 [2.13; 3.26]	2.58 [1.79; 3.66]	-0.06 [-1.13; 1.18]	4.2 [3.3; 5.3]	1.6 [1.2; 2.3]	0.95 [0.77; 1.18]	0.94 [0.66; 1.34]	-0.01 [-0.39; 0.44]
Czech Republic	2.64 [2.46; 2.84]	2.49 [2.19; 2.83]	-0.15 [-0.52; 0.24]	4.9 [4.5; 5.3]	2.0 [1.8; 2.2]	0.96 [0.88; 1.03]	0.97 [0.86; 1.11]	0.02 [-0.13; 0.18]
Denmark	2.45 [2.23; 2.69]	2.15 [1.85; 2.51]	-0.30 [-0.69; 0.12]	3.3 [2.9; 3.6]	1.5 [1.3; 1.7]	1.03 [0.94; 1.14]	1.10 [0.93; 1.30]	0.06 [-0.14; 0.29]
Djibouti	0.90 [0.69; 1.17]	1.54 [0.93; 2.50]	0.63 [-0.05; 1.62]	30.6 [21.7; 41.0]	19.9 [13.2; 28.9]	1.01 [0.65; 2.09]	0.96 [0.38; 2.20]	-0.05 [-1.06; 1.11]
Dominica	2.60 [2.02; 3.32]	2.63 [1.53; 4.34]	0.03 [-1.32; 1.87]	9.2 [5.0; 16.6]	3.5 [1.7; 7.1]	0.96 [0.73; 1.24]	0.95 [0.55; 1.64]	-0.01 [-0.51; 0.73]
Dominican Republic	2.18 [1.75; 2.69]	2.52 [1.55; 3.90]	0.34 [-0.82; 1.85]	17.2 [8.6; 33.0]	6.8 [3.2; 14.4]	1.01 [0.66; 1.32]	0.88 [0.31; 1.53]	-0.13 [-0.79; 0.64]
Ecuador¶	1.93 [1.83; 2.04]	2.54 [2.24; 2.86]	0.61 [0.29; 0.94]†	12.2 [11.3; 13.1]	4.8 [4.3; 5.4]	1.21 [1.13; 1.30]*	1.00 [0.88; 1.14]	-0.21 [-0.35; -0.06]†
Egypt¶	1.36 [1.21; 1.54]	2.36 [1.90; 2.91]	1.00 [0.50; 1.57]†	10.7 [9.2; 12.3]	4.5 [3.7; 5.5]	1.78 [1.56; 2.01]*	1.05 [0.85; 1.31]	-0.72 [-1.03; -0.39]†
El Salvador¶§	4.81 [4.40; 5.25]	4.90 [4.00; 5.99]	0.09 [-0.92; 1.23]	17.0 [15.0; 19.2]	3.5 [2.8; 4.3]	0.28 [0.24; 0.33]*	0.49 [0.40; 0.60]*	0.21 [0.10; 0.33]†
Equatorial Guinea	0.91 [0.71; 1.18]	1.61 [0.98; 2.57]	0.70 [-0.01; 1.67]	29.7 [21.5; 39.4]	18.4 [12.3; 27.2]	1.02 [0.65; 2.03]	0.97 [0.40; 2.05]	-0.05 [-1.01; 0.98]
Eritrea	0.81 [0.62; 1.05]	1.96 [1.14; 3.21]	1.15 [0.29; 2.42]†	26.7 [19.2; 35.4]	13.6 [8.9; 20.4]	1.01 [0.69; 1.94]	0.96 [0.37; 1.90]	-0.05 [-0.96; 0.90]
Estonia¶	3.12 [2.80; 3.48]	1.77 [1.37; 2.26]	-1.36 [-1.89; -0.76]†	4.4 [3.5; 5.4]	2.5 [2.0; 3.1]	0.74 [0.65; 0.83]*	1.36 [1.06; 1.74]*	0.62 [0.31; 1.02]†
Ethiopia¶	1.27 [1.07; 1.51]	1.90 [1.30; 2.72]	0.63 [-0.04; 1.48]	20.9 [11.8; 38.2]	11.0 [6.0; 20.7]	0.66 [0.55; 0.81]*	1.01 [0.41; 1.68]	0.35 [-0.28; 1.04]
Federated States of Micronesia	2.21 [1.70; 2.85]	2.41 [1.45; 3.88]	0.20 [-0.99; 1.75]	15.4 [11.3; 20.2]	6.4 [4.1; 9.8]	0.99 [0.71; 1.34]	1.02 [0.61; 1.69]	0.03 [-0.51; 0.75]
Fiji§	1.95 [1.51; 2.49]	1.50 [1.12; 1.96]	-0.44 [-1.12; 0.22]	12.0 [9.7; 14.4]	8.0 [6.3; 10.0]	1.05 [0.43; 1.49]	1.68 [1.27; 2.24]*	0.63 [0.00; 1.45]†
Finland	2.97 [2.71; 3.25]	2.32 [2.02; 2.66]	-0.65 [-1.06; -0.23]†	6.5 [6.0; 7.2]	2.8 [2.5; 3.2]	0.84 [0.77; 0.93]*	1.08 [0.94; 1.24]	0.24 [0.08; 0.42]†
France	3.00 [2.88; 3.13]	2.48 [2.19; 2.82]	-0.52 [-0.84; -0.17]†	4.0 [3.7; 4.3]	1.6 [1.4; 1.8]	0.85 [0.81; 0.89]*	0.95 [0.83; 1.09]	0.11 [-0.02; 0.25]
Gabon	1.34 [1.08; 1.67]	1.68 [1.09; 2.57]	0.34 [-0.38; 1.29]	18.6 [8.8; 41.9]	11.1 [5.1; 26.1]	1.19 [0.59; 1.94]	1.17 [0.39; 2.15]	-0.02 [-1.16; 1.16]
The Gambia	0.94 [0.74; 1.21]	1.80 [1.30; 2.48]	0.86 [0.27; 1.57]†	23.3 [17.7; 30.3]	12.9 [9.4; 17.7]	1.03 [0.65; 2.05]	1.15 [0.70; 1.72]	0.12 [-0.87; 0.75]
Georgia	2.79 [2.38; 3.26]	2.64 [2.19; 3.19]	-0.15 [-0.81; 0.54]	10.1 [9.2; 11.2]	3.8 [3.3; 4.5]	0.90 [0.77; 1.06]	0.96 [0.79; 1.15]	0.05 [-0.18; 0.29]
Germany	2.60 [2.50; 2.70]	2.25 [2.11; 2.40]	-0.35 [-0.52; -0.17]†	3.5 [3.4; 3.7]	1.6 [1.5; 1.7]	0.97 [0.93; 1.01]	1.05 [0.95; 1.16]	0.08 [-0.03; 0.20]
Ghana	1.14 [0.90; 1.46]	1.54 [1.04; 2.25]	0.39 [-0.22; 1.17]	17.4 [9.5; 34.2]	11.3 [6.0; 23.7]	0.95 [0.56; 2.05]	1.36 [0.55; 2.26]	0.41 [-0.93; 1.39]
Greece	3.14 [2.90; 3.38]	2.77 [2.35; 3.28]	-0.36 [-0.85; 0.19]	4.0 [3.6; 4.4]	1.4 [1.2; 1.7]	0.80 [0.74; 0.87]*	0.86 [0.72; 1.02]	0.05 [-0.10; 0.23]
Grenada	2.22 [1.72; 2.83]	2.51 [1.64; 3.82]	0.29 [-0.82; 1.74]	8.0 [5.3; 12.1]	3.2 [1.8; 5.6]	1.11 [0.85; 1.45]	0.98 [0.62; 1.57]	-0.13 [-0.63; 0.53]
Guatemala¶	1.67 [1.60; 1.75]	2.69 [2.16; 3.29]	1.02 [0.49; 1.63]†	21.0 [15.9; 27.5]	7.8 [5.8; 10.5]	1.23 [1.12; 1.33]*	0.83 [0.58; 1.06]	-0.40 [-0.66; -0.14]†
Guinea	0.99 [0.82; 1.19]	1.08 [0.74; 1.59]	0.09 [-0.33; 0.63]	32.1 [17.2; 59.4]	29.8 [15.9; 54.3]	1.11 [0.72; 1.75]	1.07 [0.52; 2.55]	-0.03 [-0.91; 1.54]
Guinea-Bissau	0.83 [0.65; 1.07]	1.26 [0.85; 1.88]	0.44 [-0.07; 1.08]	26.5 [19.3; 35.4]	21.0 [14.8; 29.2]	1.02 [0.68; 1.99]	1.45 [0.64; 2.52]	0.42 [-0.62; 1.47]
Guyana¶	1.91 [1.71; 2.15]	2.18 [1.57; 2.95]	0.26 [-0.40; 1.05]	22.6 [13.7; 36.6]	10.4 [6.4; 17.1]	1.24 [1.08; 1.40]*	0.93 [0.34; 1.49]	-0.31 [-0.93; 0.27]
Haiti	0.76 [0.63; 0.94]	1.45 [0.99; 2.11]	0.69 [0.18; 1.37]†	20.5 [12.1; 35.2]	14.1 [8.1; 24.6]	1.33 [0.88; 2.31]	1.43 [0.52; 2.33]	0.10 [-1.25; 1.13]
Honduras	2.07 [1.59; 2.67]	2.56 [1.54; 4.02]	0.49 [-0.74; 2.05]	14.3 [10.5; 18.6]	5.6 [3.6; 8.6]	0.96 [0.51; 1.37]	0.98 [0.60; 1.61]	0.02 [-0.54; 0.79]
Hungary	2.56 [2.39; 2.75]	2.26 [1.91; 2.66]	-0.31 [-0.70; 0.13]	4.6 [4.1; 5.1]	2.0 [1.7; 2.4]	0.98 [0.91; 1.05]	1.07 [0.90; 1.26]	0.09 [-0.09; 0.30]
Iceland	2.65 [2.13; 3.27]	2.67 [1.82; 3.83]	0.02 [-1.08; 1.33]	3.8 [2.9; 5.0]	1.4 [0.9; 2.1]	0.94 [0.75; 1.17]	0.89 [0.61; 1.29]	-0.05 [-0.42; 0.40]

¶: Sex ratio is outlying in 1990. §: Sex ratio is outlying in 2021. *: The ratio of estimated to expected female mortality is significantly different from one. †: Change is significantly different from zero.

Continued on next page

	Sex ratio 10q15			Sex-specific 10q15 in 2021 (per 1,000)		Estimated/Expected female 10q15		
	1990	2021	Change 1990–2021	Male	Female	1990	2021	Change 1990–2021
India¶§	0.77 [0.70; 0.85]	1.41 [1.16; 1.71]	0.64 [0.38; 0.95]†	9.8 [8.4; 11.3]	7.0 [5.9; 8.2]	2.82 [2.46; 3.19]*	1.79 [1.47; 2.17]*	-1.03 [-1.52; -0.49]†
Indonesia¶	1.44 [1.19; 1.72]	2.35 [1.57; 3.47]	0.92 [0.04; 2.09]†	13.3 [6.6; 27.8]	5.7 [2.9; 12.0]	1.60 [1.29; 1.99]*	1.00 [0.48; 1.53]	-0.60 [-1.25; 0.04]
Iran (Islamic Republic of)	1.58 [1.21; 2.04]	2.53 [1.94; 3.24]	0.95 [0.18; 1.78]†	13.8 [10.4; 18.2]	5.4 [4.0; 7.5]	0.95 [0.44; 1.35]	0.96 [0.74; 1.26]	0.01 [-0.47; 0.60]
Iraq	2.36 [1.83; 3.01]	2.30 [1.42; 3.62]	-0.06 [-1.20; 1.40]	11.4 [8.5; 14.8]	4.9 [3.2; 7.4]	1.01 [0.77; 1.31]	1.08 [0.67; 1.75]	0.07 [-0.45; 0.79]
Ireland	2.84 [2.57; 3.14]	2.52 [2.14; 2.96]	-0.32 [-0.80; 0.19]	3.0 [2.6; 3.5]	1.2 [1.0; 1.4]	0.90 [0.81; 0.99]*	0.94 [0.79; 1.13]	0.04 [-0.14; 0.25]
Israel	2.52 [2.29; 2.78]	2.78 [2.44; 3.16]	0.26 [-0.16; 0.69]	4.0 [3.7; 4.3]	1.4 [1.3; 1.6]	1.00 [0.90; 1.10]	0.85 [0.74; 0.98]*	-0.15 [-0.29; 0.01]
Italy	3.29 [3.15; 3.44]	2.49 [2.18; 2.83]	-0.80 [-1.14; -0.43]†	2.8 [2.6; 3.0]	1.1 [1.0; 1.3]	0.77 [0.73; 0.80]*	0.94 [0.81; 1.10]	0.18 [0.04; 0.34]†
Jamaica	2.37 [1.81; 3.06]	2.86 [1.78; 4.39]	0.49 [-0.80; 2.12]	12.7 [9.6; 16.5]	4.5 [3.0; 6.7]	0.99 [0.74; 1.32]	0.89 [0.56; 1.40]	-0.10 [-0.58; 0.46]
Japan	2.59 [2.50; 2.68]	1.79 [1.70; 1.89]	-0.80 [-0.93; -0.67]†	3.7 [3.6; 3.9]	2.1 [2.0; 2.2]	0.96 [0.92; 1.00]	1.31 [1.21; 1.43]*	0.35 [0.25; 0.48]†
Jordan	2.42 [1.90; 3.06]	2.36 [1.51; 3.57]	-0.06 [-1.14; 1.27]	9.4 [7.0; 12.2]	4.0 [2.7; 5.8]	1.00 [0.79; 1.28]	1.07 [0.70; 1.65]	0.07 [-0.41; 0.68]
Kazakhstan	2.12 [2.02; 2.22]	2.04 [1.90; 2.19]	-0.08 [-0.25; 0.10]	9.7 [9.3; 10.1]	4.8 [4.5; 5.0]	1.11 [1.05; 1.18]*	1.24 [1.15; 1.34]*	0.13 [0.02; 0.25]†
Kenya	1.16 [0.95; 1.41]	1.57 [1.06; 2.26]	0.41 [-0.18; 1.15]	19.2 [10.0; 36.6]	12.2 [6.1; 24.0]	1.70 [0.87; 2.29]	1.35 [0.48; 2.18]	-0.35 [-1.40; 0.83]
Kiribati	1.82 [1.41; 2.33]	2.22 [1.34; 3.55]	0.39 [-0.65; 1.79]	21.4 [15.8; 28.2]	9.7 [6.4; 14.5]	0.98 [0.50; 1.45]	0.99 [0.55; 1.73]	0.01 [-0.58; 0.83]
Democratic People’s Republic of Korea	2.24 [1.73; 2.88]	2.15 [1.34; 3.37]	-0.09 [-1.17; 1.25]	11.5 [8.5; 15.1]	5.4 [3.6; 8.0]	1.00 [0.74; 1.34]	1.17 [0.73; 1.86]	0.17 [-0.40; 0.92]
Republic of Korea	2.32 [2.03; 2.64]	1.56 [1.31; 1.86]	-0.75 [-1.17; -0.33]†	3.5 [3.2; 3.8]	2.2 [2.0; 2.5]	1.07 [0.93; 1.22]	1.50 [1.25; 1.82]*	0.44 [0.13; 0.78]†
Kosovo	2.37 [1.81; 3.06]	2.53 [1.50; 4.12]	0.16 [-1.11; 1.88]	8.4 [6.2; 11.0]	3.3 [2.1; 5.1]	1.00 [0.74; 1.34]	1.01 [0.61; 1.66]	0.01 [-0.52; 0.72]
Kuwait	5.70 [4.82; 6.74]	2.76 [2.20; 3.41]	-2.94 [-4.12; -1.82]†	7.0 [5.9; 8.2]	2.6 [2.0; 3.2]	0.89 [0.75; 1.06]	0.91 [0.73; 1.14]	0.02 [-0.23; 0.29]
Kyrgyzstan¶§	1.90 [1.75; 2.05]	1.59 [1.46; 1.74]	-0.31 [-0.51; -0.10]†	9.1 [8.5; 9.7]	5.7 [5.3; 6.1]	1.31 [1.20; 1.42]*	1.59 [1.45; 1.75]*	0.28 [0.10; 0.47]†
Lao People’s Democratic Republic	1.29 [1.03; 1.61]	1.40 [0.90; 2.14]	0.11 [-0.51; 0.93]	13.6 [7.1; 26.5]	9.7 [4.9; 20.1]	0.75 [0.43; 1.71]	1.66 [0.73; 2.76]	0.90 [-0.39; 2.02]
Latvia¶	3.25 [2.96; 3.57]	2.43 [1.99; 2.94]	-0.83 [-1.37; -0.23]†	7.9 [6.9; 8.9]	3.2 [2.7; 3.9]	0.69 [0.62; 0.77]*	1.05 [0.86; 1.27]	0.35 [0.15; 0.59]†
Lebanon	11.95 [9.28; 15.39]	2.52 [1.51; 3.96]	-9.43 [-13.13; -6.34]†	7.6 [5.7; 9.8]	3.0 [1.9; 4.6]	1.00 [0.77; 1.30]	1.01 [0.63; 1.63]	0.02 [-0.48; 0.68]
Lesotho	1.21 [0.96; 1.51]	1.45 [1.00; 2.08]	0.24 [-0.33; 0.94]	31.4 [17.1; 56.4]	21.7 [12.4; 38.8]	0.87 [0.53; 1.81]	0.81 [0.39; 2.00]	-0.06 [-1.06; 1.17]
Liberia	4.60 [3.58; 5.98]	1.19 [0.85; 1.67]	-3.41 [-4.82; -2.24]†	30.2 [19.8; 46.9]	25.4 [16.2; 40.3]	0.91 [0.60; 1.72]	0.99 [0.52; 2.19]	0.08 [-0.86; 1.31]
Libya	2.42 [1.87; 3.10]	2.53 [1.52; 4.02]	0.12 [-1.14; 1.73]	8.5 [6.3; 10.9]	3.3 [2.1; 5.2]	1.00 [0.77; 1.30]	1.00 [0.62; 1.62]	0.00 [-0.50; 0.68]
Lithuania¶	3.33 [3.05; 3.62]	2.72 [2.29; 3.20]	-0.61 [-1.13; -0.05]†	7.6 [6.9; 8.5]	2.8 [2.4; 3.3]	0.71 [0.65; 0.78]*	0.94 [0.79; 1.11]	0.22 [0.06; 0.41]†
Luxembourg	2.59 [2.11; 3.15]	2.29 [1.61; 3.22]	-0.29 [-1.22; 0.75]	3.1 [2.3; 4.2]	1.4 [0.9; 2.0]	0.95 [0.77; 1.17]	1.03 [0.72; 1.47]	0.07 [-0.31; 0.56]
Macedonia	2.10 [1.80; 2.44]	1.96 [1.54; 2.48]	-0.14 [-0.69; 0.46]	4.1 [3.4; 5.0]	2.1 [1.7; 2.6]	1.19 [1.03; 1.39]*	1.22 [0.96; 1.55]	0.03 [-0.29; 0.39]
Madagascar	1.01 [0.85; 1.20]	1.31 [0.86; 1.97]	0.30 [-0.21; 0.98]	24.8 [11.4; 57.4]	19.0 [8.7; 45.4]	1.29 [0.81; 2.01]	1.16 [0.45; 2.47]	-0.13 [-1.20; 1.32]
Malawi	0.89 [0.77; 1.04]	1.65 [1.19; 2.27]	0.75 [0.27; 1.40]†	22.5 [13.9; 35.9]	13.7 [8.3; 22.6]	1.08 [0.78; 1.64]	1.21 [0.47; 1.89]	0.13 [-0.77; 0.88]
Malaysia	2.51 [2.24; 2.82]	2.43 [2.29; 2.57]	-0.08 [-0.42; 0.22]	7.7 [7.4; 8.1]	3.2 [3.0; 3.4]	0.96 [0.85; 1.09]	1.04 [0.98; 1.11]	0.08 [-0.06; 0.21]
Maldives¶	1.30 [1.08; 1.57]	1.94 [1.34; 2.75]	0.64 [-0.04; 1.49]	4.5 [3.3; 5.8]	2.3 [1.6; 3.3]	1.86 [1.51; 2.27]*	1.24 [0.87; 1.76]	-0.61 [-1.19; 0.03]
Mali	0.83 [0.70; 0.99]	1.24 [0.91; 1.68]	0.41 [0.04; 0.88]†	23.5 [15.3; 36.8]	18.9 [12.1; 30.4]	1.42 [0.91; 2.21]	1.44 [0.63; 2.38]	0.01 [-1.14; 1.10]
Malta	2.63 [2.06; 3.32]	2.65 [1.83; 3.74]	0.02 [-1.10; 1.32]	3.1 [2.2; 4.2]	1.2 [0.8; 1.7]	0.94 [0.75; 1.20]	0.90 [0.62; 1.30]	-0.05 [-0.43; 0.42]
Marshall Islands	2.23 [1.71; 2.88]	2.38 [1.42; 3.77]	0.15 [-1.04; 1.68]	16.7 [12.4; 21.8]	7.0 [4.5; 10.7]	1.00 [0.71; 1.33]	1.01 [0.60; 1.70]	0.02 [-0.52; 0.75]
Mauritania	1.75 [1.37; 2.24]	1.31 [0.96; 1.79]	-0.44 [-1.05; 0.19]	16.3 [10.4; 27.4]	12.4 [7.7; 21.1]	0.96 [0.46; 1.49]	1.68 [0.82; 2.49]	0.72 [-0.26; 1.71]
Mauritius¶	1.42 [1.25; 1.62]	2.40 [1.97; 2.92]	0.98 [0.50; 1.53]†	11.2 [9.9; 12.7]	4.7 [3.9; 5.6]	1.76 [1.55; 2.02]*	1.06 [0.86; 1.28]	-0.71 [-1.03; -0.39]†
Mexico§	2.53 [2.47; 2.59]	2.98 [2.67; 3.32]	0.45 [0.13; 0.80]†	18.0 [16.7; 19.5]	6.1 [5.4; 6.8]	0.96 [0.92; 0.99]*	0.80 [0.71; 0.91]*	-0.16 [-0.25; -0.05]†
Republic of Moldova¶	2.75 [2.52; 2.99]	2.52 [2.09; 3.01]	-0.23 [-0.72; 0.30]	9.9 [8.8; 11.1]	3.9 [3.3; 4.6]	0.86 [0.78; 0.95]*	1.00 [0.83; 1.21]	0.14 [-0.05; 0.36]
Monaco	2.54 [1.93; 3.27]	2.40 [1.42; 3.88]	-0.14 [-1.41; 1.51]	4.6 [3.4; 5.9]	1.9 [1.2; 3.0]	1.00 [0.77; 1.31]	1.02 [0.63; 1.68]	0.02 [-0.49; 0.72]
Mongolia¶	1.79 [1.49; 2.12]	2.18 [1.75; 2.71]	0.40 [-0.16; 1.01]	9.7 [8.2; 11.5]	4.5 [3.6; 5.5]	1.28 [1.03; 1.57]*	1.15 [0.92; 1.44]	-0.13 [-0.50; 0.25]
Montenegro	2.11 [1.74; 2.56]	2.36 [1.67; 3.28]	0.25 [-0.62; 1.25]	5.4 [4.3; 6.6]	2.3 [1.7; 3.1]	1.21 [0.99; 1.47]	1.05 [0.75; 1.47]	-0.16 [-0.56; 0.33]
Montserrat	2.46 [1.89; 3.18]	2.52 [1.51; 4.05]	0.05 [-1.21; 1.72]	6.9 [5.1; 8.9]	2.7 [1.8; 4.2]	1.00 [0.76; 1.31]	1.01 [0.62; 1.63]	0.01 [-0.51; 0.69]
Morocco¶	1.77 [1.45; 2.15]	2.27 [1.44; 3.43]	0.50 [-0.45; 1.73]	7.0 [3.3; 15.3]	3.1 [1.5; 7.0]	1.35 [1.08; 1.67]*	1.10 [0.67; 1.74]	-0.25 [-0.81; 0.46]
Mozambique	1.07 [0.88; 1.30]	1.15 [0.74; 1.77]	0.08 [-0.41; 0.75]	32.0 [15.5; 66.5]	27.9 [12.3; 61.6]	0.84 [0.60; 1.28]	1.00 [0.47; 2.66]	0.16 [-0.57; 1.85]
Myanmar	1.39 [1.02; 1.88]	2.14 [1.40; 3.27]	0.75 [-0.13; 1.98]	14.9 [8.0; 27.5]	7.0 [3.4; 13.9]	0.82 [0.45; 1.59]	1.51 [0.92; 2.34]	0.68 [-0.32; 1.61]
Namibia	1.41 [1.17; 1.68]	1.59 [1.05; 2.37]	0.19 [-0.43; 1.02]	26.1 [12.9; 51.6]	16.4 [8.2; 32.7]	0.90 [0.58; 1.45]	1.05 [0.37; 2.08]	0.14 [-0.79; 1.24]

¶: Sex ratio is outlying in 1990. §: Sex ratio is outlying in 2021. *: The ratio of estimated to expected female mortality is significantly different from one. †: Change is significantly different from zero.

Continued on next page

Table 21 – continued from previous page

	Sex ratio 10q15			Sex-specific 10q15 in 2021 (per 1,000)		Estimated/Expected female 10q15		
	1990	2021	Change 1990–2021	Male	Female	1990	2021	Change 1990–2021
Nauru	2.06 [1.59; 2.64]	2.40 [1.43; 3.78]	0.34 [-0.82; 1.82]	16.2 [11.9; 21.2]	6.8 [4.4; 10.3]	0.98 [0.60; 1.36]	1.01 [0.61; 1.70]	0.03 [-0.53; 0.81]
Nepal¶§	1.06 [0.87; 1.27]	1.43 [0.96; 2.10]	0.37 [-0.17; 1.09]	11.6 [6.5; 20.8]	8.1 [4.5; 14.8]	1.99 [1.38; 2.55]*	1.69 [1.07; 2.55]*	-0.30 [-1.17; 0.77]
Netherlands	2.15 [2.00; 2.30]	1.92 [1.73; 2.14]	-0.22 [-0.47; 0.04]	3.1 [2.9; 3.3]	1.6 [1.5; 1.8]	1.15 [1.07; 1.24]*	1.22 [1.07; 1.39]*	0.07 [-0.10; 0.25]
New Zealand¶	2.95 [2.71; 3.21]	1.85 [1.64; 2.09]	-1.10 [-1.43; -0.76]†	6.0 [5.6; 6.6]	3.3 [2.9; 3.6]	0.81 [0.74; 0.89]*	1.34 [1.18; 1.51]*	0.53 [0.35; 0.71]†
Nicaragua	2.21 [1.71; 2.88]	2.47 [1.50; 4.03]	0.26 [-0.97; 1.89]	12.6 [9.4; 16.6]	5.1 [3.3; 7.8]	0.98 [0.48; 1.42]	1.00 [0.61; 1.67]	0.02 [-0.56; 0.85]
Niger	0.92 [0.77; 1.12]	1.04 [0.70; 1.56]	0.11 [-0.29; 0.67]	27.0 [13.6; 54.8]	26.0 [12.2; 54.7]	1.04 [0.72; 1.62]	1.41 [0.58; 2.96]	0.37 [-0.69; 1.97]
Nigeria	1.00 [0.80; 1.25]	1.17 [0.89; 1.52]	0.17 [-0.22; 0.59]	19.5 [13.3; 28.8]	16.7 [11.1; 25.2]	0.96 [0.63; 1.68]	1.82 [0.97; 2.60]	0.86 [-0.22; 1.72]
Niue	2.41 [1.86; 3.10]	2.47 [1.47; 4.08]	0.05 [-1.19; 1.78]	18.2 [12.4; 25.4]	7.4 [5.1; 10.5]	1.00 [0.77; 1.31]	0.98 [0.55; 1.66]	-0.02 [-0.57; 0.71]
Norway	3.05 [2.76; 3.37]	2.23 [1.91; 2.60]	-0.82 [-1.26; -0.36]†	4.2 [3.8; 4.7]	1.9 [1.7; 2.2]	0.82 [0.74; 0.91]*	1.07 [0.91; 1.25]	0.25 [0.06; 0.44]†
Oman	2.42 [1.87; 3.06]	2.50 [1.50; 3.98]	0.09 [-1.16; 1.67]	8.0 [6.0; 10.3]	3.2 [2.0; 5.0]	1.01 [0.78; 1.30]	1.02 [0.63; 1.65]	0.01 [-0.50; 0.69]
Pakistan¶	1.22 [1.04; 1.44]	2.63 [1.76; 3.82]	1.41 [0.45; 2.64]†	16.1 [7.4; 34.5]	6.1 [2.9; 13.1]	1.62 [1.18; 2.04]*	0.86 [0.33; 1.33]	-0.76 [-1.43; -0.08]†
Palau	2.65 [2.08; 3.36]	0.98 [0.76; 1.26]	-1.68 [-2.41; -1.02]†	27.9 [17.2; 46.5]	28.6 [16.6; 49.0]	0.77 [0.45; 1.05]	1.25 [0.67; 2.69]	0.48 [-0.17; 1.97]
Panama	2.43 [2.08; 2.84]	2.75 [2.32; 3.24]	0.32 [-0.28; 0.93]	13.8 [12.9; 14.8]	5.0 [4.4; 5.8]	1.00 [0.85; 1.17]	0.91 [0.76; 1.08]	-0.09 [-0.32; 0.14]
Papua New Guinea§	1.86 [1.44; 2.39]	1.29 [0.90; 1.85]	-0.56 [-1.23; 0.15]	16.3 [12.2; 21.5]	12.7 [9.2; 17.3]	1.00 [0.56; 1.44]	1.86 [1.24; 2.72]*	0.85 [0.10; 1.80]†
Paraguay	2.05 [1.59; 2.60]	2.41 [1.45; 3.84]	0.36 [-0.79; 1.88]	14.7 [10.9; 19.2]	6.1 [3.9; 9.2]	1.06 [0.72; 1.42]	1.03 [0.63; 1.70]	-0.03 [-0.60; 0.73]
Peru	1.78 [1.56; 2.03]	2.70 [1.74; 3.98]	0.92 [-0.09; 2.24]	9.3 [4.2; 20.0]	3.5 [1.7; 7.4]	1.16 [0.91; 1.39]	0.93 [0.53; 1.47]	-0.22 [-0.71; 0.38]
Philippines¶	1.82 [1.57; 2.11]	2.23 [1.78; 2.76]	0.41 [-0.12; 0.99]	12.8 [11.1; 14.6]	5.7 [4.7; 6.9]	1.30 [1.10; 1.53]*	1.12 [0.90; 1.41]	-0.17 [-0.49; 0.17]
Poland¶	3.60 [3.43; 3.77]	2.77 [2.58; 2.97]	-0.83 [-1.09; -0.57]†	7.1 [6.8; 7.4]	2.6 [2.4; 2.7]	0.70 [0.66; 0.73]*	0.92 [0.85; 0.99]*	0.22 [0.14; 0.30]†
Portugal¶	3.52 [3.30; 3.75]	2.28 [2.00; 2.60]	-1.23 [-1.60; -0.84]†	3.9 [3.6; 4.2]	1.7 [1.5; 1.9]	0.67 [0.63; 0.73]*	1.03 [0.90; 1.20]	0.36 [0.21; 0.53]†
Qatar	2.53 [2.00; 3.13]	2.58 [1.91; 3.41]	0.05 [-0.90; 1.06]	4.3 [3.6; 5.1]	1.7 [1.2; 2.2]	0.97 [0.77; 1.22]	0.93 [0.69; 1.24]	-0.04 [-0.40; 0.34]
Romania	2.24 [2.13; 2.35]	2.60 [2.38; 2.84]	0.37 [0.12; 0.63]†	7.0 [6.7; 7.4]	2.7 [2.5; 2.9]	1.12 [1.06; 1.18]*	0.97 [0.88; 1.06]	-0.15 [-0.26; -0.04]†
Russian Federation¶	3.12 [3.05; 3.19]	2.53 [2.27; 2.82]	-0.59 [-0.86; -0.29]†	10.9 [10.0; 11.9]	4.3 [3.9; 4.8]	0.73 [0.70; 0.76]*	1.00 [0.89; 1.12]	0.27 [0.16; 0.40]†
Rwanda¶	1.20 [1.00; 1.43]	1.70 [1.22; 2.38]	0.50 [-0.04; 1.23]	11.6 [6.5; 20.8]	6.8 [3.6; 12.7]	0.58 [0.48; 0.73]*	1.43 [0.94; 2.02]	0.84 [0.32; 1.45]†
Saint Kitts and Nevis	2.39 [1.85; 3.06]	2.71 [1.68; 4.23]	0.32 [-0.94; 1.99]	22.9 [13.1; 38.6]	8.5 [4.5; 15.9]	1.03 [0.78; 1.35]	0.71 [0.26; 1.32]	-0.32 [-0.88; 0.35]
Saint Lucia	2.29 [1.82; 2.87]	2.30 [1.58; 3.30]	0.01 [-0.96; 1.14]	12.4 [8.6; 17.4]	5.4 [3.4; 8.4]	1.07 [0.83; 1.36]	1.07 [0.72; 1.58]	0.00 [-0.46; 0.58]
Samoa	1.41 [1.02; 1.93]	1.65 [1.10; 2.45]	0.24 [-0.71; 1.15]	9.0 [5.1; 15.8]	5.5 [3.1; 10.1]	0.74 [0.42; 1.86]	1.49 [0.97; 2.25]	0.75 [-0.48; 1.56]
San Marino	2.49 [1.92; 3.23]	2.36 [1.39; 3.81]	-0.14 [-1.39; 1.44]	3.2 [2.3; 4.1]	1.3 [0.9; 2.0]	1.00 [0.76; 1.30]	1.02 [0.62; 1.67]	0.02 [-0.50; 0.72]
Sao Tome and Principe	1.54 [1.19; 1.99]	1.91 [1.18; 3.01]	0.36 [-0.55; 1.53]	23.6 [10.9; 49.5]	12.4 [5.3; 28.5]	0.63 [0.37; 1.70]	0.91 [0.31; 1.86]	0.28 [-1.04; 1.23]
Saudi Arabia	2.13 [1.67; 2.69]	3.06 [2.09; 4.36]	0.93 [-0.24; 2.32]	14.0 [8.8; 23.3]	4.6 [2.7; 8.3]	0.85 [0.30; 1.31]	0.76 [0.45; 1.17]	-0.09 [-0.65; 0.60]
Senegal	1.18 [0.97; 1.43]	1.54 [1.10; 2.11]	0.36 [-0.16; 0.97]	14.1 [8.8; 22.8]	9.2 [5.6; 15.3]	1.41 [0.79; 2.06]	1.48 [0.95; 2.16]	0.07 [-0.80; 1.02]
Serbia	2.50 [2.20; 2.85]	2.63 [2.18; 3.13]	0.12 [-0.45; 0.72]	5.8 [5.2; 6.4]	2.2 [1.9; 2.6]	1.01 [0.88; 1.15]	0.94 [0.79; 1.13]	-0.07 [-0.28; 0.16]
Seychelles	2.53 [1.96; 3.22]	3.36 [2.31; 4.73]	0.84 [-0.51; 2.37]	11.9 [7.5; 19.6]	3.5 [2.1; 6.5]	1.00 [0.77; 1.29]	0.70 [0.46; 1.06]	-0.30 [-0.69; 0.14]
Sierra Leone	0.78 [0.59; 1.04]	1.19 [0.89; 1.59]	0.42 [-0.01; 0.86]	36.5 [21.5; 56.4]	30.6 [18.9; 46.2]	1.01 [0.71; 1.67]	0.80 [0.49; 1.69]	-0.21 [-0.96; 0.68]
Singapore	2.20 [1.90; 2.55]	2.01 [1.58; 2.54]	-0.19 [-0.74; 0.42]	2.6 [2.2; 3.1]	1.3 [1.1; 1.6]	1.15 [1.00; 1.33]	1.16 [0.91; 1.49]	0.01 [-0.30; 0.37]
Slovakia	2.78 [2.55; 3.03]	2.39 [2.06; 2.76]	-0.40 [-0.81; 0.04]	5.8 [5.3; 6.2]	2.4 [2.1; 2.7]	0.91 [0.83; 0.99]*	1.03 [0.89; 1.19]	0.12 [-0.04; 0.30]
Slovenia	3.02 [2.69; 3.39]	2.39 [1.80; 3.12]	-0.63 [-1.34; 0.16]	3.7 [3.2; 4.3]	1.5 [1.2; 2.0]	0.83 [0.74; 0.94]*	0.99 [0.76; 1.31]	0.16 [-0.10; 0.48]
Solomon Islands	2.29 [1.75; 2.92]	2.05 [1.29; 3.17]	-0.23 [-1.25; 1.05]	12.6 [9.4; 16.6]	6.1 [4.1; 9.0]	1.00 [0.75; 1.34]	1.22 [0.77; 1.93]	0.22 [-0.36; 0.96]
Somalia	0.76 [0.59; 1.00]	0.92 [0.56; 1.54]	0.16 [-0.27; 0.79]	38.8 [26.6; 54.4]	42.0 [29.0; 58.1]	1.00 [0.71; 1.74]	1.03 [0.49; 2.85]	0.02 [-0.76; 1.72]
South Africa	2.09 [1.69; 2.58]	1.60 [1.35; 1.88]	-0.49 [-1.04; 0.00]	24.5 [21.4; 28.0]	15.3 [13.2; 17.8]	0.72 [0.37; 1.19]	1.28 [0.82; 1.62]	0.56 [-0.10; 1.07]
South Sudan	0.69 [0.53; 0.92]	0.97 [0.59; 1.62]	0.28 [-0.17; 0.94]	36.8 [25.2; 50.8]	37.9 [25.7; 52.9]	1.04 [0.78; 1.54]	1.04 [0.48; 2.91]	0.00 [-0.70; 1.83]
Spain	3.13 [3.00; 3.27]	2.26 [1.97; 2.58]	-0.87 [-1.19; -0.52]†	2.7 [2.5; 3.0]	1.2 [1.1; 1.4]	0.81 [0.77; 0.85]*	1.05 [0.89; 1.23]	0.24 [0.08; 0.43]†
Sri Lanka¶	2.22 [1.95; 2.52]	2.25 [1.63; 3.08]	0.03 [-0.67; 0.89]	5.6 [3.5; 8.8]	2.5 [1.5; 4.0]	0.68 [0.49; 0.90]*	1.09 [0.80; 1.50]	0.42 [0.05; 0.85]†
Saint Vincent and the Grenadines	2.49 [1.97; 3.11]	2.25 [1.47; 3.33]	-0.25 [-1.29; 1.00]	19.0 [12.3; 28.4]	8.5 [5.2; 13.7]	1.00 [0.79; 1.27]	0.97 [0.48; 1.58]	-0.03 [-0.59; 0.63]
State of Palestine	2.85 [2.20; 3.63]	2.73 [1.64; 4.32]	-0.12 [-1.53; 1.60]	11.0 [8.1; 14.4]	4.0 [2.6; 6.1]	1.00 [0.77; 1.30]	1.01 [0.63; 1.64]	0.01 [-0.48; 0.69]
Sudan	0.89 [0.70; 1.15]	1.55 [0.93; 2.53]	0.65 [-0.05; 1.66]	31.0 [21.9; 41.6]	20.0 [13.2; 29.4]	0.95 [0.65; 1.80]	0.92 [0.37; 2.16]	-0.02 [-0.86; 1.15]
Suriname¶§	1.80 [1.51; 2.14]	1.33 [1.10; 1.60]	-0.47 [-0.87; -0.07]†	13.0 [10.6; 15.8]	9.8 [7.9; 12.1]	1.31 [1.08; 1.58]*	1.86 [1.52; 2.27]*	0.55 [0.11; 1.01]†

¶: Sex ratio is outlying in 1990. §: Sex ratio is outlying in 2021. *: The ratio of estimated to expected female mortality is significantly different from one. †: Change is significantly different from zero.

Continued on next page

Table 21 – continued from previous page

	**Sex ratio 10q15**			**Sex-specific 10q15 in 2021 (per 1,000)**		**Estimated/Expected female 10q15**		
	**1990**	**2021**	**Change 1990–2021**	**Male**	**Female**	**1990**	**2021**	**Change 1990–2021**
Eswatini§	1.13<br>[0.89; 1.44]	0.90<br>[0.58; 1.43]	-0.23<br>[-0.71; 0.38]	22.4<br>[15.7; 31.1]	24.8<br>[17.5; 34.0]	1.36<br>[0.68; 2.24]	2.30<br>[1.08; 3.94]*	0.95<br>[-0.44; 2.69]
Sweden	2.49<br>[2.29; 2.71]	2.22<br>[1.96; 2.52]	-0.27<br>[-0.61; 0.09]	4.5<br>[4.2; 4.8]	2.0<br>[1.8; 2.3]	1.01<br>[0.93; 1.10]	1.08<br>[0.95; 1.23]	0.07<br>[-0.09; 0.24]
Switzerland	2.93<br>[2.70; 3.18]	2.43<br>[2.10; 2.81]	-0.50<br>[-0.92; -0.06]†	3.8<br>[3.5; 4.1]	1.6<br>[1.4; 1.8]	0.86<br>[0.79; 0.94]*	0.97<br>[0.83; 1.14]	0.11<br>[-0.05; 0.30]
Syria	1.63<br>[1.29; 2.03]	3.24<br>[2.19; 4.58]	1.61<br>[0.42; 3.15]†	13.6<br>[7.1; 32.7]	4.2<br>[2.1; 10.8]	1.26<br>[0.74; 1.68]	1.17<br>[0.76; 1.72]	-0.08<br>[-0.71; 0.67]
Tajikistan¶§	1.38<br>[1.28; 1.48]	1.50<br>[1.36; 1.64]	0.12<br>[-0.05; 0.30]	5.1<br>[4.8; 5.4]	3.4<br>[3.2; 3.7]	1.80<br>[1.66; 1.95]*	1.61<br>[1.46; 1.79]*	-0.19<br>[-0.40; 0.04]
Tanzania	1.12<br>[0.94; 1.33]	1.36<br>[0.96; 1.91]	0.25<br>[-0.22; 0.83]	20.0<br>[10.5; 40.9]	14.7<br>[8.0; 29.4]	1.32<br>[0.79; 1.96]	1.51<br>[0.53; 2.39]	0.18<br>[-0.99; 1.24]
Thailand¶§	2.88<br>[2.47; 3.36]	3.30<br>[2.63; 4.07]	0.42<br>[-0.43; 1.31]	18.4<br>[16.2; 20.9]	5.6<br>[4.6; 6.9]	0.74<br>[0.62; 0.88]*	0.69<br>[0.55; 0.87]*	-0.05<br>[-0.25; 0.17]
Timor Leste	1.84<br>[1.44; 2.33]	0.66<br>[0.48; 0.93]	-1.18<br>[-1.72; -0.68]†	24.7<br>[12.7; 56.5]	37.4<br>[19.5; 86.0]	0.69<br>[0.35; 1.40]	2.14<br>[0.94; 4.40]	1.45<br>[-0.06; 3.70]
Togo	1.23<br>[1.01; 1.49]	1.33<br>[0.91; 1.97]	0.10<br>[-0.42; 0.81]	19.5<br>[10.2; 38.0]	14.7<br>[7.0; 30.1]	1.29<br>[0.75; 1.91]	1.51<br>[0.58; 2.51]	0.23<br>[-0.91; 1.40]
Tonga	2.64<br>[2.02; 3.42]	2.76<br>[1.75; 4.15]	0.12<br>[-1.22; 1.67]	12.3<br>[8.3; 17.7]	4.5<br>[2.9; 7.2]	0.96<br>[0.73; 1.25]	0.91<br>[0.58; 1.43]	-0.05<br>[-0.51; 0.55]
Trinidad and Tobago¶§	1.96<br>[1.74; 2.20]	3.20<br>[2.46; 4.09]	1.24<br>[0.47; 2.14]†	21.0<br>[15.1; 29.7]	6.6<br>[4.6; 9.7]	1.27<br>[1.13; 1.44]*	0.69<br>[0.32; 0.94]*	-0.58<br>[-0.97; -0.29]†
Tunisia	2.11<br>[1.70; 2.60]	2.72<br>[2.06; 3.55]	0.61<br>[-0.23; 1.54]	11.0<br>[8.1; 14.6]	4.0<br>[2.9; 5.6]	1.18<br>[0.95; 1.47]	0.93<br>[0.70; 1.23]	-0.25<br>[-0.62; 0.13]
Turkey	1.97<br>[1.50; 2.68]	2.46<br>[2.11; 2.86]	0.49<br>[-0.28; 1.15]	5.4<br>[4.7; 6.1]	2.2<br>[1.9; 2.6]	0.91<br>[0.40; 1.37]	0.99<br>[0.85; 1.15]	0.08<br>[-0.41; 0.61]
Turkmenistan¶§	1.54<br>[1.35; 1.76]	1.35<br>[1.01; 1.79]	-0.19<br>[-0.60; 0.29]	11.6<br>[7.5; 17.6]	8.6<br>[5.6; 13.0]	1.58<br>[1.37; 1.82]*	1.84<br>[1.35; 2.49]*	0.26<br>[-0.30; 0.96]
Turks and Caicos Islands	2.51<br>[1.94; 3.20]	2.47<br>[1.46; 3.97]	-0.05<br>[-1.31; 1.57]	7.5<br>[5.5; 9.9]	3.1<br>[2.0; 4.6]	0.99<br>[0.77; 1.28]	1.03<br>[0.64; 1.69]	0.04<br>[-0.46; 0.75]
Tuvalu	2.28<br>[1.79; 2.90]	2.61<br>[1.60; 4.13]	0.33<br>[-0.91; 1.97]	14.4<br>[10.8; 18.8]	5.5<br>[3.6; 8.4]	0.95<br>[0.66; 1.26]	0.96<br>[0.58; 1.56]	0.01<br>[-0.49; 0.68]
Uganda	0.87<br>[0.75; 1.01]	1.39<br>[1.02; 1.89]	0.52<br>[0.12; 1.02]†	31.5<br>[18.1; 56.8]	22.7<br>[13.2; 40.6]	0.94<br>[0.74; 1.28]	0.81<br>[0.43; 1.75]	-0.13<br>[-0.64; 0.81]
Ukraine¶	2.97<br>[2.86; 3.08]	2.69<br>[2.52; 2.87]	-0.28<br>[-0.48; -0.08]†	9.2<br>[8.8; 9.6]	3.4<br>[3.2; 3.6]	0.81<br>[0.77; 0.85]*	0.95<br>[0.88; 1.01]	0.13<br>[0.06; 0.21]†
United Arab Emirates	2.48<br>[1.91; 3.15]	2.46<br>[1.48; 3.85]	-0.02<br>[-1.23; 1.54]	6.2<br>[4.7; 8.1]	2.5<br>[1.6; 4.1]	1.01<br>[0.78; 1.31]	1.02<br>[0.64; 1.65]	0.01<br>[-0.49; 0.68]
United Kingdom	2.74<br>[2.62; 2.87]	2.13<br>[1.81; 2.49]	-0.61<br>[-0.96; -0.23]†	4.2<br>[3.7; 4.8]	2.0<br>[1.7; 2.3]	0.92<br>[0.87; 0.97]*	1.12<br>[0.95; 1.32]	0.20<br>[0.03; 0.41]†
United States of America¶	2.98<br>[2.93; 3.04]	2.59<br>[2.23; 2.99]	-0.39<br>[-0.76; 0.01]	12.1<br>[11.1; 13.4]	4.7<br>[4.1; 5.4]	0.82<br>[0.79; 0.84]*	0.98<br>[0.85; 1.14]	0.16<br>[0.02; 0.32]†
Uruguay§	2.18<br>[1.97; 2.40]	3.34<br>[2.84; 3.91]	1.16<br>[0.62; 1.77]†	13.9<br>[12.5; 15.5]	4.2<br>[3.6; 4.9]	1.14<br>[1.03; 1.27]*	0.75<br>[0.63; 0.88]*	-0.39<br>[-0.57; -0.22]†
Uzbekistan¶§	1.57<br>[1.38; 1.80]	1.21<br>[1.04; 1.40]	-0.37<br>[-0.65; -0.09]†	7.9<br>[7.3; 8.5]	6.6<br>[6.0; 7.2]	1.59<br>[1.38; 1.81]*	2.07<br>[1.78; 2.39]*	0.48<br>[0.11; 0.89]†
Vanuatu	2.33<br>[1.79; 3.00]	2.22<br>[1.39; 3.42]	-0.11<br>[-1.21; 1.23]	14.6<br>[10.8; 19.0]	6.6<br>[4.4; 9.6]	1.00<br>[0.74; 1.33]	1.11<br>[0.70; 1.78]	0.12<br>[-0.42; 0.82]
Venezuela (Bolivarian Republic of)¶§	2.76<br>[2.40; 3.15]	5.00<br>[3.68; 6.68]	2.24<br>[0.85; 3.96]†	44.2<br>[32.1; 60.2]	8.8<br>[6.2; 12.9]	0.86<br>[0.74; 0.99]*	0.17<br>[0.11; 0.28]*	-0.68<br>[-0.83; -0.52]†
Vietnam	2.11<br>[1.67; 2.65]	2.57<br>[1.85; 3.53]	0.46<br>[-0.48; 1.53]	8.8<br>[6.7; 11.5]	3.4<br>[2.4; 4.9]	1.09<br>[0.78; 1.42]	0.97<br>[0.70; 1.35]	-0.11<br>[-0.56; 0.39]
Yemen	2.18<br>[1.69; 2.76]	4.26<br>[2.60; 6.86]	2.08<br>[0.31; 4.73]†	34.5<br>[25.6; 45.3]	8.1<br>[5.3; 12.3]	0.99<br>[0.69; 1.33]	1.01<br>[0.59; 1.66]	0.02<br>[-0.50; 0.74]
Zambia	0.78<br>[0.67; 0.89]	1.59<br>[1.16; 2.18]	0.81<br>[0.36; 1.42]†	25.1<br>[15.9; 39.0]	15.8<br>[9.4; 25.8]	1.09<br>[0.86; 1.52]	1.06<br>[0.47; 1.80]	-0.03<br>[-0.75; 0.75]
Zimbabwe	1.22<br>[1.02; 1.45]	1.29<br>[0.91; 1.82]	0.07<br>[-0.38; 0.63]	26.9<br>[16.1; 44.7]	20.9<br>[11.9; 35.3]	1.71<br>[0.92; 2.19]	1.24<br>[0.52; 2.30]	-0.47<br>[-1.38; 0.83]

Table 22: **Sex ratio data sources for age group 0–1, by country.** For each country, the total number of observations and the most recent reference year are shown after the country name. For each country-specific data series, the number of observations and the most recent reference year within that series is shown before each data series name. The source type that each data series falls in is shown in parentheses after each data series name.

Country	# obs.	Most recent obs. year	Data series name [source type]
Afghanistan	18	2015.5	
	8	2008.5	2010 Afghanistan Mortality Survey [DHS Direct]
	5	2012.5	2015 Demographic and Health Survey [DHS Direct]
	5	2015.5	2018 Afghanistan Health Survey [Others Direct]
Albania	26	2021.5	
	5	1999.5	2002 Reproductive Health Survey [Others Direct]
	5	2005.5	2008-2009 Demographic and Health Survey [DHS Direct]
	16	2021.5	WHO Vital Registration Data 2022 version [VR]
Algeria	39	2020.5	
	1	1976.5	1986 Enquete nationale sur la fecondite [Others Direct]
	5	1989.5	1992 Pan Arab Project for Child Development Maternal and Child Health Survey [Others Direct]
	1	1993	1995 Enquete nationale sur les objectifs [Others Direct]
	5	2000	2002 Pan Arab Project for Family Health Family Health Survey [Others Direct]
	22	2020.5	Vital Registration from Demographie Algerienne [VR]
	5	1986.5	WHO Vital Registration Data 2022 version [VR]
Andorra	58	2014.5	
	11	2014.5	WHO Vital Registration Data 2019 version [VR]
	7	2014.5	WHO Vital Registration Data 2020 version [VR]
	19	2014.5	WHO Vital Registration Data 2021 version [VR]
	21	2014.5	WHO Vital Registration Data 2022 version [VR]
Angola	5	2012.5	
	5	2012.5	2015-2016 Inquerito de Indicadores Multiplos e de Saude [DHS Direct]
Anguilla	16	2016.5	
	16	2016.5	2019 UNPD Vital Registration Data 2019 version [VR]
Antigua and Barbuda	58	2017.5	
	11	1960.5	UNPD Demographic Yearbook Data 2020 version [VR]
	47	2017.5	WHO Vital Registration Data 2022 version [VR]
Argentina	45	2020.5	
	45	2020.5	2021 WHO Vital Registration Data 2022 version [VR]
Armenia	15	2007.5	
	5	1997.5	2000 Demographic and Health Survey [DHS Direct]
	5	2002.5	2005 Demographic and Health Survey [DHS Direct]
	5	2007.5	2010 Demographic and Health Survey [DHS Direct]
Australia	71	2020.5	
	71	2020.5	2021 WHO Vital Registration Data 2022 version [VR]
Austria	66	2020.5	
	66	2020.5	2021 WHO Vital Registration Data 2022 version [VR]
Azerbaijan	11	2008.5	
	1	1996	2001 Reproductive Health Survey [Others Direct]

Continued on next page

Table 22 – continued from previous page

Country	# obs.	Most recent obs. year	Data series name [source type]
	5	2003.5	2006 Demographic and Health Survey [DHS Direct]
	5	2008.5	2011 Azerbaijan National Demographic and Health Survey [Other DHS Direct]
Bahamas	48	2020.5	
	48	2020.5	2021 WHO Vital Registration Data 2022 version [VR]
Bahrain	42	2019.5	
	1	1984	1989 Child Health Survey [Others Direct]
	1	1990.5	1995 Gulf Family Health Survey [Others Direct]
	5	2019.5	UNPD Vital Registration Data 2019 version [VR]
	35	2014.5	WHO Vital Registration Data 2022 version [VR]
Bangladesh	94	2020.5	
	5	1973	1975-1976 World Fertility Survey [Other DHS Direct]
	1	1981	1988-1989 Fertility Survey [Others Direct]
	5	1991	1993-1994 Demographic and Health Survey [DHS Direct]
	5	1994	1996-1997 Demographic and Health Survey [DHS Direct]
	5	1996.5	1999-2000 Demographic and Health Survey [DHS Direct]
	1	1999	2001 Maternal Health Services and Maternal Mortality Survey [Other DHS Direct]
	5	2001.5	2004 Demographic and Health Survey [DHS Direct]
	5	2004.5	2007 Demographic and Health Survey [DHS Direct]
	1	2010.7	2011 Census [Others Direct]
	5	2008.5	2011 Demographic and Health Survey [DHS Direct]
	1	2008.1	2012-2013 Multiple Indicator Cluster Survey [MICS Direct]
	5	2011.5	2014 Demographic and Health Survey [DHS Direct]
	5	2014.5	2017-2018 Demographic and Health Survey [DHS Direct]
	5	2016.5	2019 Multiple Indicator Cluster Survey [MICS Direct]
	10	2020.5	Sample Vital Statistics [VR]
	30	2010.5	SVital Registration from Report on Sample Vital Registration System 2010 [VR]
Barbados	59	2013.5	
	59	2013.5	2021 WHO Vital Registration Data 2022 version [VR]
Belarus	39	2019.5	
	39	2019.5	2021 WHO Vital Registration Data 2022 version [VR]
Belgium	70	2019.5	
	4	1953.5	UNPD Demographic Yearbook Data 2020 version [VR]
	66	2019.5	WHO Vital Registration Data 2022 version [VR]
Belize	23	2018.5	
	5	1988.5	1991 Family Health Survey [Others Direct]
	18	2018.5	WHO Vital Registration Data 2022 version [VR]
Benin	33	2015	
	5	1979	1981-1982 World Fertility Survey [Other DHS Direct]
	5	1994	1996 Demographic and Health Survey [DHS Direct]
	5	1998.5	2001 Demographic and Health Survey [DHS Direct]
	8	2004.5	2006 Demographic and Health Survey [DHS Direct]
	5	2011.5	2014 Multiple Indicator Cluster Survey [MICS Direct]

Continued on next page

Table 22 – continued from previous page

Country	# obs.	Most recent obs. year	Data series name [source type]
	5	2015	2017-2018 Demographic and Health Survey [DHS Direct]
Bhutan	3	1999.7	
	1	1983.5	1984 Demographic Sample Survey [Others Direct]
	1	1993.5	1994 National Health Survey [Others Direct]
	1	1999.7	2000 National Health Survey [Others Direct]
Bolivia (Plurinational State of)	30	2013.5	
	5	1986.5	1989 Demographic and Health Survey [DHS Direct]
	5	1990.5	1993-1994 Demographic and Health Survey [DHS Direct]
	5	1995.5	1998 Demographic and Health Survey [DHS Direct]
	5	2000.5	2003 Demographic and Health Survey [DHS Direct]
	5	2005.5	2008 Demographic and Health Survey [DHS Direct]
	5	2013.5	2016 Demographic and Health Survey [Other DHS Direct]
Bosnia and Herzegovina	25	2016.5	
	25	2016.5	2021 WHO Vital Registration Data 2022 version [VR]
Botswana	11	2005.4	
	5	1985.5	1988 Family Health Survey [DHS Direct]
	6	2005.4	2007 Family Health Survey [Others Direct]
Brazil	10	1993.5	
	5	1983.5	1986 Demographic and Health Survey [DHS Direct]
	5	1993.5	1996 Demographic and Health Survey [DHS Direct]
British Virgin Islands	40	2008.5	
	40	2008.5	2019 UNPD Vital Registration Data 2019 version [VR]
Brunei	63	2019.5	
	25	1978.5	UNPD Demographic Yearbook Data 2020 version [VR]
	38	2019.5	WHO Vital Registration Data 2022 version [VR]
Bulgaria	58	2021.5	
	58	2021.5	2021 WHO Vital Registration Data 2022 version [VR]
Burkina Faso	23	2008.5	
	5	1989.5	1993 Demographic and Health Survey [DHS Direct]
	6	1996.5	1998-1999 Demographic and Health Survey [DHS Direct]
	5	2000.5	2003 Demographic and Health Survey [DHS Direct]
	7	2008.5	2010 Demographic and Health Survey [DHS Direct]
Burundi	15	2013.5	
	1	1970.5	1970 Demographic Survey [Others Direct]
	5	1984.5	1987 Demographic and Health Survey [DHS Direct]
	5	2007.5	2010-2011 Demographic and Health Survey [DHS Direct]
	4	2013.5	2016-2017 Demographic and Health Survey [DHS Direct]
Cambodia	22	2011.5	
	7	1998.5	2000 Demographic and Health Survey [DHS Direct]
	5	2002.5	2005 Demographic and Health Survey [DHS Direct]
	5	2007.5	2010 Demographic and Health Survey [DHS Direct]

Continued on next page

Table 22 – continued from previous page

Country	# obs.	Most recent obs. year	Data series name [source type]
	5	2011.5	2014 Demographic and Health Survey [DHS Direct]
Cameroon	31	2015.5	
	5	1975.5	1978 World Fertility Survey [Other DHS Direct]
	5	1988.5	1991 Demographic and Health Survey [DHS Direct]
	5	1995.5	1998 Demographic and Health Survey [DHS Direct]
	6	2002	2004 Demographic and Health Survey [DHS Direct]
	5	2008.5	2011 Demographic and Health Survey [DHS Direct]
	5	2015.5	2018 Demographic and Health Survey [DHS Direct]
Canada	71	2020.5	
	71	2020.5	2021 WHO Vital Registration Data 2022 version [VR]
Cape Verde	32	2016.5	
	5	1995.5	1998 Demographic and Reproductive Health Survey [Others Direct]
	1	2002.2	2005 Demographic and Health Survey [DHS Direct]
	26	2016.5	WHO Vital Registration Data 2022 version [VR]
Central African Republic	10	2015.5	
	5	1991.5	1994-1995 Demographic and Health Survey [DHS Direct]
	5	2015.5	2018-2019 Multiple Indicator Cluster Survey [MICS Direct]
Chad	16	2011.5	
	6	1994.5	1996-1997 Demographic and Health Survey [DHS Direct]
	5	2001.5	2004 Demographic and Health Survey [DHS Direct]
	5	2011.5	2014-2015 Demographic and Health Survey [DHS Direct]
Chile	56	2015.5	
	56	2015.5	2021 WHO Vital Registration Data 2022 version [VR]
China	5	1986	
	5	1986	1988 National Survey on Fertility and Birth Control [Others Direct]
Colombia	35	2012.5	
	5	1983.5	1986 Demographic and Health Survey [DHS Direct]
	5	1987.5	1990 Demographic and Health Survey [DHS Direct]
	5	1992.5	1995 Demographic and Health Survey [DHS Direct]
	5	1997.5	2000 Demographic and Health Survey [DHS Direct]
	5	2001.5	2005 Demographic and Health Survey [DHS Direct]
	5	2006.5	2010 Demographic and Health Survey [DHS Direct]
	5	2012.5	2015 Demographic and Health Survey [DHS Direct]
Comoros	6	2017.5	
	5	1993.5	1996 Demographic and Health Survey [DHS Direct]
	1	2017.5	2017 Census [Others Direct]
Congo	10	2008.5	
	5	2002.5	2005 Demographic and Health Survey [DHS Direct]
	5	2008.5	2011-2012 Demographic and Health Survey [DHS Direct]
Democratic Republic of the Congo	10	2010.5	
	5	2004.5	2007 Demographic and Health Survey [DHS Direct]
	5	2010.5	2013-2014 Demographic and Health Survey [DHS Direct]
Cook Islands	22	2009.5	

Continued on next page

Table 22 – continued from previous page

Country	# obs.	Most recent obs. year	Data series name [source type]
	22	2009.5	2021 WHO Vital Registration Data 2022 version [VR]
Costa Rica	71	2020.5	
	6	1955.5	UNPD Demographic Yearbook Data 2020 version [VR]
	65	2020.5	WHO Vital Registration Data 2022 version [VR]
Cote d'Ivoire	33	2013.5	
	1	1978.5	1978 Demographic Survey Repeated Passages [Others Direct]
	6	1978.5	1980-1981 World Fertility Survey [Other DHS Direct]
	6	1992	1994 Demographic and Health Survey [DHS Direct]
	5	1995.5	1998-1999 Demographic and Health Survey [DHS Direct]
	5	2002.5	2005 AIDS Indicator Survey [Other DHS Direct]
	5	2008.5	2011-2012 Demographic and Health Survey [DHS Direct]
	5	2013.5	2016 Multiple Indicator Cluster Survey [MICS Direct]
Croatia	39	2021.5	
	39	2021.5	2021 WHO Vital Registration Data 2022 version [VR]
Cuba	55	2020.5	
	55	2020.5	2021 WHO Vital Registration Data 2022 version [VR]
Cyprus	41	2020.5	
	41	2020.5	2021 WHO Vital Registration Data 2022 version [VR]
Czech Republic	39	2021.5	
	39	2021.5	2021 WHO Vital Registration Data 2022 version [VR]
Denmark	71	2021.5	
	71	2021.5	2021 WHO Vital Registration Data 2022 version [VR]
Djibouti	6	2010	
	5	1999.5	2002 Pan Arab Project for Family Health Family Health Survey [Others Direct]
	1	2010	2012 Pan Arab Project for Family Health Family Health Survey [Others Direct]
Dominica	49	2015.5	
	49	2015.5	2021 WHO Vital Registration Data 2022 version [VR]
Dominican Republic	45	2016.5	
	5	1972.5	1975 World Fertility Survey [Other DHS Direct]
	5	1983.5	1986 Demographic and Health Survey [DHS Direct]
	5	1988.5	1991 Demographic and Health Survey [DHS Direct]
	5	1993.5	1996 Demographic and Health Survey [DHS Direct]
	5	1999.5	2002 Demographic and Health Survey [DHS Direct]
	5	2004.5	2007 Demographic and Health Survey [DHS Direct]
	5	2010.5	2013 Demographic and Health Survey [DHS Direct]
	5	2011.5	2014 Multiple Indicator Cluster Survey [MICS Direct]
	5	2016.5	2019 Multiple Indicator Cluster Survey [MICS Direct]
Ecuador	30	2017.5	
	5	1976.5	1979-1980 World Fertility Survey [Other DHS Direct]
	5	1984.5	1987 Demographic and Health Survey [DHS Direct]
	3	1986.5	1989 Demographic and Maternal and Child Health Survey [Others Direct]

Continued on next page

Table 22 – continued from previous page

Country	# obs.	Most recent obs. year	Data series name [source type]
	5	1996.5	1999 Demographic and Maternal and Child Health Survey [Others Direct]
	5	2001.5	2004 Demographic and Maternal and Child Health Survey [Others Direct]
	5	2009.5	2012 Encuesta Nacional de Salud y Nutricion, Imputed birth and deaths [Others Direct]
	2	2017.5	Adjusted Vital Registration (Live Births) [VR]
Egypt	61	2011.5	
	11	1979	1980 World Fertility Survey [Other DHS Direct]
	6	1986	1988 Demographic and Health Survey [DHS Direct]
	6	1989	1991 Pan Arab Project for Child Development Maternal and Child Health Survey [Others Direct]
	5	1989.5	1992 Demographic and Health Survey [DHS Direct]
	6	1993	1995 Demographic and Health Survey [DHS Direct]
	1	1993	1997 Interim Demographic and Health Survey [Other DHS Direct]
	1	1994	1998 Interim Demographic and Health Survey [Other DHS Direct]
	5	1997.5	2000 Demographic and Health Survey [DHS Direct]
	5	2000.5	2003 Demographic and Health Survey [Other DHS Direct]
	5	2002.5	2005 Demographic and Health Survey [DHS Direct]
	5	2005.5	2008 Demographic and Health Survey [DHS Direct]
	5	2011.5	2014 Demographic and Health Survey [DHS Direct]
El Salvador	8	2011.5	
	1	1985.5	1988 National Family Health Survey [Others Direct]
	1	1996	1998 National Family Health Survey [Others Direct]
	1	1999.5	2002-2003 National Family Health Survey [Others Direct]
	5	2011.5	2014 Multiple Indicator Cluster Survey [MICS Direct]
Equatorial Guinea	1	2006.7	
	1	2006.7	2011 Demographic and Health Survey [DHS Direct]
Eritrea	15	2007.5	
	4	1992.5	1995-1996 Demographic and Health Survey [DHS Direct]
	5	1999.5	2002 Demographic and Health Survey [DHS Direct]
	5	2007.5	2010 Population and Health Survey [Other DHS Direct]
	1	2005	2010 Population and Health Survey (Preliminary) [Other DHS Direct]
Estonia	40	2020.5	
	40	2020.5	2021 WHO Vital Registration Data 2022 version [VR]
Ethiopia	22	2013.5	
	1	1977.7	1981 Demographic Survey [Others Direct]
	6	1998.5	2000 Demographic and Health Survey [DHS Direct]
	5	2002.5	2005 Demographic and Health Survey [DHS Direct]
	5	2007.5	2011 Demographic and Health Survey [DHS Direct]
	5	2013.5	2016 Demographic and Health Survey [DHS Direct]
Federated States of Micronesia			
Fiji	19	2019.5	
	5	1971.5	1974 World Fertility Survey [Other DHS Direct]

Continued on next page

Table 22 – continued from previous page

Country	# obs.	Most recent obs. year	Data series name [source type]
	14	2019.5	WHO Vital Registration Data 2022 version [VR]
Finland	72	2021.5	
	2	1951.5	UNPD Demographic Yearbook Data 2020 version [VR]
	70	2021.5	WHO Vital Registration Data 2022 version [VR]
France	70	2019.5	
	70	2019.5	2021 WHO Vital Registration Data 2022 version [VR]
Gabon	10	2009.5	
	5	1997.5	2000 Demographic and Health Survey [DHS Direct]
	5	2009.5	2012 Demographic and Health Survey [DHS Direct]
The Gambia	10	2016.5	
	5	2015.5	2018 Multiple Indicator Cluster Survey [MICS Direct]
	5	2016.5	2019-2020 Demographic and Health Survey [DHS Direct]
Georgia	10	2002.5	
	5	1996.5	1999-2000 Reproductive Health Survey [Others Direct]
	5	2002.5	2005 Reproductive Health Survey [Others Direct]
Germany	52	2021.5	
	52	2021.5	2021 WHO Vital Registration Data 2022 version [VR]
Ghana	46	2014.5	
	5	1985.5	1988 Demographic and Health Survey [DHS Direct]
	5	1990.5	1993-1994 Demographic and Health Survey [DHS Direct]
	5	1995.5	1998-1999 Demographic and Health Survey [DHS Direct]
	5	2000.5	2003 Demographic and Health Survey [DHS Direct]
	1	2002	2007 Maternal Health Survey [Other DHS Direct]
	5	2005.5	2008 Demographic and Health Survey [DHS Direct]
	5	2008.5	2011 Multiple Indicator Cluster Survey [MICS Direct]
	5	2011.5	2014 Demographic and Health Survey [DHS Direct]
	5	2014.5	2017 Maternal Health Survey [Other DHS Direct]
	5	2014.5	2017-2018 Multiple Indicator Cluster Survey [MICS Direct]
Greece	65	2020.5	
	65	2020.5	2021 WHO Vital Registration Data 2022 version [VR]
Grenada	56	2020.5	
	21	1978.5	UNPD Demographic Yearbook Data 2020 version [VR]
	35	2020.5	WHO Vital Registration Data 2022 version [VR]
Guatemala	21	2011.5	
	5	1984.5	1987 Demographic and Health Survey [DHS Direct]
	4	1992.5	1995 Demographic and Health Survey [DHS Direct]
	5	1995.5	1998-1999 Demographic and Health Survey [Other DHS Direct]
	1	1996.5	2002 Reproductive Health Survey-CDC [Others Direct]
	1	2003.8	2008-2009 Encuesta Nacional de Salud Materno Infantil (ENSMI) [Others Direct]
	5	2011.5	2014-2015 Demographic and Health Survey [DHS Direct]
Guinea	23	2010.5	
	1	1955	1954-1955 Survey [Others Direct]
	1	1987.8	1992 Demographic and Health Survey [DHS Direct]
	7	1997.5	1999 Demographic and Health Survey [DHS Direct]
	5	2002.5	2005 Demographic and Health Survey [DHS Direct]

Continued on next page

Table 22 – continued from previous page

Country	# obs.	Most recent obs. year	Data series name [source type]
	5	2009.5	2012 Demographic and Health Survey [DHS Direct]
	4	2010.5	2018 Demographic and Health Survey [DHS Direct]
Guinea-Bissau	10	2011.5	
	5	2007.5	2010 Multiple Indicator Cluster Survey [MICS Direct]
	5	2011.5	2014 Multiple Indicator Cluster Survey [MICS Direct]
Guyana	20	2011.5	
	5	1972.5	1975 World Fertility Survey [Other DHS Direct]
	5	2002.5	2005 AIDS Indicator Survey [Other DHS Direct]
	5	2006.5	2009 Demographic and Health Survey [DHS Direct]
	5	2011.5	2014 Multiple Indicator Cluster Survey [MICS Direct]
Haiti	30	2013.5	
	4	1974.5	1977 World Fertility Survey [Other DHS Direct]
	1	1984.5	1987 Mortality, morbidity and service utilization survey [Others Direct]
	5	1991.5	1994-1995 Demographic and Health Survey [DHS Direct]
	5	1997.5	2000 Demographic and Health Survey [DHS Direct]
	5	2002.5	2005-2006 Demographic and Health Survey [DHS Direct]
	5	2009.5	2012 Demographic and Health Survey [DHS Direct]
	5	2013.5	2016-2017 Demographic and Health Survey [DHS Direct]
Honduras	17	2016.5	
	1	1971.5	1972 National Demographic Survey [Others Direct]
	2	1979	1987 National Survey of Epidemiology and Family Health [Others Direct]
	5	2002.5	2005-2006 Demographic and Health Survey [DHS Direct]
	4	2008.5	2011-2012 Demographic and Health Survey [DHS Direct]
	5	2016.5	2019 Multiple Indicator Cluster Survey [MICS Direct]
Hungary	71	2020.5	
	5	1954.5	UNPD Demographic Yearbook Data 2020 version [VR]
	66	2020.5	WHO Vital Registration Data 2022 version [VR]
Iceland	72	2021.5	
	1	1950.5	UNPD Demographic Yearbook Data 2020 version [VR]
	71	2021.5	WHO Vital Registration Data 2022 version [VR]
India	98	2020.5	
	22	1991.5	1992-1993 Demographic and Health Survey [DHS Direct]
	12	1997	1998-1999 Demographic and Health Survey [DHS Direct]
	12	2004	2005-2006 Demographic and Health Survey [DHS Direct]
	21	1989.5	Sample Registration System [VR]
	22	2011.5	Sample Registration System [VR]
	1	2012.5	Sample Registration System [VR]
	1	2013.5	Sample Registration System [VR]
	2	2015.5	Sample Registration System [VR]
	1	2017.5	Sample Registration System [VR]
	1	2018.5	Sample Registration System [VR]
	1	2019.5	Sample Registration System [VR]
	1	2020.5	Sample Registration System [VR]
	1	2016.5	Sample Registration System Statistical Report [VR]
Indonesia	44	2014.5	

Continued on next page

Table 22 – continued from previous page

Country	# obs.	Most recent obs. year	Data series name [source type]
	6	1974	1976 World Fertility Survey [Other DHS Direct]
	6	1985	1987 Demographic and Health Survey [DHS Direct]
	5	1989.5	1991 Demographic and Health Survey [DHS Direct]
	5	1991.5	1994 Demographic and Health Survey [DHS Direct]
	5	1994.5	1997 Demographic and Health Survey [DHS Direct]
	5	1999.5	2002-2003 Demographic and Health Survey [DHS Direct]
	4	1999.5	2007 Demographic and Health Survey [DHS Direct]
	4	2009.5	2012 Demographic and Health Survey [DHS Direct]
	4	2014.5	2017 Demographic and Health Survey [DHS Direct]
Iran (Islamic Republic of)	28	2008.5	
	1	1974.5	1973-1976 Population Growth Survey [Others Direct]
	26	2000	2000 Demographic and Health Survey [Other DHS Direct]
	1	2008.5	2010 National Multiple-Indicator Demographic and Health Survey [Other DHS Direct]
Iraq	14	2008.5	
	1	1973.5	1973 Demographic Sample Survey and Sample Registration System [Others Direct]
	3	2001	2004 Living Conditions Survey [Others Direct]
	5	2003.5	2006 Multiple Indicator Cluster Survey [MICS Direct]
	5	2008.5	2011 Multiple Indicator Cluster Survey [MICS Direct]
Ireland	69	2018.5	
	69	2018.5	2021 WHO Vital Registration Data 2022 version [VR]
Israel	69	2021.5	
	22	1974.5	UNPD Demographic Yearbook Data 2020 version [VR]
	47	2021.5	WHO Vital Registration Data 2022 version [VR]
Italy	70	2020.5	
	70	2020.5	2021 WHO Vital Registration Data 2022 version [VR]
Jamaica	11	2005.5	
	5	1972.5	1975-1976 World Fertility Survey [Other DHS Direct]
	6	2005.5	2008-2009 Reproductive Health Survey [Others Direct]
Japan	71	2021.5	
	71	2021.5	2021 WHO Vital Registration Data 2022 version [VR]
Jordan	35	2014.5	
	5	1973.5	1976 World Fertility Survey [Other DHS Direct]
	5	1987.5	1990 Demographic and Health Survey [DHS Direct]
	5	1994.5	1997 Demographic and Health Survey [DHS Direct]
	5	1999.5	2002 Demographic and Health Survey [DHS Direct]
	5	2004.5	2007 Demographic and Health Survey [DHS Direct]
	5	2009.5	2012 Demographic and Health Survey [DHS Direct]
	5	2014.5	2017 Demographic and Health Survey [DHS Direct]
Kazakhstan	10	1996.5	
	5	1992.5	1995 Demographic and Health Survey [DHS Direct]
	5	1996.5	1999 Demographic and Health Survey [DHS Direct]
Kenya	37	2019.2	
	6	1976	1977-1978 World Fertility Survey [Other DHS Direct]

Continued on next page

Table 22 – continued from previous page

Country	# obs.	Most recent obs. year	Data series name [source type]
	5	1985.5	1989 Demographic and Health Survey [DHS Direct]
	5	1990.5	1993 Demographic and Health Survey [DHS Direct]
	5	1995.5	1998 Demographic and Health Survey [DHS Direct]
	5	2000.5	2003 Demographic and Health Survey [DHS Direct]
	5	2005.5	2008-2009 Demographic and Health Survey [DHS Direct]
	5	2011.5	2014 Demographic and Health Survey [DHS Direct]
	1	2019.2	2019 Census [Others Direct]
Kiribati			
Democratic People's Republic of Korea			
Republic of Korea	26	2021.5	
	5	1971.5	1974 World Fertility Survey [Other DHS Direct]
	21	2021.5	WHO Vital Registration Data 2022 version [VR]
Kosovo	12	2019.5	
	5	2010.5	2013-2014 Multiple Indicator Cluster Survey [MICS Direct]
	5	2016.5	2019-2020 Multiple Indicator Cluster Survey [MICS Direct]
Kuwait	44	2019.5	
	44	2019.5	2021 WHO Vital Registration Data 2022 version [VR]
Kyrgyzstan	19	2015.5	
	4	1994.5	1997 Demographic and Health Survey [DHS Direct]
	5	2009.5	2012 Demographic and Health Survey [DHS Direct]
	5	2011.5	2014 Multiple Indicator Cluster Survey [MICS Direct]
	5	2015.5	2018 Multiple Indicator Cluster Survey [MICS Direct]
Lao People's Democratic Republic	11	2008.5	
	1	1989.5	1994 Fertility and Birth Spacing Survey [Others Direct]
	5	2002.5	2005 Reproductive Health Survey [Others Direct]
	5	2008.5	2012 Lao Social Indicator Survey (combined Multiple Indicator Cluster Survey4/Demographic and Health Survey) [DHS Direct]
Latvia	42	2021.5	
	42	2021.5	2021 WHO Vital Registration Data 2022 version [VR]
Lebanon	5	2002	
	5	2002	2004 Pan Arab Project for Family Health Family Health Survey [Others Direct]
Lesotho	26	2016.3	
	5	1974.5	1977 World Fertility Survey [Other DHS Direct]
	5	2001.5	2004 Demographic and Health Survey [DHS Direct]
	5	2006.5	2009 Demographic and Health Survey [DHS Direct]
	5	2011.5	2014 Demographic and Health Survey [DHS Direct]
	1	2016.3	2016 Census [Others Direct]
	5	2015.5	2018 Multiple Indicator Cluster Survey [MICS Direct]
Liberia	26	2016.5	
	6	1984	1986 Demographic and Health Survey [DHS Direct]
	5	2003.5	2006-2007 Demographic and Health Survey [DHS Direct]

Continued on next page

Table 22 – continued from previous page

Country	# obs.	Most recent obs. year	Data series name [source type]
	5	2005.5	2008-2009 Malaria Indicator Survey [Other DHS Direct]
	5	2010.5	2013 Demographic and Health Survey [DHS Direct]
	5	2016.5	2019-2020 Demographic and Health Survey [DHS Direct]
Libya	10	2005	
	5	1992.5	1995 Pan Arab Project for Child Development Maternal and Child Health Survey [Others Direct]
	5	2005	2007 Pan Arab Project for Family Health Family Health Survey [Others Direct]
Lithuania	54	2021.5	
	54	2021.5	2021 WHO Vital Registration Data 2022 version [VR]
Luxembourg	66	2019.5	
	10	1959.5	UNPD Demographic Yearbook Data 2020 version [VR]
	1	1961.5	WHO Good Vital Registration Data 2018 version [VR]
	55	2019.5	WHO Vital Registration Data 2022 version [VR]
Macedonia	39	2020.5	
	39	2020.5	2021 WHO Vital Registration Data 2022 version [VR]
Madagascar	26	2015.5	
	5	1989.5	1992 Demographic and Health Survey [DHS Direct]
	5	1995	1997 Demographic and Health Survey [DHS Direct]
	5	2000.5	2003-2004 Demographic and Health Survey [DHS Direct]
	5	2005.5	2008-2009 Demographic and Health Survey [DHS Direct]
	1	2005.4	2012-2013 National Survey on Monitoring the MDGs (ENSOMD) [DHS Direct]
	5	2015.5	2018 Multiple Indicator Cluster Survey [MICS Direct]
Malawi	47	2016.5	
	1	1971	1970 Population Change Survey [Others Direct]
	1	1983.5	1984 Family Formation Survey [Others Direct]
	5	1989.5	1992 Demographic and Health Survey [DHS Direct]
	8	1999	2000 Demographic and Health Survey [DHS Direct]
	5	2001.5	2004 Demographic and Health Survey [DHS Direct]
	7	2004.5	2006 Multiple Indicator Cluster Survey [MICS Direct]
	5	2007.5	2010 Demographic and Health Survey [DHS Direct]
	5	2010.5	2013-2014 MDG Endline Survey [MICS Direct]
	5	2012.5	2015-2016 Demographic and Health Survey [DHS Direct]
	5	2016.5	2019-2020 Multiple Indicator Cluster Survey [MICS Direct]
Malaysia	5	2015.5	
	2	2012.5	Vital Statistics [VR]
	1	2013.5	Vital Statistics [VR]
	2	2015.5	Vital Statistics [VR]
Maldives	26	2020.5	
	1	1998	1997 Poverty and Vulnerability Survey [Others Direct]
	5	2006.5	2009 Demographic and Health Survey [DHS Direct]
	5	2013.5	2016-2017 Demographic and Health Survey [DHS Direct]
	15	2020.5	WHO Vital Registration Data 2022 version [VR]
Mali	38	2015.5	
	5	1984.5	1987 Demographic and Health Survey [DHS Direct]

Continued on next page

Table 22 – continued from previous page

Country	# obs.	Most recent obs. year	Data series name [source type]
	10	1994	1995-1996 Demographic and Health Survey [DHS Direct]
	10	2000	2001 Demographic and Health Survey [DHS Direct]
	8	2004.5	2006 Demographic and Health Survey [DHS Direct]
	5	2015.5	2018 Demographic and Health Survey [DHS Direct]
Malta	67	2021.5	
	67	2021.5	2021 WHO Vital Registration Data 2022 version [VR]
Marshall Islands	2	2010.8	
	1	2002.1	2007 Demographic and Health Survey [Other DHS Direct]
	1	2010.8	2011 Census [Others Direct]
Mauritania	25	2016.5	
	5	1979	1981-1982 World Fertility Survey [Other DHS Direct]
	3	1985	1990 Maternal and Child Health Survey [Others Direct]
	5	1997.5	2000-2001 Demographic and Health Survey [DHS Direct]
	1	1998.9	2003-2004 EMIP survey [Others Direct]
	1	2012.8	2013 Census [Others Direct]
	5	2012.5	2015 Multiple Indicator Cluster Survey [MICS Direct]
	5	2016.5	2019-2021 Demographic and Health Survey [DHS Direct]
Mauritius	70	2020.5	
	6	1956.5	UNPD Demographic Yearbook Data 2020 version [VR]
	64	2020.5	WHO Vital Registration Data 2022 version [VR]
Mexico	21	2016.5	
	5	1984.5	1987 Demographic and Health Survey [DHS Direct]
	5	2015.5	2018 Encuesta Nacional de la Dinamica Demografica (ENADID) [Others Direct]
	11	2016.5	Vital Registration Data From Mexico Ministry Of Health [VR]
Republic of Moldova	10	2009.5	
	5	2002.5	2005 Demographic and Health Survey [DHS Direct]
	5	2009.5	2012 Multiple Indicator Cluster Survey [MICS Direct]
Monaco	22	2011.5	
	22	2011.5	2018 WHO Vital Registration Data 2018 version neighbouring [VR]
Mongolia	16	2019.5	
	1	1993	1998 Reproductive Health Survey [Others Direct]
	5	2010.5	2013-2014 Multiple Indicator Cluster Survey [MICS Direct]
	10	2019.5	WHO Vital Registration Data 2022 version [VR]
Montenegro	37	2021.5	
	37	2021.5	2021 WHO Vital Registration Data 2022 version [VR]
Montserrat	30	2008.5	
	30	2008.5	2019 UNPD Vital Registration Data 2019 version [VR]
Morocco	28	2015.5	
	5	1977.5	1980 World Fertility Survey [Other DHS Direct]
	6	1985	1987 Demographic and Health Survey [DHS Direct]
	5	1989.5	1992 Demographic and Health Survey [DHS Direct]
	5	1992.5	1995 Demographic and Health Survey [DHS Direct]
	1	1994.5	1997 PAPG ENSME petit-echantillon 1997 [Others Direct]
	5	2000.5	2003-2004 Demographic and Health Survey [DHS Direct]

Continued on next page

Table 22 – continued from previous page

Country	# obs.	Most recent obs. year	Data series name [source type]
	1	2015.5	2018 National Survey of Population and Family Health [Others Direct]
Mozambique	25	2008.5	
	7	2001.5	2003-2004 Demographic and Health Survey [DHS Direct]
	13	2006.5	2008 Multiple Indicator Cluster Survey [MICS Direct]
	5	2008.5	2011 Demographic and Health Survey [DHS Direct]
Myanmar	10	2012.5	
	2	1985.5	1991 Population Change and Fertility Survey [Others Direct]
	1	1992	1997 Fertility and Reproductive Health Survey [Others Direct]
	1	1996	2001 Fertility and Reproductive Health Survey [Others Direct]
	1	2001	2007 Fertility and Reproductive Health Survey [Others Direct]
	5	2012.5	2015-2016 Demographic and Health Survey [DHS Direct]
Namibia	20	2010.5	
	5	1989.5	1992 Demographic and Health Survey [DHS Direct]
	5	1997.5	2000 Demographic and Health Survey [DHS Direct]
	5	2003.5	2006-2007 Demographic and Health Survey [DHS Direct]
	5	2010.5	2013 Demographic and Health Survey [DHS Direct]
Nauru			
Nepal	44	2016.5	
	7	1974.5	1976 World Fertility Survey [Other DHS Direct]
	1	1986	1991 Fertility and Family Planning Survey [Others Direct]
	6	1994	1996 Demographic and Health Survey [DHS Direct]
	5	1998.5	2001 Demographic and Health Survey [DHS Direct]
	5	2003.5	2006 Demographic and Health Survey [DHS Direct]
	5	2008.5	2011 Demographic and Health Survey [DHS Direct]
	5	2011.5	2014 Multiple Indicator Cluster Survey [MICS Direct]
	5	2013.5	2016 Demographic and Health Survey [DHS Direct]
	5	2016.5	2019 Multiple Indicator Cluster Survey [MICS Direct]
Netherlands	72	2021.5	
	72	2021.5	2021 WHO Vital Registration Data 2022 version [VR]
New Zealand	67	2016.5	
	67	2016.5	2021 WHO Vital Registration Data 2022 version [VR]
Nicaragua	18	2008.5	
	5	1994.5	1998 Demographic and Health Survey [DHS Direct]
	4	1993.5	2001 Demographic and Health Survey [DHS Direct]
	1	2004	2006 Demographic and Health Survey [Other DHS Direct]
	4	2003.5	2006-2007 Encuesta Nicaraguense de Demografia y Salud [Others Direct]
	4	2008.5	2011-2012 Encuesta Nicaraguense de Demografia y Salud [Others Direct]
Niger	32	2018.5	
	6	1990.5	1992 Demographic and Health Survey [DHS Direct]
	7	1996.5	1998 Demographic and Health Survey [DHS Direct]
	6	2004	2006 Demographic and Health Survey [DHS Direct]
	3	2007.5	2010 Child Survival and Mortality Survey New [Others Direct]
	5	2009.5	2012 Demographic and Health Survey [DHS Direct]

Continued on next page

Table 22 – continued from previous page

Country	# obs.	Most recent obs. year	Data series name [source type]
	5	2018.5	2021 Enquete Nationale sur la Fecondite et la Mortalite des Enfants de moins de 5 ans (ENAFEME) [Others Direct]
Nigeria	46	2016.5	
	5	1987.5	1990 Demographic and Health Survey [DHS Direct]
	5	2000.5	2003 Demographic and Health Survey [DHS Direct]
	16	2007.5	2008 Demographic and Health Survey [DHS Direct]
	5	2007.5	2010 Malaria Indicator Survey [Other DHS Direct]
	8	2011.5	2013 Demographic and Health Survey [DHS Direct]
	7	2016.5	2018 Demographic and Health Survey [DHS Direct]
Niue	2	2002.5	
	2	2002.5	2021 WHO Vital Registration Data 2022 version [VR]
Norway	71	2021.5	
	71	2021.5	2021 WHO Vital Registration Data 2022 version [VR]
Oman	4	1990	
	3	1983.5	1988 Child Health Survey [Others Direct]
	1	1990	1995 Family Health Survey [Others Direct]
Pakistan	67	2019.5	
	1	1969.5	1971 Population Growth Survey I [Others Direct]
	6	1973.5	1975 World Fertility Survey [Other DHS Direct]
	1	1977	1976-1978 Population Growth Survey II [Others Direct]
	1	1986	1988 Demographic Survey [Others Direct]
	5	1987.5	1990-1991 Demographic and Health Survey [DHS Direct]
	4	1995.5	1998 Integrated Household Survey [Others Direct]
	1	1999	2000-2001 Reproductive Health and Family Planning Survey [Others Direct]
	4	1997.5	2001 Integrated Household Survey [Others Direct]
	6	2004	2006-2007 Demographic and Health Survey [DHS Direct]
	20	2007.5	2008 Pakistan Demographic Survey [Others Direct]
	5	2009.5	2012-2013 Demographic and Health Survey [DHS Direct]
	1	2010.5	2013-2014 Social
Living Standard Measurement Survey [Others Direct]			
	5	2014.5	2017-2018 Demographic and Health Survey [DHS Direct]
	5	2016.5	2018-2019 Social
Living Standard Measurement Survey [Others Direct]			
	1	2019.5	2020 Pakistan Demographic Survey [Others Direct]
	1	1964	Population Growth Estimation Experiment [VR]
Palau	17	2005.5	
	17	2005.5	2021 WHO Vital Registration Data 2022 version [VR]
Panama	5	1972.5	

Continued on next page

Table 22 – continued from previous page

Country	# obs.	Most recent obs. year	Data series name [source type]
	5	1972.5	1975-1976 World Fertility Survey [Other DHS Direct]
Papua New Guinea	7	2013.5	
	1	1991	1996 Demographic and Health Survey [Other DHS Direct]
	1	2001	2006 Demographic and Health Survey [Other DHS Direct]
	5	2013.5	2016-2018 Demographic and Health Survey [DHS Direct]
Paraguay	19	2005.5	
	5	1976.5	1979 World Fertility Survey [Other DHS Direct]
	5	1987.5	1990 Demographic and Health Survey [DHS Direct]
	4	2001.5	2004 Reproductive Health Survey [Others Direct]
	5	2005.5	2008 Encuesta Nacional de Demografia y Salud Sexual y Reproductiva [Others Direct]
Peru	83	2018.5	
	6	1975	1977-1978 World Fertility Survey [Other DHS Direct]
	5	1983.5	1986 Demographic and Health Survey [DHS Direct]
	5	1988.5	1991-1992 Demographic and Health Survey [DHS Direct]
	7	1994.5	1996 Demographic and Health Survey [DHS Direct]
	5	1997.5	2000 Demographic and Health Survey [DHS Direct]
	4	2000.5	2003-2008 Demographic and Health Survey [DHS Direct]
	4	2006.5	2009 Demographic and Health Survey [DHS Direct]
	5	2007.5	2010 Demographic and Health Survey [DHS Direct]
	5	2008.5	2011 Demographic and Health Survey [DHS Direct]
	5	2009.5	2012 Demographic and Health Survey [DHS Direct]
	1	2008	2013 Demographic and Health Survey [DHS Direct]
	1	2009.5	2014 Encuesta Demografica y de Salud Familiar-ENDES Continua [DHS Direct]
	5	2011.5	2014 National Demographic and Health Survey [Other DHS Direct]
	5	2014.5	2017 Demographic and Family Health Survey [Others Direct]
	5	2015.5	2018 Demographic and Family Health Survey [Other DHS Direct]
	5	2016.5	2019 Encuesta Demografica y de Salud Familiar [Other DHS Direct]
	5	2017.5	2020 Encuesta Demografica y de Salud Familiar [Other DHS Direct]
	5	2018.5	2021 Encuesta Demografica y de Salud Familiar [Other DHS Direct]
Philippines	35	2014.5	
	5	1975.5	1978 World Fertility Survey [Other DHS Direct]
	5	1990.5	1993 Demographic and Health Survey [DHS Direct]
	5	1995.5	1998 Demographic and Health Survey [DHS Direct]
	5	2000.5	2003 Demographic and Health Survey [DHS Direct]
	5	2005.5	2008 Demographic and Health Survey [DHS Direct]
	5	2010.5	2013 National Demographic and Health Survey [DHS Direct]
	5	2014.5	2017 Demographic and Health Survey [DHS Direct]
Poland	72	2021.5	
	9	1958.5	UNPD Demographic Yearbook Data 2020 version [VR]
	63	2021.5	WHO Vital Registration Data 2022 version [VR]
Portugal	72	2021.5	
	5	1954.5	UNPD Demographic Yearbook Data 2020 version [VR]
	67	2021.5	WHO Vital Registration Data 2022 version [VR]
Qatar	30	2020.5	

Continued on next page

Table 22 – continued from previous page

Country	# obs.	Most recent obs. year	Data series name [source type]
	30	2020.5	2021 WHO Vital Registration Data 2022 version [VR]
Romania	65	2021.5	
	3	1958.5	UNPD Demographic Yearbook Data 2020 version [VR]
	62	2021.5	WHO Vital Registration Data 2022 version [VR]
Russian Federation	41	2020.5	
	41	2020.5	2021 WHO Vital Registration Data 2022 version [VR]
Rwanda	34	2016.5	
	5	1981	1983 World Fertility Survey [Other DHS Direct]
	5	1989.5	1992 Demographic and Health Survey [DHS Direct]
	4	1992.5	2000 Demographic and Health Survey [DHS Direct]
	4	2003	2005 Demographic and Health Survey [DHS Direct]
	3	2004.5	2007-2008 Interim Demographic and Health Survey [Other DHS Direct]
	4	2007.5	2010 Demographic and Health Survey [DHS Direct]
	4	2011.5	2014-2015 Demographic and Health Survey [DHS Direct]
	5	2016.5	2019-2020 Demographic and Health Survey [DHS Direct]
Saint Kitts and Nevis	45	2016.5	
	45	2016.5	2021 WHO Vital Registration Data 2022 version [VR]
Saint Lucia	61	2018.5	
	14	1968.5	UNPD Demographic Yearbook Data 2020 version [VR]
	47	2018.5	WHO Vital Registration Data 2022 version [VR]
Samoa	8	2016.5	
	1	1999	1999 Demographic and Health Survey [Others Direct]
	1	1995	2000 Demographic and Vital Statistics Survey [Others Direct]
	1	2011.4	2011 Population and Housing Census [Others Direct]
	5	2016.5	2019-2020 Multiple Indicator Cluster Survey [MICS Direct]
San Marino	11	2003.5	
	2	2003.5	WHO Vital Registration Data 2020 version [VR]
	9	1998.5	WHO Vital Registration Data 2022 version [VR]
Sao Tome and Principe	15	2016.5	
	5	2005.5	2008-2009 Demographic and Health Survey [DHS Direct]
	5	2011.5	2014 Multiple Indicator Cluster Survey [MICS Direct]
	5	2016.5	2019 Multiple Indicator Cluster Survey [MICS Direct]
Saudi Arabia	6	2017.4	
	1	1998.6	1999 Demographic Survey [Others Direct]
	1	1999.6	2000 Demographic Survey [Others Direct]
	1	2004.2	2004 Census [Others Direct]
	1	2006.6	2007 Demographic Survey [Others Direct]
	1	2013.9	2016 Demographic Survey [Others Direct]
	1	2017.4	2017 Household Health Survey [Others Direct]
Senegal	77	2016.5	
	5	1975.5	1978 World Fertility Survey [Other DHS Direct]
	5	1983.5	1986 Demographic and Health Survey [DHS Direct]
	5	1989.5	1992-1993 Demographic and Health Survey [DHS Direct]

Continued on next page

Table 22 – continued from previous page

Country	# obs.	Most recent obs. year	Data series name [source type]
	5	1994.5	1997 Demographic and Health Survey [DHS Direct]
	6	1997	1999-2000 Demographic and Health Survey [DHS Direct]
	6	2003	2005 Demographic and Health Survey [DHS Direct]
	5	2005.5	2008-2009 Malaria Indicator Survey [Other DHS Direct]
	5	2007.5	2010-2011 Demographic and Health Survey [DHS Direct]
	5	2009.5	2012-2013 Demographic and Health Survey [DHS Direct]
	5	2011.5	2014 Demographic and Health Survey [DHS Direct]
	5	2012.5	2015 Demographic and Health Survey [DHS Direct]
	5	2013.5	2016 Demographic and Health Survey [DHS Direct]
	5	2014.5	2017 Demographic and Health Survey [DHS Direct]
	5	2015.5	2018 Demographic and Health Survey [DHS Direct]
	5	2016.5	2019 Demographic and Health Survey [DHS Direct]
Serbia	37	2021.5	
	37	2021.5	2021 WHO Vital Registration Data 2022 version [VR]
Seychelles	51	2019.5	
	51	2019.5	2021 WHO Vital Registration Data 2022 version [VR]
Sierra Leone	18	2016.5	
	1	1992	1992 DSMS [Others Direct]
	5	2000	2004 Census [Census Indirect]
	7	2011.5	2013 Demographic and Health Survey [DHS Direct]
	5	2016.5	2019 Demographic and Health Survey [DHS Direct]
Singapore	71	2020.5	
	5	1954.5	UNPD Demographic Yearbook Data 2020 version [VR]
	66	2020.5	WHO Vital Registration Data 2022 version [VR]
Slovakia	40	2021.5	
	40	2021.5	2021 WHO Vital Registration Data 2022 version [VR]
Slovenia	40	2021.5	
	40	2021.5	2021 WHO Vital Registration Data 2022 version [VR]
Solomon Islands	6	2012.5	
	1	2002	2007 Demographic and Health Survey [Other DHS Direct]
	5	2012.5	2015 Demographic and Health Survey [Other DHS Direct]
Somalia	5	2004	
	5	2004	2006 Multiple Indicator Cluster Survey [MICS Direct]
South Africa	54	2021.5	
	5	1995.5	1998 Demographic and Health Survey [DHS Direct]
	5	2013.5	2016 Demographic and Health Survey [DHS Direct]
	44	2021.5	Rapid Mortality Surveillance (Preliminary) [VR]
South Sudan			
Spain	71	2020.5	
	71	2020.5	2021 WHO Vital Registration Data 2022 version [VR]
Sri Lanka	79	2014.5	
	5	1972.5	1975 World Fertility Survey [Other DHS Direct]
	5	1984.5	1987 Demographic and Health Survey [DHS Direct]
	6	1990.5	1993 Demographic and Health Survey [Other DHS Direct]
	6	1997.5	2000 Demographic and Health Survey [Other DHS Direct]
	6	2003.5	2006 Demographic and Health Survey [Other DHS Direct]

Continued on next page

Table 22 – continued from previous page

Country	# obs.	Most recent obs. year	Data series name [source type]
	5	2013.5	2016 Demographic and Health Survey [Other DHS Direct]
	46	2014.5	WHO Vital Registration Data 2022 version [VR]
Saint Vincent and the Grenadines	58	2017.5	
	10	1964.5	UNPD Demographic Yearbook Data 2020 version [VR]
	48	2017.5	WHO Vital Registration Data 2022 version [VR]
State of Palestine	25	2016.5	
	1	1992.5	1995 Demographic Survey [Others Direct]
	1	1997.5	2000 Health Survey [Others Direct]
	1	2002.3	2004 Demographic and Health Survey [Other DHS Direct]
	6	2005	2006 Pan Arab Project for Family Health Family Health Survey [Others Direct]
	5	2008.1	2010 Multiple Indicator Cluster Survey-Family Health Survey [MICS Direct]
	1	2008	2011 Palestinian Family Survey [Others Direct]
	5	2011.5	2014 Multiple Indicator Cluster Survey [MICS Direct]
	5	2016.5	2019-2020 Multiple Indicator Cluster Survey [MICS Direct]
Sudan	24	2011.5	
	5	1975.5	1978-1979 World Fertility Survey [Other DHS Direct]
	5	1986.5	1989-1990 Demographic and Health Survey [DHS Direct]
	4	1989.5	1992 Pan Arab Project for Child Development Maternal and Child Health Survey [Others Direct]
	1	1995	1999 Safe Motherhood Survey [Others Direct]
	4	2007.5	2010 Multiple Indicator Cluster Survey [MICS Direct]
	5	2011.5	2014 Multiple Indicator Cluster Survey [MICS Direct]
Suriname	5	2015.5	
	5	2015.5	2018 Multiple Indicator Cluster Survey [MICS Direct]
Swaziland	14	2011.5	
	4	2003.5	2006-2007 Demographic and Health Survey [DHS Direct]
	5	2007.5	2010 Multiple Indicator Cluster Survey [MICS Direct]
	5	2011.5	2014 Multiple Indicator Cluster Survey [MICS Direct]
Sweden	71	2021.5	
	71	2021.5	2021 WHO Vital Registration Data 2022 version [VR]
Switzerland	71	2021.5	
	71	2021.5	2021 WHO Vital Registration Data 2022 version [VR]
Syria	16	1999	
	5	1976	1978 World Fertility Survey [Other DHS Direct]
	5	1990.5	1993 Pan Arab Project for Child Development Maternal and Child Health Survey [Others Direct]
	6	1999	2001 Pan Arab Project for Family Health Family Health Survey [Others Direct]
Tajikistan	5	2014.5	
	5	2014.5	2017 Demographic and Health Survey [DHS Direct]
Tanzania	30	2012.5	
	5	1988.5	1991-1992 Demographic and Health Survey [DHS Direct]
	5	1994	1996 Demographic and Health Survey [DHS Direct]

Continued on next page

Table 22 – continued from previous page

Country	# obs.	Most recent obs. year	Data series name [source type]
	5	1996.5	1999 Demographic and Health Survey [DHS Direct]
	5	2001.5	2004-2005 Demographic and Health Survey [DHS Direct]
	5	2006.5	2010 Demographic and Health Survey [DHS Direct]
	5	2012.5	2015-2016 Demographic and Health Survey [DHS Direct]
Thailand	14	1991	
	1	1975	1974-1975 Survey of Population Change [Others Direct]
	5	1972.5	1975 World Fertility Survey [Other DHS Direct]
	1	1985	1985-1986 Survey of Population Change [Others Direct]
	5	1984.5	1987 Demographic and Health Survey [DHS Direct]
	2	1991	1989 Survey of Population Change [Others Direct]
Timor Leste	6	2006.5	
	1	1999	2003 Demographic and Health Survey [Other DHS Direct]
	5	2006.5	2009-2010 Demographic and Health Survey [DHS Direct]
Togo	21	2014.5	
	5	1985.5	1988 Demographic and Health Survey [DHS Direct]
	6	1996	1998 Demographic and Health Survey [DHS Direct]
	5	2010.5	2013 Demographic and Health Survey [DHS Direct]
	5	2014.5	2017 Multiple Indicator Cluster Survey [MICS Direct]
Tonga	5	2016.5	
	1	2007.8	2012 National Demographic and Health Survey [DHS Direct]
	4	2016.5	2019 Multiple Indicator Cluster Survey [MICS Direct]
Trinidad and Tobago	10	1984.5	
	5	1974.5	1977 World Fertility Survey [Other DHS Direct]
	5	1984.5	1987 Demographic and Health Survey [DHS Direct]
Tunisia	24	2017.5	
	5	1985.5	1988 Demographic and Health Survey [DHS Direct]
	5	1992.4	1994 Pan Arab Project for Child Development Maternal and Child Health Survey [Others Direct]
	5	1999	2001 Pan Arab Project for Family Health Family Health Survey [Others Direct]
	1	2009.6	2011-2012 Multiple Indicator Cluster Survey [MICS Direct]
	5	2014.5	Vital Registration from Institut National de la Statistique [VR]
	3	2017.5	WHO Vital Registration Data 2022 version [VR]
Turkey	38	2019.5	
	5	1975.5	1978 World Fertility Survey [Other DHS Direct]
	5	1990.5	1993 Demographic and Health Survey [DHS Direct]
	5	1995.5	1998 Demographic and Health Survey [DHS Direct]
	5	2000.5	2003-2004 Demographic and Health Survey [Other DHS Direct]
	1	2003.8	2008 Turkey Demographic and Health Survey [Other DHS Direct]
	6	2010.5	2013 Demographic and Health Survey [Other DHS Direct]
	11	2019.5	WHO Vital Registration Data 2022 version [VR]
Turkmenistan	6	2016.5	
	1	1995.6	2000 Demographic and Health Survey [DHS Direct]
	5	2016.5	2019 Multiple Indicator Cluster Survey [MICS Direct]

Continued on next page

Table 22 – continued from previous page

Country	# obs.	Most recent obs. year	Data series name [source type]
Turks and Caicos Islands	39	2018.5	
	2	2011.5	2019-2020 Multiple Indicator Cluster Survey [MICS Direct]
	37	2018.5	UNPD Vital Registration Data 2019 version [VR]
Tuvalu	18	2016.5	
	1	2000.3	2002 Census [Others Direct]
	1	2002.7	2007 Demographic and Health Survey [Other DHS Direct]
	5	2016.5	2019-2020 Multiple Indicator Cluster Survey [MICS Direct]
	11	2005.5	WHO Vital Registration Data 2022 version [VR]
Uganda	36	2013.5	
	5	1985.5	1988-1989 Demographic and Health Survey [DHS Direct]
	5	1992.5	1995 Demographic and Health Survey [DHS Direct]
	5	1998	2000-2001 Demographic and Health Survey [DHS Direct]
	6	2004	2006 Demographic and Health Survey [DHS Direct]
	5	2007.5	2009-2010 Malaria Indicator Survey [Other DHS Direct]
	5	2008.5	2011 Demographic and Health Survey [DHS Direct]
	5	2013.5	2016 Demographic and Health Survey [DHS Direct]
Ukraine	10	2009.5	
	1	1995	1999 Reproductive Health Survey [Others Direct]
	5	2004.5	2007 Demographic and Health Survey [DHS Direct]
	4	2009.5	2012 Multiple Indicator Cluster Survey [MICS Direct]
United Arab Emirates	9	2019.5	
	1	1991	1995 Family Health Survey [Others Direct]
	2	2019.5	Vital Registration by Federal Competitiveness and Statistics Center [VR]
	6	2014.5	Vital Registration Data published in UAE IN FIGURES 2014 [VR]
United Kingdom	71	2020.5	
	71	2020.5	2021 WHO Vital Registration Data 2022 version [VR]
United States of America	70	2019.5	
	70	2019.5	2021 WHO Vital Registration Data 2022 version [VR]
Uruguay	62	2020.5	
	62	2020.5	2021 WHO Vital Registration Data 2022 version [VR]
Uzbekistan	1	1997	
	1	1997	2002 Demographic and Health Survey [Other DHS Direct]
Vanuatu	1	2009	
	1	2009	2013 Demographic and Health Survey [Other DHS Direct]
Venezuela (Bolivarian Republic of)	31	2014.5	
	5	1974.5	1977 World Fertility Survey [Other DHS Direct]
	1	1995.5	1998 National Population and Family Survey [Others Direct]
	25	2014.5	WHO Vital Registration Data 2022 version [VR]
Vietnam	34	2020.8	
	5	1985.5	1988 National Demographic and Health Survey [Other DHS Direct]

Continued on next page

Table 22 – continued from previous page

Country	# obs.	Most recent obs. year	Data series name [source type]
	1	1988.5	1994 Intercensal Demographic Survey [Others Direct]
	5	1994.5	1997 Demographic and Health Survey [DHS Direct]
	3	1993.4	1998 Vietnam Longitudinal Survey (VLSS) [Others Direct]
	5	1999.5	2002 Demographic and Health Survey [DHS Direct]
	1	2011.8	2012 Population Change and Family Planning Survey [Others Direct]
	5	2010.5	2013-2014 Multiple Indicator Cluster Survey [MICS Direct]
	1	2012.8	2013 Population Change and Family Planning Survey [Others Direct]
	1	2013.8	2014 Intercensal Population and Housing Survey [Others Direct]
	1	2014.8	2015 Population Change and Family Planning Survey [Others Direct]
	1	2015.8	2016 Population Change and Family Planning Survey [Others Direct]
	1	2016.8	2017 Population Change and Family Planning Survey [Others Direct]
	1	2017.8	2018 Population Change and Family Planning Survey [Others Direct]
	1	2018.8	2019 Census [Others Direct]
	1	2019.8	2020 Population Change and Family Planning Survey (Preliminary) [Others Direct]
	1	2020.8	2021 Population Change and Family Planning Survey (Preliminary) [Others Direct]
Yemen	32	2010.5	
	3	1977	1979 World Fertility Survey [Other DHS Direct]
	6	1989.5	1991-1992 Demographic and Health Survey [DHS Direct]
	8	1996	1997 Demographic and Health Survey [DHS Direct]
	5	2001.5	2003 Pan Arab Project for Family Health Family Health Survey [Others Direct]
	5	2003.5	2006 Multiple Indicator Cluster Survey [MICS Direct]
	5	2010.5	2013 Demographic and Health Survey [DHS Direct]
Zambia	33	2015.5	
	5	1990	1992 Demographic and Health Survey [DHS Direct]
	6	1994.5	1996-1997 Demographic and Health Survey [DHS Direct]
	6	1999.5	2001-2002 Demographic and Health Survey [DHS Direct]
	6	2004.5	2007 Demographic and Health Survey [DHS Direct]
	5	2010.5	2013-2014 Demographic and Health Survey [DHS Direct]
	5	2015.5	2018 Demographic and Health Survey [DHS Direct]
Zimbabwe	41	2016.5	
	5	1985.5	1988-1989 Demographic and Health Survey [DHS Direct]
	5	1991.5	1994 Demographic and Health Survey [DHS Direct]
	1	1997	1997 Inter-censal Demographic Survey [Others Direct]
	5	1996.5	1999 Demographic and Health Survey [DHS Direct]
	5	2006.5	2009 Multiple Indicator Cluster Survey [MICS Direct]
	5	2007.5	2010-2011 Demographic and Health Survey [DHS Direct]
	5	2011.5	2014 Multiple Indicator Cluster Survey [MICS Direct]
	5	2012.5	2015 Demographic and Health Survey [DHS Direct]
	5	2016.5	2019 Multiple Indicator Cluster Survey [MICS Direct]

Table 23: **Sex ratio data sources for age group 1–4, by country.** For each country, the total number of observations and the most recent reference year are shown after the country name. For each country-specific data series, the number of observations and the most recent reference year within that series is shown before each data series name. The source type that each data series falls in is shown in parentheses after each data series name.

Country	# obs.	Most recent obs. year	Data series name [source type]
Afghanistan	15	2015.5	
	5	2007.5	2010 Afghanistan Mortality Survey [DHS Direct]
	5	2012.5	2015 Demographic and Health Survey [DHS Direct]
	5	2015.5	2018 Afghanistan Health Survey [Others Direct]
Albania	24	2021.5	
	4	1999.5	2002 Reproductive Health Survey [Others Direct]
	4	2000.5	2008-2009 Demographic and Health Survey [DHS Direct]
	16	2021.5	WHO Vital Registration Data 2022 version [VR]
Algeria	39	2020.5	
	1	1976.5	1986 Enquete nationale sur la fecondite [Others Direct]
	5	1989.5	1992 Pan Arab Project for Child Development Maternal and Child Health Survey [Others Direct]
	1	1993	1995 Enquete nationale sur les objectifs [Others Direct]
	5	2000	2002 Pan Arab Project for Family Health Family Health Survey [Others Direct]
	22	2020.5	Vital Registration from Demographie Algerienne [VR]
	5	1986.5	WHO Vital Registration Data 2022 version [VR]
Andorra	32	2014.5	
	5	2014.5	WHO Vital Registration Data 2019 version [VR]
	5	2014.5	WHO Vital Registration Data 2020 version [VR]
	10	2014.5	WHO Vital Registration Data 2021 version [VR]
	12	2014.5	WHO Vital Registration Data 2022 version [VR]
Angola	5	2012.5	
	5	2012.5	2015-2016 Inquerito de Indicadores Multiplos e de Saude [DHS Direct]
Anguilla	11	2015.5	
	11	2015.5	2019 UNPD Vital Registration Data 2019 version [VR]
Antigua and Barbuda	51	2017.5	
	11	1960.5	UNPD Demographic Yearbook Data 2020 version [VR]
	40	2017.5	WHO Vital Registration Data 2022 version [VR]
Argentina	45	2020.5	
	45	2020.5	2021 WHO Vital Registration Data 2022 version [VR]
Armenia	12	2007.5	
	5	1997.5	2000 Demographic and Health Survey [DHS Direct]
	4	1997.5	2005 Demographic and Health Survey [DHS Direct]
	3	2007.5	2010 Demographic and Health Survey [DHS Direct]
Australia	71	2020.5	
	71	2020.5	2021 WHO Vital Registration Data 2022 version [VR]
Austria	66	2020.5	
	66	2020.5	2021 WHO Vital Registration Data 2022 version [VR]
Azerbaijan	11	2008.5	
	1	1996	2001 Reproductive Health Survey [Others Direct]

Continued on next page

Table 23 – continued from previous page

Country	# obs.	Most recent obs. year	Data series name [source type]
	5	2003.5	2006 Demographic and Health Survey [DHS Direct]
	5	2008.5	2011 Azerbaijan National Demographic and Health Survey [Other DHS Direct]
Bahamas	48	2020.5	
	48	2020.5	2021 WHO Vital Registration Data 2022 version [VR]
Bahrain	42	2019.5	
	1	1984	1989 Child Health Survey [Others Direct]
	1	1990.5	1995 Gulf Family Health Survey [Others Direct]
	5	2019.5	UNPD Vital Registration Data 2019 version [VR]
	35	2014.5	WHO Vital Registration Data 2022 version [VR]
Bangladesh	91	2020.5	
	5	1972.5	1975-1976 World Fertility Survey [Other DHS Direct]
	1	1981	1988-1989 Fertility Survey [Others Direct]
	4	1990.5	1993-1994 Demographic and Health Survey [DHS Direct]
	5	1993.5	1996-1997 Demographic and Health Survey [DHS Direct]
	5	1996.5	1999-2000 Demographic and Health Survey [DHS Direct]
	1	1999	2001 Maternal Health Services and Maternal Mortality Survey [Other DHS Direct]
	5	2001.5	2004 Demographic and Health Survey [DHS Direct]
	5	2004.5	2007 Demographic and Health Survey [DHS Direct]
	5	2008.5	2011 Demographic and Health Survey [DHS Direct]
	1	2008.1	2012-2013 Multiple Indicator Cluster Survey [MICS Direct]
	5	2011.5	2014 Demographic and Health Survey [DHS Direct]
	5	2014.5	2017-2018 Demographic and Health Survey [DHS Direct]
	5	2016.5	2019 Multiple Indicator Cluster Survey [MICS Direct]
	10	2020.5	Sample Vital Statistics [VR]
	29	2010.5	SVital Registration from Report on Sample Vital Registration System 2010 [VR]
Barbados	54	2013.5	
	54	2013.5	2021 WHO Vital Registration Data 2022 version [VR]
Belarus	39	2019.5	
	39	2019.5	2021 WHO Vital Registration Data 2022 version [VR]
Belgium	70	2019.5	
	4	1953.5	UNPD Demographic Yearbook Data 2020 version [VR]
	66	2019.5	WHO Vital Registration Data 2022 version [VR]
Belize	23	2018.5	
	5	1988.5	1991 Family Health Survey [Others Direct]
	18	2018.5	WHO Vital Registration Data 2022 version [VR]
Benin	30	2014.5	
	5	1978.5	1981-1982 World Fertility Survey [Other DHS Direct]
	5	1993.5	1996 Demographic and Health Survey [DHS Direct]
	5	1998.5	2001 Demographic and Health Survey [DHS Direct]
	5	2003.5	2006 Demographic and Health Survey [DHS Direct]
	5	2011.5	2014 Multiple Indicator Cluster Survey [MICS Direct]
	5	2014.5	2017-2018 Demographic and Health Survey [DHS Direct]
Bhutan	3	1999.7	

Continued on next page

Table 23 – continued from previous page

Country	# obs.	Most recent obs. year	Data series name [source type]
	1	1983.5	1984 Demographic Sample Survey [Others Direct]
	1	1993.5	1994 National Health Survey [Others Direct]
	1	1999.7	2000 National Health Survey [Others Direct]
Bolivia (Plurinational State of)	30	2013.5	
	5	1986.5	1989 Demographic and Health Survey [DHS Direct]
	5	1990.5	1993-1994 Demographic and Health Survey [DHS Direct]
	5	1995.5	1998 Demographic and Health Survey [DHS Direct]
	5	2000.5	2003 Demographic and Health Survey [DHS Direct]
	5	2005.5	2008 Demographic and Health Survey [DHS Direct]
	5	2013.5	2016 Demographic and Health Survey [Other DHS Direct]
Bosnia and Herzegovina	25	2016.5	
	25	2016.5	2021 WHO Vital Registration Data 2022 version [VR]
Botswana	11	2005.4	
	5	1985.5	1988 Family Health Survey [DHS Direct]
	6	2005.4	2007 Family Health Survey [Others Direct]
Brazil	10	1993.5	
	5	1983.5	1986 Demographic and Health Survey [DHS Direct]
	5	1993.5	1996 Demographic and Health Survey [DHS Direct]
British Virgin Islands	37	2008.5	
	37	2008.5	2019 UNPD Vital Registration Data 2019 version [VR]
Brunei	63	2019.5	
	25	1978.5	UNPD Demographic Yearbook Data 2020 version [VR]
	38	2019.5	WHO Vital Registration Data 2022 version [VR]
Bulgaria	58	2021.5	
	58	2021.5	2021 WHO Vital Registration Data 2022 version [VR]
Burkina Faso	20	2007.5	
	5	1989.5	1993 Demographic and Health Survey [DHS Direct]
	5	1995.5	1998-1999 Demographic and Health Survey [DHS Direct]
	5	2000.5	2003 Demographic and Health Survey [DHS Direct]
	5	2007.5	2010 Demographic and Health Survey [DHS Direct]
Burundi	15	2013.5	
	1	1970.5	1970 Demographic Survey [Others Direct]
	5	1984.5	1987 Demographic and Health Survey [DHS Direct]
	5	2007.5	2010-2011 Demographic and Health Survey [DHS Direct]
	4	2013.5	2016-2017 Demographic and Health Survey [DHS Direct]
Cambodia	20	2011.5	
	5	1997.5	2000 Demographic and Health Survey [DHS Direct]
	5	2002.5	2005 Demographic and Health Survey [DHS Direct]
	5	2007.5	2010 Demographic and Health Survey [DHS Direct]
	5	2011.5	2014 Demographic and Health Survey [DHS Direct]
Cameroon	30	2015.5	
	5	1975.5	1978 World Fertility Survey [Other DHS Direct]

Continued on next page

Table 23 – continued from previous page

Country	# obs.	Most recent obs. year	Data series name [source type]
	5	1988.5	1991 Demographic and Health Survey [DHS Direct]
	5	1995.5	1998 Demographic and Health Survey [DHS Direct]
	5	2001.5	2004 Demographic and Health Survey [DHS Direct]
	5	2008.5	2011 Demographic and Health Survey [DHS Direct]
	5	2015.5	2018 Demographic and Health Survey [DHS Direct]
Canada	71	2020.5	
	71	2020.5	2021 WHO Vital Registration Data 2022 version [VR]
Cape Verde	32	2016.5	
	5	1995.5	1998 Demographic and Reproductive Health Survey [Others Direct]
	1	2002.2	2005 Demographic and Health Survey [DHS Direct]
	26	2016.5	WHO Vital Registration Data 2022 version [VR]
Central African Republic	10	2015.5	
	5	1991.5	1994-1995 Demographic and Health Survey [DHS Direct]
	5	2015.5	2018-2019 Multiple Indicator Cluster Survey [MICS Direct]
Chad	15	2011.5	
	5	1993.5	1996-1997 Demographic and Health Survey [DHS Direct]
	5	2001.5	2004 Demographic and Health Survey [DHS Direct]
	5	2011.5	2014-2015 Demographic and Health Survey [DHS Direct]
Chile	56	2015.5	
	56	2015.5	2021 WHO Vital Registration Data 2022 version [VR]
China	5	1986	
	5	1986	1988 National Survey on Fertility and Birth Control [Others Direct]
Colombia	35	2012.5	
	5	1983.5	1986 Demographic and Health Survey [DHS Direct]
	5	1987.5	1990 Demographic and Health Survey [DHS Direct]
	5	1992.5	1995 Demographic and Health Survey [DHS Direct]
	5	1997.5	2000 Demographic and Health Survey [DHS Direct]
	5	2001.5	2005 Demographic and Health Survey [DHS Direct]
	5	2006.5	2010 Demographic and Health Survey [DHS Direct]
	5	2012.5	2015 Demographic and Health Survey [DHS Direct]
Comoros	6	2017.5	
	5	1993.5	1996 Demographic and Health Survey [DHS Direct]
	1	2017.5	2017 Census [Others Direct]
Congo	10	2008.5	
	5	2002.5	2005 Demographic and Health Survey [DHS Direct]
	5	2008.5	2011-2012 Demographic and Health Survey [DHS Direct]
Democratic Republic of the Congo	10	2010.5	
	5	2004.5	2007 Demographic and Health Survey [DHS Direct]
	5	2010.5	2013-2014 Demographic and Health Survey [DHS Direct]
Cook Islands	7	2001.5	
	7	2001.5	2021 WHO Vital Registration Data 2022 version [VR]
Costa Rica	71	2020.5	
	6	1955.5	UNPD Demographic Yearbook Data 2020 version [VR]

Continued on next page

Table 23 – continued from previous page

Country	# obs.	Most recent obs. year	Data series name [source type]
	65	2020.5	WHO Vital Registration Data 2022 version [VR]
Cote d'Ivoire	31	2013.5	
	1	1978.5	1978 Demographic Survey Repeated Passages [Others Direct]
	5	1977.5	1980-1981 World Fertility Survey [Other DHS Direct]
	5	1991.5	1994 Demographic and Health Survey [DHS Direct]
	5	1995.5	1998-1999 Demographic and Health Survey [DHS Direct]
	5	2002.5	2005 AIDS Indicator Survey [Other DHS Direct]
	5	2008.5	2011-2012 Demographic and Health Survey [DHS Direct]
	5	2013.5	2016 Multiple Indicator Cluster Survey [MICS Direct]
Croatia	39	2021.5	
	39	2021.5	2021 WHO Vital Registration Data 2022 version [VR]
Cuba	55	2020.5	
	55	2020.5	2021 WHO Vital Registration Data 2022 version [VR]
Cyprus	35	2020.5	
	35	2020.5	2021 WHO Vital Registration Data 2022 version [VR]
Czech Republic	39	2021.5	
	39	2021.5	2021 WHO Vital Registration Data 2022 version [VR]
Denmark	70	2021.5	
	70	2021.5	2021 WHO Vital Registration Data 2022 version [VR]
Djibouti	6	2010	
	5	1999.5	2002 Pan Arab Project for Family Health Family Health Survey [Others Direct]
	1	2010	2012 Pan Arab Project for Family Health Family Health Survey [Others Direct]
Dominica	36	2015.5	
	36	2015.5	2021 WHO Vital Registration Data 2022 version [VR]
Dominican Republic	45	2016.5	
	5	1972.5	1975 World Fertility Survey [Other DHS Direct]
	5	1983.5	1986 Demographic and Health Survey [DHS Direct]
	5	1988.5	1991 Demographic and Health Survey [DHS Direct]
	5	1993.5	1996 Demographic and Health Survey [DHS Direct]
	5	1999.5	2002 Demographic and Health Survey [DHS Direct]
	5	2004.5	2007 Demographic and Health Survey [DHS Direct]
	5	2010.5	2013 Demographic and Health Survey [DHS Direct]
	5	2011.5	2014 Multiple Indicator Cluster Survey [MICS Direct]
	5	2016.5	2019 Multiple Indicator Cluster Survey [MICS Direct]
Ecuador	30	2017.5	
	5	1976.5	1979-1980 World Fertility Survey [Other DHS Direct]
	5	1984.5	1987 Demographic and Health Survey [DHS Direct]
	3	1986.5	1989 Demographic and Maternal and Child Health Survey [Others Direct]
	5	1996.5	1999 Demographic and Maternal and Child Health Survey [Others Direct]
	5	2001.5	2004 Demographic and Maternal and Child Health Survey [Others Direct]

Continued on next page

Table 23 – continued from previous page

Country	# obs.	Most recent obs. year	Data series name [source type]
	5	2009.5	2012 Encuesta Nacional de Salud y Nutricion, Imputed birth and deaths [Others Direct]
	2	2017.5	Adjusted Vital Registration (Live Births) [VR]
Egypt	52	2011.5	
	5	1977.5	1980 World Fertility Survey [Other DHS Direct]
	5	1985.5	1988 Demographic and Health Survey [DHS Direct]
	5	1988.5	1991 Pan Arab Project for Child Development Maternal and Child Health Survey [Others Direct]
	5	1989.5	1992 Demographic and Health Survey [DHS Direct]
	5	1992.5	1995 Demographic and Health Survey [DHS Direct]
	1	1993	1997 Interim Demographic and Health Survey [Other DHS Direct]
	1	1994	1998 Interim Demographic and Health Survey [Other DHS Direct]
	5	1997.5	2000 Demographic and Health Survey [DHS Direct]
	5	2000.5	2003 Demographic and Health Survey [Other DHS Direct]
	5	2002.5	2005 Demographic and Health Survey [DHS Direct]
	5	2005.5	2008 Demographic and Health Survey [DHS Direct]
	5	2011.5	2014 Demographic and Health Survey [DHS Direct]
El Salvador	8	2010.5	
	1	1985.5	1988 National Family Health Survey [Others Direct]
	1	1996	1998 National Family Health Survey [Others Direct]
	1	1999.5	2002-2003 National Family Health Survey [Others Direct]
	5	2010.5	2014 Multiple Indicator Cluster Survey [MICS Direct]
Equatorial Guinea	1	2006.7	
	1	2006.7	2011 Demographic and Health Survey [DHS Direct]
Eritrea	15	2007.5	
	4	1992.5	1995-1996 Demographic and Health Survey [DHS Direct]
	5	1999.5	2002 Demographic and Health Survey [DHS Direct]
	5	2007.5	2010 Population and Health Survey [Other DHS Direct]
	1	2005	2010 Population and Health Survey (Preliminary) [Other DHS Direct]
Estonia	40	2020.5	
	40	2020.5	2021 WHO Vital Registration Data 2022 version [VR]
Ethiopia	21	2013.5	
	1	1977.7	1981 Demographic Survey [Others Direct]
	5	1997.5	2000 Demographic and Health Survey [DHS Direct]
	5	2002.5	2005 Demographic and Health Survey [DHS Direct]
	5	2007.5	2011 Demographic and Health Survey [DHS Direct]
	5	2013.5	2016 Demographic and Health Survey [DHS Direct]
Federated States of Micronesia			
Fiji	19	2019.5	
	5	1971.5	1974 World Fertility Survey [Other DHS Direct]
	14	2019.5	WHO Vital Registration Data 2022 version [VR]
Finland	72	2021.5	
	2	1951.5	UNPD Demographic Yearbook Data 2020 version [VR]
	70	2021.5	WHO Vital Registration Data 2022 version [VR]
France	70	2019.5	

Continued on next page

Table 23 – continued from previous page

Country	# obs.	Most recent obs. year	Data series name [source type]
	70	2019.5	2021 WHO Vital Registration Data 2022 version [VR]
Gabon	9	2009.5	
	4	1997.5	2000 Demographic and Health Survey [DHS Direct]
	5	2009.5	2012 Demographic and Health Survey [DHS Direct]
The Gambia	10	2016.5	
	5	2015.5	2018 Multiple Indicator Cluster Survey [MICS Direct]
	5	2016.5	2019-2020 Demographic and Health Survey [DHS Direct]
Georgia	6	2002.5	
	4	1996.5	1999-2000 Reproductive Health Survey [Others Direct]
	2	2002.5	2005 Reproductive Health Survey [Others Direct]
Germany	52	2021.5	
	52	2021.5	2021 WHO Vital Registration Data 2022 version [VR]
Ghana	46	2014.5	
	5	1985.5	1988 Demographic and Health Survey [DHS Direct]
	5	1990.5	1993-1994 Demographic and Health Survey [DHS Direct]
	5	1995.5	1998-1999 Demographic and Health Survey [DHS Direct]
	5	2000.5	2003 Demographic and Health Survey [DHS Direct]
	1	2002	2007 Maternal Health Survey [Other DHS Direct]
	5	2005.5	2008 Demographic and Health Survey [DHS Direct]
	5	2008.5	2011 Multiple Indicator Cluster Survey [MICS Direct]
	5	2011.5	2014 Demographic and Health Survey [DHS Direct]
	5	2014.5	2017 Maternal Health Survey [Other DHS Direct]
	5	2014.5	2017-2018 Multiple Indicator Cluster Survey [MICS Direct]
Greece	65	2020.5	
	65	2020.5	2021 WHO Vital Registration Data 2022 version [VR]
Grenada	48	2018.5	
	21	1978.5	UNPD Demographic Yearbook Data 2020 version [VR]
	27	2018.5	WHO Vital Registration Data 2022 version [VR]
Guatemala	21	2011.5	
	5	1984.5	1987 Demographic and Health Survey [DHS Direct]
	4	1992.5	1995 Demographic and Health Survey [DHS Direct]
	5	1995.5	1998-1999 Demographic and Health Survey [Other DHS Direct]
	1	1996.5	2002 Reproductive Health Survey-CDC [Others Direct]
	1	2003.8	2008-2009 Encuesta Nacional de Salud Materno Infantil (ENSMI) [Others Direct]
	5	2011.5	2014-2015 Demographic and Health Survey [DHS Direct]
Guinea	21	2010.5	
	1	1955	1954-1955 Survey [Others Direct]
	1	1987.8	1992 Demographic and Health Survey [DHS Direct]
	5	1996.5	1999 Demographic and Health Survey [DHS Direct]
	5	2002.5	2005 Demographic and Health Survey [DHS Direct]
	5	2009.5	2012 Demographic and Health Survey [DHS Direct]
	4	2010.5	2018 Demographic and Health Survey [DHS Direct]
Guinea-Bissau	10	2011.5	
	5	2007.5	2010 Multiple Indicator Cluster Survey [MICS Direct]

Continued on next page

Table 23 – continued from previous page

**Country**	**# obs.**	**Most recent obs. year**	**Data series name [source type]**
	5	2011.5	2014 Multiple Indicator Cluster Survey [MICS Direct]
Guyana	18	2011.5	
	5	1972.5	1975 World Fertility Survey [Other DHS Direct]
	4	2002.5	2005 AIDS Indicator Survey [Other DHS Direct]
	4	2001.5	2009 Demographic and Health Survey [DHS Direct]
	5	2011.5	2014 Multiple Indicator Cluster Survey [MICS Direct]
Haiti	30	2013.5	
	4	1974.5	1977 World Fertility Survey [Other DHS Direct]
	1	1984.5	1987 Mortality, morbidity and service utilization survey [Others Direct]
	5	1991.5	1994-1995 Demographic and Health Survey [DHS Direct]
	5	1997.5	2000 Demographic and Health Survey [DHS Direct]
	5	2002.5	2005-2006 Demographic and Health Survey [DHS Direct]
	5	2009.5	2012 Demographic and Health Survey [DHS Direct]
	5	2013.5	2016-2017 Demographic and Health Survey [DHS Direct]
Honduras	17	2016.5	
	1	1971.5	1972 National Demographic Survey [Others Direct]
	2	1979	1987 National Survey of Epidemiology and Family Health [Others Direct]
	5	2002.5	2005-2006 Demographic and Health Survey [DHS Direct]
	4	2008.5	2011-2012 Demographic and Health Survey [DHS Direct]
	5	2016.5	2019 Multiple Indicator Cluster Survey [MICS Direct]
Hungary	71	2020.5	
	5	1954.5	UNPD Demographic Yearbook Data 2020 version [VR]
	66	2020.5	WHO Vital Registration Data 2022 version [VR]
Iceland	59	2018.5	
	1	1950.5	UNPD Demographic Yearbook Data 2020 version [VR]
	58	2018.5	WHO Vital Registration Data 2022 version [VR]
India	60	2020.5	
	5	1989.5	1992-1993 Demographic and Health Survey [DHS Direct]
	5	1995.5	1998-1999 Demographic and Health Survey [DHS Direct]
	5	2002.5	2005-2006 Demographic and Health Survey [DHS Direct]
	15	1989.5	Sample Registration System [VR]
	21	2011.5	Sample Registration System [VR]
	1	2012.5	Sample Registration System [VR]
	1	2013.5	Sample Registration System [VR]
	2	2015.5	Sample Registration System [VR]
	1	2017.5	Sample Registration System [VR]
	1	2018.5	Sample Registration System [VR]
	1	2019.5	Sample Registration System [VR]
	1	2020.5	Sample Registration System [VR]
	1	2016.5	Sample Registration System Statistical Report [VR]
Indonesia	42	2014.5	
	5	1973.5	1976 World Fertility Survey [Other DHS Direct]
	5	1984.5	1987 Demographic and Health Survey [DHS Direct]
	5	1988.5	1991 Demographic and Health Survey [DHS Direct]
	5	1991.5	1994 Demographic and Health Survey [DHS Direct]

Continued on next page

Table 23 – continued from previous page

Country	# obs.	Most recent obs. year	Data series name [source type]
	5	1994.5	1997 Demographic and Health Survey [DHS Direct]
	5	1999.5	2002-2003 Demographic and Health Survey [DHS Direct]
	4	1999.5	2007 Demographic and Health Survey [DHS Direct]
	4	2009.5	2012 Demographic and Health Survey [DHS Direct]
	4	2014.5	2017 Demographic and Health Survey [DHS Direct]
Iran (Islamic Republic of)	28	2008.5	
	1	1974.5	1973-1976 Population Growth Survey [Others Direct]
	26	2000	2000 Demographic and Health Survey [Other DHS Direct]
	1	2008.5	2010 National Multiple-Indicator Demographic and Health Survey [Other DHS Direct]
Iraq	14	2008.5	
	1	1973.5	1973 Demographic Sample Survey and Sample Registration System [Others Direct]
	3	2001	2004 Living Conditions Survey [Others Direct]
	5	2003.5	2006 Multiple Indicator Cluster Survey [MICS Direct]
	5	2008.5	2011 Multiple Indicator Cluster Survey [MICS Direct]
Ireland	69	2018.5	
	69	2018.5	2021 WHO Vital Registration Data 2022 version [VR]
Israel	69	2021.5	
	22	1974.5	UNPD Demographic Yearbook Data 2020 version [VR]
	47	2021.5	WHO Vital Registration Data 2022 version [VR]
Italy	70	2020.5	
	70	2020.5	2021 WHO Vital Registration Data 2022 version [VR]
Jamaica	7	2003	
	5	1972.5	1975-1976 World Fertility Survey [Other DHS Direct]
	2	2003	2008-2009 Reproductive Health Survey [Others Direct]
Japan	71	2021.5	
	71	2021.5	2021 WHO Vital Registration Data 2022 version [VR]
Jordan	35	2014.5	
	5	1973.5	1976 World Fertility Survey [Other DHS Direct]
	5	1987.5	1990 Demographic and Health Survey [DHS Direct]
	5	1994.5	1997 Demographic and Health Survey [DHS Direct]
	5	1999.5	2002 Demographic and Health Survey [DHS Direct]
	5	2004.5	2007 Demographic and Health Survey [DHS Direct]
	5	2009.5	2012 Demographic and Health Survey [DHS Direct]
	5	2014.5	2017 Demographic and Health Survey [DHS Direct]
Kazakhstan	9	1996.5	
	4	1992.5	1995 Demographic and Health Survey [DHS Direct]
	5	1996.5	1999 Demographic and Health Survey [DHS Direct]
Kenya	36	2019.2	
	5	1974.5	1977-1978 World Fertility Survey [Other DHS Direct]
	5	1985.5	1989 Demographic and Health Survey [DHS Direct]
	5	1990.5	1993 Demographic and Health Survey [DHS Direct]
	5	1995.5	1998 Demographic and Health Survey [DHS Direct]
	5	2000.5	2003 Demographic and Health Survey [DHS Direct]

Continued on next page

Table 23 – continued from previous page

Country	# obs.	Most recent obs. year	Data series name [source type]
	5	2005.5	2008-2009 Demographic and Health Survey [DHS Direct]
	5	2011.5	2014 Demographic and Health Survey [DHS Direct]
	1	2019.2	2019 Census [Others Direct]
Kiribati			
Democratic People's Republic of Korea			
Republic of Korea	26	2021.5	
	5	1971.5	1974 World Fertility Survey [Other DHS Direct]
	21	2021.5	WHO Vital Registration Data 2022 version [VR]
Kosovo	11	2019.5	
	5	2010.5	2013-2014 Multiple Indicator Cluster Survey [MICS Direct]
	4	2016.5	2019-2020 Multiple Indicator Cluster Survey [MICS Direct]
Kuwait	44	2019.5	
	44	2019.5	2021 WHO Vital Registration Data 2022 version [VR]
Kyrgyzstan	17	2015.5	
	4	1994.5	1997 Demographic and Health Survey [DHS Direct]
	5	2009.5	2012 Demographic and Health Survey [DHS Direct]
	5	2011.5	2014 Multiple Indicator Cluster Survey [MICS Direct]
	3	2015.5	2018 Multiple Indicator Cluster Survey [MICS Direct]
Lao People's Democratic Republic	11	2008.5	
	1	1989.5	1994 Fertility and Birth Spacing Survey [Others Direct]
	5	2002.5	2005 Reproductive Health Survey [Others Direct]
	5	2008.5	2012 Lao Social Indicator Survey (combined Multiple Indicator Cluster Survey4/Demographic and Health Survey) [DHS Direct]
Latvia	42	2021.5	
	42	2021.5	2021 WHO Vital Registration Data 2022 version [VR]
Lebanon	4	2002	
	4	2002	2004 Pan Arab Project for Family Health Family Health Survey [Others Direct]
Lesotho	26	2016.3	
	5	1974.5	1977 World Fertility Survey [Other DHS Direct]
	5	2001.5	2004 Demographic and Health Survey [DHS Direct]
	5	2006.5	2009 Demographic and Health Survey [DHS Direct]
	5	2011.5	2014 Demographic and Health Survey [DHS Direct]
	1	2016.3	2016 Census [Others Direct]
	5	2015.5	2018 Multiple Indicator Cluster Survey [MICS Direct]
Liberia	25	2016.5	
	5	1983.5	1986 Demographic and Health Survey [DHS Direct]
	5	2003.5	2006-2007 Demographic and Health Survey [DHS Direct]
	5	2005.5	2008-2009 Malaria Indicator Survey [Other DHS Direct]
	5	2010.5	2013 Demographic and Health Survey [DHS Direct]
	5	2016.5	2019-2020 Demographic and Health Survey [DHS Direct]
Libya	10	2005	

Continued on next page

Table 23 – continued from previous page

Country	# obs.	Most recent obs. year	Data series name [source type]
	5	1992.5	1995 Pan Arab Project for Child Development Maternal and Child Health Survey [Others Direct]
	5	2005	2007 Pan Arab Project for Family Health Family Health Survey [Others Direct]
Lithuania	54	2021.5	
	54	2021.5	2021 WHO Vital Registration Data 2022 version [VR]
Luxembourg	60	2017.5	
	10	1959.5	UNPD Demographic Yearbook Data 2020 version [VR]
	1	1961.5	WHO Good Vital Registration Data 2018 version [VR]
	49	2017.5	WHO Vital Registration Data 2022 version [VR]
Macedonia	39	2020.5	
	39	2020.5	2021 WHO Vital Registration Data 2022 version [VR]
Madagascar	26	2015.5	
	5	1989.5	1992 Demographic and Health Survey [DHS Direct]
	5	1994.5	1997 Demographic and Health Survey [DHS Direct]
	5	2000.5	2003-2004 Demographic and Health Survey [DHS Direct]
	5	2005.5	2008-2009 Demographic and Health Survey [DHS Direct]
	1	2005.4	2012-2013 National Survey on Monitoring the MDGs (ENSOMD) [DHS Direct]
	5	2015.5	2018 Multiple Indicator Cluster Survey [MICS Direct]
Malawi	37	2016.5	
	1	1971	1970 Population Change Survey [Others Direct]
	1	1983.5	1984 Family Formation Survey [Others Direct]
	5	1989.5	1992 Demographic and Health Survey [DHS Direct]
	5	1997.5	2000 Demographic and Health Survey [DHS Direct]
	5	2001.5	2004 Demographic and Health Survey [DHS Direct]
	5	2007.5	2010 Demographic and Health Survey [DHS Direct]
	5	2010.5	2013-2014 MDG Endline Survey [MICS Direct]
	5	2012.5	2015-2016 Demographic and Health Survey [DHS Direct]
	5	2016.5	2019-2020 Multiple Indicator Cluster Survey [MICS Direct]
Malaysia	5	2015.5	
	2	2012.5	Vital Statistics [VR]
	1	2013.5	Vital Statistics [VR]
	2	2015.5	Vital Statistics [VR]
Maldives	25	2020.5	
	1	1998	1997 Poverty and Vulnerability Survey [Others Direct]
	5	2006.5	2009 Demographic and Health Survey [DHS Direct]
	5	2013.5	2016-2017 Demographic and Health Survey [DHS Direct]
	14	2020.5	WHO Vital Registration Data 2022 version [VR]
Mali	25	2015.5	
	5	1984.5	1987 Demographic and Health Survey [DHS Direct]
	5	1992.5	1995-1996 Demographic and Health Survey [DHS Direct]
	5	1998.5	2001 Demographic and Health Survey [DHS Direct]
	5	2003.5	2006 Demographic and Health Survey [DHS Direct]
	5	2015.5	2018 Demographic and Health Survey [DHS Direct]
Malta	58	2020.5	

Continued on next page

Table 23 – continued from previous page

Country	# obs.	Most recent obs. year	Data series name [source type]
	58	2020.5	2021 WHO Vital Registration Data 2022 version [VR]
Marshall Islands	1	2002.1	
	1	2002.1	2007 Demographic and Health Survey [Other DHS Direct]
Mauritania	25	2016.5	
	5	1978.5	1981-1982 World Fertility Survey [Other DHS Direct]
	3	1985	1990 Maternal and Child Health Survey [Others Direct]
	5	1997.5	2000-2001 Demographic and Health Survey [DHS Direct]
	1	1998.9	2003-2004 EMIP survey [Others Direct]
	1	2012.8	2013 Census [Others Direct]
	5	2012.5	2015 Multiple Indicator Cluster Survey [MICS Direct]
	5	2016.5	2019-2021 Demographic and Health Survey [DHS Direct]
Mauritius	70	2020.5	
	6	1956.5	UNPD Demographic Yearbook Data 2020 version [VR]
	64	2020.5	WHO Vital Registration Data 2022 version [VR]
Mexico	21	2016.5	
	5	1984.5	1987 Demographic and Health Survey [DHS Direct]
	5	2015.5	2018 Encuesta Nacional de la Dinamica Demografica (ENADID) [Others Direct]
	11	2016.5	Vital Registration Data From Mexico Ministry Of Health [VR]
Republic of Moldova	9	2009.5	
	5	2002.5	2005 Demographic and Health Survey [DHS Direct]
	4	2009.5	2012 Multiple Indicator Cluster Survey [MICS Direct]
Monaco	22	2011.5	
	22	2011.5	2018 WHO Vital Registration Data 2018 version neighbouring [VR]
Mongolia	16	2019.5	
	1	1993	1998 Reproductive Health Survey [Others Direct]
	5	2010.5	2013-2014 Multiple Indicator Cluster Survey [MICS Direct]
	10	2019.5	WHO Vital Registration Data 2022 version [VR]
Montenegro	36	2021.5	
	36	2021.5	2021 WHO Vital Registration Data 2022 version [VR]
Montserrat	24	2008.5	
	24	2008.5	2019 UNPD Vital Registration Data 2019 version [VR]
Morocco	27	2015.5	
	5	1977.5	1980 World Fertility Survey [Other DHS Direct]
	5	1984.5	1987 Demographic and Health Survey [DHS Direct]
	5	1989.5	1992 Demographic and Health Survey [DHS Direct]
	5	1992.5	1995 Demographic and Health Survey [DHS Direct]
	1	1994.5	1997 PAPG ENSME petit-echantillon 1997 [Others Direct]
	5	2000.5	2003-2004 Demographic and Health Survey [DHS Direct]
	1	2015.5	2018 National Survey of Population and Family Health [Others Direct]
Mozambique	15	2008.5	
	5	2000.5	2003-2004 Demographic and Health Survey [DHS Direct]
	5	2005.5	2008 Multiple Indicator Cluster Survey [MICS Direct]
	5	2008.5	2011 Demographic and Health Survey [DHS Direct]
Myanmar	10	2012.5	

Continued on next page

Table 23 – continued from previous page

Country	# obs.	Most recent obs. year	Data series name [source type]
	2	1985.5	1991 Population Change and Fertility Survey [Others Direct]
	1	1992	1997 Fertility and Reproductive Health Survey [Others Direct]
	1	1996	2001 Fertility and Reproductive Health Survey [Others Direct]
	1	2001	2007 Fertility and Reproductive Health Survey [Others Direct]
	5	2012.5	2015-2016 Demographic and Health Survey [DHS Direct]
Namibia	20	2010.5	
	5	1989.5	1992 Demographic and Health Survey [DHS Direct]
	5	1997.5	2000 Demographic and Health Survey [DHS Direct]
	5	2003.5	2006-2007 Demographic and Health Survey [DHS Direct]
	5	2010.5	2013 Demographic and Health Survey [DHS Direct]
Nauru			
Nepal	41	2016.5	
	5	1973.5	1976 World Fertility Survey [Other DHS Direct]
	1	1986	1991 Fertility and Family Planning Survey [Others Direct]
	5	1993.5	1996 Demographic and Health Survey [DHS Direct]
	5	1998.5	2001 Demographic and Health Survey [DHS Direct]
	5	2003.5	2006 Demographic and Health Survey [DHS Direct]
	5	2008.5	2011 Demographic and Health Survey [DHS Direct]
	5	2011.5	2014 Multiple Indicator Cluster Survey [MICS Direct]
	5	2013.5	2016 Demographic and Health Survey [DHS Direct]
	5	2016.5	2019 Multiple Indicator Cluster Survey [MICS Direct]
Netherlands	72	2021.5	
	72	2021.5	2021 WHO Vital Registration Data 2022 version [VR]
New Zealand	67	2016.5	
	67	2016.5	2021 WHO Vital Registration Data 2022 version [VR]
Nicaragua	17	2008.5	
	5	1994.5	1998 Demographic and Health Survey [DHS Direct]
	4	1993.5	2001 Demographic and Health Survey [DHS Direct]
	4	2003.5	2006-2007 Encuesta Nicaraguense de Demografia y Salud [Others Direct]
	4	2008.5	2011-2012 Encuesta Nicaraguense de Demografia y Salud [Others Direct]
Niger	28	2018.5	
	5	1989.5	1992 Demographic and Health Survey [DHS Direct]
	5	1995.5	1998 Demographic and Health Survey [DHS Direct]
	5	2003.5	2006 Demographic and Health Survey [DHS Direct]
	3	2007.5	2010 Child Survival and Mortality Survey New [Others Direct]
	5	2009.5	2012 Demographic and Health Survey [DHS Direct]
	5	2018.5	2021 Enquete Nationale sur la Fecondite et la Mortalite des Enfants de moins de 5 ans (ENAFEME) [Others Direct]
Nigeria	36	2017.5	
	5	1987.5	1990 Demographic and Health Survey [DHS Direct]
	5	2000.5	2003 Demographic and Health Survey [DHS Direct]
	5	2005.5	2008 Demographic and Health Survey [DHS Direct]
	5	2007.5	2010 Malaria Indicator Survey [Other DHS Direct]
	8	2011.5	2013 Demographic and Health Survey [DHS Direct]

Continued on next page

Table 23 – continued from previous page

Country	# obs.	Most recent obs. year	Data series name [source type]
	8	2017.5	2018 Demographic and Health Survey [DHS Direct]
Niue			
Norway	71	2021.5	
	71	2021.5	2021 WHO Vital Registration Data 2022 version [VR]
Oman	4	1990	
	3	1983.5	1988 Child Health Survey [Others Direct]
	1	1990	1995 Family Health Survey [Others Direct]
Pakistan	69	2016.5	
	1	1969.5	1971 Population Growth Survey I [Others Direct]
	5	1972.5	1975 World Fertility Survey [Other DHS Direct]
	1	1977	1976-1978 Population Growth Survey II [Others Direct]
	1	1986	1988 Demographic Survey [Others Direct]
	5	1987.5	1990-1991 Demographic and Health Survey [DHS Direct]
	7	1995.5	1998 Integrated Household Survey [Others Direct]
	1	1999	2000-2001 Reproductive Health and Family Planning Survey [Others Direct]
	7	1997.5	2001 Integrated Household Survey [Others Direct]
	5	2003.5	2006-2007 Demographic and Health Survey [DHS Direct]
	20	2007.5	2008 Pakistan Demographic Survey [Others Direct]
	5	2009.5	2012-2013 Demographic and Health Survey [DHS Direct]
	5	2014.5	2017-2018 Demographic and Health Survey [DHS Direct]
	5	2016.5	2018-2019 Social
Living Standard Measurement Survey [Others Direct]			
	1	1964	Population Growth Estimation Experiment [VR]
Palau	10	2002.5	
	10	2002.5	2021 WHO Vital Registration Data 2022 version [VR]
Panama	5	1972.5	
	5	1972.5	1975-1976 World Fertility Survey [Other DHS Direct]
Papua New Guinea	7	2013.5	
	1	1991	1996 Demographic and Health Survey [Other DHS Direct]
	1	2001	2006 Demographic and Health Survey [Other DHS Direct]
	5	2013.5	2016-2018 Demographic and Health Survey [DHS Direct]
Paraguay	18	2005.5	
	5	1976.5	1979 World Fertility Survey [Other DHS Direct]
	5	1987.5	1990 Demographic and Health Survey [DHS Direct]
	4	2001.5	2004 Reproductive Health Survey [Others Direct]
	4	2005.5	2008 Encuesta Nacional de Demografia y Salud Sexual y Reproductiva [Others Direct]
Peru	80	2018.5	
	5	1974.5	1977-1978 World Fertility Survey [Other DHS Direct]
	5	1983.5	1986 Demographic and Health Survey [DHS Direct]
	5	1988.5	1991-1992 Demographic and Health Survey [DHS Direct]
	5	1993.5	1996 Demographic and Health Survey [DHS Direct]

Continued on next page

Table 23 – continued from previous page

Country	# obs.	Most recent obs. year	Data series name [source type]
	5	1997.5	2000 Demographic and Health Survey [DHS Direct]
	4	2000.5	2003-2008 Demographic and Health Survey [DHS Direct]
	4	2006.5	2009 Demographic and Health Survey [DHS Direct]
	5	2007.5	2010 Demographic and Health Survey [DHS Direct]
	5	2008.5	2011 Demographic and Health Survey [DHS Direct]
	5	2009.5	2012 Demographic and Health Survey [DHS Direct]
	1	2008	2013 Demographic and Health Survey [DHS Direct]
	1	2009.5	2014 Encuesta Demografica y de Salud Familiar-ENDES Continua [DHS Direct]
	5	2011.5	2014 National Demographic and Health Survey [Other DHS Direct]
	5	2014.5	2017 Demographic and Family Health Survey [Others Direct]
	5	2015.5	2018 Demographic and Family Health Survey [Other DHS Direct]
	5	2016.5	2019 Encuesta Demografica y de Salud Familiar [Other DHS Direct]
	5	2017.5	2020 Encuesta Demografica y de Salud Familiar [Other DHS Direct]
	5	2018.5	2021 Encuesta Demografica y de Salud Familiar [Other DHS Direct]
Philippines	35	2014.5	
	5	1975.5	1978 World Fertility Survey [Other DHS Direct]
	5	1990.5	1993 Demographic and Health Survey [DHS Direct]
	5	1995.5	1998 Demographic and Health Survey [DHS Direct]
	5	2000.5	2003 Demographic and Health Survey [DHS Direct]
	5	2005.5	2008 Demographic and Health Survey [DHS Direct]
	5	2010.5	2013 National Demographic and Health Survey [DHS Direct]
	5	2014.5	2017 Demographic and Health Survey [DHS Direct]
Poland	71	2021.5	
	8	1958.5	UNPD Demographic Yearbook Data 2020 version [VR]
	63	2021.5	WHO Vital Registration Data 2022 version [VR]
Portugal	72	2021.5	
	5	1954.5	UNPD Demographic Yearbook Data 2020 version [VR]
	67	2021.5	WHO Vital Registration Data 2022 version [VR]
Qatar	30	2020.5	
	30	2020.5	2021 WHO Vital Registration Data 2022 version [VR]
Romania	65	2021.5	
	3	1958.5	UNPD Demographic Yearbook Data 2020 version [VR]
	62	2021.5	WHO Vital Registration Data 2022 version [VR]
Russian Federation	41	2020.5	
	41	2020.5	2021 WHO Vital Registration Data 2022 version [VR]
Rwanda	34	2016.5	
	5	1980.5	1983 World Fertility Survey [Other DHS Direct]
	5	1989.5	1992 Demographic and Health Survey [DHS Direct]
	4	1992.5	2000 Demographic and Health Survey [DHS Direct]
	4	2002.5	2005 Demographic and Health Survey [DHS Direct]
	3	2004.5	2007-2008 Interim Demographic and Health Survey [Other DHS Direct]
	4	2007.5	2010 Demographic and Health Survey [DHS Direct]
	4	2011.5	2014-2015 Demographic and Health Survey [DHS Direct]

Continued on next page

Table 23 – continued from previous page

Country	# obs.	Most recent obs. year	Data series name [source type]
	5	2016.5	2019-2020 Demographic and Health Survey [DHS Direct]
Saint Kitts and Nevis	30	2015.5	
	30	2015.5	2021 WHO Vital Registration Data 2022 version [VR]
Saint Lucia	54	2017.5	
	14	1968.5	UNPD Demographic Yearbook Data 2020 version [VR]
	40	2017.5	WHO Vital Registration Data 2022 version [VR]
Samoa	7	2016.5	
	1	1999	1999 Demographic and Health Survey [Others Direct]
	1	1995	2000 Demographic and Vital Statistics Survey [Others Direct]
	1	2011.4	2011 Population and Housing Census [Others Direct]
	4	2016.5	2019-2020 Multiple Indicator Cluster Survey [MICS Direct]
San Marino	3	2003.5	
	2	2003.5	WHO Vital Registration Data 2020 version [VR]
	1	1993.5	WHO Vital Registration Data 2022 version [VR]
Sao Tome and Principe	15	2015.5	
	5	2005.5	2008-2009 Demographic and Health Survey [DHS Direct]
	5	2011.5	2014 Multiple Indicator Cluster Survey [MICS Direct]
	5	2015.5	2019 Multiple Indicator Cluster Survey [MICS Direct]
Saudi Arabia	6	2017.4	
	1	1998.6	1999 Demographic Survey [Others Direct]
	1	1999.6	2000 Demographic Survey [Others Direct]
	1	2004.2	2004 Census [Others Direct]
	1	2006.6	2007 Demographic Survey [Others Direct]
	1	2013.9	2016 Demographic Survey [Others Direct]
	1	2017.4	2017 Household Health Survey [Others Direct]
Senegal	75	2016.5	
	5	1975.5	1978 World Fertility Survey [Other DHS Direct]
	5	1983.5	1986 Demographic and Health Survey [DHS Direct]
	5	1989.5	1992-1993 Demographic and Health Survey [DHS Direct]
	5	1994.5	1997 Demographic and Health Survey [DHS Direct]
	5	1996.5	1999-2000 Demographic and Health Survey [DHS Direct]
	5	2002.5	2005 Demographic and Health Survey [DHS Direct]
	5	2005.5	2008-2009 Malaria Indicator Survey [Other DHS Direct]
	5	2007.5	2010-2011 Demographic and Health Survey [DHS Direct]
	5	2009.5	2012-2013 Demographic and Health Survey [DHS Direct]
	5	2011.5	2014 Demographic and Health Survey [DHS Direct]
	5	2012.5	2015 Demographic and Health Survey [DHS Direct]
	5	2013.5	2016 Demographic and Health Survey [DHS Direct]
	5	2014.5	2017 Demographic and Health Survey [DHS Direct]
	5	2015.5	2018 Demographic and Health Survey [DHS Direct]
	5	2016.5	2019 Demographic and Health Survey [DHS Direct]
Serbia	37	2021.5	
	37	2021.5	2021 WHO Vital Registration Data 2022 version [VR]
Seychelles	37	2017.5	

Continued on next page

Table 23 – continued from previous page

Country	# obs.	Most recent obs. year	Data series name [source type]
	37	2017.5	2021 WHO Vital Registration Data 2022 version [VR]
Sierra Leone	11	2016.5	
	1	1992	1992 DSMS [Others Direct]
	5	2010.5	2013 Demographic and Health Survey [DHS Direct]
	5	2016.5	2019 Demographic and Health Survey [DHS Direct]
Singapore	71	2020.5	
	5	1954.5	UNPD Demographic Yearbook Data 2020 version [VR]
	66	2020.5	WHO Vital Registration Data 2022 version [VR]
Slovakia	40	2021.5	
	40	2021.5	2021 WHO Vital Registration Data 2022 version [VR]
Slovenia	40	2021.5	
	40	2021.5	2021 WHO Vital Registration Data 2022 version [VR]
Solomon Islands	6	2012.5	
	1	2002	2007 Demographic and Health Survey [Other DHS Direct]
	5	2012.5	2015 Demographic and Health Survey [Other DHS Direct]
Somalia	4	2003.5	
	4	2003.5	2006 Multiple Indicator Cluster Survey [MICS Direct]
South Africa	55	2021.5	
	5	1995.5	1998 Demographic and Health Survey [DHS Direct]
	1	2006.7	2007 Community Survey [Others Direct]
	5	2013.5	2016 Demographic and Health Survey [DHS Direct]
	44	2021.5	Rapid Mortality Surveillance (Preliminary) [VR]
South Sudan			
Spain	71	2020.5	
	71	2020.5	2021 WHO Vital Registration Data 2022 version [VR]
Sri Lanka	79	2014.5	
	5	1972.5	1975 World Fertility Survey [Other DHS Direct]
	5	1984.5	1987 Demographic and Health Survey [DHS Direct]
	6	1990.5	1993 Demographic and Health Survey [Other DHS Direct]
	6	1997.5	2000 Demographic and Health Survey [Other DHS Direct]
	6	2003.5	2006 Demographic and Health Survey [Other DHS Direct]
	5	2013.5	2016 Demographic and Health Survey [Other DHS Direct]
	46	2014.5	WHO Vital Registration Data 2022 version [VR]
Saint Vincent and the Grenadines	55	2017.5	
	10	1964.5	UNPD Demographic Yearbook Data 2020 version [VR]
	45	2017.5	WHO Vital Registration Data 2022 version [VR]
State of Palestine	25	2016.5	
	1	1992.5	1995 Demographic Survey [Others Direct]
	1	1997.5	2000 Health Survey [Others Direct]
	1	2002.3	2004 Demographic and Health Survey [Other DHS Direct]
	6	2005	2006 Pan Arab Project for Family Health Family Health Survey [Others Direct]
	5	2008.1	2010 Multiple Indicator Cluster Survey-Family Health Survey [MICS Direct]
	1	2008	2011 Palestinian Family Survey [Others Direct]

Continued on next page

Table 23 – continued from previous page

Country	# obs.	Most recent obs. year	Data series name [source type]
	5	2011.5	2014 Multiple Indicator Cluster Survey [MICS Direct]
	5	2016.5	2019-2020 Multiple Indicator Cluster Survey [MICS Direct]
Sudan	24	2011.5	
	5	1975.5	1978-1979 World Fertility Survey [Other DHS Direct]
	5	1986.5	1989-1990 Demographic and Health Survey [DHS Direct]
	4	1989.5	1992 Pan Arab Project for Child Development Maternal and Child Health Survey [Others Direct]
	1	1995	1999 Safe Motherhood Survey [Others Direct]
	4	2007.5	2010 Multiple Indicator Cluster Survey [MICS Direct]
	5	2011.5	2014 Multiple Indicator Cluster Survey [MICS Direct]
Suriname	3	2015.5	
	3	2015.5	2018 Multiple Indicator Cluster Survey [MICS Direct]
Swaziland	14	2011.5	
	4	2003.5	2006-2007 Demographic and Health Survey [DHS Direct]
	5	2007.5	2010 Multiple Indicator Cluster Survey [MICS Direct]
	5	2011.5	2014 Multiple Indicator Cluster Survey [MICS Direct]
Sweden	71	2021.5	
	71	2021.5	2021 WHO Vital Registration Data 2022 version [VR]
Switzerland	71	2021.5	
	71	2021.5	2021 WHO Vital Registration Data 2022 version [VR]
Syria	16	1999	
	5	1975.5	1978 World Fertility Survey [Other DHS Direct]
	5	1990.5	1993 Pan Arab Project for Child Development Maternal and Child Health Survey [Others Direct]
	6	1999	2001 Pan Arab Project for Family Health Family Health Survey [Others Direct]
Tajikistan	5	2014.5	
	5	2014.5	2017 Demographic and Health Survey [DHS Direct]
Tanzania	30	2012.5	
	5	1988.5	1991-1992 Demographic and Health Survey [DHS Direct]
	5	1993.5	1996 Demographic and Health Survey [DHS Direct]
	5	1996.5	1999 Demographic and Health Survey [DHS Direct]
	5	2001.5	2004-2005 Demographic and Health Survey [DHS Direct]
	5	2006.5	2010 Demographic and Health Survey [DHS Direct]
	5	2012.5	2015-2016 Demographic and Health Survey [DHS Direct]
Thailand	14	1991	
	1	1975	1974-1975 Survey of Population Change [Others Direct]
	5	1972.5	1975 World Fertility Survey [Other DHS Direct]
	1	1985	1985-1986 Survey of Population Change [Others Direct]
	5	1984.5	1987 Demographic and Health Survey [DHS Direct]
	2	1991	1989 Survey of Population Change [Others Direct]
Timor Leste	6	2006.5	
	1	1999	2003 Demographic and Health Survey [Other DHS Direct]
	5	2006.5	2009-2010 Demographic and Health Survey [DHS Direct]
Togo	20	2014.5	
	5	1985.5	1988 Demographic and Health Survey [DHS Direct]

Continued on next page

Table 23 – continued from previous page

Country	# obs.	Most recent obs. year	Data series name [source type]
	5	1995.5	1998 Demographic and Health Survey [DHS Direct]
	5	2010.5	2013 Demographic and Health Survey [DHS Direct]
	5	2014.5	2017 Multiple Indicator Cluster Survey [MICS Direct]
Tonga	3	2011.5	
	1	2007.8	2012 National Demographic and Health Survey [DHS Direct]
	2	2011.5	2019 Multiple Indicator Cluster Survey [MICS Direct]
Trinidad and Tobago	10	1984.5	
	5	1974.5	1977 World Fertility Survey [Other DHS Direct]
	5	1984.5	1987 Demographic and Health Survey [DHS Direct]
Tunisia	19	2017.5	
	5	1985.5	1988 Demographic and Health Survey [DHS Direct]
	5	1992.4	1994 Pan Arab Project for Child Development Maternal and Child Health Survey [Others Direct]
	5	1999	2001 Pan Arab Project for Family Health Family Health Survey [Others Direct]
	1	2009.6	2011-2012 Multiple Indicator Cluster Survey [MICS Direct]
	3	2017.5	WHO Vital Registration Data 2022 version [VR]
Turkey	38	2019.5	
	5	1975.5	1978 World Fertility Survey [Other DHS Direct]
	5	1990.5	1993 Demographic and Health Survey [DHS Direct]
	5	1995.5	1998 Demographic and Health Survey [DHS Direct]
	5	2000.5	2003-2004 Demographic and Health Survey [Other DHS Direct]
	1	2003.8	2008 Turkey Demographic and Health Survey [Other DHS Direct]
	6	2010.5	2013 Demographic and Health Survey [Other DHS Direct]
	11	2019.5	WHO Vital Registration Data 2022 version [VR]
Turkmenistan	6	2016.5	
	1	1995.6	2000 Demographic and Health Survey [DHS Direct]
	5	2016.5	2019 Multiple Indicator Cluster Survey [MICS Direct]
Turks and Caicos Islands	23	2016.5	
	23	2016.5	2019 UNPD Vital Registration Data 2019 version [VR]
Tuvalu	14	2016.5	
	1	2000.3	2002 Census [Others Direct]
	1	2002.7	2007 Demographic and Health Survey [Other DHS Direct]
	3	2016.5	2019-2020 Multiple Indicator Cluster Survey [MICS Direct]
	9	2005.5	WHO Vital Registration Data 2022 version [VR]
Uganda	35	2013.5	
	5	1985.5	1988-1989 Demographic and Health Survey [DHS Direct]
	5	1992.5	1995 Demographic and Health Survey [DHS Direct]
	5	1997.5	2000-2001 Demographic and Health Survey [DHS Direct]
	5	2003.5	2006 Demographic and Health Survey [DHS Direct]
	5	2007.5	2009-2010 Malaria Indicator Survey [Other DHS Direct]
	5	2008.5	2011 Demographic and Health Survey [DHS Direct]
	5	2013.5	2016 Demographic and Health Survey [DHS Direct]
Ukraine	7	2004.5	

Continued on next page

Table 23 – continued from previous page

Country	# obs.	Most recent obs. year	Data series name [source type]
	1	1995	1999 Reproductive Health Survey [Others Direct]
	5	2004.5	2007 Demographic and Health Survey [DHS Direct]
	1	1989.5	2012 Multiple Indicator Cluster Survey [MICS Direct]
United Arab Emirates	7	2014.5	
	1	1991	1995 Family Health Survey [Others Direct]
	6	2014.5	Vital Registration Data published in UAE IN FIGURES 2014 [VR]
United Kingdom	71	2020.5	
	71	2020.5	2021 WHO Vital Registration Data 2022 version [VR]
United States of America	70	2019.5	
	70	2019.5	2021 WHO Vital Registration Data 2022 version [VR]
Uruguay	62	2020.5	
	62	2020.5	2021 WHO Vital Registration Data 2022 version [VR]
Uzbekistan	1	1997	
	1	1997	2002 Demographic and Health Survey [Other DHS Direct]
Vanuatu	1	2009	
	1	2009	2013 Demographic and Health Survey [Other DHS Direct]
Venezuela (Bolivarian Republic of)	31	2014.5	
	5	1974.5	1977 World Fertility Survey [Other DHS Direct]
	1	1995.5	1998 National Population and Family Survey [Others Direct]
	25	2014.5	WHO Vital Registration Data 2022 version [VR]
Vietnam	20	2014.8	
	1	1988.5	1994 Intercensal Demographic Survey [Others Direct]
	5	1994.5	1997 Demographic and Health Survey [DHS Direct]
	5	1999.5	2002 Demographic and Health Survey [DHS Direct]
	1	2011.8	2012 Population Change and Family Planning Survey [Others Direct]
	5	2010.5	2013-2014 Multiple Indicator Cluster Survey [MICS Direct]
	1	2012.8	2013 Population Change and Family Planning Survey [Others Direct]
	1	2013.8	2014 Intercensal Population and Housing Survey [Others Direct]
	1	2014.8	2015 Population Change and Family Planning Survey [Others Direct]
Yemen	28	2010.5	
	3	1976.5	1979 World Fertility Survey [Other DHS Direct]
	5	1988.5	1991-1992 Demographic and Health Survey [DHS Direct]
	5	1994.5	1997 Demographic and Health Survey [DHS Direct]
	5	2000.5	2003 Pan Arab Project for Family Health Family Health Survey [Others Direct]
	5	2003.5	2006 Multiple Indicator Cluster Survey [MICS Direct]
	5	2010.5	2013 Demographic and Health Survey [DHS Direct]
Zambia	31	2015.5	
	5	1989.5	1992 Demographic and Health Survey [DHS Direct]
	5	1993.5	1996-1997 Demographic and Health Survey [DHS Direct]
	5	1998.5	2001-2002 Demographic and Health Survey [DHS Direct]
	6	2004.5	2007 Demographic and Health Survey [DHS Direct]

Continued on next page

Table 23 – continued from previous page

Country	# obs.	Most recent obs. year	Data series name [source type]
	5	2010.5	2013-2014 Demographic and Health Survey [DHS Direct]
	5	2015.5	2018 Demographic and Health Survey [DHS Direct]
Zimbabwe	41	2016.5	
	5	1985.5	1988-1989 Demographic and Health Survey [DHS Direct]
	5	1991.5	1994 Demographic and Health Survey [DHS Direct]
	1	1997	1997 Inter-censal Demographic Survey [Others Direct]
	5	1996.5	1999 Demographic and Health Survey [DHS Direct]
	5	2006.5	2009 Multiple Indicator Cluster Survey [MICS Direct]
	5	2007.5	2010-2011 Demographic and Health Survey [DHS Direct]
	5	2011.5	2014 Multiple Indicator Cluster Survey [MICS Direct]
	5	2012.5	2015 Demographic and Health Survey [DHS Direct]
	5	2016.5	2019 Multiple Indicator Cluster Survey [MICS Direct]

Table 24: **Sex ratio data sources for age group 0–4, by country.** For each country, the total number of observations and the most recent reference year are shown after the country name. For each country-specific data series, the number of observations and the most recent reference year within that series is shown before each data series name. The source type that each data series falls in is shown in parentheses after each data series name.

Country	# obs.	Most recent obs. year	Data series name [source type]
Afghanistan	8	2006.6	
	3	1969.4	1972 National Demographic and Family Guidance Survey [Others Indirect]
	1	2004.3	2007-2008 National Risk and Vulnerability Assessment Survey [Others Indirect]
	4	2006.6	2010-2011 Multiple Indicator Cluster Survey [MICS Indirect]
Albania	7	1997.4	
	4	1995.3	2000 Multiple Indicator Cluster Survey [MICS Indirect]
	3	1997.4	2005 Multiple Indicator Cluster Survey [MICS Indirect]
Algeria	6	2002.9	
	1	1993.8	2000 Multiple Indicator Cluster Survey [MICS Indirect]
	5	2002.9	2006 Multiple Indicator Cluster Survey [MICS Indirect]
Andorra	20	2014.5	
	20	2014.5	2018 WHO Vital Registration Data 2018 version [VR]
Angola	15	2003.7	
	5	1992	1996 Multiple Indicator Cluster Survey [MICS Indirect]
	4	1997	2001 Multiple Indicator Cluster Survey [MICS Indirect]
	5	2002.7	2006-2007 Malaria Indicator Survey [Others Indirect]
	1	2003.7	2008-2009 Household Incomes and Expenditures Survey IBEP [Others Indirect]
Anguilla			
Antigua and Barbuda			
Argentina			
Armenia			
Australia			
Austria			
Azerbaijan	4	1995.6	
	4	1995.6	2000 Multiple Indicator Cluster Survey [MICS Indirect]
Bahamas			
Bahrain			
Bangladesh	13	2008.3	
	4	1967.9	1974 Retrospective Fertility and Mortality Survey (UN SA) [Others Indirect]
	5	2005.1	2009 Multiple Indicator Cluster Survey [Others Indirect]
	4	2008.3	2012-2013 Multiple Indicator Cluster Survey [MICS Indirect]
Barbados			
Belarus			
Belgium			
Belize	8	2006.8	
	4	2001.5	2006 Multiple Indicator Cluster Survey [MICS Indirect]

Continued on next page

Table 24 – continued from previous page

Country	# obs.	Most recent obs. year	Data series name [source type]
	4	2006.8	2011 Multiple Indicator Cluster Survey [MICS Indirect]
Benin	5	1997.6	
	5	1997.6	2002 Census [Census Indirect]
Bhutan	9	2005.7	
	5	1980.6	1984 Demographic Sample Survey [Others Indirect]
	4	2005.7	2010 Multiple Indicator Cluster Survey [MICS Indirect]
Bolivia (Plurinational State of)	14	1996.1	
	5	1972	1975 EDEN [Others Indirect]
	5	1976.9	1980 National Demographic Survey [Others Indirect]
	4	1996.1	2000 Multiple Indicator Cluster Survey [MICS Indirect]
Bosnia and Herzegovina			
Botswana	4	2007.2	
	1	1996	2000 Multiple Indicator Cluster Survey [MICS Indirect]
	1	1997.2	2001 Census [Census Indirect]
	1	2002	2006 Demographic Survey [Others Indirect]
	1	2007.2	2011 Census [Census Indirect]
Brazil			
British Virgin Islands			
Brunei			
Bulgaria			
Burkina Faso	15	2015.7	
	4	2002	2006 Multiple Indicator Cluster Survey [MICS Indirect]
	5	2010.4	2014 Malaria Indicator Survey [Others Indirect]
	6	2015.7	2017-2018 Malaria Indicator Survey [Others Indirect]
Burundi	12	2011.5	
	3	1995.7	2000 Multiple Indicator Cluster Survey [MICS Indirect]
	3	2001.5	2005 Multiple Indicator Cluster Survey [MICS Indirect]
	1	2011.5	2012 Enquete menages pour le suivi et lÃevaluation de lÃimpact de lÃappui au systeme de remboursement du Paquet Minimum des Services de sante [Others Indirect]
	5	2007	2012 Malaria Indicator Survey [Others Indirect]
Cambodia	8	2002.2	
	5	1994.3	1998 Census [Census Indirect]
	3	2002.2	2004 Inter-censal Population Survey [Others Indirect]
Cameroon	4	1996.2	
	4	1996.2	2000 Multiple Indicator Cluster Survey [MICS Indirect]
Canada			
Cape Verde	5	2005.8	
	5	2005.8	2010 Census [Census Indirect]
Central African Republic	12	2006.4	
	4	1996.4	2000 Multiple Indicator Cluster Survey [MICS Indirect]

Continued on next page

Table 24 – continued from previous page

Country	# obs.	Most recent obs. year	Data series name [source type]
	4	2002.3	2006 Multiple Indicator Cluster Survey [MICS Indirect]
	4	2006.4	2010 Multiple Indicator Cluster Survey [MICS Indirect]
Chad	8	2005.8	
	4	1996.1	2000 Multiple Indicator Cluster Survey [MICS Indirect]
	4	2005.8	2010 Multiple Indicator Cluster Survey [MICS Indirect]
Chile			
China	13	2011	
	1	1986	1987 Population Sample Survey [Others Indirect]
	12	2011	Sample Vital Registration [VR]
Colombia			
Comoros			
Congo	5	2004.9	
	5	2004.9	2009 AIDS Indicator Survey [Others Indirect]
Democratic Republic of the Congo	8	2005.8	
	4	1997	2001 Multiple Indicator Cluster Survey [MICS Indirect]
	4	2005.8	2010 Multiple Indicator Cluster Survey [MICS Indirect]
Cook Islands	5	2002.7	
	5	2002.7	2006 Census [Census Indirect]
Costa Rica			
Cote d'Ivoire			
Croatia			
Cuba			
Cyprus			
Czech Republic			
Denmark			
Djibouti	4	2002	
	4	2002	2006 Multiple Indicator Cluster Survey [MICS Indirect]
Dominica			
Dominican Republic	9	1996.2	
	5	1977.5	1981 Census [Census Indirect]
	4	1996.2	2000 Multiple Indicator Cluster Survey [MICS Indirect]
Ecuador			
Egypt	5	2011.7	
	5	2011.7	2015 Health Indicator Survey [Others Indirect]
El Salvador	4	1978.6	
	4	1978.6	1985 Demographic and Health Survey [Others Indirect]
Equatorial Guinea			
Eritrea			
Estonia			
Ethiopia			
Federated States of Micronesia	4	2006.5	

Continued on next page

Table 24 – continued from previous page

Country	# obs.	Most recent obs. year	Data series name [source type]
	4	2006.5	2010 Census [Census Indirect]
Fiji	10	2003.5	
	5	1973.1	1976 Census [Census Indirect]
	5	2003.5	2007 Census [Census Indirect]
Finland			
France			
Gabon			
The Gambia	13	2001.9	
	4	1995.5	2000 Multiple Indicator Cluster Survey [MICS Indirect]
	4	2001.9	2005-2006 Multiple Indicator Cluster Survey [MICS Indirect]
Georgia	4	2000.7	
	4	2000.7	2005 Multiple Indicator Cluster Survey [MICS Indirect]
Germany			
Ghana	19	2015.7	
	4	2002	2006 Multiple Indicator Cluster Survey [MICS Indirect]
	5	2006.9	2010 Census [Census Indirect]
	5	2012.5	2016 Malaria Indicator Survey [Others Indirect]
	5	2015.7	2019 Malaria Indicator Survey [Others Indirect]
Greece			
Grenada			
Guatemala	26	2014	
	4	1974.4	1981 Census [Census Indirect]
	4	1980.7	1987 Enc. Nacional SocioDemografica [Others Indirect]
	4	1985.1	1989 Enc. Nacional Sociodemografica [Others Indirect]
	4	1989.8	1994 Census [Census Indirect]
	5	1998.5	2002 Census [Census Indirect]
	5	2014	2018 Census [Census Indirect]
Guinea	19	2017.1	
	5	1992.3	1996 Census [Census Indirect]
	5	1999	2003 Multiple Indicator Cluster Survey [Others Indirect]
	5	2009.7	2014 Census [Census Indirect]
	4	2017.1	2021 Malaria Indicator Survey [Others Indirect]
Guinea-Bissau	8	2000.9	
	4	1995.8	2000 Multiple Indicator Cluster Survey [MICS Indirect]
	4	2000.9	2006 Multiple Indicator Cluster Survey [MICS Indirect]
Guyana	8	2001.6	
	4	1996	2000-2001 Multiple Indicator Cluster Survey [MICS Indirect]
	4	2001.6	2006 Multiple Indicator Cluster Survey [MICS Indirect]
Haiti			
Honduras	4	1979.4	
	4	1979.4	1983 National Demographic Survey [Others Indirect]
Hungary			
Iceland			
India	18	2007.4	
	4	1994.5	1999 District Level Household Survey [Others Indirect]

Continued on next page

Table 24 – continued from previous page

Country	# obs.	Most recent obs. year	Data series name [source type]
	4	1999.3	2004 District Level Household Survey [Others Indirect]
	5	2003.6	2008 District Level Household Survey [Others Indirect]
	5	2007.4	2011 Census [Census Indirect]
Indonesia	9	2006.4	
	5	1986.9	1990 Census [Census Indirect]
	4	2006.4	2010 Census [Census Indirect]
Iran (Islamic Republic of)	10	2003.9	
	5	2003.1	2006 Census [Census Indirect]
	5	2003.9	2011 Census [Census Indirect]
Iraq	5	1986	
	5	1986	1990 Immunization, Diarrhoeal Disease, Maternal and Child Mortality Survey [Others Indirect]
Ireland			
Israel			
Italy			
Jamaica	4	2001	
	4	2001	2005 Multiple Indicator Cluster Survey [MICS Indirect]
Japan			
Jordan	20	1987.5	
	5	1970.9	1972 Jordan Fertility Survey [Others Indirect]
	5	1978.5	1981 Jordan Demographic Survey [Others Indirect]
	5	1984.9	1988 EPI/CDD and Child Mortality Survey [Others Indirect]
	5	1987.5	1990 EPI/CDD and Child Mortality Survey [Others Indirect]
Kazakhstan	12	2010.6	
	4	2000.9	2006 Multiple Indicator Cluster Survey [MICS Indirect]
	4	2005.9	2010-2011 Multiple Indicator Cluster Survey [MICS Indirect]
	4	2010.6	2015 Multiple Indicator Cluster Survey [MICS Indirect]
Kenya	8	2016.9	
	1	1985.2	1989 Census [Census Indirect]
	1	1995.2	1999 Census [Census Indirect]
	4	1996	2000 Multiple Indicator Cluster Survey [MICS Indirect]
	1	2011.3	2015 Malaria Indicator Survey [Others Indirect]
	1	2016.9	2020 Malaria Indicator Survey [Others Indirect]
Kiribati	15	2006.9	
	5	1991.9	1995 Census [Census Indirect]
	5	1997	2000 Census [Census Indirect]
	5	2006.9	2010 Census [Census Indirect]
Democratic People's Republic of Korea			
Republic of Korea			
Kosovo			
Kuwait	5	1987	
	5	1987	1987 Child Health Survey [Others Indirect]
Kyrgyzstan	4	2001.2	

Continued on next page

Table 24 – continued from previous page

Country	# obs.	Most recent obs. year	Data series name [source type]
	4	2001.2	2005-2006 Multiple Indicator Cluster Survey [MICS Indirect]
Lao People's Democratic Republic			
Latvia			
Lebanon	11	2006.6	
	5	1992.9	1996 Population and Housing Survey [Others Indirect]
	1	1996.6	2000 Multiple Indicator Cluster Survey [MICS Indirect]
	5	2006.6	2009 Multiple Indicator Cluster Survey [Others Indirect]
Lesotho	6	1996.1	
	1	1967.5	1971 Demographic Survey [Others Indirect]
	4	1995.6	2000 Multiple Indicator Cluster Survey [MICS Indirect]
	1	1996.1	2001 Demographic Survey [Others Indirect]
Liberia	10	2011.9	
	5	2006.9	2011 Malaria Indicator Survey [Others Indirect]
	5	2011.9	2016 Malaria Indicator Survey [Others Indirect]
Libya			
Lithuania			
Luxembourg			
Macedonia			
Madagascar	14	2011.4	
	4	1996.2	2000 Multiple Indicator Cluster Survey [MICS Indirect]
	5	2006.9	2013 Malaria Indicator Survey [Others Indirect]
	5	2011.4	2016 Malaria Indicator Survey [Others Indirect]
Malawi	10	2014.4	
	1	1978.4	1982 Demographic Survey [Others Indirect]
	1	1982.5	1987 Census [Census Indirect]
	1	1994	1998 Census [Census Indirect]
	1	2009.7	2014 Malaria Indicator Survey [Others Indirect]
	5	2012.9	2017 Malaria Indicator Survey [Others Indirect]
	1	2014.4	2018 Census [Census Indirect]
Malaysia			
Maldives			
Mali	5	2017.3	
	5	2017.3	2021 Malaria Indicator Survey [Others Indirect]
Malta			
Marshall Islands			
Mauritania	4	2003.1	
	4	2003.1	2007 Multiple Indicator Cluster Survey [MICS Indirect]
Mauritius			
Mexico			
Republic of Moldova			
Monaco			
Mongolia	22	2005.8	

Continued on next page

Table 24 – continued from previous page

Country	# obs.	Most recent obs. year	Data series name [source type]
	4	1996	2000 Multiple Indicator Cluster Survey [MICS Indirect]
	5	1999.5	2003 Reproductive Health Survey [Others Indirect]
	4	2001.2	2005 Multiple Indicator Cluster Survey [MICS Indirect]
	5	2004.5	2008 Reproductive Health Survey [Others Indirect]
	4	2005.8	2010 Multiple Indicator Cluster Survey [MICS Indirect]
Montenegro			
Montserrat			
Morocco			
Mozambique	4	2013.2	
	1	2005.1	2009 AIDS Indicator Survey [Others Indirect]
	1	2010.5	2015 AIDS Indicator Survey [Others Indirect]
	1	2012.5	2017 Census [Census Indirect]
	1	2013.2	2018 Malaria Indicator Survey [Others Indirect]
Myanmar			
Namibia	1	2007.1	
	1	2007.1	2011 Census [Census Indirect]
Nauru			
Nepal			
Netherlands			
New Zealand			
Nicaragua	14	2000.6	
	5	1981.3	1985 Enc.Socio-Demografica Nicaraguense [Others Indirect]
	5	1990.3	1995 Census [Census Indirect]
	4	2000.6	2005 Census [Census Indirect]
Niger	4	1996	
	4	1996	2000 Multiple Indicator Cluster Survey [MICS Indirect]
Nigeria	4	2006.6	
	4	2006.6	2011 Multiple Indicator Cluster Survey [MICS Indirect]
Niue			
Norway			
Oman			
Pakistan	10	1993.2	
	5	1992	1996 Integrated Household Survey [Others Indirect]
	5	1993.2	1998 Census [Census Indirect]
Palau			
Panama			
Papua New Guinea	5	1995.5	
	5	1995.5	2000 Census [Census Indirect]
Paraguay			
Peru			
Philippines			
Poland			
Portugal			
Qatar			
Romania			

Continued on next page

Table 24 – continued from previous page

Country	# obs.	Most recent obs. year	Data series name [source type]
Russian Federation			
Rwanda	4	2014.4	
	1	1988.1	1991 Census [Census Indirect]
	1	2009.3	2012 Census [Census Indirect]
	1	2009.8	2013 Malaria Indicator Survey [Others Indirect]
	1	2014.4	2017 Malaria Indicator Survey [Others Indirect]
Saint Kitts and Nevis			
Saint Lucia			
Samoa	5	2013.1	
	5	2013.1	2016 Population and Housing Census [Census Indirect]
San Marino			
Sao Tome and Principe	4	1996.1	
	4	1996.1	2000 Multiple Indicator Cluster Survey [MICS Indirect]
Saudi Arabia	4	1985	
	4	1985	1987 Child Health Survey [Others Indirect]
Senegal	10	2016.8	
	5	2003	2006 Malaria Indicator Survey [Others Indirect]
	5	2016.8	2020-2021 Malaria Indicator Survey [Others Indirect]
Serbia			
Seychelles			
Sierra Leone	22	2011.7	
	4	1995.8	2000 Multiple Indicator Cluster Survey [MICS Indirect]
	4	2000.9	2005-2006 Multiple Indicator Cluster Survey [MICS Indirect]
	4	2006.3	2010 Multiple Indicator Cluster Survey [MICS Indirect]
	5	2011.1	2015 Census [Census Indirect]
	5	2011.7	2016 Malaria Indicator Survey [Others Indirect]
Singapore			
Slovakia			
Slovenia			
Solomon Islands			
Somalia	1	1999.1	
	1	1999.1	1999 Multiple Indicator Cluster Survey [MICS Indirect]
South Africa			
South Sudan	5	2003.1	
	5	2003.1	2008 Census [Census Indirect]
Spain			
Sri Lanka			
Saint Vincent and the Grenadines			
State of Palestine	4	2011.6	
	4	2011.6	2017 Census [Census Indirect]
Sudan	9	2003.4	
	4	1986.5	1989-1990 Demographic and Health Survey [Others Indirect]

Continued on next page

Table 24 – continued from previous page

Country	# obs.	Most recent obs. year	Data series name [source type]
	5	2003.4	2008 Census [Census Indirect]
Suriname	8	2001.5	
	4	1995.2	1999-2000 Multiple Indicator Cluster Survey [MICS Indirect]
	4	2001.5	2006 Multiple Indicator Cluster Survey [MICS Indirect]
Swaziland	4	1995.9	
	4	1995.9	1999-2000 Multiple Indicator Cluster Survey [MICS Indirect]
Sweden			
Switzerland			
Syria	8	2001.8	
	4	1986.6	1990 EPI/CDD and Child Mortality Survey [Others Indirect]
	4	2001.8	2006 Multiple Indicator Cluster Survey [MICS Indirect]
Tajikistan	8	2001	
	4	1996.1	2000 Multiple Indicator Cluster Survey [MICS Indirect]
	4	2001	2005 Multiple Indicator Cluster Survey [MICS Indirect]
Tanzania	4	2013.4	
	1	1984.4	1988 Census [Census Indirect]
	1	1998	2002 Census [Census Indirect]
	1	2008.2	2012 Census [Census Indirect]
	1	2013.4	2017 Malaria Indicator Survey [Others Indirect]
Thailand	8	2007.4	
	4	2000.9	2005-2006 Multiple Indicator Cluster Survey [MICS Indirect]
	4	2007.4	2012-2013 Multiple Indicator Cluster Survey [MICS Indirect]
Timor Leste	6	2007	
	1	1999.6	2002 Multiple Indicator Cluster Survey [Others Indirect]
	5	2007	2010 Census [Census Indirect]
Togo	21	2013.8	
	3	1955.4	1958 Census [Census Indirect]
	4	2001.8	2006 Multiple Indicator Cluster Survey [MICS Indirect]
	5	2006.6	2010 Census [Census Indirect]
	4	2006.1	2010 Multiple Indicator Cluster Survey [MICS Indirect]
	5	2013.8	2017 Malaria Indicator Survey [Others Indirect]
Tonga	5	2010.2	
	5	2010.2	2016 Census [Census Indirect]
Trinidad and Tobago	8	2001.2	
	4	1995.2	2000 Multiple Indicator Cluster Survey [MICS Indirect]
	4	2001.2	2006 Multiple Indicator Cluster Survey [MICS Indirect]
Tunisia	5	2001.4	
	5	2001.4	2004 Census [Census Indirect]
Turkey	10	1994.8	
	5	1985.8	1989 Demographic Survey [Others Indirect]
	5	1994.8	2000 Census [Census Indirect]
Turkmenistan	4	2001.6	
	4	2001.6	2006 Multiple Indicator Cluster Survey [MICS Indirect]

Continued on next page

Table 24 – continued from previous page

Country	# obs.	Most recent obs. year	Data series name [source type]
Turks and Caicos Islands			
Tuvalu	5	2009.4	
	5	2009.4	2012 Census [Census Indirect]
Uganda	8	2014.8	
	1	1998.1	2002 Census [Census Indirect]
	5	2009.7	2014 Census [Census Indirect]
	1	2010.9	2014-2015 Malaria Indicator Survey [Others Indirect]
	1	2014.8	2018-2019 Malaria Indicator Survey [Others Indirect]
Ukraine			
United Arab Emirates	5	1987	
	5	1987	1987 Child Health Survey [Others Indirect]
United Kingdom			
United States of America			
Uruguay			
Uzbekistan	8	2001.4	
	4	1995.9	2000 Multiple Indicator Cluster Survey [MICS Indirect]
	4	2001.4	2006 Multiple Indicator Cluster Survey [MICS Indirect]
Vanuatu			
Venezuela (Bolivarian Republic of)			
Vietnam	4	1995.3	
	4	1995.3	2000 Multiple Indicator Cluster Survey [MICS Indirect]
Yemen			
Zambia	1	2006.5	
	1	2006.5	2010 Census [Census Indirect]
Zimbabwe	1	1985.6	
	1	1985.6	1987 Inter-censal Demographic Survey [Others Indirect]

Table 25: **Sex ratio data sources for age group 5–9, by country.** For each country, the total number of observations and the most recent reference year are shown after the country name. For each country-specific data series, the number of observations and the most recent reference year within that series is shown before each data series name. The source type that each data series falls in is shown in parentheses after each data series name.

Country	# obs.	Most recent obs. year	Data series name [source type]
Afghanistan	42	2015.2	
	3	1973	1972 National Demographic and Family Guidance Survey [Others Direct]
	3	1979	1979 Census [Others Direct]
	3	2008.1	2010 Afghanistan Mortality Survey (AMS) Excluding South Zone (Household data) [Other DHS Direct]
	18	2009	2010 Afghanistan Mortality Survey (AMS) [DHS Direct]
	9	2013.4	2015 Demographic and Health Survey [DHS Direct]
	6	2015.2	2018 Afghanistan Health Survey 2018 [Others Direct]
Albania	77	2021.5	
	2	1996.7	2002 Reproductive Health Survey [Others Direct]
	3	2002.7	2008-2009 Demographic and Health Survey [DHS Direct]
	3	2011.1	2011 Census [Others Direct]
	3	2011.7	2018 Demographic and Health Survey [DHS Direct]
	66	2021.5	WHO Vital Registration (recalculated - unadjusted) [VR]
Algeria	72	2020.5	
	6	1989	1992 Pan Arab Project for Child Development Maternal and Child Health Survey [Others Direct]
	3	1996	2002 Pan Arab Project for Family Health Family Health Survey [Others Direct]
	63	2020.5	WHO Vital Registration (recalculated - unadjusted) [VR]
Andorra	10	2018.5	
	10	2018.5	WHO Vital Registration (recalculated - unadjusted) [VR]
Angola	12	2014.3	
	12	2014.3	2015-2016 Inquerito de Indicadores Multiplos e de Saude [DHS Direct]
Anguilla			
Antigua and Barbuda	18	2016.5	
	18	2016.5	WHO Vital Registration (recalculated - unadjusted) [VR]
Argentina	96	2020.5	
	96	2020.5	WHO Vital Registration (recalculated - unadjusted) [VR]
Armenia	108	2021.5	
	3	1994.7	2000 Demographic and Health Survey [DHS Direct]
	3	1999.7	2005 Demographic and Health Survey [DHS Direct]
	3	2004.7	2010 Demographic and Health Survey [DHS Direct]
	99	2021.5	WHO Vital Registration (recalculated - unadjusted) [VR]
Australia	153	2020.5	
	153	2020.5	WHO/Human Mortality Database Vital Registration (recalculated - unadjusted) [VR]
Austria	153	2020.5	
	153	2020.5	WHO/Human Mortality Database Vital Registration (recalculated - unadjusted) [VR]

Continued on next page

Table 25 – continued from previous page

Country	# obs.	Most recent obs. year	Data series name [source type]
Azerbaijan	92	2021.5	
	3	2005	2011 Azerbaijan National Demographic and Health Survey [Other DHS Direct]
	87	2021.5	Recalculated based on WHO - adjusted for incompleteness [VR]
Bahamas	86	2020.5	
	86	2020.5	Recalculated based on WHO/UNPD - adjusted for incompleteness [VR]
Bahrain	93	2019.5	
	93	2019.5	WHO/UNPD Vital Registration (recalculated - unadjusted) [VR]
Bangladesh	261	2020.5	
	3	1964	1962-1965 Population Growth Estimation Experiment [Others Direct]
	3	1974.5	1974 Retrospective Fertility and Mortality Survey (UN SA) [Others Direct]
	18	1975	1976 World Fertility Survey [Other DHS Direct]
	18	1992.8	1993-1994 Demographic and Health Survey [DHS Direct]
	15	1995.8	1996-1997 Demographic and Health Survey [DHS Direct]
	3	1999.8	1999-2000 Demographic and Health Survey [DHS Direct]
	18	1998.8	1999-2000 Demographic and Health Survey [DHS Direct]
	18	2003	2004 Demographic and Health Survey [DHS Direct]
	9	2005.2	2007 Demographic and Health Survey [DHS Direct]
	3	2010	2010 Maternal Health Services and Maternal Mortality Survey [Other DHS Direct]
	3	2010.7	2011 Census [Others Direct]
	18	2010.5	2011 Demographic and Health Survey [DHS Direct]
	12	2012.9	2014 Demographic and Health Survey [DHS Direct]
	3	2016	2016 Maternal Health Services and Maternal Mortality Survey [Others Direct]
	12	2016.2	2017-2018 Demographic and Health Survey [DHS Direct]
	18	2018.1	2019 Multiple Indicator Cluster Survey [Others Direct]
	3	2016.5	Report on Sample Vital Registration System 2016 [Others Direct]
	3	2017.5	Report on Sample Vital Registration System 2016 [Others Direct]
	3	2018.5	Report on Sample Vital Registration System 2016 [Others Direct]
	3	2019.5	Report on Sample Vital Registration System 2016 [Others Direct]
	3	2020.5	Report on Sample Vital Registration System 2016 [Others Direct]
	72	2015.5	Sample Vital Statistics [VR]
Barbados	54	2013	
	54	2013	WHO Vital Registration (recalculated - unadjusted) [VR]
Belarus	150	2019.5	
	150	2019.5	WHO/Human Mortality Database Vital Registration (recalculated - unadjusted) [VR]
Belgium	153	2020.5	
	153	2020.5	WHO/Human Mortality Database Vital Registration (recalculated - unadjusted) [VR]
Belize	85	2018.5	
	2	1985.1	1991 Family Health Survey [Others Direct]
	81	2018.5	Recalculated based on WHO - adjusted for incompleteness [VR]
Benin	107	2016.8	

Continued on next page

Table 25 – continued from previous page

Country	# obs.	Most recent obs. year	Data series name [source type]
	17	1981	1982 World Fertility Survey [Other DHS Direct]
	18	1995.4	1996 Demographic and Health Survey [DHS Direct]
	18	2000.6	2001 Demographic and Health Survey [DHS Direct]
	18	2005.6	2006 Demographic and Health Survey [DHS Direct]
	18	2013.5	2014 Multiple Indicator Cluster Survey [Others Direct]
	18	2016.8	2017-2018 Demographic and Health Survey [DHS Direct]
Bhutan	9	2012.5	
	3	1993.1	1994 National Health Survey [Others Direct]
	3	2004.9	2005 Census [Others Direct]
	3	2012.5	2012 National Health Survey [Others Direct]
Bolivia (Plurinational State of)	51	2010.4	
	3	1979.8	1980 National Demographic Survey [Others Direct]
	6	1986.2	1989 Demographic and Health Survey [DHS Direct]
	9	1991.8	1993-1994 Demographic and Health Survey [DHS Direct]
	9	1996.2	1998 Demographic and Health Survey [DHS Direct]
	12	2002.1	2003 Demographic and Health Survey [DHS Direct]
	9	2006.1	2008 Demographic and Health Survey [DHS Direct]
	3	2010.4	2016 Encuesta de Demografma y Salud [Others Direct]
Bosnia and Herzegovina	75	2020.5	
	75	2020.5	WHO Vital Registration (recalculated - unadjusted) [VR]
Botswana	18	2016.5	
	3	1981.1	1981 Census [Others Direct]
	3	1991.1	1991 Census [Others Direct]
	3	2001.1	2001 Census [Others Direct]
	3	2001.8	2008 Family Health Survey [Others Direct]
	3	2011.1	2011 Census [Others Direct]
	3	2016.5	2017 Demographic Survey [Others Direct]
Brazil	104	2020.5	
	2	1980.3	1986 Demographic and Health Survey [DHS Direct]
	3	1990.2	1996 Demographic and Health Survey [DHS Direct]
	3	2010.2	2010 Census [Others Direct]
	96	2020.5	WHO Vital Registration (recalculated - unadjusted) [VR]
British Virgin Islands			
Brunei	87	2019.5	
	87	2019.5	WHO Vital Registration (recalculated - unadjusted) [VR]
Bulgaria	156	2021.5	
	156	2021.5	WHO/Human Mortality Database Vital Registration (recalculated - unadjusted) [VR]
Burkina Faso	70	2009.3	
	3	1960.5	1960-1961 Survey [Others Direct]
	12	1991.4	1993 Demographic and Health Survey [DHS Direct]
	16	1997.8	1998-1999 Demographic and Health Survey [DHS Direct]

Continued on next page

Table 25 – continued from previous page

Country	# obs.	Most recent obs. year	Data series name [source type]
	18	2002.4	2003 Demographic and Health Survey [DHS Direct]
	3	2007.3	2008 Global Fund Evaluation Survey [Others Direct]
	18	2009.3	2010 Demographic and Health Survey [DHS Direct]
Burundi	53	2015.7	
	3	1964.7	1965 Population Survey [Others Direct]
	3	1969.5	1970 Demographic Survey [Others Direct]
	11	1985.7	1987 Demographic and Health Survey [DHS Direct]
	18	2009.6	2010-2011 Demographic and Health Survey [DHS Direct]
	18	2015.7	2016-2017 Demographic and Health Survey [DHS Direct]
Cambodia	57	2012.4	
	18	1999.1	2000 Demographic and Health Survey [DHS Direct]
	18	2004.7	2005 Demographic and Health Survey [DHS Direct]
	12	2009	2010 Demographic and Health Survey [DHS Direct]
	9	2012.4	2014 Demographic and Health Survey [DHS Direct]
Cameroon	99	2016.9	
	3	1975.8	1976 Census [Others Direct]
	12	1976.5	1978 World Fertility Survey [Other DHS Direct]
	3	1986.8	1987 Census [Others Direct]
	9	1989.2	1991 Demographic and Health Survey [DHS Direct]
	12	1996.6	1998 Demographic and Health Survey [DHS Direct]
	18	2003.1	2004 Demographic and Health Survey [DHS Direct]
	18	2010	2011 Demographic and Health Survey [DHS Direct]
	12	2013	2014 Multiple Indicator Cluster Surveys [Others Direct]
	12	2016.9	2018 Demographic and Health Survey [DHS Direct]
Canada	153	2020.5	
	153	2020.5	WHO/Human Mortality Database Vital Registration (recalculated - unadjusted) [VR]
Cape Verde	78	2018.5	
	3	1992.2	1998 Demographic and Reproductive Health Survey [Others Direct]
	3	2000	2000 Census [Others Direct]
	3	2009.5	2010 Census [Others Direct]
	18	1999.5	Recalculated based on WHO/UNPD - adjusted for incompleteness [VR]
	51	2018.5	WHO/UNPD Vital Registration (recalculated - unadjusted) [VR]
Central African Republic	39	2017.4	
	3	1959.5	1959-1960 Survey [Others Direct]
	3	1988.4	1988 Census [Others Direct]
	18	1993.7	1994-1995 Demographic and Health Survey [DHS Direct]
	3	2003.4	2003 Census [Others Direct]
	12	2017.4	2019 Multiple Indicator Cluster Survey [Others Direct]
Chad	75	2018.3	
	18	1995.9	1996-1997 Demographic and Health Survey [DHS Direct]
	18	2003.5	2004 Demographic and Health Survey [DHS Direct]
	3	2008.9	2009 Census [Others Direct]
	18	2013.7	2014-2015 Demographic and Health Survey [DHS Direct]

Continued on next page

Table 25 – continued from previous page

Country	# obs.	Most recent obs. year	Data series name [source type]
	18	2018.3	2019 Multiple Indicator Cluster Survey [Others Direct]
Chile	150	2020.5	
	150	2020.5	WHO/Human Mortality Database/UNPD Vital Registration (recalculated - unadjusted) [VR]
China	33	2010.5	
	3	1973.5	1964-1982 Adjusted Census Deaths (1964-1982) [Others Direct]
	3	1986.5	1982-1990 Adjusted Census Deaths (1982-1990) [Others Direct]
	3	1995.5	1990-2000 Adjusted Census Deaths (1990-2000) [Others Direct]
	3	2000.5	1999-2000 Adjusted Census Deaths (1999-2000) [Others Direct]
	3	2005.5	2000-2010 Adjusted Census Deaths (2000-2010) [Others Direct]
	18	2010.5	China CDC Surveillance surveys [VR]
Colombia	117	2020.5	
	3	1980.7	1986 Demographic and Health Survey [DHS Direct]
	3	1984.3	1990 Demographic and Health Survey [DHS Direct]
	3	1989.2	1995 Demographic and Health Survey [DHS Direct]
	3	1994.1	2000 Demographic and Health Survey [DHS Direct]
	3	2005.4	2005 Census [Others Direct]
	6	2001.7	2005 Demographic and Health Survey [DHS Direct]
	9	2007.8	2010 Demographic and Health Survey [DHS Direct]
	3	2009.1	2015 Demographic and Health Survey [DHS Direct]
	84	2020.5	Recalculated based on WHO - adjusted for incompleteness [VR]
Comoros	15	2017.6	
	3	1958.2	1958 Census [Others Direct]
	3	1990.2	1996 Demographic and Health Survey [DHS Direct]
	3	2003.2	2003 Census [Others Direct]
	3	2006.6	2012 Demographic and Health Survey [DHS Direct]
	3	2017.6	2017 Census [Others Direct]
Congo	23	2011.9	
	8	2003.5	2005 Demographic and Health Survey [DHS Direct]
	9	2009.7	2011-2012 Demographic and Health Survey [DHS Direct]
	6	2011.9	2014-2015 Multiple Indicator Cluster Survey [Others Direct]
Democratic Republic of the Congo	30	2015	
	9	2005.5	2007 Demographic and Health Survey [DHS Direct]
	15	2012.6	2013-2014 Demographic and Health Survey [DHS Direct]
	6	2015	2018 Multiple Indicator Cluster Survey [Others Direct]
Cook Islands	12	2008	
	12	2008	WHO Vital Registration (recalculated - unadjusted) [VR]
Costa Rica	96	2020.5	
	96	2020.5	Recalculated based on WHO - adjusted for incompleteness [VR]
Cote d'Ivoire	71	2014.8	
	3	1977.5	1978-1979 Demographic Survey Repeated Passages [Others Direct]
	17	1979.6	1981 World Fertility Survey [Other DHS Direct]
	18	1993.4	1994 Demographic and Health Survey [DHS Direct]
	6	1995.7	1998-1999 Demographic and Health Survey [DHS Direct]

Continued on next page

Table 25 – continued from previous page

Country	# obs.	Most recent obs. year	Data series name [source type]
	3	1999.6	2005 National Demographic and Health Survey [Other DHS Direct]
	12	2010.4	2011-2012 Demographic and Health Survey [DHS Direct]
	12	2014.8	2016 Multiple Indicator Cluster Survey [Others Direct]
Croatia	117	2021.5	
	3	2010.8	2011 Census [Others Direct]
	114	2021.5	WHO/Human Mortality Database Vital Registration (recalculated - unadjusted) [VR]
Cuba	96	2020.5	
	96	2020.5	WHO Vital Registration (recalculated - unadjusted) [VR]
Cyprus	82	2019.5	
	46	2019.5	Recalculated based on WHO - adjusted for incompleteness [VR]
	36	2000.5	WHO Vital Registration (recalculated - unadjusted) [VR]
Czech Republic	156	2021.5	
	156	2021.5	WHO/Human Mortality Database Vital Registration (recalculated - unadjusted) [VR]
Denmark	156	2021.5	
	156	2021.5	WHO/Human Mortality Database Vital Registration (recalculated - unadjusted) [VR]
Djibouti	6	2006.5	
	3	1996.8	2002 Pan Arab Project for Family Health Family Health Survey [Others Direct]
	3	2006.5	2012 Pan Arab Project for Family Health Family Health Survey [Others Direct]
Dominica	32	2015.5	
	11	2000.5	Recalculated based on WHO - adjusted for incompleteness [VR]
	21	2015.5	WHO Vital Registration (recalculated - unadjusted) [VR]
Dominican Republic	39	2013.8	
	3	1969	1975 World Fertility Survey [Other DHS Direct]
	3	1972.3	1980 World Fertility Survey [Other DHS Direct]
	6	1983.7	1986 Demographic and Health Survey [DHS Direct]
	3	1985.5	1991 Demographic and Health Survey [DHS Direct]
	3	1990.7	1996 Demographic and Health Survey [DHS Direct]
	6	1999.5	2002 Demographic and Health Survey [DHS Direct]
	6	2004.2	2007 Demographic and Health Survey [DHS Direct]
	3	2007.5	2013 Demographic and Health Survey [DHS Direct]
	3	2008.5	2014 Multiple Indicator Cluster Survey [Others Direct]
	3	2013.8	2019 Multiple Indicator Cluster Survey [Others Direct]
Ecuador	117	2020.5	
	3	1981	1987 Demographic and Health Survey [DHS Direct]
	6	1991.4	1994 Demographic and Maternal and Child Health Survey [Others Direct]
	9	1997.2	1999 Demographic and Maternal and Child Health Survey [Others Direct]
	3	1998.6	2004 Demographic and Maternal and Child Health Survey [Others Direct]

Continued on next page

Table 25 – continued from previous page

Country	# obs.	Most recent obs. year	Data series name [source type]
	3	2006.6	2012 Encuesta Nacional de Salud y Nutricion: Salud Sexual y Reproductiva [Others Direct]
	3	2012.9	2018 Encuesta Nacional de Salud y Nutricion [Others Direct]
	90	2020.5	WHO Vital Registration (recalculated - unadjusted) [VR]
Egypt	201	2019.5	
	18	1979.1	1980 World Fertility Survey [Other DHS Direct]
	12	1987.2	1988 Demographic and Health Survey [DHS Direct]
	12	1989.5	1991 Pan Arab Project for Child Development Maternal and Child Health Survey [Others Direct]
	12	1991.3	1992 Demographic and Health Survey [DHS Direct]
	12	1994.3	1995 Demographic and Health Survey [DHS Direct]
	12	1998.6	2000 Demographic and Health Survey [DHS Direct]
	6	2000.3	2003 Interim Demographic and Health Survey [DHS Direct]
	12	2003.7	2005 Demographic and Health Survey [DHS Direct]
	6	2005.2	2008 Demographic and Health Survey [DHS Direct]
	6	2011.2	2014 Demographic and Health Survey [DHS Direct]
	42	2019.5	Recalculated based on WHO - adjusted for incompleteness [VR]
	51	2005.5	WHO Vital Registration (recalculated - unadjusted) [VR]
El Salvador	101	2019.5	
	3	1992.2	1992 Census [Others Direct]
	2	1987.2	1993 National Family Health Survey [Others Direct]
	3	1992.3	1998 National Family Health Survey [Others Direct]
	3	1996.9	2002 National Family Health Survey [Others Direct]
	3	2006.9	2007 Census [Others Direct]
	3	2002.3	2008 National Family Health Survey [Others Direct]
	3	2008.2	2014 Multiple Indicator Cluster Survey [Others Direct]
	81	2019.5	WHO/UNPD Vital Registration (recalculated - unadjusted) [VR]
Equatorial Guinea			
Eritrea	39	2009.1	
	9	1993.7	1995-1996 Demographic and Health Survey [DHS Direct]
	12	2000.7	2002 Demographic and Health Survey [DHS Direct]
	18	2009.1	2010 Population and Health Survey [Other DHS Direct]
Estonia	156	2020.5	
	3	2011.6	2011-2012 Census [Others Direct]
	153	2020.5	WHO/Human Mortality Database Vital Registration (recalculated - unadjusted) [VR]
Ethiopia	72	2016.2	
	18	1999.1	2000 Demographic and Health Survey [DHS Direct]
	18	2004.2	2005 Demographic and Health Survey [DHS Direct]
	18	2009.9	2011 Demographic and Health Survey [DHS Direct]
	12	2014.5	2016 Demographic and Health Survey [DHS Direct]
	6	2016.2	2019 Demographic and Health Survey [DHS Direct]
Federated States of Micronesia			
Fiji	63	2019.5	
	6	1971.1	1974 World Fertility Survey [Other DHS Direct]

Continued on next page

Table 25 – continued from previous page

Country	# obs.	Most recent obs. year	Data series name [source type]
	57	2019.5	Recalculated based on WHO - adjusted for incompleteness [VR]
Finland	156	2021.5	
	156	2021.5	WHO/Human Mortality Database Vital Registration (recalculated - unadjusted) [VR]
France	153	2020.5	
	153	2020.5	WHO/Human Mortality Database Vital Registration (recalculated - unadjusted) [VR]
Gabon	12	2006	
	9	1998.5	2000 Demographic and Health Survey [DHS Direct]
	3	2006	2012 Demographic and Health Survey [DHS Direct]
The Gambia	24	2017.8	
	3	2012.8	2013 Census [Others Direct]
	12	2016.6	2018 Multiple Indicator Cluster Survey [Others Direct]
	9	2017.8	2019-2020 Demographic and Health Survey [DHS Direct]
Georgia	98	2021.5	
	3	1993.9	1999 Reproductive Health Survey [Others Direct]
	3	1999.2	2005 Reproductive Health Survey [Others Direct]
	33	2001.5	Recalculated based on WHO - adjusted for incompleteness [VR]
	57	2021.5	WHO Vital Registration (recalculated - unadjusted) [VR]
Germany	153	2021.5	
	153	2021.5	WHO/Human Mortality Database Vital Registration (recalculated - unadjusted) [VR]
Ghana	94	2016.3	
	11	1986.6	1988 Demographic and Health Survey [DHS Direct]
	12	1992.2	1993-1994 Demographic and Health Survey [DHS Direct]
	9	1996.8	1998-1999 Demographic and Health Survey [DHS Direct]
	6	2000.5	2003 Demographic and Health Survey [DHS Direct]
	6	2005.7	2008 Demographic and Health Survey [DHS Direct]
	3	2010.3	2010 Census [Others Direct]
	9	2009.8	2011 Multiple Indicator Cluster Survey [Others Direct]
	8	2012.7	2014 Demographic and Health Survey [DHS Direct]
	18	2013.7	2017 Demographic and Health Survey [DHS Direct]
	12	2016.3	2017-2018 Multiple Indicator Cluster Survey [Others Direct]
Greece	153	2020.5	
	153	2020.5	WHO/Human Mortality Database Vital Registration (recalculated - unadjusted) [VR]
Grenada	61	2018	
	25	1999.5	Recalculated based on WHO - adjusted for incompleteness [VR]
	36	2018	WHO Vital Registration (recalculated - unadjusted) [VR]
Guatemala	123	2020.5	
	6	1984.7	1987 Demographic and Health Survey [DHS Direct]
	12	1993.9	1995 Demographic and Health Survey [DHS Direct]
	3	1992.8	1998-1999 Interim Demographic and Health Survey [DHS Direct]
	3	1996.3	2002 Encuesta Nacional de Salud Materno Infantil (ENSMI) [Others Direct]

Continued on next page

Table 25 – continued from previous page

Country	# obs.	Most recent obs. year	Data series name [source type]
	3	2002.8	2008 Encuesta Nacional de Salud Materno Infantil (ENSMI) [Others Direct]
	3	2011.7	2014-2015 Demographic and Health Survey [DHS Direct]
	93	2020.5	WHO Vital Registration (recalculated - unadjusted) [VR]
Guinea	81	2017.1	
	3	1954.5	1954-1955 Survey [Others Direct]
	3	1996.4	1996 Census [Others Direct]
	18	1998.3	1999 Demographic and Health Survey [DHS Direct]
	18	2004.1	2005 Demographic and Health Survey [DHS Direct]
	18	2011.4	2012 Demographic and Health Survey [DHS Direct]
	3	2013.8	2014 Census [Others Direct]
	18	2017.1	2018 Demographic and Health Survey [DHS Direct]
Guinea-Bissau	37	2016.1	
	10	2008.8	2010 Multiple Indicator Cluster Survey [Others Direct]
	18	2013.2	2014 Multiple Indicator Cluster Survey [Others Direct]
	9	2016.1	2018-2019 Multiple Indicator Cluster Survey [Others Direct]
Guyana	99	2014.5	
	6	1972.4	1975 World Fertility Survey [Other DHS Direct]
	3	1999.4	2005 AIDS Indicator Survey [Other DHS Direct]
	3	2003.2	2009 Demographic and Health Survey [DHS Direct]
	3	2012.2	2012 Census [Others Direct]
	3	2008.3	2014 Multiple Indicator Cluster Survey [Others Direct]
	3	2013.5	2019-2020 Multiple Indicator Cluster Survey [Others Direct]
	78	2014.5	WHO Vital Registration (recalculated - unadjusted) [VR]
Haiti	44	2014.8	
	8	1975.5	1977 World Fertility Survey [Other DHS Direct]
	9	1992.5	1994-1995 Demographic and Health Survey [DHS Direct]
	6	1997.1	2000 Demographic and Health Survey [DHS Direct]
	9	2001.2	2005-2006 Demographic and Health Survey [DHS Direct]
	6	2007.5	2012 Demographic and Health Survey [DHS Direct]
	6	2014.8	2016-2017 Demographic and Health Survey [DHS Direct]
Honduras	38	2016.5	
	3	1971.3	1970-1972 National Demographic Survey [Others Direct]
	3	1982.5	1983 National Demographic Survey [Others Direct]
	3	1990.1	1996 Reproductive Health Survey [Others Direct]
	3	2001.1	2001 Census [Others Direct]
	3	1992.2	2001 Reproductive Health Survey [Others Direct]
	8	2003.7	2005-2006 Demographic and Health Survey [DHS Direct]
	6	2008.7	2011-2012 Demographic and Health Survey [DHS Direct]
	3	2013.1	2013 Census [Others Direct]
	6	2016.5	2019 Multiple Indicator Cluster Survey [Others Direct]
Hungary	153	2020.5	
	153	2020.5	WHO/Human Mortality Database Vital Registration (recalculated - unadjusted) [VR]
Iceland	91	2018.5	

Continued on next page

Table 25 – continued from previous page

Country	# obs.	Most recent obs. year	Data series name [source type]
	91	2018.5	WHO/Human Mortality Database Vital Registration (recalculated - unadjusted) [VR]
India	177	2020.5	
	3	1970.5	1971 Census [Others Direct]
	3	1980.5	1981 Census [Others Direct]
	18	1991.2	1992-1993 Demographic and Health Survey [DHS Direct]
	3	1991.9	1992-1993 National Family Health Survey [DHS Direct]
	18	1997.8	1998-1999 Demographic and Health Survey [DHS Direct]
	3	1998.2	1998-1999 National Family Health Survey [DHS Direct]
	18	2004.8	2005-2006 Demographic and Health Survey [DHS Direct]
	111	2020.5	Sample Registration System [VR]
Indonesia	147	2016.6	
	3	1964.5	1964-1965 National Socio-economic Survey [Others Direct]
	18	1975.2	1976 World Fertility Survey [Other DHS Direct]
	18	1986.7	1987 Demographic and Health Survey [DHS Direct]
	18	1990.3	1991 Demographic and Health Survey [DHS Direct]
	18	1993.5	1994 Demographic and Health Survey [DHS Direct]
	18	1996.7	1997 Demographic and Health Survey [DHS Direct]
	12	2001.2	2002-2003 Demographic and Health Survey [DHS Direct]
	12	2005.9	2007 Demographic and Health Survey [DHS Direct]
	3	2009.9	2010 Census [Others Direct]
	9	2010.8	2012 Demographic and Health Survey [DHS Direct]
	18	2016.6	2017 Demographic and Health Survey [DHS Direct]
Iran (Islamic Republic of)	42	2017.5	
	3	1975	1973-1976 Population Growth Survey [Others Direct]
	3	1985.5	1986 Census [Others Direct]
	3	1999.5	2000 Demographic and Health Survey [Other DHS Direct]
	33	2017.5	Recalculated based on WHO - adjusted for incompleteness [VR]
Iraq	24	2012.2	
	9	2004.2	2006 Multiple Indicator Cluster Survey [Others Direct]
	9	2009.2	2011 Multiple Indicator Cluster Survey [Others Direct]
	3	2012.2	2018 Multiple Indicator Cluster Survey [Others Direct]
	3	1974	Demographic Sample Survey and Sample Registration System [VR]
Ireland	153	2020.5	
	153	2020.5	WHO/Human Mortality Database Vital Registration (recalculated - unadjusted) [VR]
Israel	141	2021.5	
	141	2021.5	WHO/Human Mortality Database Vital Registration (recalculated - unadjusted) [VR]
Italy	153	2020.5	
	153	2020.5	WHO/Human Mortality Database Vital Registration (recalculated - unadjusted) [VR]
Jamaica	3	2010.2	
	3	2010.2	2011 Census [Others Direct]
Japan	150	2021.5	

Continued on next page

Table 25 – continued from previous page

Country	# obs.	Most recent obs. year	Data series name [source type]
	150	2021.5	WHO/Human Mortality Database Vital Registration (recalculated - unadjusted) [VR]
Jordan	20	2011.7	
	3	1984.7	1990 Demographic and Health Survey [DHS Direct]
	3	1991.4	1997 Demographic and Health Survey [DHS Direct]
	3	1996.5	2002 Demographic and Health Survey [DHS Direct]
	3	2001.4	2007 Demographic and Health Survey [DHS Direct]
	2	2003.7	2009 Demographic and Health Survey [DHS Direct]
	3	2006.7	2012 Demographic and Health Survey [DHS Direct]
	3	2011.7	2017 Demographic and Health Survey [DHS Direct]
Kazakhstan	105	2021.5	
	3	1989.3	1995 Demographic and Health Survey [DHS Direct]
	3	1993.5	1999 Demographic and Health Survey [DHS Direct]
	99	2021.5	WHO Vital Registration (recalculated - unadjusted) [VR]
Kenya	73	2013.3	
	11	1976.1	1978 World Fertility Survey [Other DHS Direct]
	6	1985.9	1989 Demographic and Health Survey [DHS Direct]
	9	1991.1	1993 Demographic and Health Survey [DHS Direct]
	11	1996.6	1998 Demographic and Health Survey [DHS Direct]
	12	2001.7	2003 Demographic and Health Survey [DHS Direct]
	6	2005.8	2008-2009 Demographic and Health Survey [DHS Direct]
	18	2013.3	2014 Demographic and Health Survey [DHS Direct]
Kiribati	3	2012.9	
	3	2012.9	2019 Multiple Indicator Cluster Survey [Others Direct]
Democratic People's Republic of Korea	6	2008.3	
	3	1993.5	1993 Census [Others Direct]
	3	2008.3	2008 Census [Others Direct]
Republic of Korea	105	2021.5	
	6	1971.7	1974 World Fertility Survey [Other DHS Direct]
	99	2021.5	WHO/Human Mortality Database Vital Registration (recalculated - unadjusted) [VR]
Kosovo	4	2014	
	2	2014	2019-2020 Multiple Indicator Cluster Survey [Others Direct]
Kuwait	87	2019.5	
	87	2019.5	WHO Vital Registration (recalculated - unadjusted) [VR]
Kyrgyzstan	110	2021.5	
	3	1991.6	1997 Demographic and Health Survey [DHS Direct]
	2	2006.6	2012 Demographic and Health Survey [DHS Direct]
	3	2008.3	2014 Multiple Indicator Cluster Survey [Others Direct]
	3	2012.8	2018 Multiple Indicator Cluster Survey [Others Direct]
	99	2021.5	WHO Vital Registration (recalculated - unadjusted) [VR]
Lao People's Democratic Republic	51	2016.1	

Continued on next page

Table 25 – continued from previous page

Country	# obs.	Most recent obs. year	Data series name [source type]
	3	2004.8	2005 Census [Others Direct]
	18	2004.8	2005 Reproductive Health Survey [Others Direct]
	18	2010.8	2012 Lao Social Indicator Survey (combined Multiple Indicator Cluster Survey4/Demographic and Health Survey) [DHS Direct]
	12	2016.1	2017 Multiple Indicator Cluster Survey [Others Direct]
Latvia	153	2021.5	
	153	2021.5	WHO/Human Mortality Database Vital Registration (recalculated - unadjusted) [VR]
Lebanon	3	1990	
	3	1990	1996 Pan Arab Project for Child Development Maternal and Child Health Survey [Others Direct]
Lesotho	27	2015.5	
	3	1971.9	1971-1973 Demographic Survey [Others Direct]
	6	1974.6	1977 World Fertility Survey [Other DHS Direct]
	3	2000.9	2001 Demographic Survey [Others Direct]
	3	1998.7	2004 Demographic and Health Survey [DHS Direct]
	3	2003.7	2009 Demographic and Health Survey [DHS Direct]
	3	2008.7	2014 Demographic and Health Survey [DHS Direct]
	3	2015.5	2016 Census [Others Direct]
	3	2012.3	2018 Multiple Indicator Cluster Survey [Others Direct]
Liberia	51	2017.7	
	3	1969.5	1969-1970 Population Growth Survey [Others Direct]
	6	1983.1	1986 Demographic and Health Survey [DHS Direct]
	9	2004.9	2006-2007 Demographic and Health Survey [DHS Direct]
	12	2007.4	2008-2009 Malaria Indicator Survey [Other DHS Direct]
	12	2011.7	2013 Demographic and Health Survey [DHS Direct]
	9	2017.7	2019-2020 Demographic and Health Survey [DHS Direct]
Libya	12	2004.4	
	6	1992	1995 Pan Arab Project for Child Development Maternal and Child Health Survey [Others Direct]
	6	2004.4	2007 Pan Arab Project for Family Health Family Health Survey [Others Direct]
Lithuania	162	2021.5	
	3	2010.8	2011 Census [Others Direct]
	3	2017.5	2018 Statistical Survey [Others Direct]
	156	2021.5	WHO/Human Mortality Database Vital Registration (recalculated - unadjusted) [VR]
Luxembourg	90	2020.5	
	90	2020.5	WHO/Human Mortality Database Vital Registration (recalculated - unadjusted) [VR]
Macedonia	93	2020.5	
	93	2020.5	Recalculated based on WHO - adjusted for incompleteness [VR]
Madagascar	87	2020.2	
	3	1966.1	1966 Demographic Survey [Others Direct]
	3	1993.1	1993 Census [Others Direct]
	18	1996.7	1997 Demographic and Health Survey [DHS Direct]

Continued on next page

Table 25 – continued from previous page

Country	# obs.	Most recent obs. year	Data series name [source type]
	9	2001.8	2003-2004 Demographic and Health Survey [DHS Direct]
	18	2007.8	2008-2009 Demographic and Health Survey [DHS Direct]
	18	2017.7	2018 Multiple Indicator Cluster Survey [Others Direct]
	18	2020.2	2021 Demographic and Health Survey [DHS Direct]
Malawi	152	2019	
	2	1971.1	1970-1972 Population Change Survey [Others Direct]
	3	1977.2	1977 Census [Others Direct]
	3	1983.9	1984 Family Formation Survey [Others Direct]
	3	1987.2	1987 Census [Others Direct]
	12	1991.2	1992 Demographic and Health Survey [DHS Direct]
	18	1999.5	2000 Demographic and Health Survey [DHS Direct]
	18	2003.7	2004 Demographic and Health Survey [DHS Direct]
	18	2005.6	2006 MDG Endline Survey [Others Direct]
	18	2009.4	2010 Demographic and Health Survey [DHS Direct]
	18	2012.9	2013-2014 MDG Endline Survey [Others Direct]
	18	2014.7	2015-2016 Demographic and Health Survey [DHS Direct]
	3	2018.2	2018 Census [Others Direct]
	18	2019	2019-2020 MDG Endline Survey [Others Direct]
Malaysia	99	2021.5	
	9	1972.6	1974 World Fertility Survey [Other DHS Direct]
	90	2021.5	WHO Vital Registration (recalculated - unadjusted) [VR]
Maldives	93	2020.5	
	3	2003	2009 Demographic and Health Survey [DHS Direct]
	3	2010.2	2016-2017 Demographic and Health Survey [DHS Direct]
	18	1994.5	Recalculated based on WHO - adjusted for incompleteness [VR]
	69	2020.5	WHO Vital Registration (recalculated - unadjusted) [VR]
Mali	107	2017.6	
	3	1976.4	1976 Census [Others Direct]
	12	1985.7	1987 Demographic and Health Survey [DHS Direct]
	18	1994.8	1995-1996 Demographic and Health Survey [DHS Direct]
	3	1997.8	1998 Census [Others Direct]
	18	2000	2001 Demographic and Health Survey [DHS Direct]
	18	2005.3	2006 Demographic and Health Survey [DHS Direct]
	18	2014.6	2015 Multiple Indicator Cluster Survey [Others Direct]
	17	2017.6	2018 Demographic and Health Survey [DHS Direct]
Malta	51	2021.5	
	51	2021.5	WHO Vital Registration (recalculated - unadjusted) [VR]
Marshall Islands			
Mauritania	63	2017.9	
	3	1977	1976-1977 Census [Others Direct]
	12	1979.8	1981 World Fertility Survey [Other DHS Direct]
	3	1987.8	1988 Census [Others Direct]
	12	1988.5	1990 Pan Arab Project for Child Development Maternal and Child Health Survey [Others Direct]
	6	1997.6	2000-2001 Demographic and Health Survey [DHS Direct]
	9	2009.5	2011 Multiple Indicator Cluster Survey [Others Direct]

Continued on next page

Table 25 – continued from previous page

Country	# obs.	Most recent obs. year	Data series name [source type]
	9	2013.6	2015 Multiple Indicator Cluster Survey [Others Direct]
	9	2017.9	2021 Demographic and Health Survey [DHS Direct]
Mauritius	96	2020.5	
	96	2020.5	WHO Vital Registration (recalculated - unadjusted) [VR]
Mexico	132	2020.5	
	3	1981.1	1987 Demographic and Health Survey [DHS Direct]
	12	2007.6	2009 Encuesta Nacional de la Dinamica Demografica (ENADID) [Others Direct]
	9	2012.7	2014 Encuesta Nacional de la Dinamica Demografica (ENADID) [Others Direct]
	12	2017.2	2018 Encuesta Nacional de la Dinamica Demografica (ENADID) [Others Direct]
	96	2020.5	WHO Vital Registration (recalculated - unadjusted) [VR]
Republic of Moldova	99	2020.5	
	3	1999.4	2005 Demographic and Health Survey [DHS Direct]
	3	2006.3	2012 Multiple Indicator Cluster Survey [Others Direct]
	93	2020.5	WHO Vital Registration (recalculated - unadjusted) [VR]
Monaco			
Mongolia	99	2019.5	
	3	1992.8	1998 Reproductive Health Survey [Others Direct]
	3	2009.5	2010 Census [Others Direct]
	3	2007.8	2013-2014 Social Indicator Sample Survey (SISS) [Others Direct]
	3	2012.8	2018 Social Indicator Sample Survey (SISS) [Others Direct]
	27	1999.5	Recalculated based on WHO - adjusted for incompleteness [VR]
	60	2019.5	WHO Vital Registration (recalculated - unadjusted) [VR]
Montenegro	78	2021	
	36	2002.5	Recalculated based on WHO - adjusted for incompleteness [VR]
	42	2021	WHO Vital Registration (recalculated - unadjusted) [VR]
Montserrat	3	2004.5	
	3	2004.5	WHO Vital Registration (recalculated - unadjusted) [VR]
Morocco	57	2010	
	9	1978.3	1980 World Fertility Survey [Other DHS Direct]
	12	1985.8	1987 Demographic and Health Survey [DHS Direct]
	6	1989	1992 Demographic and Health Survey [DHS Direct]
	3	1989	1995 Demographic and Health Survey [DHS Direct]
	6	1994	1997 Pan Arab Project for Child Development Maternal and Child Health Survey [Others Direct]
	9	2001.7	2003-2004 Demographic and Health Survey [DHS Direct]
	9	2001	2004 Pan Arab Project for Family Health Family Health Survey [Others Direct]
	3	2010	2009-2010 National Demographic Survey with repeated passages [Others Direct]
Mozambique	48	2009.9	
	18	2002.6	2003-2004 Demographic and Health Survey [DHS Direct]
	18	2007.7	2008 Multiple Indicator Cluster Survey [Others Direct]

Continued on next page

Table 25 – continued from previous page

Country	# obs.	Most recent obs. year	Data series name [source type]
	12	2009.9	2011 Demographic and Health Survey [DHS Direct]
Myanmar	9	2013.8	
	3	2013.8	2014 Census [Others Direct]
	6	2012.9	2015-2016 Demographic and Health Survey [DHS Direct]
Namibia	33	2015.5	
	9	1990.5	1992 Demographic and Health Survey [DHS Direct]
	3	1994.7	2000 Demographic and Health Survey [DHS Direct]
	3	2001	2001 Census [Others Direct]
	6	2003.8	2006-2007 Demographic and Health Survey [DHS Direct]
	3	2011.2	2011 Census [Others Direct]
	6	2010.3	2013 Demographic and Health Survey [DHS Direct]
	3	2015.5	2016 Inter-censal Demographic Survey [Others Direct]
Nauru			
Nepal	91	2013.4	
	18	1990.6	1991 Fertility and Family Planning Survey [Others Direct]
	18	1995	1996 Demographic and Health Survey [DHS Direct]
	3	2001	2001 Census [Others Direct]
	17	2000	2001 Demographic and Health Survey [DHS Direct]
	11	2004.6	2006 Demographic and Health Survey [DHS Direct]
	3	2010.5	2011 Census [Others Direct]
	6	2008	2011 Demographic and Health Survey [DHS Direct]
	6	2011.2	2014 Multiple Indicator Cluster Survey [Others Direct]
	6	2013.4	2016 Demographic and Health Survey [DHS Direct]
	3	2013.3	2019 Multiple Indicator Cluster Survey [Others Direct]
Netherlands	156	2021.5	
	156	2021.5	WHO/Human Mortality Database Vital Registration (recalculated - unadjusted) [VR]
New Zealand	156	2021.5	
	3	2005.7	2006 Census [Others Direct]
	153	2021.5	WHO/Human Mortality Database Vital Registration (recalculated - unadjusted) [VR]
Nicaragua	24	2005.6	
	9	1995.9	1998 Demographic and Health Survey [DHS Direct]
	9	1999.7	2001 Demographic and Health Survey [DHS Direct]
	3	2000.8	2006 Encuesta NicaragÃense de Demografâ a y Salud [Others Direct]
	3	2005.6	2012 Encuesta Nicaraguense de Demografia y Salud [Others Direct]
Niger	93	2020.3	
	18	1991.2	1992 Demographic and Health Survey [DHS Direct]
	18	1997.2	1998 Demographic and Health Survey [DHS Direct]
	3	2000.9	2001 Census [Others Direct]
	18	2005	2006 Demographic and Health Survey [DHS Direct]
	18	2011.1	2012 Demographic and Health Survey [DHS Direct]
	18	2020.3	2021 Enquete Nationale sur la Fecondite et la Mortalite des Enfants de moins de 5 ans (ENAFEME) [Others Direct]
Nigeria	100	2017.6	
	3	1965.6	1965-1966 Rural Demographic Sample Survey [Others Direct]

Continued on next page

Table 25 – continued from previous page

Country	# obs.	Most recent obs. year	Data series name [source type]
	16	1989.2	1990 Demographic and Health Survey [DHS Direct]
	12	2001.7	2003 Demographic and Health Survey [DHS Direct]
	3	2008.1	2008 Demographic and Health Survey [DHS Direct]
	18	2007.4	2008 Demographic and Health Survey [DHS Direct]
	9	2008.7	2010 Malaria Indicator Survey [Other DHS Direct]
	3	2012.8	2013 Demographic and Health Survey [DHS Direct]
	18	2012.1	2013 Demographic and Health Survey [DHS Direct]
	18	2017.6	2018 Demographic and Health Survey [DHS Direct]
Niue	3	1995	
	3	1995	WHO Vital Registration (recalculated - unadjusted) [VR]
Norway	156	2021.5	
	156	2021.5	WHO/Human Mortality Database Vital Registration (recalculated - unadjusted) [VR]
Oman			
Pakistan	90	2014.8	
	3	1964	1962-1965 Population Growth Estimation Experiment [Others Direct]
	3	1970	1971 Population Growth Survey I [Others Direct]
	6	1987.9	1990-1991 Demographic and Health Survey [DHS Direct]
	9	2002.2	2006-2007 Demographic and Health Survey [DHS Direct]
	9	2010.7	2012-2013 Demographic and Health Survey [DHS Direct]
	6	2014.8	2017-2018 Demographic and Health Survey [DHS Direct]
	54	2007.5	Pakistan Demographic Survey [VR]
Palau	24	2004	
	24	2004	WHO/UNPD Vital Registration (recalculated - unadjusted) [VR]
Panama	96	2021.5	
	96	2021.5	Recalculated based on WHO - adjusted for incompleteness [VR]
Papua New Guinea	6	2013.7	
	6	2013.7	2016-2018 Demographic and Health Survey [DHS Direct]
Paraguay	23	2010.5	
	6	1976.1	1979 World Fertility Survey [Other DHS Direct]
	3	1984.3	1990 Demographic and Health Survey [DHS Direct]
	3	1989.8	1996 Encuesta Nacional de Demografia y Salud Sexual y Reproductiva [Others Direct]
	3	2002.2	2002 Census [Others Direct]
	2	1998.2	2004 Encuesta Nacional de Demografia y Salud Sexual y Reproductiva [Others Direct]
	3	2002.5	2008 Encuesta Nacional de Demografia y Salud Sexual y Reproductiva [Others Direct]
	3	2010.5	2016 Multiple Indicator Cluster Survey [Others Direct]
Peru	111	2015.1	
	6	1983.7	1986 Demographic and Health Survey [DHS Direct]
	12	1990.2	1991-1992 Demographic and Health Survey [DHS Direct]
	18	1995.6	1996 Demographic and Health Survey [DHS Direct]
	18	1999.5	2000 Demographic and Health Survey [DHS Direct]
	12	2005.5	2004-2008 Demographic and Health Survey [DHS Direct]
	9	2007.2	2009 Demographic and Health Survey [DHS Direct]

Continued on next page

Table 25 – continued from previous page

Country	# obs.	Most recent obs. year	Data series name [source type]
	6	2007.2	2010 Demographic and Health Survey [DHS Direct]
	6	2008.2	2011 Demographic and Health Survey [DHS Direct]
	6	2009.2	2012 Demographic and Health Survey [DHS Direct]
	3	2007.2	2013 Demographic and Health Survey [DHS Direct]
	6	2012.2	2014 Demographic and Health Survey [DHS Direct]
	3	2011.2	2017 National Demographic and Health Survey [Other DHS Direct]
	3	2012.2	2018 National Demographic and Health Survey [Other DHS Direct]
	3	2015.1	2021 National Demographic and Health Survey [Other DHS Direct]
Philippines	159	2019.5	
	17	1977.1	1978 World Fertility Survey [Other DHS Direct]
	17	1992.2	1993 Demographic and Health Survey [DHS Direct]
	14	1997.1	1998 Demographic and Health Survey [DHS Direct]
	12	2001.9	2003 Demographic and Health Survey [DHS Direct]
	6	2005.6	2008 Demographic and Health Survey [DHS Direct]
	9	2011.6	2013 Demographic and Health Survey [DHS Direct]
	6	2014.6	2017 Demographic and Health Survey [DHS Direct]
	57	2019.5	Recalculated based on WHO/UNPD - adjusted for incompleteness [VR]
	21	1999.5	WHO Vital Registration (recalculated - unadjusted) [VR]
Poland	156	2021.5	
	156	2021.5	WHO/Human Mortality Database Vital Registration (recalculated - unadjusted) [VR]
Portugal	159	2021.5	
	3	2010.8	2011 Census [Others Direct]
	156	2021.5	WHO/Human Mortality Database Vital Registration (recalculated - unadjusted) [VR]
Qatar	89	2020.5	
	89	2020.5	WHO Vital Registration (recalculated - unadjusted) [VR]
Romania	99	2021.5	
	99	2021.5	WHO Vital Registration (recalculated - unadjusted) [VR]
Russian Federation	150	2020.5	
	150	2020.5	WHO/Human Mortality Database Vital Registration (recalculated - unadjusted) [VR]
Rwanda	134	2018.3	
	18	1982.6	1983 World Fertility Survey [Other DHS Direct]
	17	1991.4	1992 Demographic and Health Survey [DHS Direct]
	15	1999.4	2000 Demographic and Health Survey [DHS Direct]
	15	2004.1	2005 Demographic and Health Survey [DHS Direct]
	18	2006.9	2008 Demographic and Health Survey [DHS Direct]
	18	2009.7	2010 Demographic and Health Survey [DHS Direct]
	3	2012.1	2012 Census [Others Direct]
	18	2013.8	2014-2015 Demographic and Health Survey [DHS Direct]
	12	2018.3	2019-2020 Demographic and Health Survey [DHS Direct]
Saint Kitts and Nevis	23	2015.5	
	23	2015.5	WHO Vital Registration (recalculated - unadjusted) [VR]
Saint Lucia	74	2018.5	

Continued on next page

Table 25 – continued from previous page

Country	# obs.	Most recent obs. year	Data series name [source type]
	74	2018.5	WHO Vital Registration (recalculated - unadjusted) [VR]
Samoa	15	2016.4	
	3	1999	1999 Demographic and Health Survey [Other DHS Direct]
	2	1999.5	2000 Demographic and Vital Statistics Survey [Others Direct]
	3	2006.4	2006 Census [Others Direct]
	3	2011.4	2011 Population and Housing Census [Others Direct]
	2	2013.8	2019-2020 Multiple Indicator Cluster Survey [Others Direct]
San Marino	3	1991	
	3	1991	WHO Vital Registration (recalculated - unadjusted) [VR]
Sao Tome and Principe	11	2013.6	
	3	2002.7	2008-2009 Demographic and Health Survey [DHS Direct]
	3	2011.9	2012 Census [Others Direct]
	2	2008.3	2014 Multiple Indicator Cluster Survey [Others Direct]
	3	2013.6	2019 Multiple Indicator Cluster Survey [Others Direct]
Saudi Arabia			
Senegal	218	2017.3	
	18	1977.4	1978 World Fertility Survey [Other DHS Direct]
	18	1985.2	1986 Demographic and Health Survey [DHS Direct]
	18	1991.8	1992-1993 Demographic and Health Survey [DHS Direct]
	17	1996	1997 Demographic and Health Survey [DHS Direct]
	18	1998.8	1999-2000 Demographic and Health Survey [DHS Direct]
	3	2002.5	2002 Census [Others Direct]
	18	2004.1	2005 Demographic and Health Survey [DHS Direct]
	18	2007.9	2008-2009 Malaria Indicator Survey [Other DHS Direct]
	18	2009.7	2010-2011 Demographic and Health Survey [DHS Direct]
	12	2011.2	2012-2013 Demographic and Health Survey [DHS Direct]
	3	2013.4	2013 Census [Others Direct]
	6	2011	2014 Demographic and Health Survey [DHS Direct]
	6	2012	2015 Demographic and Health Survey [DHS Direct]
	9	2014	2016 Demographic and Health Survey [DHS Direct]
	18	2016.2	2017 Demographic and Health Survey [DHS Direct]
	9	2016.4	2018 Demographic and Health Survey [DHS Direct]
	9	2017.3	2019 Demographic and Health Survey [DHS Direct]
Serbia	99	2021.5	
	60	2021.5	Recalculated based on WHO - adjusted for incompleteness [VR]
	39	2001.5	WHO Vital Registration (recalculated - unadjusted) [VR]
Seychelles	53	2016.5	
	3	1959.8	1960 Census [Others Direct]
	50	2016.5	WHO Vital Registration (recalculated - unadjusted) [VR]
Sierra Leone	66	2018.3	
	12	2006.7	2008 Demographic and Health Survey [DHS Direct]
	18	2012.4	2013 Demographic and Health Survey [DHS Direct]
	18	2016.4	2017 Multiple Indicator Cluster Survey [Others Direct]
	18	2018.3	2019 Demographic and Health Survey [DHS Direct]
Singapore	96	2020.5	

Continued on next page

Table 25 – continued from previous page

Country	# obs.	Most recent obs. year	Data series name [source type]
	96	2020.5	WHO Vital Registration (recalculated - unadjusted) [VR]
Slovakia	156	2021.5	
	156	2021.5	WHO/Human Mortality Database Vital Registration (recalculated - unadjusted) [VR]
Slovenia	117	2021.5	
	117	2021.5	WHO/Human Mortality Database Vital Registration (recalculated - unadjusted) [VR]
Solomon Islands	6	2009.4	
	3	2009.4	2009 Census [Others Direct]
	3	2008	2015 Demographic and Health Survey [Other DHS Direct]
Somalia	12	2005.2	
	12	2005.2	2006 Multiple Indicator Cluster Survey [Others Direct]
South Africa	75	2020.5	
	6	1995	1998 Demographic and Health Survey [DHS Direct]
	3	2011.3	2011 Census [Others Direct]
	3	2010.4	2016 Demographic and Health Survey [DHS Direct]
	63	2020.5	Rapid Mortality Surveillance [VR]
South Sudan	6	2007.2	
	6	2007.2	2010 Multiple Indicator Cluster Survey [Others Direct]
Spain	153	2020.5	
	153	2020.5	WHO/Human Mortality Database Vital Registration (recalculated - unadjusted) [VR]
Sri Lanka	93	2014.5	
	6	1984	1987 Demographic and Health Survey [DHS Direct]
	6	1990.6	1993 Demographic and Health Survey [Other DHS Direct]
	3	1994.4	2000 Demographic and Health Survey [Other DHS Direct]
	6	2003.7	2007 Demographic and Health Survey [Other DHS Direct]
	3	2010.4	2016 Demographic and Health Survey [Other DHS Direct]
	69	2014.5	WHO Vital Registration (recalculated - unadjusted) [VR]
Saint Vincent and the Grenadines	64	2017	
	64	2017	WHO Vital Registration (recalculated - unadjusted) [VR]
State of Palestine	27	2013.9	
	9	1993.9	1995 Demographic Survey [Others Direct]
	6	2003	2006 Pan Arab Project for Family Health Family Health Survey [Others Direct]
	6	2007.4	2010 Multiple Indicator Cluster Survey [Others Direct]
	3	2008.2	2014 Multiple Indicator Cluster Survey [Others Direct]
	3	2013.9	2019-2020 Multiple Indicator Cluster Survey [Others Direct]
Sudan	63	2013.2	
	9	1976	1979 World Fertility Survey [Other DHS Direct]
	18	1988.8	1989-1990 Demographic and Health Survey [DHS Direct]
	18	1991	1993 Pan Arab Project for Child Development Maternal and Child Health Survey [Others Direct]
	6	2007.2	2010 Multiple Indicator Cluster Survey [Others Direct]

Continued on next page

Table 25 – continued from previous page

Country	# obs.	Most recent obs. year	Data series name [source type]
	12	2013.2	2014 Multiple Indicator Cluster Survey [Others Direct]
Suriname	96	2019.5	
	3	2012.2	2018 Multiple Indicator Cluster Survey [Others Direct]
	93	2019.5	Recalculated based on WHO/UNPD - adjusted for incompleteness [VR]
Eswatini	21	2013.1	
	3	1996.9	1997 Census [Others Direct]
	3	2000.5	2006-2007 Demographic and Health Survey [DHS Direct]
	6	2007.7	2010 Multiple Indicator Cluster Survey [Others Direct]
	9	2013.1	2014 Multiple Indicator Cluster Survey [Others Direct]
Sweden	156	2021.5	
	156	2021.5	WHO/Human Mortality Database Vital Registration (recalculated - unadjusted) [VR]
Switzerland	156	2021.5	
	156	2021.5	WHO/Human Mortality Database Vital Registration (recalculated - unadjusted) [VR]
Syria	51	2010.5	
	6	1975.5	1978 World Fertility Survey [Other DHS Direct]
	3	1987	1993 Pan Arab Project for Child Development Maternal and Child Health Survey [Others Direct]
	3	1995.8	2001 Pan Arab Project for Family Health Family Health Survey [Others Direct]
	39	2010.5	WHO Vital Registration (recalculated - unadjusted) [VR]
Tajikistan	75	2021.5	
	3	2006.5	2012 Demographic and Health Survey [DHS Direct]
	3	2011.6	2017 Demographic and Health Survey [DHS Direct]
	27	1999.5	Recalculated based on WHO/UNPD - adjusted for incompleteness [VR]
	42	2021.5	WHO Vital Registration (recalculated - unadjusted) [VR]
Tanzania	101	2014.1	
	3	1973.3	1973 National Demographic Survey [Others Direct]
	18	1990.7	1991-1992 Demographic and Health Survey [DHS Direct]
	17	1995.5	1996 Demographic and Health Survey [DHS Direct]
	3	1993.7	1999 Demographic and Health Survey [DHS Direct]
	18	2003.7	2004-2005 Demographic and Health Survey [DHS Direct]
	12	2006.2	2007-2008 AIDS Indicator Survey [Other DHS Direct]
	12	2008.4	2010 Demographic and Health Survey [DHS Direct]
	3	2010.4	2010-2011 National Panel Survey [Others Direct]
	3	2012.2	2012-2013 National Panel Survey [Others Direct]
	12	2014.1	2015-2016 Demographic and Health Survey [DHS Direct]
Thailand	98	2019.5	
	3	1981.2	1987 Demographic and Health Survey [DHS Direct]
	3	1989	1989 Survey of Population Change [Others Direct]
	3	1995.5	1995 Survey of Population Change [Others Direct]
	2	2005.5	2005-2006 Survey of Population Change [Others Direct]
	87	2019.5	Recalculated based on WHO - adjusted for incompleteness [VR]
Timor Leste	15	2008.4	
	12	2008.1	2009-2010 Demographic and Health Survey [DHS Direct]

Continued on next page

Table 25 – continued from previous page

Country	# obs.	Most recent obs. year	Data series name [source type]
	3	2008.4	2015 Census [Others Direct]
Togo	66	2016.3	
	3	1961.4	1961 Demographic survey [Others Direct]
	12	1986.9	1988 Demographic and Health Survey [DHS Direct]
	18	1997.1	1998 Demographic and Health Survey [DHS Direct]
	3	2010.4	2010 Census [Others Direct]
	18	2012.8	2013-2014 Demographic and Health Survey [DHS Direct]
	12	2016.3	2017 Multiple Indicator Cluster Survey [Others Direct]
Tonga	75	2018.5	
	3	2006.4	2006 Census [Others Direct]
	2	2013.8	2019 Multiple Indicator Cluster Survey [Others Direct]
	41	2005.5	Recalculated based on WHO - adjusted for incompleteness [VR]
	29	2018.5	WHO Vital Registration (recalculated - unadjusted) [VR]
Trinidad and Tobago	92	2017.5	
	3	1971.2	1977 World Fertility Survey [Other DHS Direct]
	2	1981.3	1987 Demographic and Health Survey [DHS Direct]
	87	2017.5	WHO/UNPD Vital Registration (recalculated - unadjusted) [VR]
Tunisia	51	2017.5	
	3	1969	1968-1969 National Demographic Survey [Others Direct]
	6	1985.4	1988 Demographic and Health Survey [DHS Direct]
	3	1988	1994 Pan Arab Project for Child Development Maternal and Child Health Survey [Others Direct]
	3	1995	2001 Pan Arab Project for Family Health Family Health Survey [Others Direct]
	3	2006	2011-2012 Multiple Indicator Cluster Survey [Others Direct]
	3	2012.2	2018 Multiple Indicator Cluster Survey [Others Direct]
	21	2000.5	Recalculated based on WHO - adjusted for incompleteness [VR]
	9	2017.5	WHO Vital Registration (recalculated - unadjusted) [VR]
Turkey	59	2019.5	
	8	1976.7	1978 World Fertility Survey [Other DHS Direct]
	3	1987.6	1993 Demographic and Health Survey [DHS Direct]
	3	1992.6	1998 Demographic and Health Survey [DHS Direct]
	3	1997.9	2003-2004 Demographic and Health Survey [Other DHS Direct]
	3	2002.7	2003 National Verbal Autopsy Survey [Others Direct]
	3	2002.7	2008 Turkey Demographic and Health Survey [Other DHS Direct]
	3	2007.7	2013 NDemographic and Health Survey [Other DHS Direct]
	3	2012.8	2018 Demographic and Health Survey [Other DHS Direct]
	30	2019.5	WHO Vital Registration (recalculated - unadjusted) [VR]
Turkmenistan	88	2015.5	
	3	1994.5	2000 Demographic and Health Survey [DHS Direct]
	2	2009.8	2016 Multiple Indicator Cluster Survey [Others Direct]
	2	2013.4	2019 Multiple Indicator Cluster Survey [Others Direct]
	81	2015.5	Recalculated based on WHO/UNPD - adjusted for incompleteness [VR]
Turks and Caicos Islands	16	2013.8	

Continued on next page

Table 25 – continued from previous page

Country	# obs.	Most recent obs. year	Data series name [source type]
	2	2013.8	2019-2020 Multiple Indicator Cluster Survey [Others Direct]
	14	2006.5	WHO Vital Registration (recalculated - unadjusted) [VR]
Tuvalu	11	2013.9	
	2	2013.9	2019-2020 Multiple Indicator Cluster Survey [Others Direct]
	9	2002	WHO Vital Registration (recalculated - unadjusted) [VR]
Uganda	95	2015.4	
	12	1987.2	1988-1989 Demographic and Health Survey [DHS Direct]
	12	1993.7	1995 Demographic and Health Survey [DHS Direct]
	12	1999.2	2000-2001 Demographic and Health Survey [DHS Direct]
	3	2006	2006 Demographic and Health Survey [DHS Direct]
	18	2005.3	2006 Demographic and Health Survey [DHS Direct]
	3	2003.8	2009-2010 Demographic and Health Survey [DHS Direct]
	17	2010.4	2011 Demographic and Health Survey [DHS Direct]
	18	2015.4	2016 Demographic and Health Survey [DHS Direct]
Ukraine	160	2021.5	
	3	1993.5	1999 Reproductive Health Survey [Others Direct]
	153	2021.5	WHO/Human Mortality Database Vital Registration (recalculated - unadjusted) [VR]
United Arab Emirates			
United Kingdom	201	2020.5	
	201	2020.5	WHO Vital Registration (recalculated - unadjusted) [VR]
United States of America	153	2020.5	
	153	2020.5	WHO/Human Mortality Database Vital Registration (recalculated - unadjusted) [VR]
Uruguay	93	2020.5	
	93	2020.5	WHO Vital Registration (recalculated - unadjusted) [VR]
Uzbekistan	102	2021.5	
	3	1996	2002 Demographic and Health Survey [Other DHS Direct]
	99	2021.5	Recalculated based on WHO - adjusted for incompleteness [VR]
Vanuatu	3	2009.4	
	3	2009.4	2009 Census [Others Direct]
Venezuela (Bolivarian Republic of)	81	2016.5	
	3	1971.2	1977 World Fertility Survey [Other DHS Direct]
	45	2016.5	Recalculated based on WHO/UNPD - adjusted for incompleteness [VR]
	33	2000.5	WHO Vital Registration (recalculated - unadjusted) [VR]
Vietnam	54	2020.3	
	3	1978.8	1979 Census [Others Direct]
	9	1986	1988 National Demographic and Health Survey [Other DHS Direct]
	3	1991.5	1997 Demographic and Health Survey [DHS Direct]
	3	1998.7	1999 Census [Others Direct]
	3	1996.7	2002 Demographic and Health Survey [DHS Direct]
	3	2006.8	2007 Population Change and Family Planning Survey [Others Direct]

Continued on next page

Table 25 – continued from previous page

Country	# obs.	Most recent obs. year	Data series name [source type]
	3	2010.8	2011 Population Change and Family Planning Survey [Others Direct]
	3	2011.8	2012 Population Change and Family Planning Survey [Others Direct]
	3	2008	2013-2014 Multiple Indicator Cluster Survey [Others Direct]
	3	2012.8	2013 Population Change and Family Planning Survey [Others Direct]
	3	2013.5	2014 Intercensal Demographic Survey [Others Direct]
	3	2014.8	2015 Population Change and Family Planning Survey [Others Direct]
	3	2016.5	2017 Population Change and Family Planning Survey [Others Direct]
	3	2017.5	2018 Population Change and Family Planning Survey [Others Direct]
	3	2020.3	2020 Population Change and Family Planning Survey [Others Direct]
	3	2014.9	2021 Multiple Indicator Cluster Survey [Others Direct]
Yemen	60	2007.7	
	6	1976.7	1979 World Fertility Survey [Other DHS Direct]
	9	1989.8	1991-1992 Demographic and Health Survey [DHS Direct]
	18	1996.7	1997 Demographic and Health Survey [DHS Direct]
	18	2002	2003 Pan Arab Project for Family Health Family Health Survey [Others Direct]
	3	2000.7	2006 Multiple Indicator Cluster Survey [Others Direct]
	6	2007.7	2013 Demographic and Health Survey [DHS Direct]
Zambia	95	2017	
	18	1991	1992 Demographic and Health Survey [DHS Direct]
	17	1995.5	1996-1997 Demographic and Health Survey [DHS Direct]
	18	2000.8	2001-2002 Demographic and Health Survey [DHS Direct]
	12	2005.7	2007 Demographic and Health Survey [DHS Direct]
	18	2012.6	2013-2014 Demographic and Health Survey [DHS Direct]
	12	2017	2018 Demographic and Health Survey [DHS Direct]
Zimbabwe	62	2016.5	
	6	1985.7	1988-1989 Demographic and Health Survey [DHS Direct]
	3	1992.1	1992 Census [Others Direct]
	6	1991.5	1994 Demographic and Health Survey [DHS Direct]
	3	1993.7	1999 Demographic and Health Survey [DHS Direct]
	6	2006.3	2009 Multiple Indicator Cluster Survey [Others Direct]
	8	2008.7	2010-2011 Demographic and Health Survey [DHS Direct]
	3	2012.1	2012 Census [Others Direct]
	12	2012.7	2014 Multiple Indicator Cluster Survey [Others Direct]
	6	2012.5	2015 Demographic and Health Survey [DHS Direct]
	3	2016.5	2017 Inter-Censal Demographic Survey [Others Direct]
	6	2016.1	2019 Multiple Indicator Cluster Survey [Others Direct]

Table 26: **Sex ratio data sources for age group 10–14, by country.** For each country, the total number of observations and the most recent reference year are shown after the country name. For each country-specific data series, the number of observations and the most recent reference year within that series is shown before each data series name. The source type that each data series falls in is shown in parentheses after each data series name.

**Country**	**# obs.**	**Most recent obs. year**	**Data series name [source type]**
Afghanistan	42	2015.2	
	3	1973	1972 National Demographic and Family Guidance Survey [Others Direct]
	3	1979	1979 Census [Others Direct]
	3	2008.1	2010 Afghanistan Mortality Survey (AMS) Excluding South Zone (Household data) [Other DHS Direct]
	18	2009	2010 Afghanistan Mortality Survey (AMS) [DHS Direct]
	9	2013.4	2015 Demographic and Health Survey [DHS Direct]
	6	2015.2	2018 Afghanistan Health Survey 2018 [Others Direct]
Albania	77	2021.5	
	3	2002.7	2008-2009 Demographic and Health Survey [DHS Direct]
	3	2011.1	2011 Census [Others Direct]
	3	2011.7	2018 Demographic and Health Survey [DHS Direct]
	66	2021.5	WHO Vital Registration (recalculated - unadjusted) [VR]
Algeria	72	2020.5	
	6	1989	1992 Pan Arab Project for Child Development Maternal and Child Health Survey [Others Direct]
	3	1996	2002 Pan Arab Project for Family Health Family Health Survey [Others Direct]
	63	2020.5	WHO Vital Registration (recalculated - unadjusted) [VR]
Andorra	10	2018.5	
	10	2018.5	WHO Vital Registration (recalculated - unadjusted) [VR]
Angola	12	2014.3	
	12	2014.3	2015-2016 Inquerito de Indicadores Multiplos e de Saude [DHS Direct]
Anguilla			
Antigua and Barbuda	18	2016.5	
	18	2016.5	WHO Vital Registration (recalculated - unadjusted) [VR]
Argentina	96	2020.5	
	96	2020.5	WHO Vital Registration (recalculated - unadjusted) [VR]
Armenia	108	2021.5	
	3	1994.7	2000 Demographic and Health Survey [DHS Direct]
	3	1999.7	2005 Demographic and Health Survey [DHS Direct]
	3	2004.7	2010 Demographic and Health Survey [DHS Direct]
	99	2021.5	WHO Vital Registration (recalculated - unadjusted) [VR]
Australia	153	2020.5	
	153	2020.5	WHO/Human Mortality Database Vital Registration (recalculated - unadjusted) [VR]
Austria	153	2020.5	
	153	2020.5	WHO/Human Mortality Database Vital Registration (recalculated - unadjusted) [VR]

Continued on next page

Table 26 – continued from previous page

Country	# obs.	Most recent obs. year	Data series name [source type]
Azerbaijan	92	2021.5	
	2	2000.5	2006 Demographic and Health Survey [DHS Direct]
	3	2005	2011 Azerbaijan National Demographic and Health Survey [Other DHS Direct]
	87	2021.5	Recalculated based on WHO - adjusted for incompleteness [VR]
Bahamas	86	2020.5	
	86	2020.5	Recalculated based on WHO/UNPD - adjusted for incompleteness [VR]
Bahrain	93	2019.5	
	93	2019.5	WHO/UNPD Vital Registration (recalculated - unadjusted) [VR]
Bangladesh	261	2020.5	
	3	1964	1962-1965 Population Growth Estimation Experiment [Others Direct]
	3	1974.5	1974 Retrospective Fertility and Mortality Survey (UN SA) [Others Direct]
	18	1975	1976 World Fertility Survey [Other DHS Direct]
	18	1992.8	1993-1994 Demographic and Health Survey [DHS Direct]
	15	1995.8	1996-1997 Demographic and Health Survey [DHS Direct]
	3	1999.8	1999-2000 Demographic and Health Survey [DHS Direct]
	18	1998.8	1999-2000 Demographic and Health Survey [DHS Direct]
	18	2003	2004 Demographic and Health Survey [DHS Direct]
	9	2005.2	2007 Demographic and Health Survey [DHS Direct]
	3	2010	2010 Maternal Health Services and Maternal Mortality Survey [Other DHS Direct]
	3	2010.7	2011 Census [Others Direct]
	18	2010.5	2011 Demographic and Health Survey [DHS Direct]
	12	2012.9	2014 Demographic and Health Survey [DHS Direct]
	3	2016	2016 Maternal Health Services and Maternal Mortality Survey [Others Direct]
	12	2016.2	2017-2018 Demographic and Health Survey [DHS Direct]
	18	2018.1	2019 Multiple Indicator Cluster Survey [Others Direct]
	3	2016.5	Report on Sample Vital Registration System 2016 [Others Direct]
	3	2017.5	Report on Sample Vital Registration System 2016 [Others Direct]
	3	2018.5	Report on Sample Vital Registration System 2016 [Others Direct]
	3	2019.5	Report on Sample Vital Registration System 2016 [Others Direct]
	3	2020.5	Report on Sample Vital Registration System 2016 [Others Direct]
	72	2015.5	Sample Vital Statistics [VR]
Barbados	54	2013	
	54	2013	WHO Vital Registration (recalculated - unadjusted) [VR]
Belarus	150	2019.5	
	150	2019.5	WHO/Human Mortality Database Vital Registration (recalculated - unadjusted) [VR]
Belgium	153	2020.5	
	153	2020.5	WHO/Human Mortality Database Vital Registration (recalculated - unadjusted) [VR]
Belize	85	2018.5	
	2	2009.8	2015-2016 Multiple Indicator Cluster Survey [Others Direct]

Continued on next page

Table 26 – continued from previous page

Country	# obs.	Most recent obs. year	Data series name [source type]
	81	2018.5	Recalculated based on WHO - adjusted for incompleteness [VR]
Benin	107	2016.8	
	17	1981	1982 World Fertility Survey [Other DHS Direct]
	18	1995.4	1996 Demographic and Health Survey [DHS Direct]
	18	2000.6	2001 Demographic and Health Survey [DHS Direct]
	18	2005.6	2006 Demographic and Health Survey [DHS Direct]
	18	2013.5	2014 Multiple Indicator Cluster Survey [Others Direct]
	18	2016.8	2017-2018 Demographic and Health Survey [DHS Direct]
Bhutan	9	2012.5	
	3	1993.1	1994 National Health Survey [Others Direct]
	3	2004.9	2005 Census [Others Direct]
	3	2012.5	2012 National Health Survey [Others Direct]
Bolivia (Plurinational State of)	51	2010.4	
	3	1979.8	1980 National Demographic Survey [Others Direct]
	6	1986.2	1989 Demographic and Health Survey [DHS Direct]
	9	1991.8	1993-1994 Demographic and Health Survey [DHS Direct]
	9	1996.2	1998 Demographic and Health Survey [DHS Direct]
	12	2002.1	2003 Demographic and Health Survey [DHS Direct]
	9	2006.1	2008 Demographic and Health Survey [DHS Direct]
	3	2010.4	2016 Encuesta de Demografma y Salud [Others Direct]
Bosnia and Herzegovina	75	2020.5	
	75	2020.5	WHO Vital Registration (recalculated - unadjusted) [VR]
Botswana	18	2016.5	
	3	1981.1	1981 Census [Others Direct]
	3	1991.1	1991 Census [Others Direct]
	3	2001.1	2001 Census [Others Direct]
	3	2001.8	2008 Family Health Survey [Others Direct]
	3	2011.1	2011 Census [Others Direct]
	3	2016.5	2017 Demographic Survey [Others Direct]
Brazil	104	2020.5	
	3	1990.2	1996 Demographic and Health Survey [DHS Direct]
	3	2010.2	2010 Census [Others Direct]
	96	2020.5	WHO Vital Registration (recalculated - unadjusted) [VR]
British Virgin Islands			
Brunei	87	2019.5	
	87	2019.5	WHO Vital Registration (recalculated - unadjusted) [VR]
Bulgaria	156	2021.5	
	156	2021.5	WHO/Human Mortality Database Vital Registration (recalculated - unadjusted) [VR]
Burkina Faso	70	2009.3	
	3	1960.5	1960-1961 Survey [Others Direct]
	12	1991.4	1993 Demographic and Health Survey [DHS Direct]

Continued on next page

Table 26 – continued from previous page

Country	# obs.	Most recent obs. year	Data series name [source type]
	16	1997.8	1998-1999 Demographic and Health Survey [DHS Direct]
	18	2002.4	2003 Demographic and Health Survey [DHS Direct]
	3	2007.3	2008 Global Fund Evaluation Survey [Others Direct]
	18	2009.3	2010 Demographic and Health Survey [DHS Direct]
Burundi	53	2015.7	
	3	1964.7	1965 Population Survey [Others Direct]
	3	1969.5	1970 Demographic Survey [Others Direct]
	11	1985.7	1987 Demographic and Health Survey [DHS Direct]
	18	2009.6	2010-2011 Demographic and Health Survey [DHS Direct]
	18	2015.7	2016-2017 Demographic and Health Survey [DHS Direct]
Cambodia	57	2012.4	
	18	1999.1	2000 Demographic and Health Survey [DHS Direct]
	18	2004.7	2005 Demographic and Health Survey [DHS Direct]
	12	2009	2010 Demographic and Health Survey [DHS Direct]
	9	2012.4	2014 Demographic and Health Survey [DHS Direct]
Cameroon	99	2016.9	
	3	1975.8	1976 Census [Others Direct]
	12	1976.5	1978 World Fertility Survey [Other DHS Direct]
	3	1986.8	1987 Census [Others Direct]
	9	1989.2	1991 Demographic and Health Survey [DHS Direct]
	12	1996.6	1998 Demographic and Health Survey [DHS Direct]
	18	2003.1	2004 Demographic and Health Survey [DHS Direct]
	18	2010	2011 Demographic and Health Survey [DHS Direct]
	12	2013	2014 Multiple Indicator Cluster Surveys [Others Direct]
	12	2016.9	2018 Demographic and Health Survey [DHS Direct]
Canada	153	2020.5	
	153	2020.5	WHO/Human Mortality Database Vital Registration (recalculated - unadjusted) [VR]
Cape Verde	78	2018.5	
	3	1992.2	1998 Demographic and Reproductive Health Survey [Others Direct]
	3	2000	2000 Census [Others Direct]
	3	2009.5	2010 Census [Others Direct]
	18	1999.5	Recalculated based on WHO/UNPD - adjusted for incompleteness [VR]
	51	2018.5	WHO/UNPD Vital Registration (recalculated - unadjusted) [VR]
Central African Republic	39	2017.4	
	3	1959.5	1959-1960 Survey [Others Direct]
	3	1988.4	1988 Census [Others Direct]
	18	1993.7	1994-1995 Demographic and Health Survey [DHS Direct]
	3	2003.4	2003 Census [Others Direct]
	12	2017.4	2019 Multiple Indicator Cluster Survey [Others Direct]
Chad	75	2018.3	
	18	1995.9	1996-1997 Demographic and Health Survey [DHS Direct]
	18	2003.5	2004 Demographic and Health Survey [DHS Direct]
	3	2008.9	2009 Census [Others Direct]

Continued on next page

Table 26 – continued from previous page

Country	# obs.	Most recent obs. year	Data series name [source type]
	18	2013.7	2014-2015 Demographic and Health Survey [DHS Direct]
	18	2018.3	2019 Multiple Indicator Cluster Survey [Others Direct]
Chile	150	2020.5	
	150	2020.5	WHO/Human Mortality Database/UNPD Vital Registration (recalculated - unadjusted) [VR]
China	33	2010.5	
	3	1973.5	1964-1982 Adjusted Census Deaths (1964-1982) [Others Direct]
	3	1986.5	1982-1990 Adjusted Census Deaths (1982-1990) [Others Direct]
	3	1995.5	1990-2000 Adjusted Census Deaths (1990-2000) [Others Direct]
	3	2000.5	1999-2000 Adjusted Census Deaths (1999-2000) [Others Direct]
	3	2005.5	2000-2010 Adjusted Census Deaths (2000-2010) [Others Direct]
	18	2010.5	China CDC Surveillance surveys [VR]
Colombia	117	2020.5	
	3	1980.7	1986 Demographic and Health Survey [DHS Direct]
	3	1984.3	1990 Demographic and Health Survey [DHS Direct]
	3	1989.2	1995 Demographic and Health Survey [DHS Direct]
	3	1994.1	2000 Demographic and Health Survey [DHS Direct]
	3	2005.4	2005 Census [Others Direct]
	6	2001.7	2005 Demographic and Health Survey [DHS Direct]
	9	2007.8	2010 Demographic and Health Survey [DHS Direct]
	3	2009.1	2015 Demographic and Health Survey [DHS Direct]
	84	2020.5	Recalculated based on WHO - adjusted for incompleteness [VR]
Comoros	15	2017.6	
	3	1958.2	1958 Census [Others Direct]
	3	1990.2	1996 Demographic and Health Survey [DHS Direct]
	3	2003.2	2003 Census [Others Direct]
	3	2006.6	2012 Demographic and Health Survey [DHS Direct]
	3	2017.6	2017 Census [Others Direct]
Congo	23	2011.9	
	8	2003.5	2005 Demographic and Health Survey [DHS Direct]
	9	2009.7	2011-2012 Demographic and Health Survey [DHS Direct]
	6	2011.9	2014-2015 Multiple Indicator Cluster Survey [Others Direct]
Democratic Republic of the Congo	30	2015	
	9	2005.5	2007 Demographic and Health Survey [DHS Direct]
	15	2012.6	2013-2014 Demographic and Health Survey [DHS Direct]
	6	2015	2018 Multiple Indicator Cluster Survey [Others Direct]
Cook Islands	12	2008	
	12	2008	WHO Vital Registration (recalculated - unadjusted) [VR]
Costa Rica	96	2020.5	
	96	2020.5	Recalculated based on WHO - adjusted for incompleteness [VR]
Cote d'Ivoire	71	2014.8	
	3	1977.5	1978-1979 Demographic Survey Repeated Passages [Others Direct]
	17	1979.6	1981 World Fertility Survey [Other DHS Direct]
	18	1993.4	1994 Demographic and Health Survey [DHS Direct]

Continued on next page

Table 26 – continued from previous page

Country	# obs.	Most recent obs. year	Data series name [source type]
	6	1995.7	1998-1999 Demographic and Health Survey [DHS Direct]
	3	1999.6	2005 National Demographic and Health Survey [Other DHS Direct]
	12	2010.4	2011-2012 Demographic and Health Survey [DHS Direct]
	12	2014.8	2016 Multiple Indicator Cluster Survey [Others Direct]
Croatia	117	2021.5	
	3	2010.8	2011 Census [Others Direct]
	114	2021.5	WHO/Human Mortality Database Vital Registration (recalculated - unadjusted) [VR]
Cuba	96	2020.5	
	96	2020.5	WHO Vital Registration (recalculated - unadjusted) [VR]
Cyprus	82	2019.5	
	46	2019.5	Recalculated based on WHO - adjusted for incompleteness [VR]
	36	2000.5	WHO Vital Registration (recalculated - unadjusted) [VR]
Czech Republic	156	2021.5	
	156	2021.5	WHO/Human Mortality Database Vital Registration (recalculated - unadjusted) [VR]
Denmark	156	2021.5	
	156	2021.5	WHO/Human Mortality Database Vital Registration (recalculated - unadjusted) [VR]
Djibouti	6	2006.5	
	3	1996.8	2002 Pan Arab Project for Family Health Family Health Survey [Others Direct]
	3	2006.5	2012 Pan Arab Project for Family Health Family Health Survey [Others Direct]
Dominica	32	2015.5	
	11	2000.5	Recalculated based on WHO - adjusted for incompleteness [VR]
	21	2015.5	WHO Vital Registration (recalculated - unadjusted) [VR]
Dominican Republic	39	2013.8	
	3	1969	1975 World Fertility Survey [Other DHS Direct]
	3	1972.3	1980 World Fertility Survey [Other DHS Direct]
	6	1983.7	1986 Demographic and Health Survey [DHS Direct]
	3	1985.5	1991 Demographic and Health Survey [DHS Direct]
	3	1990.7	1996 Demographic and Health Survey [DHS Direct]
	6	1999.5	2002 Demographic and Health Survey [DHS Direct]
	6	2004.2	2007 Demographic and Health Survey [DHS Direct]
	3	2007.5	2013 Demographic and Health Survey [DHS Direct]
	3	2008.5	2014 Multiple Indicator Cluster Survey [Others Direct]
	3	2013.8	2019 Multiple Indicator Cluster Survey [Others Direct]
Ecuador	117	2020.5	
	3	1981	1987 Demographic and Health Survey [DHS Direct]
	6	1991.4	1994 Demographic and Maternal and Child Health Survey [Others Direct]
	9	1997.2	1999 Demographic and Maternal and Child Health Survey [Others Direct]

Continued on next page

Table 26 – continued from previous page

Country	# obs.	Most recent obs. year	Data series name [source type]
	3	1998.6	2004 Demographic and Maternal and Child Health Survey [Others Direct]
	3	2006.6	2012 Encuesta Nacional de Salud y Nutricion: Salud Sexual y Reproductiva [Others Direct]
	3	2012.9	2018 Encuesta Nacional de Salud y Nutricion [Others Direct]
	90	2020.5	WHO Vital Registration (recalculated - unadjusted) [VR]
Egypt	201	2019.5	
	18	1979.1	1980 World Fertility Survey [Other DHS Direct]
	12	1987.2	1988 Demographic and Health Survey [DHS Direct]
	12	1989.5	1991 Pan Arab Project for Child Development Maternal and Child Health Survey [Others Direct]
	12	1991.3	1992 Demographic and Health Survey [DHS Direct]
	12	1994.3	1995 Demographic and Health Survey [DHS Direct]
	12	1998.6	2000 Demographic and Health Survey [DHS Direct]
	6	2000.3	2003 Interim Demographic and Health Survey [DHS Direct]
	12	2003.7	2005 Demographic and Health Survey [DHS Direct]
	6	2005.2	2008 Demographic and Health Survey [DHS Direct]
	6	2011.2	2014 Demographic and Health Survey [DHS Direct]
	42	2019.5	Recalculated based on WHO - adjusted for incompleteness [VR]
	51	2005.5	WHO Vital Registration (recalculated - unadjusted) [VR]
El Salvador	101	2019.5	
	3	1992.2	1992 Census [Others Direct]
	3	1992.3	1998 National Family Health Survey [Others Direct]
	3	1996.9	2002 National Family Health Survey [Others Direct]
	3	2006.9	2007 Census [Others Direct]
	3	2002.3	2008 National Family Health Survey [Others Direct]
	3	2008.2	2014 Multiple Indicator Cluster Survey [Others Direct]
	81	2019.5	WHO/UNPD Vital Registration (recalculated - unadjusted) [VR]
Equatorial Guinea			
Eritrea	39	2009.1	
	9	1993.7	1995-1996 Demographic and Health Survey [DHS Direct]
	12	2000.7	2002 Demographic and Health Survey [DHS Direct]
	18	2009.1	2010 Population and Health Survey [Other DHS Direct]
Estonia	156	2020.5	
	3	2011.6	2011-2012 Census [Others Direct]
	153	2020.5	WHO/Human Mortality Database Vital Registration (recalculated - unadjusted) [VR]
Ethiopia	72	2016.2	
	18	1999.1	2000 Demographic and Health Survey [DHS Direct]
	18	2004.2	2005 Demographic and Health Survey [DHS Direct]
	18	2009.9	2011 Demographic and Health Survey [DHS Direct]
	12	2014.5	2016 Demographic and Health Survey [DHS Direct]
	6	2016.2	2019 Demographic and Health Survey [DHS Direct]
Federated States of Micronesia			
Fiji	63	2019.5	

Continued on next page

Table 26 – continued from previous page

Country	# obs.	Most recent obs. year	Data series name [source type]
	6	1971.1	1974 World Fertility Survey [Other DHS Direct]
	57	2019.5	Recalculated based on WHO - adjusted for incompleteness [VR]
Finland	156	2021.5	
	156	2021.5	WHO/Human Mortality Database Vital Registration (recalculated - unadjusted) [VR]
France	153	2020.5	
	153	2020.5	WHO/Human Mortality Database Vital Registration (recalculated - unadjusted) [VR]
Gabon	12	2006	
	9	1998.5	2000 Demographic and Health Survey [DHS Direct]
	3	2006	2012 Demographic and Health Survey [DHS Direct]
The Gambia	24	2017.8	
	3	2012.8	2013 Census [Others Direct]
	12	2016.6	2018 Multiple Indicator Cluster Survey [Others Direct]
	9	2017.8	2019-2020 Demographic and Health Survey [DHS Direct]
Georgia	98	2021.5	
	3	1993.9	1999 Reproductive Health Survey [Others Direct]
	3	1999.2	2005 Reproductive Health Survey [Others Direct]
	2	2004.8	2010 Reproductive Health Survey [Others Direct]
	33	2001.5	Recalculated based on WHO - adjusted for incompleteness [VR]
	57	2021.5	WHO Vital Registration (recalculated - unadjusted) [VR]
Germany	153	2021.5	
	153	2021.5	WHO/Human Mortality Database Vital Registration (recalculated - unadjusted) [VR]
Ghana	94	2016.3	
	11	1986.6	1988 Demographic and Health Survey [DHS Direct]
	12	1992.2	1993-1994 Demographic and Health Survey [DHS Direct]
	9	1996.8	1998-1999 Demographic and Health Survey [DHS Direct]
	6	2000.5	2003 Demographic and Health Survey [DHS Direct]
	6	2005.7	2008 Demographic and Health Survey [DHS Direct]
	3	2010.3	2010 Census [Others Direct]
	9	2009.8	2011 Multiple Indicator Cluster Survey [Others Direct]
	8	2012.7	2014 Demographic and Health Survey [DHS Direct]
	18	2013.7	2017 Demographic and Health Survey [DHS Direct]
	12	2016.3	2017-2018 Multiple Indicator Cluster Survey [Others Direct]
Greece	153	2020.5	
	153	2020.5	WHO/Human Mortality Database Vital Registration (recalculated - unadjusted) [VR]
Grenada	61	2018	
	25	1999.5	Recalculated based on WHO - adjusted for incompleteness [VR]
	36	2018	WHO Vital Registration (recalculated - unadjusted) [VR]
Guatemala	123	2020.5	
	6	1984.7	1987 Demographic and Health Survey [DHS Direct]
	12	1993.9	1995 Demographic and Health Survey [DHS Direct]
	3	1992.8	1998-1999 Interim Demographic and Health Survey [DHS Direct]

Continued on next page

Table 26 – continued from previous page

Country	# obs.	Most recent obs. year	Data series name [source type]
	3	1996.3	2002 Encuesta Nacional de Salud Materno Infantil (ENSMI) [Others Direct]
	3	2002.8	2008 Encuesta Nacional de Salud Materno Infantil (ENSMI) [Others Direct]
	3	2011.7	2014-2015 Demographic and Health Survey [DHS Direct]
	93	2020.5	WHO Vital Registration (recalculated - unadjusted) [VR]
Guinea	81	2017.1	
	3	1954.5	1954-1955 Survey [Others Direct]
	3	1996.4	1996 Census [Others Direct]
	18	1998.3	1999 Demographic and Health Survey [DHS Direct]
	18	2004.1	2005 Demographic and Health Survey [DHS Direct]
	18	2011.4	2012 Demographic and Health Survey [DHS Direct]
	3	2013.8	2014 Census [Others Direct]
	18	2017.1	2018 Demographic and Health Survey [DHS Direct]
Guinea-Bissau	37	2016.1	
	10	2008.8	2010 Multiple Indicator Cluster Survey [Others Direct]
	18	2013.2	2014 Multiple Indicator Cluster Survey [Others Direct]
	9	2016.1	2018-2019 Multiple Indicator Cluster Survey [Others Direct]
Guyana	99	2014.5	
	6	1972.4	1975 World Fertility Survey [Other DHS Direct]
	3	1999.4	2005 AIDS Indicator Survey [Other DHS Direct]
	3	2003.2	2009 Demographic and Health Survey [DHS Direct]
	3	2012.2	2012 Census [Others Direct]
	3	2008.3	2014 Multiple Indicator Cluster Survey [Others Direct]
	3	2013.5	2019-2020 Multiple Indicator Cluster Survey [Others Direct]
	78	2014.5	WHO Vital Registration (recalculated - unadjusted) [VR]
Haiti	44	2014.8	
	8	1975.5	1977 World Fertility Survey [Other DHS Direct]
	9	1992.5	1994-1995 Demographic and Health Survey [DHS Direct]
	6	1997.1	2000 Demographic and Health Survey [DHS Direct]
	9	2001.2	2005-2006 Demographic and Health Survey [DHS Direct]
	6	2007.5	2012 Demographic and Health Survey [DHS Direct]
	6	2014.8	2016-2017 Demographic and Health Survey [DHS Direct]
Honduras	38	2016.5	
	3	1971.3	1970-1972 National Demographic Survey [Others Direct]
	3	1982.5	1983 National Demographic Survey [Others Direct]
	3	1990.1	1996 Reproductive Health Survey [Others Direct]
	3	2001.1	2001 Census [Others Direct]
	3	1992.2	2001 Reproductive Health Survey [Others Direct]
	8	2003.7	2005-2006 Demographic and Health Survey [DHS Direct]
	6	2008.7	2011-2012 Demographic and Health Survey [DHS Direct]
	3	2013.1	2013 Census [Others Direct]
	6	2016.5	2019 Multiple Indicator Cluster Survey [Others Direct]
Hungary	153	2020.5	
	153	2020.5	WHO/Human Mortality Database Vital Registration (recalculated - unadjusted) [VR]

Continued on next page

Table 26 – continued from previous page

Country	# obs.	Most recent obs. year	Data series name [source type]
Iceland	91	2018.5	
	91	2018.5	WHO/Human Mortality Database Vital Registration (recalculated - unadjusted) [VR]
India	177	2020.5	
	3	1970.5	1971 Census [Others Direct]
	3	1980.5	1981 Census [Others Direct]
	18	1991.2	1992-1993 Demographic and Health Survey [DHS Direct]
	3	1991.9	1992-1993 National Family Health Survey [DHS Direct]
	18	1997.8	1998-1999 Demographic and Health Survey [DHS Direct]
	3	1998.2	1998-1999 National Family Health Survey [DHS Direct]
	18	2004.8	2005-2006 Demographic and Health Survey [DHS Direct]
	111	2020.5	Sample Registration System [VR]
Indonesia	147	2016.6	
	3	1964.5	1964-1965 National Socio-economic Survey [Others Direct]
	18	1975.2	1976 World Fertility Survey [Other DHS Direct]
	18	1986.7	1987 Demographic and Health Survey [DHS Direct]
	18	1990.3	1991 Demographic and Health Survey [DHS Direct]
	18	1993.5	1994 Demographic and Health Survey [DHS Direct]
	18	1996.7	1997 Demographic and Health Survey [DHS Direct]
	12	2001.2	2002-2003 Demographic and Health Survey [DHS Direct]
	12	2005.9	2007 Demographic and Health Survey [DHS Direct]
	3	2009.9	2010 Census [Others Direct]
	9	2010.8	2012 Demographic and Health Survey [DHS Direct]
	18	2016.6	2017 Demographic and Health Survey [DHS Direct]
Iran (Islamic Republic of)	42	2017.5	
	3	1975	1973-1976 Population Growth Survey [Others Direct]
	3	1985.5	1986 Census [Others Direct]
	3	1999.5	2000 Demographic and Health Survey [Other DHS Direct]
	33	2017.5	Recalculated based on WHO - adjusted for incompleteness [VR]
Iraq	24	2012.2	
	9	2004.2	2006 Multiple Indicator Cluster Survey [Others Direct]
	9	2009.2	2011 Multiple Indicator Cluster Survey [Others Direct]
	3	2012.2	2018 Multiple Indicator Cluster Survey [Others Direct]
	3	1974	Demographic Sample Survey and Sample Registration System [VR]
Ireland	153	2020.5	
	153	2020.5	WHO/Human Mortality Database Vital Registration (recalculated - unadjusted) [VR]
Israel	141	2021.5	
	141	2021.5	WHO/Human Mortality Database Vital Registration (recalculated - unadjusted) [VR]
Italy	153	2020.5	
	153	2020.5	WHO/Human Mortality Database Vital Registration (recalculated - unadjusted) [VR]
Jamaica	3	2010.2	

Continued on next page

Table 26 – continued from previous page

Country	# obs.	Most recent obs. year	Data series name [source type]
	3	2010.2	2011 Census [Others Direct]
Japan	150	2021.5	
	150	2021.5	WHO/Human Mortality Database Vital Registration (recalculated - unadjusted) [VR]
Jordan	20	2011.7	
	3	1984.7	1990 Demographic and Health Survey [DHS Direct]
	3	1991.4	1997 Demographic and Health Survey [DHS Direct]
	3	1996.5	2002 Demographic and Health Survey [DHS Direct]
	3	2001.4	2007 Demographic and Health Survey [DHS Direct]
	3	2006.7	2012 Demographic and Health Survey [DHS Direct]
	3	2011.7	2017 Demographic and Health Survey [DHS Direct]
Kazakhstan	105	2021.5	
	3	1989.3	1995 Demographic and Health Survey [DHS Direct]
	3	1993.5	1999 Demographic and Health Survey [DHS Direct]
	99	2021.5	WHO Vital Registration (recalculated - unadjusted) [VR]
Kenya	73	2013.3	
	11	1976.1	1978 World Fertility Survey [Other DHS Direct]
	6	1985.9	1989 Demographic and Health Survey [DHS Direct]
	9	1991.1	1993 Demographic and Health Survey [DHS Direct]
	11	1996.6	1998 Demographic and Health Survey [DHS Direct]
	12	2001.7	2003 Demographic and Health Survey [DHS Direct]
	6	2005.8	2008-2009 Demographic and Health Survey [DHS Direct]
	18	2013.3	2014 Demographic and Health Survey [DHS Direct]
Kiribati	3	2012.9	
	3	2012.9	2019 Multiple Indicator Cluster Survey [Others Direct]
Democratic People's Republic of Korea	6	2008.3	
	3	1993.5	1993 Census [Others Direct]
	3	2008.3	2008 Census [Others Direct]
Republic of Korea	105	2021.5	
	6	1971.7	1974 World Fertility Survey [Other DHS Direct]
	99	2021.5	WHO/Human Mortality Database Vital Registration (recalculated - unadjusted) [VR]
Kosovo	4	2014	
	2	2007.9	2013-2014 Multiple Indicator Cluster Survey [Others Direct]
Kuwait	87	2019.5	
	87	2019.5	WHO Vital Registration (recalculated - unadjusted) [VR]
Kyrgyzstan	110	2021.5	
	3	1991.6	1997 Demographic and Health Survey [DHS Direct]
	3	2008.3	2014 Multiple Indicator Cluster Survey [Others Direct]
	3	2012.8	2018 Multiple Indicator Cluster Survey [Others Direct]
	99	2021.5	WHO Vital Registration (recalculated - unadjusted) [VR]
Lao People's Democratic Republic	51	2016.1	

Continued on next page

Table 26 – continued from previous page

Country	# obs.	Most recent obs. year	Data series name [source type]
	3	2004.8	2005 Census [Others Direct]
	18	2004.8	2005 Reproductive Health Survey [Others Direct]
	18	2010.8	2012 Lao Social Indicator Survey (combined Multiple Indicator Cluster Survey4/Demographic and Health Survey) [DHS Direct]
	12	2016.1	2017 Multiple Indicator Cluster Survey [Others Direct]
Latvia	153	2021.5	
	153	2021.5	WHO/Human Mortality Database Vital Registration (recalculated - unadjusted) [VR]
Lebanon	3	1990	
	3	1990	1996 Pan Arab Project for Child Development Maternal and Child Health Survey [Others Direct]
Lesotho	27	2015.5	
	3	1971.9	1971-1973 Demographic Survey [Others Direct]
	6	1974.6	1977 World Fertility Survey [Other DHS Direct]
	3	2000.9	2001 Demographic Survey [Others Direct]
	3	1998.7	2004 Demographic and Health Survey [DHS Direct]
	3	2003.7	2009 Demographic and Health Survey [DHS Direct]
	3	2008.7	2014 Demographic and Health Survey [DHS Direct]
	3	2015.5	2016 Census [Others Direct]
	3	2012.3	2018 Multiple Indicator Cluster Survey [Others Direct]
Liberia	51	2017.7	
	3	1969.5	1969-1970 Population Growth Survey [Others Direct]
	6	1983.1	1986 Demographic and Health Survey [DHS Direct]
	9	2004.9	2006-2007 Demographic and Health Survey [DHS Direct]
	12	2007.4	2008-2009 Malaria Indicator Survey [Other DHS Direct]
	12	2011.7	2013 Demographic and Health Survey [DHS Direct]
	9	2017.7	2019-2020 Demographic and Health Survey [DHS Direct]
Libya	12	2004.4	
	6	1992	1995 Pan Arab Project for Child Development Maternal and Child Health Survey [Others Direct]
	6	2004.4	2007 Pan Arab Project for Family Health Family Health Survey [Others Direct]
Lithuania	162	2021.5	
	3	2010.8	2011 Census [Others Direct]
	3	2017.5	2018 Statistical Survey [Others Direct]
	156	2021.5	WHO/Human Mortality Database Vital Registration (recalculated - unadjusted) [VR]
Luxembourg	90	2020.5	
	90	2020.5	WHO/Human Mortality Database Vital Registration (recalculated - unadjusted) [VR]
Macedonia	93	2020.5	
	93	2020.5	Recalculated based on WHO - adjusted for incompleteness [VR]
Madagascar	87	2020.2	
	3	1966.1	1966 Demographic Survey [Others Direct]
	3	1993.1	1993 Census [Others Direct]
	18	1996.7	1997 Demographic and Health Survey [DHS Direct]

Continued on next page

Table 26 – continued from previous page

Country	# obs.	Most recent obs. year	Data series name [source type]
	9	2001.8	2003-2004 Demographic and Health Survey [DHS Direct]
	18	2007.8	2008-2009 Demographic and Health Survey [DHS Direct]
	18	2017.7	2018 Multiple Indicator Cluster Survey [Others Direct]
	18	2020.2	2021 Demographic and Health Survey [DHS Direct]
Malawi	152	2019	
	3	1977.2	1977 Census [Others Direct]
	3	1983.9	1984 Family Formation Survey [Others Direct]
	3	1987.2	1987 Census [Others Direct]
	12	1991.2	1992 Demographic and Health Survey [DHS Direct]
	18	1999.5	2000 Demographic and Health Survey [DHS Direct]
	18	2003.7	2004 Demographic and Health Survey [DHS Direct]
	18	2005.6	2006 MDG Endline Survey [Others Direct]
	18	2009.4	2010 Demographic and Health Survey [DHS Direct]
	18	2012.9	2013-2014 MDG Endline Survey [Others Direct]
	18	2014.7	2015-2016 Demographic and Health Survey [DHS Direct]
	3	2018.2	2018 Census [Others Direct]
	18	2019	2019-2020 MDG Endline Survey [Others Direct]
Malaysia	99	2021.5	
	9	1972.6	1974 World Fertility Survey [Other DHS Direct]
	90	2021.5	WHO Vital Registration (recalculated - unadjusted) [VR]
Maldives	93	2020.5	
	3	2003	2009 Demographic and Health Survey [DHS Direct]
	3	2010.2	2016-2017 Demographic and Health Survey [DHS Direct]
	18	1994.5	Recalculated based on WHO - adjusted for incompleteness [VR]
	69	2020.5	WHO Vital Registration (recalculated - unadjusted) [VR]
Mali	107	2017.6	
	3	1976.4	1976 Census [Others Direct]
	12	1985.7	1987 Demographic and Health Survey [DHS Direct]
	18	1994.8	1995-1996 Demographic and Health Survey [DHS Direct]
	3	1997.8	1998 Census [Others Direct]
	18	2000	2001 Demographic and Health Survey [DHS Direct]
	18	2005.3	2006 Demographic and Health Survey [DHS Direct]
	18	2014.6	2015 Multiple Indicator Cluster Survey [Others Direct]
	17	2017.6	2018 Demographic and Health Survey [DHS Direct]
Malta	51	2021.5	
	51	2021.5	WHO Vital Registration (recalculated - unadjusted) [VR]
Marshall Islands			
Mauritania	63	2017.9	
	3	1977	1976-1977 Census [Others Direct]
	12	1979.8	1981 World Fertility Survey [Other DHS Direct]
	3	1987.8	1988 Census [Others Direct]
	12	1988.5	1990 Pan Arab Project for Child Development Maternal and Child Health Survey [Others Direct]
	6	1997.6	2000-2001 Demographic and Health Survey [DHS Direct]
	9	2009.5	2011 Multiple Indicator Cluster Survey [Others Direct]
	9	2013.6	2015 Multiple Indicator Cluster Survey [Others Direct]

Continued on next page

Table 26 – continued from previous page

Country	# obs.	Most recent obs. year	Data series name [source type]
	9	2017.9	2021 Demographic and Health Survey [DHS Direct]
Mauritius	96	2020.5	
	96	2020.5	WHO Vital Registration (recalculated - unadjusted) [VR]
Mexico	132	2020.5	
	3	1981.1	1987 Demographic and Health Survey [DHS Direct]
	12	2007.6	2009 Encuesta Nacional de la Dinamica Demografica (ENADID) [Others Direct]
	9	2012.7	2014 Encuesta Nacional de la Dinamica Demografica (ENADID) [Others Direct]
	12	2017.2	2018 Encuesta Nacional de la Dinamica Demografica (ENADID) [Others Direct]
	96	2020.5	WHO Vital Registration (recalculated - unadjusted) [VR]
Republic of Moldova	99	2020.5	
	3	1999.4	2005 Demographic and Health Survey [DHS Direct]
	3	2006.3	2012 Multiple Indicator Cluster Survey [Others Direct]
	93	2020.5	WHO Vital Registration (recalculated - unadjusted) [VR]
Monaco			
Mongolia	99	2019.5	
	3	1992.8	1998 Reproductive Health Survey [Others Direct]
	3	2009.5	2010 Census [Others Direct]
	3	2007.8	2013-2014 Social Indicator Sample Survey (SISS) [Others Direct]
	3	2012.8	2018 Social Indicator Sample Survey (SISS) [Others Direct]
	27	1999.5	Recalculated based on WHO - adjusted for incompleteness [VR]
	60	2019.5	WHO Vital Registration (recalculated - unadjusted) [VR]
Montenegro	78	2021	
	36	2002.5	Recalculated based on WHO - adjusted for incompleteness [VR]
	42	2021	WHO Vital Registration (recalculated - unadjusted) [VR]
Montserrat	3	2004.5	
	3	2004.5	WHO Vital Registration (recalculated - unadjusted) [VR]
Morocco	57	2010	
	9	1978.3	1980 World Fertility Survey [Other DHS Direct]
	12	1985.8	1987 Demographic and Health Survey [DHS Direct]
	6	1989	1992 Demographic and Health Survey [DHS Direct]
	3	1989	1995 Demographic and Health Survey [DHS Direct]
	6	1994	1997 Pan Arab Project for Child Development Maternal and Child Health Survey [Others Direct]
	9	2001.7	2003-2004 Demographic and Health Survey [DHS Direct]
	9	2001	2004 Pan Arab Project for Family Health Family Health Survey [Others Direct]
	3	2010	2009-2010 National Demographic Survey with repeated passages [Others Direct]
Mozambique	48	2009.9	
	18	2002.6	2003-2004 Demographic and Health Survey [DHS Direct]
	18	2007.7	2008 Multiple Indicator Cluster Survey [Others Direct]

Continued on next page

Table 26 – continued from previous page

Country	# obs.	Most recent obs. year	Data series name [source type]
	12	2009.9	2011 Demographic and Health Survey [DHS Direct]
Myanmar	9	2013.8	
	3	2013.8	2014 Census [Others Direct]
	6	2012.9	2015-2016 Demographic and Health Survey [DHS Direct]
Namibia	33	2015.5	
	9	1990.5	1992 Demographic and Health Survey [DHS Direct]
	3	1994.7	2000 Demographic and Health Survey [DHS Direct]
	3	2001	2001 Census [Others Direct]
	6	2003.8	2006-2007 Demographic and Health Survey [DHS Direct]
	3	2011.2	2011 Census [Others Direct]
	6	2010.3	2013 Demographic and Health Survey [DHS Direct]
	3	2015.5	2016 Inter-censal Demographic Survey [Others Direct]
Nauru			
Nepal	91	2013.4	
	18	1990.6	1991 Fertility and Family Planning Survey [Others Direct]
	18	1995	1996 Demographic and Health Survey [DHS Direct]
	3	2001	2001 Census [Others Direct]
	17	2000	2001 Demographic and Health Survey [DHS Direct]
	11	2004.6	2006 Demographic and Health Survey [DHS Direct]
	3	2010.5	2011 Census [Others Direct]
	6	2008	2011 Demographic and Health Survey [DHS Direct]
	6	2011.2	2014 Multiple Indicator Cluster Survey [Others Direct]
	6	2013.4	2016 Demographic and Health Survey [DHS Direct]
	3	2013.3	2019 Multiple Indicator Cluster Survey [Others Direct]
Netherlands	156	2021.5	
	156	2021.5	WHO/Human Mortality Database Vital Registration (recalculated - unadjusted) [VR]
New Zealand	156	2021.5	
	3	2005.7	2006 Census [Others Direct]
	153	2021.5	WHO/Human Mortality Database Vital Registration (recalculated - unadjusted) [VR]
Nicaragua	24	2005.6	
	9	1995.9	1998 Demographic and Health Survey [DHS Direct]
	9	1999.7	2001 Demographic and Health Survey [DHS Direct]
	3	2000.8	2006 Encuesta NicaragÃense de Demografâ a y Salud [Others Direct]
	3	2005.6	2012 Encuesta Nicaraguense de Demografia y Salud [Others Direct]
Niger	93	2020.3	
	18	1991.2	1992 Demographic and Health Survey [DHS Direct]
	18	1997.2	1998 Demographic and Health Survey [DHS Direct]
	3	2000.9	2001 Census [Others Direct]
	18	2005	2006 Demographic and Health Survey [DHS Direct]
	18	2011.1	2012 Demographic and Health Survey [DHS Direct]
	18	2020.3	2021 Enquete Nationale sur la Fecondite et la Mortalite des Enfants de moins de 5 ans (ENAFEME) [Others Direct]
Nigeria	100	2017.6	
	3	1965.6	1965-1966 Rural Demographic Sample Survey [Others Direct]

Continued on next page

Table 26 – continued from previous page

Country	# obs.	Most recent obs. year	Data series name [source type]
	16	1989.2	1990 Demographic and Health Survey [DHS Direct]
	12	2001.7	2003 Demographic and Health Survey [DHS Direct]
	3	2008.1	2008 Demographic and Health Survey [DHS Direct]
	18	2007.4	2008 Demographic and Health Survey [DHS Direct]
	9	2008.7	2010 Malaria Indicator Survey [Other DHS Direct]
	3	2012.8	2013 Demographic and Health Survey [DHS Direct]
	18	2012.1	2013 Demographic and Health Survey [DHS Direct]
	18	2017.6	2018 Demographic and Health Survey [DHS Direct]
Niue	3	1995	
	3	1995	WHO Vital Registration (recalculated - unadjusted) [VR]
Norway	156	2021.5	
	156	2021.5	WHO/Human Mortality Database Vital Registration (recalculated - unadjusted) [VR]
Oman			
Pakistan	90	2014.8	
	3	1964	1962-1965 Population Growth Estimation Experiment [Others Direct]
	3	1970	1971 Population Growth Survey I [Others Direct]
	6	1987.9	1990-1991 Demographic and Health Survey [DHS Direct]
	9	2002.2	2006-2007 Demographic and Health Survey [DHS Direct]
	9	2010.7	2012-2013 Demographic and Health Survey [DHS Direct]
	6	2014.8	2017-2018 Demographic and Health Survey [DHS Direct]
	54	2007.5	Pakistan Demographic Survey [VR]
Palau	24	2004	
	24	2004	WHO/UNPD Vital Registration (recalculated - unadjusted) [VR]
Panama	96	2021.5	
	96	2021.5	Recalculated based on WHO - adjusted for incompleteness [VR]
Papua New Guinea	6	2013.7	
	6	2013.7	2016-2018 Demographic and Health Survey [DHS Direct]
Paraguay	23	2010.5	
	6	1976.1	1979 World Fertility Survey [Other DHS Direct]
	3	1984.3	1990 Demographic and Health Survey [DHS Direct]
	3	1989.8	1996 Encuesta Nacional de Demografia y Salud Sexual y Reproductiva [Others Direct]
	3	2002.2	2002 Census [Others Direct]
	3	2002.5	2008 Encuesta Nacional de Demografia y Salud Sexual y Reproductiva [Others Direct]
	3	2010.5	2016 Multiple Indicator Cluster Survey [Others Direct]
Peru	111	2015.1	
	6	1983.7	1986 Demographic and Health Survey [DHS Direct]
	12	1990.2	1991-1992 Demographic and Health Survey [DHS Direct]
	18	1995.6	1996 Demographic and Health Survey [DHS Direct]
	18	1999.5	2000 Demographic and Health Survey [DHS Direct]
	12	2005.5	2004-2008 Demographic and Health Survey [DHS Direct]
	9	2007.2	2009 Demographic and Health Survey [DHS Direct]
	6	2007.2	2010 Demographic and Health Survey [DHS Direct]
	6	2008.2	2011 Demographic and Health Survey [DHS Direct]

Continued on next page

Table 26 – continued from previous page

Country	# obs.	Most recent obs. year	Data series name [source type]
	6	2009.2	2012 Demographic and Health Survey [DHS Direct]
	3	2007.2	2013 Demographic and Health Survey [DHS Direct]
	6	2012.2	2014 Demographic and Health Survey [DHS Direct]
	3	2011.2	2017 National Demographic and Health Survey [Other DHS Direct]
	3	2012.2	2018 National Demographic and Health Survey [Other DHS Direct]
	3	2015.1	2021 National Demographic and Health Survey [Other DHS Direct]
Philippines	159	2019.5	
	17	1977.1	1978 World Fertility Survey [Other DHS Direct]
	17	1992.2	1993 Demographic and Health Survey [DHS Direct]
	14	1997.1	1998 Demographic and Health Survey [DHS Direct]
	12	2001.9	2003 Demographic and Health Survey [DHS Direct]
	6	2005.6	2008 Demographic and Health Survey [DHS Direct]
	9	2011.6	2013 Demographic and Health Survey [DHS Direct]
	6	2014.6	2017 Demographic and Health Survey [DHS Direct]
	57	2019.5	Recalculated based on WHO/UNPD - adjusted for incompleteness [VR]
	21	1999.5	WHO Vital Registration (recalculated - unadjusted) [VR]
Poland	156	2021.5	
	156	2021.5	WHO/Human Mortality Database Vital Registration (recalculated - unadjusted) [VR]
Portugal	159	2021.5	
	3	2010.8	2011 Census [Others Direct]
	156	2021.5	WHO/Human Mortality Database Vital Registration (recalculated - unadjusted) [VR]
Qatar	89	2020.5	
	89	2020.5	WHO Vital Registration (recalculated - unadjusted) [VR]
Romania	99	2021.5	
	99	2021.5	WHO Vital Registration (recalculated - unadjusted) [VR]
Russian Federation	150	2020.5	
	150	2020.5	WHO/Human Mortality Database Vital Registration (recalculated - unadjusted) [VR]
Rwanda	134	2018.3	
	18	1982.6	1983 World Fertility Survey [Other DHS Direct]
	17	1991.4	1992 Demographic and Health Survey [DHS Direct]
	15	1999.4	2000 Demographic and Health Survey [DHS Direct]
	15	2004.1	2005 Demographic and Health Survey [DHS Direct]
	18	2006.9	2008 Demographic and Health Survey [DHS Direct]
	18	2009.7	2010 Demographic and Health Survey [DHS Direct]
	3	2012.1	2012 Census [Others Direct]
	18	2013.8	2014-2015 Demographic and Health Survey [DHS Direct]
	12	2018.3	2019-2020 Demographic and Health Survey [DHS Direct]
Saint Kitts and Nevis	23	2015.5	
	23	2015.5	WHO Vital Registration (recalculated - unadjusted) [VR]
Saint Lucia	74	2018.5	

Continued on next page

Table 26 – continued from previous page

Country	# obs.	Most recent obs. year	Data series name [source type]
	74	2018.5	WHO Vital Registration (recalculated - unadjusted) [VR]
Samoa	15	2016.4	
	3	1999	1999 Demographic and Health Survey [Other DHS Direct]
	3	2006.4	2006 Census [Others Direct]
	3	2011.4	2011 Population and Housing Census [Others Direct]
	2	2016.4	2016 Population and Housing Census [Others Direct]
San Marino	3	1991	
	3	1991	WHO Vital Registration (recalculated - unadjusted) [VR]
Sao Tome and Principe	11	2013.6	
	3	2002.7	2008-2009 Demographic and Health Survey [DHS Direct]
	3	2011.9	2012 Census [Others Direct]
	3	2013.6	2019 Multiple Indicator Cluster Survey [Others Direct]
Saudi Arabia			
Senegal	218	2017.3	
	18	1977.4	1978 World Fertility Survey [Other DHS Direct]
	18	1985.2	1986 Demographic and Health Survey [DHS Direct]
	18	1991.8	1992-1993 Demographic and Health Survey [DHS Direct]
	17	1996	1997 Demographic and Health Survey [DHS Direct]
	18	1998.8	1999-2000 Demographic and Health Survey [DHS Direct]
	3	2002.5	2002 Census [Others Direct]
	18	2004.1	2005 Demographic and Health Survey [DHS Direct]
	18	2007.9	2008-2009 Malaria Indicator Survey [Other DHS Direct]
	18	2009.7	2010-2011 Demographic and Health Survey [DHS Direct]
	12	2011.2	2012-2013 Demographic and Health Survey [DHS Direct]
	3	2013.4	2013 Census [Others Direct]
	6	2011	2014 Demographic and Health Survey [DHS Direct]
	6	2012	2015 Demographic and Health Survey [DHS Direct]
	9	2014	2016 Demographic and Health Survey [DHS Direct]
	18	2016.2	2017 Demographic and Health Survey [DHS Direct]
	9	2016.4	2018 Demographic and Health Survey [DHS Direct]
	9	2017.3	2019 Demographic and Health Survey [DHS Direct]
Serbia	99	2021.5	
	60	2021.5	Recalculated based on WHO - adjusted for incompleteness [VR]
	39	2001.5	WHO Vital Registration (recalculated - unadjusted) [VR]
Seychelles	53	2016.5	
	3	1959.8	1960 Census [Others Direct]
	50	2016.5	WHO Vital Registration (recalculated - unadjusted) [VR]
Sierra Leone	66	2018.3	
	12	2006.7	2008 Demographic and Health Survey [DHS Direct]
	18	2012.4	2013 Demographic and Health Survey [DHS Direct]
	18	2016.4	2017 Multiple Indicator Cluster Survey [Others Direct]
	18	2018.3	2019 Demographic and Health Survey [DHS Direct]
Singapore	96	2020.5	
	96	2020.5	WHO Vital Registration (recalculated - unadjusted) [VR]
Slovakia	156	2021.5	

Continued on next page

Table 26 – continued from previous page

Country	# obs.	Most recent obs. year	Data series name [source type]
	156	2021.5	WHO/Human Mortality Database Vital Registration (recalculated - unadjusted) [VR]
Slovenia	117	2021.5	
	117	2021.5	WHO/Human Mortality Database Vital Registration (recalculated - unadjusted) [VR]
Solomon Islands	6	2009.4	
	3	2009.4	2009 Census [Others Direct]
	3	2008	2015 Demographic and Health Survey [Other DHS Direct]
Somalia	12	2005.2	
	12	2005.2	2006 Multiple Indicator Cluster Survey [Others Direct]
South Africa	75	2020.5	
	6	1995	1998 Demographic and Health Survey [DHS Direct]
	3	2011.3	2011 Census [Others Direct]
	3	2010.4	2016 Demographic and Health Survey [DHS Direct]
	63	2020.5	Rapid Mortality Surveillance [VR]
South Sudan	6	2007.2	
	6	2007.2	2010 Multiple Indicator Cluster Survey [Others Direct]
Spain	153	2020.5	
	153	2020.5	WHO/Human Mortality Database Vital Registration (recalculated - unadjusted) [VR]
Sri Lanka	93	2014.5	
	6	1984	1987 Demographic and Health Survey [DHS Direct]
	6	1990.6	1993 Demographic and Health Survey [Other DHS Direct]
	3	1994.4	2000 Demographic and Health Survey [Other DHS Direct]
	6	2003.7	2007 Demographic and Health Survey [Other DHS Direct]
	3	2010.4	2016 Demographic and Health Survey [Other DHS Direct]
	69	2014.5	WHO Vital Registration (recalculated - unadjusted) [VR]
Saint Vincent and the Grenadines	64	2017	
	64	2017	WHO Vital Registration (recalculated - unadjusted) [VR]
State of Palestine	27	2013.9	
	9	1993.9	1995 Demographic Survey [Others Direct]
	6	2003	2006 Pan Arab Project for Family Health Family Health Survey [Others Direct]
	6	2007.4	2010 Multiple Indicator Cluster Survey [Others Direct]
	3	2008.2	2014 Multiple Indicator Cluster Survey [Others Direct]
	3	2013.9	2019-2020 Multiple Indicator Cluster Survey [Others Direct]
Sudan	63	2013.2	
	9	1976	1979 World Fertility Survey [Other DHS Direct]
	18	1988.8	1989-1990 Demographic and Health Survey [DHS Direct]
	18	1991	1993 Pan Arab Project for Child Development Maternal and Child Health Survey [Others Direct]
	6	2007.2	2010 Multiple Indicator Cluster Survey [Others Direct]
	12	2013.2	2014 Multiple Indicator Cluster Survey [Others Direct]
Suriname	96	2019.5	
	3	2012.2	2018 Multiple Indicator Cluster Survey [Others Direct]

Continued on next page

Table 26 – continued from previous page

Country	# obs.	Most recent obs. year	Data series name [source type]
	93	2019.5	Recalculated based on WHO/UNPD - adjusted for incompleteness [VR]
Eswatini	21	2013.1	
	3	1996.9	1997 Census [Others Direct]
	3	2000.5	2006-2007 Demographic and Health Survey [DHS Direct]
	6	2007.7	2010 Multiple Indicator Cluster Survey [Others Direct]
	9	2013.1	2014 Multiple Indicator Cluster Survey [Others Direct]
Sweden	156	2021.5	
	156	2021.5	WHO/Human Mortality Database Vital Registration (recalculated - unadjusted) [VR]
Switzerland	156	2021.5	
	156	2021.5	WHO/Human Mortality Database Vital Registration (recalculated - unadjusted) [VR]
Syria	51	2010.5	
	6	1975.5	1978 World Fertility Survey [Other DHS Direct]
	3	1987	1993 Pan Arab Project for Child Development Maternal and Child Health Survey [Others Direct]
	3	1995.8	2001 Pan Arab Project for Family Health Family Health Survey [Others Direct]
	39	2010.5	WHO Vital Registration (recalculated - unadjusted) [VR]
Tajikistan	75	2021.5	
	3	2006.5	2012 Demographic and Health Survey [DHS Direct]
	3	2011.6	2017 Demographic and Health Survey [DHS Direct]
	27	1999.5	Recalculated based on WHO/UNPD - adjusted for incompleteness [VR]
	42	2021.5	WHO Vital Registration (recalculated - unadjusted) [VR]
Tanzania	101	2014.1	
	3	1973.3	1973 National Demographic Survey [Others Direct]
	18	1990.7	1991-1992 Demographic and Health Survey [DHS Direct]
	17	1995.5	1996 Demographic and Health Survey [DHS Direct]
	3	1993.7	1999 Demographic and Health Survey [DHS Direct]
	18	2003.7	2004-2005 Demographic and Health Survey [DHS Direct]
	12	2006.2	2007-2008 AIDS Indicator Survey [Other DHS Direct]
	12	2008.4	2010 Demographic and Health Survey [DHS Direct]
	3	2010.4	2010-2011 National Panel Survey [Others Direct]
	3	2012.2	2012-2013 National Panel Survey [Others Direct]
	12	2014.1	2015-2016 Demographic and Health Survey [DHS Direct]
Thailand	98	2019.5	
	3	1981.2	1987 Demographic and Health Survey [DHS Direct]
	3	1989	1989 Survey of Population Change [Others Direct]
	3	1995.5	1995 Survey of Population Change [Others Direct]
	87	2019.5	Recalculated based on WHO - adjusted for incompleteness [VR]
Timor Leste	15	2008.4	
	12	2008.1	2009-2010 Demographic and Health Survey [DHS Direct]
	3	2008.4	2015 Census [Others Direct]
Togo	66	2016.3	

Continued on next page

Table 26 – continued from previous page

Country	# obs.	Most recent obs. year	Data series name [source type]
	3	1961.4	1961 Demographic survey [Others Direct]
	12	1986.9	1988 Demographic and Health Survey [DHS Direct]
	18	1997.1	1998 Demographic and Health Survey [DHS Direct]
	3	2010.4	2010 Census [Others Direct]
	18	2012.8	2013-2014 Demographic and Health Survey [DHS Direct]
	12	2016.3	2017 Multiple Indicator Cluster Survey [Others Direct]
Tonga	75	2018.5	
	3	2006.4	2006 Census [Others Direct]
	41	2005.5	Recalculated based on WHO - adjusted for incompleteness [VR]
	29	2018.5	WHO Vital Registration (recalculated - unadjusted) [VR]
Trinidad and Tobago	92	2017.5	
	3	1971.2	1977 World Fertility Survey [Other DHS Direct]
	87	2017.5	WHO/UNPD Vital Registration (recalculated - unadjusted) [VR]
Tunisia	51	2017.5	
	3	1969	1968-1969 National Demographic Survey [Others Direct]
	6	1985.4	1988 Demographic and Health Survey [DHS Direct]
	3	1988	1994 Pan Arab Project for Child Development Maternal and Child Health Survey [Others Direct]
	3	1995	2001 Pan Arab Project for Family Health Family Health Survey [Others Direct]
	3	2006	2011-2012 Multiple Indicator Cluster Survey [Others Direct]
	3	2012.2	2018 Multiple Indicator Cluster Survey [Others Direct]
	21	2000.5	Recalculated based on WHO - adjusted for incompleteness [VR]
	9	2017.5	WHO Vital Registration (recalculated - unadjusted) [VR]
Turkey	59	2019.5	
	8	1976.7	1978 World Fertility Survey [Other DHS Direct]
	3	1987.6	1993 Demographic and Health Survey [DHS Direct]
	3	1992.6	1998 Demographic and Health Survey [DHS Direct]
	3	1997.9	2003-2004 Demographic and Health Survey [Other DHS Direct]
	3	2002.7	2003 National Verbal Autopsy Survey [Others Direct]
	3	2002.7	2008 Turkey Demographic and Health Survey [Other DHS Direct]
	3	2007.7	2013 NDemographic and Health Survey [Other DHS Direct]
	3	2012.8	2018 Demographic and Health Survey [Other DHS Direct]
	30	2019.5	WHO Vital Registration (recalculated - unadjusted) [VR]
Turkmenistan	88	2015.5	
	3	1994.5	2000 Demographic and Health Survey [DHS Direct]
	81	2015.5	Recalculated based on WHO/UNPD - adjusted for incompleteness [VR]
Turks and Caicos Islands	16	2013.8	
	14	2006.5	WHO Vital Registration (recalculated - unadjusted) [VR]
Tuvalu	11	2013.9	
	9	2002	WHO Vital Registration (recalculated - unadjusted) [VR]
Uganda	95	2015.4	
	12	1987.2	1988-1989 Demographic and Health Survey [DHS Direct]

Continued on next page

Table 26 – continued from previous page

Country	# obs.	Most recent obs. year	Data series name [source type]
	12	1993.7	1995 Demographic and Health Survey [DHS Direct]
	12	1999.2	2000-2001 Demographic and Health Survey [DHS Direct]
	3	2006	2006 Demographic and Health Survey [DHS Direct]
	18	2005.3	2006 Demographic and Health Survey [DHS Direct]
	3	2003.8	2009-2010 Demographic and Health Survey [DHS Direct]
	17	2010.4	2011 Demographic and Health Survey [DHS Direct]
	18	2015.4	2016 Demographic and Health Survey [DHS Direct]
Ukraine	160	2021.5	
	3	1993.5	1999 Reproductive Health Survey [Others Direct]
	2	2001.5	2007 Demographic and Health Survey [DHS Direct]
	2	2006.8	2012 Multiple Indicator Cluster Survey [Others Direct]
	153	2021.5	WHO/Human Mortality Database Vital Registration (recalculated - unadjusted) [VR]
United Arab Emirates			
United Kingdom	201	2020.5	
	201	2020.5	WHO Vital Registration (recalculated - unadjusted) [VR]
United States of America	153	2020.5	
	153	2020.5	WHO/Human Mortality Database Vital Registration (recalculated - unadjusted) [VR]
Uruguay	93	2020.5	
	93	2020.5	WHO Vital Registration (recalculated - unadjusted) [VR]
Uzbekistan	102	2021.5	
	3	1996	2002 Demographic and Health Survey [Other DHS Direct]
	99	2021.5	Recalculated based on WHO - adjusted for incompleteness [VR]
Vanuatu	3	2009.4	
	3	2009.4	2009 Census [Others Direct]
Venezuela (Bolivarian Republic of)	81	2016.5	
	3	1971.2	1977 World Fertility Survey [Other DHS Direct]
	45	2016.5	Recalculated based on WHO/UNPD - adjusted for incompleteness [VR]
	33	2000.5	WHO Vital Registration (recalculated - unadjusted) [VR]
Vietnam	54	2020.3	
	3	1978.8	1979 Census [Others Direct]
	9	1986	1988 National Demographic and Health Survey [Other DHS Direct]
	3	1991.5	1997 Demographic and Health Survey [DHS Direct]
	3	1998.7	1999 Census [Others Direct]
	3	1996.7	2002 Demographic and Health Survey [DHS Direct]
	3	2006.8	2007 Population Change and Family Planning Survey [Others Direct]
	3	2010.8	2011 Population Change and Family Planning Survey [Others Direct]
	3	2011.8	2012 Population Change and Family Planning Survey [Others Direct]
	3	2008	2013-2014 Multiple Indicator Cluster Survey [Others Direct]
	3	2012.8	2013 Population Change and Family Planning Survey [Others Direct]

Continued on next page

Table 26 – continued from previous page

Country	# obs.	Most recent obs. year	Data series name [source type]
	3	2013.5	2014 Intercensal Demographic Survey [Others Direct]
	3	2014.8	2015 Population Change and Family Planning Survey [Others Direct]
	3	2016.5	2017 Population Change and Family Planning Survey [Others Direct]
	3	2017.5	2018 Population Change and Family Planning Survey [Others Direct]
	3	2020.3	2020 Population Change and Family Planning Survey [Others Direct]
	3	2014.9	2021 Multiple Indicator Cluster Survey [Others Direct]
Yemen	60	2007.7	
	6	1976.7	1979 World Fertility Survey [Other DHS Direct]
	9	1989.8	1991-1992 Demographic and Health Survey [DHS Direct]
	18	1996.7	1997 Demographic and Health Survey [DHS Direct]
	18	2002	2003 Pan Arab Project for Family Health Family Health Survey [Others Direct]
	3	2000.7	2006 Multiple Indicator Cluster Survey [Others Direct]
	6	2007.7	2013 Demographic and Health Survey [DHS Direct]
Zambia	95	2017	
	18	1991	1992 Demographic and Health Survey [DHS Direct]
	17	1995.5	1996-1997 Demographic and Health Survey [DHS Direct]
	18	2000.8	2001-2002 Demographic and Health Survey [DHS Direct]
	12	2005.7	2007 Demographic and Health Survey [DHS Direct]
	18	2012.6	2013-2014 Demographic and Health Survey [DHS Direct]
	12	2017	2018 Demographic and Health Survey [DHS Direct]
Zimbabwe	62	2016.5	
	6	1985.7	1988-1989 Demographic and Health Survey [DHS Direct]
	3	1992.1	1992 Census [Others Direct]
	6	1991.5	1994 Demographic and Health Survey [DHS Direct]
	3	1993.7	1999 Demographic and Health Survey [DHS Direct]
	6	2006.3	2009 Multiple Indicator Cluster Survey [Others Direct]
	8	2008.7	2010-2011 Demographic and Health Survey [DHS Direct]
	3	2012.1	2012 Census [Others Direct]
	12	2012.7	2014 Multiple Indicator Cluster Survey [Others Direct]
	6	2012.5	2015 Demographic and Health Survey [DHS Direct]
	3	2016.5	2017 Inter-Censal Demographic Survey [Others Direct]
	6	2016.1	2019 Multiple Indicator Cluster Survey [Others Direct]

Table 27: **Sex ratio data sources for age group 15–19, by country.** For each country, the total number of observations and the most recent reference year are shown after the country name. For each country-specific data series, the number of observations and the most recent reference year within that series is shown before each data series name. The source type that each data series falls in is shown in parentheses after each data series name.

Country	# obs.	Most recent obs. year	Data series name [source type]
Afghanistan	39	2014.4	
	3	1973	1972 National Demographic and Family Guidance Survey [Others Direct]
	3	1979	1979 Census [Others Direct]
	3	2008.1	2010 Afghanistan Mortality Survey (AMS) Excluding South Zone (Household data) [Others Direct]
	12	2008.7	2010 Afghanistan Mortality Survey (AMS) [DHS Direct]
	18	2014.4	2015 Demographic and Health Survey [DHS Direct]
Albania	69	2021.5	
	3	2011.1	2011 Census [Others Direct]
	66	2021.5	WHO Vital Registration (recalculated - unadjusted) [VR]
Algeria	69	2020.5	
	3	1970.5	1970 Demographic Survey [Others Direct]
	66	2020.5	WHO Vital Registration (recalculated - unadjusted) [VR]
Andorra	10	2019.5	
	10	2019.5	WHO Vital Registration (recalculated - unadjusted) [VR]
Angola	21	2014.8	
	3	2013.9	2014 Census [Others Direct]
	18	2014.8	2016 Inquerito de Indicadores Multiplos e de Saude [DHS Direct]
Anguilla			
Antigua and Barbuda	40	2017.5	
	40	2017.5	WHO Vital Registration (recalculated - unadjusted) [VR]
Argentina	96	2020.5	
	96	2020.5	WHO Vital Registration (recalculated - unadjusted) [VR]
Armenia	95	2021.5	
	95	2021.5	WHO Vital Registration (recalculated - unadjusted) [VR]
Australia	153	2020.5	
	153	2020.5	WHO/Human Mortality Database Vital Registration (recalculated - unadjusted) [VR]
Austria	153	2020.5	
	153	2020.5	WHO/Human Mortality Database Vital Registration (recalculated - unadjusted) [VR]
Azerbaijan	90	2021.5	
	90	2021.5	Recalculated based on WHO - adjusted for incompleteness [VR]
Bahamas	93	2020.5	
	93	2020.5	Recalculated based on WHO/UNPD - adjusted for incompleteness [VR]
Bahrain	92	2019.5	
	92	2019.5	WHO/UNPD Vital Registration (recalculated - unadjusted) [VR]
Bangladesh	123	2020.5	
	3	1964	1962-1965 Population Growth Estimation Experiment [Others Direct]

Continued on next page

Table 27 – continued from previous page

Country	# obs.	Most recent obs. year	Data series name [source type]
	3	1974.5	1974 Retrospective Fertility and Mortality Survey (UN SA) [Others Direct]
	3	1999.8	1999-2000 Demographic and Health Survey [Others Direct]
	12	2001.7	2003 World Health Survey [Others Direct]
	3	2010	2010 Maternal Health Services and Maternal Mortality Survey [Others Direct]
	3	2010.7	2011 Census [Others Direct]
	3	2016	2016 Maternal Health Services and Maternal Mortality Survey [Others Direct]
	3	2016.5	Report on Sample Vital Registration System 2016 [Others Direct]
	3	2017.5	Report on Sample Vital Registration System 2016 [Others Direct]
	3	2018.5	Report on Sample Vital Registration System 2016 [Others Direct]
	3	2019.5	Report on Sample Vital Registration System 2016 [Others Direct]
	3	2020.5	Report on Sample Vital Registration System 2016 [Others Direct]
	78	2015.5	Sample Vital Statistics [VR]
Barbados	75	2013.5	
	75	2013.5	WHO Vital Registration (recalculated - unadjusted) [VR]
Belarus	150	2019.5	
	150	2019.5	WHO/Human Mortality Database Vital Registration (recalculated - unadjusted) [VR]
Belgium	153	2020.5	
	153	2020.5	WHO/Human Mortality Database Vital Registration (recalculated - unadjusted) [VR]
Belize	81	2018.5	
	81	2018.5	Recalculated based on WHO - adjusted for incompleteness [VR]
Benin	63	2016.8	
	18	1995.4	1996 Demographic and Health Survey [DHS Direct]
	18	2005.6	2006 Demographic and Health Survey [DHS Direct]
	9	2012.5	2014 Multiple Indicator Cluster Survey [Others Direct]
	18	2016.8	2018 Demographic and Health Survey [DHS Direct]
Bhutan	9	2012.5	
	3	1993.1	1994 National Health Survey [Others Direct]
	3	2004.9	2005 Census [Others Direct]
	3	2012.5	2012 National Health Survey [Others Direct]
Bolivia (Plurinational State of)	60	2007.1	
	3	1979.8	1980 National Demographic Survey [Others Direct]
	3	1991.9	1992 Census [Others Direct]
	18	1992.8	1993-1994 Demographic and Health Survey [DHS Direct]
	18	2002.6	2003 Demographic and Health Survey [DHS Direct]
	18	2007.1	2008 Demographic and Health Survey [DHS Direct]
Bosnia and Herzegovina	63	2016.5	
	63	2016.5	WHO Vital Registration (recalculated - unadjusted) [VR]
Botswana	21	2016.5	

Continued on next page

Table 27 – continued from previous page

Country	# obs.	Most recent obs. year	Data series name [source type]
	3	1981.1	1981 Census [Others Direct]
	3	1991.1	1991 Census [Others Direct]
	3	1997.9	1998 Demographic Survey [Others Direct]
	3	2001.1	2001 Census [Others Direct]
	3	2006.2	2006 Demographic Survey [Others Direct]
	3	2011.1	2011 Census [Others Direct]
	3	2016.5	2017 Demographic Survey [Others Direct]
Brazil	117	2020.5	
	18	1995.2	1996 Demographic and Health Survey [DHS Direct]
	3	2010.2	2010 Census [Others Direct]
	96	2020.5	WHO Vital Registration (recalculated - unadjusted) [VR]
British Virgin Islands			
Brunei	83	2019.5	
	83	2019.5	WHO Vital Registration (recalculated - unadjusted) [VR]
Bulgaria	156	2021.5	
	156	2021.5	WHO/Human Mortality Database Vital Registration (recalculated - unadjusted) [VR]
Burkina Faso	81	2009.3	
	3	1960.5	1960-1961 Survey [Others Direct]
	3	1985.5	1985 Census [Others Direct]
	3	1995.5	1996 Census [Others Direct]
	18	1997.8	1998-1999 Demographic and Health Survey [DHS Direct]
	18	2002.4	2003 Demographic and Health Survey [DHS Direct]
	12	2001.7	2003 World Health Survey [Others Direct]
	3	2006.4	2006 Census [Others Direct]
	3	2007.3	2008 Global Fund Evaluation Survey [Others Direct]
	18	2009.3	2010 Demographic and Health Survey [DHS Direct]
Burundi	42	2015.8	
	3	1964.7	1965 Population Survey [Others Direct]
	3	2008.2	2008 Census [Others Direct]
	18	2009.6	2010 Demographic and Health Survey [DHS Direct]
	18	2015.8	2017 Demographic and Health Survey [DHS Direct]
Cambodia	72	2013.4	
	18	1999.1	2000 Demographic and Health Survey [DHS Direct]
	18	2004.7	2005 Demographic and Health Survey [DHS Direct]
	18	2009.5	2010 Demographic and Health Survey [DHS Direct]
	18	2013.4	2014 Demographic and Health Survey [DHS Direct]
Cameroon	81	2017.4	
	3	1975.8	1976 Census [Others Direct]
	3	1986.8	1987 Census [Others Direct]
	18	1997.1	1998 Demographic and Health Survey [DHS Direct]
	18	2003.1	2004 Demographic and Health Survey [DHS Direct]
	3	2005.4	2005 Census [Others Direct]
	18	2010	2011 Demographic and Health Survey [DHS Direct]

Continued on next page

Table 27 – continued from previous page

Country	# obs.	Most recent obs. year	Data series name [source type]
	18	2017.4	2018 Demographic and Health Survey [DHS Direct]
Canada	153	2020.5	
	153	2020.5	WHO/Human Mortality Database Vital Registration (recalculated - unadjusted) [VR]
Cape Verde	81	2018.5	
	3	2000	2000 Census [Others Direct]
	3	2009.5	2010 Census [Others Direct]
	18	1999.5	Recalculated based on WHO/UNPD - adjusted for incompleteness [VR]
	57	2018.5	WHO/UNPD Vital Registration (recalculated - unadjusted) [VR]
Central African Republic	27	2003.4	
	3	1959.5	1959-1960 Survey [Others Direct]
	3	1988.4	1988 Census [Others Direct]
	18	1993.7	1994-1995 Demographic and Health Survey [DHS Direct]
	3	2003.4	2003 Census [Others Direct]
Chad	57	2013.7	
	18	1995.9	1996-1997 Demographic and Health Survey [DHS Direct]
	18	2003.5	2004 Demographic and Health Survey [DHS Direct]
	3	2008.9	2009 Census [Others Direct]
	18	2013.7	2014-2015 Demographic and Health Survey [DHS Direct]
Chile	153	2020.5	
	153	2020.5	WHO/Human Mortality Database Vital Registration (recalculated - unadjusted) [VR]
China	48	2010.5	
	3	1973.5	1964-1982 Adjusted Census Deaths (1964-1982) [Others Direct]
	3	1986.5	1982-1990 Adjusted Census Deaths (1982-1990) [Others Direct]
	3	1987	1987 China 1987 One-per-Hundred National Population Survey [Others Direct]
	3	1995.5	1990-2000 Adjusted Census Deaths (1990-2000) [Others Direct]
	3	1995.4	1995 China 1995 One-percent Sample Survey [Others Direct]
	3	2000.5	1999-2000 Adjusted Census Deaths (1999-2000) [Others Direct]
	3	2005.5	2000-2010 Adjusted Census Deaths (2000-2010) [Others Direct]
	3	2007.4	2007 China 2007 Annual Population Change Survey [Others Direct]
	3	2008.4	2008 China 2008 Annual Population Change Survey [Others Direct]
	21	2010.5	China CDC Surveillance surveys [VR]
Colombia	102	2020.5	
	18	2014.1	2015 Demographic and Health Survey [DHS Direct]
	84	2020.5	Recalculated based on WHO - adjusted for incompleteness [VR]
Comoros	12	2009.6	
	3	1958.2	1958 Census [Others Direct]
	3	2003.2	2003 Census [Others Direct]
	6	2009.6	2012 Demographic and Health Survey [DHS Direct]
Congo	45	2012.9	
	18	2004.5	2005 Demographic and Health Survey [DHS Direct]
	18	2010.7	2011-2012 Demographic and Health Survey [DHS Direct]

Continued on next page

Table 27 – continued from previous page

Country	# obs.	Most recent obs. year	Data series name [source type]
	9	2012.9	2015 Multiple Indicator Cluster Survey [Others Direct]
Democratic Republic of the Congo	33	2012.6	
	15	2006	2007 Demographic and Health Survey [DHS Direct]
	18	2012.6	2013-2014 Demographic and Health Survey [DHS Direct]
Cook Islands	25	2008.5	
	25	2008.5	WHO Vital Registration (recalculated - unadjusted) [VR]
Costa Rica	96	2020.5	
	96	2020.5	Recalculated based on WHO - adjusted for incompleteness [VR]
Cote d'Ivoire	60	2010.9	
	3	1977.5	1978-1979 Demographic Survey Repeated Passages [Others Direct]
	18	1993.4	1994 Demographic and Health Survey [DHS Direct]
	3	1998.4	1998 Census [Others Direct]
	6	2000.2	2003 World Health Survey [Others Direct]
	12	2004.1	2005 AIDS Indicator Survey [DHS Direct]
	18	2010.9	2011-2012 Demographic and Health Survey [DHS Direct]
Croatia	117	2021.5	
	3	2010.8	2011 Census [Others Direct]
	114	2021.5	WHO/Human Mortality Database Vital Registration (recalculated - unadjusted) [VR]
Cuba	96	2020.5	
	96	2020.5	WHO Vital Registration (recalculated - unadjusted) [VR]
Cyprus	92	2020.5	
	56	2020.5	Recalculated based on WHO - adjusted for incompleteness [VR]
	36	2000.5	WHO Vital Registration (recalculated - unadjusted) [VR]
Czech Republic	156	2021.5	
	156	2021.5	WHO/Human Mortality Database Vital Registration (recalculated - unadjusted) [VR]
Denmark	156	2021.5	
	156	2021.5	WHO/Human Mortality Database Vital Registration (recalculated - unadjusted) [VR]
Djibouti			
Dominica	37	2015	
	17	1999.5	Recalculated based on WHO - adjusted for incompleteness [VR]
	20	2015	WHO Vital Registration (recalculated - unadjusted) [VR]
Dominican Republic	48	2010.4	
	18	2001.5	2002 Demographic and Health Survey [DHS Direct]
	9	2001.2	2003 World Health Survey [Others Direct]
	18	2006.2	2007 Demographic and Health Survey [DHS Direct]
	3	2010.4	2010 Census [Others Direct]
Ecuador	96	2020.5	
	3	1997.2	2003 World Health Survey [Others Direct]
	93	2020.5	WHO Vital Registration (recalculated - unadjusted) [VR]
Egypt	93	2019.5	

Continued on next page

Table 27 – continued from previous page

Country	# obs.	Most recent obs. year	Data series name [source type]
	42	2019.5	Recalculated based on WHO - adjusted for incompleteness [VR]
	51	2005.5	WHO Vital Registration (recalculated - unadjusted) [VR]
El Salvador	87	2019.5	
	3	1992.2	1992 Census [Others Direct]
	84	2019.5	WHO/UNPD Vital Registration (recalculated - unadjusted) [VR]
Equatorial Guinea			
Eritrea			
Estonia	156	2020.5	
	3	2011.6	2011-2012 Census [Others Direct]
	153	2020.5	WHO/Human Mortality Database Vital Registration (recalculated - unadjusted) [VR]
Ethiopia	84	2015	
	3	1983.9	1984 Census [Others Direct]
	15	1997.1	2000 Demographic and Health Survey [DHS Direct]
	12	2001.7	2003 World Health Survey [Others Direct]
	18	2004.2	2005 Demographic and Health Survey [DHS Direct]
	3	2006.9	2007 Census [Others Direct]
	15	2009.9	2011 Demographic and Health Survey [DHS Direct]
	18	2015	2016 Demographic and Health Survey [DHS Direct]
Federated States of Micronesia			
Fiji	42	2019.5	
	42	2019.5	Recalculated based on WHO - adjusted for incompleteness [VR]
Finland	156	2021.5	
	156	2021.5	WHO/Human Mortality Database Vital Registration (recalculated - unadjusted) [VR]
France	153	2020.5	
	153	2020.5	WHO/Human Mortality Database Vital Registration (recalculated - unadjusted) [VR]
Gabon	24	2010.5	
	12	1999	2000 Demographic and Health Survey [DHS Direct]
	12	2010.5	2012 Demographic and Health Survey [DHS Direct]
The Gambia	33	2018.8	
	3	2012.8	2013 Census [Others Direct]
	12	2011.6	2013 Demographic and Health Survey [DHS Direct]
	18	2018.8	2020 Demographic and Health Survey [DHS Direct]
Georgia	90	2021.5	
	33	2001.5	Recalculated based on WHO - adjusted for incompleteness [VR]
	57	2021.5	WHO Vital Registration (recalculated - unadjusted) [VR]
Germany	153	2021.5	
	153	2021.5	WHO/Human Mortality Database Vital Registration (recalculated - unadjusted) [VR]
Ghana	30	2014.2	
	12	2001.7	2003 World Health Survey [Others Direct]
	12	2005.5	2007 Maternal Health Survey [DHS Direct]
	3	2010.3	2010 Census [Others Direct]

Continued on next page

Table 27 – continued from previous page

Country	# obs.	Most recent obs. year	Data series name [source type]
	3	2014.2	2017 Demographic and Health Survey [Others Direct]
Greece	153	2020.5	
	153	2020.5	WHO/Human Mortality Database Vital Registration (recalculated - unadjusted) [VR]
Grenada	76	2018.5	
	32	2000.5	Recalculated based on WHO - adjusted for incompleteness [VR]
	44	2018.5	WHO Vital Registration (recalculated - unadjusted) [VR]
Guatemala	117	2017.5	
	12	1993.9	1995 Demographic and Health Survey [DHS Direct]
	18	2013.7	2014-2015 Demographic and Health Survey [DHS Direct]
	87	2017.5	WHO Vital Registration (recalculated - unadjusted) [VR]
Guinea	60	2013.8	
	3	1996.4	1996 Census [Others Direct]
	18	1998.3	1999 Demographic and Health Survey [DHS Direct]
	18	2004.1	2005 Demographic and Health Survey [DHS Direct]
	18	2011.4	2012 Demographic and Health Survey [DHS Direct]
	3	2013.8	2014 Census [Others Direct]
Guinea-Bissau	12	2012.2	
	3	2008.8	2009 Census [Others Direct]
	9	2012.2	2014 Multiple Indicator Cluster Survey [Others Direct]
Guyana	78	2014.5	
	78	2014.5	WHO Vital Registration (recalculated - unadjusted) [VR]
Haiti	48	2015.8	
	12	1998.6	2000 Demographic and Health Survey [DHS Direct]
	18	2004.7	2005-2006 Demographic and Health Survey [DHS Direct]
	18	2015.8	2017 Demographic and Health Survey [DHS Direct]
Honduras	3	2001.1	
	3	2001.1	2001 Census [Others Direct]
Hungary	153	2020.5	
	153	2020.5	WHO/Human Mortality Database Vital Registration (recalculated - unadjusted) [VR]
Iceland	132	2021	
	132	2021	WHO/Human Mortality Database Vital Registration (recalculated - unadjusted) [VR]
India	141	2019.5	
	3	1970.5	1971 Census [Others Direct]
	3	1980.5	1981 Census [Others Direct]
	3	1991.9	1992-1993 National Family Health Survey [Others Direct]
	3	1998.2	1998-1999 National Family Health Survey [Others Direct]
	12	2001.7	2003 World Health Survey [Others Direct]
	117	2019.5	Sample Registration System [VR]
Indonesia	63	2010.3	
	6	1964.5	1964-1965 National Socio-economic Survey [Others Direct]
	18	1993.5	1994 Demographic and Health Survey [DHS Direct]
	12	2001.2	2002-2003 Demographic and Health Survey [DHS Direct]
	15	2006.4	2007 Demographic and Health Survey [DHS Direct]

Continued on next page

Table 27 – continued from previous page

Country	# obs.	Most recent obs. year	Data series name [source type]
	3	2009.9	2010 Census [Others Direct]
	9	2010.3	2012 Demographic and Health Survey [DHS Direct]
Iran (Islamic Republic of)	42	2017.5	
	3	1975.5	1976 Census [Others Direct]
	3	1999.8	2000 Demographic and Health Survey [Others Direct]
	3	2004.5	Based on 2004 DH - adjusted for incompleteness [Others Direct]
	33	2017.5	Recalculated based on WHO - adjusted for incompleteness [VR]
Iraq	3	2004.6	
	3	2004.6	2006 Iraq Family Health Survey [Others Direct]
Ireland	156	2020.5	
	3	2010.8	2011 Census [Others Direct]
	153	2020.5	WHO/Human Mortality Database Vital Registration (recalculated - unadjusted) [VR]
Israel	141	2021.5	
	141	2021.5	WHO/Human Mortality Database Vital Registration (recalculated - unadjusted) [VR]
Italy	153	2020.5	
	153	2020.5	WHO/Human Mortality Database Vital Registration (recalculated - unadjusted) [VR]
Jamaica	3	2010.2	
	3	2010.2	2011 Census [Others Direct]
Japan	153	2021.5	
	153	2021.5	WHO/Human Mortality Database Vital Registration (recalculated - unadjusted) [VR]
Jordan	9	2014.9	
	6	1995.4	1997 Population and Family Health Survey [DHS Direct]
	3	2014.9	2015 Census [Others Direct]
Kazakhstan	102	2021.5	
	3	1997.2	2003 World Health Survey [Others Direct]
	99	2021.5	WHO Vital Registration (recalculated - unadjusted) [VR]
Kenya	84	2013.3	
	18	1997.1	1998 Demographic and Health Survey [DHS Direct]
	18	2002.2	2003 Demographic and Health Survey [DHS Direct]
	9	2001.2	2003 World Health Survey [Others Direct]
	18	2007.8	2008-2009 Demographic and Health Survey [DHS Direct]
	3	2009.2	2009 Census [Others Direct]
	18	2013.3	2014 Demographic and Health Survey [DHS Direct]
Kiribati			
Democratic People's Republic of Korea	3	2008.3	
	3	2008.3	2008 Census [Others Direct]
Republic of Korea	99	2021.5	

Continued on next page

Table 27 – continued from previous page

**Country**	**# obs.**	**Most recent obs. year**	**Data series name [source type]**
	99	2021.5	WHO/Human Mortality Database Vital Registration (recalculated - unadjusted) [VR]
Kosovo			
Kuwait	87	2019.5	
	87	2019.5	WHO Vital Registration (recalculated - unadjusted) [VR]
Kyrgyzstan	99	2021.5	
	99	2021.5	WHO Vital Registration (recalculated - unadjusted) [VR]
Lao People's Democratic Republic	15	2014.8	
	9	2001.2	2003 World Health Survey [Others Direct]
	3	2004.8	2005 Census [Others Direct]
	3	2014.8	2015 Census [Others Direct]
Latvia	156	2021.5	
	156	2021.5	WHO/Human Mortality Database Vital Registration (recalculated - unadjusted) [VR]
Lebanon			
Lesotho	63	2015.5	
	3	1995.8	1996 Census [Others Direct]
	3	2000.9	2001 Demographic Survey [Others Direct]
	18	2003.7	2004 Demographic and Health Survey [DHS Direct]
	18	2008.7	2009 Demographic and Health Survey [DHS Direct]
	18	2013.7	2014 Demographic and Health Survey [DHS Direct]
	3	2015.5	2016 Census [Others Direct]
Liberia	45	2018.2	
	3	1969.5	1969-1970 Population Growth Survey [Others Direct]
	15	2005.9	2006-2007 Demographic and Health Survey [DHS Direct]
	18	2012.2	2013 Demographic and Health Survey [DHS Direct]
	9	2018.2	2020 Demographic and Health Survey [DHS Direct]
Libya			
Lithuania	162	2021.5	
	3	2010.8	2011 Census [Others Direct]
	3	2017.5	2018 Statistical Survey [Others Direct]
	156	2021.5	WHO/Human Mortality Database Vital Registration (recalculated - unadjusted) [VR]
Luxembourg	138	2020.5	
	138	2020.5	WHO/Human Mortality Database Vital Registration (recalculated - unadjusted) [VR]
Macedonia	93	2020.5	
	93	2020.5	Recalculated based on WHO - adjusted for incompleteness [VR]
Madagascar	51	2007.8	
	3	1993.1	1993 Census [Others Direct]
	18	1996.7	1997 Demographic and Health Survey [DHS Direct]
	12	2002.3	2003-2004 Demographic and Health Survey [DHS Direct]
	18	2007.8	2008-2009 Demographic and Health Survey [DHS Direct]
Malawi	123	2018.2	

Continued on next page

Table 27 – continued from previous page

Country	# obs.	Most recent obs. year	Data series name [source type]
	3	1971.1	1970-1972 Population Change Survey [Others Direct]
	12	1991.2	1992 Demographic and Health Survey [DHS Direct]
	3	1998.2	1998 Census [Others Direct]
	18	1999.5	2000 Demographic and Health Survey [DHS Direct]
	18	2002.2	2003 World Health Survey [Others Direct]
	18	2003.8	2004 Demographic and Health Survey [DHS Direct]
	3	2008	2008 Census [Others Direct]
	18	2009.4	2010 Demographic and Health Survey [DHS Direct]
	9	2011.9	2014 Multiple Indicator Cluster Survey [Others Direct]
	18	2014.7	2015-2016 Demographic and Health Survey [DHS Direct]
	3	2018.2	2018 Census [Others Direct]
Malaysia	90	2021.5	
	90	2021.5	WHO Vital Registration (recalculated - unadjusted) [VR]
Maldives	92	2020.5	
	18	1994.5	Recalculated based on WHO - adjusted for incompleteness [VR]
	74	2020.5	WHO Vital Registration (recalculated - unadjusted) [VR]
Mali	84	2017.6	
	3	1986.8	1987 Census [Others Direct]
	18	2000	2001 Demographic and Health Survey [DHS Direct]
	9	2001.2	2003 World Health Survey [Others Direct]
	18	2005.3	2006 Demographic and Health Survey [DHS Direct]
	18	2011.8	2012-2013 Demographic and Health Survey [DHS Direct]
	18	2017.6	2018 Demographic and Health Survey [DHS Direct]
Malta	87	2021.5	
	87	2021.5	WHO Vital Registration (recalculated - unadjusted) [VR]
Marshall Islands			
Mauritania	21	2018.4	
	3	1977	1976-1977 Census [Others Direct]
	3	1987.8	1988 Census [Others Direct]
	3	2012.8	2013 Census [Others Direct]
	12	2018.4	2021 Demographic and Health Survey [DHS Direct]
Mauritius	96	2020.5	
	96	2020.5	WHO Vital Registration (recalculated - unadjusted) [VR]
Mexico	96	2020.5	
	96	2020.5	WHO Vital Registration (recalculated - unadjusted) [VR]
Republic of Moldova	93	2020.5	
	93	2020.5	WHO Vital Registration (recalculated - unadjusted) [VR]
Monaco			
Mongolia	90	2019.5	
	3	2009.5	2010 Census [Others Direct]
	27	1999.5	Recalculated based on WHO - adjusted for incompleteness [VR]
	60	2019.5	WHO Vital Registration (recalculated - unadjusted) [VR]
Montenegro	92	2021.5	
	42	2002.5	Recalculated based on WHO - adjusted for incompleteness [VR]

Continued on next page

Table 27 – continued from previous page

Country	# obs.	Most recent obs. year	Data series name [source type]
	50	2021.5	WHO Vital Registration (recalculated - unadjusted) [VR]
Montserrat			
Morocco	41	2010	
	17	1991	1992 Demographic and Health Survey [DHS Direct]
	18	2002.7	2003-2004 Demographic and Health Survey [DHS Direct]
	3	1997.2	2003 World Health Survey [Others Direct]
	3	2010	2009-2010 National Demographic Survey with repeated passages [Others Direct]
Mozambique	45	2010.4	
	9	1995.2	1997 Demographic and Health Survey [DHS Direct]
	18	2002.6	2003 Demographic and Health Survey [DHS Direct]
	18	2010.4	2011 Demographic and Health Survey [DHS Direct]
Myanmar	12	2014.4	
	3	2013.8	2014 Census [Others Direct]
	9	2014.4	2015-2016 Demographic and Health Survey [DHS Direct]
Namibia	84	2012.3	
	18	1991.5	1992 Demographic and Health Survey [DHS Direct]
	18	1999.7	2000 Demographic and Health Survey [DHS Direct]
	3	2001	2001 Census [Others Direct]
	6	2000.2	2003 World Health Survey [Others Direct]
	18	2005.8	2006-2007 Demographic and Health Survey [DHS Direct]
	3	2011.2	2011 Census [Others Direct]
	18	2012.3	2013 Demographic and Health Survey [DHS Direct]
Nauru			
Nepal	63	2014.9	
	18	1995	1996 Demographic and Health Survey [DHS Direct]
	3	2001	2001 Census [Others Direct]
	15	2000.2	2003 World Health Survey [Others Direct]
	12	2004.6	2006 Demographic and Health Survey [DHS Direct]
	3	2010.5	2011 Census [Others Direct]
	12	2014.9	2016 Demographic and Health Survey [DHS Direct]
Netherlands	156	2021.5	
	156	2021.5	WHO/Human Mortality Database Vital Registration (recalculated - unadjusted) [VR]
New Zealand	159	2021.5	
	3	2005.7	2006 Census [Others Direct]
	156	2021.5	WHO/Human Mortality Database Vital Registration (recalculated - unadjusted) [VR]
Nicaragua			
Niger	57	2011.1	
	18	1991.2	1992 Demographic and Health Survey [DHS Direct]
	3	2000.9	2001 Census [Others Direct]
	18	2005	2006 Demographic and Health Survey [DHS Direct]
	18	2011.1	2012 Demographic and Health Survey [DHS Direct]
Nigeria	63	2017.6	
	3	1965.6	1965-1966 Rural Demographic Sample Survey [Others Direct]

Continued on next page

Table 27 – continued from previous page

Country	# obs.	Most recent obs. year	Data series name [source type]
	18	2007.4	2008 Demographic and Health Survey [DHS Direct]
	3	2010.5	2010 GHS Panel Survey [Others Direct]
	3	2012.5	2012-2013 GHS Panel Survey [Others Direct]
	18	2012.1	2013 Demographic and Health Survey [DHS Direct]
	18	2017.6	2018 Demographic and Health Survey [DHS Direct]
Niue			
Norway	156	2021.5	
	156	2021.5	WHO/Human Mortality Database Vital Registration (recalculated - unadjusted) [VR]
Oman			
Pakistan	33	2007.5	
	12	1965.5	1962-1965 Population Growth Estimation Experiment [Others Direct]
	3	1970	1971 Population Growth Survey I [Others Direct]
	3	2006.4	2006-2007 Demographic and Health Survey [Others Direct]
	15	2007.5	Pakistan Demographic Survey [VR]
Palau	44	2018.5	
	44	2018.5	WHO/UNPD Vital Registration (recalculated - unadjusted) [VR]
Panama	96	2021.5	
	96	2021.5	Recalculated based on WHO - adjusted for incompleteness [VR]
Papua New Guinea	18	2015.8	
	18	2015.8	2018 Demographic and Health Survey [DHS Direct]
Paraguay	15	2002.2	
	3	2002.2	2002 Census [Others Direct]
	12	2001.7	2003 World Health Survey [Others Direct]
Peru	102	2009.7	
	18	1990.7	1991-1992 Demographic and Health Survey [DHS Direct]
	18	1995.6	1996 Demographic and Health Survey [DHS Direct]
	18	1999.5	2000 Demographic and Health Survey [DHS Direct]
	18	2002.9	2006 Demographic and Health Survey [DHS Direct]
	18	2006	2008 Demographic and Health Survey [DHS Direct]
	12	2009.7	2011 Demographic and Health Survey [DHS Direct]
Philippines	117	2019.5	
	18	1992.2	1993 National Demographic and Health Survey [DHS Direct]
	18	1997.1	1998 National Demographic and Health Survey [DHS Direct]
	60	2019.5	Recalculated based on WHO/UNPD - adjusted for incompleteness [VR]
	21	1999.5	WHO Vital Registration (recalculated - unadjusted) [VR]
Poland	156	2021.5	
	156	2021.5	WHO/Human Mortality Database Vital Registration (recalculated - unadjusted) [VR]
Portugal	159	2021.5	
	3	2010.8	2011 Census [Others Direct]
	156	2021.5	WHO/Human Mortality Database Vital Registration (recalculated - unadjusted) [VR]
Qatar	87	2020.5	
	87	2020.5	WHO Vital Registration (recalculated - unadjusted) [VR]
Romania	99	2021.5	

Continued on next page

Table 27 – continued from previous page

Country	# obs.	Most recent obs. year	Data series name [source type]
	99	2021.5	WHO Vital Registration (recalculated - unadjusted) [VR]
Russian Federation	150	2020.5	
	150	2020.5	WHO/Human Mortality Database Vital Registration (recalculated - unadjusted) [VR]
Rwanda	90	2018.8	
	15	1999.4	2000 Demographic and Health Survey [DHS Direct]
	3	2002.1	2002 Census [Others Direct]
	15	2004.1	2005 Demographic and Health Survey [DHS Direct]
	18	2009.7	2010 Demographic and Health Survey [DHS Direct]
	3	2012.1	2012 Census [Others Direct]
	18	2013.8	2014-2015 Demographic and Health Survey [DHS Direct]
	18	2018.8	2020 Demographic and Health Survey [DHS Direct]
Saint Kitts and Nevis	50	2015.5	
	50	2015.5	WHO Vital Registration (recalculated - unadjusted) [VR]
Saint Lucia	84	2018.5	
	84	2018.5	WHO Vital Registration (recalculated - unadjusted) [VR]
Samoa	12	2016.4	
	3	1999	1999 Demographic and Health Survey [Others Direct]
	3	2006.4	2006 Census [Others Direct]
	3	2011.4	2011 Population and Housing Census [Others Direct]
	3	2016.4	2016 Population and Housing Census [Others Direct]
San Marino			
Sao Tome and Principe	12	2011.9	
	6	2005.7	2008-2009 Demographic and Health Survey [DHS Direct]
	3	2011.9	2012 Census [Others Direct]
	3	2008.3	2014 Multiple Indicator Cluster Survey [Others Direct]
Saudi Arabia	14	2017.5	
	3	1998.6	1999 Demographic Survey [Others Direct]
	3	2004.2	2004 Census [Others Direct]
	3	2009.8	2010 Census [Others Direct]
	3	2017.5	2018 Household Health Survey [Others Direct]
Senegal	78	2016.2	
	18	1991.8	1992-1993 Demographic and Health Survey [DHS Direct]
	3	2002.5	2002 Census [Others Direct]
	18	2004.1	2005 Demographic and Health Survey [DHS Direct]
	18	2009.7	2010-2011 Demographic and Health Survey [DHS Direct]
	3	2013.4	2013 Census [Others Direct]
	18	2016.2	2017 Demographic and Health Survey [DHS Direct]
Serbia	99	2021.5	
	60	2021.5	Recalculated based on WHO - adjusted for incompleteness [VR]
	39	2001.5	WHO Vital Registration (recalculated - unadjusted) [VR]
Seychelles	66	2016.5	
	3	1959.8	1960 Census [Others Direct]

Continued on next page

Table 27 – continued from previous page

Country	# obs.	Most recent obs. year	Data series name [source type]
	63	2016.5	WHO Vital Registration (recalculated - unadjusted) [VR]
Sierra Leone	57	2018.3	
	3	2004.4	2004 Census [Others Direct]
	18	2007.2	2008 Demographic and Health Survey [DHS Direct]
	18	2012.4	2013 Demographic and Health Survey [DHS Direct]
	18	2018.3	2019 Demographic and Health Survey [DHS Direct]
Singapore	96	2020.5	
	96	2020.5	WHO Vital Registration (recalculated - unadjusted) [VR]
Slovakia	156	2021.5	
	156	2021.5	WHO/Human Mortality Database Vital Registration (recalculated - unadjusted) [VR]
Slovenia	120	2021.5	
	120	2021.5	WHO/Human Mortality Database Vital Registration (recalculated - unadjusted) [VR]
Solomon Islands	3	2009.4	
	3	2009.4	2009 Census [Others Direct]
Somalia			
South Africa	99	2020.5	
	3	1997	1997 Household Survey [Others Direct]
	18	1997	1998 Demographic and Health Survey [DHS Direct]
	3	1997.2	2003 World Health Survey [Others Direct]
	3	2011.3	2011 Census [Others Direct]
	9	2014.4	2016 Demographic and Health Survey [DHS Direct]
	63	2020.5	Rapid Mortality Surveillance [VR]
South Sudan			
Spain	153	2020.5	
	153	2020.5	WHO/Human Mortality Database Vital Registration (recalculated - unadjusted) [VR]
Sri Lanka	75	2014.5	
	3	1997.2	2003 World Health Survey [Others Direct]
	72	2014.5	WHO Vital Registration (recalculated - unadjusted) [VR]
Saint Vincent and the Grenadines	84	2017.5	
	84	2017.5	WHO Vital Registration (recalculated - unadjusted) [VR]
State of Palestine			
Sudan	3	1992.8	
	3	1992.8	1993 Census [Others Direct]
Suriname	93	2019.5	
	93	2019.5	Recalculated based on WHO/UNPD - adjusted for incompleteness [VR]
Eswatini	27	2006.8	
	3	1996.9	1997 Census [Others Direct]
	3	1997.2	2003 World Health Survey [Others Direct]
	18	2005.5	2006-2007 Demographic and Health Survey [DHS Direct]
	3	2006.8	2007 Census [Others Direct]
Sweden	156	2021.5	

Continued on next page

Table 27 – continued from previous page

Country	# obs.	Most recent obs. year	Data series name [source type]
	156	2021.5	WHO/Human Mortality Database Vital Registration (recalculated - unadjusted) [VR]
Switzerland	156	2021.5	
	156	2021.5	WHO/Human Mortality Database Vital Registration (recalculated - unadjusted) [VR]
Syria	39	2010.5	
	39	2010.5	WHO Vital Registration (recalculated - unadjusted) [VR]
Tajikistan	63	2021.5	
	33	1999.5	Recalculated based on WHO/UNPD - adjusted for incompleteness [VR]
	30	2021.5	WHO Vital Registration (recalculated - unadjusted) [VR]
Tanzania	93	2014.6	
	3	1967.2	1967 Census [Others Direct]
	3	1973.3	1973 National Demographic Survey [Others Direct]
	3	1988.2	1988 Census [Others Direct]
	18	1995.5	1996 Demographic and Health Survey [DHS Direct]
	3	2002.2	2002 Census [Others Direct]
	18	2003.8	2004-2005 Demographic and Health Survey [DHS Direct]
	18	2008.9	2010 Demographic and Health Survey [DHS Direct]
	3	2010.4	2010-2011 National Panel Survey [Others Direct]
	6	2012.2	2012-2013 National Panel Survey [Others Direct]
	18	2014.6	2015-2016 Demographic and Health Survey [DHS Direct]
Thailand	93	2019.5	
	3	1995.5	1995-1996 Survey of Population Change [Others Direct]
	90	2019.5	Recalculated based on WHO - adjusted for incompleteness [VR]
Timor Leste	39	2015.2	
	3	1991.7	1997 Demographic and Health Survey [DHS Direct]
	18	2008.6	2009-2010 Demographic and Health Survey [DHS Direct]
	6	2014.5	2015 Census [Others Direct]
	12	2015.2	2016 Demographic and Health Survey [DHS Direct]
Togo	41	2012.8	
	3	1961.4	1961 Demographic survey [Others Direct]
	3	1981.4	1981 Census [Others Direct]
	18	1997.1	1998 Demographic and Health Survey [DHS Direct]
	17	2012.8	2013-2014 Demographic and Health Survey [DHS Direct]
Tonga	72	2018.5	
	3	2006.4	2006 Census [Others Direct]
	39	2005.5	Recalculated based on WHO - adjusted for incompleteness [VR]
	30	2018.5	WHO Vital Registration (recalculated - unadjusted) [VR]
Trinidad and Tobago	87	2017.5	
	87	2017.5	WHO/UNPD Vital Registration (recalculated - unadjusted) [VR]
Tunisia	37	2017.5	
	7	2000.2	2003 World Health Survey [Others Direct]
	21	2000.5	Recalculated based on WHO - adjusted for incompleteness [VR]
	9	2017.5	WHO Vital Registration (recalculated - unadjusted) [VR]
Turkey	33	2019.5	

Continued on next page

Table 27 – continued from previous page

Country	# obs.	Most recent obs. year	Data series name [source type]
	3	2002.7	2003 National Verbal Autopsy Survey [Others Direct]
	30	2019.5	WHO Vital Registration (recalculated - unadjusted) [VR]
Turkmenistan	81	2015.5	
	81	2015.5	Recalculated based on WHO/UNPD - adjusted for incompleteness [VR]
Turks and Caicos Islands	6	2002	
	6	2002	WHO Vital Registration (recalculated - unadjusted) [VR]
Tuvalu	8	2002	
	8	2002	WHO Vital Registration (recalculated - unadjusted) [VR]
Uganda	90	2015.4	
	18	1994.2	1995 Demographic and Health Survey [DHS Direct]
	18	1999.7	2000-2001 Demographic and Health Survey [DHS Direct]
	18	2005.3	2006 Demographic and Health Survey [DHS Direct]
	18	2010.4	2011 Demographic and Health Survey [DHS Direct]
	18	2015.4	2016 Demographic and Health Survey [DHS Direct]
Ukraine	153	2021.5	
	153	2021.5	WHO/Human Mortality Database Vital Registration (recalculated - unadjusted) [VR]
United Arab Emirates	3	1997.2	
	3	1997.2	2003 World Health Survey [Others Direct]
United Kingdom	93	2019.5	
	93	2019.5	WHO/Human Mortality Database Vital Registration (recalculated - unadjusted) [VR]
United States of America	153	2020.5	
	153	2020.5	WHO/Human Mortality Database Vital Registration (recalculated - unadjusted) [VR]
Uruguay	93	2020.5	
	93	2020.5	WHO Vital Registration (recalculated - unadjusted) [VR]
Uzbekistan	99	2021.5	
	99	2021.5	Recalculated based on WHO - adjusted for incompleteness [VR]
Vanuatu	3	2009.4	
	3	2009.4	2009 Census [Others Direct]
Venezuela (Bolivarian Republic of)	78	2016.5	
	45	2016.5	Recalculated based on WHO/UNPD - adjusted for incompleteness [VR]
	33	2000.5	WHO Vital Registration (recalculated - unadjusted) [VR]
Vietnam	39	2017.5	
	3	1978.8	1979 Census [Others Direct]
	3	1988.5	1989 Census [Others Direct]
	3	1998.7	1999 Census [Others Direct]
	3	1997.2	2003 World Health Survey [Others Direct]
	3	2006.8	2007 Population Change and Family Planning Survey [Others Direct]
	3	2007.5	2008 Population Change and Family Planning Survey [Others Direct]

Continued on next page

Table 27 – continued from previous page

Country	# obs.	Most recent obs. year	Data series name [source type]
	3	2010.8	2011 Population Change and Family Planning Survey [Others Direct]
	3	2011.8	2012 Population Change and Family Planning Survey [Others Direct]
	3	2012.8	2013 Population Change and Family Planning Survey [Others Direct]
	3	2013.5	2014 Intercensal Demographic Survey [Others Direct]
	3	2014.8	2015 Population Change and Family Planning Survey [Others Direct]
	3	2016.5	2017 Population Change and Family Planning Survey [Others Direct]
	3	2017.5	2018 Population Change and Family Planning Survey [Others Direct]
Yemen			
Zambia	111	2017.5	
	18	1995.5	1996 Demographic and Health Survey [DHS Direct]
	18	2000.8	2001-2002 Demographic and Health Survey [DHS Direct]
	18	2002.2	2003 World Health Survey [Others Direct]
	18	2006.2	2007 Demographic and Health Survey [DHS Direct]
	3	2010.3	2010 Census [Others Direct]
	18	2012.6	2013-2014 Demographic and Health Survey [DHS Direct]
	18	2017.5	2018 Demographic and Health Survey [DHS Direct]
Zimbabwe	130	2016.5	
	3	1992.1	1992 Census [Others Direct]
	18	1993.5	1994 Demographic and Health Survey [DHS Direct]
	3	1997.1	1997 Inter-Censal Demographic Survey [Others Direct]
	18	1998.7	1999 Demographic and Health Survey [DHS Direct]
	3	2002.1	2002 Census [Others Direct]
	16	2002.2	2003 World Health Survey [Others Direct]
	18	2004.6	2005-2006 Demographic and Health Survey [DHS Direct]
	18	2009.7	2010-2011 Demographic and Health Survey [DHS Direct]
	3	2012.1	2012 Census [Others Direct]
	9	2012.2	2014 Multiple Indicator Cluster Survey [Others Direct]
	18	2014.5	2015 Demographic and Health Survey [DHS Direct]
	3	2016.5	2017 Inter-Censal Demographic Survey [Others Direct]

Table 28: **Sex ratio data sources for age group 20–24, by country.** For each country, the total number of observations and the most recent reference year are shown after the country name. For each country-specific data series, the number of observations and the most recent reference year within that series is shown before each data series name. The source type that each data series falls in is shown in parentheses after each data series name.

Country	# obs.	Most recent obs. year	Data series name [source type]
Afghanistan	39	2014.4	
	3	1973	1972 National Demographic and Family Guidance Survey [Others Direct]
	3	1979	1979 Census [Others Direct]
	3	2008.1	2010 Afghanistan Mortality Survey (AMS) Excluding South Zone (Household data) [Others Direct]
	12	2008.7	2010 Afghanistan Mortality Survey (AMS) [DHS Direct]
	18	2014.4	2015 Demographic and Health Survey [DHS Direct]
Albania	69	2021.5	
	3	2011.1	2011 Census [Others Direct]
	66	2021.5	WHO Vital Registration (recalculated - unadjusted) [VR]
Algeria	69	2020.5	
	3	1970.5	1970 Demographic Survey [Others Direct]
	66	2020.5	WHO Vital Registration (recalculated - unadjusted) [VR]
Andorra	10	2019.5	
	10	2019.5	WHO Vital Registration (recalculated - unadjusted) [VR]
Angola	21	2014.8	
	3	2013.9	2014 Census [Others Direct]
	18	2014.8	2016 Inquerito de Indicadores Multiplos e de Saude [DHS Direct]
Anguilla			
Antigua and Barbuda	40	2017.5	
	40	2017.5	WHO Vital Registration (recalculated - unadjusted) [VR]
Argentina	96	2020.5	
	96	2020.5	WHO Vital Registration (recalculated - unadjusted) [VR]
Armenia	95	2021.5	
	95	2021.5	WHO Vital Registration (recalculated - unadjusted) [VR]
Australia	153	2020.5	
	153	2020.5	WHO/Human Mortality Database Vital Registration (recalculated - unadjusted) [VR]
Austria	153	2020.5	
	153	2020.5	WHO/Human Mortality Database Vital Registration (recalculated - unadjusted) [VR]
Azerbaijan	90	2021.5	
	90	2021.5	Recalculated based on WHO - adjusted for incompleteness [VR]
Bahamas	93	2020.5	
	93	2020.5	Recalculated based on WHO/UNPD - adjusted for incompleteness [VR]
Bahrain	92	2019.5	
	92	2019.5	WHO/UNPD Vital Registration (recalculated - unadjusted) [VR]
Bangladesh	123	2020.5	
	3	1964	1962-1965 Population Growth Estimation Experiment [Others Direct]

Continued on next page

Table 28 – continued from previous page

Country	# obs.	Most recent obs. year	Data series name [source type]
	3	1974.5	1974 Retrospective Fertility and Mortality Survey (UN SA) [Others Direct]
	3	1999.8	1999-2000 Demographic and Health Survey [Others Direct]
	12	2001.7	2003 World Health Survey [Others Direct]
	3	2010	2010 Maternal Health Services and Maternal Mortality Survey [Others Direct]
	3	2010.7	2011 Census [Others Direct]
	3	2016	2016 Maternal Health Services and Maternal Mortality Survey [Others Direct]
	3	2016.5	Report on Sample Vital Registration System 2016 [Others Direct]
	3	2017.5	Report on Sample Vital Registration System 2016 [Others Direct]
	3	2018.5	Report on Sample Vital Registration System 2016 [Others Direct]
	3	2019.5	Report on Sample Vital Registration System 2016 [Others Direct]
	3	2020.5	Report on Sample Vital Registration System 2016 [Others Direct]
	78	2015.5	Sample Vital Statistics [VR]
Barbados	75	2013.5	
	75	2013.5	WHO Vital Registration (recalculated - unadjusted) [VR]
Belarus	150	2019.5	
	150	2019.5	WHO/Human Mortality Database Vital Registration (recalculated - unadjusted) [VR]
Belgium	153	2020.5	
	153	2020.5	WHO/Human Mortality Database Vital Registration (recalculated - unadjusted) [VR]
Belize	81	2018.5	
	81	2018.5	Recalculated based on WHO - adjusted for incompleteness [VR]
Benin	63	2016.8	
	18	1995.4	1996 Demographic and Health Survey [DHS Direct]
	18	2005.6	2006 Demographic and Health Survey [DHS Direct]
	9	2012.5	2014 Multiple Indicator Cluster Survey [Others Direct]
	18	2016.8	2018 Demographic and Health Survey [DHS Direct]
Bhutan	9	2012.5	
	3	1993.1	1994 National Health Survey [Others Direct]
	3	2004.9	2005 Census [Others Direct]
	3	2012.5	2012 National Health Survey [Others Direct]
Bolivia (Plurinational State of)	60	2007.1	
	3	1979.8	1980 National Demographic Survey [Others Direct]
	3	1991.9	1992 Census [Others Direct]
	18	1992.8	1993-1994 Demographic and Health Survey [DHS Direct]
	18	2002.6	2003 Demographic and Health Survey [DHS Direct]
	18	2007.1	2008 Demographic and Health Survey [DHS Direct]
Bosnia and Herzegovina	63	2016.5	
	63	2016.5	WHO Vital Registration (recalculated - unadjusted) [VR]
Botswana	21	2016.5	

Continued on next page

Table 28 – continued from previous page

Country	# obs.	Most recent obs. year	Data series name [source type]
	3	1981.1	1981 Census [Others Direct]
	3	1991.1	1991 Census [Others Direct]
	3	1997.9	1998 Demographic Survey [Others Direct]
	3	2001.1	2001 Census [Others Direct]
	3	2006.2	2006 Demographic Survey [Others Direct]
	3	2011.1	2011 Census [Others Direct]
	3	2016.5	2017 Demographic Survey [Others Direct]
Brazil	117	2020.5	
	18	1995.2	1996 Demographic and Health Survey [DHS Direct]
	3	2010.2	2010 Census [Others Direct]
	96	2020.5	WHO Vital Registration (recalculated - unadjusted) [VR]
British Virgin Islands			
Brunei	83	2019.5	
	83	2019.5	WHO Vital Registration (recalculated - unadjusted) [VR]
Bulgaria	156	2021.5	
	156	2021.5	WHO/Human Mortality Database Vital Registration (recalculated - unadjusted) [VR]
Burkina Faso	81	2009.3	
	3	1960.5	1960-1961 Survey [Others Direct]
	3	1985.5	1985 Census [Others Direct]
	3	1995.5	1996 Census [Others Direct]
	18	1997.8	1998-1999 Demographic and Health Survey [DHS Direct]
	18	2002.4	2003 Demographic and Health Survey [DHS Direct]
	12	2001.7	2003 World Health Survey [Others Direct]
	3	2006.4	2006 Census [Others Direct]
	3	2007.3	2008 Global Fund Evaluation Survey [Others Direct]
	18	2009.3	2010 Demographic and Health Survey [DHS Direct]
Burundi	42	2015.8	
	3	1964.7	1965 Population Survey [Others Direct]
	3	2008.2	2008 Census [Others Direct]
	18	2009.6	2010 Demographic and Health Survey [DHS Direct]
	18	2015.8	2017 Demographic and Health Survey [DHS Direct]
Cambodia	72	2013.4	
	18	1999.1	2000 Demographic and Health Survey [DHS Direct]
	18	2004.7	2005 Demographic and Health Survey [DHS Direct]
	18	2009.5	2010 Demographic and Health Survey [DHS Direct]
	18	2013.4	2014 Demographic and Health Survey [DHS Direct]
Cameroon	81	2017.4	
	3	1975.8	1976 Census [Others Direct]
	3	1986.8	1987 Census [Others Direct]
	18	1997.1	1998 Demographic and Health Survey [DHS Direct]
	18	2003.1	2004 Demographic and Health Survey [DHS Direct]
	3	2005.4	2005 Census [Others Direct]
	18	2010	2011 Demographic and Health Survey [DHS Direct]

Continued on next page

Table 28 – continued from previous page

Country	# obs.	Most recent obs. year	Data series name [source type]
	18	2017.4	2018 Demographic and Health Survey [DHS Direct]
Canada	153	2020.5	
	153	2020.5	WHO/Human Mortality Database Vital Registration (recalculated - unadjusted) [VR]
Cape Verde	81	2018.5	
	3	2000	2000 Census [Others Direct]
	3	2009.5	2010 Census [Others Direct]
	18	1999.5	Recalculated based on WHO/UNPD - adjusted for incompleteness [VR]
	57	2018.5	WHO/UNPD Vital Registration (recalculated - unadjusted) [VR]
Central African Republic	27	2003.4	
	3	1959.5	1959-1960 Survey [Others Direct]
	3	1988.4	1988 Census [Others Direct]
	18	1993.7	1994-1995 Demographic and Health Survey [DHS Direct]
	3	2003.4	2003 Census [Others Direct]
Chad	57	2013.7	
	18	1995.9	1996-1997 Demographic and Health Survey [DHS Direct]
	18	2003.5	2004 Demographic and Health Survey [DHS Direct]
	3	2008.9	2009 Census [Others Direct]
	18	2013.7	2014-2015 Demographic and Health Survey [DHS Direct]
Chile	153	2020.5	
	153	2020.5	WHO/Human Mortality Database Vital Registration (recalculated - unadjusted) [VR]
China	48	2010.5	
	3	1973.5	1964-1982 Adjusted Census Deaths (1964-1982) [Others Direct]
	3	1986.5	1982-1990 Adjusted Census Deaths (1982-1990) [Others Direct]
	3	1987	1987 China 1987 One-per-Hundred National Population Survey [Others Direct]
	3	1995.5	1990-2000 Adjusted Census Deaths (1990-2000) [Others Direct]
	3	1995.4	1995 China 1995 One-percent Sample Survey [Others Direct]
	3	2000.5	1999-2000 Adjusted Census Deaths (1999-2000) [Others Direct]
	3	2005.5	2000-2010 Adjusted Census Deaths (2000-2010) [Others Direct]
	3	2007.4	2007 China 2007 Annual Population Change Survey [Others Direct]
	3	2008.4	2008 China 2008 Annual Population Change Survey [Others Direct]
	21	2010.5	China CDC Surveillance surveys [VR]
Colombia	102	2020.5	
	18	2014.1	2015 Demographic and Health Survey [DHS Direct]
	84	2020.5	Recalculated based on WHO - adjusted for incompleteness [VR]
Comoros	12	2009.6	
	3	1958.2	1958 Census [Others Direct]
	3	2003.2	2003 Census [Others Direct]
	6	2009.6	2012 Demographic and Health Survey [DHS Direct]
Congo	45	2012.9	
	18	2004.5	2005 Demographic and Health Survey [DHS Direct]
	18	2010.7	2011-2012 Demographic and Health Survey [DHS Direct]

Continued on next page

Table 28 – continued from previous page

Country	# obs.	Most recent obs. year	Data series name [source type]
	9	2012.9	2015 Multiple Indicator Cluster Survey [Others Direct]
Democratic Republic of the Congo	33	2012.6	
	15	2006	2007 Demographic and Health Survey [DHS Direct]
	18	2012.6	2013-2014 Demographic and Health Survey [DHS Direct]
Cook Islands	25	2008.5	
	25	2008.5	WHO Vital Registration (recalculated - unadjusted) [VR]
Costa Rica	96	2020.5	
	96	2020.5	Recalculated based on WHO - adjusted for incompleteness [VR]
Cote d'Ivoire	60	2010.9	
	3	1977.5	1978-1979 Demographic Survey Repeated Passages [Others Direct]
	18	1993.4	1994 Demographic and Health Survey [DHS Direct]
	3	1998.4	1998 Census [Others Direct]
	6	2000.2	2003 World Health Survey [Others Direct]
	12	2004.1	2005 AIDS Indicator Survey [DHS Direct]
	18	2010.9	2011-2012 Demographic and Health Survey [DHS Direct]
Croatia	117	2021.5	
	3	2010.8	2011 Census [Others Direct]
	114	2021.5	WHO/Human Mortality Database Vital Registration (recalculated - unadjusted) [VR]
Cuba	96	2020.5	
	96	2020.5	WHO Vital Registration (recalculated - unadjusted) [VR]
Cyprus	92	2020.5	
	56	2020.5	Recalculated based on WHO - adjusted for incompleteness [VR]
	36	2000.5	WHO Vital Registration (recalculated - unadjusted) [VR]
Czech Republic	156	2021.5	
	156	2021.5	WHO/Human Mortality Database Vital Registration (recalculated - unadjusted) [VR]
Denmark	156	2021.5	
	156	2021.5	WHO/Human Mortality Database Vital Registration (recalculated - unadjusted) [VR]
Djibouti			
Dominica	37	2015	
	17	1999.5	Recalculated based on WHO - adjusted for incompleteness [VR]
	20	2015	WHO Vital Registration (recalculated - unadjusted) [VR]
Dominican Republic	48	2010.4	
	18	2001.5	2002 Demographic and Health Survey [DHS Direct]
	9	2001.2	2003 World Health Survey [Others Direct]
	18	2006.2	2007 Demographic and Health Survey [DHS Direct]
	3	2010.4	2010 Census [Others Direct]
Ecuador	96	2020.5	
	3	1997.2	2003 World Health Survey [Others Direct]
	93	2020.5	WHO Vital Registration (recalculated - unadjusted) [VR]
Egypt	93	2019.5	

Continued on next page

Table 28 – continued from previous page

Country	# obs.	Most recent obs. year	Data series name [source type]
	42	2019.5	Recalculated based on WHO - adjusted for incompleteness [VR]
	51	2005.5	WHO Vital Registration (recalculated - unadjusted) [VR]
El Salvador	87	2019.5	
	3	1992.2	1992 Census [Others Direct]
	84	2019.5	WHO/UNPD Vital Registration (recalculated - unadjusted) [VR]
Equatorial Guinea			
Eritrea			
Estonia	156	2020.5	
	3	2011.6	2011-2012 Census [Others Direct]
	153	2020.5	WHO/Human Mortality Database Vital Registration (recalculated - unadjusted) [VR]
Ethiopia	84	2015	
	3	1983.9	1984 Census [Others Direct]
	15	1997.1	2000 Demographic and Health Survey [DHS Direct]
	12	2001.7	2003 World Health Survey [Others Direct]
	18	2004.2	2005 Demographic and Health Survey [DHS Direct]
	3	2006.9	2007 Census [Others Direct]
	15	2009.9	2011 Demographic and Health Survey [DHS Direct]
	18	2015	2016 Demographic and Health Survey [DHS Direct]
Federated States of Micronesia			
Fiji	42	2019.5	
	42	2019.5	Recalculated based on WHO - adjusted for incompleteness [VR]
Finland	156	2021.5	
	156	2021.5	WHO/Human Mortality Database Vital Registration (recalculated - unadjusted) [VR]
France	153	2020.5	
	153	2020.5	WHO/Human Mortality Database Vital Registration (recalculated - unadjusted) [VR]
Gabon	24	2010.5	
	12	1999	2000 Demographic and Health Survey [DHS Direct]
	12	2010.5	2012 Demographic and Health Survey [DHS Direct]
The Gambia	33	2018.8	
	3	2012.8	2013 Census [Others Direct]
	12	2011.6	2013 Demographic and Health Survey [DHS Direct]
	18	2018.8	2020 Demographic and Health Survey [DHS Direct]
Georgia	90	2021.5	
	33	2001.5	Recalculated based on WHO - adjusted for incompleteness [VR]
	57	2021.5	WHO Vital Registration (recalculated - unadjusted) [VR]
Germany	153	2021.5	
	153	2021.5	WHO/Human Mortality Database Vital Registration (recalculated - unadjusted) [VR]
Ghana	30	2014.2	
	12	2001.7	2003 World Health Survey [Others Direct]
	12	2005.5	2007 Maternal Health Survey [DHS Direct]
	3	2010.3	2010 Census [Others Direct]

Continued on next page

Table 28 – continued from previous page

Country	# obs.	Most recent obs. year	Data series name [source type]
	3	2014.2	2017 Demographic and Health Survey [Others Direct]
Greece	153	2020.5	
	153	2020.5	WHO/Human Mortality Database Vital Registration (recalculated - unadjusted) [VR]
Grenada	76	2018.5	
	32	2000.5	Recalculated based on WHO - adjusted for incompleteness [VR]
	44	2018.5	WHO Vital Registration (recalculated - unadjusted) [VR]
Guatemala	117	2017.5	
	12	1993.9	1995 Demographic and Health Survey [DHS Direct]
	18	2013.7	2014-2015 Demographic and Health Survey [DHS Direct]
	87	2017.5	WHO Vital Registration (recalculated - unadjusted) [VR]
Guinea	60	2013.8	
	3	1996.4	1996 Census [Others Direct]
	18	1998.3	1999 Demographic and Health Survey [DHS Direct]
	18	2004.1	2005 Demographic and Health Survey [DHS Direct]
	18	2011.4	2012 Demographic and Health Survey [DHS Direct]
	3	2013.8	2014 Census [Others Direct]
Guinea-Bissau	12	2012.2	
	3	2008.8	2009 Census [Others Direct]
	9	2012.2	2014 Multiple Indicator Cluster Survey [Others Direct]
Guyana	78	2014.5	
	78	2014.5	WHO Vital Registration (recalculated - unadjusted) [VR]
Haiti	48	2015.8	
	12	1998.6	2000 Demographic and Health Survey [DHS Direct]
	18	2004.7	2005-2006 Demographic and Health Survey [DHS Direct]
	18	2015.8	2017 Demographic and Health Survey [DHS Direct]
Honduras	3	2001.1	
	3	2001.1	2001 Census [Others Direct]
Hungary	153	2020.5	
	153	2020.5	WHO/Human Mortality Database Vital Registration (recalculated - unadjusted) [VR]
Iceland	132	2021	
	132	2021	WHO/Human Mortality Database Vital Registration (recalculated - unadjusted) [VR]
India	141	2019.5	
	3	1970.5	1971 Census [Others Direct]
	3	1980.5	1981 Census [Others Direct]
	3	1991.9	1992-1993 National Family Health Survey [Others Direct]
	3	1998.2	1998-1999 National Family Health Survey [Others Direct]
	12	2001.7	2003 World Health Survey [Others Direct]
	117	2019.5	Sample Registration System [VR]
Indonesia	63	2010.3	
	6	1964.5	1964-1965 National Socio-economic Survey [Others Direct]
	18	1993.5	1994 Demographic and Health Survey [DHS Direct]
	12	2001.2	2002-2003 Demographic and Health Survey [DHS Direct]
	15	2006.4	2007 Demographic and Health Survey [DHS Direct]

Continued on next page

Table 28 – continued from previous page

Country	# obs.	Most recent obs. year	Data series name [source type]
	3	2009.9	2010 Census [Others Direct]
	9	2010.3	2012 Demographic and Health Survey [DHS Direct]
Iran (Islamic Republic of)	42	2017.5	
	3	1975.5	1976 Census [Others Direct]
	3	1999.8	2000 Demographic and Health Survey [Others Direct]
	3	2004.5	Based on 2004 DH - adjusted for incompleteness [Others Direct]
	33	2017.5	Recalculated based on WHO - adjusted for incompleteness [VR]
Iraq	3	2004.6	
	3	2004.6	2006 Iraq Family Health Survey [Others Direct]
Ireland	156	2020.5	
	3	2010.8	2011 Census [Others Direct]
	153	2020.5	WHO/Human Mortality Database Vital Registration (recalculated - unadjusted) [VR]
Israel	141	2021.5	
	141	2021.5	WHO/Human Mortality Database Vital Registration (recalculated - unadjusted) [VR]
Italy	153	2020.5	
	153	2020.5	WHO/Human Mortality Database Vital Registration (recalculated - unadjusted) [VR]
Jamaica	3	2010.2	
	3	2010.2	2011 Census [Others Direct]
Japan	153	2021.5	
	153	2021.5	WHO/Human Mortality Database Vital Registration (recalculated - unadjusted) [VR]
Jordan	9	2014.9	
	6	1995.4	1997 Population and Family Health Survey [DHS Direct]
	3	2014.9	2015 Census [Others Direct]
Kazakhstan	102	2021.5	
	3	1997.2	2003 World Health Survey [Others Direct]
	99	2021.5	WHO Vital Registration (recalculated - unadjusted) [VR]
Kenya	84	2013.3	
	18	1997.1	1998 Demographic and Health Survey [DHS Direct]
	18	2002.2	2003 Demographic and Health Survey [DHS Direct]
	9	2001.2	2003 World Health Survey [Others Direct]
	18	2007.8	2008-2009 Demographic and Health Survey [DHS Direct]
	3	2009.2	2009 Census [Others Direct]
	18	2013.3	2014 Demographic and Health Survey [DHS Direct]
Kiribati			
Democratic People's Republic of Korea	3	2008.3	
	3	2008.3	2008 Census [Others Direct]
Republic of Korea	99	2021.5	

Continued on next page

Table 28 – continued from previous page

Country	# obs.	Most recent obs. year	Data series name [source type]
	99	2021.5	WHO/Human Mortality Database Vital Registration (recalculated - unadjusted) [VR]
Kosovo			
Kuwait	87	2019.5	
	87	2019.5	WHO Vital Registration (recalculated - unadjusted) [VR]
Kyrgyzstan	99	2021.5	
	99	2021.5	WHO Vital Registration (recalculated - unadjusted) [VR]
Lao People's Democratic Republic	15	2014.8	
	9	2001.2	2003 World Health Survey [Others Direct]
	3	2004.8	2005 Census [Others Direct]
	3	2014.8	2015 Census [Others Direct]
Latvia	156	2021.5	
	156	2021.5	WHO/Human Mortality Database Vital Registration (recalculated - unadjusted) [VR]
Lebanon			
Lesotho	63	2015.5	
	3	1995.8	1996 Census [Others Direct]
	3	2000.9	2001 Demographic Survey [Others Direct]
	18	2003.7	2004 Demographic and Health Survey [DHS Direct]
	18	2008.7	2009 Demographic and Health Survey [DHS Direct]
	18	2013.7	2014 Demographic and Health Survey [DHS Direct]
	3	2015.5	2016 Census [Others Direct]
Liberia	45	2018.2	
	3	1969.5	1969-1970 Population Growth Survey [Others Direct]
	15	2005.9	2006-2007 Demographic and Health Survey [DHS Direct]
	18	2012.2	2013 Demographic and Health Survey [DHS Direct]
	9	2018.2	2020 Demographic and Health Survey [DHS Direct]
Libya			
Lithuania	162	2021.5	
	3	2010.8	2011 Census [Others Direct]
	3	2017.5	2018 Statistical Survey [Others Direct]
	156	2021.5	WHO/Human Mortality Database Vital Registration (recalculated - unadjusted) [VR]
Luxembourg	138	2020.5	
	138	2020.5	WHO/Human Mortality Database Vital Registration (recalculated - unadjusted) [VR]
Macedonia	93	2020.5	
	93	2020.5	Recalculated based on WHO - adjusted for incompleteness [VR]
Madagascar	51	2007.8	
	3	1993.1	1993 Census [Others Direct]
	18	1996.7	1997 Demographic and Health Survey [DHS Direct]
	12	2002.3	2003-2004 Demographic and Health Survey [DHS Direct]
	18	2007.8	2008-2009 Demographic and Health Survey [DHS Direct]
Malawi	123	2018.2	

Continued on next page

Table 28 – continued from previous page

Country	# obs.	Most recent obs. year	Data series name [source type]
	3	1971.1	1970-1972 Population Change Survey [Others Direct]
	12	1991.2	1992 Demographic and Health Survey [DHS Direct]
	3	1998.2	1998 Census [Others Direct]
	18	1999.5	2000 Demographic and Health Survey [DHS Direct]
	18	2002.2	2003 World Health Survey [Others Direct]
	18	2003.8	2004 Demographic and Health Survey [DHS Direct]
	3	2008	2008 Census [Others Direct]
	18	2009.4	2010 Demographic and Health Survey [DHS Direct]
	9	2011.9	2014 Multiple Indicator Cluster Survey [Others Direct]
	18	2014.7	2015-2016 Demographic and Health Survey [DHS Direct]
	3	2018.2	2018 Census [Others Direct]
Malaysia	90	2021.5	
	90	2021.5	WHO Vital Registration (recalculated - unadjusted) [VR]
Maldives	92	2020.5	
	18	1994.5	Recalculated based on WHO - adjusted for incompleteness [VR]
	74	2020.5	WHO Vital Registration (recalculated - unadjusted) [VR]
Mali	84	2017.6	
	3	1986.8	1987 Census [Others Direct]
	18	2000	2001 Demographic and Health Survey [DHS Direct]
	9	2001.2	2003 World Health Survey [Others Direct]
	18	2005.3	2006 Demographic and Health Survey [DHS Direct]
	18	2011.8	2012-2013 Demographic and Health Survey [DHS Direct]
	18	2017.6	2018 Demographic and Health Survey [DHS Direct]
Malta	87	2021.5	
	87	2021.5	WHO Vital Registration (recalculated - unadjusted) [VR]
Marshall Islands			
Mauritania	21	2018.4	
	3	1977	1976-1977 Census [Others Direct]
	3	1987.8	1988 Census [Others Direct]
	3	2012.8	2013 Census [Others Direct]
	12	2018.4	2021 Demographic and Health Survey [DHS Direct]
Mauritius	96	2020.5	
	96	2020.5	WHO Vital Registration (recalculated - unadjusted) [VR]
Mexico	96	2020.5	
	96	2020.5	WHO Vital Registration (recalculated - unadjusted) [VR]
Republic of Moldova	93	2020.5	
	93	2020.5	WHO Vital Registration (recalculated - unadjusted) [VR]
Monaco			
Mongolia	90	2019.5	
	3	2009.5	2010 Census [Others Direct]
	27	1999.5	Recalculated based on WHO - adjusted for incompleteness [VR]
	60	2019.5	WHO Vital Registration (recalculated - unadjusted) [VR]
Montenegro	92	2021.5	
	42	2002.5	Recalculated based on WHO - adjusted for incompleteness [VR]

Continued on next page

Table 28 – continued from previous page

Country	# obs.	Most recent obs. year	Data series name [source type]
	50	2021.5	WHO Vital Registration (recalculated - unadjusted) [VR]
Montserrat			
Morocco	41	2010	
	17	1991	1992 Demographic and Health Survey [DHS Direct]
	18	2002.7	2003-2004 Demographic and Health Survey [DHS Direct]
	3	1997.2	2003 World Health Survey [Others Direct]
	3	2010	2009-2010 National Demographic Survey with repeated passages [Others Direct]
Mozambique	45	2010.4	
	9	1995.2	1997 Demographic and Health Survey [DHS Direct]
	18	2002.6	2003 Demographic and Health Survey [DHS Direct]
	18	2010.4	2011 Demographic and Health Survey [DHS Direct]
Myanmar	12	2014.4	
	3	2013.8	2014 Census [Others Direct]
	9	2014.4	2015-2016 Demographic and Health Survey [DHS Direct]
Namibia	84	2012.3	
	18	1991.5	1992 Demographic and Health Survey [DHS Direct]
	18	1999.7	2000 Demographic and Health Survey [DHS Direct]
	3	2001	2001 Census [Others Direct]
	6	2000.2	2003 World Health Survey [Others Direct]
	18	2005.8	2006-2007 Demographic and Health Survey [DHS Direct]
	3	2011.2	2011 Census [Others Direct]
	18	2012.3	2013 Demographic and Health Survey [DHS Direct]
Nauru	2	2000	
	2	2000	WHO Vital Registration (recalculated - unadjusted) [VR]
Nepal	63	2014.9	
	18	1995	1996 Demographic and Health Survey [DHS Direct]
	3	2001	2001 Census [Others Direct]
	15	2000.2	2003 World Health Survey [Others Direct]
	12	2004.6	2006 Demographic and Health Survey [DHS Direct]
	3	2010.5	2011 Census [Others Direct]
	12	2014.9	2016 Demographic and Health Survey [DHS Direct]
Netherlands	156	2021.5	
	156	2021.5	WHO/Human Mortality Database Vital Registration (recalculated - unadjusted) [VR]
New Zealand	159	2021.5	
	3	2005.7	2006 Census [Others Direct]
	156	2021.5	WHO/Human Mortality Database Vital Registration (recalculated - unadjusted) [VR]
Nicaragua			
Niger	57	2011.1	
	18	1991.2	1992 Demographic and Health Survey [DHS Direct]
	3	2000.9	2001 Census [Others Direct]
	18	2005	2006 Demographic and Health Survey [DHS Direct]
	18	2011.1	2012 Demographic and Health Survey [DHS Direct]
Nigeria	63	2017.6	

Continued on next page

Table 28 – continued from previous page

Country	# obs.	Most recent obs. year	Data series name [source type]
	3	1965.6	1965-1966 Rural Demographic Sample Survey [Others Direct]
	18	2007.4	2008 Demographic and Health Survey [DHS Direct]
	3	2010.5	2010 GHS Panel Survey [Others Direct]
	3	2012.5	2012-2013 GHS Panel Survey [Others Direct]
	18	2012.1	2013 Demographic and Health Survey [DHS Direct]
	18	2017.6	2018 Demographic and Health Survey [DHS Direct]
Niue			
Norway	156	2021.5	
	156	2021.5	WHO/Human Mortality Database Vital Registration (recalculated - unadjusted) [VR]
Oman			
Pakistan	33	2007.5	
	12	1965.5	1962-1965 Population Growth Estimation Experiment [Others Direct]
	3	1970	1971 Population Growth Survey I [Others Direct]
	3	2006.4	2006-2007 Demographic and Health Survey [Others Direct]
	15	2007.5	Pakistan Demographic Survey [VR]
Palau	44	2018.5	
	44	2018.5	WHO/UNPD Vital Registration (recalculated - unadjusted) [VR]
Panama	96	2021.5	
	96	2021.5	Recalculated based on WHO - adjusted for incompleteness [VR]
Papua New Guinea	18	2015.8	
	18	2015.8	2018 Demographic and Health Survey [DHS Direct]
Paraguay	15	2002.2	
	3	2002.2	2002 Census [Others Direct]
	12	2001.7	2003 World Health Survey [Others Direct]
Peru	102	2009.7	
	18	1990.7	1991-1992 Demographic and Health Survey [DHS Direct]
	18	1995.6	1996 Demographic and Health Survey [DHS Direct]
	18	1999.5	2000 Demographic and Health Survey [DHS Direct]
	18	2002.9	2006 Demographic and Health Survey [DHS Direct]
	18	2006	2008 Demographic and Health Survey [DHS Direct]
	12	2009.7	2011 Demographic and Health Survey [DHS Direct]
Philippines	117	2019.5	
	18	1992.2	1993 National Demographic and Health Survey [DHS Direct]
	18	1997.1	1998 National Demographic and Health Survey [DHS Direct]
	60	2019.5	Recalculated based on WHO/UNPD - adjusted for incompleteness [VR]
	21	1999.5	WHO Vital Registration (recalculated - unadjusted) [VR]
Poland	156	2021.5	
	156	2021.5	WHO/Human Mortality Database Vital Registration (recalculated - unadjusted) [VR]
Portugal	159	2021.5	
	3	2010.8	2011 Census [Others Direct]
	156	2021.5	WHO/Human Mortality Database Vital Registration (recalculated - unadjusted) [VR]
Qatar	87	2020.5	

Continued on next page

Table 28 – continued from previous page

Country	# obs.	Most recent obs. year	Data series name [source type]
	87	2020.5	WHO Vital Registration (recalculated - unadjusted) [VR]
Romania	99	2021.5	
	99	2021.5	WHO Vital Registration (recalculated - unadjusted) [VR]
Russian Federation	150	2020.5	
	150	2020.5	WHO/Human Mortality Database Vital Registration (recalculated - unadjusted) [VR]
Rwanda	90	2018.8	
	15	1999.4	2000 Demographic and Health Survey [DHS Direct]
	3	2002.1	2002 Census [Others Direct]
	15	2004.1	2005 Demographic and Health Survey [DHS Direct]
	18	2009.7	2010 Demographic and Health Survey [DHS Direct]
	3	2012.1	2012 Census [Others Direct]
	18	2013.8	2014-2015 Demographic and Health Survey [DHS Direct]
	18	2018.8	2020 Demographic and Health Survey [DHS Direct]
Saint Kitts and Nevis	50	2015.5	
	50	2015.5	WHO Vital Registration (recalculated - unadjusted) [VR]
Saint Lucia	84	2018.5	
	84	2018.5	WHO Vital Registration (recalculated - unadjusted) [VR]
Samoa	12	2016.4	
	3	1999	1999 Demographic and Health Survey [Others Direct]
	3	2006.4	2006 Census [Others Direct]
	3	2011.4	2011 Population and Housing Census [Others Direct]
	3	2016.4	2016 Population and Housing Census [Others Direct]
San Marino			
Sao Tome and Principe	12	2011.9	
	6	2005.7	2008-2009 Demographic and Health Survey [DHS Direct]
	3	2011.9	2012 Census [Others Direct]
	3	2008.3	2014 Multiple Indicator Cluster Survey [Others Direct]
Saudi Arabia	14	2017.5	
	3	1998.6	1999 Demographic Survey [Others Direct]
	3	2004.2	2004 Census [Others Direct]
	3	2009.8	2010 Census [Others Direct]
	2	2015.5	2016 Demographic Survey [Others Direct]
	3	2017.5	2018 Household Health Survey [Others Direct]
Senegal	78	2016.2	
	18	1991.8	1992-1993 Demographic and Health Survey [DHS Direct]
	3	2002.5	2002 Census [Others Direct]
	18	2004.1	2005 Demographic and Health Survey [DHS Direct]
	18	2009.7	2010-2011 Demographic and Health Survey [DHS Direct]
	3	2013.4	2013 Census [Others Direct]
	18	2016.2	2017 Demographic and Health Survey [DHS Direct]
Serbia	99	2021.5	
	60	2021.5	Recalculated based on WHO - adjusted for incompleteness [VR]

Continued on next page

Table 28 – continued from previous page

Country	# obs.	Most recent obs. year	Data series name [source type]
	39	2001.5	WHO Vital Registration (recalculated - unadjusted) [VR]
Seychelles	66	2016.5	
	3	1959.8	1960 Census [Others Direct]
	63	2016.5	WHO Vital Registration (recalculated - unadjusted) [VR]
Sierra Leone	57	2018.3	
	3	2004.4	2004 Census [Others Direct]
	18	2007.2	2008 Demographic and Health Survey [DHS Direct]
	18	2012.4	2013 Demographic and Health Survey [DHS Direct]
	18	2018.3	2019 Demographic and Health Survey [DHS Direct]
Singapore	96	2020.5	
	96	2020.5	WHO Vital Registration (recalculated - unadjusted) [VR]
Slovakia	156	2021.5	
	156	2021.5	WHO/Human Mortality Database Vital Registration (recalculated - unadjusted) [VR]
Slovenia	120	2021.5	
	120	2021.5	WHO/Human Mortality Database Vital Registration (recalculated - unadjusted) [VR]
Solomon Islands	3	2009.4	
	3	2009.4	2009 Census [Others Direct]
Somalia			
South Africa	99	2020.5	
	3	1997	1997 Household Survey [Others Direct]
	18	1997	1998 Demographic and Health Survey [DHS Direct]
	3	1997.2	2003 World Health Survey [Others Direct]
	3	2011.3	2011 Census [Others Direct]
	9	2014.4	2016 Demographic and Health Survey [DHS Direct]
	63	2020.5	Rapid Mortality Surveillance [VR]
South Sudan			
Spain	153	2020.5	
	153	2020.5	WHO/Human Mortality Database Vital Registration (recalculated - unadjusted) [VR]
Sri Lanka	75	2014.5	
	3	1997.2	2003 World Health Survey [Others Direct]
	72	2014.5	WHO Vital Registration (recalculated - unadjusted) [VR]
Saint Vincent and the Grenadines	84	2017.5	
	84	2017.5	WHO Vital Registration (recalculated - unadjusted) [VR]
State of Palestine			
Sudan	3	1992.8	
	3	1992.8	1993 Census [Others Direct]
Suriname	93	2019.5	
	93	2019.5	Recalculated based on WHO/UNPD - adjusted for incompleteness [VR]
Eswatini	27	2006.8	
	3	1996.9	1997 Census [Others Direct]
	3	1997.2	2003 World Health Survey [Others Direct]
	18	2005.5	2006-2007 Demographic and Health Survey [DHS Direct]

Continued on next page

Table 28 – continued from previous page

Country	# obs.	Most recent obs. year	Data series name [source type]
	3	2006.8	2007 Census [Others Direct]
Sweden	156	2021.5	
	156	2021.5	WHO/Human Mortality Database Vital Registration (recalculated - unadjusted) [VR]
Switzerland	156	2021.5	
	156	2021.5	WHO/Human Mortality Database Vital Registration (recalculated - unadjusted) [VR]
Syria	39	2010.5	
	39	2010.5	WHO Vital Registration (recalculated - unadjusted) [VR]
Tajikistan	63	2021.5	
	33	1999.5	Recalculated based on WHO/UNPD - adjusted for incompleteness [VR]
	30	2021.5	WHO Vital Registration (recalculated - unadjusted) [VR]
Tanzania	93	2014.6	
	3	1967.2	1967 Census [Others Direct]
	3	1973.3	1973 National Demographic Survey [Others Direct]
	3	1988.2	1988 Census [Others Direct]
	18	1995.5	1996 Demographic and Health Survey [DHS Direct]
	3	2002.2	2002 Census [Others Direct]
	18	2003.8	2004-2005 Demographic and Health Survey [DHS Direct]
	18	2008.9	2010 Demographic and Health Survey [DHS Direct]
	3	2010.4	2010-2011 National Panel Survey [Others Direct]
	6	2012.2	2012-2013 National Panel Survey [Others Direct]
	18	2014.6	2015-2016 Demographic and Health Survey [DHS Direct]
Thailand	93	2019.5	
	3	1995.5	1995-1996 Survey of Population Change [Others Direct]
	90	2019.5	Recalculated based on WHO - adjusted for incompleteness [VR]
Timor Leste	39	2015.2	
	3	1991.7	1997 Demographic and Health Survey [DHS Direct]
	18	2008.6	2009-2010 Demographic and Health Survey [DHS Direct]
	6	2014.5	2015 Census [Others Direct]
	12	2015.2	2016 Demographic and Health Survey [DHS Direct]
Togo	41	2012.8	
	3	1961.4	1961 Demographic survey [Others Direct]
	3	1981.4	1981 Census [Others Direct]
	18	1997.1	1998 Demographic and Health Survey [DHS Direct]
	17	2012.8	2013-2014 Demographic and Health Survey [DHS Direct]
Tonga	72	2018.5	
	3	2006.4	2006 Census [Others Direct]
	39	2005.5	Recalculated based on WHO - adjusted for incompleteness [VR]
	30	2018.5	WHO Vital Registration (recalculated - unadjusted) [VR]
Trinidad and Tobago	87	2017.5	
	87	2017.5	WHO/UNPD Vital Registration (recalculated - unadjusted) [VR]
Tunisia	37	2017.5	
	7	2000.2	2003 World Health Survey [Others Direct]
	21	2000.5	Recalculated based on WHO - adjusted for incompleteness [VR]

Continued on next page

Table 28 – continued from previous page

Country	# obs.	Most recent obs. year	Data series name [source type]
	9	2017.5	WHO Vital Registration (recalculated - unadjusted) [VR]
Turkey	33	2019.5	
	3	2002.7	2003 National Verbal Autopsy Survey [Others Direct]
	30	2019.5	WHO Vital Registration (recalculated - unadjusted) [VR]
Turkmenistan	81	2015.5	
	81	2015.5	Recalculated based on WHO/UNPD - adjusted for incompleteness [VR]
Turks and Caicos Islands	6	2002	
	6	2002	WHO Vital Registration (recalculated - unadjusted) [VR]
Tuvalu	8	2002	
	8	2002	WHO Vital Registration (recalculated - unadjusted) [VR]
Uganda	90	2015.4	
	18	1994.2	1995 Demographic and Health Survey [DHS Direct]
	18	1999.7	2000-2001 Demographic and Health Survey [DHS Direct]
	18	2005.3	2006 Demographic and Health Survey [DHS Direct]
	18	2010.4	2011 Demographic and Health Survey [DHS Direct]
	18	2015.4	2016 Demographic and Health Survey [DHS Direct]
Ukraine	153	2021.5	
	153	2021.5	WHO/Human Mortality Database Vital Registration (recalculated - unadjusted) [VR]
United Arab Emirates	3	1997.2	
	3	1997.2	2003 World Health Survey [Others Direct]
United Kingdom	93	2019.5	
	93	2019.5	WHO/Human Mortality Database Vital Registration (recalculated - unadjusted) [VR]
United States of America	153	2020.5	
	153	2020.5	WHO/Human Mortality Database Vital Registration (recalculated - unadjusted) [VR]
Uruguay	93	2020.5	
	93	2020.5	WHO Vital Registration (recalculated - unadjusted) [VR]
Uzbekistan	99	2021.5	
	99	2021.5	Recalculated based on WHO - adjusted for incompleteness [VR]
Vanuatu	3	2009.4	
	3	2009.4	2009 Census [Others Direct]
Venezuela (Bolivarian Republic of)	78	2016.5	
	45	2016.5	Recalculated based on WHO/UNPD - adjusted for incompleteness [VR]
	33	2000.5	WHO Vital Registration (recalculated - unadjusted) [VR]
Vietnam	39	2017.5	
	3	1978.8	1979 Census [Others Direct]
	3	1988.5	1989 Census [Others Direct]
	3	1998.7	1999 Census [Others Direct]
	3	1997.2	2003 World Health Survey [Others Direct]

Continued on next page

Table 28 – continued from previous page

Country	# obs.	Most recent obs. year	Data series name [source type]
	3	2006.8	2007 Population Change and Family Planning Survey [Others Direct]
	3	2007.5	2008 Population Change and Family Planning Survey [Others Direct]
	3	2010.8	2011 Population Change and Family Planning Survey [Others Direct]
	3	2011.8	2012 Population Change and Family Planning Survey [Others Direct]
	3	2012.8	2013 Population Change and Family Planning Survey [Others Direct]
	3	2013.5	2014 Intercensal Demographic Survey [Others Direct]
	3	2014.8	2015 Population Change and Family Planning Survey [Others Direct]
	3	2016.5	2017 Population Change and Family Planning Survey [Others Direct]
	3	2017.5	2018 Population Change and Family Planning Survey [Others Direct]
Yemen			
Zambia	111	2017.5	
	18	1995.5	1996 Demographic and Health Survey [DHS Direct]
	18	2000.8	2001-2002 Demographic and Health Survey [DHS Direct]
	18	2002.2	2003 World Health Survey [Others Direct]
	18	2006.2	2007 Demographic and Health Survey [DHS Direct]
	3	2010.3	2010 Census [Others Direct]
	18	2012.6	2013-2014 Demographic and Health Survey [DHS Direct]
	18	2017.5	2018 Demographic and Health Survey [DHS Direct]
Zimbabwe	130	2016.5	
	3	1992.1	1992 Census [Others Direct]
	18	1993.5	1994 Demographic and Health Survey [DHS Direct]
	3	1997.1	1997 Inter-Censal Demographic Survey [Others Direct]
	18	1998.7	1999 Demographic and Health Survey [DHS Direct]
	3	2002.1	2002 Census [Others Direct]
	16	2002.2	2003 World Health Survey [Others Direct]
	18	2004.6	2005-2006 Demographic and Health Survey [DHS Direct]
	18	2009.7	2010-2011 Demographic and Health Survey [DHS Direct]
	3	2012.1	2012 Census [Others Direct]
	9	2012.2	2014 Multiple Indicator Cluster Survey [Others Direct]
	18	2014.5	2015 Demographic and Health Survey [DHS Direct]
	3	2016.5	2017 Inter-Censal Demographic Survey [Others Direct]

References

[1] Barbieri M, Wilmoth JR, Shkolnikov VM, Glei D, Jasilionis D, Jdanov D, et al. Data resource profile: the human mortality database (HMD). International journal of epidemiology. 2015;44(5):1549-56.

[2] World Health Organization. WHO methods and data sources for country-level causes of death 2000â2016 (Global Health Estimates Technical Paper WHO/HIS/IER/GHE/2018.3). 2018. Available from: https://terrance.who.int/mediacentre/data/ghe/GlobalCOD_method_2000_2016.pdf.

[3] Moultrie T, Dorrington R, Hill A, Hill K, Timaeus I, Zaba B. Tools for Demographic Estimation. Paris: International Union for the Scientific Study of Population; 2013. Available from: http://demographicestimation.iussp.org.

[4] UN, DESA. World Population Prospects: The 2022 Revision; 2019. Available at http://esa.un.org/unpd/wpp/Download/Standard/Population/. Accessed 16 Feb 2023.

[5] Office of the Registrar General Census Commissioner, India. India Sample Registration System. 2022. Available from: https://censusindia.gov.in/census.website/node/294.

[6] Bangladesh Bureau of Statistics. Bangladesh Sample Vital Registration System. 2022. Available from: http://www.bbs.gov.bd/site/page/ef4d6756-2685-485a-b707-aa2d96bd4c6c/Vital-Statistics.

[7] Liu S, Wu X, Lopez AD, Wang L, Cai Y, Page A, et al. An integrated national mortality surveillance system for death registration and mortality surveillance, China. Bulletin of the World Health Organization. 2016;94(1):46.

[8] Dorrington R, Bradshaw D, Laubscher R, Nannan N. Rapid mortality surveillance report 2018. Medical Research Council, Burden of Disease Research Unit; 2020.

[9] Pedersen J, Liu J. Child mortality estimation: appropriate time periods for child mortality estimates from full birth histories. 2012.

[10] Hill K. Estimating Census and Death Registration Completeness. Asian and Pacific Population Forum. 1987;1(3):8-13.

[11] Dorrington R. The generalized growth balance method. In: Moultrie T, Dorrington R, Hill A, Hill K, Timæus I, Zaba B, editors. Tools for demographic estimation. Paris: International Union for the Scientific Study of Population; 2013. .

[12] Bennett N, Horiuchi S. Mortality estimation from registered deaths in less developed countries. Demography. 1984;21(2):217-34.

[13] Moultrie T, Dorrington R, Hill A, Hill K, Timæus I, Zaba B. Tools for demographic estimation. Paris: International Union for the Scientific Study of Population; 2013.

[14] Hill K, You D, Choi Y. Death distribution methods for estimating adult mortality: Sensitivity analysis with simulated data error. Demographic Research. 2009;21:235-54.

[15] Riffe T, Lima E, Queiroz B. DDM: Death Registration Coverage Estimation; 2017. R package version 1.0-0. Available from: https://CRAN.R-project.org/package=DDM.

[16] World Health Organization. Neonatal and Perinatal Mortality: Country, Regional and Global Estimates. World Health Organization; 2006.

[17] Masquelier B, Hug L, Sharrow D, You D, Mathers C, Gerland P, et al. Global, regional, and national mortality trends in youth aged 15–24 years between 1990 and 2019: a systematic analysis. The Lancet Global Health. 2021 Apr;9(4):e409-17.

[18] Murray C, Rajaratnam Jl, Marcus J, Laakso T, Lopez A. What Can We Conclude from Death Registration? Improved Methods for Evaluating Completeness. PLoS Med. 2010 04;7(4):e1000262.

[19] Sharrow D, Hug L, You D, Alkema L, Black R, Cousens S, et al. Global, regional, and national trends in under-5 mortality between 1990 and 2019 with scenario-based projections until 2030: a systematic analysis by the UN Inter-agency Group for Child Mortality Estimation. The Lancet Global Health. 2022 Feb;10(2):e195-206. Available from: https://doi.org/10.1016/s2214-109x(21)00515-5.

[20] Masquelier B, Hug L, Sharrow D, You D, Hogan D, Hill K, et al. Global, regional, and national mortality trends in older children and young adolescents (5-14 years) from 1990 to 2016: an analysis of empirical data. The Lancet Global Health. 2018 Sep;6(10):e1087-99.

[21] Hill K. Direct estimation of child mortality from birth histories. In: Moultrie T, Dorrington R, Hill A, Hill K, Timæus I, Zaba Z, editors. Tools for Demographic Estimation; 2013. .

[22] Hill K. Indirect estimation of child mortality. In: Moultrie T, Dorrington R, Hill A, Hill K, Timaeus I, Zaba B, editors. Tools for Demographic Estimation. UNFPA/IUSSP; 2013. .

[23] Alkema L, Chao F, You D, Pedersen J, Sawyer CC. National, regional, and global sex ratios of infant, child, and under-5 mortality and identification of countries with outlying ratios: a systematic assessment. The Lancet Global Health. 2014;2(9):e521-30.

[24] Simpson D, Rue H, Riebler A, Martins TG, Sørbye SH. Penalising model component complexity: A principled, practical approach to constructing priors. Statistical science. 2017;32(1):1-28.

[25] Nareeba T, Dzabeng F, Alam N, Biks GA, Thysen SM, Akuze J, et al. Neonatal and child mortality data in retrospective population-based surveys compared with prospective demographic surveillance: EN-INDEPTH study. Population health metrics. 2021;19:1-17.

[26] Masquelier B, Reniers G, Pison G. Divergences in trends in child and adult mortality in sub-Saharan Africa: survey evidence on the survival of children and siblings. Population studies. 2014;68(2):161-77.

[27] UN IGME. Levels & Trends in Child Mortality. Report 2022: Estimates Developed by the UN Inter-Agency Group for Child Mortality Estimation. 2023. Available from: https://childmortality.org.

[28] Rue H, Martino S, Chopin N. Approximate Bayesian Inference for Latent Gaussian Models Using Integrated Nested Laplace Approximations (with discussion). Journal of the Royal Statistical Society B. 2009;71:319-92.

[29] Rue H, Martino S, Lindgren F, Simpson D, Riebler A. R-INLA: Approximate Bayesian Inference Using Integrated Nested Laplace Approximations. Trondheim, Norway. 2013. Available at: http://www.r-inla.org.

[30] You D, Hug L, Ejdemyr S, Idele P, Hogan D, Mathers C, et al. Global, regional, and national levels and trends in under-5 mortality between 1990 and 2015, with scenario-based projections to 2030: a systematic analysis by the UN Inter-agency Group for Child Mortality Estimation. The Lancet. 2015;386(10010):2275-86.

[31] WHO. WHO methods and data sources for life tables 1990-2016; 2018. Global Health Estimates Technical Paper WHO/HIS/IER/GHE/2018.2, available at https://www.who.int/healthinfo/statistics/en/.

[32] Centre for Research on the Epidemiology of Disasters. EM-DAT: The International Disaster Database;. Available at https://www.emdat.be/.

[33] Raleigh C, Linke A, Hegre H, Karlsen J. Introducing ACLED: An Armed Conflict Location and Event Dataset. Journal of Peace Research. 2010 Sep;47(5):651-60. Available from: https://doi.org/10.1177/0022343310378914.

[34] Department of Peace and Conflict Research, Uppsala University. Uppsala Conflict Data Program;. Available at https://ucdp.uu.se/.

[35] Alkema L, Wong MB, Seah PR. Monitoring progress towards Millennium Development Goal 4: A call for improved validation of under-five mortality rate estimates. Statistics, Politics and Policy. 2012;3(2).

[36] Chao F, You D, Pedersen J, Hug L, Alkema L. National and regional under-5 mortality rate by economic status for low-income and middle-income countries: a systematic assessment. The Lancet Global Health. 2018;6(5):e535-47.

[37] Chao F, You D, Pedersen J, Hug L, Alkema L. Web appendix National and regional under-5 mortality rate by economic status for low-income and middle-income countries: a systematic assessment. 2018. DOI: 10.6084/m9.figshare.12442244. Available at https://ars.els-cdn.com/content/image/1-s2.0-S2214109X18300597-mmc1.pdf.

[38] Chao F, Gerland P, Cook AR, Alkema L. Systematic assessment of the sex ratio at birth for all countries and estimation of national imbalances and regional reference levels. Proceedings of the National Academy of Sciences. 2019;116(19):9303-11.

[39] Chao F, Gerland P, Cook AR, Alkema L. Web Appendix Systematic assessment of the sex ratio at birth for all countries and estimation of national imbalances and regional reference levels. 2019. DOI: 10.6084/m9.figshare.12442373. Available at https://www.pnas.org/content/pnas/suppl/2019/04/10/1812593116.DCSupplemental/pnas.1812593116.sapp.pdf.

[40] Chao F, Yadav AK. Levels and trends in the sex ratio at birth and missing female births for 29 states and union territories in India 1990–2016: A Bayesian modeling study. Foundations of Data Science. 2019;1(2):177-96.

[41] Chao F, Gerland P, Cook AR, Alkema L. Global estimation and scenario-based projections of sex ratio at birth and missing female births using a Bayesian hierarchical time series mixture model. Ann Appl Statist. 2021;15(3):1499-528.

[42] Jahagirdar D, Walters MK, Novotney A, Brewer ED, Frank TD, Carter A, et al. Global, regional, and national sex-specific burden and control of the HIV epidemic, 1990–2019, for 204 countries and territories: the Global Burden of Diseases Study 2019. The Lancet HIV. 2021;8(10):e633-51.

[43] Alkema L, Chou D, Hogan D, Zhang S, Moller AB, Gemmill A, et al. Global, regional, and national levels and trends in maternal mortality between 1990 and 2015, with scenario-based projections to 2030: a systematic analysis by the UN Maternal Mortality Estimation Inter-Agency Group. The lancet. 2016;387(10017):462-74.

[44] Sule WF, Oluwayelu DO, Hernández-Triana LM, Fooks AR, Venter M, Johnson N. Epidemiology and ecology of West Nile virus in sub-Saharan Africa. Parasites & vectors. 2018;11:1-10.

[45] Costa F, Hagan JE, Calcagno J, Kane M, Torgerson P, Martinez-Silveira MS, et al. Global morbidity and mortality of leptospirosis: a systematic review. PLoS neglected tropical diseases. 2015;9(9):e0003898.

[46] Fischinger S, Boudreau CM, Butler AL, Streeck H, Alter G. Sex differences in vaccine-induced humoral immunity. In: Seminars in immunopathology. vol. 41. Springer; 2019. p. 239-49.
